# Nickel-catalysed enantioselective alkene dicarbofunctionalization enabled by photochemical aliphatic C–H bond activation

**DOI:** 10.1038/s41929-024-01153-0

**Published:** 2024-04-29

**Authors:** Xia Hu, Iván Cheng-Sánchez, Wangqing Kong, Gary A. Molander, Cristina Nevado

**Affiliations:** 1 Department of Chemistry, University of Zurich, Zurich, Switzerland; 2 The Institute for Advanced Studies, Wuhan University, Wuhan, China; 3 Department of Chemistry, University of Pennsylvania, Philadelphia, PA USA

**Keywords:** Homogeneous catalysis, Asymmetric synthesis, Synthetic chemistry methodology

## Abstract

The development of novel strategies to rapidly construct complex chiral molecules from readily available feedstocks is a long-term pursuit in the chemistry community. Radical-mediated alkene difunctionalizations represent an excellent platform towards this goal. However, asymmetric versions remain highly challenging, and more importantly, examples featuring simple hydrocarbons as reaction partners are elusive. Here we report an asymmetric three-component alkene dicarbofunctionalization capitalizing on the direct activation of C(*sp*^3^)–H bonds through the combination of photocatalysed hydrogen atom transfer and nickel catalysis. This protocol provides an efficient platform for installing two vicinal carbon–carbon bonds across alkenes in an atom-economic fashion, providing a wide array of high-value chiral α-aryl/alkenyl carbonyls and phosphonates, as well as 1,1-diarylalkanes from ubiquitous alkane, ether and alcohol feedstocks. This method exhibits operational simplicity, broad substrate scope and excellent regioselectivity, chemoselectivity and enantioselectivity. The compatibility with bioactive motifs and expedient synthesis of pharmaceutically relevant molecules highlight the synthetic potential of this protocol.

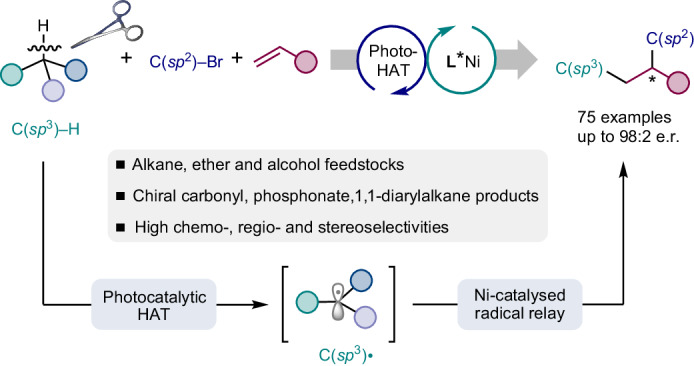

## Abstract (web summary)

Asymmetric versions of radical-mediated alkene difunctionalizations featuring hydrocarbon precursors are currently elusive. Here the authors report an asymmetric vicinal alkene dicarbofunctionalization based on the activation of C(*sp*^3^)–H bonds through the combination of photocatalysed hydrogen atom transfer and nickel catalysis.

## Main

The efficient assembly of high-added-value chiral molecules from readily available, inexpensive hydrocarbons with minimal waste generation represents a long-standing challenge for synthetic chemists^1,2^. Two major challenges have hampered the broad implementation of these processes: on the one hand, C(*sp*^3^)–H bonds present a much lower reactivity compared to most functional groups. Furthermore, the presence of multiple non-equivalent C(*sp*^3^)–H bonds requires exquisite control over the chemoselectivity, regioselectivity and stereoselectivity to attain the desired outcome^3,4^. Recently, hydrogen atom transfer (HAT) photocatalysis has emerged as a mild strategy for direct C(*sp*^3^)–H functionalizations^5^. Upon irradiation with light, the excited state of a photocatalyst can abstract hydrogen atoms from strong and inert C(*sp*^3^)–H bonds to produce nucleophilic carbon-centred radicals. Easy-to-synthesize *tetra*-*n*-butylammonium decatungstate (TBADT) and diaryl ketones have attracted particular interest as HAT photocatalysts owing to their ability to cleave electronically and sterically accessible C–H bonds with high regioselectivity^6–9^. As a result, synthetically useful C(*sp*^3^)–H functionalizations promoting the formation of new C–C (refs. ^10–16^), C–O (refs. ^17,18^), C–N (refs. ^19,20^), C–F (refs. ^21,22^) and C–S (ref. ^23^) bonds have been realized capitalizing on this strategy. More recently, the Kong^24^ and Molander^25^ groups have independently developed elegant methods for the racemic three-component dicarbofunctionalization of alkenes via photocatalysed HAT (photo-HAT)/nickel dual catalysis.

Catalytic three-component radical alkene difunctionalizations represent a straightforward and efficient approach for the rapid construction of molecular complexity, as two new functional groups can be simultaneously installed across the *π* system in a single operation^26–36^. However, controlling the enantioselectivity of the generated stereogenic centres remains a formidable challenge owing to the high reactivity and instability of the open shell radical intermediates participating in these transformations. Chiral copper^37–43^ and nickel ^44–52^ complexes have opened a robust platform for asymmetric radical conjunctive cross-coupling of olefins. For example, impressive progress has been made in nickel-catalysed asymmetric radical relayed reductive coupling^46–49^, in which C(*sp*^3^)-hybridized electrophiles such as alkyl halides and perfluoroalkyl halides have been employed as radical precursors. In addition, processes merging photoredox catalysis and nickel catalysis have enabled the asymmetric redox-neutral dicarbofunctionalization of alkenes with alkyltrifluoroborates as competent synthons for these protocols^50,51^. All of the aforementioned transformations strongly rely on the use of pre-activated radical precursors, use that not only leads to the generation of additional waste but also results in poor step economy and atom economy. To improve the synthetic efficiency and expand the pool of radical precursors, the direct use of abundant hydrocarbons as coupling partners in the asymmetric dicarbofunctionalization of alkenes represents a more ideal but also highly challenging approach.

**Fig. 1:**
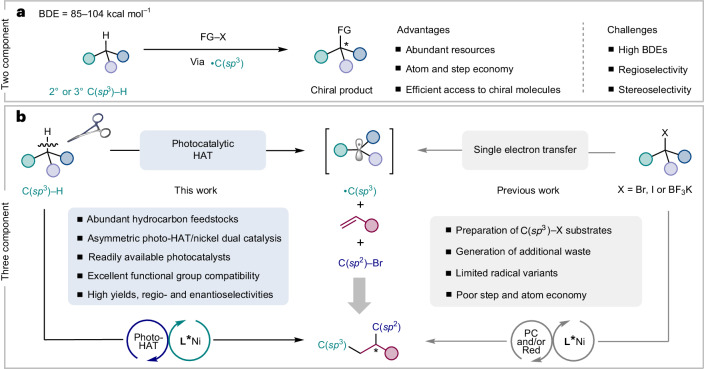
Development of asymmetric C(*sp*^3^)–H functionalizations and dicarbofunctionalization of alkenes. **a**, Asymmetric two-component C(*sp*^3^)–H functionalization through a synergistic HAT process and transition metal catalysis. **b**, Previous strategies and our design for asymmetric three-component radical dicarbofunctionalization of alkenes. BDE, bond dissociation energy; FG, functional group; PC, photocatalyst; Red, reductant.

Although synergistic HAT processes and transition metal catalysis have been used in the enantioselective arylation, alkylation, alkenylation, acylation and cyanation of C(*sp*^3^)–H bonds (Fig. 1a)^53–64^, the direct activation of C(*sp*^3^)–H bonds towards the asymmetric difunctionalization of alkenes is certainly underdeveloped (Fig. 1b). Two main challenges need to be addressed: first, the identification of a chiral ligand that can overcome competitive two-component side reactions while efficiently imparting absolute stereocontrol in the formation of the new stereogenic centres in the three-component process; and second, the implementation of reaction conditions wherein no competitive activation of distinct, even more activated C–H bonds present in the solvent and/or the participating reaction partners compromise the efficiency and selectivity of the overall process.

Here we show that the combination of a decatungstate or a diaryl ketone HAT photocatalyst with a chiral biimidazoline (BiIm) nickel catalyst enables the asymmetric dicarbofunctionalization of alkenes so that valuable α-aryl/alkenyl carbonyls and phosphonates as well as 1,1-diarylalkanes can be obtained in enantioenriched form through the regioselective activation of abundant hydrocarbons and their derivatives.

## Results

### Optimization of the reaction conditions

**Fig. 2:**
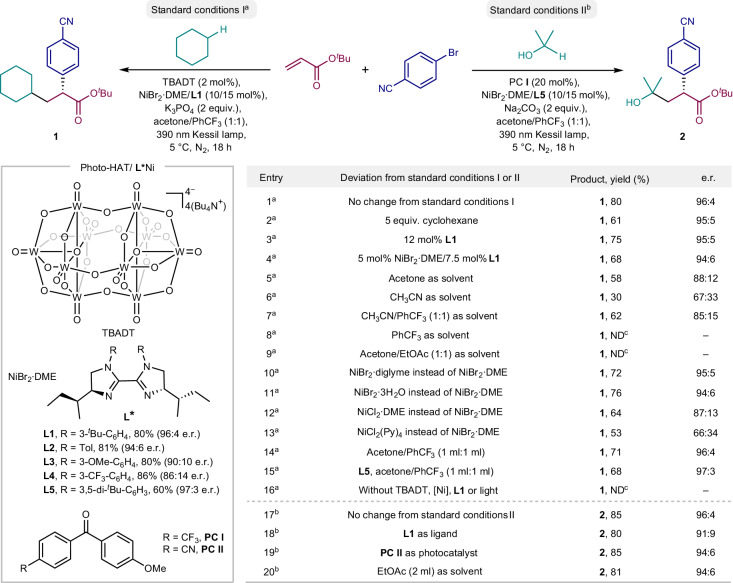
Optimization of reaction conditions. The optimization of reaction conditions. ^a^Standard reaction conditions I are as follows: cyclohexane (2 mmol, 10 equiv.), 4-bromobenzonitrile (0.2 mmol, 1 equiv.), *tert*-butyl acrylate (0.6 mmol, 3 equiv.), TBADT (0.004 mmol, 2 mol%), NiBr_2_**·**DME (0.02 mmol, 10 mol%), **L1** (0.03 mmol, 15 mol%), K_3_PO_4_ (0.4 mmol, 2 equiv.), acetone/PhCF_3_ (0.5 ml:0.5 ml), 40 W 390 nm Kessil lamp, N_2_, 5 °C, 18 h. ^b^Standard reaction conditions II are as follows: isopropanol (2 mmol, 10 equiv.), 4-bromobenzonitrile (0.2 mmol, 1 equiv.), *tert*-butyl acrylate (0.6 mmol, 3 equiv.), **PC I** (0.04 mmol, 20 mol%), NiBr_2_**·**DME (0.02 mmol, 10 mol%), **L5** (0.03 mmol, 15 mol%), Na_2_CO_3_ (0.4 mmol, 2 equiv.), acetone/PhCF_3_ (1.0 ml:1.0 ml), 40 W 390 nm Kessil lamp, N_2_, 5 °C, 18 h. Isolated yields. The e.r. values were determined by chiral HPLC. ^c^Detected by gas chromatography/mass spectrometry. ND, not detected.

Our investigation to identify suitable reaction conditions commenced with cyclohexane, *tert*-butyl acrylate and 4-bromobenzonitrile as model substrates under near-ultraviolet light irradiation (Kessil 40 W, 390 nm light-emitting diodes (LEDs); Fig. 2). After extensive evaluation of the reaction parameters, we found that the combination of TBADT (2 mol%), NiBr_2_·DME (DME, dimethoxyethane; 10 mol%), chiral BiIm **L1** (15 mol%) and potassium phosphate (K_3_PO_4_, 2 equiv.) provided *tert*-butyl (*R*)-2-(4-cyanophenyl)-3-cyclohexylpropanoate **1** in 80% isolated yield and 96:4 enantiomeric ratio (e.r.) in an acetone/trifluorotoluene (PhCF_3_) binary solvent system at 5 °C (Fig. 2, entry 1). The examination of different ligands revealed that the electronic and steric nature of the chiral BiIm template has a noticeable effect on the reaction efficiency (**L1**–**L5**). In general, BiIm ligands bearing electron-withdrawing groups on the aromatic ring result in higher yield but lower enantioselectivity (**L4**). Electron-rich and sterically demanding substituents on the nitrogen atom are beneficial for imparting stereocontrol, albeit with lower yield (**L5**). By comparison, 3-(*tert*-butyl)phenyl-substituted biimidazole **L1** exhibited the best compromise between reactivity and enantioselectivity. Reducing the amount of cyclohexane (Fig. 2, entry 2) or ligand (Fig. 2, entry 3), as well as decreasing the loading of nickel/**L1** (Fig. 2, entry 4) had a negative effect on the reaction efficiency. The dual acetone/PhCF_3_ solvent system was essential for the success of this transformation, as a substantial decrease in yield and enantioselectivity was observed when acetone, acetonitrile or CH_3_CN/PhCF_3_ was used as solvent (Fig. 2, entries 5–7). Furthermore, replacing the optimal solvent with PhCF_3_ or acetone/EtOAc resulted in complete failure of the reaction (Fig. 2, entries 8 and 9). Different nickel catalysts such as NiBr_2_·diglyme and NiBr_2_·3H_2_O delivered the product with slightly decreased yield and e.r. (Fig. 2, entries 10 and 11), while NiCl_2_·DME and NiCl_2_(Py)_4_ led to a dramatically reduced yield and enantioselectivity (Fig. 2, entries 12 and 13). The reaction still proceeded smoothly and provided a comparable e.r. upon dilution (Fig. 2, entries 14 and 15). Control experiments confirmed that photocatalyst, nickel, ligand and light were all necessary for a successful outcome (Fig. 2, entry 16). The absolute configuration of product **1** was unambiguously confirmed by X-ray diffraction analysis. In parallel, optimal conditions for isopropanol as radical precursor were sought. Gratifyingly, the desired three-component coupling product **2** could be obtained in 85% yield and 96:4 e.r. in the presence of diaryl ketone **PC I**, NiBr_2_·DME, **L5** and Na_2_CO_3_ under 40 W, 390 nm LED irradiation at 5 °C. The influence of ligand, photocatalyst and solvent is shown in Fig. 2, entries 18–20.

### Substrate scope

**Fig. 3:**
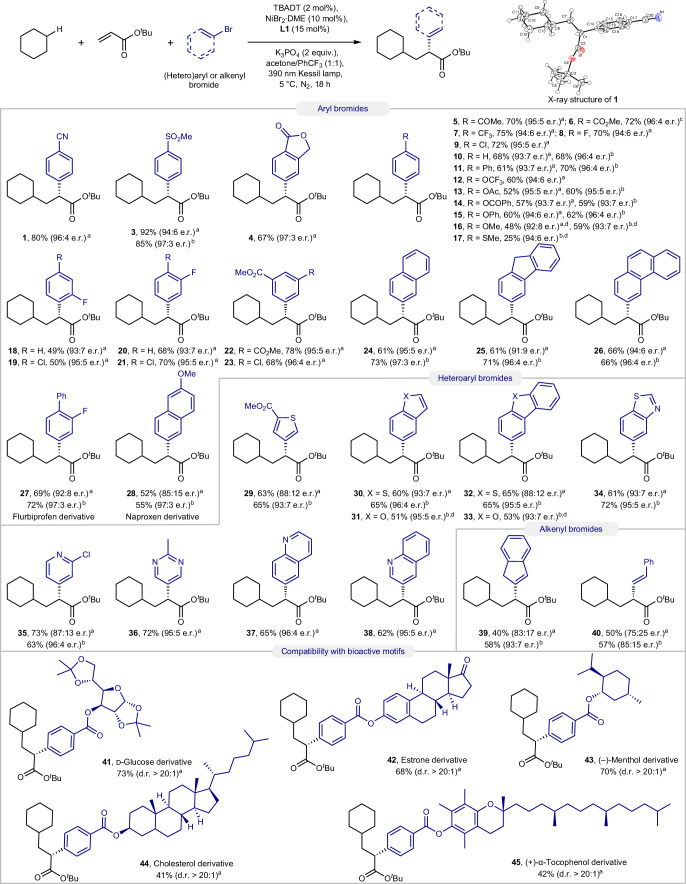
Scope of (hetero)aryl/alkenyl bromides. The reaction and conditions are shown at the top. ^a^Standard reaction conditions I are the same as in Fig. 2. ^b^**L5** was used as ligand, and acetone/PhCF_3_ (1 ml:1 ml) was used as solvent. ^c^NiBr_2_**·**3H_2_O was used instead of NiBr_2_**·**DME. ^d^Two 40 W 390 nm Kessil lamps were used. d.r., diastereomeric ratio.

With the optimized conditions in hand, the scope of this transformation was investigated next. We initially focused on examining a diverse array of aryl/alkenyl bromides (Fig. 3). Aryl bromides bearing cyanide (**1**), sulfone (**3**), lactone (**4**), ketone (**5**), ester (**6**), trifluomethyl (**7**), fluorine (**8**), chlorine (**9**), phenyl (**11**), trifluoromethoxy (**12**), acetoxy (**13**), benzoyloxy (**14**) and phenoxy (**15**) groups were all successfully converted to the corresponding dicarbofunctionalization products in good yields and high e.r. In general, electron-deficient aryl bromides exhibited improved efficacy compared to electron-neutral (**10**) and electron-rich ones. Aryl halides bearing strong electron-donating groups, such as 4-bromoanisole and 4-bromothioanisole, were not amenable to the standard reaction conditions. By increasing the light intensity in the reaction, the formation of the corresponding dicarbofunctionalization adducts could be successfully accomplished in these cases (**16** and **17**). The reactivities and enantioselectivities were not largely impacted by increased steric hindrance, because *ortho*- and *meta*-fluoro aryl bromides, as well as 2,4-, 3,4- and 3,5-disubstituted aryl bromides were still compatible with the established protocol (**18**–**23**). Polycyclic aryl bromides such as 2-bromonaphthalene, 3-bromo-9*H*-fluorene and 3-bromophenanthrene were also competent coupling partners (**24**–**26**). Moreover, derivatives of flurbiprofen (**27**) and naproxen (**28**), two well-known non-steroidal anti-inflammatory drugs (NSAIDs), could be readily synthesized applying our method. Heteroaryl bromides containing thiophene (**29**), benzothiophene (**30**), benzofuran (**31**), dibenzothiophene (**32**), dibenzofuran (**33**), benzothiazole (**34**), pyridines (**35**), pyrimidines (**36**) and quinolones (**37** and **38**), were successfully incorporated into this protocol, delivering the desired products with high to excellent enantioselectivities. Notably, these transformations displayed excellent chemoselectivity towards bromides in the presence of chlorides (**9**, **19**, **21**, **23**, **35**), paving the way for subsequent synthetic manipulations of the products. Alkenyl bromides such as 2-bromo-1*H*-indene (**39**) and β-bromostyrene (**40**) turned out to be viable partners despite a slight reduction of enantioselectivity. In some cases, especially for electron-rich (hetero)aryl and alkenyl bromides, using **L5** as the chiral ligand and diluting the reaction improved both the yields and enantioselectivities. Importantly, aryl bromides derived from d-glucose (**41**), estrone (**42**), (–)-menthol (**43**), cholesterol (**44**) and (+)-α-tocopherol (**45**) furnished the desired three-component coupling products in moderate to good yields with excellent diastereocontrol, despite the presence of several distinct C(*sp*^3^)–H bonds in the substrates, thus highlighting the chemoselectivity of the method and its applicability towards the synthesis of pharmaceutical and bioactive motifs.

**Fig. 4:**
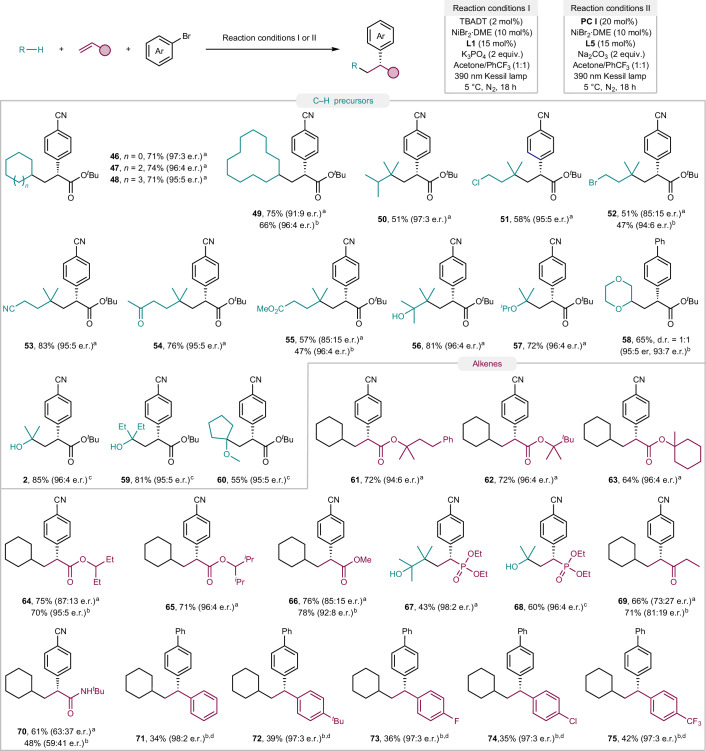
Scope of C–H precursors and alkenes. The reaction and conditions are shown at the top. ^a^Standard reaction conditions I are the same as in Fig. 2. ^b^**L5** was used as ligand, and acetone/PhCF_3_ (1 ml:1 ml) was used as solvent. ^c^Standard reaction conditions II are the same as in Fig. 2. ^d^Two 40 W 390 nm Kessil lamps were used.

The scope of C–H precursors was explored next (Fig. 4). As expected, cycloalkanes with various ring sizes such as cyclopentane (**46**), cycloheptane (**47**), cyclooctane (**48**) and cyclododecane (**49**) were well tolerated. In the last case, because of the lack of solubility of the reagent, an e.r. of 91:9 was obtained under standard reaction conditions, which was improved to 96:4 by increasing the amount of solvent and the use of **L5** as ligand. Interestingly, 2,3-dimethylbutane (**50**) was selectively activated on the secondary C–H bonds owing to the high bond dissociation energies of the primary C–H bonds. Remarkably, hydrocarbon derivatives bearing different electron-withdrawing groups (–Cl, –Br, –CN, –COMe, –COOMe) were successfully functionalized with excellent control of the regioselectivities and enantioselectivities (**51**–**55**). This result can be attributed to the hydridic nature of tertiary C–H bonds and the electrophilic nature of excited decatungstate^65^, thus providing products that are selectively functionalized distal to the electron-withdrawing moieties. Complete regioselectivity was also observed in the case of alcohols containing tertiary C–H bonds, with 2,3-dimethylbutan-2-ol affording product **56** in 81% yield and 96:4 e.r. The compatibility of diverse functional groups further emphasizes the advantages of this photo-HAT/nickel dual-catalysed strategy, as these types of alkyl radicals were inaccessible with previously developed radical precursors. This protocol is not restricted to electron-neutral, unactivated C–H systems. Ethers were regioselectively functionalized at the α-oxy position (**57** and **58**), which is consistent with the selectivity of decatungstate for more electron-rich C–H bonds. However, methyl *tert*-butyl ether, toluene and Boc-protected pyrrolidine were not able to participate in this three-component reaction, producing two-component aryl–alkyl cross-coupling products instead (Supplementary Table 9). The aforementioned reaction conditions were not suitable for the direct functionalization of hydrogen bonds in the α-position to hydroxy functions. Inspired by literature precedents^25^, we were delighted to find that isopropanol (**2**) and pentan-3-ol (**59**) participated in this transformation when benzophenone derivative **PC I** was used as the photocatalyst under modified reaction conditions. Furthermore, extraordinary regiocontrol was achieved in the presence of both tertiary and primary C–H bonds adjacent to the oxygen atom, as only the target product **60** was formed when cyclopentyl methyl ether was used as substrate. This result highlights the ability of diaryl ketone photocatalysts in site-selective activation.

We next turned our attention to expanding the scope with respect to alkenes (Fig. 4). A series of acrylates with different substituents was investigated, generating highly enantioenriched α-aryl ester products (**61**–**66**). Among them, a slight erosion in enantioselectivity was observed with less sterically hindered acrylate reactants, which was addressed by replacing ligand **L1** with **L5**. Pleasingly, vinyl phosphonates were also suitable coupling partners under either decatungstate or diaryl ketone photocatalytic conditions. Chiral organophosphonates were obtained in good yields and excellent enantioselectivities (**67** and **68**). Other electron-deficient alkenes were also explored. The α,β-unsaturated ketones as well as acrylamides were viable electron acceptors, although the products (**69** and **70**) were obtained with moderate enantioselectivity. Tertiary acrylamides furnished a trace amount of the desired dicarbofunctionalization adducts (Supplementary Table 9). Vinylarenes, a class of activated olefins that was not compatible in previous reports^49,50^, were tolerated in the present system. Styrenes bearing both electron-donating groups (*t*-Bu) as well as electron-withdrawing groups (F, Cl, CF_3_) could be engaged, under slightly modified reaction conditions, to deliver the corresponding chiral 1,1-diarylalkanes with excellent enantioselectivities (**71**–**75**).

### Synthetic applications

**Fig. 5:**
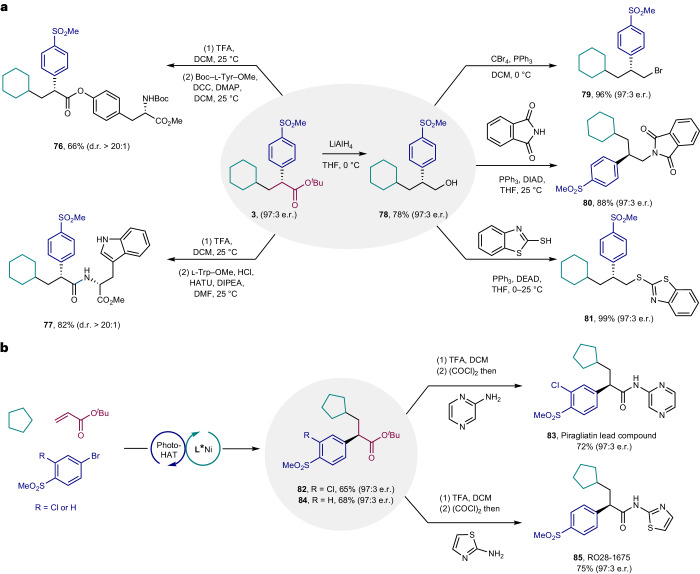
Synthetic applications. **a**, Derivatization of products. **b**, Synthesis of piragliatin lead compound and RO28-1675. TFA, trifluoroacetic acid; DCM, dichloromethane; DCC, *N*,*N*′-dicyclohexylcarbodiimide; DMAP, 4-dimethylaminopyridine; HATU, hexafluorophosphate azabenzotriazole tetramethyl uronium; DIPEA, *N*,*N*-diisopropylethylamine; DMF, dimethylformamide; THF, tetrahydrofuran; DEAD, diethyl azodicarboxylate; DIAD, diisopropyl azodicarboxylate.

The obtained α-aryl/alkenyl carbonyls and phosphonates as well as 1,1-diarylalkanes are often found as prevalent structural motifs in pharmacologically and biologically active molecules^66–69^. To further demonstrate the utility of this asymmetric multicomponent reaction, transformations of the products (Fig. 5a) and synthesis of pharmaceutically relevant molecules were performed. Specifically, through acidic hydrolysis followed by esterification or amidation processes, l-tyrosine and l-tryptophan derivatives (**76** and **77**) were obtained with high levels of diastereocontrol, which indicated the potential of this method for rapidly accessing complex amino acid derivatives. In addition, the reduction of α-aryl ester **3** was successfully accomplished by treatment with LiAlH_4_, delivering alcohol **78** with excellent stereofidelity (97:3 e.r.). Because alcohols are versatile intermediates in organic synthesis, several derivatizations of **78** were also carried out. Nucleophilic substitution reactions converted **78** into the corresponding β-aryl bromide (**79**), β-aryl amide (**80**) and β-aryl thiol (**81**) in high yields and enantioselectivities. To illustrate its practical value further, the method was applied to a concise synthesis of two glucokinase activators. The synthetic route towards piragliatin lead compound is shown in Fig. 5b (top). The combination of commercially available cyclopentane, *tert*-butyl acrylate and 4-bromo-2-chloro-1-(methylsulfonyl)benzene produced the corresponding three-component cross-coupling product **83** in 65% yield and 97:3 e.r. Hydrolysis of **82** with trifluoroacetic acid followed by amidation with 2-aminopyrazine offered piragliatin lead compound **83** in 72% yield and 97:3 e.r. Compared to previous reports, this method simplifies the operation steps by using cyclopentane instead of alkyltrifluoroborate as the synthetic precursors^50^. Similarly, enantioenriched glucokinase activator RO28-1675 (**85**, 97:3 e.r.) was successfully synthesized by using the abovementioned three-step synthetic procedure (Fig. 5b, bottom).

### Mechanistic studies

**Fig. 6:**
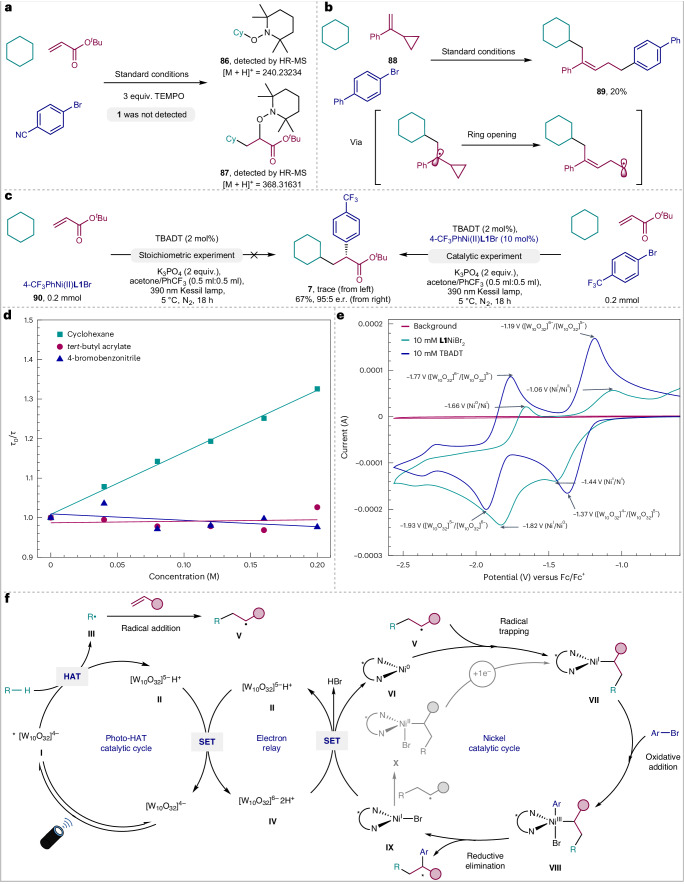
Mechanistic studies and proposed mechanism. **a**, Radical trapping experiment. **b**, Radical clock experiment. **c**, Stoichiometric and catalytic experiments with ArNi(II)**L1**Br complex. **d**, Stern–Volmer studies of TBADT with cyclohexane, *tert*-butyl acrylate and 4-bromobenzonitrile. **e**, Cyclic voltammogram studies of TBADT and **L1**NiBr_2_. **f**, Proposed mechanism. SET, single-electron transfer; *τ*, measured lifetime of excited photocatalyst with quencher; *τ*_0_, lifetime of the excited photocatalyst without quencher.

Several experiments were designed to probe the reaction mechanism of this dual-catalytic transformation. The desired alkylarylation reaction was completely suppressed by adding 2,2,6,6-tetramethyl-1-piperidinyloxy (TEMPO; 3.0 equiv.). TEMPO adducts **86** and **87** were detected by high-resolution mass spectrometry (HR-MS) in the mixture (Fig. 6a), thus demonstrating the intermediacy of an alkyl radical along the reaction pathway. Next, radical clock experiments were conducted with cyclopropyl alkene **88** under the standard reaction conditions, affording product **89** in 20% yield through a sequential radical addition, ring opening and arylation process (Fig. 6b). Product deracemization or Giese-type addition of radicals to the double bond followed by enantioselective α-arylation were ruled out based on controlled experiments (Supplementary Discussion). Subsequently, the reactivity and catalytic efficiency of presynthesized 4-CF_3_C_6_H_4_Ni(II)**L1**Br complex **90** was further investigated. Only a trace amount of **7** was detected with stoichiometric complex **90**, cyclohexane and *tert*-butyl acrylate. One possible reason is that complex **90** is unstable in the absence of aryl bromides, and it possesses strong visible-light absorption properties^54^. By contrast, using 10 mol% of complex **90** instead of NiBr_2_**·**DME/**L1** as catalyst in the presence of aryl bromides, product **7** was obtained in 67% yield with an identical e.r. (95:5; Fig. 6c). Taken together, these results indicate that the putative aryl-Ni(II) complex might not be a productive intermediate in the main catalytic cycle. In addition, laser flash photolysis experiments were performed to study the quenching of the excited state of TBADT (TBADT*) in the presence of increasing concentrations of cyclohexane, *tert*-butyl acrylate and 4-bromobenzonitrile, respectively. A clear decay of TBADT* was observed in the presence of cyclohexane following a linear Stern–Volmer behaviour (bimolecular rate constant *k* = 3.14 × 10^7^ M^−1^ s^−1^). By contrast, *tert*-butyl acrylate and 4-bromobenzonitrile were not able to quench TBADT* (Fig. 6d). These results confirm the activation of cyclohexane by the excited state of the photocatalyst to form carbon radicals, in line with the radical trapping and clock experiments summarized in Fig. 6a,b.

On the basis of the aforementioned mechanistic studies and precedents in the literature^24^, a plausible mechanism for this TBADT/nickel dual-catalysed enantioselective alkene dicarbofunctionalization can be proposed (Fig. 6f). Excited-state tetrabutylammonium decatungstate **I** would be formed under photoexcitation conditions, enabling the abstraction of a hydrogen atom from C(*sp*^3^)–H nucleophiles. This process generates singly reduced decatungstate **II** and carbon-centred radical **III**. Disproportionation of **II** provides doubly reduced decatungstate **IV** and regenerates ground-state TBADT. In parallel, radical addition of **III** to an olefin would form adduct **V**, which can be captured by Ni(0) species **VI** to furnish alkyl-Ni(I) intermediate **VII**. Subsequent oxidative addition of alkenyl bromide to Ni(I) species **VII** generates alkyl-Ni(III)-aryl intermediate **VIII**. Reductive elimination produces the desired dicarbofunctionalization product as well as Ni(I) species **IX**. Both catalytic cycles converge to completion through final single-electron transfer between this Ni(I) species and doubly reduced TBADT **IV** to regenerate the active Ni(0) catalyst **VI** and singly reduced TBADT **II**. This process is supported by cyclic voltammetry studies (Fig. 6e), as the reductive potential of [W_10_O_32_]^5–^/[W_10_O_32_]^6–^ (*E*_1/2_ = –1.85 V versus Fc/Fc^+^ in MeCN, reductive peak observed at –1.93 V) is more negative than that of Ni^I^/Ni^0^ (*E*_1/2_ = –1.74 V versus Fc/Fc^+^ in MeCN, reductive peak observed at –1.82 V). A Ni^I^/Ni^II^/Ni^III^ catalytic sequence is also possible (Fig. 6f, grey). Alternatively, Ni(I) species **VII** could be obtained through the combination of Ni(I) species **IX** with radical adduct **V**, followed by the subsequent single-electron reduction of alkyl-Ni(II) intermediate **X**.

## Conclusions

In summary, the combination of photoredox-mediated HAT with a nickel-catalysed radical relay has enabled a highly enantioselective three-component difunctionalization of alkenes. Unactivated hydrocarbons can be directly used as coupling agents without the requirement of pre-activated radical precursors, making this strategy more attractive for step-economical and atom-economical synthesis. Site-selective activation and the conversion of alkanes, ethers and alcohols were achieved by employing decatungstate or diaryl ketone photocatalysts. The method exhibits mild conditions, broad substrate scope and excellent regioselectivity, chemoselectivity and enantioselectivity, providing a versatile synthesis of value-added enantioenriched α-aryl/alkenyl carbonyls/phosphonates and 1,1-diarylalkanes. The synthetic potential of this method has been demonstrated by derivatization of the products and the synthesis of medicinally relevant molecules. We believe that the lessons obtained here will inspire the development of more asymmetric multicomponent reactions enabled by C(*sp*^3^)–H activation in the future.

## Methods

### Method A

In a nitrogen-filled glove box, a reaction vial (5 ml) equipped with a stirring bar was charged with TBADT (0.004 mmol, 2 mol%), NiBr_2_·DME (0.02 mmol, 10 mol%), (4 *S*,4′*S*)-4,4′-di((*S*)-*sec*-butyl)-1,1′-bis(3-(*tert*-butyl)phenyl)-4,4′,5,5′-tetrahydro-1*H*,1′*H*-2,2′-biimidazole (**L1**; 0.03 mmol, 15 mol%) or (4 *S*,4′*S*)-4,4′-di((*S*)-*sec*-butyl)-1,1′-bis(3,5-di-*tert*-butylphenyl)-4,4′,5,5′-tetrahydro-1*H*,1′*H*-2,2′-biimidazole (**L5**; 0.03 mmol, 15 mol%), anhydrous K_3_PO_4_ (0.4 mmol, 2 equiv.), aryl bromide (0.2 mmol, 1 equiv.), C–H radical precursor (2 mmol, 10 equiv.), alkene (0.6 mmol, 3 equiv.), dry acetone (0.5 ml, 1.0 ml when **L5** was used) and dry α,α,α-trifluorotoluene (0.5 ml, 1.0 ml when **L5** was used). The vial was sealed and removed from the glove box. The reaction mixture was prestirred for 20 min, then irradiated with a Kessil PR160 390 nm lamp at 5 °C with a distance of ~3 cm from the surface of the reaction vial. After 18 h of irradiation, the resulting mixture was passed through a pipette plug of Celite and silica gel and eluted with EtOAc. After concentration under reduced pressure, the crude mixture was purified by chromatography on silica gel with hexane/EtOAc mixtures to give the corresponding products.

### Method B

In a nitrogen-filled glove box, a reaction vial (5 ml) equipped with a stirring bar was charged with (4-methoxyphenyl)(4-(trifluoromethyl)phenyl)methanone (**PC I**; 0.04 mmol, 20 mol%), NiBr_2_·DME (0.02 mmol, 10 mol%), **L5** (0.03 mmol, 15 mol%), anhydrous Na_2_CO_3_ (0.4 mmol, 2 equiv.), aryl bromide (0.2 mmol, 1 equiv.), C–H radical precursor (2 mmol, 10 equiv.), alkene (0.6 mmol, 3 equiv.), dry acetone (1.0 ml) and dry α,α,α-trifluorotoluene (1.0 ml). The vial was sealed and removed from the glove box. The reaction mixture was prestirred for 20 min, then irradiated with a Kessil PR160 390 nm lamp at 5 °C with a distance of ~3 cm from the surface of the reaction vial. After 18 h of irradiation, the resulting mixture was passed through a pipette plug of Celite and silica gel and eluted with EtOAc. After concentration under reduced pressure, the crude mixture was purified by chromatography on silica gel with hexane/EtOAc mixtures to give the corresponding products.

### Supplementary information

Supplementary Information Experimental procedures, characterization data, NMR spectra, HPLC traces and crystallographic data.

Supplementary Data Crystallographic data for compound **1**; CCDC reference 2268422.

Supplementary Data CheckCIF report for compound **1**.

## Data Availability

The X-ray crystallographic coordinates for compound **1** reported in this article have been deposited at the Cambridge Crystallographic Data Centre (CCDC), under deposition number 2268422. These data can be obtained free of charge from the CCDC via www.ccdc.cam.ac.uk/data_request/cif. The authors declare that the data supporting the findings of this study are available within the article and its Supplementary Information. Data are available from the corresponding author upon request.
