# Supplementary material for: Nickel-catalysed enantioselective alkene dicarbofunctionalization enabled by photochemical aliphatic C–H bond activation
Source: Nat Catal. 2024 Apr 29;7(6):655–65. doi: 10.1038/s41929-024-01153-0 (PMC11208155; doi:10.1038/s41929-024-01153-0)
Supplement: Supplementary file 1 — Experimental procedures, characterization data, NMR spectra, HPLC traces and crystallographic data. [file 41929_2024_1153_MOESM1_ESM.pdf]

nature catalysis

Article

https://doi.org/10.1038/s41929-024-01153-0

Nickel-catalysed enantioselective alkene dicarbofunctionalization enabled by photochemical aliphatic C–H bond activation

In the format provided by the authors and unedited

Supplementary Information

Contents

Supplementary Methods......2

General Information......2

Experimental Procedures for the Asymmetric Alkene Dicarbofunctionalization......2

Characterization Data of Asymmetric Alkene Dicarbofunctionalization Products......3

Experimental Procedures and Characterization Data for Synthetic Applications......78

Experimental Procedures for Radical Trapping Experiment......89

Experimental Procedures for Radical Clock Experiment......89

Synthesis and Characterization of Aryl–Ni(II) Bromide Complex **90**......90

Stoichiometric Experiment with Aryl–Ni(II) Bromide Complex **90**......90

Catalytic Experiment with Aryl–Ni(II) Bromide Complex **90**......91

General Procedures for Laser Flash Photolysis......91

General Procedures for Cyclic Voltammetry Studies......92

General Procedures for X-Ray Diffraction Analysis of Compound **1**......92

Supplementary Discussion......94

Supplementary Tables......96

Supplementary Figures......106

Supplementary References......203

Supplementary Methods

General Information

Unless otherwise stated, reactions were carried out using dry solvents under a nitrogen atmosphere. Starting materials were purchased from Aldrich, Fluka and TCI. Conversion was monitored by thin layer chromatography (TLC) using Merck TLC silica gel 60 F254 and visualized by UV light at 254 nm. Flash column chromatography was performed over silica gel (230-400 mesh). Photochemical experiments have been performed using 40 W Kessil® PR160 390nm lamp. All NMR spectra were recorded on AV2-400 Bruker spectrometers. Chemical shifts are given in ppm and the spectra are calibrated using the residual chloroform signals (7.26 ppm for ^{1}H NMR and 77.0 ppm for ^{13}C NMR) and the residual dichloromethane signals (2.05 ppm for ^{1}H NMR and 29.84 ppm, for ^{13}C NMR). Multiplicities are abbreviated as follows: singlet (s), doublet (d), triplet (t), quartet (q), doublet-doublet (dd), septet (sept), multiplet (m), and broad (b). Infrared spectra were recorded on a JASCO FT/IR - 4100 spectrometer. Absorptions are reported in wavenumber (cm^{-1}). High-resolution electrospray ionization and electron impact mass spectrometry was performed on a Finnigan MAT 900 (Thermo Finnigan, San Jose, CA; USA) double focusing magnetic sector mass spectrometer. Ten spectra were acquired. A mass accuracy ⩽ 2 ppm was obtained in the peak matching acquisition mode by using a solution containing 2 <l PEG200, 2 <l PPG450, and 1.5 mg NaOAc (all obtained from Sigma-Aldrich, CH-Buchs) dissolved in 100 mL of MeOH (HPLC Supra grade, Scharlau, E-Barcelona) as internal standard. Melting points were measured on melting point operators: MPM-MHV from Müller + Krempel AG. The enantiomeric ratios were determined by chiral HPLC analysis performed on JASCO HPLC system equipped with a PU-980 pump, a UV-970 detector, measured on a chiral column. Optical rotations were measured on a JASCO P-1010 polarimeter.

Experimental Procedures for the Asymmetric Alkene Dicarbofunctionalization

General Procedure A (GP-A):

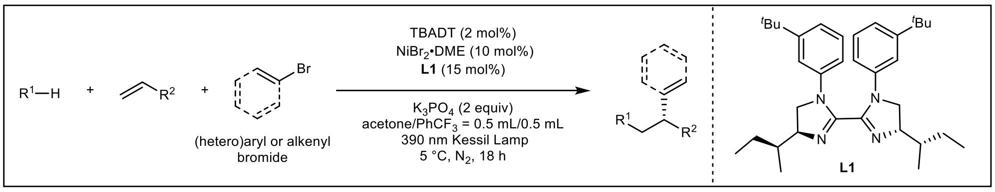

GP-A: In a nitrogen-filled glove box, a reaction vial (5 mL) equipped with a stirring bar was charged with tetrabutylammonium decatungstate (TBADT, 0.004 mmol, 2 mol%), $NiBr_2$·DME (0.02 mmol, 10 mol%), (4*S*,4'*S*)-4,4'-di((*S*)-*sec*-butyl)-1,1'-bis(3-(*tert*-butyl)phenyl)-4,4',5,5'-tetrahydro-1H,1'H-2,2'-biimidazole (**L1**, 0.03 mmol, 15 mol%), anhydrous K_3PO_4 (0.4 mmol, 2 equiv), aryl bromide (0.2 mmol, 1 equiv), C-H radical precursor (2 mmol, 10 equiv), alkene (0.6 mmol, 3 equiv), dry acetone (0.5 mL), and dry α,α,α-trifluorotoluene (0.5 mL). The vial was sealed and removed from the glovebox. The reaction mixture was pre-stirred for 20 min, then irradiated with a Kessil® PR160-390nm lamp at 5 ^{o}C with a distance of ~3 cm from the surface of the reaction vial. After 18 h of irradiation, the resulting mixture was passed through a pipette plug of Celite/silica gel and eluted with EtOAc. After concentration under reduced pressure, the crude mixture was purified by chromatography on silica gel with hexane: EtOAc mixtures to give the corresponding products.

General Procedure B (GP-B):

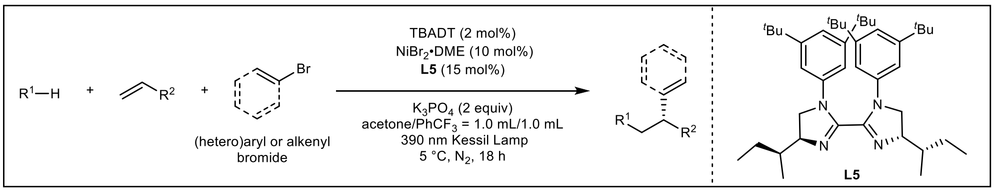

GP-B: In a nitrogen-filled glove box, a reaction vial (5 mL) equipped with a stirring bar was charged with tetrabutylammonium decatungstate (TBADT, 0.004 mmol, 2 mol%), $NiBr_2$·DME (0.02 mmol, 10 mol%), (4*S*,4'*S*)-4,4'-di((*S*)-*sec*-butyl)-1,1'-bis(3,5-di-*tert*-butylphenyl)-4,4',5,5'-tetrahydro-1H,1'H-2,2'-biimidazole (**L5**, 0.03 mmol, 15 mol%), anhydrous K_3PO_4 (0.4 mmol, 2 equiv), aryl bromide (0.2 mmol, 1 equiv), C-H radical precursor (2 mmol, 10 equiv), alkene (0.6 mmol, 3 equiv), dry acetone (1.0 mL), and dry α,α,α-trifluorotoluene (1.0 mL). The vial was sealed and removed from the glovebox. The reaction mixture was pre-stirred for 20 min, then irradiated with a Kessil® PR160-390nm lamp at 5 °C with a distance of ~3 cm from the surface of the reaction vial. After 18 h of irradiation, the resulting mixture was passed through a pipette plug of Celite/silica gel and eluted with EtOAc. After concentration under reduced pressure, the crude mixture was purified by chromatography on silica gel with hexane: EtOAc mixtures to give the corresponding products.

General Procedure C (GP-C):

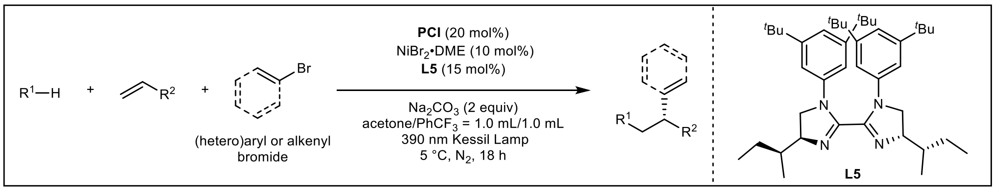

GP-C: In a nitrogen-filled glove box, a reaction vial (5 mL) equipped with a stirring bar was charged with (4-methoxyphenyl)(4-(trifluoromethyl)phenyl)methanone (**PCI**, 0.04 mmol, 20 mol%), $NiBr_2$·DME (0.02 mmol, 10 mol%), (4*S*,4'*S*)-4,4'-di((*S*)-*sec*-butyl)-1,1'-bis(3,5-di-*tert*-butylphenyl)-4,4',5,5'-tetrahydro-1H,1'H-2,2'-biimidazole (**L5**, 0.03 mmol, 15 mol%), anhydrous Na_2CO_3 (0.4 mmol, 2 equiv), aryl bromide (0.2 mmol, 1 equiv), C-H radical precursor (2 mmol, 10 equiv), alkene (0.6 mmol, 3 equiv), dry acetone (1.0 mL), and dry α,α,α-trifluorotoluene (1.0 mL). The vial was sealed and removed from the glovebox. The reaction mixture was pre-stirred for 20 min, then irradiated with a Kessil® PR160-390nm lamp at 5 °C with a distance of ~3 cm from the surface of the reaction vial. After 18 h of irradiation, the resulting mixture was passed through a pipette plug of Celite/silica gel and eluted with EtOAc. After concentration under reduced pressure, the crude mixture was purified by chromatography on silica gel with hexane: EtOAc mixtures to give the corresponding products.

Characterization Data of Asymmetric Alkene Dicarbofunctionalization Products

***tert*-Butyl (*R*)-2-(4-cyanophenyl)-3-cyclohexylpropanoate (1)**

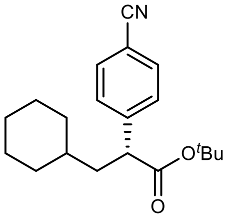

Prepared by **GP-A**. White solid, 50.4 mg, 80% yield. m.p. = 89-90 °C; ^{1}H NMR (400 MHz, $CDCl_3$) δ 7.62 – 7.56 (m, 2H), 7.43 – 7.37 (m, 2H), 3.62 (t, 1H, *J* = 8 Hz), 1.92 (ddd, *J* = 13.8, 8.3, 7.1 Hz, 1H), 1.75 – 1.52 (m, 6H), 1.37 (s, 9H), 1.18 – 1.04 (m, 4H), 0.99 – 0.80 (m, 2H); ^{13}C NMR (101 MHz, $CDCl_3$) δ 172.3, 145.4, 132.2, 128.7, 118.8, 110.8, 81.1, 50.0, 40.9, 35.4, 33.1, 32.9, 27.8, 26.3, 26.04, 26.01. IR (film): ν (cm^{-1}) 2982, 2916, 2848, 2230, 1718, 1605, 1505, 1449, 1366, 1355, 1347, 1337, 1216, 1175, 1142, 1085, 1023, 973, 902,

894, 875, 864, 842, 836, 759, 697, 574, 552, 485; HR-MS (ESI) m/z calcd for $C_{20}H_{27}NNaO_2^+$ [M+Na$^+$] 336.19340, found 336.19286; $[\alpha]_D^{22.1}$ = -18.2 (c = 0.1, $CHCl_3$); HPLC conditions: IC column, hexane/2-propanol = 99/1, flow rate = 0.5 mL/min, λ = 230 nm, t_R = 32.2 min (minor), t_R = 34.2 min (major), 96:4 er.

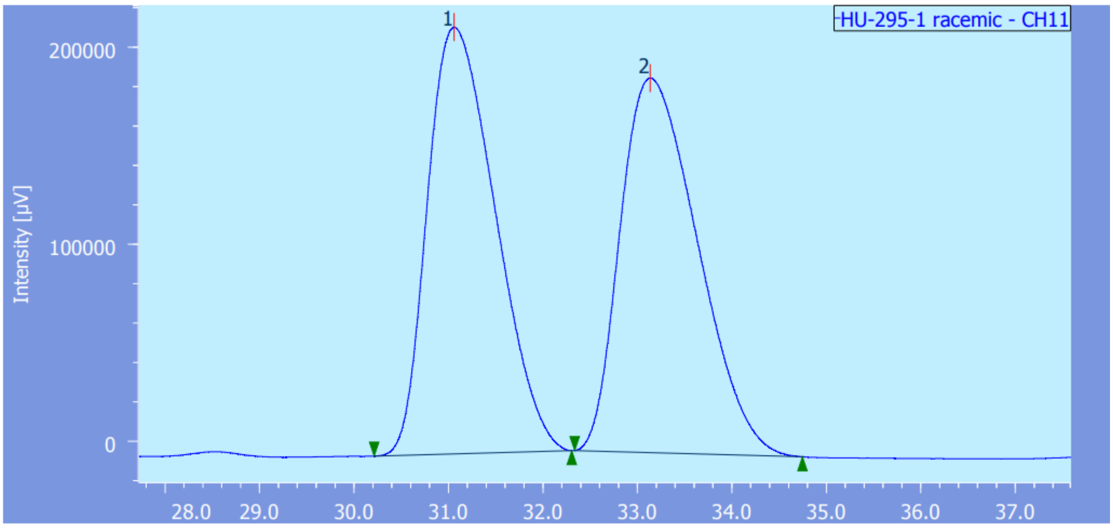

Decision

#	Peak Name	CH	tR [min]	Area [µV·sec]	Height [µV]	Area%	Height%	Quantity	NTP	Resolution	Symmetry Factor	Warning
1	Unknown	11	31.060	10801689	216355	49.966	53.255	N/A	8278	1.424	1.358	
2	Unknown	11	33.133	10816347	189905	50.034	46.745	N/A	7270	N/A	1.404	

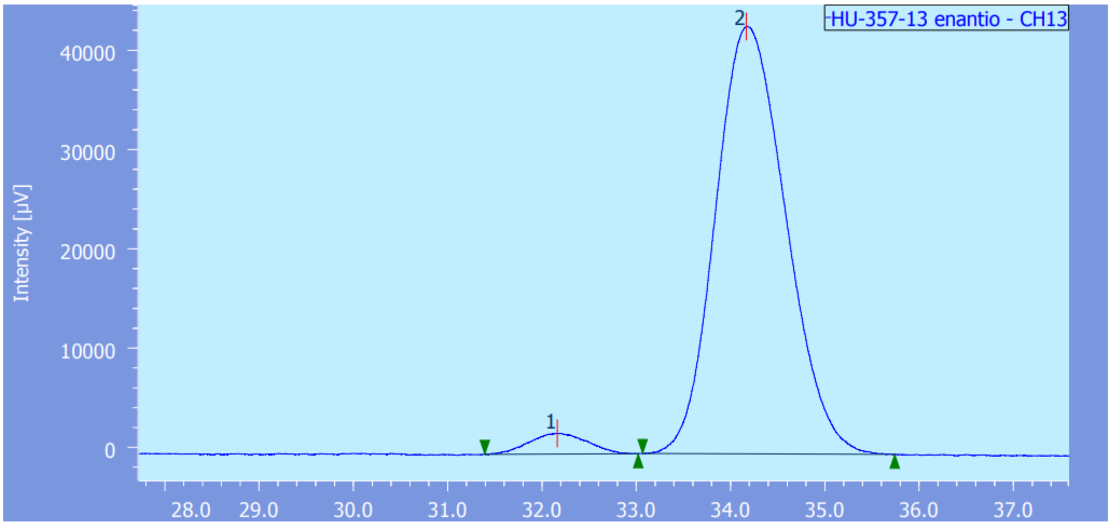

Decision

#	Peak Name	CH	tR [min]	Area [µV·sec]	Height [µV]	Area%	Height%	Quantity	NTP	Resolution	Symmetry Factor	Warning
1	Unknown	13	32.160	89819	2069	3.866	4.590	N/A	11236	1.547	1.003	
2	Unknown	13	34.167	2233527	43006	96.134	95.410	N/A	9694	N/A	1.165	

***tert*-Butyl (*R*)-2-(4-cyanophenyl)-4-hydroxy-4-methylpentanoate (2)**

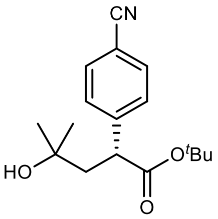

Prepared by **GP-C**. White solid, 49.4 mg, 85% yield. m.p. = 106-107 °C; ^{1}H NMR (400 MHz, $CDCl_3$) δ 7.62 – 7.57 (m, 2H), 7.44 – 7.38 (d, *J* = 8.2 Hz, 2H), 3.79 (dd, *J* = 9.9, 3.2 Hz, 1H), 2.51 (dd, *J* = 14.3, 9.9 Hz, 1H), 1.69 (dd, *J* = 14.3, 3.2 Hz, 1H), 1.37 (s, 9H), 1.26 (s, 3H), 1.24 (s, 3H); ^{13}C NMR (101 MHz, $CDCl_3$) δ 172.9, 146.3, 132.4, 128.5, 118.7, 110.9, 81.5, 70.4, 48.6, 46.4, 30.3, 29.0, 27.8. IR (film): ν (cm^{-1}) 3517, 2976, 2924, 2874, 2230, 1724, 1604, 1392, 1366, 1340, 1252, 1215, 1143, 842, 755, 567; HR-MS (ESI) m/z calcd for

$C_{17}H_{23}NNaO_3^+$ [M+Na$^+$] 312.15701, found 312.15684; $[\alpha]_D^{24.0}$ = -23.8 (c = 0.1, $CHCl_3$); HPLC conditions: IC column, hexane/2-propanol = 90/10, flow rate = 0.5 mL/min, λ = 250 nm, t_R = 18.3 min (minor), t_R = 20.2 min (major), 96:4 er.

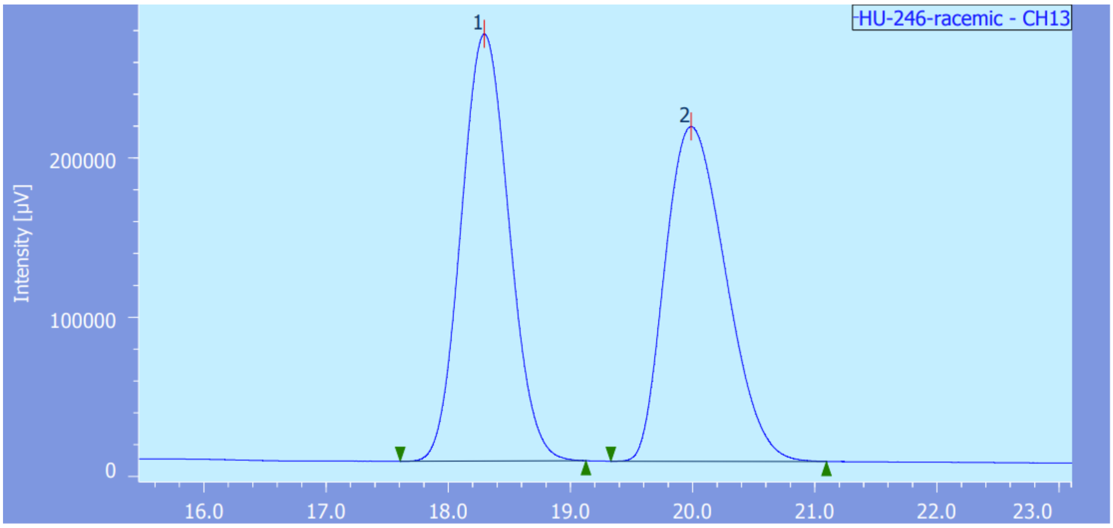

Decision

#	Peak Name	CH	tR [min]	Area [µV·sec]	Height [µV]	Area%	Height%	Quantity	NTP	Resolution	Symmetry Factor	Warning
1	Unknown	13	18.293	7309764	268165	50.192	56.059	N/A	10126	2.038	1.090	
2	Unknown	13	19.987	7253824	210198	49.808	43.941	N/A	7246	N/A	1.289	

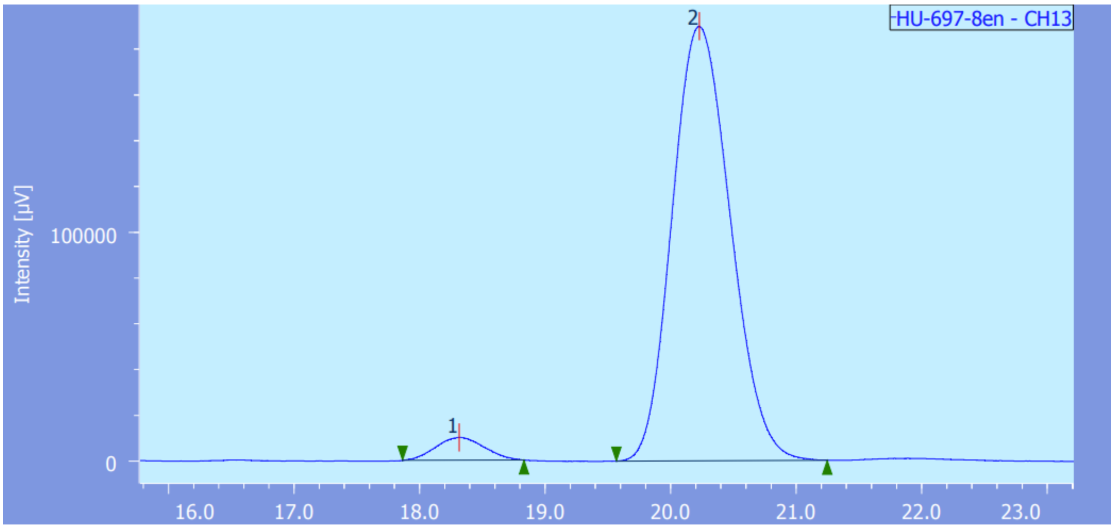

Decision

#	Peak Name	CH	tR [min]	Area [µV·sec]	Height [µV]	Area%	Height%	Quantity	NTP	Resolution	Symmetry Factor	Warning
1	Unknown	13	18.313	268330	9944	4.199	4.981	N/A	9894	2.394	1.068	
2	Unknown	13	20.227	6121265	189683	95.801	95.019	N/A	8729	N/A	1.146	

***tert*-Butyl (*R*)-3-cyclohexyl-2-(4-(methylsulfonyl)phenyl)propanoate (3)**

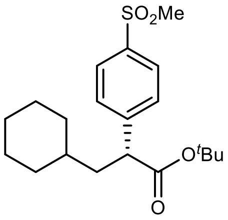

Prepared by **GP-B**. White solid, 62.2 mg, 85% yield. m.p. = 82-83 °C; ^{1}H NMR (400 MHz, $CDCl_3$) δ 7.89 – 7.85 (m, 2H), 7.53 – 7.47 (m, 2H), 3.67 (t, *J* = 7.8 Hz, 1H), 3.05 (s, 3H), 1.94 (ddd, *J* = 13.8, 8.2, 7.2 Hz, 1H), 1.74 – 1.55 (m, 6H), 1.39 (s, 9H), 1.19 – 1.07 (m, 4H), 0.97 – 0.83 (m, 2H); ^{13}C NMR (101 MHz, CH_3CN) δ 172.4, 146.4, 139.0, 128.9, 127.5, 81.2, 49.8, 44.5, 41.1, 35.3, 33.1, 32.9, 27.9, 26.4, 26.05, 26.02. IR (film): ν (cm^{-1}) 2961, 2921, 2851, 1722, 1596, 1448, 1365, 1306, 1220, 1141, 1090, 961, 835, 788, 776, 758, 742, 724, 572, 550, 540, 529; HR-MS (ESI) m/z calcd for $C_{20}H_{30}NaO_4S^+$ [M+Na$^+$] 389.17570, found

389.17564; $[\alpha]_D^{23.2}$ = -14.5 (c = 0.1, $CHCl_3$); HPLC conditions: Chiral-NR column, hexane/2-propanol = 90/10, flow rate = 1.0 mL/min, λ = 226 nm, t_R = 50.0 min (minor), t_R = 52.9 min (major), 97:3 er.

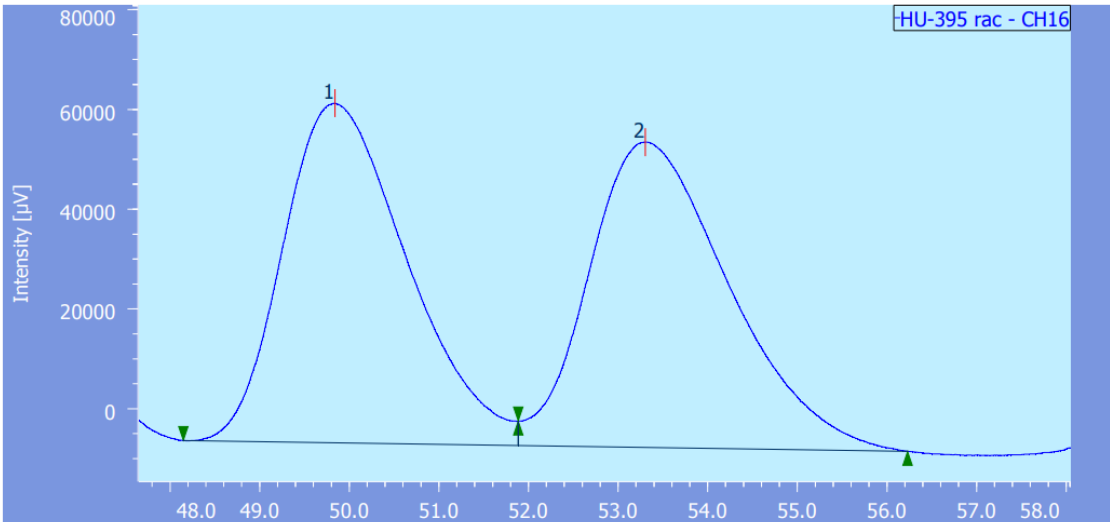

Decision

#	Peak Name	CH	tR [min]	Area [µV·sec]	Height [µV]	Area%	Height%	Quantity	NTP	Resolution	Symmetry Factor	Warning
1	Unknown	16	49.840	6536933	68025	49.605	52.650	N/A	6108	1.284	N/A	
2	Unknown	16	53.300	6640997	61177	50.395	47.350	N/A	5579	N/A	N/A	

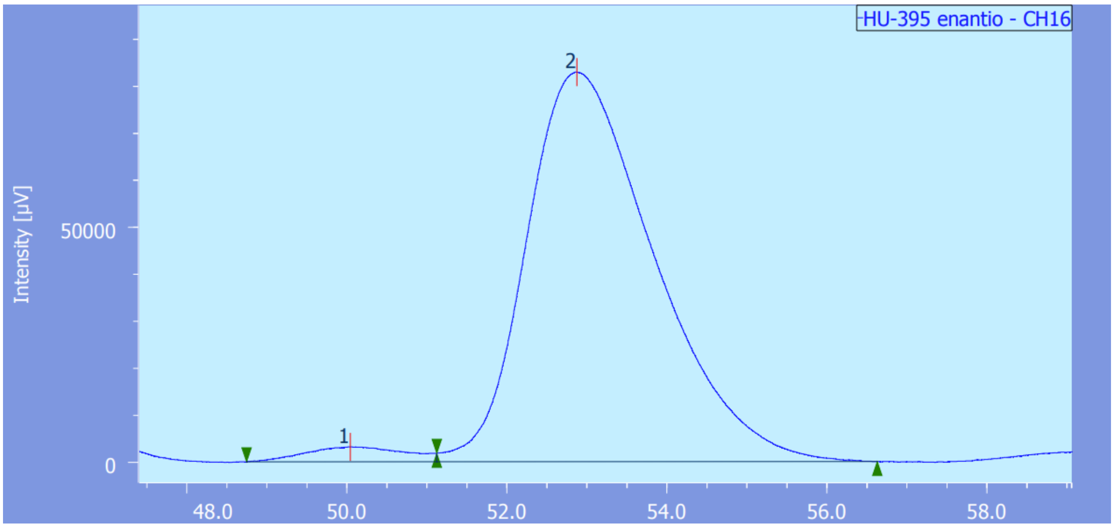

Decision

#	Peak Name	CH	tR [min]	Area [µV·sec]	Height [µV]	Area%	Height%	Quantity	NTP	Resolution	Symmetry Factor	Warning
1	Unknown	16	50.040	276894	3152	2.926	3.668	N/A	N/A	N/A	N/A	
2	Unknown	16	52.873	9185779	82785	97.074	96.332	N/A	5345	N/A	1.394	

***tert*-Butyl (*R*)-3-cyclohexyl-2-(1-oxo-1,3-dihydroisobenzofuran-5-yl)propanoate (4)**

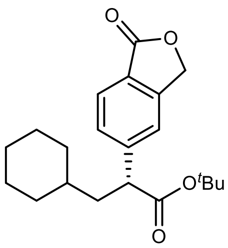

Prepared by **GP-A**. White solid, 46.4 mg, 67% yield. m.p. = 93-94 °C; ^{1}H NMR (400 MHz, $CDCl_3$) δ 7.87 – 7.81 (m, 1H), 7.45 (d, *J* = 7.3 Hz, 2H), 5.29 (s, 2H), 3.70 (dd, *J* = 8.0, 7.5 Hz, 1H), 1.96 (ddd, *J* = 13.7, 8.3, 7.1 Hz, 1H), 1.75 – 1.56 (m, 6H), 1.39 (s, 9H), 1.20 – 1.08 (m, 4H), 0.99 – 0.83 (m, 2H); ^{13}C NMR (101 MHz, $CDCl_3$) δ 172.6, 170.8, 147.02, 147.01, 129.2, 125.7, 124.5, 121.3, 81.2, 69.5, 50.2, 41.3, 35.4, 33.1, 33.0, 27.9, 26.4, 26.1, 26.0. IR (film): ν (cm^{-1}) 2978, 2960, 2928, 2840, 1756, 1720, 1619, 1448, 1393, 1366, 1347, 1244, 1213, 1147, 1137, 1125, 1049, 1001, 883, 854, 841, 786, 772, 762, 702,

689, 426; HR-MS (ESI) m/z calcd for $C_{21}H_{28}NaO_4^+$ [M+Na$^+$] 367.18798, found 367.18786; $[\alpha]_D^{23.4}$ = -26.7 (c = 0.1, $CHCl_3$); HPLC conditions: IC column, hexane/2-propanol = 90/10, flow rate = 0.5 mL/min, λ = 236 nm, t_R = 67.4 min (minor), t_R = 69.6 min (major), 97:3 er.

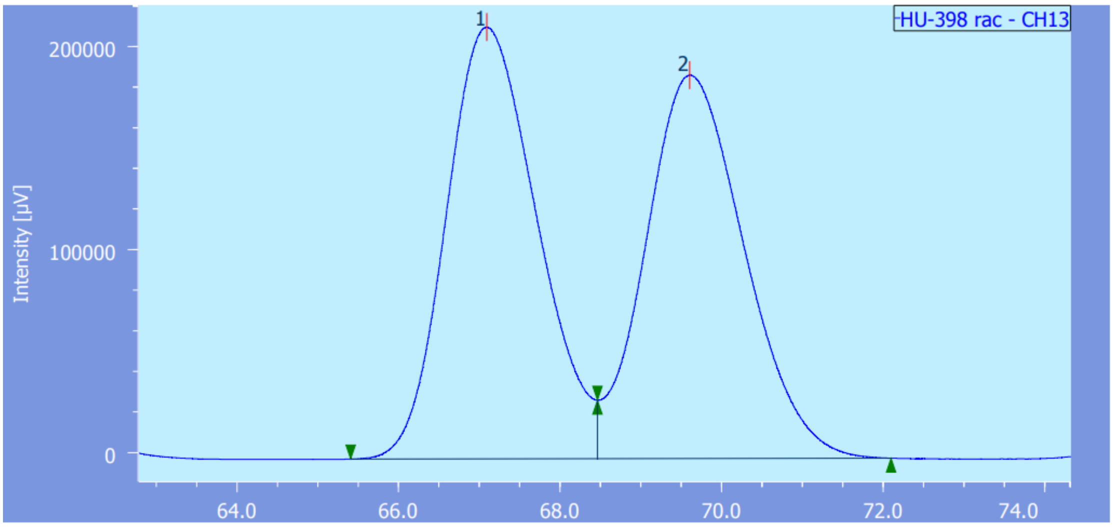

Decision

#	Peak Name	CH	tR [min]	Area [µV·sec]	Height [µV]	Area%	Height%	Quantity	NTP	Resolution	Symmetry Factor	Warning
1	Unknown	13	67.090	16607242	212546	50.770	52.962	N/A	16475	1.161	N/A	
2	Unknown	13	69.603	16103569	188772	49.230	47.038	N/A	15283	N/A	N/A	

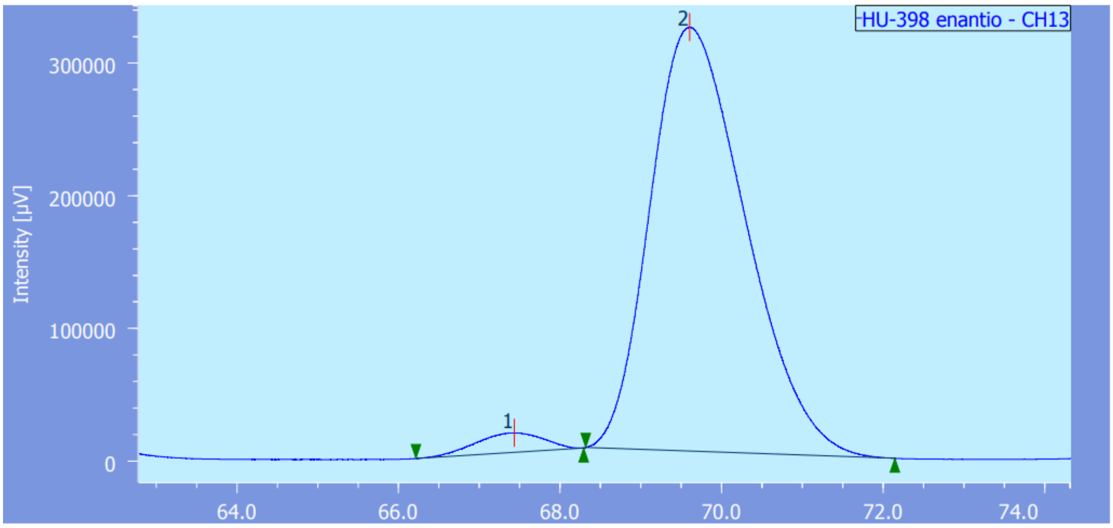

Decision

#	Peak Name	CH	tR [min]	Area [µV·sec]	Height [µV]	Area%	Height%	Quantity	NTP	Resolution	Symmetry Factor	Warning
1	Unknown	13	67.433	860299	14529	3.168	4.351	N/A	26549	1.128	0.864	
2	Unknown	13	69.603	26294941	319386	96.832	95.649	N/A	15976	N/A	1.310	

***tert*-Butyl (*R*)-2-(4-acetylphenyl)-3-cyclohexylpropanoate (5)**

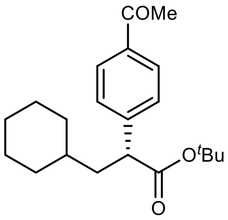

Prepared by **GP-A**. White solid, 46.5 mg, 70% yield. m.p. = 85-86 °C; ^{1}H NMR (400 MHz, $CDCl_3$) δ 7.93 – 7.85 (m, 2H), 7.42 – 7.35 (m, 2H), 3.63 (t, J = 7.8 Hz, 1H), 2.58 (s, 3H), 1.93 (ddd, J = 13.8, 8.1, 7.2 Hz, 1H), 1.78 – 1.55 (m, 6H), 1.38 (s, 9H), 1.20 – 1.07 (m, 4H), 0.98 – 0.83 (m, 2H); ^{13}C NMR (101 MHz, $CDCl_3$) δ 197.8, 172.8, 145.6, 135.8, 128.5, 128.1, 80.8, 50.0, 40.9, 35.4, 33.2, 33.0, 27.9, 26.6, 26.4, 26.11, 26.07. IR (film): ν (cm^{-1}) 2980, 2920, 2851, 1718, 1681, 1604, 1445, 1421, 1364, 1358, 1310, 1271, 1246, 1221, 1146, 1019, 960, 876, 834, 777, 751, 698, 599, 593; HR-MS (ESI) m/z calcd for $C_{21}H_{30}NaO_3^+$ [M+Na$^+$]

353.20872, found 353.20914; $[\alpha]_D^{23.4}$ = -40.4 (c = 0.1, $CHCl_3$); HPLC conditions: Chiral-NR column, hexane/2-propanol = 98/2, flow rate = 1.0 mL/min, λ = 250 nm, t_R = 25.4 min (minor), t_R = 28.3 min (major), 95:5 er.

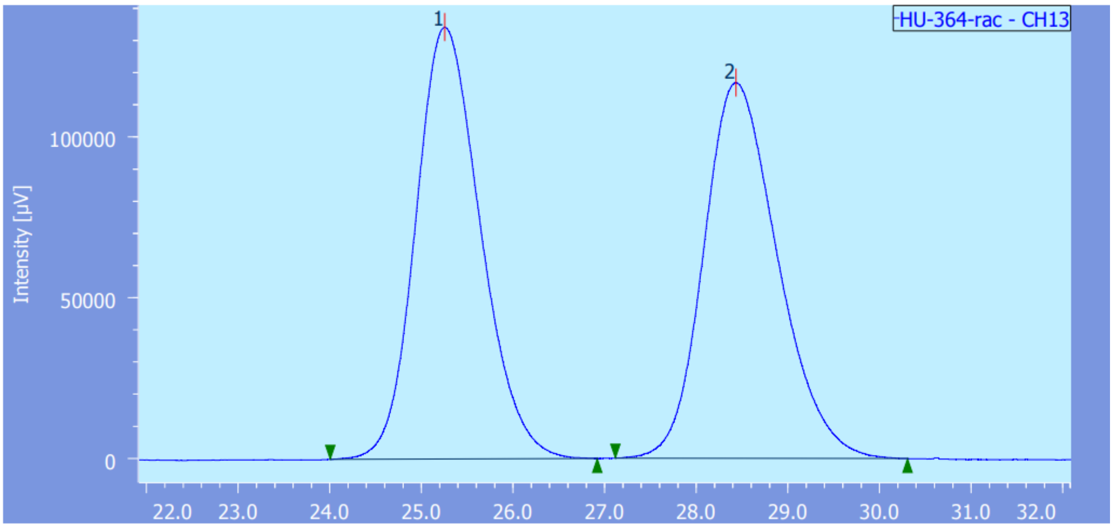

Decision

#	Peak Name	CH	tR [min]	Area [µV·sec]	Height [µV]	Area%	Height%	Quantity	NTP	Resolution	Symmetry Factor	Warning
1	Unknown	13	25.253	6599423	134252	50.112	53.468	N/A	6181	2.318	1.174	
2	Unknown	13	28.433	6569831	116835	49.888	46.532	N/A	6020	N/A	1.220	

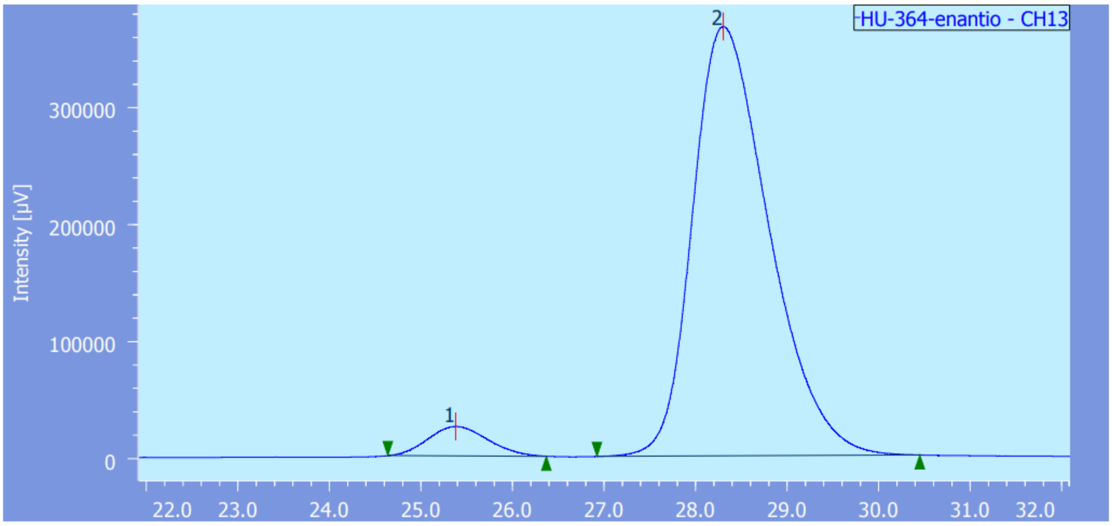

Decision

#	Peak Name	CH	tR [min]	Area [µV·sec]	Height [µV]	Area%	Height%	Quantity	NTP	Resolution	Symmetry Factor	Warning
1	Unknown	13	25.377	1148444	25155	5.103	6.416	N/A	6836	2.136	1.138	
2	Unknown	13	28.300	21357429	366886	94.897	93.584	N/A	5573	N/A	1.372	

Methyl (*R*)-4-(1-(*tert*-butoxy)-3-cyclohexyl-1-oxopropan-2-yl)benzoate (6)

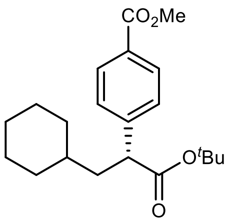

Prepared by **GP-A** by using $NiBr_2 \cdot 3H_2O$ instead of $NiBr_2 \cdot DME$. White solid, 47.6 mg, 72% yield. m.p. = 96-97 °C; ^1H NMR (400 MHz, $CDCl_3$) δ 8.00 – 7.94 (m, 2H), 7.40 – 7.33 (m, 2H), 3.89 (s, 3H), 3.62 (t, *J* = 7.8 Hz, 1H), 1.93 (ddd, *J* = 13.8, 8.2, 7.2 Hz, 1H), 1.78 – 1.55 (m, 6H), 1.37 (s, 9H), 1.19 – 1.05 (m, 4H), 0.97 – 0.82 (m, 2H); ^{13}C NMR (101 MHz, $CDCl_3$) δ 172.8, 166.9, 145.3, 129.7, 128.7, 127.9, 80.8, 52.0, 50.0, 40.9, 35.4, 33.2, 33.0, 27.9, 26.4, 26.10, 26.07. IR (film): ν (cm^{-1}) 2978, 2928, 2902, 2850, 2842, 1718, 1608, 1448, 1436, 1416, 1366, 1347, 1309, 1277, 1260, 1235, 1225, 1177, 1145, 1127, 1105, 1090, 1015, 961, 864,

840, 755, 707, 484; HR-MS (ESI) m/z calcd for $C_{21}H_{30}NaO_4^+$ [M+Na$^+$] 369.20363, found 369.20381; $[\alpha]_D^{23.5}$ = -31.1 (c = 0.1, $CHCl_3$); HPLC conditions: Chiral-NR column, hexane/2-propanol = 98/2, flow rate = 1.0 mL/min, λ = 240 nm, t_R = 16.5 min (minor), t_R = 17.6 min (major), 96:4 er.

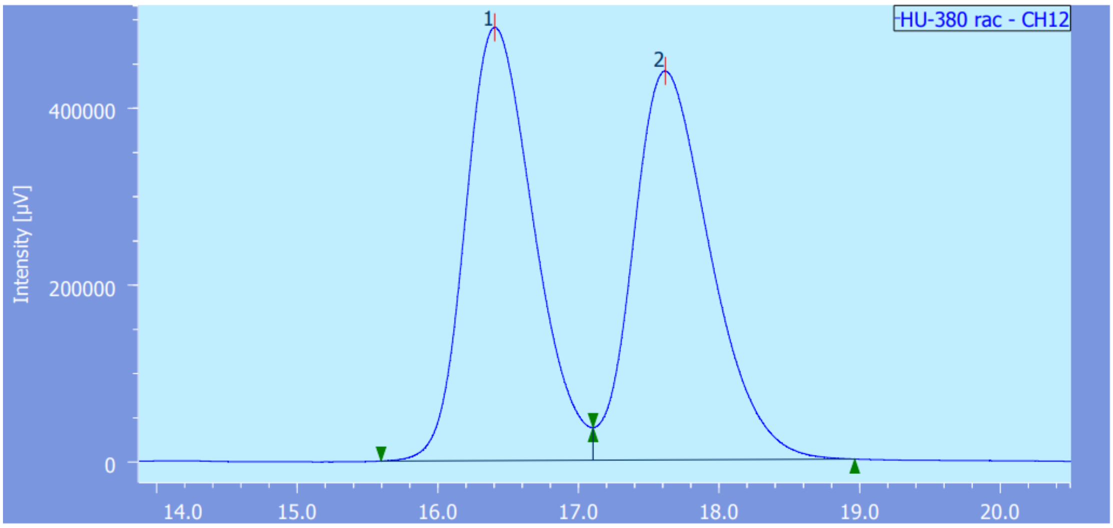

Decision

#	Peak Name	CH	tR [min]	Area [µV·sec]	Height [µV]	Area%	Height%	Quantity	NTP	Resolution	Symmetry Factor	Warning
1	Unknown	12	16.403	16608615	489777	49.740	52.702	N/A	5391	1.288	N/A	
2	Unknown	12	17.617	16782467	439549	50.260	47.298	N/A	5014	N/A	N/A	

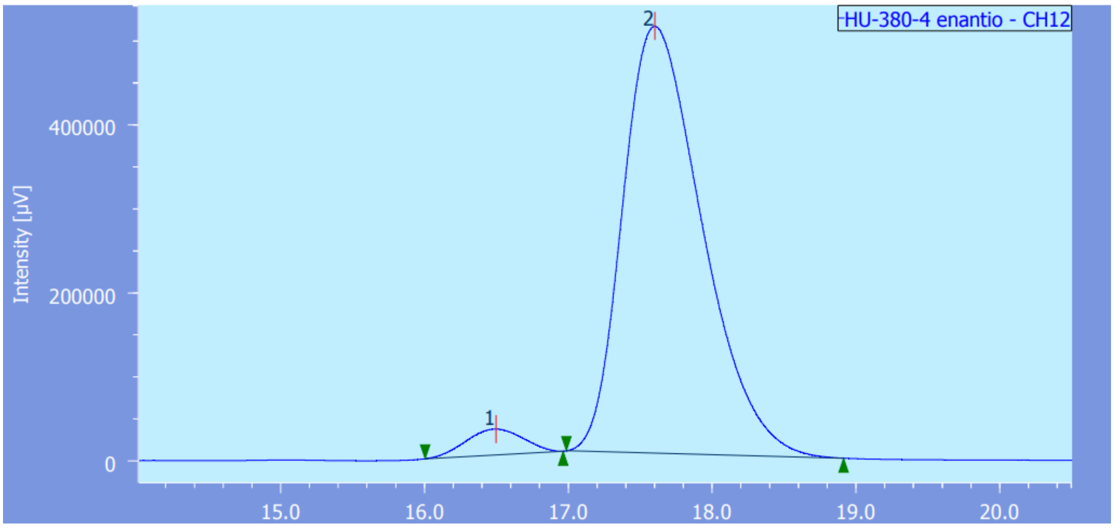

Decision

#	Peak Name	CH	tR [min]	Area [µV·sec]	Height [µV]	Area%	Height%	Quantity	NTP	Resolution	Symmetry Factor	Warning
1	Unknown	12	16.497	854988	30744	4.187	5.704	N/A	7314	1.237	0.960	
2	Unknown	12	17.600	19566865	508202	95.813	94.296	N/A	4790	N/A	1.393	

***tert*-Butyl (*R*)-3-cyclohexyl-2-(4-(trifluoromethyl)phenyl)propanoate (7)**

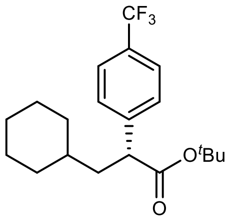

Prepared by **GP-A**. White solid, 53.2 mg, 75% yield. m.p. = 64-65 °C; ^{1}H NMR (400 MHz, $CDCl_3$) δ 7.56 (d, *J* = 8.1 Hz, 2H), 7.42 (d, *J* = 8.2 Hz, 2H), 3.64 (t, *J* = 7.8 Hz, 1H), 1.95 (ddd, *J* = 13.8, 8.3, 7.1 Hz, 1H), 1.76 – 1.54 (m, 6H), 1.39 (s, 9H), 1.22 – 1.08 (m, 4H), 0.98 – 0.84 (m, 2H); ^{13}C NMR (101 MHz, $CDCl_3$) δ 172.8, 144.1, 129.1 (q, *J* = 32.4 Hz) 128.2, 125.4 (q, *J* = 4.0 Hz), 124.2 (q, *J* = 272.7 Hz), 80.9, 49.8, 41.1, 35.4, 33.2, 33.0, 27.9, 26.4, 26.12, 26.08; ^{19}F NMR (377 MHz, $CDCl_3$) δ -62.45. IR (film): ν (cm^{-1}) 2978, 2926, 2903,

2855, 1718, 1616, 1477, 1449, 1423, 1368, 1325, 1222, 1147, 1122, 1109, 1068, 1020, 893, 839, 760, 601, 517; HR-MS (ESI) m/z calcd for $C_{20}H_{27}F_3NaO_2^+$ [M+Na$^+$] 379.18554, found 379.18572; $[\alpha]_D^{23.5}$ = -28.0 (c = 0.1, $CHCl_3$); HPLC conditions: Chiral-NR column, hexane/2-propanol = 99.8/0.2, flow rate = 0.5 mL/min, λ = 210 nm, t_R = 14.8 min (minor), t_R = 21.4 min (major), 95:5 er.

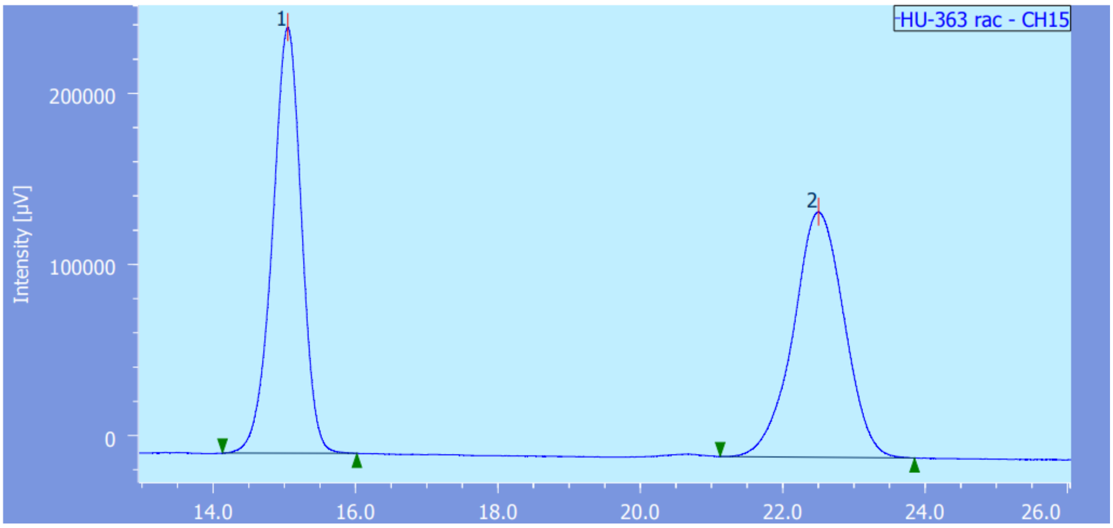

Decision

#	Peak Name	CH	tR [min]	Area [µV·sec]	Height [µV]	Area%	Height%	Quantity	NTP	Resolution	Symmetry Factor	Warning
1	Unknown	15	15.047	7060406	248832	49.740	63.436	N/A	6728	7.392	0.930	
2	Unknown	15	22.500	7134226	143423	50.260	36.564	N/A	4881	N/A	0.971	

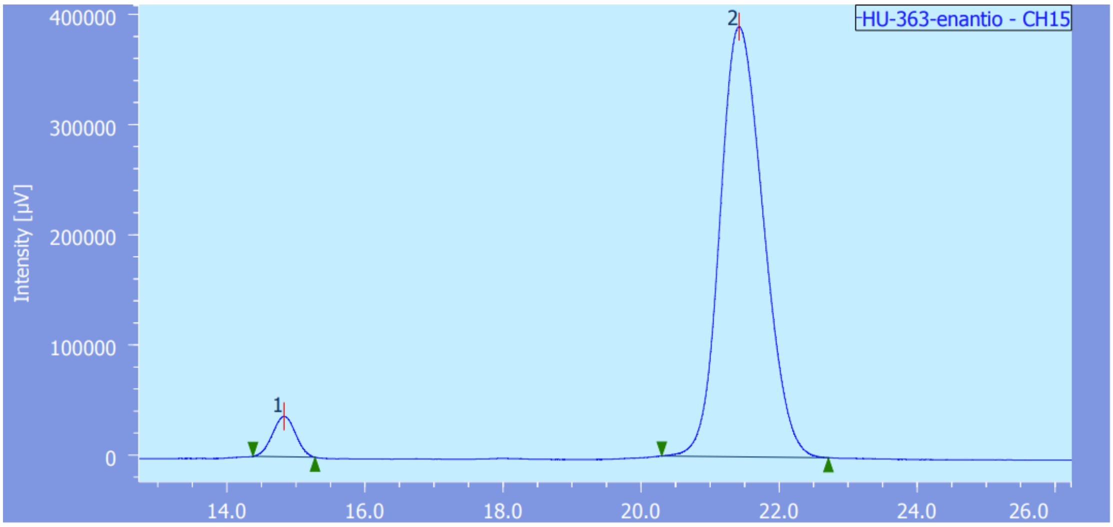

Decision

#	Peak Name	CH	tR [min]	Area [µV·sec]	Height [µV]	Area%	Height%	Quantity	NTP	Resolution	Symmetry Factor	Warning
1	Unknown	15	14.827	860252	36712	4.807	8.602	N/A	8828	7.384	1.004	
2	Unknown	15	21.417	17035268	390082	95.193	91.398	N/A	5469	N/A	1.164	

tert-Butyl (R)-3-cyclohexyl-2-(4-fluorophenyl)propanoate (8)

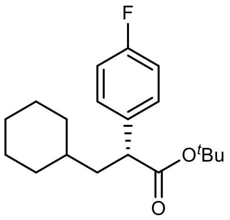

Prepared by **GP-A**. White solid, 43.2 mg, 70% yield. m.p. = 68-69 °C; ^{1}H NMR (400 MHz, $CDCl_3$) δ 7.22 – 7.15 (m, 2H), 6.95 – 6.87 (m, 2H), 3.48 (t, J = 8.0 Hz, 1H), 1.83 (ddd, J = 13.7, 8.3, 7.0 Hz, 1H), 1.69 – 1.45 (m, 6H), 1.31 (s, 9H), 1.13 – 1.01 (m, 4H), 0.90 – 0.77 (m, 2H); ^{13}C NMR (101 MHz, $CDCl_3$) δ 173.5, 163.0, 160.6, 135.8 (d, J = 3.2 Hz), 129.3 (d, J = 8.0 Hz), 115.2 (d, J = 21.3 Hz), 80.5, 49.1, 41.2, 35.4, 33.2, 33.0, 27.9, 26.5, 26.2, 26.1; ^{19}F NMR (377 MHz, $CDCl_3$) δ -116.24. IR (film): ν (cm^{-1}) 2981, 2920, 2850, 1718, 1603,

1509, 1447, 1366, 1358, 1218, 1143, 1097, 1015, 973, 904, 877, 834, 807, 763, 733, 704, 581, 524, 490, 421; HR-MS (ESI) m/z calcd for $C_{19}H_{27}FNaO_2^+$ [M+Na$^+$] 329.18873, found 329.18877; $[\alpha]_D^{23.6}$ = -18.9 (c = 0.1, $CHCl_3$); HPLC conditions: Chiral-NR column, hexane/2-propanol = 99.8/0.2, flow rate = 0.5 mL/min, λ = 207 nm, t_R = 20.5 min (minor), t_R = 24.8 min (major), 94:6 er.

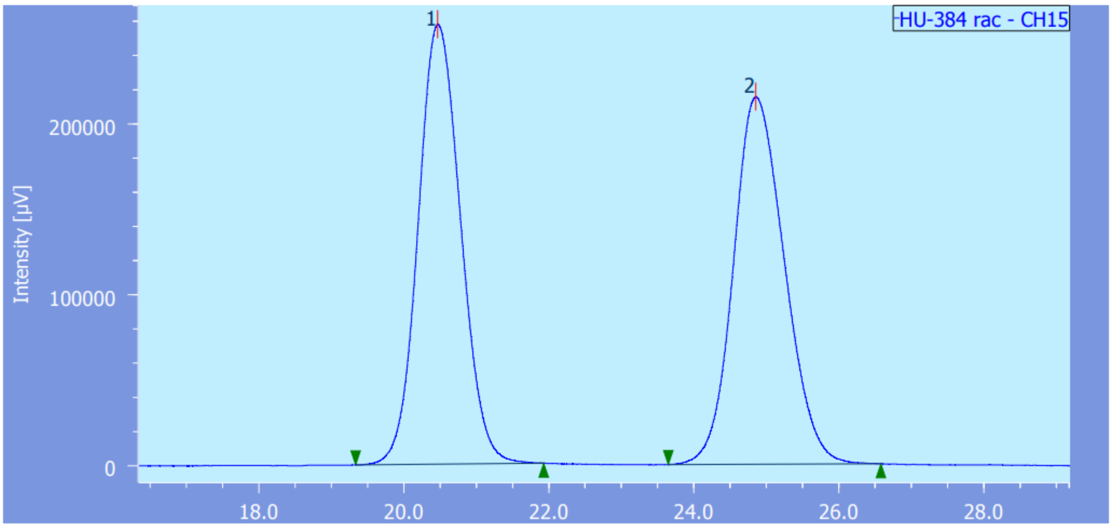

Decision

#	Peak Name	CH	tR [min]	Area [µV·sec]	Height [µV]	Area%	Height%	Quantity	NTP	Resolution	Symmetry Factor	Warning
1	Unknown	15	20.463	10475434	257282	49.873	54.484	N/A	5828	3.729	1.100	
2	Unknown	15	24.850	10528914	214934	50.127	45.516	N/A	5966	N/A	1.152	

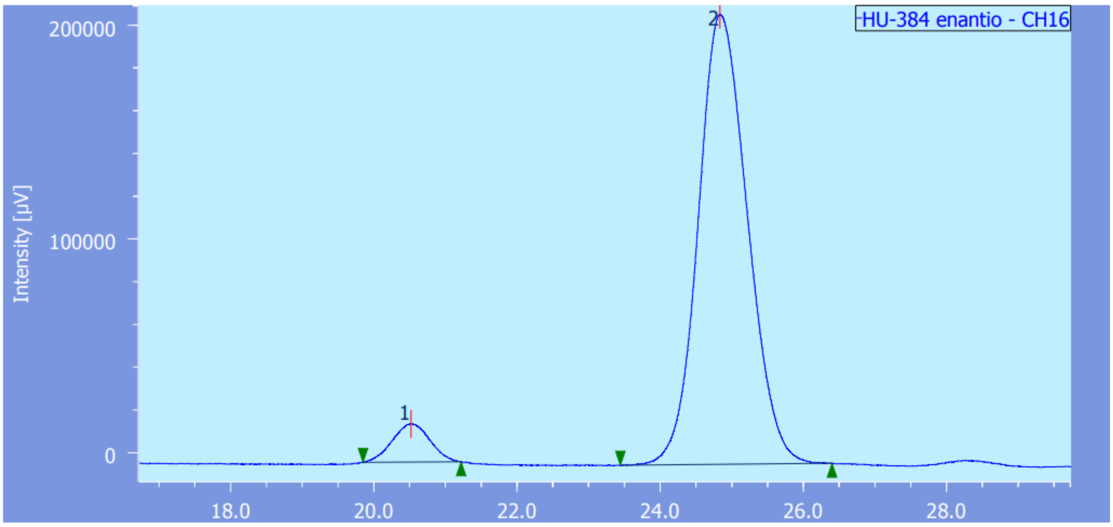

Decision

#	Peak Name	CH	tR [min]	Area [µV·sec]	Height [µV]	Area%	Height%	Quantity	NTP	Resolution	Symmetry Factor	Warning
1	Unknown	16	20.520	658284	17916	6.143	7.848	N/A	6847	3.845	0.999	
2	Unknown	16	24.833	10057527	210365	93.857	92.152	N/A	6239	N/A	1.125	

tert-Butyl (_R_)-2-(4-chlorophenyl)-3-cyclohexylpropanoate (9)

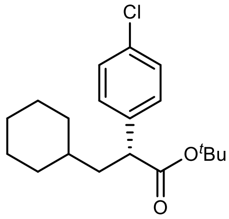

Prepared by **GP-A**. White solid, 46.8 mg, 72% yield. m.p. = 76-77 °C; ^{1}H NMR (400 MHz, $CDCl_3$) δ 7.22 – 7.12 (m, 4H), 3.47 (t, J = 7.8 Hz, 1H), 1.82 (ddd, J = 13.7, 8.2, 7.2 Hz, 1H), 1.68 – 1.44 (m, 6H), 1.31 (s, 9H), 1.14 – 1.00 (m, 4H), 0.89 – 0.76 (m, 2H); ^{13}C NMR (101 MHz, $CDCl_3$) δ 173.2, 138.5, 132.6, 129.2, 128.5, 80.6, 49.3, 41.0, 35.3, 33.2, 33.0, 27.9, 26.4, 26.13, 26.09. IR (film): ν (cm^{-1}) 2981, 2919, 2850, 1718, 1493, 1444, 1418, 1390, 1366, 1356, 1330, 1296, 1274, 1255, 1219, 1144, 1090, 1013, 972, 902, 892, 876, 841, 828,

768, 722, 564, 516, 503; HR-MS (ESI) m/z calcd for $C_{19}H_{27}ClNaO_2^+$ [M+Na$^+$] 345.15918, found 345.15928; $[\alpha]_D^{23.6}$ = -23.4 (c = 0.1, $CHCl_3$); HPLC conditions: Chiral-NR column, hexane/2-propanol = 99.8/0.2, flow rate = 0.5 mL/min, λ = 220 nm, t_R = 25.1 min (minor), t_R = 31.5 min (major), 95:5 er.

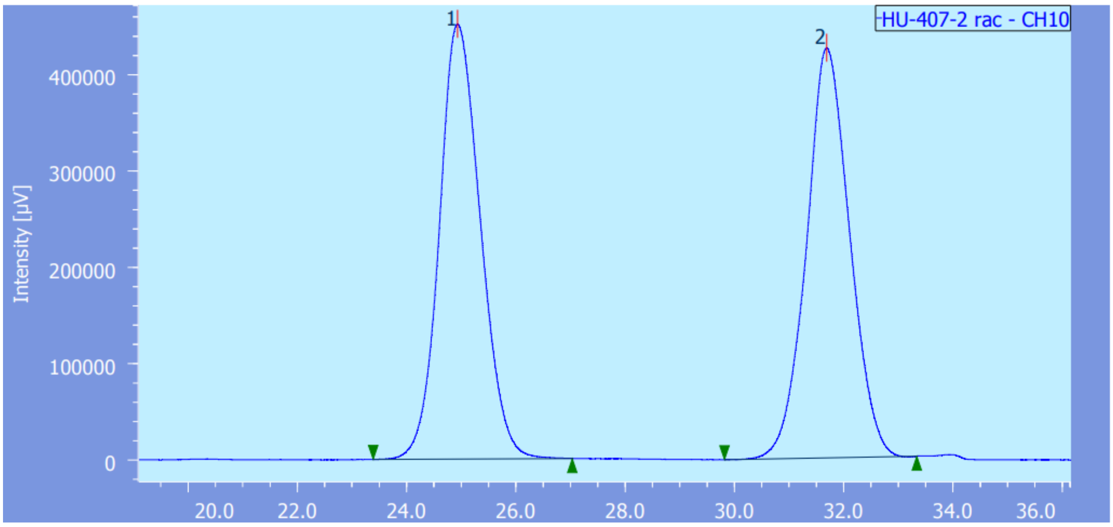

Decision

#	Peak Name	CH	tR [min]	Area [µV·sec]	Height [µV]	Area%	Height%	Quantity	NTP	Resolution	Symmetry Factor	Warning
1	Unknown	10	24.930	23583307	451503	50.151	51.490	N/A	5395	4.877	1.127	
2	Unknown	10	31.687	23441516	425374	49.849	48.510	N/A	7960	N/A	1.046	

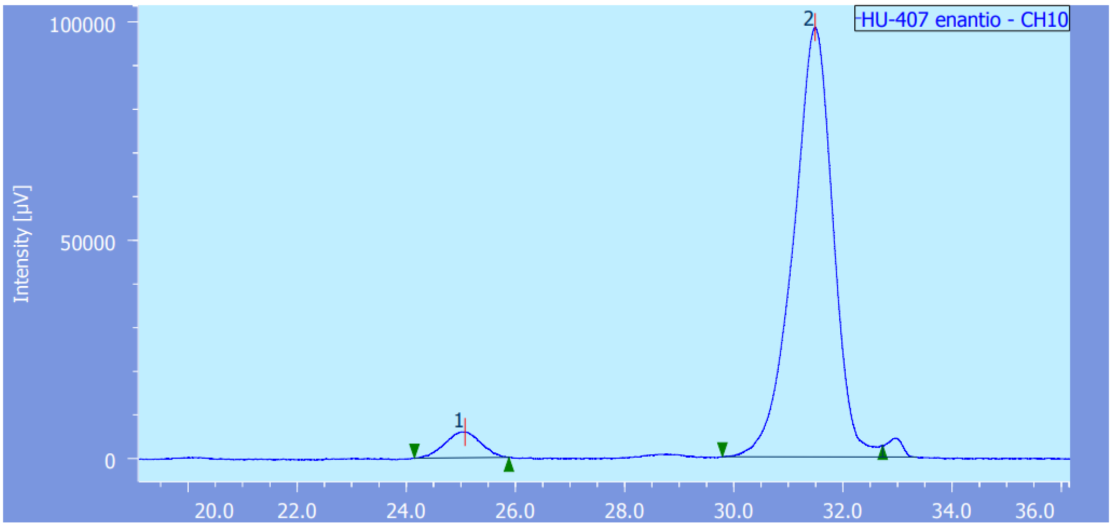

Decision

#	Peak Name	CH	tR [min]	Area [µV·sec]	Height [µV]	Area%	Height%	Quantity	NTP	Resolution	Symmetry Factor	Warning
1	Unknown	10	25.073	271664	5907	5.171	5.668	N/A	6657	5.122	0.924	
2	Unknown	10	31.487	4981907	98309	94.829	94.332	N/A	9653	N/A	0.879	

***tert*-Butyl (*R*)-3-cyclohexyl-2-phenylpropanoate (10)**

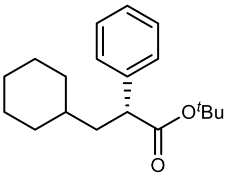

Prepared by **GP-B**. Colorless oil, 39.5 mg, 68% yield. ^{1}H NMR (400 MHz, $CDCl_3$) δ 7.25 – 7.20 (m, 4H), 7.19 – 7.13 (m, 1H), 3.50 (dd, *J* = 8.4, 7.2 Hz, 1H), 1.86 (ddd, *J* = 13.8, 8.5, 7.0 Hz, 1H), 1.70 – 1.47 (m, 6H), 1.31 (s, 9H), 1.15 – 1.02 (m, 4H), 0.91 – 0.76 (m, 2H); ^{13}C NMR (101 MHz, $CDCl_3$) δ 173.6, 140.1, 128.4, 127.8, 126.8, 80.4, 49.9, 41.2, 35.4, 33.2, 33.1, 27.9, 26.5, 26.2, 26.1. IR (film): ν (cm^{-1}) 2976, 2923, 2849, 1726, 1602, 1495, 1478, 1448, 1391, 1365, 1274, 1252, 1219, 1143, 1032, 970, 891, 842, 750, 729, 697, 547, 519; HR-MS (ESI) m/z calcd for

$C_{19}H_{28}NaO_2^+$ [M+Na$^+$] 311.19815, found 311.19827; $[\alpha]_D^{22.7}$ = -28.1 (c = 0.1, $CHCl_3$); HPLC conditions: Chiral-NR column, hexane/2-propanol = 99.8/0.2, flow rate = 0.5 mL/min, λ = 210 nm, t_R = 23.0 min (minor), t_R = 24.6 min (major), 96:4 er.

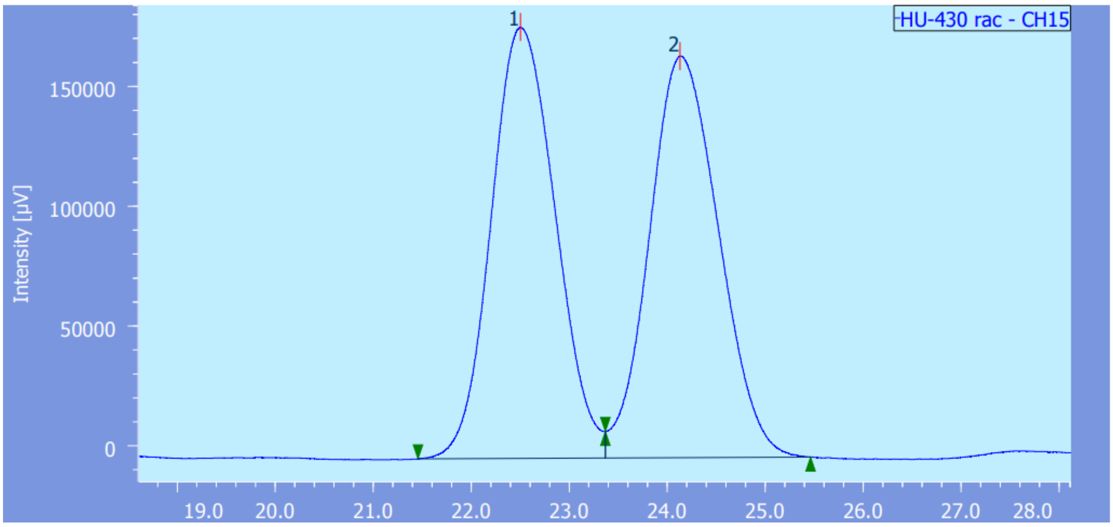

Decision

#	Peak Name	CH	tR [min]	Area [μV·sec]	Height [μV]	Area%	Height%	Quantity	NTP	Resolution	Symmetry Factor	Warning
1	Unknown	15	22.500	8203439	179910	49.868	51.784	N/A	5426	1.288	N/A	
2	Unknown	15	24.130	8246842	167517	50.132	48.216	N/A	5386	N/A	N/A	

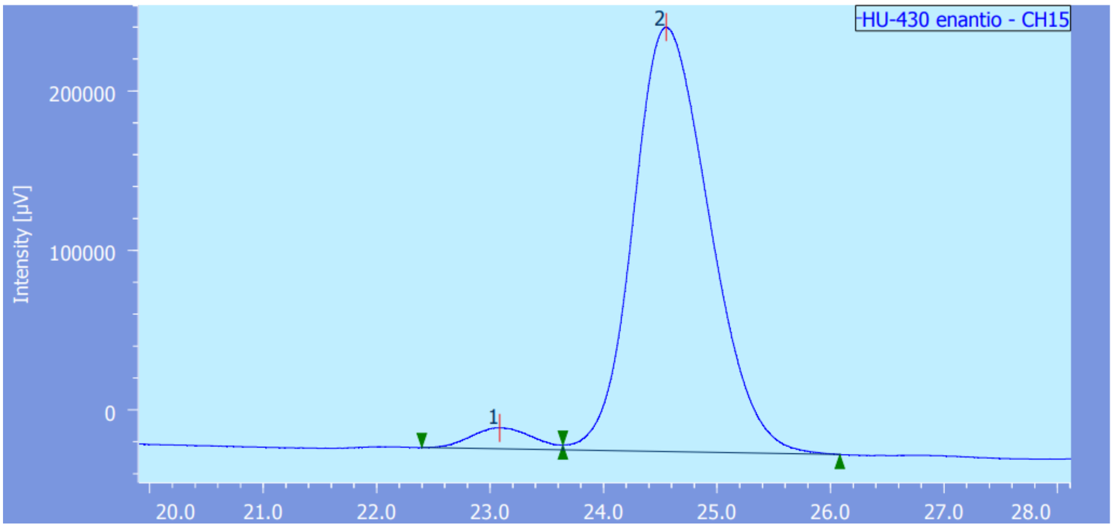

Decision

#	Peak Name	CH	tR [min]	Area [μV·sec]	Height [μV]	Area%	Height%	Quantity	NTP	Resolution	Symmetry Factor	Warning
1	Unknown	15	23.083	503753	13196	3.889	4.727	N/A	7834	1.293	N/A	
2	Unknown	15	24.550	12448801	265991	96.111	95.273	N/A	6365	N/A	1.190	

***tert*-Butyl (*R*)-2-([1,1'-biphenyl]-4-yl)-3-cyclohexylpropanoate (11)**

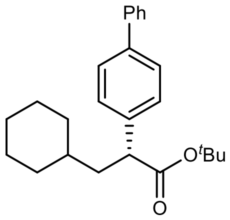

Prepared by **GP-B**. White solid, 51.0 mg, 70% yield. m.p. = 64-65 ºC; ^{1}H NMR (400 MHz, $CDCl_3$) δ 7.63 – 7.58 (m, 2H), 7.58 – 7.52 (m, 2H), 7.47 – 7.41 (m, 2H), 7.41 – 7.36 (m, 2H), 7.36 – 7.31 (m, 1H), 3.64 (dd, *J* = 8.5, 7.1 Hz, 1H), 1.99 (ddd, *J* = 13.7, 8.6, 6.9 Hz, 1H), 1.83 – 1.59 (m, 6H), 1.43 (s, 9H), 1.29 – 1.13 (m, 4H), 1.02 – 0.88 (m, 2H); ^{13}C NMR (101 MHz, $CDCl_3$) δ 173.6, 140.8, 139.6, 139.2, 128.7, 128.2, 127.12, 127.08, 127.0, 80.5, 49.5, 41.3, 35.5, 33.19, 33.16, 28.0, 26.5, 26.17, 26.15. IR (film): ν (cm^{-1}) 2975, 2931, 2917, 2847, 1720, 1484, 1447, 1393, 1367, 1337, 1319, 1259, 1218, 1142, 1126, 1008, 967, 890, 879, 825,

757, 730, 697, 681, 557, 549, 502; HR-MS (ESI) m/z calcd for $C_{25}H_{32}NaO_2^+$ [M+Na$^+$] 387.22945, found 387.22919; $[\alpha]_D^{23.8}$ = -24.6 (c = 0.1, $CHCl_3$); HPLC conditions: Chiral-NR column, hexane/2-propanol = 99/1, flow rate = 1.0 mL/min, λ = 254 nm, t_R = 11.0 min (minor), t_R = 18.1 min (major), 96:4 er.

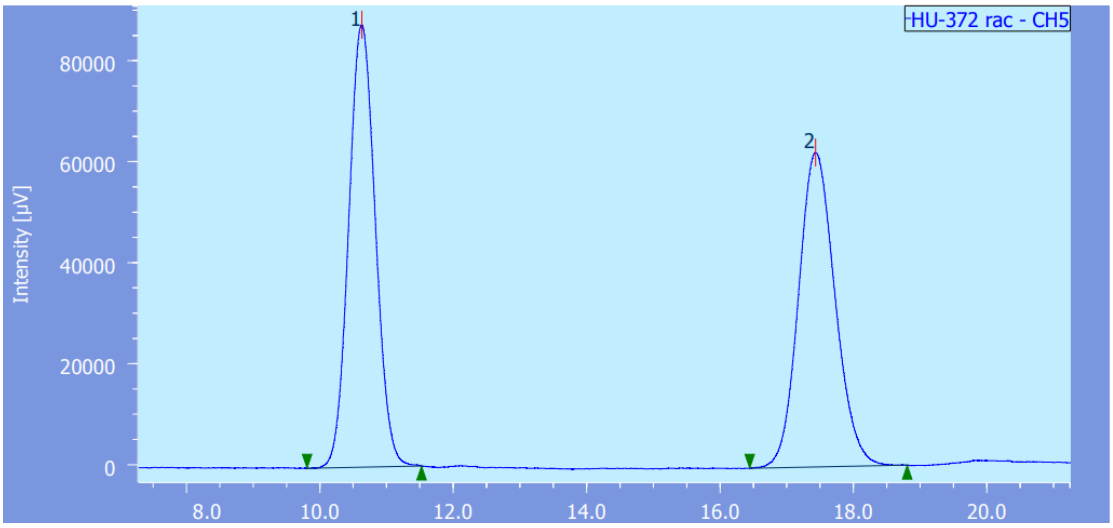

Decision

#	Peak Name	CH	tR [min]	Area [μV·sec]	Height [μV]	Area%	Height%	Quantity	NTP	Resolution	Symmetry Factor	Warning
1	Unknown	5	10.627	2428999	87561	50.576	58.440	N/A	3341	7.878	1.062	
2	Unknown	5	17.430	2373713	62270	49.424	41.560	N/A	4896	N/A	1.122	

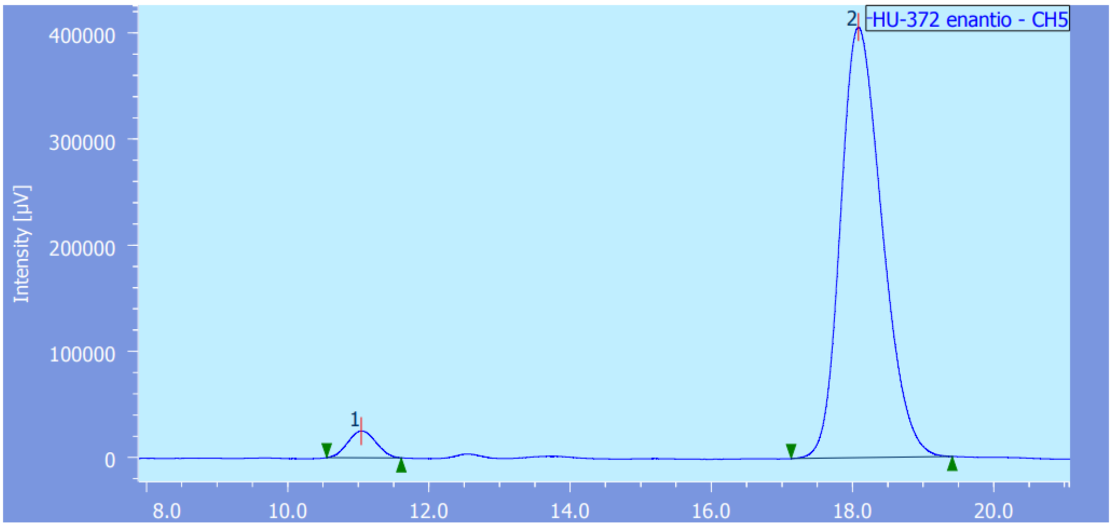

Decision

#	Peak Name	CH	tR [min]	Area [μV·sec]	Height [μV]	Area%	Height%	Quantity	NTP	Resolution	Symmetry Factor	Warning
1	Unknown	5	11.040	706312	25188	4.084	5.849	N/A	3357	7.673	1.057	
2	Unknown	5	18.080	16587493	405455	95.916	94.151	N/A	4503	N/A	1.246	

***tert*-Butyl (*R*)-3-cyclohexyl-2-(4-(trifluoromethoxy)phenyl)propanoate (12)**

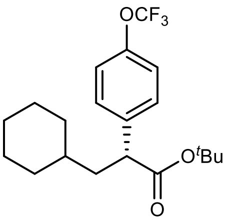

Prepared by **GP-A**. White solid, 45.1 mg, 60% yield. m.p. = 69-70 °C; ^{1}H NMR (400 MHz, $CDCl_3$) δ 7.35 – 7.29 (m, 2H), 7.14 (d, *J* = 8.1 Hz, 2H), 3.58 (dd, *J* = 8.3, 7.4 Hz, 1H), 1.92 (ddd, *J* = 13.8, 8.5, 6.9 Hz, 1H), 1.77 – 1.51 (m, 6H), 1.39 (s, 9H), 1.22 – 1.09 (m, 4H), 0.99 – 0.83 (m, 2H); ^{13}C NMR (101 MHz, $CDCl_3$) δ 173.2, 148.1, 138.8, 129.2, 120.8, 120.5 (q, *J* = 257.9 Hz), 80.8, 49.2, 41.3, 35.4, 33.2, 33.1, 27.9, 26.5, 26.14, 26.11; ^{19}F NMR (377 MHz, $CDCl_3$) δ -57.86. IR (film): ν (cm^{-1}) 2980, 2947, 2921, 2851, 1718, 1507, 1448,

1367, 1258, 1217, 1205, 1190, 1145, 1129, 1103, 920, 852, 842, 813, 765, 695, 668, 619, 539; HR-MS (ESI) m/z calcd for $C_{20}H_{27}F_3NaO_3^+$ [M+Na$^+$] 395.18045, found 395.18069; $[\alpha]_D^{23.7}$ = -19.5 (c = 0.1, $CHCl_3$); HPLC conditions: Chiral-NR column, hexane/2-propanol = 99.8/0.2, flow rate = 0.5 mL/min, λ = 210 nm, t_R = 14.0 min (minor), t_R = 19.8 min (major), 94:6 er.

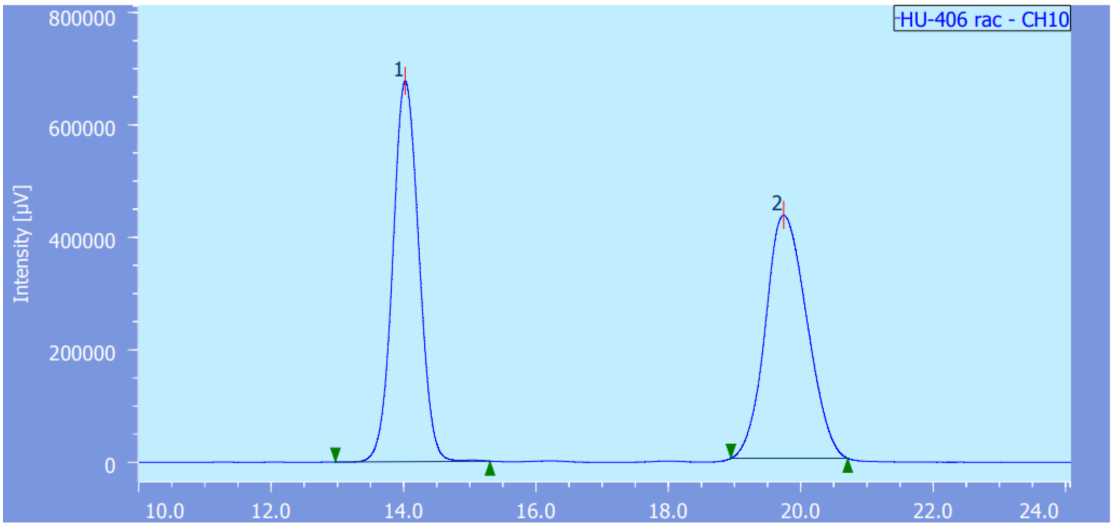

Decision

#	Peak Name	CH	tR [min]	Area [μV·sec]	Height [μV]	Area%	Height%	Quantity	NTP	Resolution	Symmetry Factor	Warning
1	Unknown	10	14.023	18576143	677190	49.610	61.034	N/A	6067	6.086	1.044	
2	Unknown	10	19.740	18868523	432336	50.390	38.966	N/A	4606	N/A	1.125	

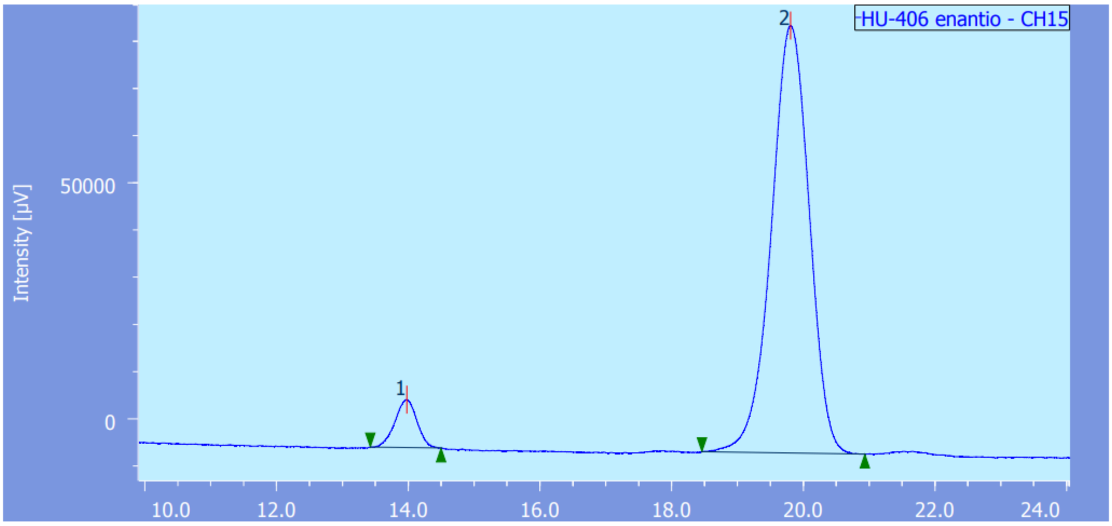

Decision

#	Peak Name	CH	tR [min]	Area [μV·sec]	Height [μV]	Area%	Height%	Quantity	NTP	Resolution	Symmetry Factor	Warning
1	Unknown	15	13.977	234629	10124	6.060	10.056	N/A	8527	7.066	0.940	
2	Unknown	15	19.803	3637130	90552	93.940	89.944	N/A	5711	N/A	0.945	

***tert*-Butyl (*R*)-2-(4-acetoxyphenyl)-3-cyclohexylpropanoate (13)**

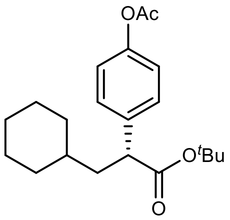

Prepared by **GP-B**. White solid, 41.9 mg, 60% yield. m.p. = 75-76 °C; ^{1}H NMR (400 MHz, $CDCl_3$) δ 7.33 – 7.27 (m, 2H), 7.06 – 6.99 (m, 2H), 3.57 (dd, *J* = 8.6, 7.0 Hz, 1H), 2.28 (s, 3H), 1.91 (ddd, *J* = 13.7, 8.7, 6.8 Hz, 1H), 1.76 – 1.53 (m, 6H), 1.39 (s, 9H), 1.22 – 1.12 (m, 4H), 0.97 – 0.83 (m, 2H); ^{13}C NMR (101 MHz, $CDCl_3$) δ 173.4, 169.4, 149.5, 137.6, 128.8, 121.4, 80.5, 49.3, 41.4, 35.4, 33.14, 33.12, 27.9, 26.5, 26.12, 26.11, 21.1. IR (film): ν (cm^{-1}) 2977, 2928, 2916, 2844, 1757, 1719, 1509, 1446, 1366, 1256, 1213, 1144, 1021, 1010, 913,

855, 842, 768, 744, 657, 593, 574, 524; HR-MS (ESI) m/z calcd for $C_{21}H_{30}NaO_4^+$ [M+Na$^+$] 369.20363, found 369.20377; $[\alpha]_D^{22.3}$ = -17.0 (c = 0.1, $CHCl_3$); HPLC conditions: NR column, hexane/2-propanol = 98/2, flow rate = 1.0 mL/min, λ = 220 nm, t_R = 21.8 min (minor), t_R = 24.2 min (major), 95:5 er.

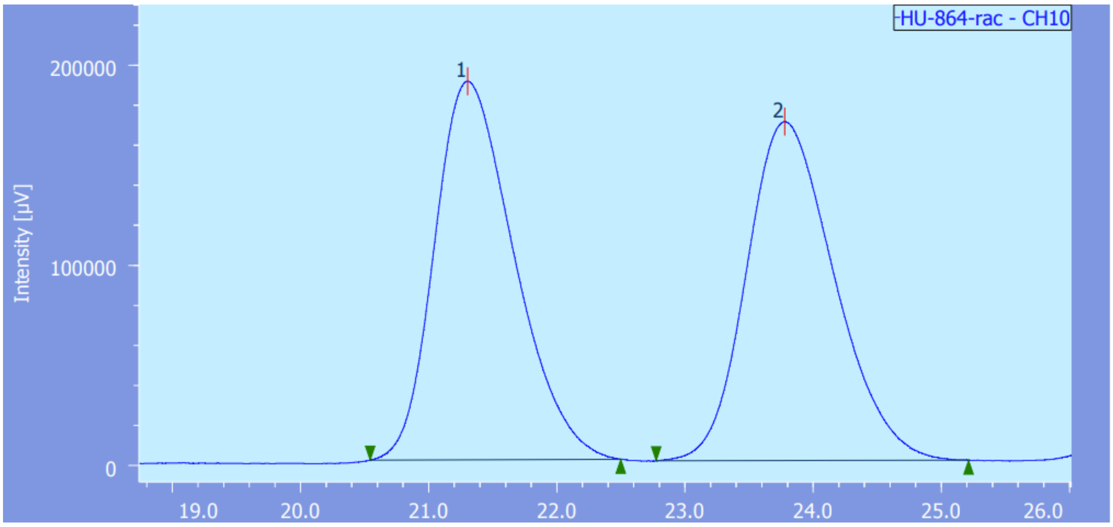

Decision

#	Peak Name	CH	tR [min]	Area [µV·sec]	Height [µV]	Area%	Height%	Quantity	NTP	Resolution	Symmetry Factor	Warning
1	Unknown	10	21.303	8168155	189065	50.272	52.768	N/A	5562	2.066	1.299	
2	Unknown	10	23.777	8079927	169228	49.728	47.232	N/A	5714	N/A	1.217	

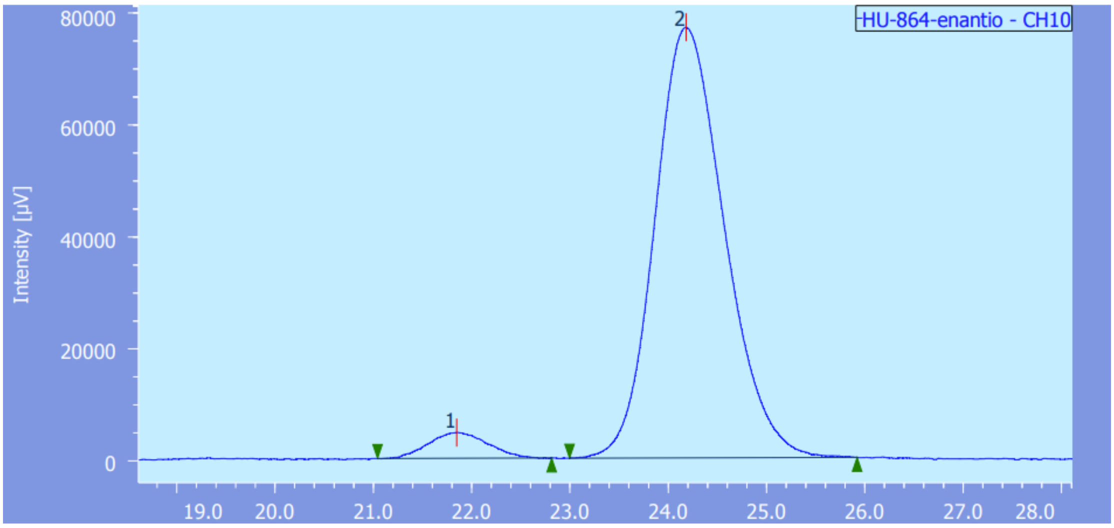

Decision

#	Peak Name	CH	tR [min]	Area [µV·sec]	Height [µV]	Area%	Height%	Quantity	NTP	Resolution	Symmetry Factor	Warning
1	Unknown	10	21.847	195321	4620	4.904	5.672	N/A	5669	1.913	1.097	
2	Unknown	10	24.180	3787459	76832	95.096	94.328	N/A	5661	N/A	1.180	

(*R*)-4-(1-(*tert*-Butoxy)-3-cyclohexyl-1-oxopropan-2-yl)phenyl benzoate (14)

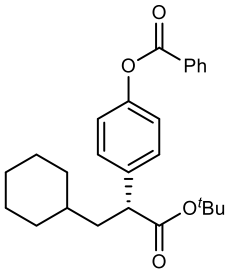

Prepared by **GP-B**. White solid, 48.0 mg, 59% yield. m.p. = 77-78 °C; ^{1}H NMR (400 MHz, $CDCl_3$) δ 8.23 – 8.17 (m, 2H), 7.67 – 7.60 (m, 1H), 7.55 – 7.47 (m, 2H), 7.41 – 7.34 (m, 2H), 7.20 – 7.14 (m, 2H), 3.66 – 3.56 (m, 1H), 1.94 (ddd, *J* = 13.8, 8.4, 7.0 Hz, 1H), 1.79 – 1.56 (m, 6H), 1.41 (s, 9H), 1.28 – 1.13 (m, 4H), 0.99 – 0.87 (m, 2H); ^{13}C NMR (101 MHz, $CDCl_3$) δ 173.4, 165.1, 149.8, 137.7, 133.5, 130.1, 129.6, 128.9, 128.5, 121.5, 80.6, 49.3, 41.3, 35.4, 33.2, 33.1, 28.0, 26.5, 26.13, 26.11. IR (film): ν (cm^{-1}) 2878, 2920,

2847, 2361, 2337, 1734, 1717, 1560, 1508, 1449, 1390, 1365, 1267, 1208, 1171, 1145, 1127, 1082, 1062, 1021, 876, 839, 821, 764, 703, 686, 671, 548, 529; HR-MS (ESI) m/z calcd for $C_{26}H_{32}NaO_4^+$ [M+Na$^+$] 431.21928, found 431.21975; $[\alpha]_D^{22.5}$ = -16.9 (c = 0.1, $CHCl_3$); HPLC conditions: NR column, hexane/2-propanol = 98/2, flow rate = 1.0 mL/min, λ = 230 nm, t_R = 23.6 min (minor), t_R = 28.8 min (major), 93:7 er.

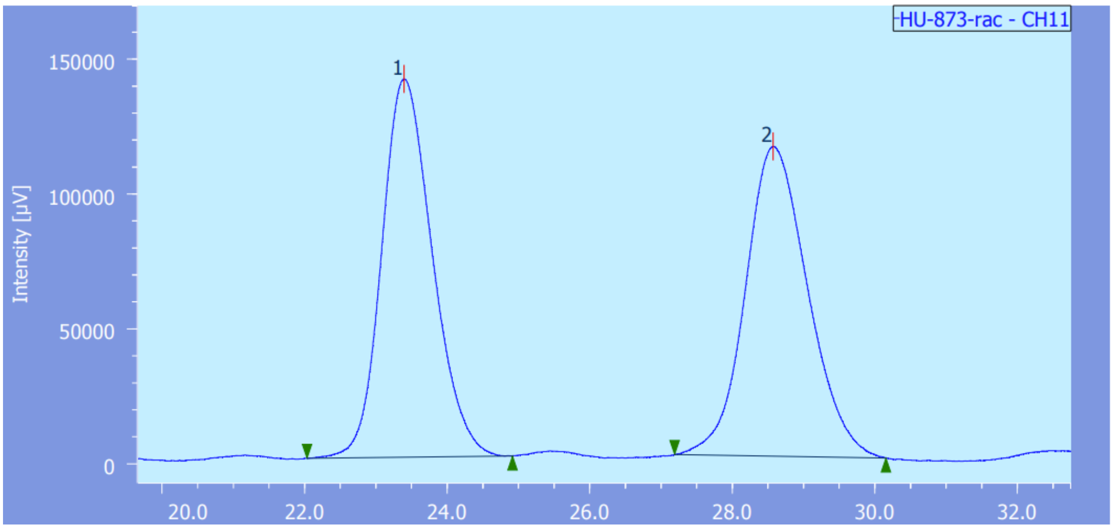

Decision

#	Peak Name	CH	tR [min]	Area [µV·sec]	Height [µV]	Area%	Height%	Quantity	NTP	Resolution	Symmetry Factor	Warning
1	Unknown	11	23.393	6913127	140196	49.701	54.977	N/A	5294	3.619	1.190	
2	Unknown	11	28.570	6996180	114811	50.299	45.023	N/A	5218	N/A	1.116	

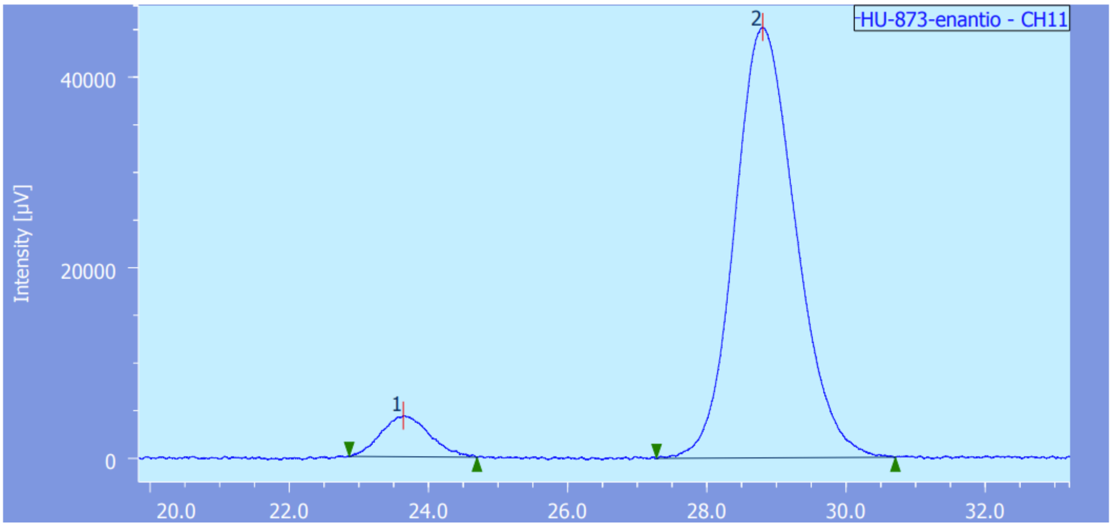

Decision

#	Peak Name	CH	tR [min]	Area [µV·sec]	Height [µV]	Area%	Height%	Quantity	NTP	Resolution	Symmetry Factor	Warning
1	Unknown	11	23.637	209577	4308	7.063	8.702	N/A	5517	3.617	1.207	
2	Unknown	11	28.800	2757690	45199	92.937	91.298	N/A	5250	N/A	1.146	

tert-Butyl (_R_)-3-cyclohexyl-2-(4-phenoxyphenyl)propanoate (15)

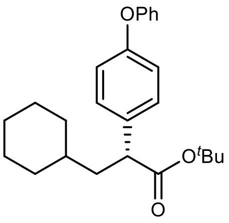

Prepared by **GP-B**. Colorless oil, 47.1 mg, 62% yield. ^{1}H NMR (400 MHz, $CDCl_3$) δ 7.28 – 7.22 (m, 2H), 7.21 – 7.16 (m, 2H), 7.05 – 6.98 (m, 1H), 6.96 – 6.90 (m, 2H), 6.89 – 6.83 (m, 2H), 3.48 (dd, J = 8.5, 7.2 Hz, 1H), 1.85 (ddd, J = 13.7, 8.6, 6.8 Hz, 1H), 1.71 – 1.46 (m, 6H), 1.33 (s, 9H), 1.17 – 1.03 (m, 4H), 0.91 – 0.78 (m, 2H); ^{13}C NMR (101 MHz, $CDCl_3$) δ 173.6, 157.2, 156.0, 135.0, 129.7, 129.1, 123.2, 118.8, 118.7, 80.4, 49.2, 41.3, 35.4,

33.18, 33.15, 28.0, 26.5, 26.18, 26.16. IR (film): ν (cm^{-1}) 2977, 2923, 2851, 2159, 2030, 1976, 1726, 1589, 1504, 1488, 1448, 1366, 1237, 1143, 1129, 970, 870, 844, 752, 692, 524, 511, 483, 418; HR-MS (ESI) m/z calcd for $C_{25}H_{32}NaO_3^+$ [M+Na$^+$] 403.22437, found 403.22436; $[\alpha]_D^{22.3}$ = -28.1 (c = 0.1, $CHCl_3$); HPLC conditions: NR column, hexane/2-propanol = 99/1, flow rate = 1.0 mL/min, λ = 240 nm, t_R = 8.9 min (minor), t_R = 11.7 min (major), 96:4 er.

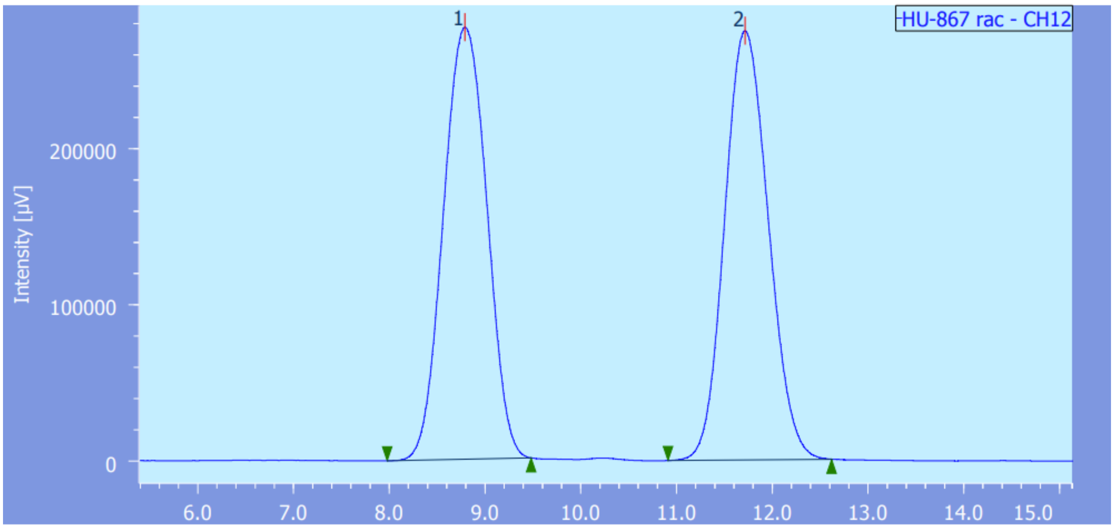

Decision

#	Peak Name	CH	tR [min]	Area [µV·sec]	Height [µV]	Area%	Height%	Quantity	NTP	Resolution	Symmetry Factor	Warning
1	Unknown	12	8.790	8727390	276485	50.001	50.157	N/A	1660	3.427	1.019	
2	Unknown	12	11.713	8727026	274759	49.999	49.843	N/A	3056	N/A	1.114	

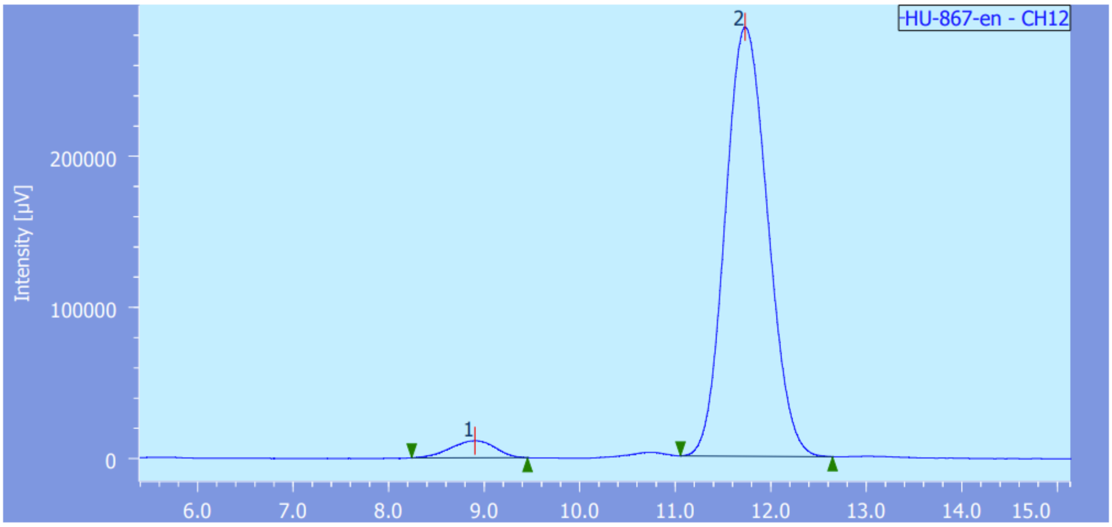

Decision

#	Peak Name	CH	tR [min]	Area [µV·sec]	Height [µV]	Area%	Height%	Quantity	NTP	Resolution	Symmetry Factor	Warning
1	Unknown	12	8.903	372190	11270	4.078	3.819	N/A	1559	3.279	0.900	
2	Unknown	12	11.730	8755247	283807	95.922	96.181	N/A	3221	N/A	1.107	

***tert*-Butyl (*R*)-3-cyclohexyl-2-(4-methoxyphenyl)propanoate (16)**

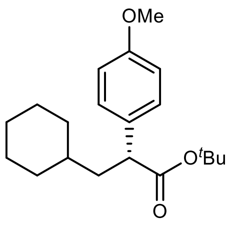

Prepared by **GP-B** (two 40 W 390 nm Kessile lamps were used). Colorless oil, 37.5 mg, 59% yield. ^{1}H NMR (400 MHz, $CDCl_3$) δ 7.25 – 7.18 (m, 2H), 6.87 – 6.81 (m, 2H), 3.79 (s, 3H), 3.55 – 3.48 (m, 1H), 1.89 (ddd, *J* = 13.7, 8.3, 7.0 Hz, 1H), 1.77 – 1.51 (m, 6H), 1.39 (s, 9H), 1.23 – 1.09 (m, 4H), 0.97 – 0.83 (m, 2H); ^{13}C NMR (101 MHz, $CDCl_3$) δ 173.9, 158.4, 132.2, 128.8, 113.8, 80.2, 55.2, 49.0, 41.2, 35.4, 33.2, 33.1, 28.0, 26.5, 26.19, 26.15. IR

(film): ν (cm^{-1}) 2978, 2922, 2850, 1724, 1612, 1510, 1447, 1390, 1366, 1303, 1248, 1177, 1143, 1127, 1037, 971, 843, 831, 796, 762, 583, 530, 466; HR-MS (ESI) m/z calcd for $C_{20}H_{30}NaO_3^+$ [M+Na$^+$] 341.20872, found 340.20842; $[\alpha]_D^{22.5}$ = -29.5 (c = 0.1, $CHCl_3$); HPLC conditions: NR column, hexane/2-propanol = 99/1, flow rate = 1.0 mL/min, λ = 240 nm, t_R = 11.9 min (minor), t_R = 16.9 min (major), 93:7 er.

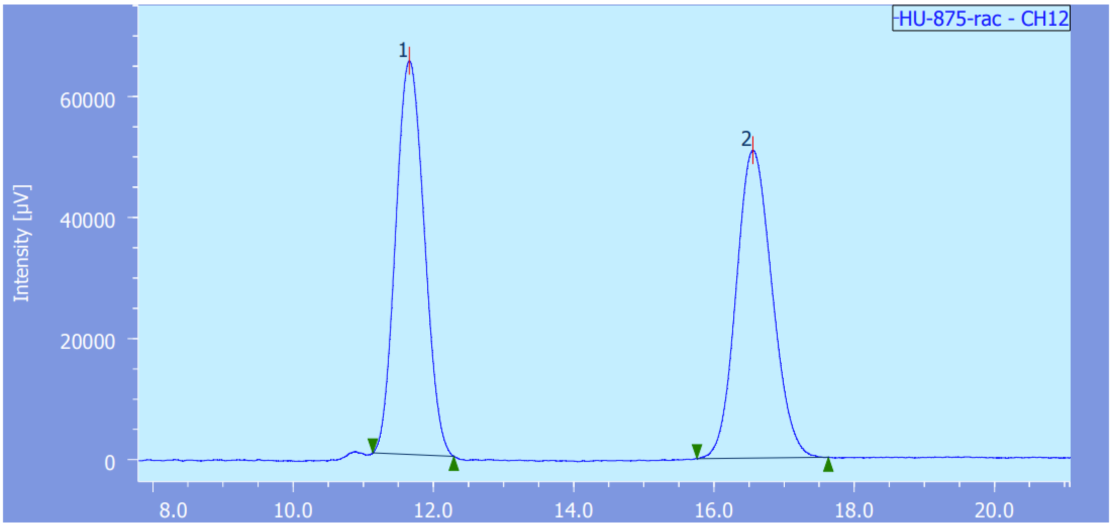

Decision

#	Peak Name	CH	tR [min]	Area [μV·sec]	Height [μV]	Area%	Height%	Quantity	NTP	Resolution	Symmetry Factor	Warning
1	Unknown	12	11.657	1841788	64980	50.410	56.102	N/A	3692	5.748	1.083	
2	Unknown	12	16.553	1811849	50844	49.590	43.898	N/A	4953	N/A	1.117	

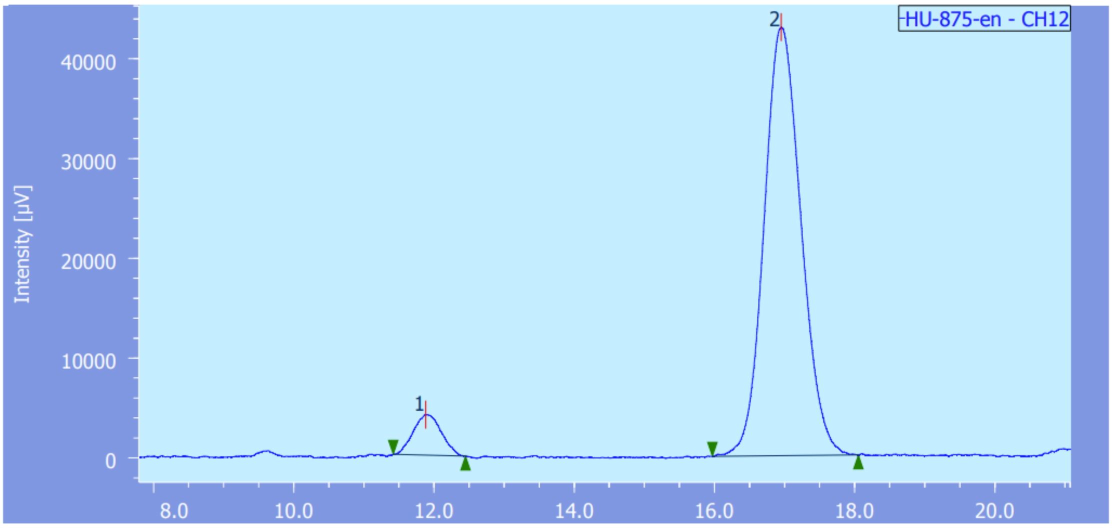

Decision

#	Peak Name	CH	tR [min]	Area [μV·sec]	Height [μV]	Area%	Height%	Quantity	NTP	Resolution	Symmetry Factor	Warning
1	Unknown	12	11.880	113740	4059	6.745	8.642	N/A	3885	5.895	1.096	
2	Unknown	12	16.950	1572639	42908	93.255	91.358	N/A	4965	N/A	1.112	

***tert*-Butyl (*R*)-3-cyclohexyl-2-(4-(methylthio)phenyl)propanoate (17)**

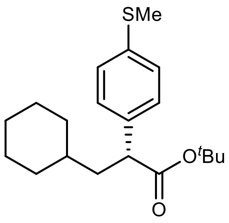

Prepared by **GP-B** (two 40 W 390 nm Kessile lamps were used). Colorless oil, 16.8 mg, 25% yield. ^{1}H NMR (400 MHz, $CDCl_3$) ^{1}H NMR (400 MHz, $CDCl_3$) δ 7.24 – 7.17 (m, 4H), 3.52 (t, *J* = 7.8 Hz, 1H), 2.47 (s, 3H), 1.93 – 1.84 (m, 1H), 1.75 – 1.53 (m, 6H), 1.38 (s, 9H), 1.20 – 1.08 (m, 4H), 0.96 – 0.84 (m, 2H); ^{13}C NMR (101 MHz, $CDCl_3$) δ 173.5, 137.0, 136.6, 128.4, 126.7, 80.5, 49.3, 41.1, 35.3, 33.2, 33.0, 28.0, 26.5, 26.2, 26.1, 15.9. IR (film):

ν (cm^{-1}) 2977, 2920, 2849, 2362, 1725, 1597, 1493, 1478, 1447, 1407, 1390, 1366, 1324, 1272, 1256, 1144, 1092, 1015, 968, 845, 820, 770, 718, 567, 508, 473; HR-MS (ESI) m/z calcd for $C_{20}H_{30}NaO_2S^+$ [M+Na$^+$] 357.18587, found 357.18585; $[\alpha]_D^{22.5}$ = -32.0 (c = 0.1, $CHCl_3$); HPLC conditions: NR column, hexane/2-propanol = 99/1, flow rate = 1.0 mL/min, λ = 240 nm, t_R = 12.5 min (minor), t_R = 21.3 min (major), 94:6 er.

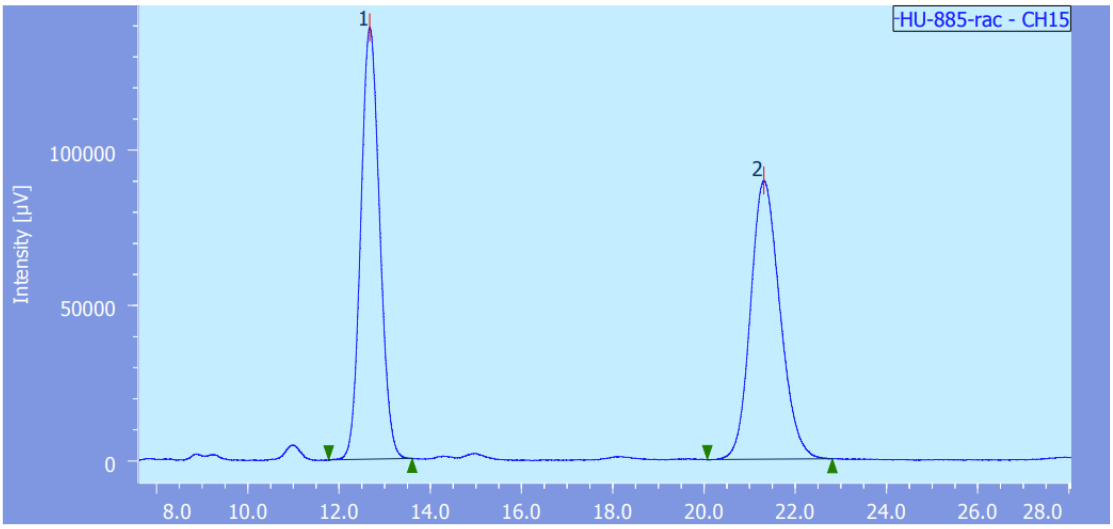

Decision

#	Peak Name	CH	tR [min]	Area [µV·sec]	Height [µV]	Area%	Height%	Quantity	NTP	Resolution	Symmetry Factor	Warning
1	Unknown	15	12.673	4004074	138833	49.880	60.773	N/A	4423	8.951	1.087	
2	Unknown	15	21.307	4023400	89612	50.120	39.227	N/A	5289	N/A	1.168	

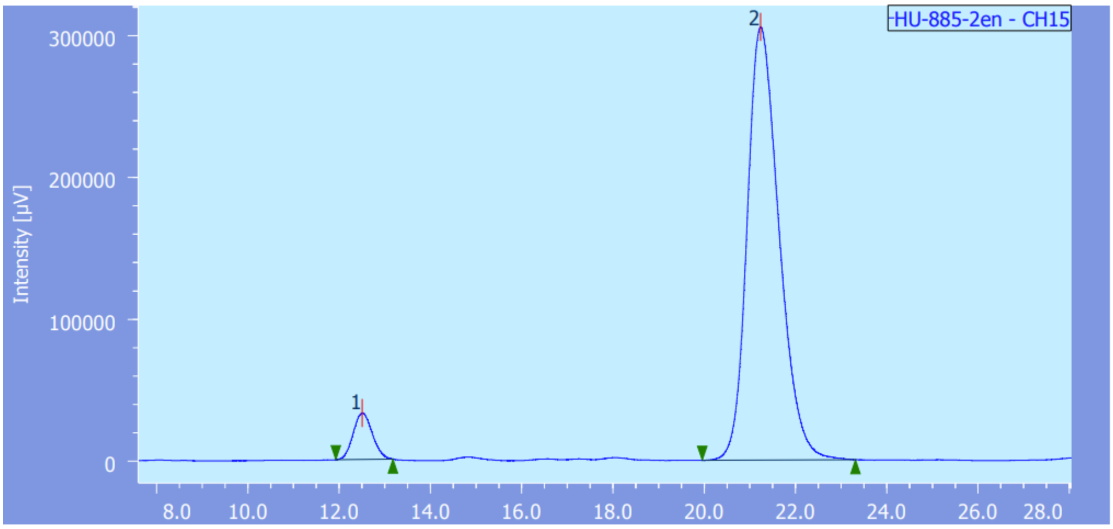

Decision

#	Peak Name	CH	tR [min]	Area [µV·sec]	Height [µV]	Area%	Height%	Quantity	NTP	Resolution	Symmetry Factor	Warning
1	Unknown	15	12.507	959021	32728	6.063	9.689	N/A	4102	8.565	1.092	
2	Unknown	15	21.230	14859341	305064	93.937	90.311	N/A	4533	N/A	1.265	

***tert*-Butyl (*R*)-3-cyclohexyl-2-(2-fluorophenyl)propanoate (18)**

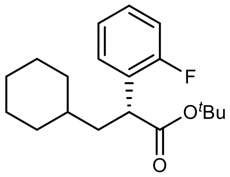

Prepared by **GP-A**. Colorless oil, 30.1 mg, 49% yield. ^{1}H NMR (400 MHz, $CDCl_3$) δ 7.35 (td, *J* = 7.6, 1.8 Hz, 1H), 7.24 – 7.16 (m, 1H), 7.09 (td, *J* = 7.5, 1.2 Hz, 1H), 7.06 – 6.98 (m, 1H), 3.96 (t, *J* = 7.8 Hz, 1H), 1.92 (dt, *J* = 13.9, 7.6 Hz, 1H), 1.78 – 1.55 (m, 6H), 1.40 (s, 9H), 1.20 – 1.11 (m, 4H), 0.97 – 0.85 (m, 2H); ^{13}C NMR (101 MHz, $CDCl_3$) δ 173.0, 160.5 (d, *J* = 246.6 Hz), 128.8 (d, *J* = 4.1 Hz), 128.2 (d, *J* = 8.4 Hz), 127.3 (d, *J* = 15.0 Hz), 124.1 (d, *J* = 3.5 Hz),

115.2 (d, J = 22.8 Hz), 80.6, 41.7 (d, J = 2.5 Hz), 40.2, 35.4, 33.2, 33.0, 27.9, 26.5, 26.2, 26.1; ^{19}F NMR (377 MHz, $CDCl_3$) δ -118.46. IR (film): ν (cm^{-1}) 2977, 2923, 2851, 1728, 1492, 1449, 1391, 1366, 1273, 1250, 1230, 1175, 1146, 1130, 1035, 970, 843, 823, 754, 475; HR-MS (ESI) m/z calcd for $C_{19}H_{27}FNaO_2^+$ [M+Na$^+$] 329.18873, found 329.18879; $[\alpha]_D^{23.7}$ = -28.8 (c = 0.1, $CHCl_3$); HPLC conditions (er was determined by reducing the product to the corresponding alcohol with $LiAlH_4$): Chiral-NR column, hexane/2-propanol = 95/5, flow rate = 1.0 mL/min, λ = 210 nm, t_R = 7.0 min (minor), t_R = 7.9 min (major), 93:7 er.

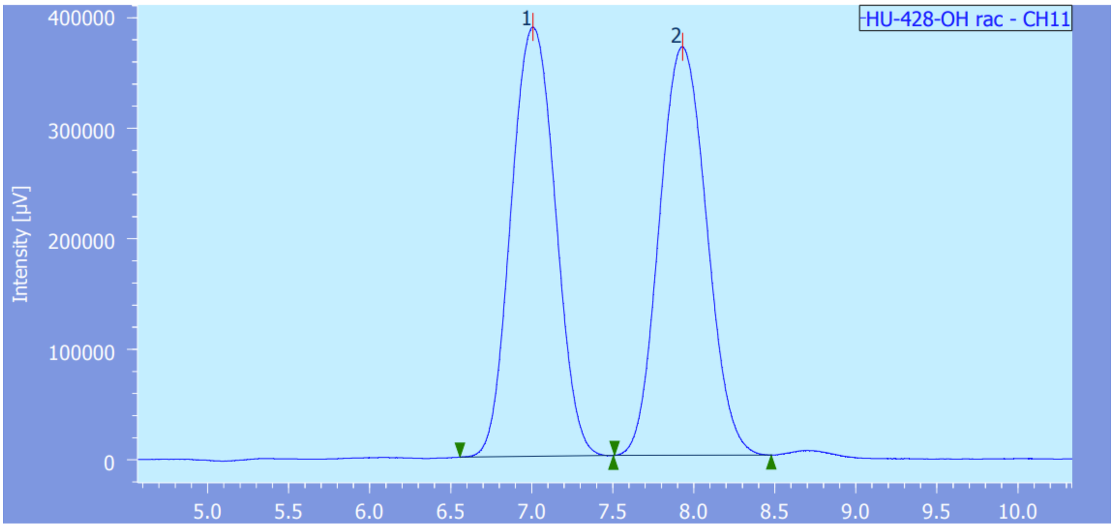

Decision

#	Peak Name	CH	tR [min]	Area [µV·sec]	Height [µV]	Area%	Height%	Quantity	NTP	Resolution	Symmetry Factor	Warning
1	Unknown	11	7.007	7380487	388214	49.586	51.242	N/A	2932	1.736	1.053	
2	Unknown	11	7.930	7503791	369395	50.414	48.758	N/A	3342	N/A	1.079	

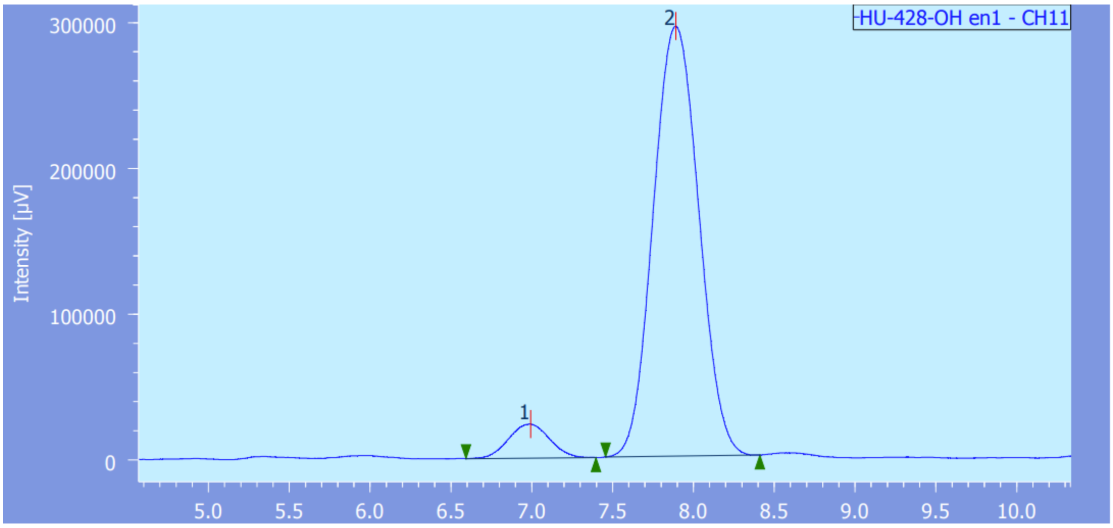

Decision

#	Peak Name	CH	tR [min]	Area [µV·sec]	Height [µV]	Area%	Height%	Quantity	NTP	Resolution	Symmetry Factor	Warning
1	Unknown	11	6.993	412024	23405	6.660	7.355	N/A	3500	1.794	0.992	
2	Unknown	11	7.890	5774602	294795	93.340	92.645	N/A	3550	N/A	1.053	

***tert*-Butyl (*R*)-2-(4-chloro-2-fluorophenyl)-3-cyclohexylpropanoate (19)**

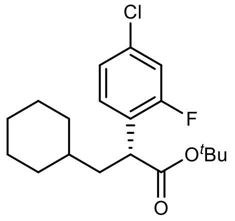

Prepared by **GP-A**. Colorless oil, 34.1 mg, 50% yield. ^{1}H NMR (400 MHz, $CDCl_3$) δ 7.33 – 7.27 (m, 1H), 7.13 – 7.02 (m, 2H), 3.91 (t, *J* = 7.8 Hz, 1H), 1.89 (dt, *J* = 13.8, 7.6 Hz, 1H), 1.77 – 1.52 (m, 6H), 1.39 (s, 9H), 1.20 – 1.06 (m, 4H), 0.98 – 0.83 (m, 2H); ^{13}C NMR (101 MHz, $CDCl_3$) δ 172.54, 160.2 (d, *J* = 250.1 Hz), 133.1 (d, *J* = 10.5 Hz), 129.7 (d, *J* = 4.9 Hz), 125.9 (d, *J* = 15.2 Hz), 124.5 (d, *J* = 3.5 Hz), 116.1 (d, *J* = 26.5 Hz), 81.0, 41.4 (d, *J* = 2.1 Hz), 40.1, 35.4, 33.3, 32.9, 27.9, 26.4, 26.14, 26.09; ^{19}F NMR (377 MHz, $CDCl_3$) δ -115.72. IR (film): ν (cm^{-1}) 2978, 2924, 2853, 1728, 1608, 1579, 1488, 1447, 1410, 1392, 1367, 1257, 1223, 1175, 1147, 1131, 1109, 1078, 972, 896, 854, 763, 592, 477; HR-MS (ESI) m/z calcd for $C_{19}H_{26}ClFNaO_2^+$ [M+Na$^+$] 363.14976, found 363.14976; $[\alpha]_D^{23.8}$ = -31.9 (c = 0.1, $CHCl_3$); HPLC conditions: Chiral-NR column, hexane/2-propanol = 99.8/0.2, flow rate = 0.5 mL/min, λ = 220 nm, t_R = 17.5 min (minor), t_R = 20.3 min (major), 95:5 er.

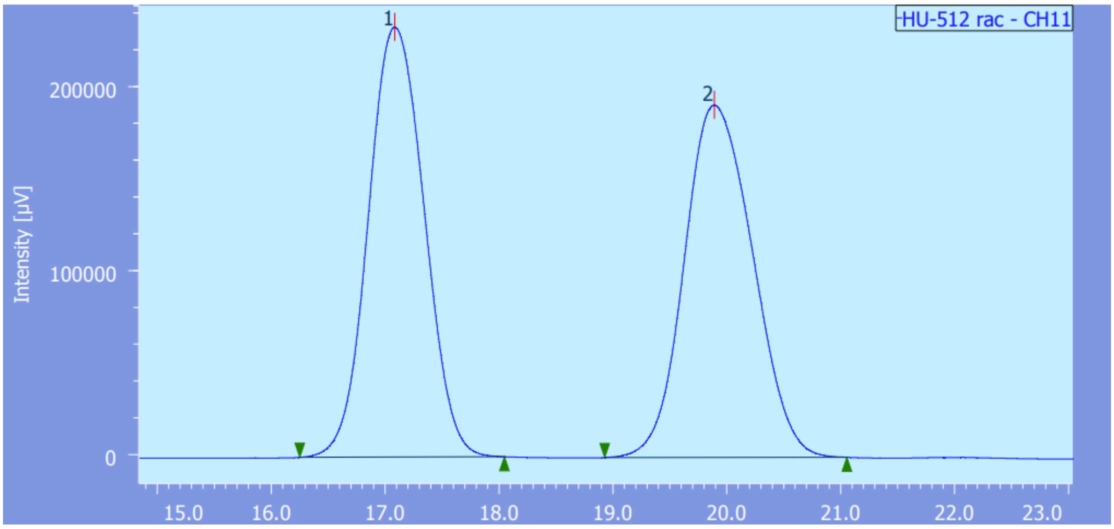

Decision

#	Peak Name	CH	tR [min]	Area [μV·sec]	Height [μV]	Area%	Height%	Quantity	NTP	Resolution	Symmetry Factor	Warning
1	Unknown	11	17.083	7972649	233467	49.913	54.949	N/A	5550	2.752	1.058	
2	Unknown	11	19.890	8000505	191409	50.087	45.051	N/A	4975	N/A	1.125	

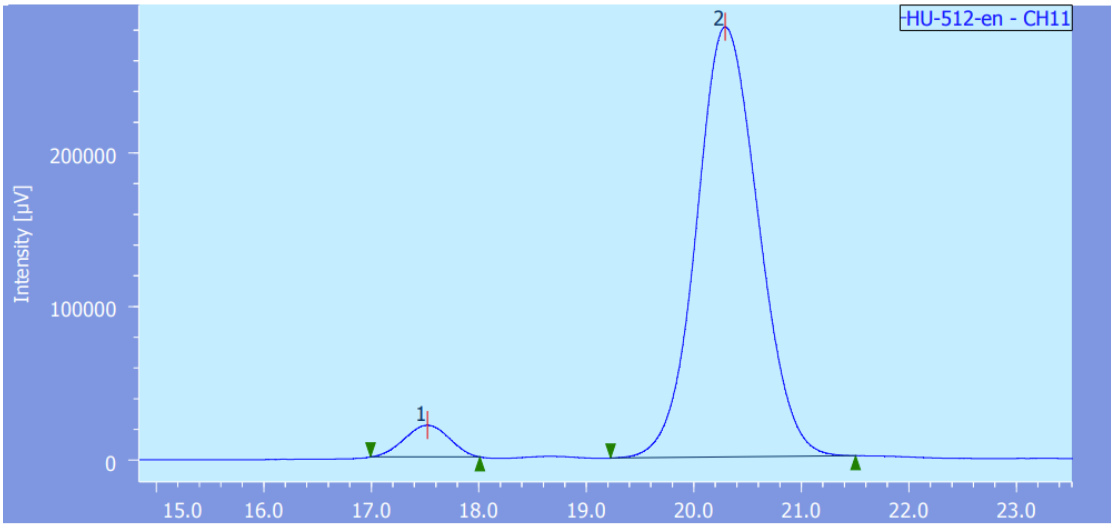

Decision

#	Peak Name	CH	tR [min]	Area [µV·sec]	Height [µV]	Area%	Height%	Quantity	NTP	Resolution	Symmetry Factor	Warning
1	Unknown	11	17.520	601712	20697	5.098	6.885	N/A	7725	3.005	0.974	
2	Unknown	11	20.290	11202117	279911	94.902	93.115	N/A	5963	N/A	1.073	

tert-Butyl (_R_)-3-cyclohexyl-2-(3-fluorophenyl)propanoate (20)

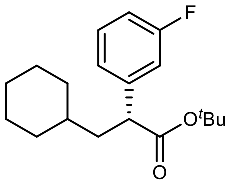

Prepared by **GP-A**. Colorless oil, 41.6 mg, 68% yield. ^{1}H NMR (400 MHz, $CDCl_3$) δ 7.29 – 7.22 (m, 1H), 7.09 – 6.99 (m, 2H), 6.96 – 6.89 (m, 1H), 3.57 (t, J = 8.0 Hz, 1H), 1.91 (ddd, J = 13.7, 8.4, 7.0 Hz, 1H), 1.75 – 1.53 (m, 6H), 1.39 (s, 9H), 1.21 – 1.10 (m, 4H), 0.97 – 0.84 (m, 2H); ^{13}C NMR (101 MHz, $CDCl_3$) δ 173.0, 162.8 (d, J = 246.5 Hz), 142.6 (d, J = 7.6 Hz), 129.7 (d, J = 8.6 Hz), 123.6 (d, J = 2.6 Hz), 114.8 (d, J = 22.0 Hz), 113.7 (d, J = 21.0 Hz), 80.7, 49.6, 41.1, 35.4, 33.2, 33.1, 27.9, 26.5, 26.2, 26.1; ^{19}F NMR (377 MHz, $CDCl_3$) δ -113.28. IR (film): ν (cm^{-1}) 2977, 2923, 2852, 1727, 1614, 1590, 1486, 1448, 1391, 1367, 1253, 1146, 1140, 958, 888, 872, 843, 783, 774, 760, 690, 522; HR-MS (ESI) m/z calcd for $C_{19}H_{27}FNaO_2^+$ [M+Na$^+$] 329.18873, found 329.18871; $[\alpha]_D^{23.7}$ = -26.6 (c = 0.1, $CHCl_3$); HPLC conditions: Chiral-NR column, hexane/2-propanol = 99.8/0.2, flow rate = 0.5 mL/min, λ = 230 nm, t_R = 16.6 min (major), t_R = 18.1 min (minor), 93:7 er.

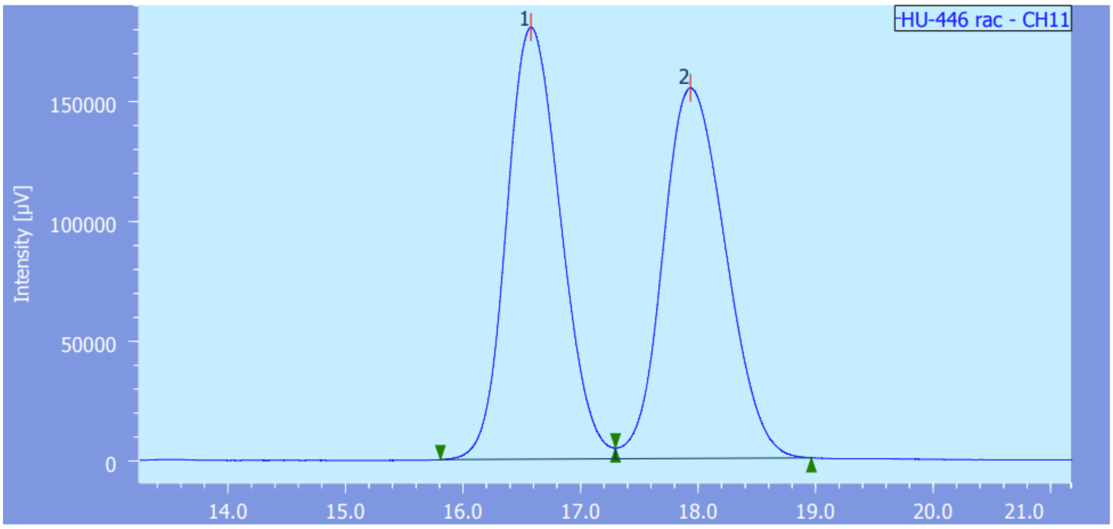

Decision

#	Peak Name	CH	tR [min]	Area [µV·sec]	Height [µV]	Area%	Height%	Quantity	NTP	Resolution	Symmetry Factor	Warning
1	Unknown	11	16.577	5753888	180270	50.036	53.833	N/A	6087	1.476	1.154	
2	Unknown	11	17.933	5745549	154602	49.964	46.167	N/A	5209	N/A	1.153	

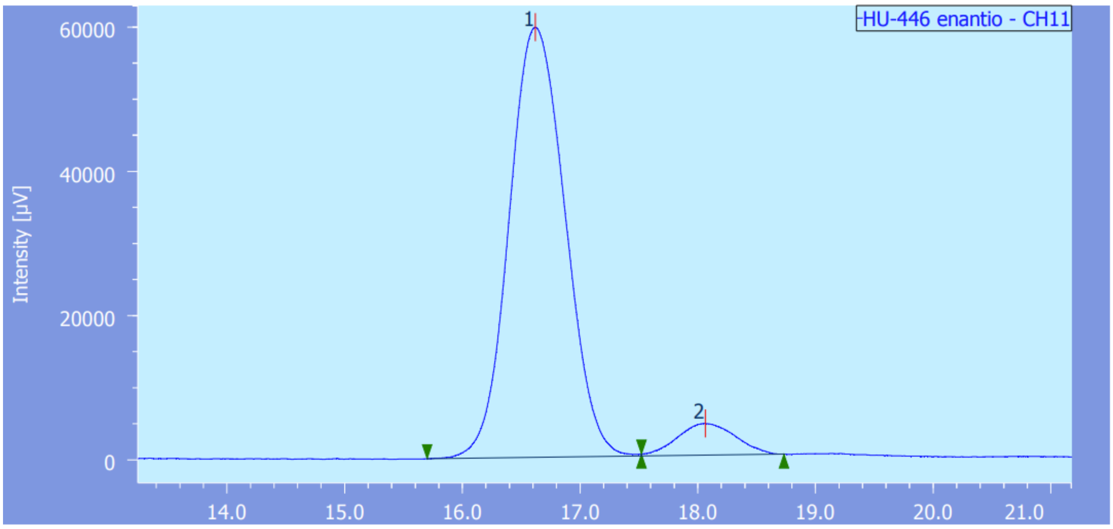

Decision

#	Peak Name	CH	tR [min]	Area [µV·sec]	Height [µV]	Area%	Height%	Quantity	NTP	Resolution	Symmetry Factor	Warning
1	Unknown	11	16.620	1971159	59613	92.865	93.133	N/A	5647	1.587	1.073	
2	Unknown	11	18.063	151437	4396	7.135	6.867	N/A	5920	N/A	N/A	

***tert*-Butyl (*R*)-2-(4-chloro-3-fluorophenyl)-3-cyclohexylpropanoate (21)**

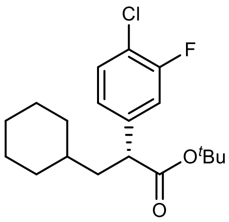

Prepared by **GP-A**. White solid, 47.5 mg, 70% yield. m.p. = 73-74 ºC; ^{1}H NMR (400 MHz, $CDCl_3$) δ 7.31 (t, *J* = 8.0 Hz, 1H), 7.12 (dd, *J* = 10.2, 2.0 Hz, 1H), 7.02 (dd, *J* = 8.3, 1.6 Hz, 1H), 3.54 (t, *J* = 7.8 Hz, 1H), 1.88 (ddd, *J* = 13.7, 8.2, 7.2 Hz, 1H), 1.76 – 1.51 (m, 6H), 1.39 (s, 9H), 1.20 – 1.07 (m, 4H), 0.96 – 0.84 (m, 2H); ^{13}C NMR (101 MHz, $CDCl_3$) δ 172.6, 158.0 (d, *J* = 249.4 Hz), 140.9 (d, *J* = 6.6 Hz), 130.35, 124.4 (d, *J* = 3.5 Hz), 119.3 (d, *J* = 17.8 Hz), 116.1 (d, *J* = 21.5 Hz), 81.0, 49.3 (d, *J* = 1.2 Hz), 41.0, 35.3, 33.2, 33.0, 27.9, 26.4, 26.11, 26.07; ^{19}F NMR (377 MHz, $CDCl_3$) δ -115.39. IR (film): ν (cm^{-1}) 2978, 2924, 2904, 2850, 1717, 1578, 1485, 1449, 1428, 1368, 1340, 1315, 1273, 1257, 1229, 1221, 1137, 1063, 963, 884, 839, 814, 757, 702, 608, 551, 505, 459; HR-MS (ESI) m/z calcd for $C_{19}H_{26}ClFNaO_2^+$ [M+Na$^+$] 363.14976, found 363.14984; $[\alpha]_D^{23.8}$ = -24.8 (c = 0.1, $CHCl_3$); HPLC conditions: Chiral-NR column, hexane/2-propanol = 99.8/0.2, flow rate = 0.5 mL/min, λ = 220 nm, t_R = 19.2 min (minor), t_R = 20.6 min (major), 95:5 er.

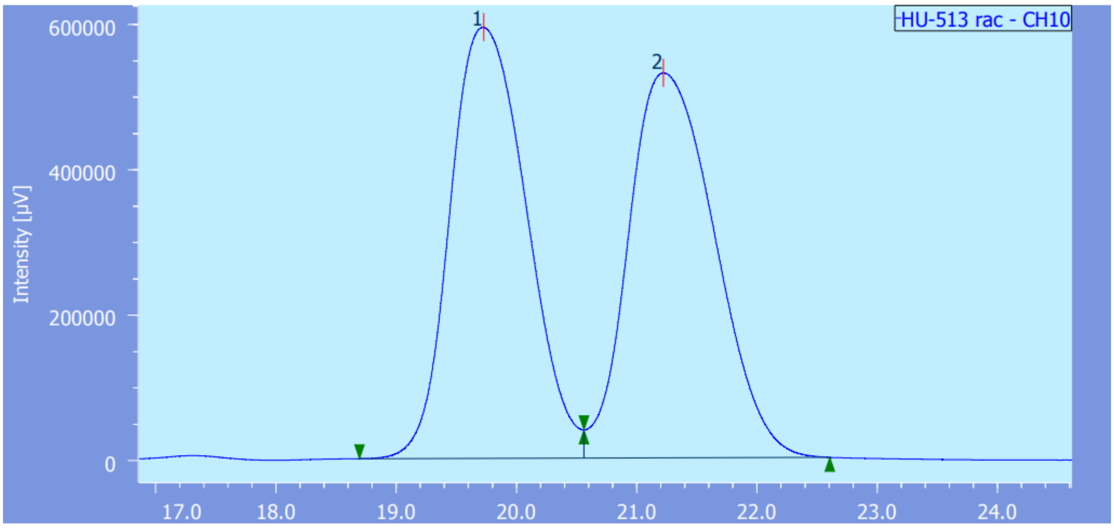

Decision

#	Peak Name	CH	tR [min]	Area [µV·sec]	Height [µV]	Area%	Height%	Quantity	NTP	Resolution	Symmetry Factor	Warning
1	Unknown	10	19.727	26184788	592833	49.773	52.830	N/A	4348	1.175	N/A	
2	Unknown	10	21.220	26423254	529324	50.227	47.170	N/A	3947	N/A	N/A	

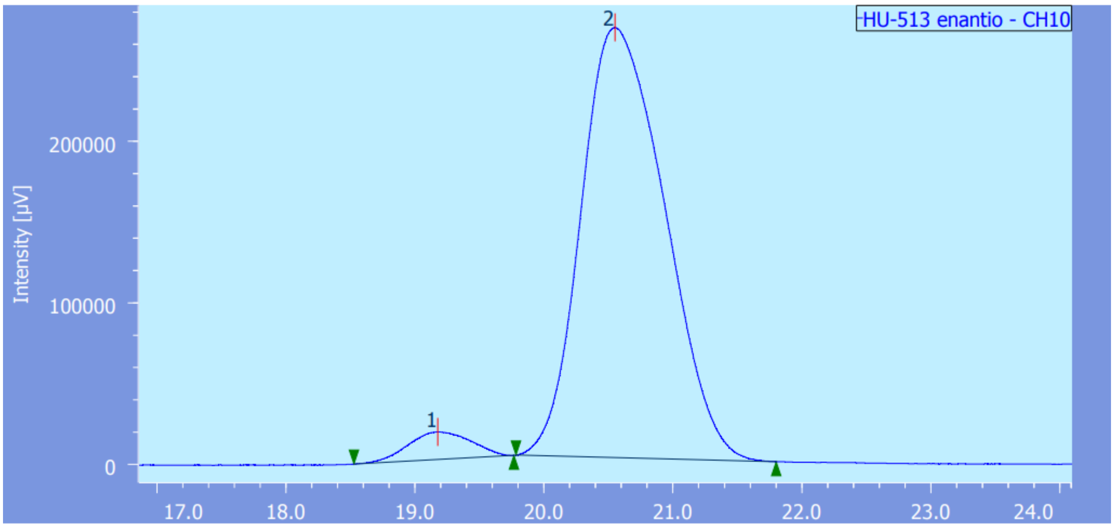

Decision

#	Peak Name	CH	tR [min]	Area [µV·sec]	Height [µV]	Area%	Height%	Quantity	NTP	Resolution	Symmetry Factor	Warning
1	Unknown	10	19.177	580071	17000	4.606	6.004	N/A	6419	1.255	1.007	
2	Unknown	10	20.550	12014501	266135	95.394	93.996	N/A	4410	N/A	1.229	

Dimethyl (*R*)-5-(1-(*tert*-butoxy)-3-cyclohexyl-1-oxopropan-2-yl)isophthalate (22)

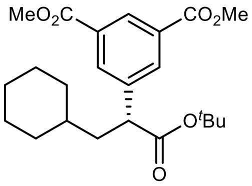

Prepared by **GP-A**. White solid, 63.4 mg, 78% yield. m.p. = 65-66 °C; ^{1}H NMR (400 MHz, CDCl$_3$) δ 8.57 (t, *J* = 1.6 Hz, 1H), 8.18 (d, *J* = 1.6 Hz, 2H), 3.94 (s, 6H), 3.70 (dd, *J* = 8.2, 7.4 Hz, 1H), 1.98 (ddd, *J* = 13.8, 8.4, 6.9 Hz, 1H), 1.77 – 1.58 (m, 6H), 1.39 (s, 9H), 1.20 – 1.09 (m, 4H), 0.99 – 0.85 (m, 2H); ^{13}C NMR (101 MHz, CDCl$_3$) δ 172.6, 166.2, 141.1, 133.4, 130.8, 129.4, 81.1, 52.4, 49.6, 41.1, 35.4, 33.1, 33.0, 27.9, 26.4, 26.11, 26.09. IR (film): ν (cm^{-1}) 2979, 2921, 2849, 1718, 1604, 1450, 1435, 1366, 1319, 1239, 1199, 1146, 1125, 1108, 1003, 964, 909, 851, 844, 747, 720, 470; HR-MS (ESI) m/z calcd for $C_{23}H_{32}NaO_6^+$ [M+Na$^+$] 427.20911, found 427.20915; $[\alpha]_D^{23.9}$ = -8.2 (c = 0.1, CHCl$_3$); HPLC conditions: Chiral-NR column, hexane/2-propanol = 98/2, flow rate = 1.0 mL/min, λ = 240 nm, t_R = 27.9 min (major), t_R = 35.0 min (minor), 95:5 er.

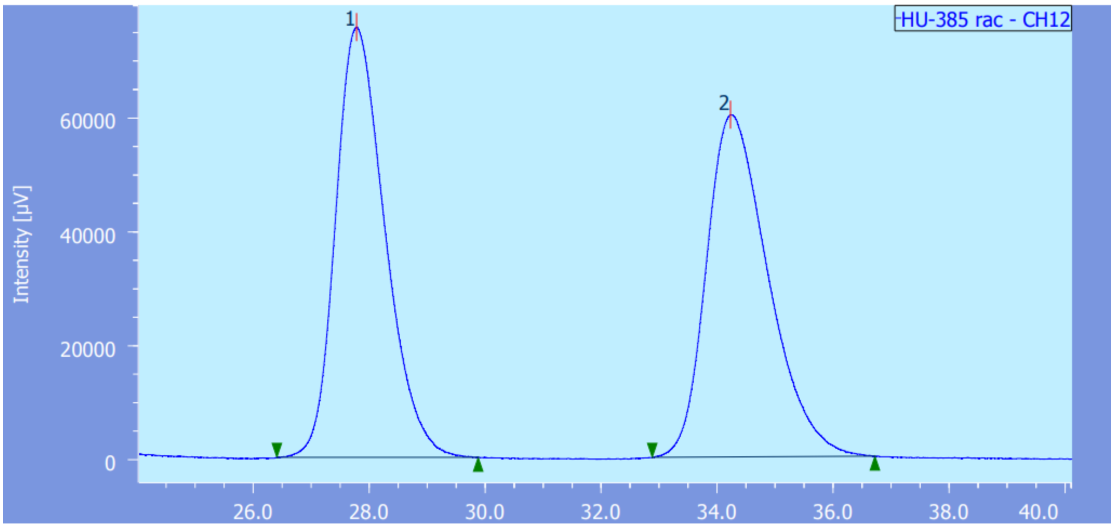

Decision

#	Peak Name	CH	tR [min]	Area [µV·sec]	Height [µV]	Area%	Height%	Quantity	NTP	Resolution	Symmetry Factor	Warning
1	Unknown	12	27.780	4467267	75500	50.029	55.689	N/A	5193	3.719	1.281	
2	Unknown	12	34.230	4462026	60075	49.971	44.311	N/A	5003	N/A	1.385	

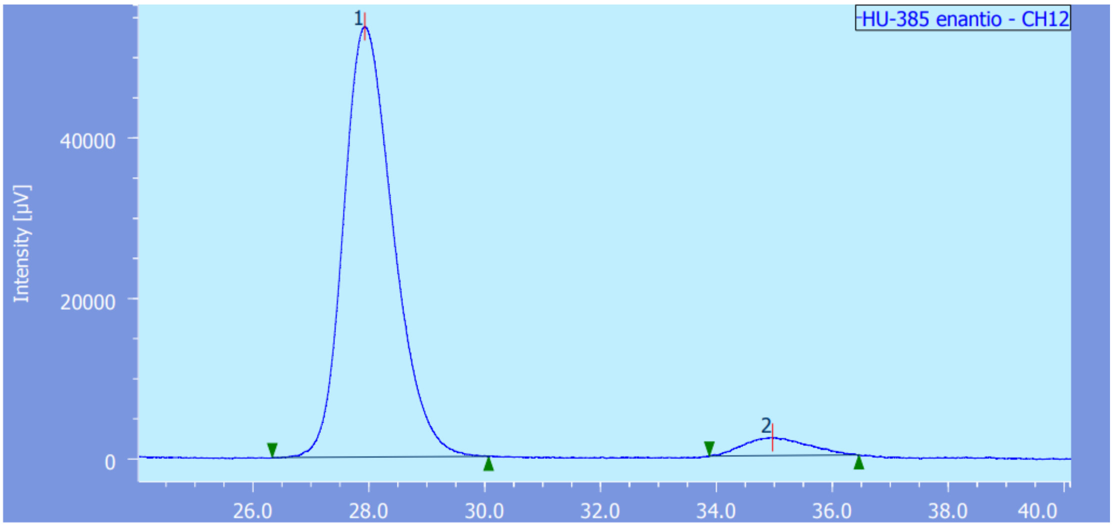

Decision

#	Peak Name	CH	tR [min]	Area [µV·sec]	Height [µV]	Area%	Height%	Quantity	NTP	Resolution	Symmetry Factor	Warning
1	Unknown	12	27.927	3199262	53628	94.792	95.942	N/A	5211	3.766	1.261	
2	Unknown	12	34.967	175781	2268	5.208	4.058	N/A	4038	N/A	1.186	

Methyl (*R*)-3-(1-(*tert*-butoxy)-3-cyclohexyl-1-oxopropan-2-yl)-5-chlorobenzoate (23)

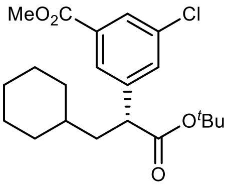

Prepared by **GP-A**. White solid, 51.8 mg, 68% yield. m.p. = 99-100 ^{o}C; ^{1}H NMR (400 MHz, $CDCl_3$) δ 7.91 – 7.87 (m, 1H), 7.85 (t, *J* = 1.5 Hz, 1H), 7.50 (t, *J* = 1.8 Hz, 1H), 3.91 (s, 3H), 3.60 (dd, *J* = 8.4, 7.2 Hz, 1H), 1.93 (ddd, *J* = 13.8, 8.5, 6.9 Hz, 1H), 1.76 – 1.52 (m, 6H), 1.39 (s, 9H), 1.22 – 1.08 (m, 4H), 0.98 – 0.82 (m, 2H); ^{13}C NMR (101 MHz, $CDCl_3$) δ 172.5, 165.8, 142.4, 134.5, 132.4, 131.8, 128.2, 127.5, 81.1, 52.4, 49.5, 41.1, 35.4, 33.1, 33.0, 27.9, 26.4, 26.08, 26.07. IR (film): ν (cm^{-1}) 2985, 2917, 2852, 1718, 1580, 1448, 1433, 1367, 1349, 1284, 1240, 1218, 1195, 1146, 1128, 1117, 996, 972, 882, 846, 796, 766, 754, 738, 703, 673, 625, 558, 502; HR-MS (ESI) m/z calcd for $C_{21}H_{29}ClNaO_4^+$ [M+Na$^+$] 403.16466, found 403.16469; $[\alpha]_D^{23.9}$ = -10.8 (c = 0.1, $CHCl_3$); HPLC conditions: Chiral-NR column, hexane/2-propanol = 98/2, flow rate = 1.0 mL/min, λ = 220 nm, t_R = 8.2 min (major), t_R = 9.7 min (minor), 96:4 er.

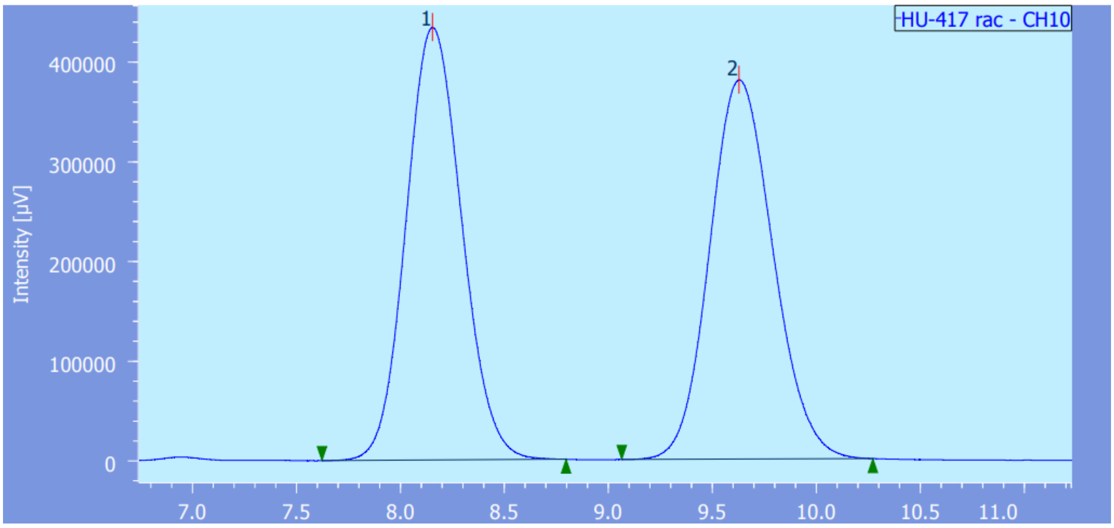

Decision

#	Peak Name	CH	tR [min]	Area [µV·sec]	Height [µV]	Area%	Height%	Quantity	NTP	Resolution	Symmetry Factor	Warning
1	Unknown	10	8.153	8082854	433913	50.006	53.295	N/A	4388	2.806	1.100	
2	Unknown	10	9.627	8080996	380252	49.994	46.705	N/A	4715	N/A	1.127	

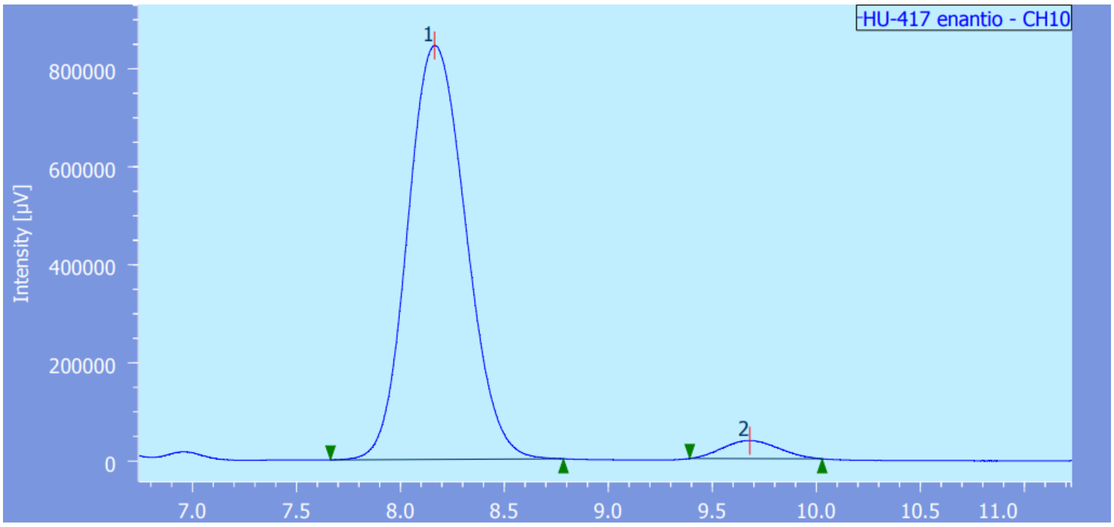

Decision

#	Peak Name	CH	tR [min]	Area [µV·sec]	Height [µV]	Area%	Height%	Quantity	NTP	Resolution	Symmetry Factor	Warning
1	Unknown	10	8.163	16420408	844200	95.947	95.820	N/A	3957	2.928	1.128	
2	Unknown	10	9.680	693577	36829	4.053	4.180	N/A	5553	N/A	1.083	

***tert*-Butyl (*R*)-3-cyclohexyl-2-(naphthalen-2-yl)propanoate (24)**

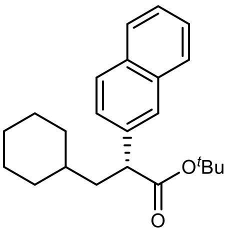

Prepared by **GP-B**. White solid, 49.5 mg, 73% yield. m.p. = 81-82 °C; ^{1}H NMR (400 MHz, CDCl$_3$) δ 7.86 – 7.78 (m, 3H), 7.75 (d, J = 1.3 Hz, 1H), 7.52 – 7.41 (m, 3H), 3.76 (t, J = 7.8 Hz, 1H), 2.03 (ddd, J = 13.7, 8.2, 7.2 Hz, 1H), 1.85 – 1.57 (m, 6H), 1.40 (s, 9H), 1.25 – 1.10 (m, 4H), 1.02 – 0.89 (m, 2H); ^{13}C NMR (101 MHz, CDCl$_3$) δ 173.6, 137.6, 133.4, 132.5, 128.0, 127.8, 127.6, 126.6, 126.1, 125.9, 125.5, 80.5, 50.0, 41.1, 35.4, 33.3, 33.1, 28.0, 26.5, 26.2, 26.1. IR (film): ν (cm^{-1}) 2960, 2924, 2848, 1719, 1632, 1600, 1507, 1477, 1446, 1392, 1365, 1339, 1310, 1273, 1249, 1219, 1172, 1146, 1139, 1092, 978, 952, 896, 859, 839, 757, 746, 652, 517, 476; HR-MS (ESI) m/z calcd for $C_{23}H_{30}NaO_2^+$ [M+Na$^+$] 361.21380, found 361.21384; $[\alpha]_D^{24.0}$ = -26.5 (c = 0.1, CHCl$_3$); HPLC conditions: IB column, hexane/2-propanol = 99.8/0.2, flow rate = 0.5 mL/min, λ = 240 nm, t_R = 17.9 min (major), t_R = 20.4 min (minor), 97:3 er.

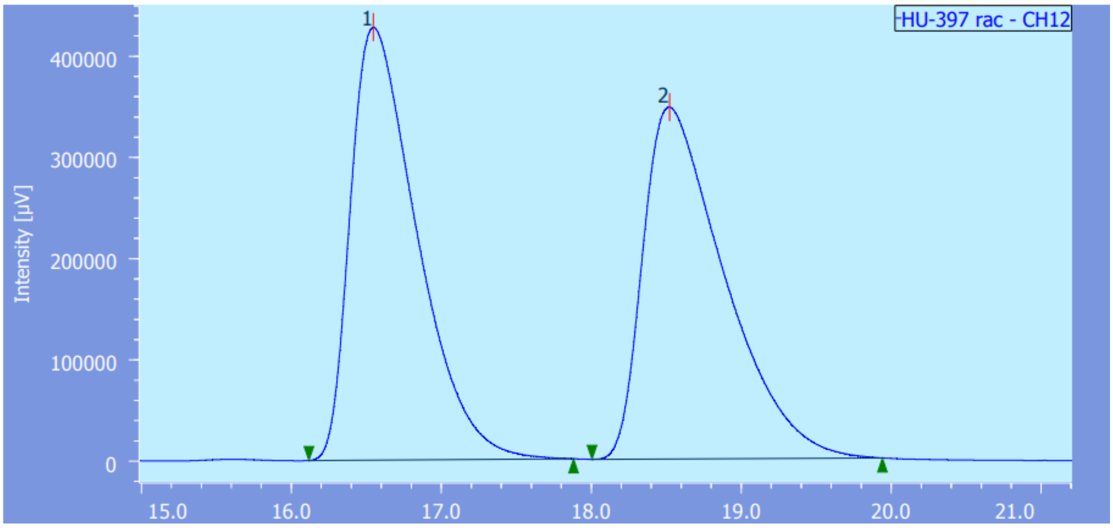

Decision

#	Peak Name	CH	tR [min]	Area [µV·sec]	Height [µV]	Area%	Height%	Quantity	NTP	Resolution	Symmetry Factor	Warning
1	Unknown	12	16.547	13205011	427298	50.113	55.159	N/A	6725	2.206	1.720	
2	Unknown	12	18.520	13145306	347370	49.887	44.841	N/A	5640	N/A	1.840	

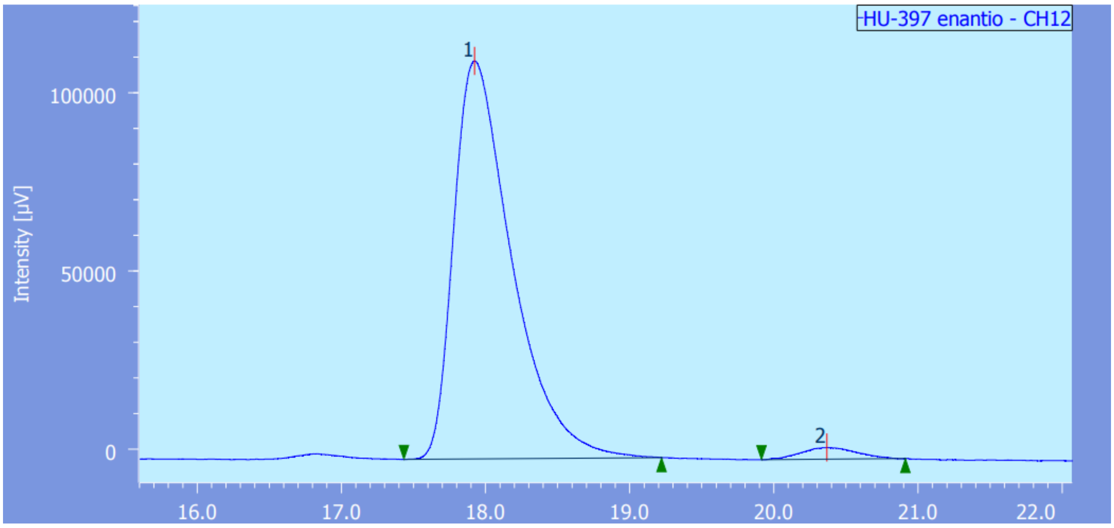

Decision

#	Peak Name	CH	tR [min]	Area [µV·sec]	Height [µV]	Area%	Height%	Quantity	NTP	Resolution	Symmetry Factor	Warning
1	Unknown	12	17.923	3133501	111555	97.277	97.160	N/A	10160	3.399	1.714	
2	Unknown	12	20.367	87724	3260	2.723	2.840	N/A	12453	N/A	1.065	

***tert*-Butyl (*R*)-3-cyclohexyl-2-(9H-fluoren-3-yl)propanoate (25)**

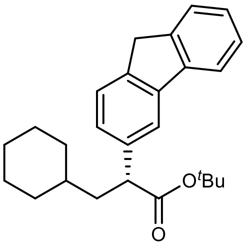

Prepared by **GP-B**. White solid, 53.4 mg, 71% yield. m.p. = 61-62 ºC; ^{1}H NMR (400 MHz, $CDCl_3$) δ 7.79 –7.70 (m, 2H), 7.57 – 7.48 (m, 2H), 7.40 – 7.27 (m, 3H), 3.90 (s, 2H), 3.67 (dd, *J* = 8.2, 7.4 Hz, 1H), 2.00 (ddd, *J* = 13.7, 8.4, 7.0 Hz, 1H), 1.84 – 1.59 (m, 6H), 1.42 (s, 9H), 1.28 – 1.12 (m, 4H), 1.01 – 0.87 (m, 2H); ^{13}C NMR (101 MHz, $CDCl_3$) δ 173.8, 143.5, 143.3, 141.5, 140.5, 138.8, 126.7, 126.6, 126.5, 125.0, 124.4, 119.7 (two peaks) , 80.4, 50.0, 41.3, 36.8, 35.5, 33.3, 33.1, 28.0, 26.5, 26.17, 26.15. IR (film): ν (cm^{-1}) 2981, 2920, 2899, 2844, 1724, 1468, 1447, 1431, 1390, 1364, 1356, 1323, 1268, 1256, 1243, 1218, 1174, 1147, 1135, 1096, 1090, 1002, 972, 881, 860, 844, 833, 761, 735, 589, 422; HR-MS (ESI) m/z calcd for $C_{26}H_{32}NaO_2^+$ [M+Na$^+$] 399.22945, found 399.22951; $[\alpha]_D^{24.0}$ = -24.6 (c = 0.1, $CHCl_3$); HPLC conditions: Chiral-NR column, hexane/2-propanol = 99/1, flow rate = 1.0

mL/min, λ = 210 nm, t_R = 32.3 min (minor), t_R = 39.5 min (major), 96:4 er.

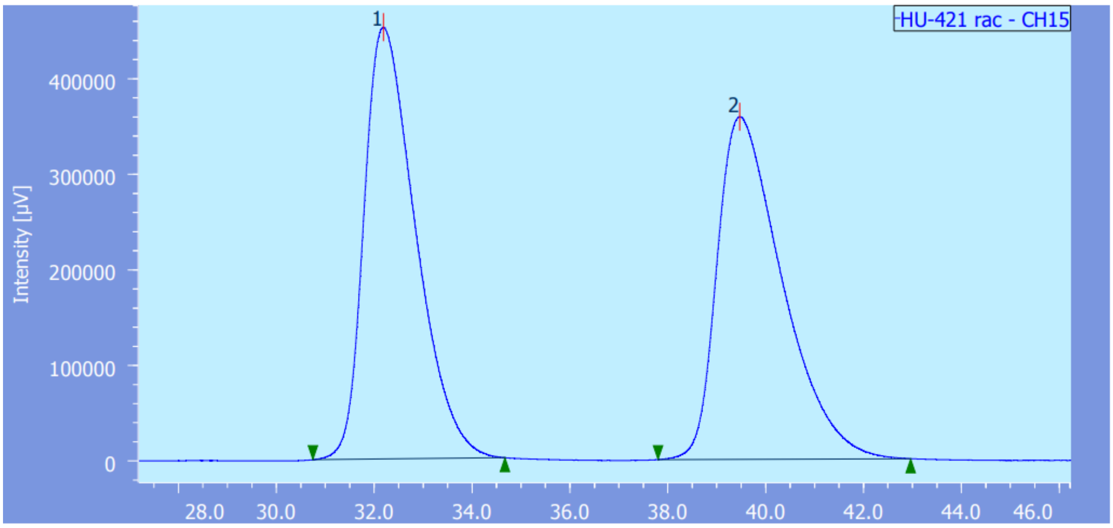

Decision

#	Peak Name	CH	tR [min]	Area [µV·sec]	Height [µV]	Area%	Height%	Quantity	NTP	Resolution	Symmetry Factor	Warning
1	Unknown	15	32.187	33094713	451547	49.950	55.764	N/A	4504	3.378	1.483	
2	Unknown	15	39.463	33160925	358199	50.050	44.236	N/A	4321	N/A	1.661	

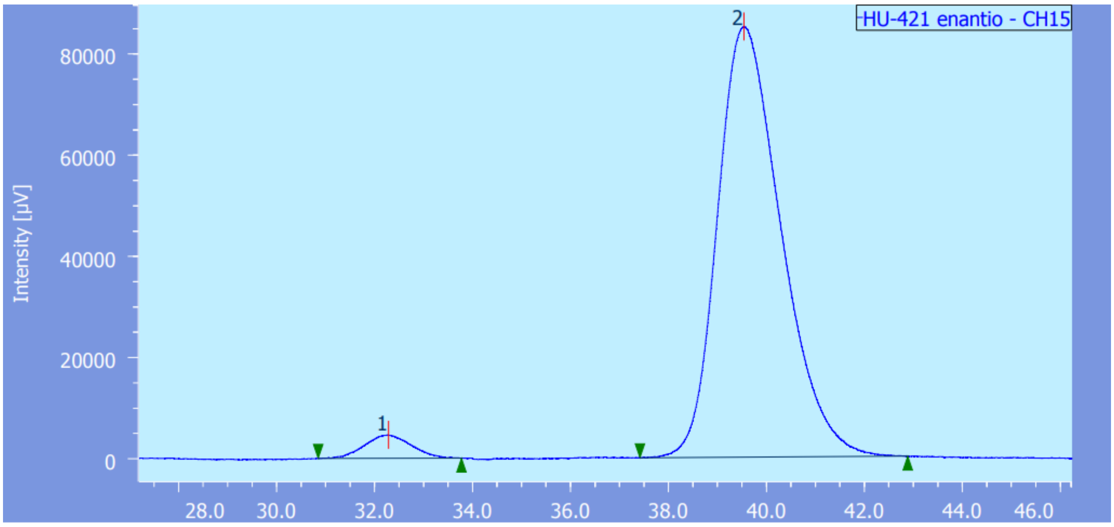

Decision

#	Peak Name	CH	tR [min]	Area [µV·sec]	Height [µV]	Area%	Height%	Quantity	NTP	Resolution	Symmetry Factor	Warning
1	Unknown	15	32.283	306750	4623	3.846	5.156	N/A	5175	3.505	1.022	
2	Unknown	15	39.540	7669135	85043	96.154	94.844	N/A	4506	N/A	1.311	

***tert*-Butyl (*R*)-3-cyclohexyl-2-(phenanthren-3-yl)propanoate (26)**

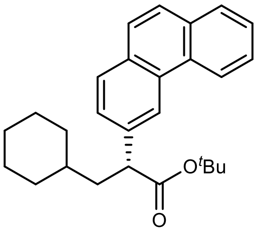

Prepared by **GP-B**. White solid, 51.3 mg, 66% yield. m.p. = 96-97 °C; ^{1}H NMR (400 MHz, $CDCl_3$) δ 8.73 (d, *J* = 8.2 Hz, 1H), 8.62 (d, *J* = 1.3 Hz, 1H), 7.93 – 7.88 (m, 1H), 7.86 (d, *J* = 8.2 Hz, 1H), 7.76 – 7.64 (m, 3H), 7.64 – 7.56 (m, 2H), 3.88 (t, *J* = 7.8 Hz, 1H), 2.11 (ddd, *J* = 13.8, 8.3, 7.1 Hz, 1H), 1.89 – 1.58 (m, 6H), 1.42 (s, 9H), 1.32 – 1.12 (m, 4H), 1.06 – 0.92 (m, 2H); ^{13}C NMR (101 MHz, $CDCl_3$) δ 173.6, 138.5, 132.1, 131.0, 130.3, 130.2, 128.7, 128.5, 126.6, 126.6, 126.52, 126.47, 122.7, 121.8, 80.6, 50.5, 41.5, 35.5, 33.3, 33.2, 28.0, 26.5, 26.2, 26.1. IR (film): ν (cm^{-1}) 2971, 2923, 2846, 1724, 1602, 1508, 1448, 1431, 1392, 1366, 1338, 1310, 1272, 1256, 1244, 1224, 1142, 1127, 1090, 1039, 972, 874, 848, 839, 807, 753, 632, 620, 554, 513, 430; HR-MS (ESI) m/z calcd for $C_{27}H_{32}NaO_2^+$

[M+Na$^+$] 411.22945, found 411.22958; $[\alpha]_D^{24.0}$ = -37.6 (c = 0.1, $CHCl_3$); HPLC conditions: Chiral-NR column, hexane/2-propanol = 99/1, flow rate = 1.0 mL/min, λ = 254 nm, t_R = 50.8 min (major), t_R = 58.8 min (minor), 96:4 er.

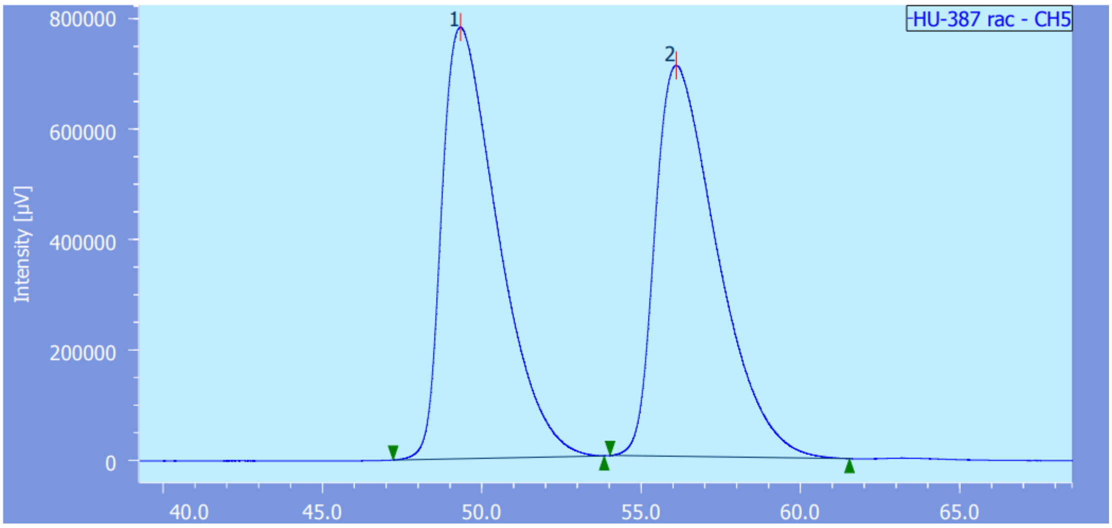

Decision

#	Peak Name	CH	tR [min]	Area [µV·sec]	Height [µV]	Area%	Height%	Quantity	NTP	Resolution	Symmetry Factor	Warning
1	Unknown	5	49.330	95645024	781087	49.835	52.451	N/A	3798	2.011	1.800	
2	Unknown	5	56.100	96277413	708075	50.165	47.549	N/A	3997	N/A	1.732	

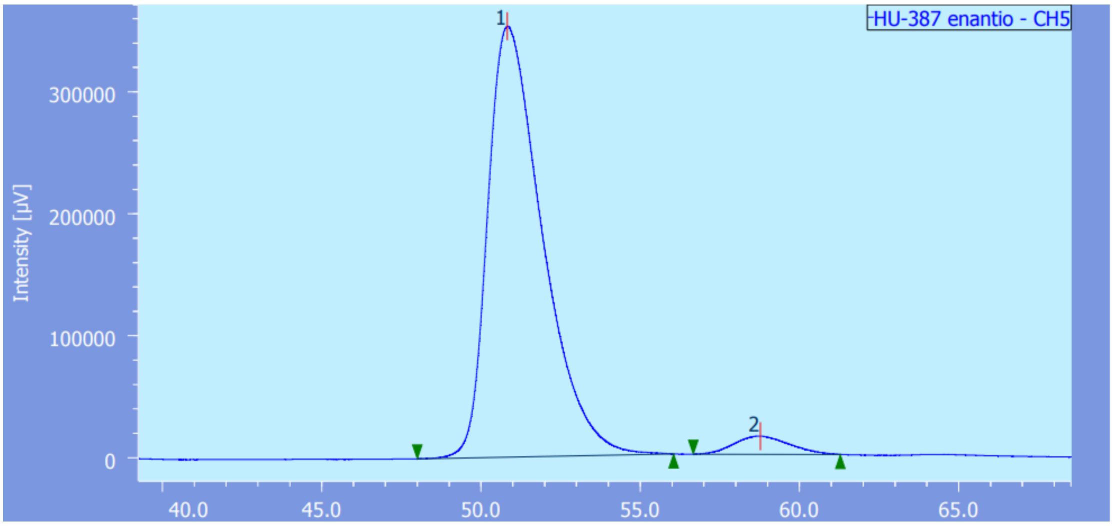

Decision

#	Peak Name	CH	tR [min]	Area [µV·sec]	Height [µV]	Area%	Height%	Quantity	NTP	Resolution	Symmetry Factor	Warning
1	Unknown	5	50.823	42186669	353059	95.901	95.953	N/A	4371	2.509	1.602	
2	Unknown	5	58.767	1803114	14889	4.099	4.047	N/A	5158	N/A	1.127	

***tert*-Butyl (*R*)-3-cyclohexyl-2-(2-fluoro-[1,1'-biphenyl]-4-yl)propanoate (27)**

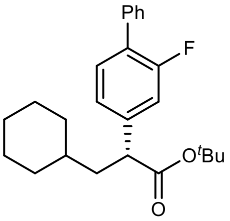

Prepared by **GP-B**. White solid, 55.1 mg, 72% yield. m.p. = 79-80 °C; ^{1}H NMR (400 MHz, $CDCl_3$) δ 7.59 – 7.53 (m, 2H), 7.48 – 7.32 (m, 4H), 7.18 – 7.10 (m, 2H), 3.62 (t, *J* = 8.0 Hz, 1H), 1.96 (ddd, *J* = 13.7, 8.5, 6.9 Hz, 1H), 1.82 – 1.57 (m, 6H), 1.44 (s, 9H), 1.28 – 1.14 (m, 4H), 1.02 – 0.88 (m, 2H); ^{13}C NMR (101 MHz, $CDCl_3$) δ 173.0, 159.6 (d, *J* = 248.8 Hz), 141.5 (d, *J* = 7.7 Hz), 135.6 (d, *J* = 1.2 Hz), 130.5 (d, *J* = 3.9 Hz), 128.9 (d, *J* = 2.9 Hz), 128.4, 127.5, 127.4 (d, *J* = 13.5 Hz), 123.9 (d, *J* = 3.2 Hz), 115.5 (d, *J* = 23.6 Hz), 80.8, 49.4, 41.2, 35.4, 33.2, 33.1, 28.0, 26.5, 26.15, 26.12; ^{19}F NMR (377 MHz, $CDCl_3$) δ -118.01. IR (film): ν (cm-

[1]) 2972, 2922, 2851, 1717, 1624, 1580, 1562, 1514, 1485, 1447, 1423, 1390, 1365, 1354, 1271, 1254, 1208, 1155, 1138, 1075, 1010, 964, 869, 836, 821, 760, 727, 691, 636, 599, 574, 518, 463; HR-MS (ESI) m/z calcd for $C_{25}H_{31}FNaO_2^+$ [M+Na$^+$] 405.22003, found 405.22002; $[\alpha]_D^{23.8}$ = -18.1 (c = 0.1, $CHCl_3$); HPLC conditions: Chiral-NR column, hexane/2-propanol = 99/1, flow rate = 1.0 mL/min, λ = 254 nm, t_R = 7.4 min (minor), t_R = 8.5 min (major), 97:3 er.

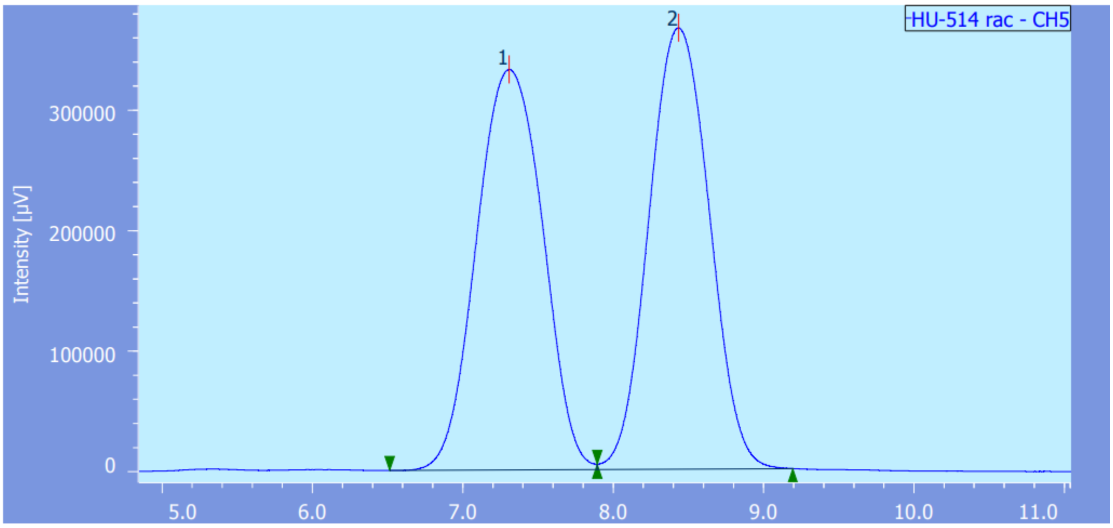

Decision

#	Peak Name	CH	tR [min]	Area [μV·sec]	Height [μV]	Area%	Height%	Quantity	NTP	Resolution	Symmetry Factor	Warning
1	Unknown	5	7.307	10183732	332283	49.838	47.574	N/A	1194	1.407	1.012	
2	Unknown	5	8.433	10249776	366166	50.162	52.426	N/A	1972	N/A	1.044	

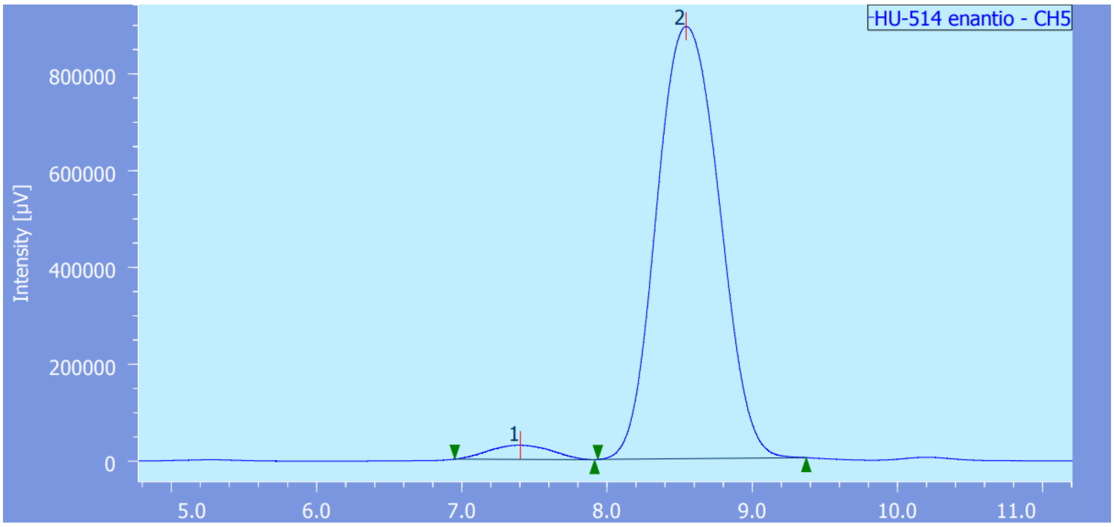

Decision

#	Peak Name	CH	tR [min]	Area [μV·sec]	Height [μV]	Area%	Height%	Quantity	NTP	Resolution	Symmetry Factor	Warning
1	Unknown	5	7.403	884533	29254	3.201	3.175	N/A	1178	1.356	1.008	
2	Unknown	5	8.543	26750393	892090	96.799	96.825	N/A	1727	N/A	1.088	

tert-Butyl (_R_)-3-cyclohexyl-2-(6-methoxynaphthalen-2-yl)propanoate (28)

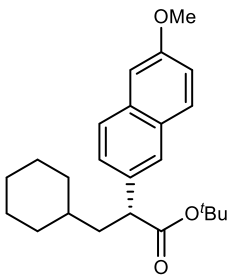

Prepared by **GP-B**. White solid, 40.5 mg, 55% yield. m.p. = 76-77 °C; ^{1}H NMR (400 MHz, $CDCl_3$) δ 7.74 – 7.63 (m, 3H), 7.43 (dd, J = 8.5, 1.7 Hz, 1H), 7.17 – 7.09 (m, 2H), 3.91 (s, 3H), 3.71 (t, J = 7.8 Hz, 1H), 2.00 (ddd, J = 13.8, 8.1, 7.3 Hz, 1H), 1.83 – 1.59 (m, 6H), 1.39 (s, 9H), 1.25 – 1.09 (m, 4H), 1.00 – 0.88 (m, 2H); ^{13}C NMR (101 MHz, $CDCl_3$) δ 173.7, 157.5, 135.3, 133.6, 129.3, 128.9, 126.8, 126.6, 126.4, 118.7, 105.5, 80.4, 55.3, 49.8, 41.1, 35.4, 33.3, 33.1, 28.0, 26.5, 26.2, 26.1. IR (film): ν (cm^{-1}) 3001, 2981, 2917, 2848, 1724, 1630, 1604, 1506, 1483, 1473, 1445, 1393, 1364, 1356, 1349, 1314, 1262, 1223, 1207, 1142, 1123, 1032, 895, 849, 819, 763, 715, 660, 620, 513, 474, 413; HR-MS (ESI) m/z calcd for $C_{24}H_{32}NaO_3^+$ [M+Na$^+$] 391.22437, found 391.22446; $[\alpha]_D^{24.0}$ = -34.3 (c = 0.1, $CHCl_3$); HPLC conditions (er was determined by reducing the product to the corresponding alcohol with $LiAlH_4$): AD-H column, hexane/2-propanol = 90/10, flow rate = 1.0 mL/min, λ = 254 nm, t_R = 10.0 min (major), t_R = 12.6 min (minor), 97:3 er.

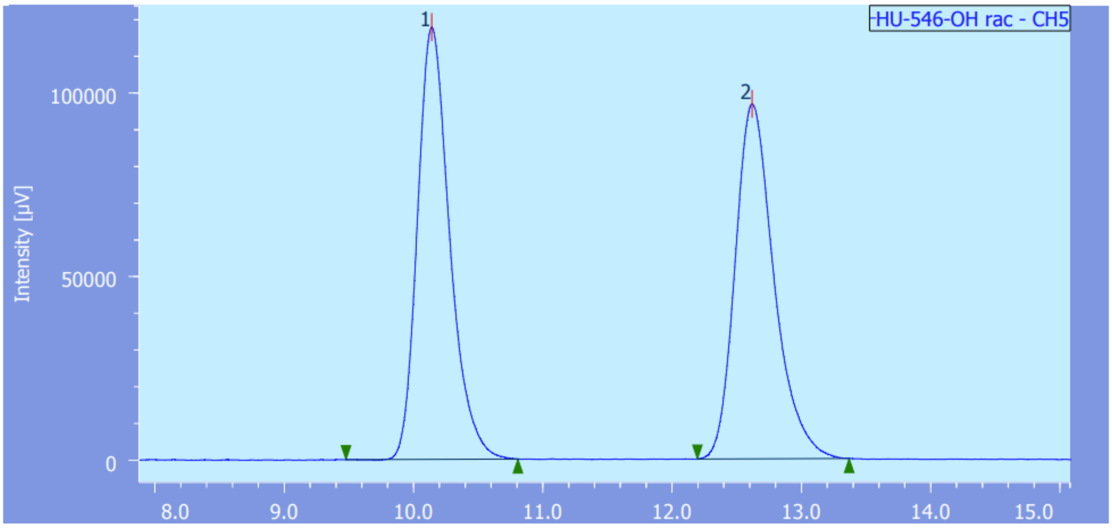

Decision

#	Peak Name	CH	tR [min]	Area [μV·sec]	Height [μV]	Area%	Height%	Quantity	NTP	Resolution	Symmetry Factor	Warning
1	Unknown	5	10.140	2042259	117680	50.247	54.906	N/A	8326	5.083	1.284	
2	Unknown	5	12.617	2022141	96648	49.753	45.094	N/A	8978	N/A	1.308	

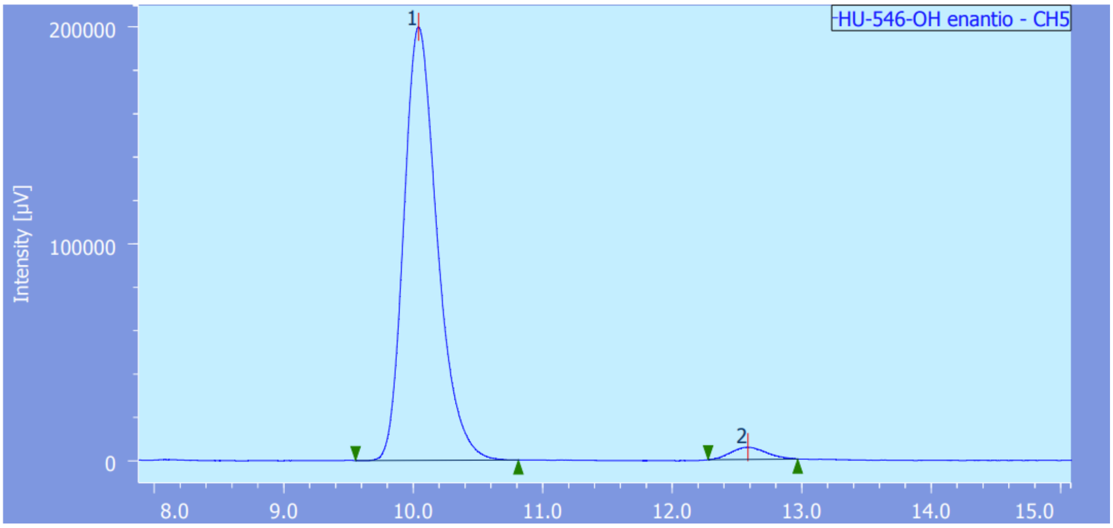

Decision

#	Peak Name	CH	tR [min]	Area [µV·sec]	Height [µV]	Area%	Height%	Quantity	NTP	Resolution	Symmetry Factor	Warning
1	Unknown	5	10.040	3575465	199622	97.069	97.256	N/A	7613	5.224	1.291	
2	Unknown	5	12.583	107945	5632	2.931	2.744	N/A	9512	N/A	1.136	

Methyl (*R*)-4-(1-(*tert*-butoxy)-3-cyclohexyl-1-oxopropan-2-yl)thiophene-2-carboxylate (29)

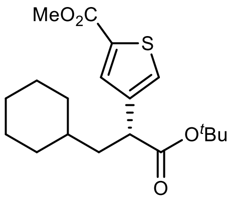

Prepared by **GP-B**. Colorless oil, 45.8 mg, 65% yield. ^{1}H NMR (400 MHz, CDCl$_3$) δ 7.74 (d, *J* = 1.5 Hz, 1H), 7.33 (dd, *J* = 0.4, 1.6 Hz, 1H), 3.86 (s, 3H), 3.66 (t, *J* = 8.0 Hz, 1H), 1.87 (ddd, *J* = 13.6, 8.4, 6.9 Hz, 1H), 1.77 – 1.54 (m, 6H), 1.40 (s, 9H), 1.21 – 1.07 (m, 4H), 0.97 – 0.82 (m, 2H); ^{13}C NMR (101 MHz, CDCl$_3$) δ 172.6, 162.6, 141.2, 133.5, 133.3, 128.5, 80.9, 52.1, 45.4, 41.1, 35.3, 33.0, 27.9, 26.4, 26.11, 26.09. IR (film): ν (cm^{-1}) 2977, 2923, 2849, 1716, 1542, 1442, 1367, 1286, 1251, 1142, 1073, 965, 844, 785, 770, 750, 446, 435; HR-MS (ESI) m/z calcd for $C_{19}H_{28}NaO_4S^+$ [M+Na$^+$] 375.16005, found 375.16041; $[\alpha]_D^{24.1}$ = -8.4 (c = 0.1, CHCl$_3$); HPLC conditions: IC column, hexane/2-propanol = 95/5, flow rate = 1.0 mL/min, λ = 254 nm, t_R = 6.0 min (minor), t_R = 6.5 min (major), 93:7 er.

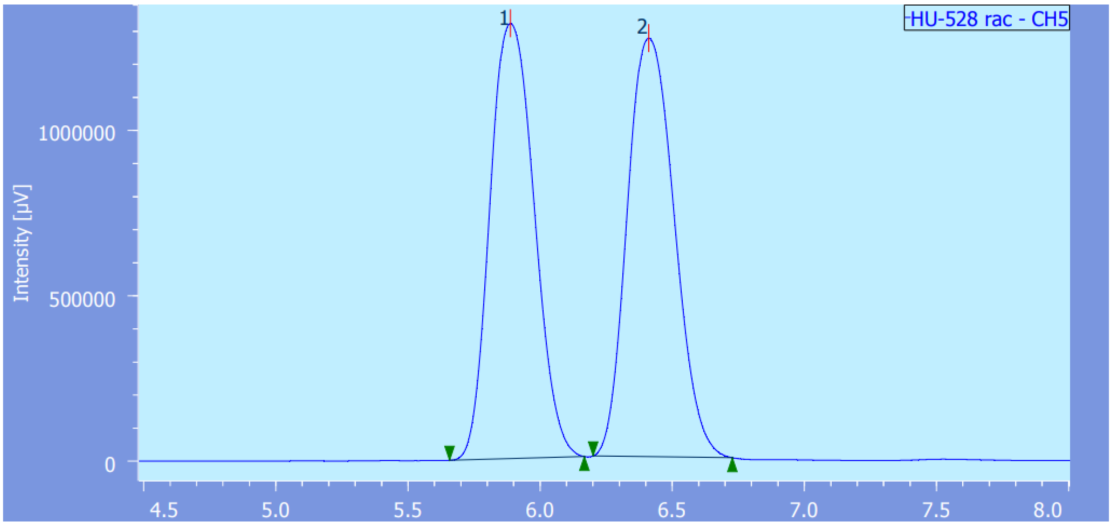

Decision

#	Peak Name	CH	tR [min]	Area [µV·sec]	Height [µV]	Area%	Height%	Quantity	NTP	Resolution	Symmetry Factor	Warning
1	Unknown	5	5.887	15334715	1315386	49.445	50.973	N/A	5550	1.611	1.147	
2	Unknown	5	6.410	15678665	1265193	50.555	49.027	N/A	5842	N/A	1.170	

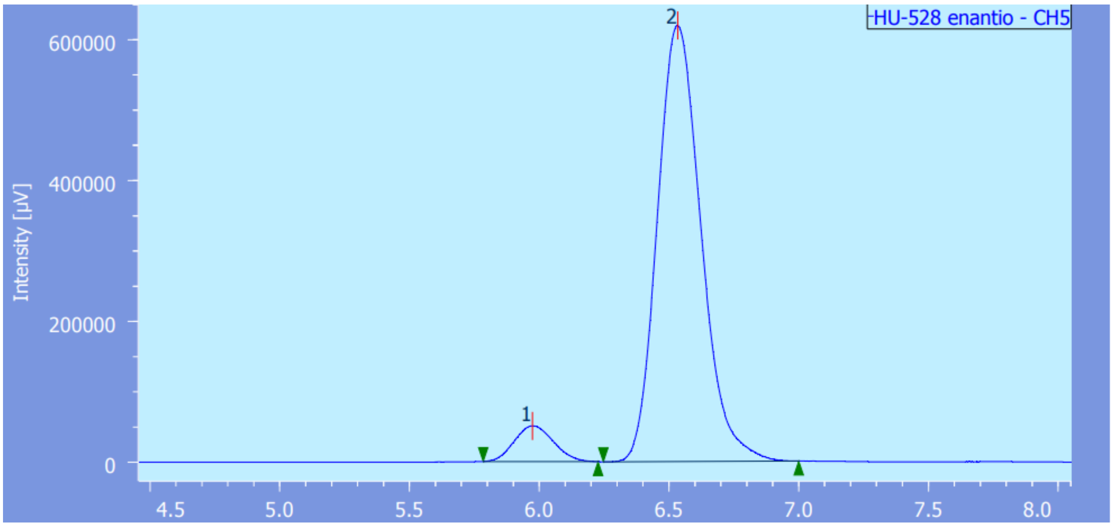

Decision

#	Peak Name	CH	tR [min]	Area [µV·sec]	Height [µV]	Area%	Height%	Quantity	NTP	Resolution	Symmetry Factor	Warning
1	Unknown	5	5.973	533037	50659	6.863	7.565	N/A	7107	1.907	1.124	
2	Unknown	5	6.533	7234146	618960	93.137	92.435	N/A	7322	N/A	1.198	

tert-Butyl (_R_)-2-(benzo[b]thiophen-5-yl)-3-cyclohexylpropanoate (30)

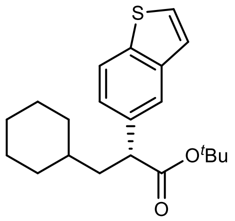

Prepared by **GP-B**. White solid, 44.8 mg, 65% yield. m.p. = 119-120 °C; ^{1}H NMR (400 MHz, $CDCl_3$) δ 7.81 (d, *J* = 8.4 Hz, 1H), 7.76 (d, *J* = 1.6 Hz, 1H), 7.42 (d, *J* = 5.4 Hz, 1H), 7.35 – 7.28 (m, 2H), 3.70 (t, *J* = 7.8 Hz, 1H), 1.99 (ddd, *J* = 13.7, 8.2, 7.1 Hz, 1H), 1.83 – 1.57 (m, 6H), 1.40 (s, 9H), 1.24 – 1.09 (m, 4H), 1.01 – 0.85 (m, 2H); ^{13}C NMR (101 MHz, $CDCl_3$) δ 173.7, 139.8, 138.3, 136.3, 126.5, 124.5, 123.9, 122.7, 122.3, 80.4, 49.7, 41.4, 35.4, 33.3, 33.1, 28.0, 26.5, 26.2, 26.1. IR (film): ν (cm^{-1}) 3101, 2963, 2926, 2848, 1716, 1601, 1446, 1421, 1392, 1365, 1348, 1309, 1274, 1257, 1226, 1212, 1151, 1138, 1124, 1090, 1052, 976, 894, 837, 825, 813, 760, 714, 703, 616, 484; HR-MS (ESI) m/z calcd for $C_{21}H_{28}NaO_2S^+$ [M+Na$^+$] 367.17022, found 367.17078; $[\alpha]_D^{24.1}$ = -21.4 (c = 0.1, $CHCl_3$); HPLC conditions: Chiral-NR column, hexane/2-propanol = 99/1, flow rate = 1.0 mL/min, λ = 230 nm, t_R = 27.4 min (minor), t_R = 32.6 min (major), 96:4 er.

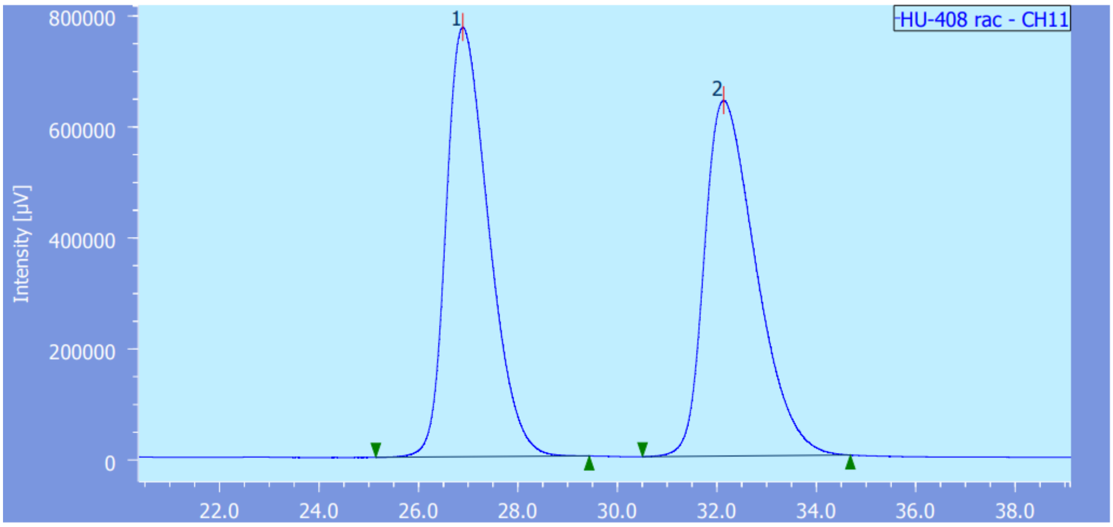

Decision

#	Peak Name	CH	tR [min]	Area [µV·sec]	Height [µV]	Area%	Height%	Quantity	NTP	Resolution	Symmetry Factor	Warning
1	Unknown	11	26.887	46010827	773829	49.858	54.706	N/A	4754	3.048	1.389	
2	Unknown	11	32.133	46273129	640707	50.142	45.294	N/A	4614	N/A	1.497	

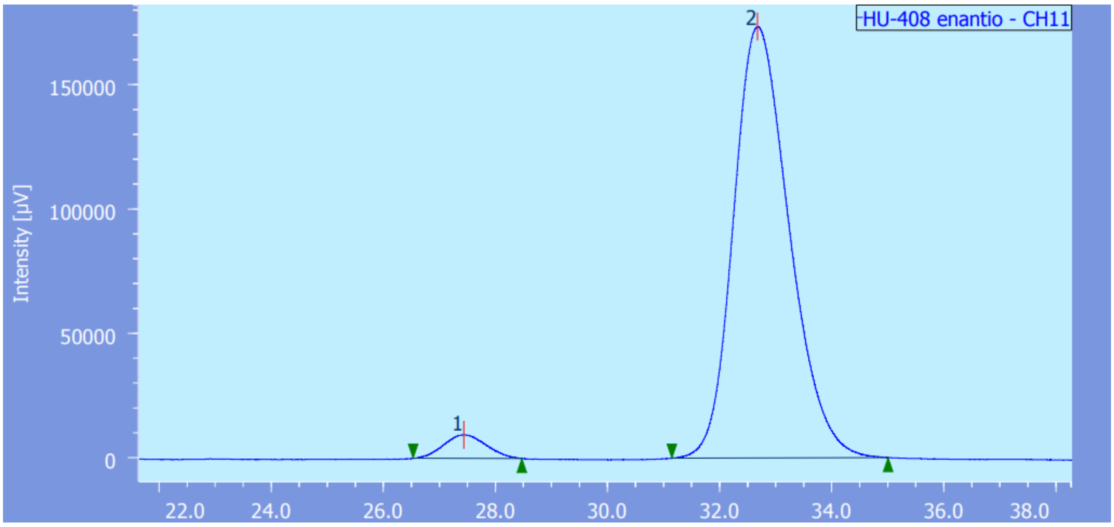

Decision

#	Peak Name	CH	tR [min]	Area [µV·sec]	Height [µV]	Area%	Height%	Quantity	NTP	Resolution	Symmetry Factor	Warning
1	Unknown	11	27.437	502218	9489	4.027	5.190	N/A	5857	3.243	1.071	
2	Unknown	11	32.673	11968824	173348	95.973	94.810	N/A	5245	N/A	1.240	

tert-Butyl (_R_)-2-(benzofuran-5-yl)-3-cyclohexylpropanoate (31)

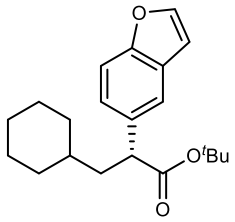

Prepared by **GP-B** (two 40 W 390 nm Kessile lamps were used). White solid, 33.6 mg, 51% yield. m.p. = 89-90 °C; ^{1}H NMR (400 MHz, $CDCl_3$) δ 7.60 (d, J = 2.2 Hz, 1H), 7.53 (d, J = 1.7 Hz, 1H), 7.43 (d, J = 8.5 Hz, 1H), 7.25 (dd, J = 8.6, 1.8 Hz, 1H), 6.74 (dd, J = 2.2, 0.9 Hz, 1H), 3.80 – 3.49 (m, 1H), 1.97 (ddd, J = 13.7, 8.3, 7.1 Hz, 1H), 1.80 – 1.61 (m, 6H), 1.39 (s, 9H), 1.23 – 1.11 (m, 4H), 0.98 – 0.86 (m, 2H); ^{13}C NMR (101 MHz, $CDCl_3$) δ 173.9, 154.1, 145.1, 134.7, 127.5, 124.3, 120.2, 111.1, 106.6, 80.3, 49.7, 41.6, 35.4, 33.3, 33.1, 28.0, 26.5, 26.2, 26.1. IR (film): ν (cm^{-1}) 2981, 2920, 2847, 1721, 1592, 1538, 1466, 1447, 1362, 1345, 1312, 1272, 1257, 1247, 1213, 1190, 1148, 1131, 1109, 1090, 1027, 976, 896, 842, 822, 770, 760, 742, 728, 696, 644, 610, 501, 455, 433; HR-MS (ESI) m/z calcd for $C_{21}H_{28}NaO_3^+$ [M+Na$^+$] 351.19307, found 351.19325; $[\alpha]_D^{22.5}$ = -35.7 (c = 0.1, $CHCl_3$); HPLC conditions: NR column, hexane/2-propanol = 99/1, flow rate = 1.0 mL/min, λ = 240 nm, t_R = 17.4 min (minor), t_R = 19.7 min (major), 95:5 er.

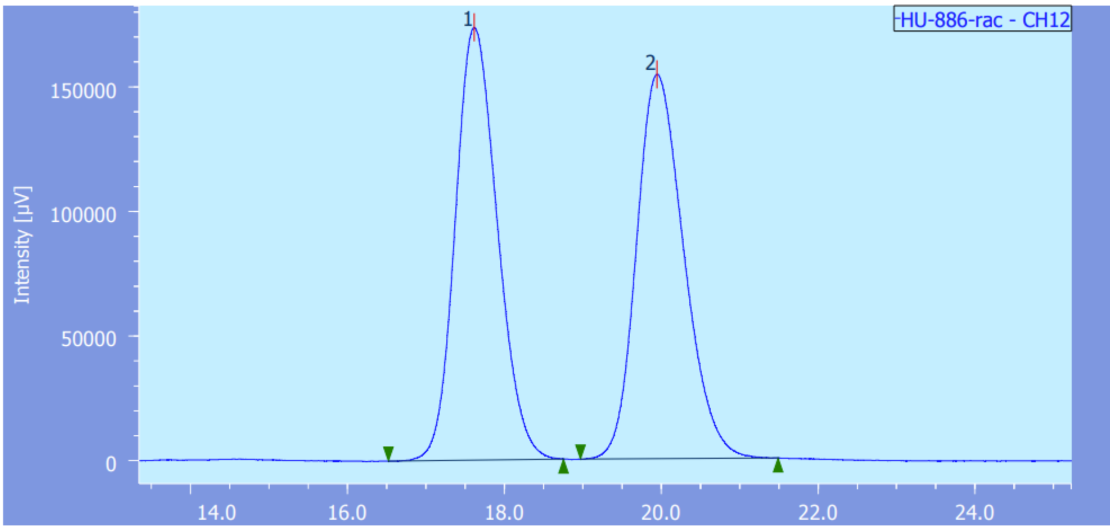

Decision

#	Peak Name	CH	tR [min]	Area [µV·sec]	Height [µV]	Area%	Height%	Quantity	NTP	Resolution	Symmetry Factor	Warning
1	Unknown	12	17.617	6473882	173531	49.986	52.952	N/A	5144	2.239	1.159	
2	Unknown	12	19.943	6477528	154180	50.014	47.048	N/A	5247	N/A	1.229	

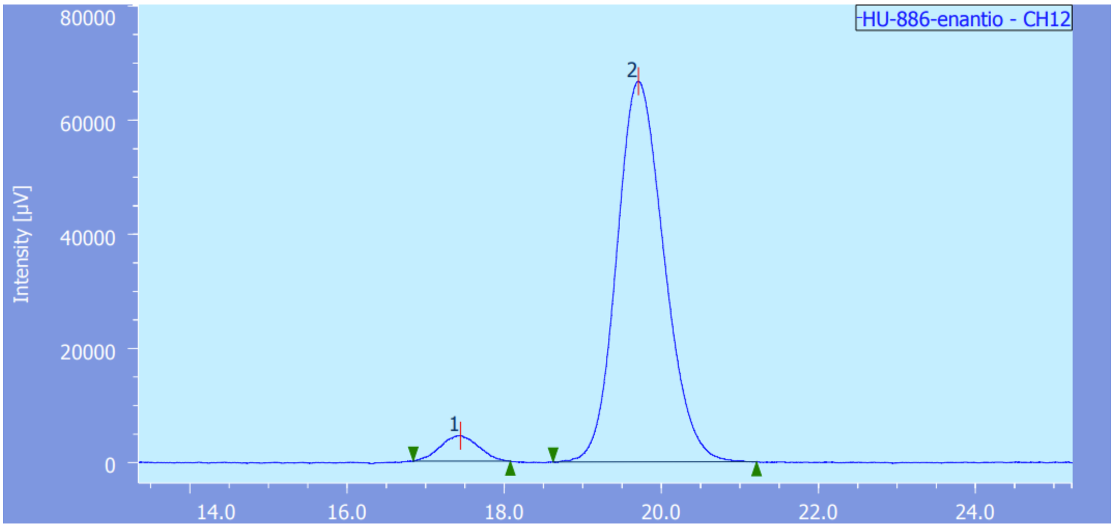

Decision

#	Peak Name	CH	tR [min]	Area [µV·sec]	Height [µV]	Area%	Height%	Quantity	NTP	Resolution	Symmetry Factor	Warning
1	Unknown	12	17.443	151709	4481	5.151	6.300	N/A	5532	2.229	0.996	
2	Unknown	12	19.707	2793717	66655	94.849	93.700	N/A	5153	N/A	1.151	

***tert*-Butyl (*R*)-3-cyclohexyl-2-(dibenzo[b,d]thiophen-2-yl)propanoate (32)**

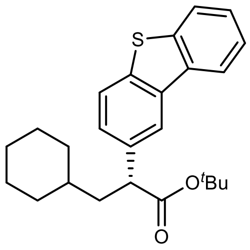

Prepared by **GP-B**. White solid, 52.5 mg, 65% yield. m.p. = 63-64 °C; ^{1}H NMR (400 MHz, $CDCl_3$) δ 8.22 – 8.15 (m, 1H), 8.10 (d, J = 1.5 Hz, 1H), 7.88 – 7.82 (m, 1H), 7.79 (d, J = 8.3 Hz, 1H), 7.49 – 7.41 (m, 3H), 3.77 (t, J = 7.8 Hz, 1H), 2.10 – 1.99 (m, 1H), 1.85 – 1.58 (m, 6H), 1.41 (s, 9H), 1.30 – 1.10 (m, 4H), 1.05 – 0.89 (m, 2H); ^{13}C NMR (101 MHz, $CDCl_3$) δ 173.6, 139.8, 137.9, 136.6, 135.7, 135.4, 126.8, 126.6, 124.3, 122.8, 122.7, 121.6, 120.8, 80.6, 49.9, 41.5, 35.4, 33.3, 33.1, 28.0, 26.5, 26.2, 26.1. IR (film): ν (cm^{-1}) 2974, 2923, 2847, 1720, 1468, 1446, 1432, 1365, 1347, 1316, 1255, 1221, 1140, 1080, 1024, 972, 883, 839, 766, 754, 734, 620, 512, 420; HR-MS (ESI) m/z calcd for $C_{25}H_{30}NaO_2S^+$ [M+Na$^+$] 417.18587, found 417.18596; $[\alpha]_D^{24.2}$ = -16.8 (c = 0.1, $CHCl_3$); HPLC conditions (er was determined by reducing the product to the corresponding alcohol with $LiAlH_4$): AD-H column, hexane/2-propanol = 90/10, flow rate = 1.0 mL/min, λ = 254 nm, t_R = 10.8 min (major), t_R = 13.5 min (minor), 95:5 er.

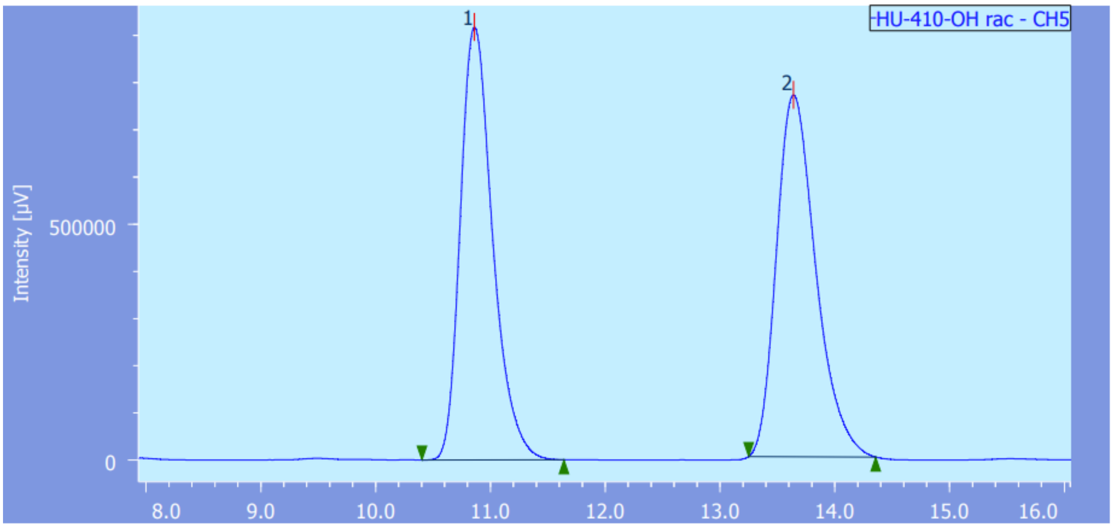

Decision

#	Peak Name	CH	tR [min]	Area [μV·sec]	Height [μV]	Area%	Height%	Quantity	NTP	Resolution	Symmetry Factor	Warning
1	Unknown	5	10.860	17952653	916714	49.544	54.463	N/A	7450	4.964	1.319	
2	Unknown	5	13.637	18282854	766485	50.456	45.537	N/A	7781	N/A	1.326	

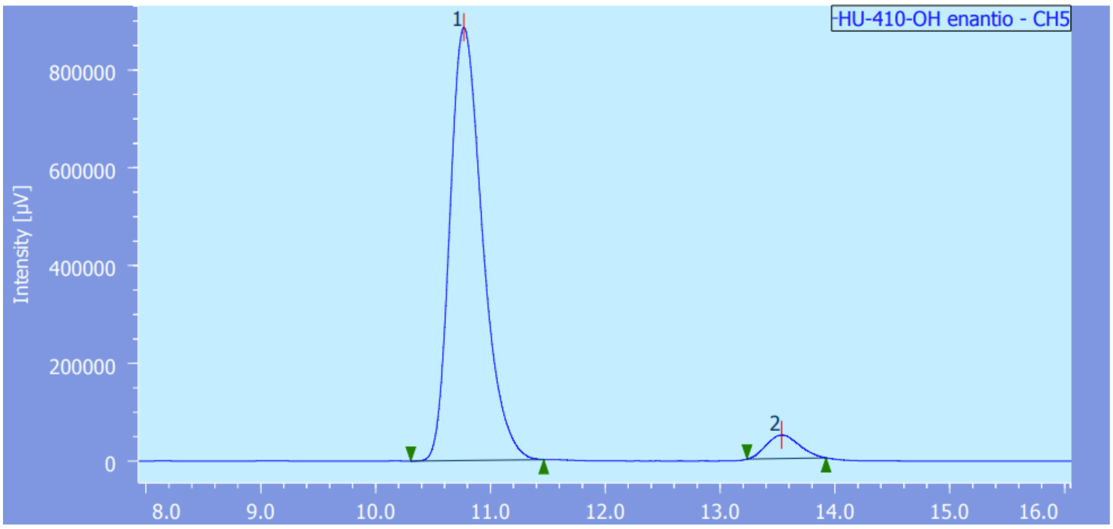

Decision

#	Peak Name	CH	tR [min]	Area [μV·sec]	Height [μV]	Area%	Height%	Quantity	NTP	Resolution	Symmetry Factor	Warning
1	Unknown	5	10.767	17463719	885306	94.785	94.842	N/A	7115	5.259	1.336	
2	Unknown	5	13.533	960881	48151	5.215	5.158	N/A	9887	N/A	1.140	

***tert*-Butyl (*R*)-3-cyclohexyl-2-(dibenzo[b,d]furan-2-yl)propanoate (33)**

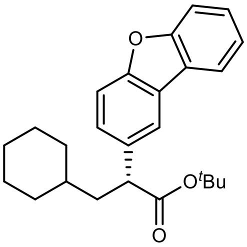

Prepared by **GP-B** (two 40 W 390 nm Kessile lamps were used). White solid, 40.1 mg, 53% yield. m.p. = 102-103 ºC; ^{1}H NMR (400 MHz, $CDCl_3$) ^{1}H NMR (400 MHz, $CDCl_3$) δ 7.96 (ddd, *J* = 7.7, 1.3, 0.6 Hz, 1H), 7.90 (d, *J* = 1.8 Hz, 1H), 7.58 – 7.53 (m, 1H), 7.50 (d, *J* = 8.5 Hz, 1H), 7.47 –7.42 (m, 1H), 7.41 (dd, *J* = 8.5, 1.9 Hz, 1H), 7.37 – 7.31 (m, 1H), 3.74 (t, *J* = 7.8 Hz, 1H), 2.03 (ddd, *J* = 13.7, 8.3, 7.1 Hz, 1H), 1.85 – 1.58 (m, 6H), 1.40 (s, 9H), 1.25 – 1.10 (m, 4H), 1.01 – 0.90 (m, 2H); ^{13}C NMR (101 MHz, $CDCl_3$) δ 173.8, 156.5, 155.3, 134.7, 127.14, 127.07, 124.3, 124.2, 122.6, 120.7, 119.8, 111.6, 111.4, 80.5, 49.8, 41.6, 35.4, 33.3, 33.1, 28.0, 26.5, 26.18, 26.15. IR (film): ν (cm^{-1}) 2934, 2916, 2905, 2846, 2360, 1720, 1477, 1447, 1392, 1335, 1310, 1270, 1262, 1246, 1221, 1198, 1166, 1149, 1137, 1119, 1104, 1092, 1023, 972, 905, 884, 839, 826, 768, 753, 735, 620, 564, 461, 420; HR-MS (ESI) m/z calcd for $C_{25}H_{30}NaO_3^+$ [M+Na$^+$] 401.20872, found 401.20875; $[\alpha]_D^{22.6}$ = -28.7 (c = 0.1, $CHCl_3$); HPLC conditions: AD-H column (er was determined by reducing the product to the corresponding alcohol with $LiAlH_4$), hexane/2-propanol = 90/10, flow rate = 1.0 mL/min, λ = 220 nm, t_R = 9.9 min (major), t_R = 11.1 min (minor), 93:7 er.

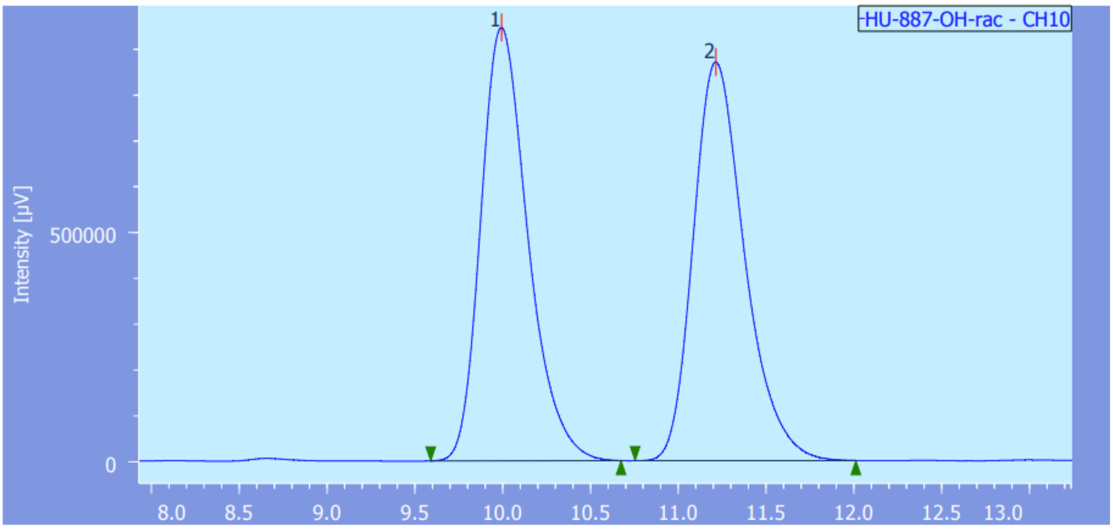

Decision

#	Peak Name	CH	tR [min]	Area [µV·sec]	Height [µV]	Area%	Height%	Quantity	NTP	Resolution	Symmetry Factor	Warning
1	Unknown	10	9.993	17626211	944998	49.863	52.065	N/A	6898	2.434	1.292	
2	Unknown	10	11.213	17722721	870031	50.137	47.935	N/A	7335	N/A	1.292	

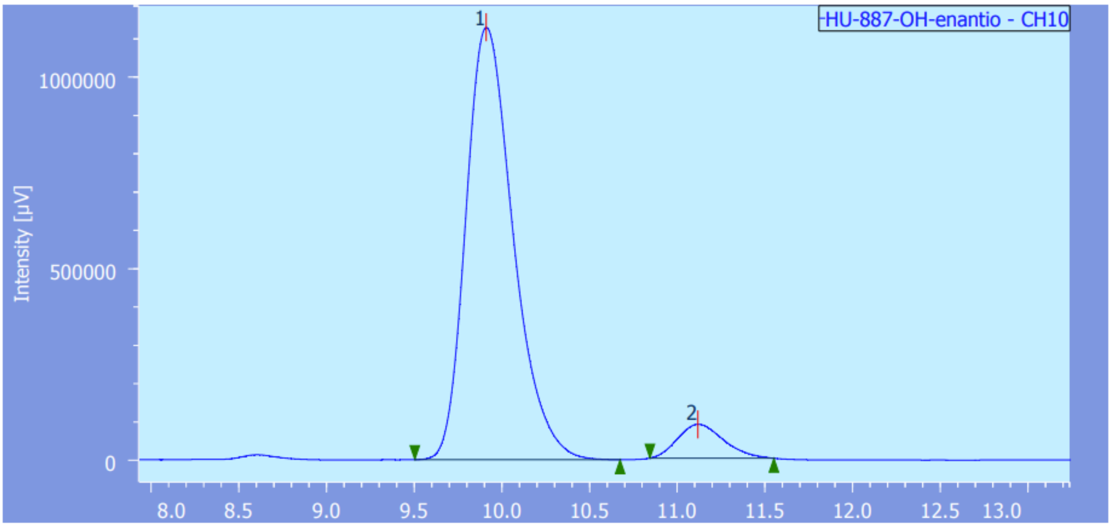

Decision

#	Peak Name	CH	tR [min]	Area [µV·sec]	Height [µV]	Area%	Height%	Quantity	NTP	Resolution	Symmetry Factor	Warning
1	Unknown	10	9.910	21159125	1127637	92.886	92.723	N/A	6664	2.491	1.313	
2	Unknown	10	11.117	1620496	88494	7.114	7.277	N/A	8384	N/A	1.249	

***tert*-Butyl (*R*)-2-(benzo[d]thiazol-5-yl)-3-cyclohexylpropanoate (34)**

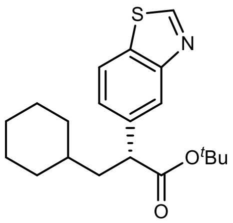

Prepared by **GP-B**. White solid, 50.0 mg, 72% yield. m.p. = 82-83 °C; ^{1}H NMR (400 MHz, $CDCl_3$) δ 8.98 (s, 1H), 8.07 (d, *J* = 1.6 Hz, 1H), 7.89 (d, *J* = 8.3 Hz, 1H), 7.43 (dd, *J* = 8.3, 1.7 Hz, 1H), 3.75 (t, *J* = 7.8 Hz, 1H), 2.05 – 1.93 (m, 1H), 1.80 – 1.57 (m, 6H), 1.39 (s, 9H), 1.21 – 1.07 (m, 4H), 1.00 – 0.86 (m, 2H); ^{13}C NMR (101 MHz, $CDCl_3$) δ 173.3, 154.2, 153.4, 138.7, 132.1, 125.7, 122.9, 121.7, 80.7, 49.8, 41.3, 35.3, 33.3, 33.0, 28.0, 26.5, 26.14, 26.10. IR (film): ν (cm^{-1}) 3069, 2966, 2924, 2903, 2849, 1715, 1547, 1476, 1441, 1392, 1366, 1347, 1313, 1255, 1225, 1147, 1131, 1063, 936, 886, 859, 837, 828, 819, 674, 632, 510, 426; HR-MS (ESI) m/z calcd for $C_{20}H_{28}NO_2S^+$ [M+H$^+$] 346.18353, found 346.18341; $[\alpha]_D^{24.1}$ = -19.9 (c = 0.1, $CHCl_3$); HPLC conditions: Chiral-NR column, hexane/2-propanol = 99/1, flow rate = 1.0 mL/min, λ = 230 nm, t_R = 23.9 min (minor), t_R = 37.1 min (major), 95:5 er.

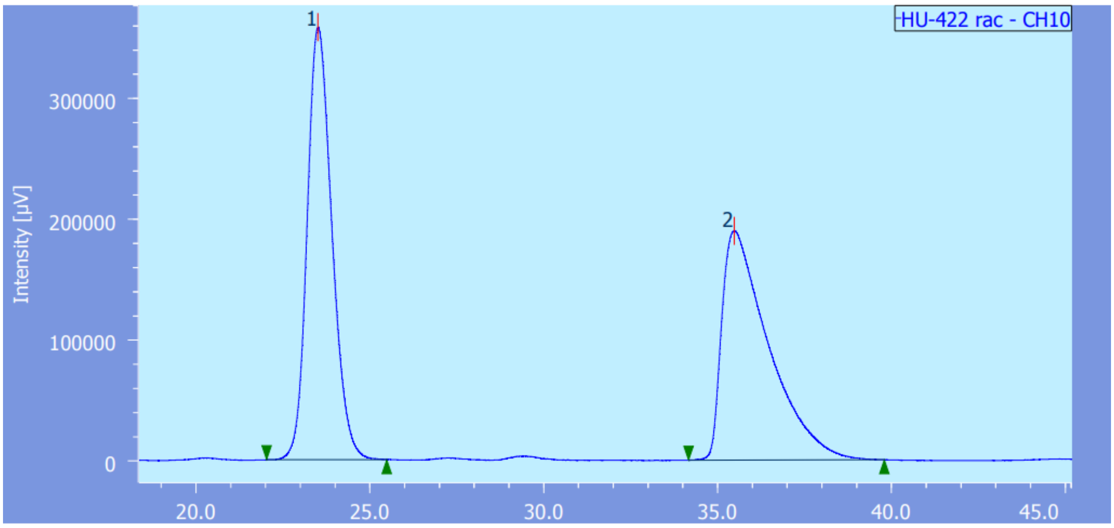

Decision

#	Peak Name	CH	tR [min]	Area [µV·sec]	Height [µV]	Area%	Height%	Quantity	NTP	Resolution	Symmetry Factor	Warning
1	Unknown	10	23.507	17710065	357598	49.956	65.359	N/A	5331	6.552	1.201	
2	Unknown	10	35.470	17741246	189528	50.044	34.641	N/A	3572	N/A	2.511	

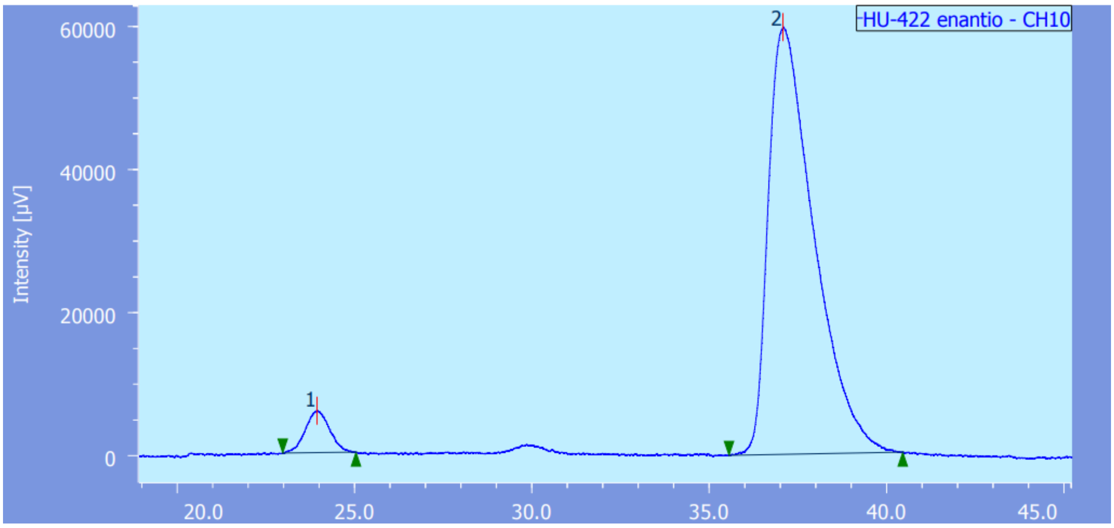

Decision

#	Peak Name	CH	tR [min]	Area [µV·sec]	Height [µV]	Area%	Height%	Quantity	NTP	Resolution	Symmetry Factor	Warning
1	Unknown	10	23.937	279491	5878	4.969	8.970	N/A	6063	7.420	1.019	
2	Unknown	10	37.073	5345187	59656	95.031	91.030	N/A	4083	N/A	1.868	

***tert*-Butyl (*R*)-2-(2-chloropyridin-4-yl)-3-cyclohexylpropanoate (35)**

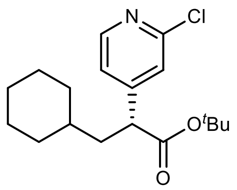

Prepared by **GP-B**. Colorless oil, 40.8 mg, 63% yield. ^{1}H NMR (400 MHz, $CDCl_3$) δ 8.24 (d, *J* = 5.1 Hz, 1H), 7.21 (s, 1H), 7.10 (dd, *J* = 5.2, 1.5 Hz, 1H), 3.48 (dd, *J* = 8.3, 7.3 Hz, 1H), 1.85 (ddd, *J* = 13.8, 8.5, 7.0 Hz, 1H), 1.69 – 1.44 (m, 6H), 1.34 (s, 9H), 1.15 – 1.02 (m, 4H), 0.91 – 0.75 (m, 2H); ^{13}C NMR (101 MHz, $CDCl_3$) δ 171.5, 152.2, 151.7, 149.6, 123.7, 121.9, 81.6, 49.2, 40.7, 35.4, 33.01, 32.97, 27.8, 26.3, 26.01, 26.00. IR (film): ν (cm^{-1}) 2976, 2924, 2851, 2360, 2343, 1727, 1590, 1547, 1464, 1449, 1390, 1367, 1252, 1224, 1146, 1088, 990, 885, 840, 823, 754, 573, 502, 455, 418; HR-MS (ESI) m/z calcd for $C_{18}H_{27}ClNO_2^+$ [M+H$^+$] 324.17248, found 324.17283; $[\alpha]_D^{24.2}$ = -9.8 (c = 0.1, $CHCl_3$); HPLC conditions: IC column, hexane/2-propanol = 95/5, flow rate = 1.0 mL/min, λ = 276 nm, t_R = 15.2 min (minor), t_R = 25.0 min (major), 96:4 er.

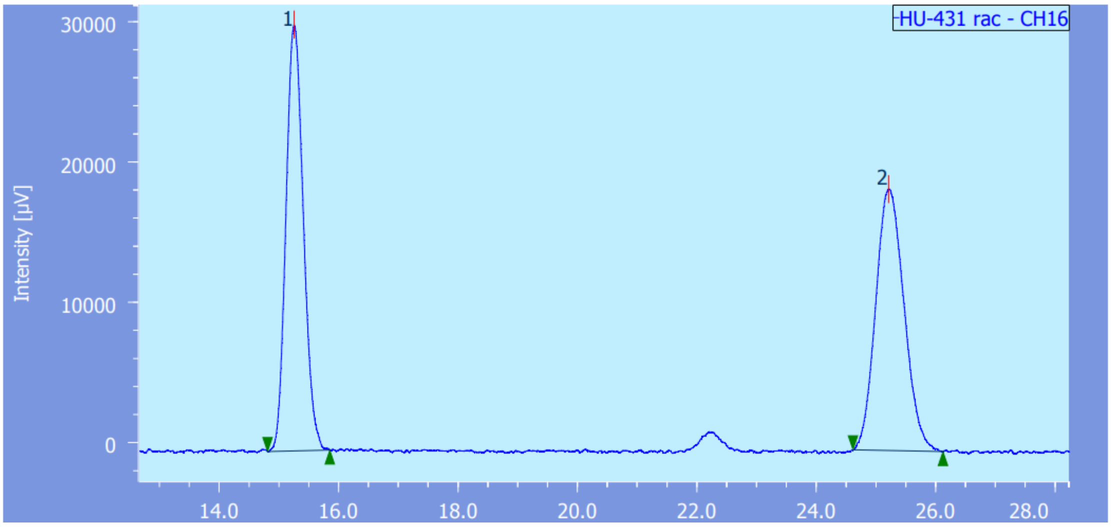

Decision

#	Peak Name	CH	tR [min]	Area [µV·sec]	Height [µV]	Area%	Height%	Quantity	NTP	Resolution	Symmetry Factor	Warning
1	Unknown	16	15.257	615008	30354	49.997	61.982	N/A	12908	14.068	1.143	
2	Unknown	16	25.210	615075	18619	50.003	38.018	N/A	13082	N/A	1.132	

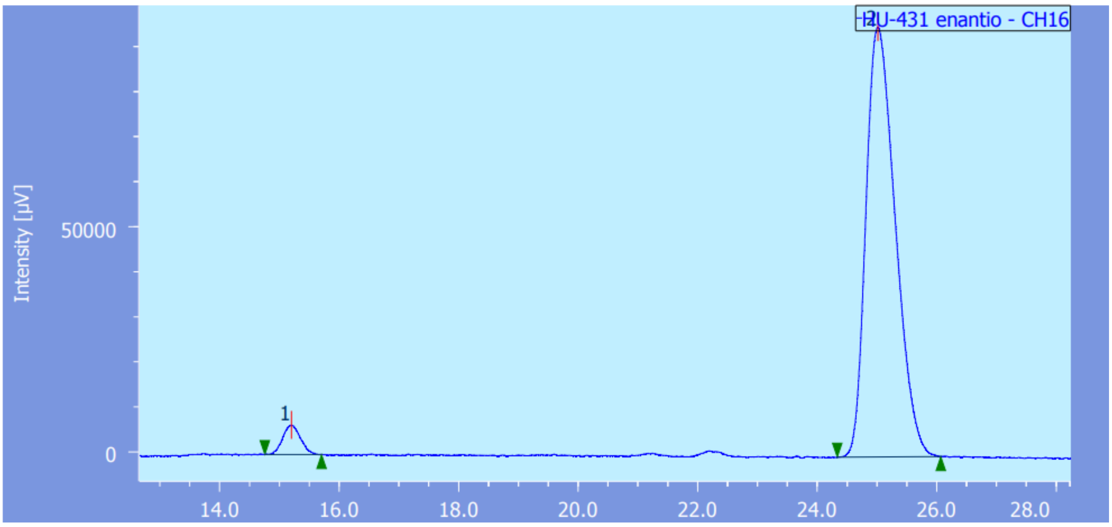

Decision

#	Peak Name	CH	tR [min]	Area [µV·sec]	Height [µV]	Area%	Height%	Quantity	NTP	Resolution	Symmetry Factor	Warning
1	Unknown	16	15.200	135375	6566	4.002	6.444	N/A	12331	13.484	1.093	
2	Unknown	16	25.013	3247556	95331	95.998	93.556	N/A	12038	N/A	1.338	

***tert*-Butyl (*R*)-3-cyclohexyl-2-(2-methylpyrimidin-5-yl)propanoate (36)**

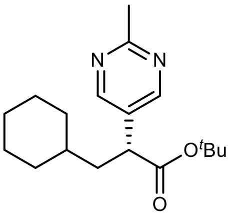

Prepared by **GP-A**. White solid, 43.8 mg, 72% yield. m.p. = 66-67 °C; ^{1}H NMR (400 MHz, CDCl$_3$) δ 8.56 (s, 2H), 3.51 (t, *J* = 7.9 Hz, 1H), 2.71 (s, 3H), 1.93 (dt, *J* = 13.8, 7.7 Hz, 1H), 1.76 – 1.53 (m, 6H), 1.39 (s, 9H), 1.18 – 1.07 (m, 4H), 0.97 – 0.83 (m, 2H); ^{13}C NMR (101 MHz, CDCl$_3$) δ 172.1, 166.8, 156.4, 129.9, 81.6, 45.2, 40.7, 35.2, 33.1, 32.8, 27.9, 26.3, 26.0, 26.0, 25.6. IR (film): ν (cm^{-1}) 2980, 2919, 2849, 1718, 1589, 1555, 1445, 1412, 1395, 1368, 1347, 1328, 1272, 1248, 1227, 1147, 1125, 1048, 970, 902, 896, 875, 840, 754, 723, 652, 515; HR-MS (ESI) m/z calcd for $C_{18}H_{29}N_2O_2^+$ [M+H$^+$] 305.22235, found 305.22188; $[\alpha]_D^{24.2}$ = -6.9 (c = 0.1, CHCl$_3$); HPLC conditions: Chiral-NR column, hexane/2-propanol = 95/5, flow rate = 1.0 mL/min, λ = 230 nm, t_R = 13.2 min (major), t_R = 18.2 min (minor), 95:5 er.

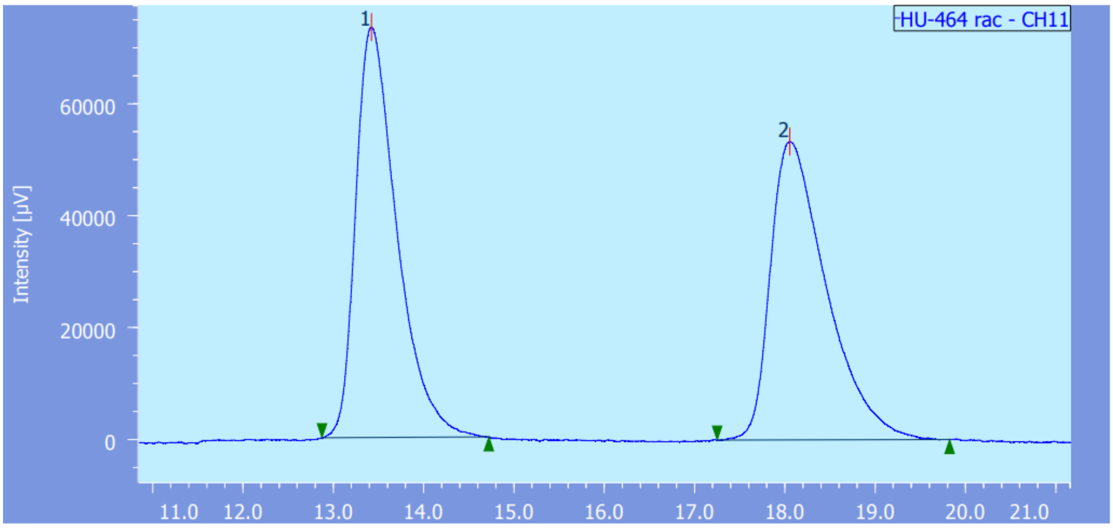

Decision

#	Peak Name	CH	tR [min]	Area [µV·sec]	Height [µV]	Area%	Height%	Quantity	NTP	Resolution	Symmetry Factor	Warning
1	Unknown	11	13.420	2327138	73307	50.295	57.899	N/A	4425	4.861	1.564	
2	Unknown	11	18.053	2299807	53305	49.705	42.101	N/A	4275	N/A	1.703	

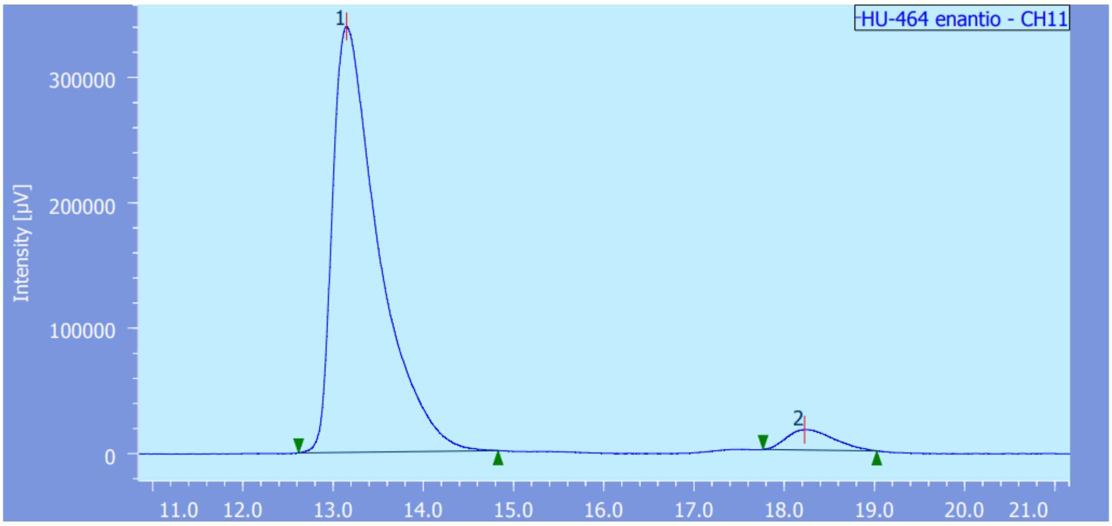

Decision

#	Peak Name	CH	tR [min]	Area [µV·sec]	Height [µV]	Area%	Height%	Quantity	NTP	Resolution	Symmetry Factor	Warning
1	Unknown	11	13.150	12053039	339526	95.290	95.421	N/A	3659	5.449	2.140	
2	Unknown	11	18.223	595766	16292	4.710	4.579	N/A	5341	N/A	1.385	

***tert*-Butyl (*R*)-3-cyclohexyl-2-(quinolin-6-yl)propanoate (37)**

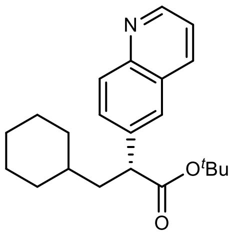

Prepared by **GP-A**. White solid, 44.2 mg, 65% yield. m.p. = 86-87 ºC; ^{1}H NMR (400 MHz, $CDCl_3$) δ 8.87 (dd, *J* = 4.2, 1.7 Hz, 1H), 8.13 (dd, *J* = 8.4, 1.0 Hz, 1H), 8.06 (d, *J* = 8.5 Hz, 1H), 7.75 – 7.67 (m, 2H), 7.38 (dd, *J* = 8.3, 4.2 Hz, 1H), 3.77 (t, *J* = 7.8 Hz, 1H), 2.02 (ddd, *J* = 13.8, 8.1, 7.2 Hz, 1H), 1.82 – 1.55 (m, 6H), 1.38 (s, 9H), 1.23 – 1.08 (m, 4H), 1.00 – 0.86 (m, 2H); ^{13}C NMR (101 MHz, $CDCl_3$) δ 173.2, 150.0, 147.4, 138.5, 136.0, 129.9, 129.4, 128.2, 126.3, 121.1, 80.8, 49.8, 41.1, 35.4, 33.2, 33.0, 27.9, 26.4, 26.11, 26.09. IR (film): ν (cm^{-1}) 2966, 2930, 2913, 2846, 1717, 1498, 1446, 1391, 1365, 1256, 1217, 1141, 1126, 1117, 895, 850, 838, 803, 777, 612, 481; HR-MS (ESI) m/z calcd for $C_{22}H_{30}NO_2^+$ [M+H$^+$] 340.22711, found 340.22715; $[\alpha]_D^{24.2}$ = -25.5 (c = 0.1, $CHCl_3$); HPLC conditions: Chiral-NR column, hexane/2-propanol = 90/10, flow rate = 1.0 mL/min, λ = 230 nm, t_R = 20.0 min (major),

t_R = 22.8 min (minor), 96:4 er.

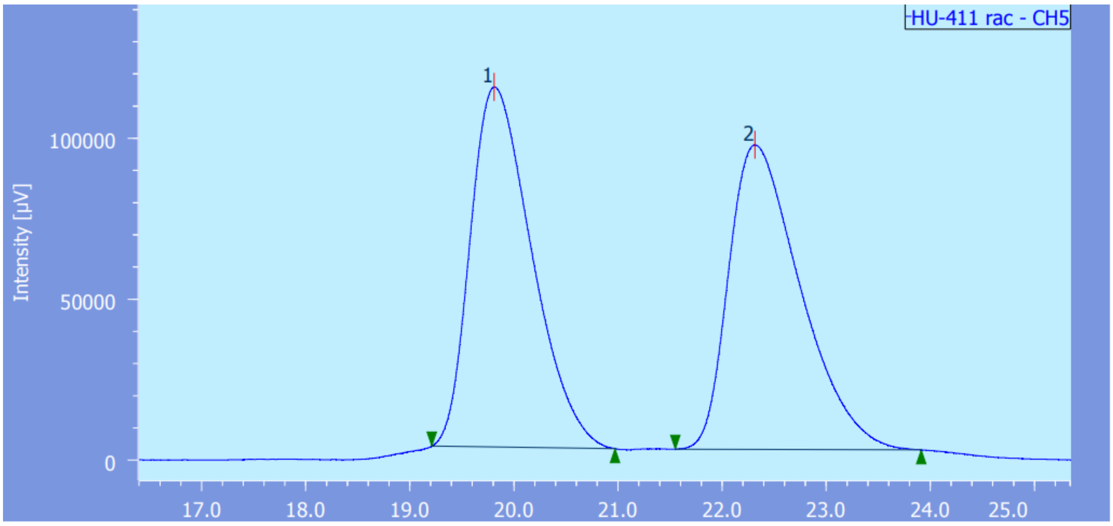

Decision

#	Peak Name	CH	tR [min]	Area [µV·sec]	Height [µV]	Area%	Height%	Quantity	NTP	Resolution	Symmetry Factor	Warning
1	Unknown	5	19.810	4595889	111773	50.337	54.161	N/A	5239	2.129	1.416	
2	Unknown	5	22.313	4534372	94599	49.663	45.839	N/A	4990	N/A	1.559	

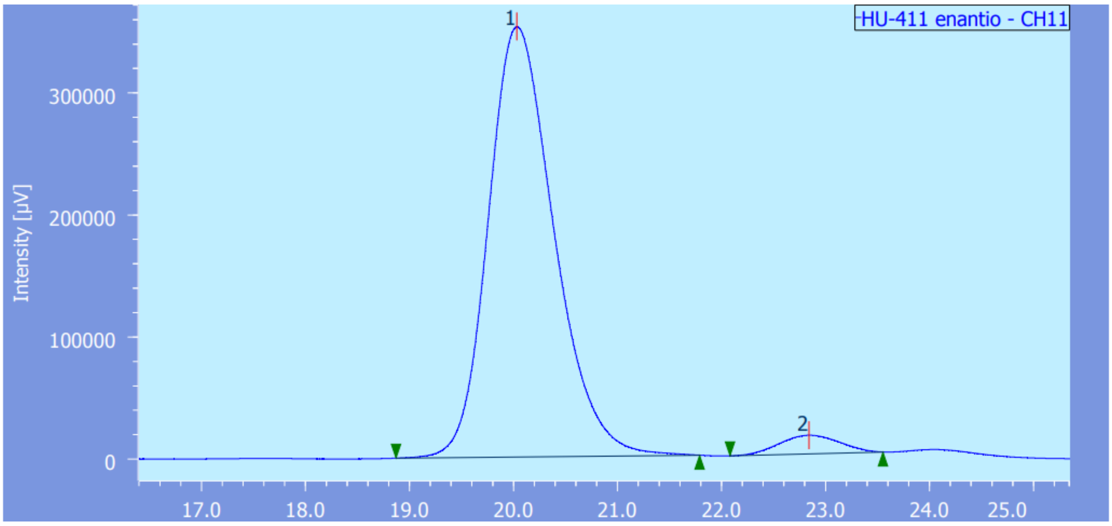

Decision

#	Peak Name	CH	tR [min]	Area [µV·sec]	Height [µV]	Area%	Height%	Quantity	NTP	Resolution	Symmetry Factor	Warning
1	Unknown	11	20.030	15479370	352428	96.151	95.853	N/A	4975	2.495	1.246	
2	Unknown	11	22.840	619646	15249	3.849	4.147	N/A	6627	N/A	1.011	

***tert*-Butyl (*R*)-3-cyclohexyl-2-(quinolin-3-yl)propanoate (38)**

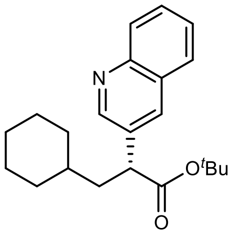

Prepared by **GP-A**. White solid, 42.2 mg, 62% yield. m.p. = 84-85 ºC; ^{1}H NMR (400 MHz, $CDCl_3$) δ 8.87 (d, *J* = 2.2 Hz, 1H), 8.15 – 8.05 (m, 2H), 7.81 (d, *J* = 8.2 Hz, 1H), 7.74 – 7.64 (m, 1H), 7.58 – 7.48 (m, 1H), 3.79 (t, *J* = 7.8 Hz, 1H), 2.05 (dt, *J* = 13.9, 7.7 Hz, 1H), 1.82 – 1.56 (m, 6H), 1.40 (s, 9H), 1.26 – 1.09 (m, 4H), 1.01 – 0.88 (m, 2H); ^{13}C NMR (101 MHz, $CDCl_3$) δ 172.8, 151.1, 147.2, 134.3, 132.9, 129.2, 129.0, 128.0, 127.7, 126.7, 81.2, 47.6, 41.0, 35.4, 33.2, 33.0, 27.9, 26.4, 26.1, 26.0. IR (film): ν (cm^{-1}) 2976, 2931, 2913, 2844, 1721, 1569, 1493, 1447, 1392, 1367, 1344, 1317, 1261, 1214, 1147, 1130, 959, 905, 855, 843, 790, 754, 615, 483, 412; HR-MS (ESI) m/z calcd for $C_{22}H_{30}NO_2^+$ [M+H$^+$] 340.22711, found 340.22705; $[\alpha]_D^{24.2}$ = -13.4 (c = 0.1, $CHCl_3$); HPLC conditions: Chiral-NR column, hexane/2-

propanol = 90/10, flow rate = 1.0 mL/min, λ = 230 nm, t_R = 14.8 min (major), t_R = 16.8 min (minor), 95:5 er.

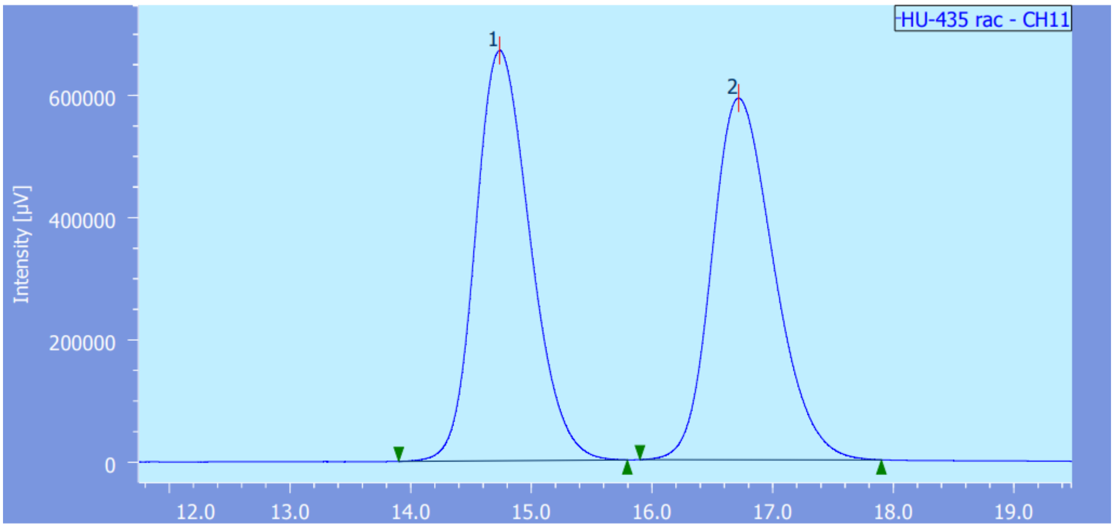

Decision

#	Peak Name	CH	tR [min]	Area [μV·sec]	Height [μV]	Area%	Height%	Quantity	NTP	Resolution	Symmetry Factor	Warning
1	Unknown	11	14.737	21135417	670649	49.958	53.142	N/A	5083	2.250	1.203	
2	Unknown	11	16.717	21170729	591347	50.042	46.858	N/A	5081	N/A	1.240	

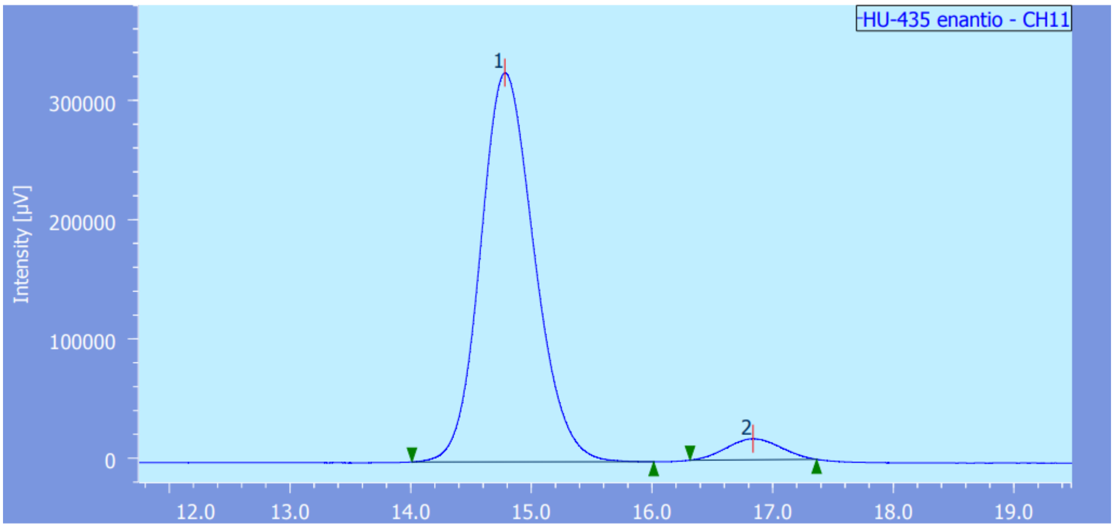

Decision

#	Peak Name	CH	tR [min]	Area [μV·sec]	Height [μV]	Area%	Height%	Quantity	NTP	Resolution	Symmetry Factor	Warning
1	Unknown	11	14.780	10116198	325982	94.830	94.876	N/A	5347	2.465	1.154	
2	Unknown	11	16.837	551570	17606	5.170	5.124	N/A	6064	N/A	1.016	

***tert*-Butyl (*R*)-3-cyclohexyl-2-(1H-inden-2-yl)propanoate (39)**

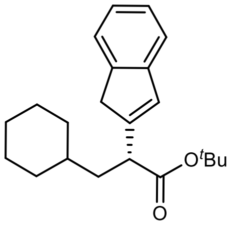

Prepared by **GP-B**. White solid, 37.9 mg, 58% yield. m.p. = 66-67 ºC; ^{1}H NMR (400 MHz, $CDCl_3$) δ 7.42 (d, *J* = 7.4 Hz, 1H), 7.32 (d, *J* = 7.4 Hz, 1H), 7.27 – 7.22 (m, 1H), 7.15 (td, *J* = 7.4, 1.2 Hz, 1H), 6.68 (s, 1H), 3.59 (t, *J* = 7.7 Hz, 1H), 3.50 – 3.35 (m, 2H), 1.85 (ddd, *J* = 13.7, 8.0, 7.2 Hz, 1H), 1.80 – 1.57 (m, 6H), 1.45 (s, 9H), 1.31 – 1.14 (m, 4H), 1.01 – 0.86 (m, 2H); ^{13}C NMR (101 MHz, $CDCl_3$) δ 173.0, 147.2, 144.8, 143.2, 128.4, 126.2, 124.1, 123.5, 120.5, 80.6, 46.3, 39.7, 39.4, 35.4, 33.2, 33.1, 28.0, 26.5, 26.19, 26.15. IR (film): ν (cm^{-1}) 2978, 2920, 2846, 1720, 1607, 1461, 1446, 1388, 1364, 1345, 1308, 1268, 1257, 1245, 1212, 1148, 1137, 1121, 1088, 971, 879, 838, 749, 717, 559, 505, 416; HR-MS (ESI) m/z calcd for

$C_{22}H_{30}NaO_2^+$ [M+Na$^+$] 349.21380, found 349.21378; $[\alpha]_D^{24.2}$ = 17.3 (c = 0.1, $CHCl_3$); HPLC conditions (er was determined by reducing the product to the corresponding alcohol with $LiAlH_4$): AD-H column, hexane/2-propanol = 90/10, flow rate = 1.0 mL/min, λ = 254 nm, t_R = 6.6 min (major), t_R = 7.8 min (minor), 93:7 er.

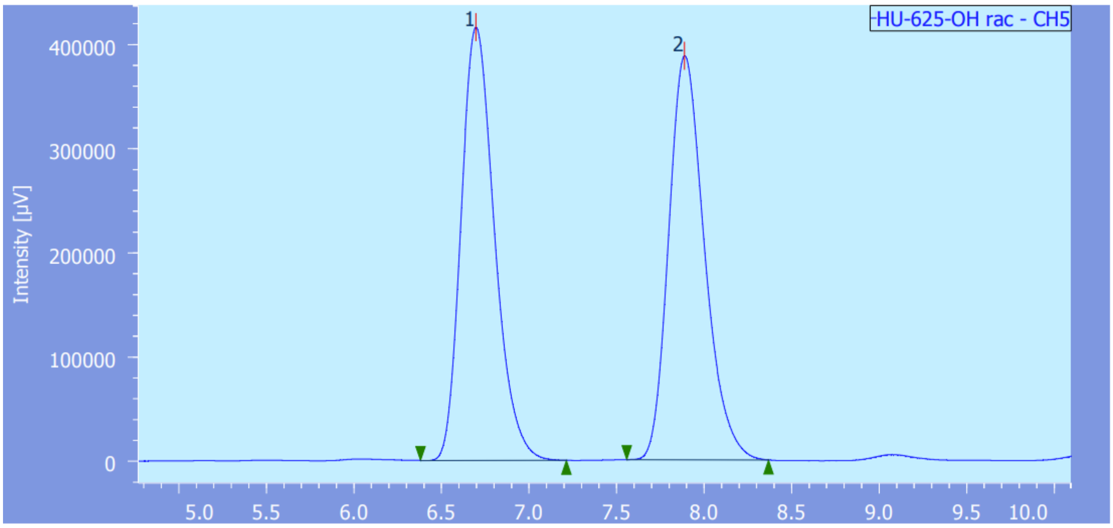

Decision

#	Peak Name	CH	tR [min]	Area [µV·sec]	Height [µV]	Area%	Height%	Quantity	NTP	Resolution	Symmetry Factor	Warning
1	Unknown	5	6.697	5524149	415555	50.113	51.735	N/A	5958	3.339	1.255	
2	Unknown	5	7.887	5499255	387686	49.887	48.265	N/A	7360	N/A	1.268	

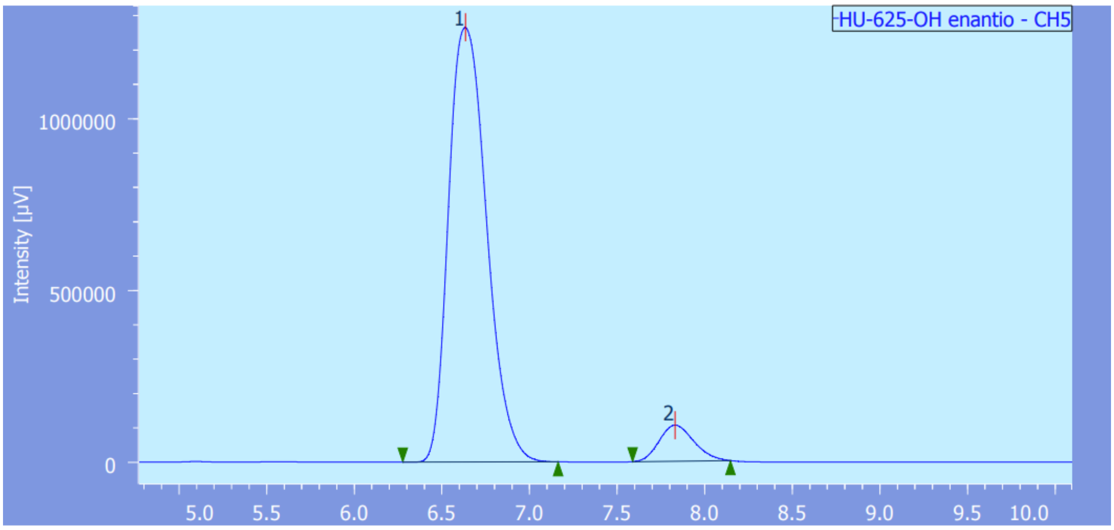

Decision

#	Peak Name	CH	tR [min]	Area [µV·sec]	Height [µV]	Area%	Height%	Quantity	NTP	Resolution	Symmetry Factor	Warning
1	Unknown	5	6.633	19197810	1264562	92.982	92.330	N/A	4308	3.115	1.272	
2	Unknown	5	7.830	1449038	105043	7.018	7.670	N/A	7317	N/A	1.185	

***tert*-Butyl (*R*, *E*)-2-(cyclohexylmethyl)-4-phenylbut-3-enoate (40)**

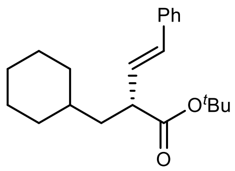

Prepared by **GP-B**. White solid, 36.0 mg, 57% yield. m.p. = 62-63 °C; ^{1}H NMR (400 MHz, $CDCl_3$) δ 7.40 – 7.35 (m, 2H), 7.33 – 7.27 (m, 2H), 7.25 – 7.19 (m, 1H), 6.44 (d, *J* = 15.9 Hz, 1H), 6.16 (dd, *J* = 15.9, 8.9 Hz, 1H), 3.17 (dd, *J* = 16.3, 7.9 Hz, 1H), 1.80 – 1.61 (m, 7H), 1.46 (s, 9H), 1.28 – 1.14 (m, 4H), 0.97 – 0.85 (m, 2H); ^{13}C NMR (101 MHz, $CDCl_3$) δ173.7, 137.2, 131.3, 128.8, 128.5, 127.3, 126.3, 80.5, 48.0, 40.4, 35.2, 33.3, 33.0, 28.1, 26.5, 26.22, 26.16. IR (film): ν (cm^{-1}) 2982, 2921, 2903, 2846, 1718, 1476, 1462, 1446, 1388, 1366, 1344, 1256, 1232,

1205, 1146, 1125, 982, 974, 887, 853, 843, 774, 743, 702, 691, 506, 484; HR-MS (ESI) m/z calcd for $C_{21}H_{30}NaO_2^+$ [M+Na$^+$] 337.21380, found 337.21404; $[\alpha]_D^{24.2}$ = -38.7 (c = 0.1, $CHCl_3$); HPLC conditions: IB column, hexane/2-propanol = 99.8/0.2, flow rate = 0.5 mL/min, λ = 254 nm, t_R = 16.3 min (major), t_R = 17.8 min (minor), 85:15 er.

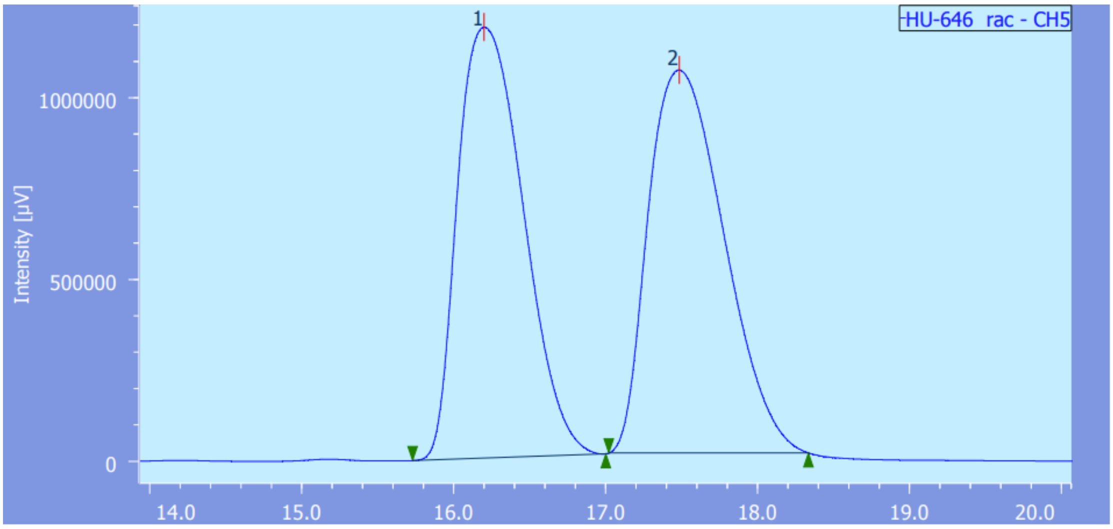

Decision

#	Peak Name	CH	tR [min]	Area [µV·sec]	Height [µV]	Area%	Height%	Quantity	NTP	Resolution	Symmetry Factor	Warning
1	Unknown	5	16.200	35700384	1184868	49.349	52.943	N/A	6122	1.442	1.385	
2	Unknown	5	17.483	36641708	1053157	50.651	47.057	N/A	5352	N/A	1.394	

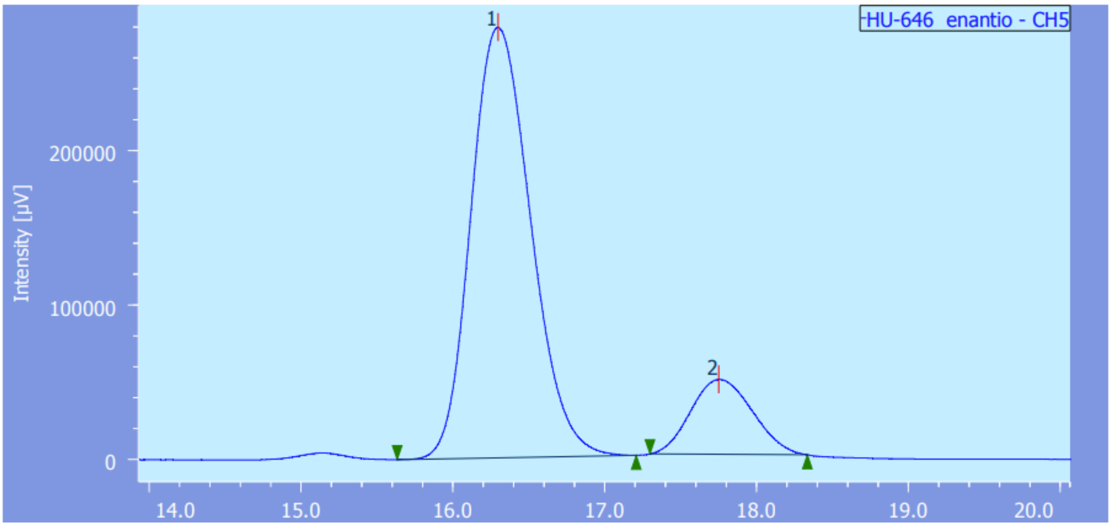

Decision

#	Peak Name	CH	tR [min]	Area [µV·sec]	Height [µV]	Area%	Height%	Quantity	NTP	Resolution	Symmetry Factor	Warning
1	Unknown	5	16.297	7629311	278622	84.780	85.161	N/A	8031	1.944	1.193	
2	Unknown	5	17.750	1369643	48550	15.220	14.839	N/A	8464	N/A	1.129	

(3a*R*, 5*R*, 6*S*, 6a*R*)-5-((*R*)-2, 2-Dimethyl-1,3-dioxolan-4-yl)-2, 2-dimethyltetrahydrofuro[2, 3-d][1,3]dioxol-6-yl 4-((*R*)-1-(*tert*-butoxy)-3-cyclohexyl-1-oxopropan-2-yl)benzoate (41)

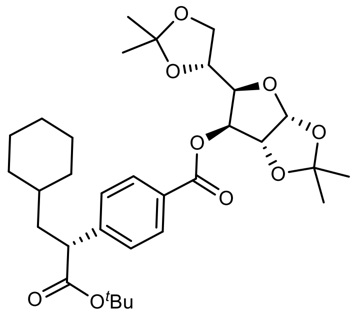

Prepared by **GP-A**. White solid, 83.6 mg, 73% yield. m.p. = 78-79 ºC; ^{1}H NMR (400 MHz, $CDCl_3$) δ 7.99 – 7.93 (m, 2H), 7.41 – 7.35 (m, 2H), 5.93 (d, *J* = 3.7 Hz, 1H), 5.48 (d, *J* = 2.8 Hz, 1H), 4.61 (d, *J* = 3.7 Hz, 1H), 4.40 – 4.30 (m, 2H), 4.15 – 4.05 (m, 2H), 3.64 (t, *J* = 7.8 Hz, 1H), 1.99 – 1.88 (m, 1H), 1.76 – 1.57 (m, 6H), 1.55 (s, 3H), 1.41 (s, 3H), 1.38 (s, 9H), 1.31 (s, 3H), 1.27 (s, 3H), 1.19 – 1.05 (m, 4H), 0.90 (dd, *J* = 21.9, 10.4 Hz, 2H); ^{13}C NMR (101 MHz, $CDCl_3$) δ 172.7, 165.1, 146.0, 129.9, 128.1, 112.3, 109.4, 105.1, 83.4, 80.9, 79.9, 76.6, 72.6, 67.2, 50.0, 40.9, 35.4, 33.2, 33.0, 27.9, 26.8, 26.7, 26.4, 26.2, 26.11, 26.07, 25.2. IR (film): ν (cm^{-1}) 2980, 2924, 2852, 2358, 2341, 1718, 1609, 1449, 1369, 1305, 1268, 1219, 1147, 1095, 1074, 1018, 890, 853, 843, 757, 735, 703, 634, 512; HR-MS (ESI) m/z calcd for $C_{32}H_{46}NaO_9^+$ [M+Na$^+$] 597.30340, found 597.30331; $[\alpha]_D^{24.2}$ = -24.3 (c = 0.1, $CHCl_3$); d.r.>20:1.

(8*R*, 9*S*, 13*S*, 14*S*)-13-Methyl-17-oxo-7, 8, 9, 11, 12, 13, 14, 15, 16, 17-decahydro-6H-cyclopenta[a]phenanthren-3-yl 4-((*R*)-1-(*tert*-butoxy)-3-cyclohexyl-1-oxopropan-2-yl)benzoate (42)

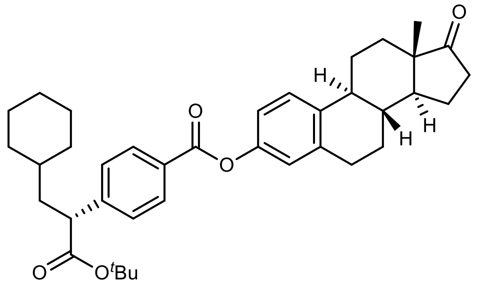

Prepared by **GP-A**. White solid, 79.8 mg, 68% yield. m.p. = 76-77 ºC; ^{1}H NMR (400 MHz, $CDCl_3$) δ 8.16 – 8.10 (m, 2H), 7.47 – 7.40 (m, 2H), 7.35 – 7.30 (m, 1H), 7.00 – 6.91 (m, 2H), 3.67 (t, *J* = 7.8 Hz, 1H), 2.97 – 2.89 (m, 2H), 2.51 (dd, *J* = 18.8, 8.6 Hz, 1H), 2.46 – 2.39 (m, 1H), 2.32 (td, *J* = 10.7, 3.7 Hz, 1H), 2.20 – 2.12 (m, 1H), 2.11 – 2.02 (m, 2H), 2.01 – 1.94 (m, 2H), 1.78 – 1.58 (m, 8H), 1.57 – 1.43 (m, 4H), 1.40 (s, 9H), 1.21 – 1.09 (m, 4H), 0.98 – 0.87 (m, 5H); ^{13}C NMR (101 MHz, $CDCl_3$) δ 220.8, 172.7, 165.3, 148.8, 146.1, 138.0, 137.4, 130.3, 128.2, 128.1, 126.4, 121.7, 118.8, 80.9, 50.4, 50.1, 47.9, 44.2, 40.9, 38.0, 35.8, 35.4, 33.3, 33.0, 31.6, 29.4, 27.9, 26.4, 26.3, 26.13, 26.10, 25.8, 21.6, 13.8. IR (film): ν (cm^{-1}) 3312, 2975, 2924, 2852, 1732, 1608, 1494, 1450, 1367, 1260, 1223, 1209, 1177, 1144, 1069, 1017, 1007, 840, 815, 755, 732, 698; HR-MS (ESI) m/z calcd for $C_{38}H_{48}NaO_5^+$ [M+Na$^+$] 607.33940, found 607.33929; $[\alpha]_D^{24.3}$ = 55.5 (c = 0.1, $CHCl_3$); d.r.>20:1.

(1*S*, 2*R*, 5*S*)-2-Isopropyl-5-methylcyclohexyl 4-((*R*)-1-(*tert*-butoxy)-3-cyclohexyl-1-oxopropan-2-yl)benzoate (43)

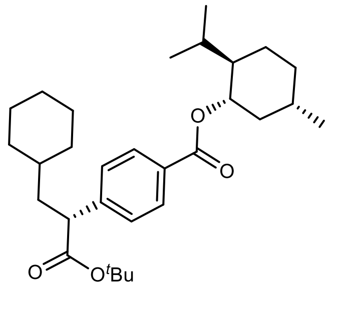

Prepared by **GP-A**. Colorless oil, 65.6 mg, 70% yield. ^{1}H NMR (400 MHz, $CDCl_3$) δ 8.02 – 7.93 (m, 2H), 7.40 – 7.32 (m, 2H), 4.92 (td, *J* = 10.9, 4.4 Hz, 1H), 3.63 (t, *J* = 7.8 Hz, 1H), 2.17 – 2.07 (m, 1H), 2.02 – 1.88 (m, 2H), 1.79 – 1.49 (m, 10H), 1.38 (s, 9H), 1.20 – 1.05 (m, 6H), 0.98 – 0.85 (m, 9H), 0.81 (d, *J* = 8.2 Hz, 3H); ^{13}C NMR (101 MHz, $CDCl_3$) δ 172.9, 166.0, 145.1, 129.7, 129.5, 127.9, 80.8, 74.7, 49.9, 47.2, 41.00, 40.97, 35.3, 34.3, 33.2, 33.0, 31.4, 27.9, 26.44, 26.40, 26.11, 26.07, 23.6, 22.0, 20.8, 16.4. IR (film): ν (cm^{-1}) 2954, 2924, 2868, 2851, 1714, 1610, 1450, 1367, 1273, 1178, 1144, 1129, 1110, 1020, 982, 962, 915, 857, 842, 756, 733, 704, 474, 466; HR-MS (ESI) m/z calcd for $C_{30}H_{46}NaO_4^+$ [M+Na$^+$]

493.32883, found 493.32920; $[\alpha]_D^{24.2}$ = -47.3 (c = 0.1, $CHCl_3$); d.r.>20:1.

(3*S*, 8*R*, 9*S*, 10*S*, 13*R*, 14*S*, 17*R*)-10, 13-Dimethyl-17-((*R*)-6-methylheptan-2-yl)hexadecahydro-1H-cyclopenta[a]phenanthren-3-yl 4-((*R*)-1-(*tert*-butoxy)-3-cyclohexyl-1-oxopropan-2-yl)benzoate (44)

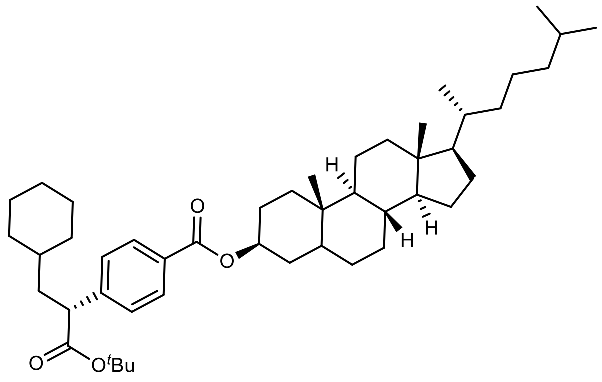

Prepared by **GP-A**. Colorless oil, 57.5 mg, 41% yield. ^{1}H NMR (400 MHz, $CDCl_3$) δ 8.00 – 7.94 (m, 2H), 7.39 – 7.31 (m, 2H), 4.99 – 4.87 (m, 1H), 3.62 (t, *J* = 7.8 Hz, 1H), 2.01 – 1.90 (m, 3H), 1.86 – 1.77 (m, 2H), 1.76 – 1.64 (m, 7H), 1.63 – 1.58 (m, 3H), 1.55 – 1.47 (m, 3H), 1.37 (s, 9H), 1.35 – 1.20 (m, 10H), 1.17 – 1.07 (m, 9H), 1.05 – 0.97 (m, 3H), 0.94 – 0.82 (m, 15H), 0.67 (d, *J* = 8.8 Hz, 4H); ^{13}C NMR (101 MHz, $CDCl_3$) δ 172.9, 166.0, 145.0, 129.7, 129.6, 127.8, 80.7, 74.3, 56.4, 56.3, 54.2, 50.0, 44.7, 42.6, 40.9, 40.0, 39.5, 36.8, 36.2, 35.8, 35.51, 35.49, 35.4, 34.1, 33.3, 33.0, 32.0, 28.6, 28.2, 28.0, 27.9, 27.6, 26.4, 26.13, 26.09, 24.2, 23.8, 22.8, 22.6, 21.2, 18.7, 12.3, 12.1. IR (film): ν (cm^{-1}) 2924, 2850, 1721, 1708, 1610, 1465, 1446, 1378, 1365, 1276, 1255, 1221, 1184, 1146, 1110, 1021, 1006, 852, 840, 757, 708, 414; HR-MS (ESI) m/z calcd for $C_{47}H_{74}NaO_4^+$ [M+Na$^+$] 725.54793, found 725.54807; $[\alpha]_D^{24.2}$ = 6.5 (c = 0.1, $CHCl_3$); d.r.>20:1.

(*R*)-2,5,7,8-Tetramethyl-2-((4*R*, 8*R*)-4, 8, 12-trimethyltridecyl)chroman-6-yl 4-((*R*)-1-(*tert*-butoxy)-3-cyclohexyl-1-oxopropan-2-yl)benzoate (45)

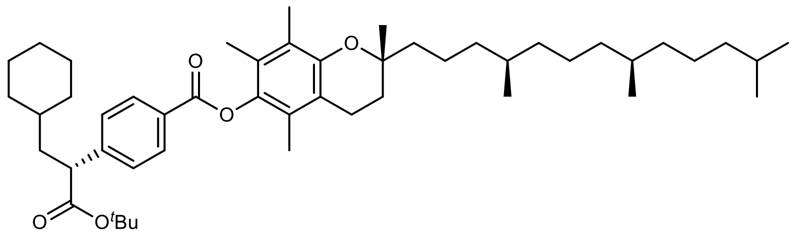

Prepared by **GP-A**. Colorless oil, 63.4 mg, 42% yield. ^{1}H NMR (400 MHz, $CDCl_3$) δ 8.23 – 8.16 (m, 2H), 7.51 – 7.41 (m, 2H), 3.70 (t, *J* = 7.8 Hz, 1H), 2.63 (t, *J* = 6.7 Hz, 2H), 2.13 (s, 3H), 2.07 (s, 3H), 2.03 (s, 3H), 2.01 – 1.93 (m, 1H), 1.89 – 1.60 (m, 9H), 1.60 – 1.50 (m, 3H), 1.45 – 1.36 (m, 12H), 1.33 – 1.23 (m, 10H), 1.23 – 1.03 (m, 11H), 1.01 – 0.91 (m, 2H), 0.90 – 0.83 (m, 12H); ^{13}C NMR (101 MHz, $CDCl_3$) δ 172.8, 165.0, 149.4, 145.9, 140.6, 130.3, 128.2, 128.1, 126.9, 125.1, 123.1, 117.4, 80.9, 75.0, 50.0, 41.1, 40.4, 39.6, 39.4, 37.4, 37.3, 35.4, 33.2, 33.0, 32.8, 32.7, 31.2, 31.0, 27.9, 26.5, 26.14, 26.10, 24.8, 24.4, 24.2, 23.7, 22.7, 22.6, 21.0, 20.6, 19.7, 19.6, 13.1, 12.2, 11.8. IR (film): ν (cm^{-1}) 2924, 2852, 1731, 1611, 1451, 1414, 1377, 1367, 1273, 1235, 1178, 1146, 1090, 1018, 913, 855, 842, 756, 733, 702; HR-MS (ESI) m/z calcd for $C_{49}H_{76}NaO_5^+$ [M+Na$^+$] 767.55850, found 767.55824; $[\alpha]_D^{24.2}$ = -6.0 (c = 0.1, $CHCl_3$); d.r.>20:1.

***tert*-Butyl (*R*)-2-(4-cyanophenyl)-3-cyclopentylpropanoate (46)**

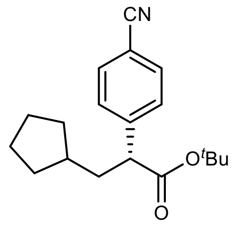

Prepared by **GP-A**. White solid, 42.6 mg, 71% yield. m.p. = 65-66 °C; ^{1}H NMR (400 MHz, $CDCl_3$) δ 7.63 – 7.57 (m, 2H), 7.44 – 7.38 (m, 2H), 3.54 (t, *J* = 7.7 Hz, 1H), 2.04 (dt, *J* = 13.5, 7.7 Hz, 1H), 1.79 – 1.68 (m, 3H), 1.65 – 1.56 (dt, *J* = 11.3, 7.7 Hz, 3H), 1.51 – 1.43 (m, 2H), 1.39 (s, 9H), 1.17 – 1.03 (m, 2H); ^{13}C NMR (101 MHz, $CDCl_3$) δ 172.3, 145.4, 132.2, 128.8, 118.8, 110.8, 81.2, 52.1, 39.7, 37.9, 32.7, 32.3, 27.9, 25.05, 25.01. IR (film):

ν (cm^{-1}) 2976, 2944, 2917, 2860, 2229, 1721, 1605, 1505, 1447, 1440, 1390, 1366, 1350, 1290, 1249, 1210, 1149, 1136, 1089, 1022, 859, 839, 783, 757, 554, 482; HR-MS (ESI) m/z calcd for $C_{19}H_{25}NNaO_2^+$ [M+Na$^+$] 322.17775, found 322.17736; $[\alpha]_D^{24.1}$ = -17.8 (c = 0.1, $CHCl_3$); HPLC conditions: Chiral-NR column, hexane/2-propanol = 98/2, flow rate = 1.0 mL/min, λ = 230 nm, t_R = 15.1 min (minor), t_R = 19.4 min (major), 97:3 er.

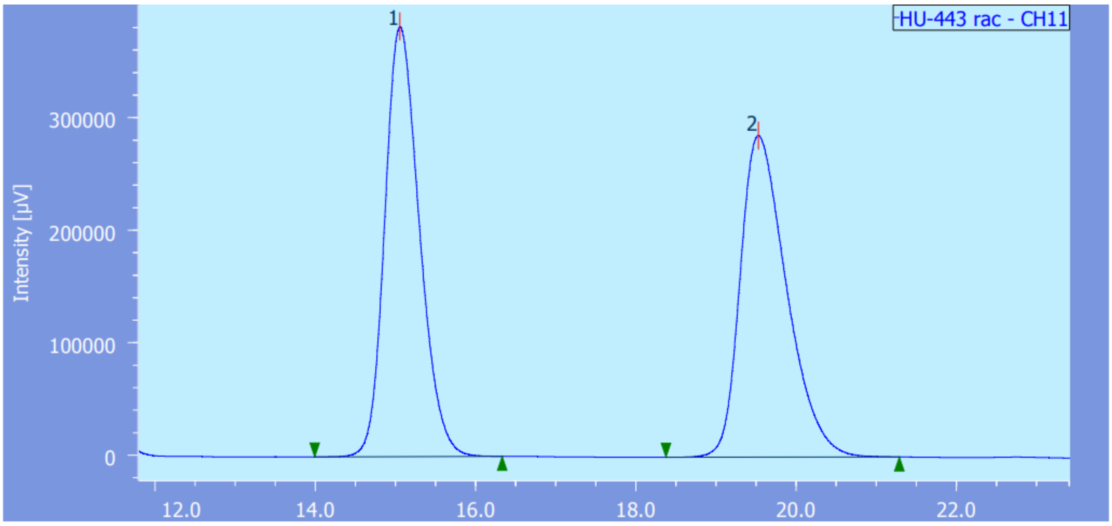

Decision

#	Peak Name	CH	tR [min]	Area [μV·sec]	Height [μV]	Area%	Height%	Quantity	NTP	Resolution	Symmetry Factor	Warning
1	Unknown	11	15.053	11472480	382009	49.960	57.218	N/A	5902	4.898	1.223	
2	Unknown	11	19.527	11490771	285625	50.040	42.782	N/A	5557	N/A	1.418	

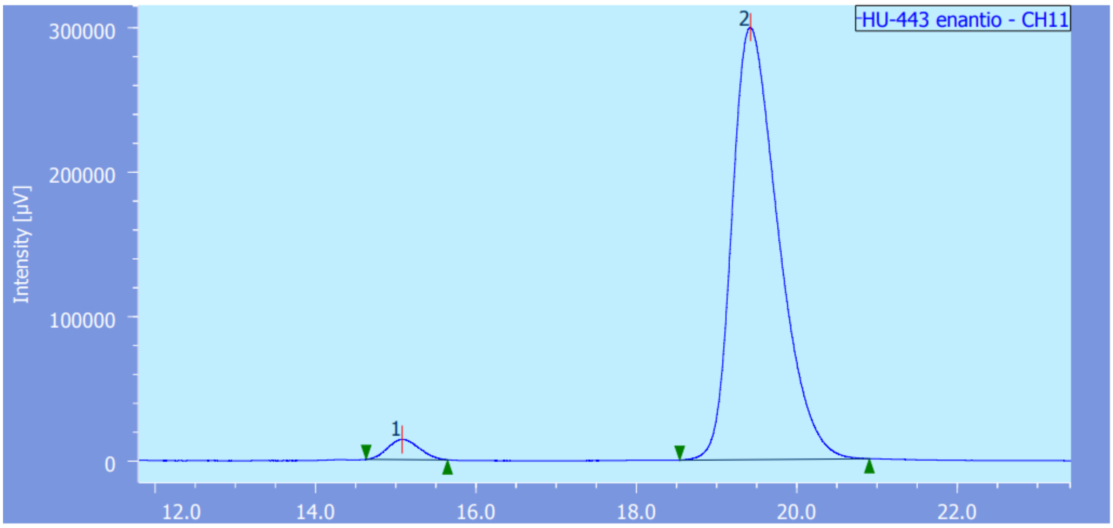

Decision

#	Peak Name	CH	tR [min]	Area [μV·sec]	Height [μV]	Area%	Height%	Quantity	NTP	Resolution	Symmetry Factor	Warning
1	Unknown	11	15.080	387593	13976	3.134	4.463	N/A	6411	4.841	1.115	
2	Unknown	11	19.420	11981193	299155	96.866	95.537	N/A	5531	N/A	1.407	

***tert*-Butyl (*R*)-2-(4-cyanophenyl)-3-cycloheptylpropanoate (47)**

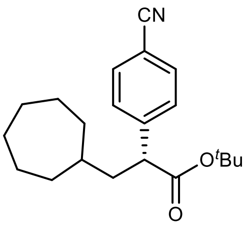

Prepared by **GP-A**. White solid, 48.3 mg, 74% yield. m.p. = 75-76 °C; ^{1}H NMR (400 MHz, $CDCl_3$) δ 7.63 – 7.56 (m, 2H), 7.44 – 7.38 (m, 2H), 3.59 (t, *J* = 7.8 Hz, 1H), 1.96 (ddd, *J* = 13.7, 8.2, 7.0 Hz, 1H), 1.73 – 1.65 (m, 2H), 1.65 – 1.56 (m, 3H), 1.55 – 1.42 (m, 4H), 1.39 (s, 9H), 1.37 – 1.27 (m, 3H), 1.24 – 1.13 (m, 2H); ^{13}C NMR (101 MHz, $CDCl_3$) δ 172.4, 145.4, 132.2, 128.8, 118.8, 110.8, 81.2, 50.6, 41.5, 36.8, 34.3, 34.2, 28.44, 28.41,

27.9, 26.2, 26.1. IR (film): ν (cm^{-1}) 2978, 2916, 2853, 2231, 1720, 1605, 1505, 1462, 1365, 1355, 1343, 1226, 1209, 1160, 1138, 1022, 863, 837, 762, 575, 550, 482; HR-MS (ESI) m/z calcd for $C_{21}H_{29}NNaO_2^+$ [M+Na$^+$] 350.20905, found 350.20917; $[\alpha]_D^{24.0}$ = -19.8 (c = 0.1, $CHCl_3$); HPLC conditions: Chiral-NR column, hexane/2-propanol = 98/2, flow rate = 1.0 mL/min, λ = 250 nm, t_R = 15.1 min (minor), t_R = 19.1 min (major), 96:4 er.

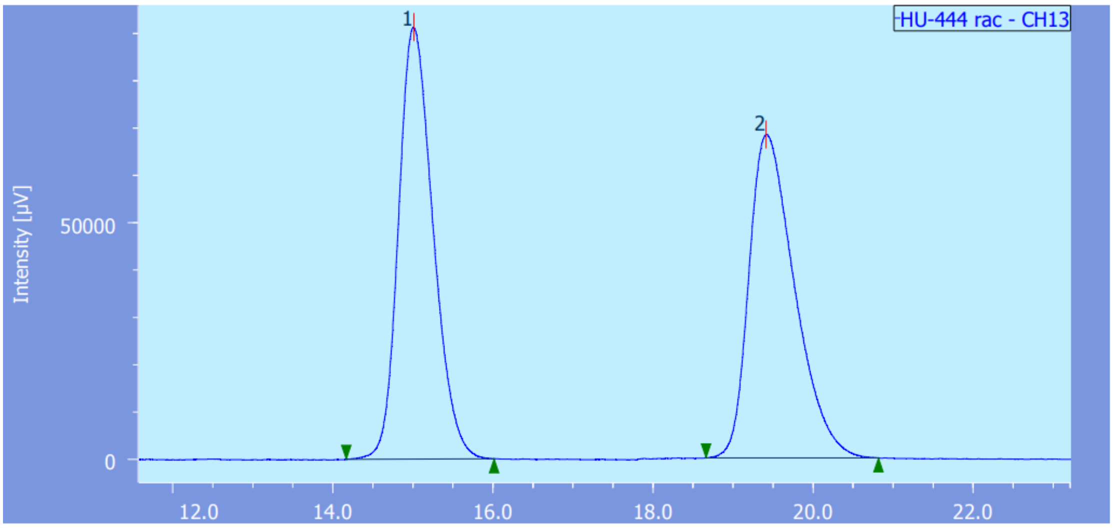

Decision

#	Peak Name	CH	tR [min]	Area [µV·sec]	Height [µV]	Area%	Height%	Quantity	NTP	Resolution	Symmetry Factor	Warning
1	Unknown	13	15.010	2739027	91179	50.249	57.187	N/A	5856	4.843	1.217	
2	Unknown	13	19.410	2711880	68262	49.751	42.813	N/A	5602	N/A	1.475	

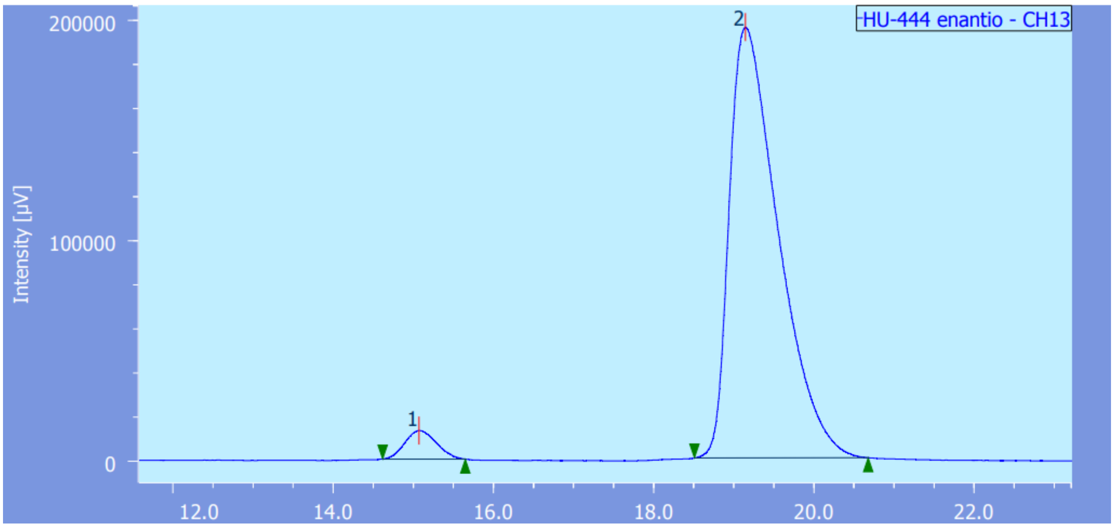

Decision

#	Peak Name	CH	tR [min]	Area [µV·sec]	Height [µV]	Area%	Height%	Quantity	NTP	Resolution	Symmetry Factor	Warning
1	Unknown	13	15.070	357196	12962	4.123	6.221	N/A	6554	4.401	1.138	
2	Unknown	13	19.143	8305441	195409	95.877	93.779	N/A	4747	N/A	1.804	

***tert*-Butyl (*R*)-2-(4-cyanophenyl)-3-cyclooctylpropanoate (48)**

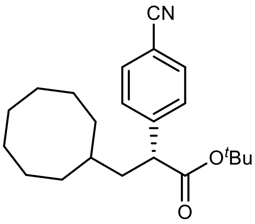

Prepared by **GP-A**. White solid, 48.5 mg, 71% yield. m.p. = 57-58 °C; ^{1}H NMR (400 MHz, $CDCl_3$) δ 7.63 – 7.56 (m, 2H), 7.44 – 7.38 (m, 2H), 3.60 (dd, *J* = 8.4, 7.1 Hz, 1H), 1.95 (ddd, *J* = 13.7, 8.5, 6.6 Hz, 1H), 1.70 – 1.45 (m, 10H), 1.43 – 1.34 (m, 13H), 1.31 – 1.22 (m, 2H); ^{13}C NMR (101 MHz, $CDCl_3$) δ 172.3, 145.5, 132.2, 128.7, 118.8, 110.8, 81.2, 50.6, 41.6, 34.7, 32.1, 32.0, 27.9, 27.2, 27.1, 26.2, 25.3, 25.2. IR (film): ν (cm^{-1}) 2974,

2913, 2849, 2231, 1715, 1606, 1506, 1472, 1460, 1446, 1364, 1313, 1226, 1219, 1146, 1027, 840, 757, 568, 546, 484; HR-MS (ESI) m/z calcd for $C_{22}H_{31}NaNO_2^+$ [M+Na$^+$] 364.22470, found 364.22423; $[\alpha]_D^{24.0}$ = -22.0 (c = 0.1, $CHCl_3$); HPLC conditions: Chiral-NR column, hexane/2-propanol = 98/2, flow rate = 1.0 mL/min, λ = 240 nm, t_R = 15.0 min (minor), t_R = 19.5 min (major), 95:5 er.

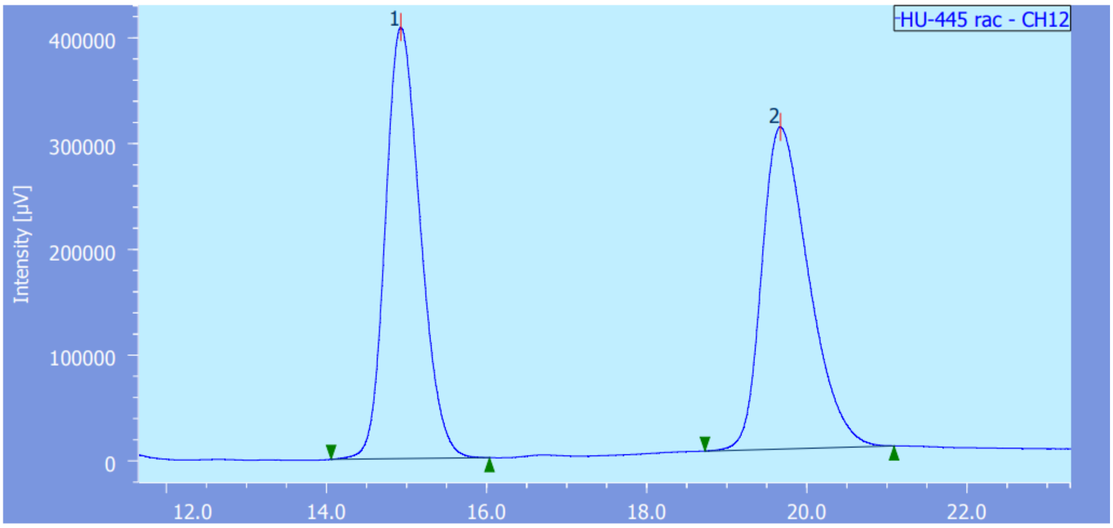

Decision

#	Peak Name	CH	tR [min]	Area [µV·sec]	Height [µV]	Area%	Height%	Quantity	NTP	Resolution	Symmetry Factor	Warning
1	Unknown	12	14.927	12369931	407684	49.849	57.259	N/A	5645	5.101	1.207	
2	Unknown	12	19.667	12444684	304313	50.151	42.741	N/A	5416	N/A	1.406	

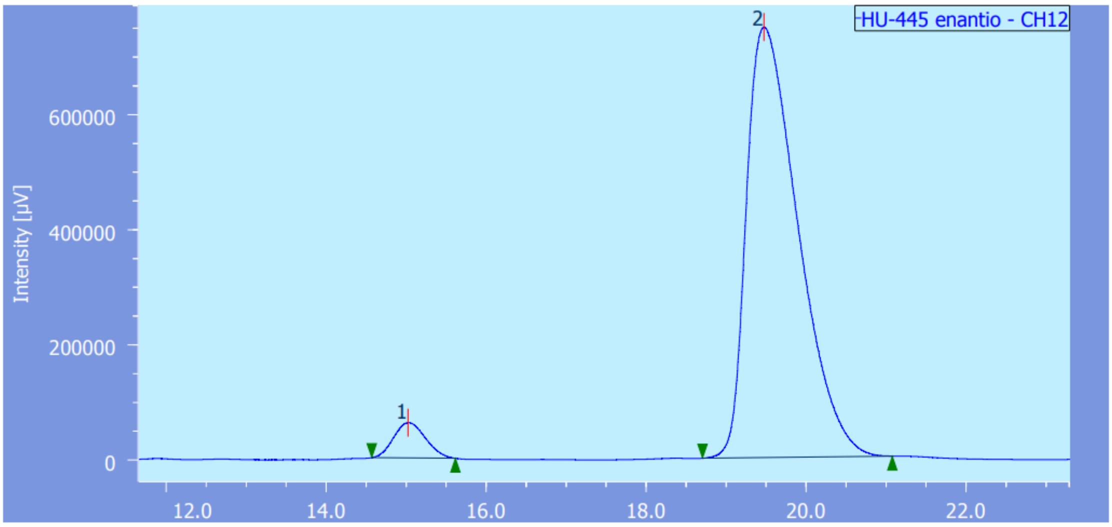

Decision

#	Peak Name	CH	tR [min]	Area [µV·sec]	Height [µV]	Area%	Height%	Quantity	NTP	Resolution	Symmetry Factor	Warning
1	Unknown	12	15.023	1705864	61447	4.848	7.598	N/A	6414	4.606	1.137	
2	Unknown	12	19.470	33478543	747254	95.152	92.402	N/A	4314	N/A	1.694	

***tert*-Butyl (*R*)-2-(4-cyanophenyl)-3-cyclododecylpropanoate (49)**

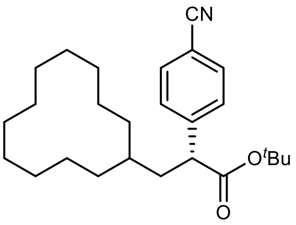

Prepared by **GP-B**. Colorless oil, 52.5 mg, 66% yield. ^{1}H NMR (400 MHz, $CDCl_3$) δ 7.60 (d, *J* = 8.3 Hz, 2H), 7.42 (d, *J* = 8.3 Hz, 2H), 3.61 (t, *J* = 7.8 Hz, 1H), 1.96 (ddd, *J* = 13.7, 8.3, 5.7 Hz, 1H), 1.62 – 1.53 (m, 1H), 1.39 (s, 9H), 1.35 – 1.15 (m, 23H); ^{13}C NMR (101 MHz, $CDCl_3$) δ 172.3, 145.5, 132.2, 128.8, 118.8, 110.8, 81.2, 50.6, 38.8, 31.5, 29.1, 29.0, 27.9, 24.6, 24.4, 23.8, 23.3, 23.23, 23.21, 21.6, 21.5.

IR (film): ν (cm^{-1}) 2930, 2860, 2360, 2340, 2228, 2176, 2159, 2033, 2025, 2016, 1976, 1727, 1607, 1505, 1470, 1455, 1446, 1367, 1252, 1143, 842, 733; HR-MS (ESI) m/z calcd for $C_{26}H_{39}NaNO_2^+$ [M+Na$^+$] 420.28730, found 420.28719; $[\alpha]_D^{23.2}$ = -14.1 (c = 0.1, $CHCl_3$); HPLC conditions: Chiral-NR column, hexane/2-propanol = 98/2, flow rate = 1.0 mL/min, λ = 230 nm, t_R = 14.8 min (minor), t_R = 18.9 min (major), 96:4 er.

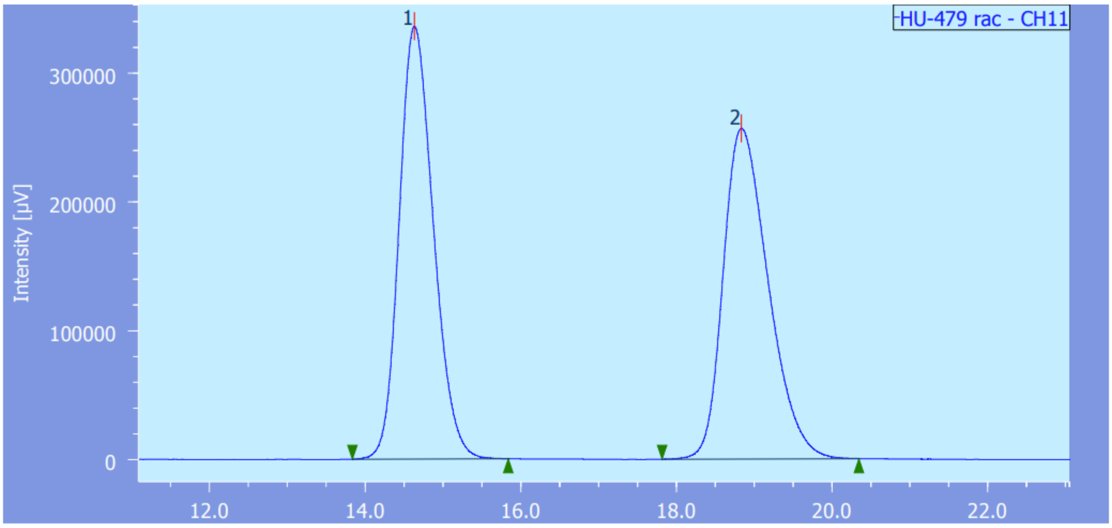

Decision

#	Peak Name	CH	tR [min]	Area [µV·sec]	Height [µV]	Area%	Height%	Quantity	NTP	Resolution	Symmetry Factor	Warning
1	Unknown	11	14.633	10246878	335665	49.807	56.686	N/A	5343	4.540	1.192	
2	Unknown	11	18.833	10326471	256485	50.193	43.314	N/A	5104	N/A	1.348	

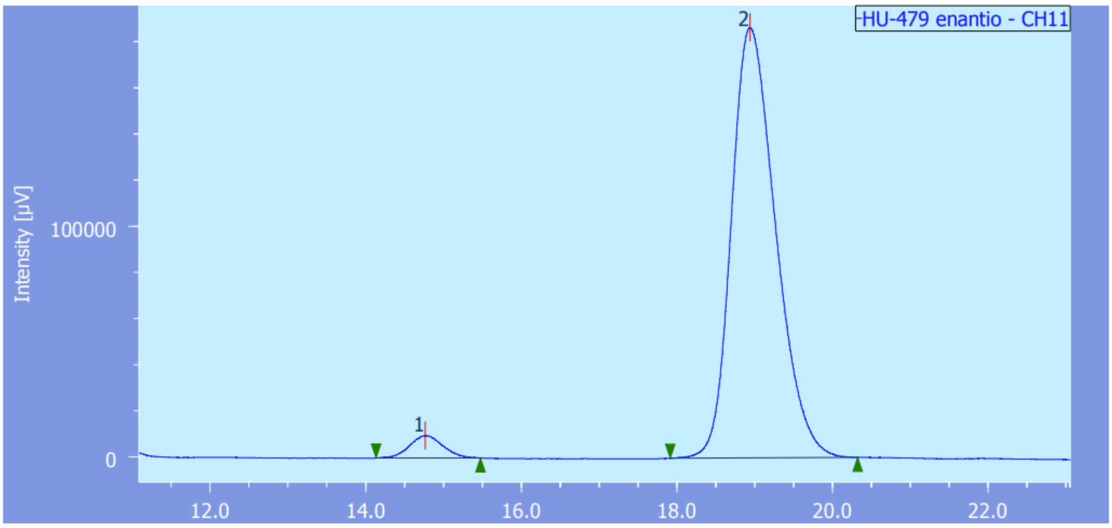

Decision

#	Peak Name	CH	tR [min]	Area [µV·sec]	Height [µV]	Area%	Height%	Quantity	NTP	Resolution	Symmetry Factor	Warning
1	Unknown	11	14.763	292048	9685	3.817	4.937	N/A	5605	4.594	1.081	
2	Unknown	11	18.937	7359539	186484	96.183	95.063	N/A	5377	N/A	1.283	

***tert*-Butyl (*R*)-2-(4-cyanophenyl)-4,4,5-trimethylhexanoate (50)**

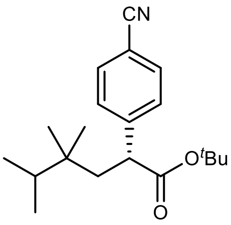

Prepared by **GP-A**. White solid, 32.0 mg, 51% yield. m.p. = 70-71 °C; ^{1}H NMR (400 MHz, $CDCl_3$) δ 7.61 – 7.56 (m, 2H), 7.45 – 7.38 (m, 2H), 3.57 (dd, J = 8.9, 3.5 Hz, 1H), 2.27 (dd, J = 14.1, 8.9 Hz, 1H), 1.54 – 1.44 (m, 2H), 1.36 (s, 9H), 0.87 – 0.76 (m, 12H); ^{13}C NMR (101 MHz, $CDCl_3$) δ 172.8, 147.2, 132.3, 128.5, 118.8, 110.7, 81.1, 48.8, 43.1, 36.0, 35.9, 27.8, 24.3, 23.7, 17.41, 17.38. IR (film): ν (cm^{-1}) 2966, 2931, 2870, 2226, 1722,

1602, 1468, 1455, 1390, 1364, 1329, 1270, 1243, 1214, 1144, 1115, 875, 842, 827, 756, 573, 547; HR-MS (ESI) m/z calcd for $C_{20}H_{29}NNaO_2^+$ [M+Na$^+$] 338.20905, found 338.20856; $[\alpha]_D^{24.1}$ = -23.0 (c = 0.1, $CHCl_3$); HPLC conditions: Chiral-NR column, hexane/2-propanol = 98/2, flow rate = 1.0 mL/min, λ = 230 nm, t_R = 11.0 min (minor), t_R = 13.9 min (major), 97:3 er.

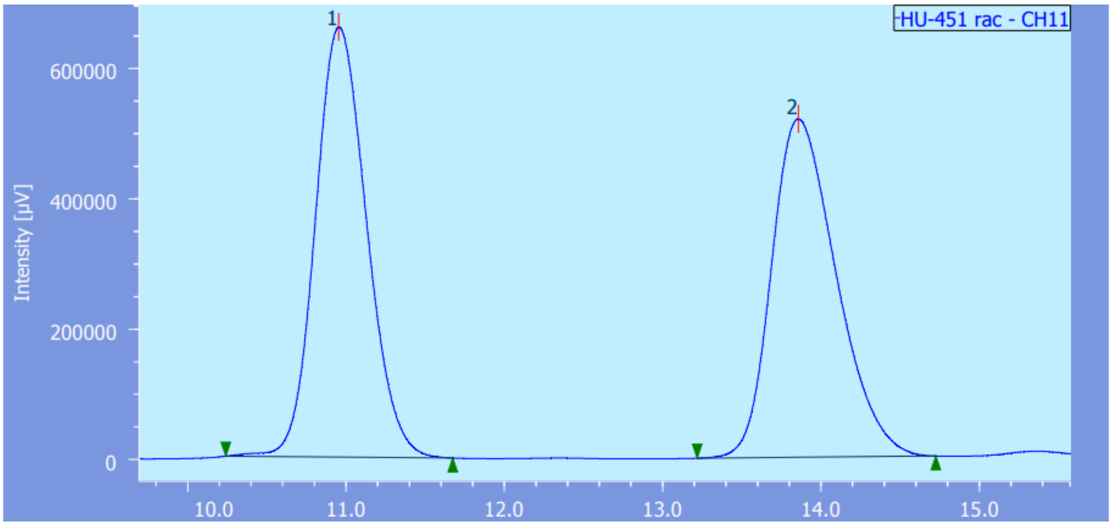

Decision

#	Peak Name	CH	tR [min]	Area [μV·sec]	Height [μV]	Area%	Height%	Quantity	NTP	Resolution	Symmetry Factor	Warning
1	Unknown	11	10.953	14859792	659434	50.205	55.973	N/A	5485	4.343	1.144	
2	Unknown	11	13.857	14738160	518699	49.795	44.027	N/A	5477	N/A	1.308	

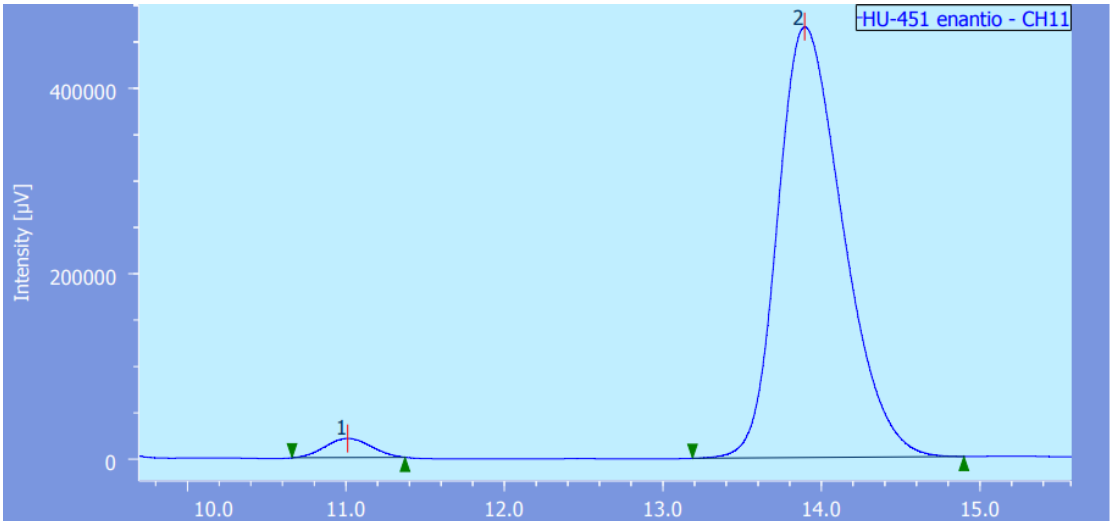

Decision

#	Peak Name	CH	tR [min]	Area [μV·sec]	Height [μV]	Area%	Height%	Quantity	NTP	Resolution	Symmetry Factor	Warning
1	Unknown	11	11.010	421295	20738	3.072	4.273	N/A	6299	4.429	1.035	
2	Unknown	11	13.893	13292272	464614	96.928	95.727	N/A	5483	N/A	1.306	

***tert*-Butyl (*R*)-6-chloro-2-(4-cyanophenyl)-4,4-dimethylhexanoate (51)**

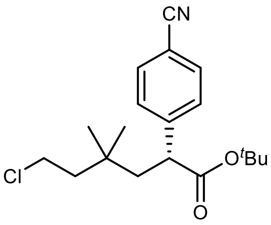

Prepared by **GP-A**. White solid, 39.1 mg, 58% yield. m.p. = 45-46 °C; ^{1}H NMR (400 MHz, $CDCl_3$) δ 7.62 – 7.56 (m, 2H), 7.44 – 7.37 (m, 2H), 3.56 (dd, *J* = 9.1, 3.4 Hz, 1H), 3.54 – 3.40 (m, 2H), 2.30 (dd, *J* = 14.2, 9.1 Hz, 1H), 1.81 – 1.68 (m, 2H), 1.47 (dd, *J* = 14.2, 3.4 Hz, 1H), 1.36 (s, 9H), 0.91 (s, 6H); ^{13}C NMR (101 MHz, $CDCl_3$) δ 172.3, 146.4, 132.4, 128.4, 118.7, 111.0, 81.5, 48.8, 45.0, 44.9, 40.7, 34.0, 27.7, 27.0, 26.7. IR (film): ν (cm^{-1}) 2978, 2955, 2924, 2874, 2229, 1726, 1604, 1501, 1470, 1456, 1392, 1367, 1340,

1254, 1211, 1143, 1021, 869, 839, 783, 729, 718, 567, 550; HR-MS (ESI) m/z calcd for $C_{19}H_{26}ClNNaO_2^+$ [M+Na$^+$] 358.15443, found 358.15388; $[\alpha]_D^{24.1}$ = -28.2 (c = 0.1, $CHCl_3$); HPLC conditions: Chiral-NR column, hexane/2-propanol = 98/2, flow rate = 1.0 mL/min, λ = 250 nm, t_R = 16.6 min (minor), t_R = 19.5 min (major), 95:5 er.

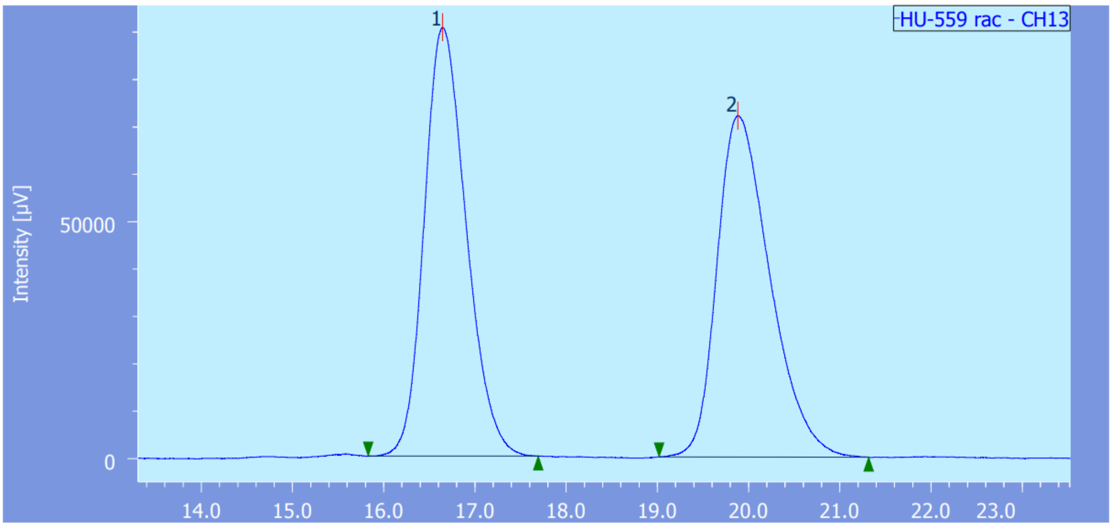

Decision

#	Peak Name	CH	tR [min]	Area [µV·sec]	Height [µV]	Area%	Height%	Quantity	NTP	Resolution	Symmetry Factor	Warning
1	Unknown	13	16.643	2897603	90462	49.864	55.674	N/A	6314	3.434	1.214	
2	Unknown	13	19.880	2913450	72024	50.136	44.326	N/A	5710	N/A	1.395	

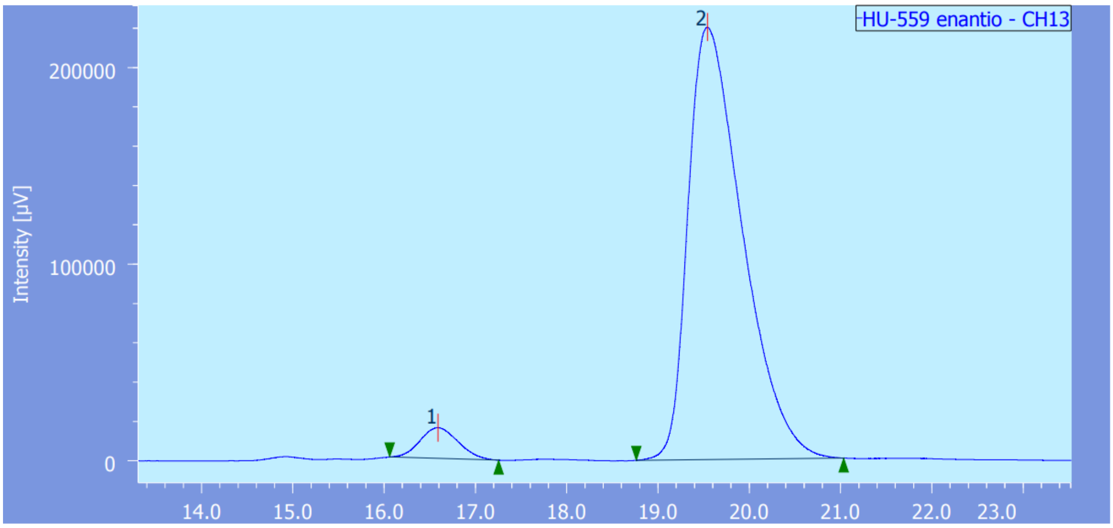

Decision

#	Peak Name	CH	tR [min]	Area [µV·sec]	Height [µV]	Area%	Height%	Quantity	NTP	Resolution	Symmetry Factor	Warning
1	Unknown	13	16.590	454858	15582	4.757	6.621	N/A	7207	3.168	1.096	
2	Unknown	13	19.540	9107504	219747	95.243	93.379	N/A	5182	N/A	1.616	

***tert*-Butyl (*R*)-6-bromo-2-(4-cyanophenyl)-4,4-dimethylhexanoate (52)**

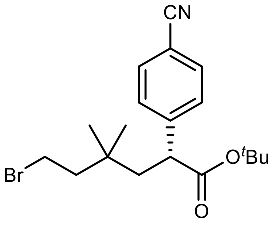

Prepared by **GP-B**. White solid, 35.9 mg, 47% yield. m.p. = 67-68 °C; ^{1}H NMR (400 MHz, $CDCl_3$) δ 7.64 – 7.57 (m, 2H), 7.44 – 7.37 (m, 2H), 3.56 (dd, *J* = 9.0, 3.5 Hz, 1H), 3.34 (dddd, *J* = 15.4, 10.4, 9.5, 6.5 Hz, 2H), 2.30 (dd, *J* = 14.2, 9.0 Hz, 1H), 1.92 – 1.77 (m, 2H), 1.46 (dd, *J* = 10.9, 3.5 Hz, 1H), 1.37 (s, 9H), 0.91 (s, 6H); ^{13}C NMR (101 MHz, $CDCl_3$) δ 172.3, 146.4, 132.4, 128.4, 118.7, 111.0, 81.5, 48.8, 45.5, 44.8, 35.0, 28.6, 27.8, 26.8, 26.6. IR (film): ν (cm^{-1}) 2978, 2955, 2928, 2872, 2230, 1725, 1605, 1501, 1467,

1391, 1366, 1271, 1229, 1144, 1021, 871, 840, 772, 652, 568; HR-MS (ESI) m/z calcd for $C_{19}H_{26}BrNNaO_2^+$ [M+Na$^+$] 402.10391, found 402.10400; $[\alpha]_D^{24.2}$ = -29.6 (c = 0.1, $CHCl_3$); HPLC conditions: Chiral-NR column, hexane/2-propanol = 98/2, flow rate = 1.0 mL/min, λ = 250 nm, t_R = 17.2 min (minor), t_R = 20.5 min (major), 94:6 er.

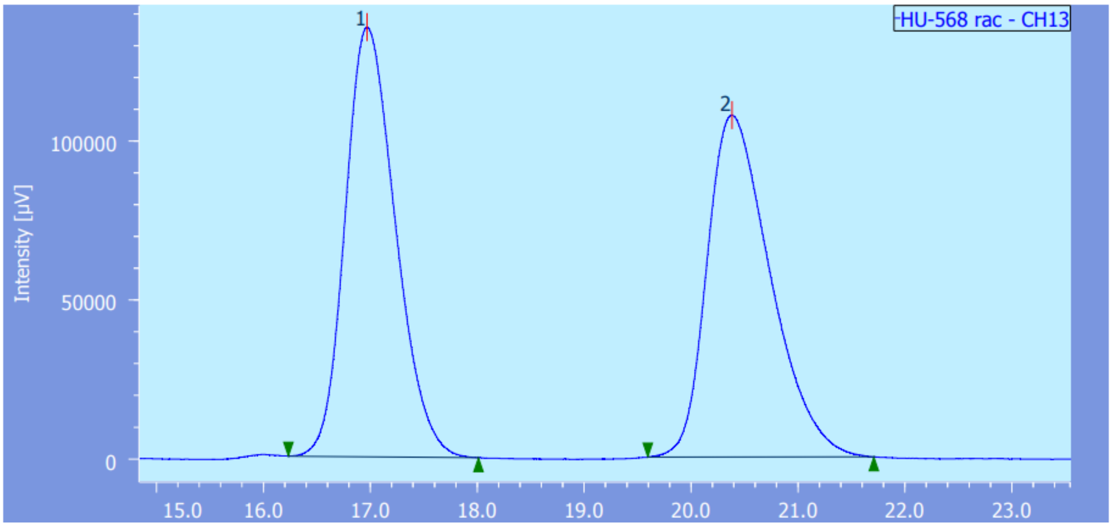

Decision

#	Peak Name	CH	tR [min]	Area [µV·sec]	Height [µV]	Area%	Height%	Quantity	NTP	Resolution	Symmetry Factor	Warning
1	Unknown	13	16.970	4422487	135027	49.918	55.675	N/A	6210	3.521	1.214	
2	Unknown	13	20.380	4437034	107500	50.082	44.325	N/A	5692	N/A	1.416	

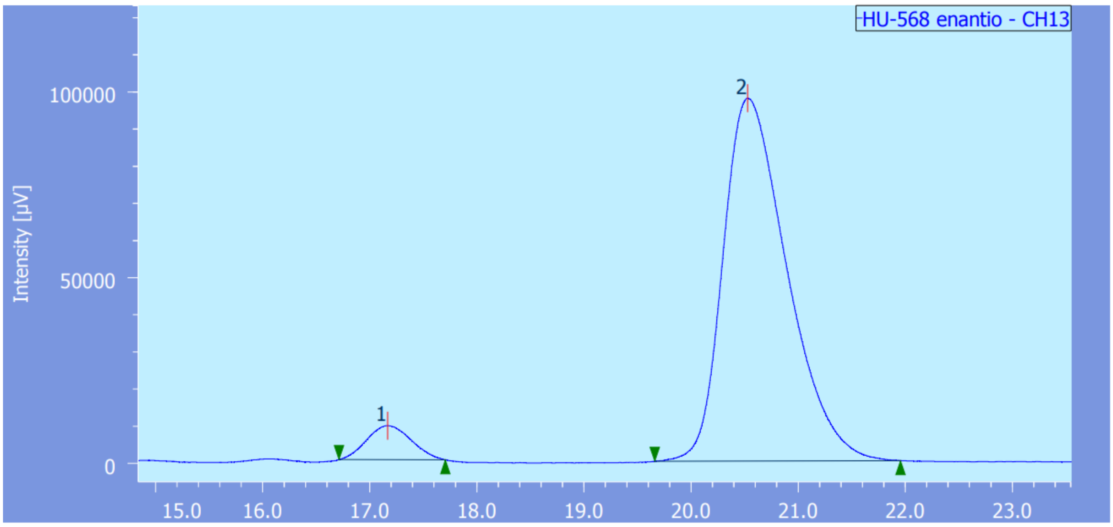

Decision

#	Peak Name	CH	tR [min]	Area [µV·sec]	Height [µV]	Area%	Height%	Quantity	NTP	Resolution	Symmetry Factor	Warning
1	Unknown	13	17.167	265401	9181	6.152	8.592	N/A	7495	3.594	1.086	
2	Unknown	13	20.527	4048602	97681	93.848	91.408	N/A	5760	N/A	1.426	

***tert*-Butyl (*R*)-6-cyano-2-(4-cyanophenyl)-4,4-dimethylhexanoate (53)**

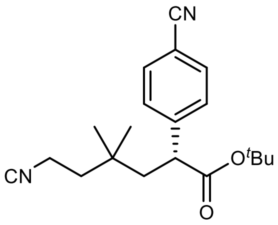

Prepared by **GP-A**. Colorless oil, 54.2 mg, 83% yield. ^1H NMR (400 MHz, $CDCl_3$) δ 7.62 – 7.57 (m, 2H), 7.41 – 7.37 (m, 2H), 3.54 (dd, J = 9.3, 3.3 Hz, 1H), 2.37 – 2.18 (m, 3H), 1.68 – 1.57 (m, 2H), 1.51 – 1.46 (m, 1H), 1.36 (s, 9H), 0.92 (s, 3H), 0.90 (s, 3H); ^{13}C NMR (101 MHz, $CDCl_3$) δ 172.2, 146.1, 132.5, 128.3, 120.1, 118.6, 111.0, 81.7, 48.6, 44.2, 37.3, 33.5, 27.7, 26.4, 26.1, 12.3. IR (film): ν (cm^{-1}) 2968, 2934, 2874, 2245, 2227, 1724, 1606, 1504, 1474, 1456, 1392, 1368, 1276, 1249, 1216, 1144, 1020, 840,

755, 564; HR-MS (ESI) m/z calcd for $C_{19}H_{26}N_2NaO_2^+$ [M+Na$^+$] 349.18865, found 349.18860; $[\alpha]_D^{24.1}$ = -28.5 (c = 0.1, $CHCl_3$); HPLC conditions: Chiral-NR column, hexane/2-propanol = 90/10, flow rate = 1.0 mL/min, λ = 230 nm, t_R = 43.4 min (minor), t_R = 46.6 min (major), 95:5 er.

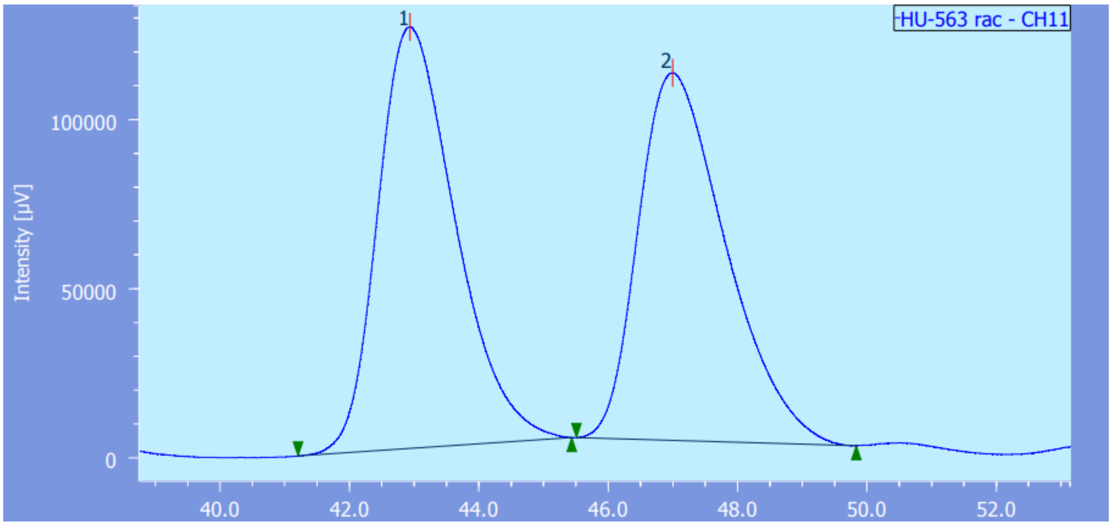

Decision

#	Peak Name	CH	tR [min]	Area [µV·sec]	Height [µV]	Area%	Height%	Quantity	NTP	Resolution	Symmetry Factor	Warning
1	Unknown	11	42.937	10400341	124531	50.412	53.421	N/A	6184	1.740	1.338	
2	Unknown	11	46.993	10230165	108581	49.588	46.579	N/A	5695	N/A	1.437	

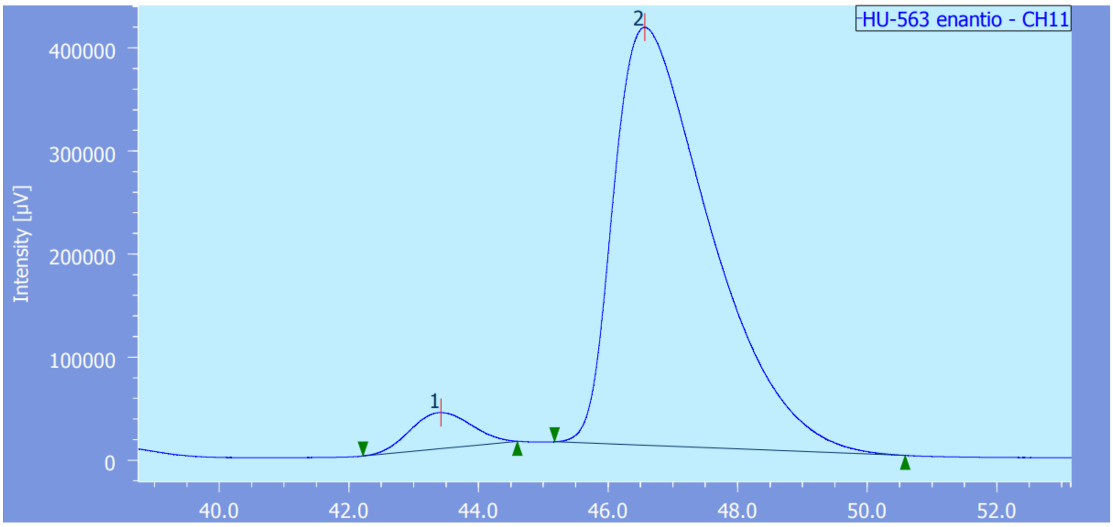

Decision

#	Peak Name	CH	tR [min]	Area [µV·sec]	Height [µV]	Area%	Height%	Quantity	NTP	Resolution	Symmetry Factor	Warning
1	Unknown	11	43.420	2300190	34863	5.167	7.921	N/A	9247	1.393	1.040	
2	Unknown	11	46.567	42219990	405286	94.833	92.079	N/A	4681	N/A	1.885	

***tert*-Butyl (*R*)-2-(4-cyanophenyl)-4,4-dimethyl-7-oxooctanoate (54)**

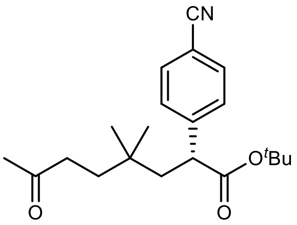

Prepared by **GP-A**. White solid, 52.5 mg, 76% yield. m.p. = 155-156 °C; ^{1}H NMR (400 MHz, $CDCl_3$) δ 7.60 – 7.55 (m, 2H), 7.41 – 7.36 (m, 2H), 3.55 (dd, *J* = 9.0, 3.4 Hz, 1H), 2.46 – 2.21 (m, 3H), 2.11 (s, 3H), 1.53 – 1.44 (m, 3H), 1.34 (s, 9H), 0.87 – 0.83 (m, 6H); ^{13}C NMR (101 MHz, $CDCl_3$) δ 208.8, 172.5, 146.7, 132.4, 128.4, 118.7, 110.8, 81.3, 48.8, 44.7, 38.6, 35.1, 33.0, 30.0, 27.7, 26.9, 26.6. IR (film): ν (cm^{-1}) 2961, 2935, 2871, 2228, 1717, 1605, 1502, 1471, 1391, 1368, 1276, 1256, 1143, 1020, 841, 786, 757, 563; HR-MS (ESI) m/z calcd for $C_{21}H_{29}NNaO_3^+$ [M+Na$^+$] 366.20396, found 366.20395;

[α]D $^{24.1}$ = -25.2 (c = 0.1, $CHCl_3$); HPLC conditions: Chiral-NR column, hexane/2-propanol = 90/10, flow rate = 0.5 mL/min, λ = 250 nm, t_R = 63.9 min (minor), t_R = 71.4 min (major), 95:5 er.

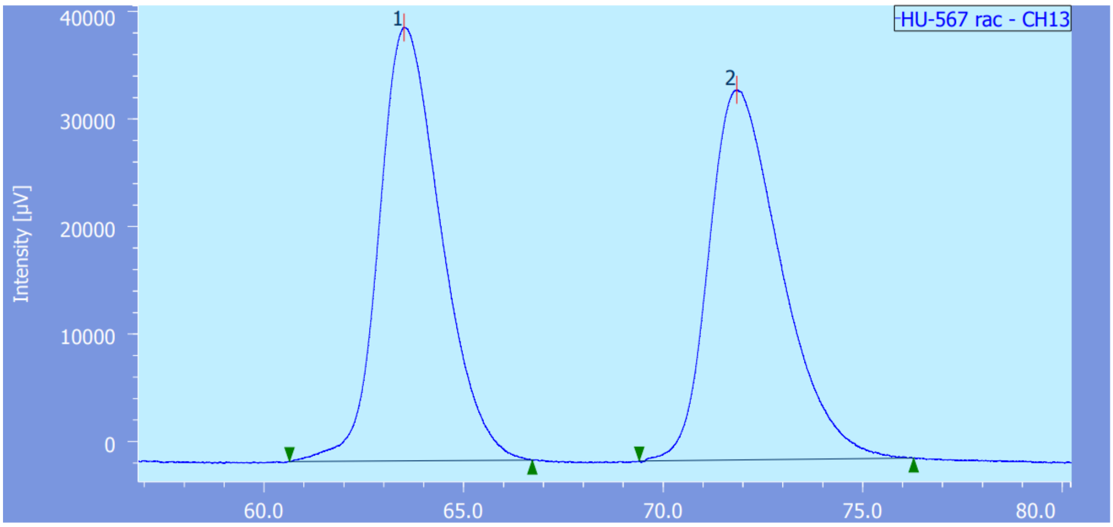

Decision

#	Peak Name	CH	tR [min]	Area [µV·sec]	Height [µV]	Area%	Height%	Quantity	NTP	Resolution	Symmetry Factor	Warning
1	Unknown	13	63.503	4361739	40299	50.136	53.933	N/A	8277	2.761	1.256	
2	Unknown	13	71.840	4338041	34421	49.864	46.067	N/A	7757	N/A	1.432	

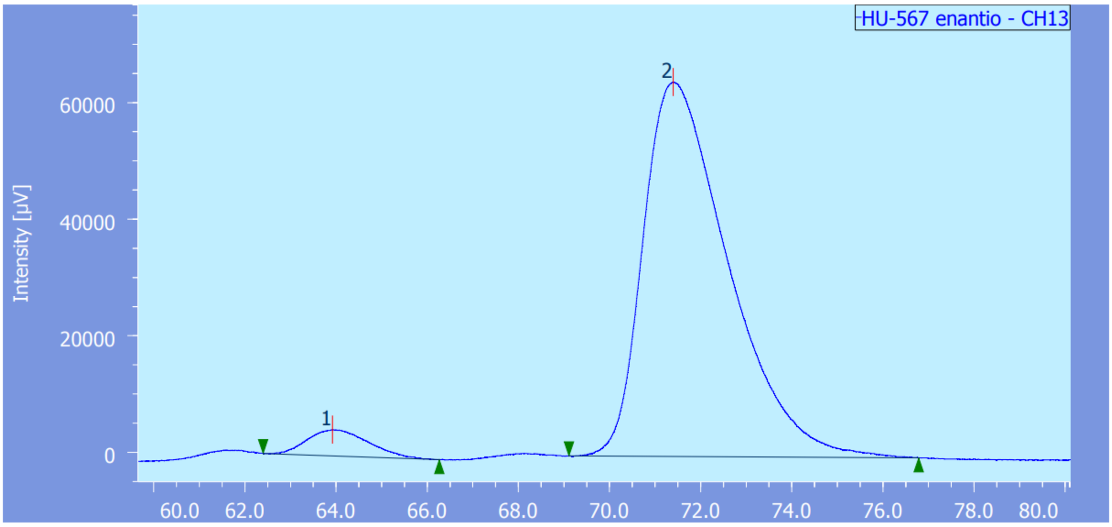

Decision

#	Peak Name	CH	tR [min]	Area [µV·sec]	Height [µV]	Area%	Height%	Quantity	NTP	Resolution	Symmetry Factor	Warning
1	Unknown	13	63.920	420305	4479	4.798	6.521	N/A	10115	2.540	1.282	
2	Unknown	13	71.393	8338994	64208	95.202	93.479	N/A	7230	N/A	1.626	

1-(*tert*-Butyl) 7-methyl (*R*)-2-(4-cyanophenyl)-4,4-dimethylheptanedioate (55)

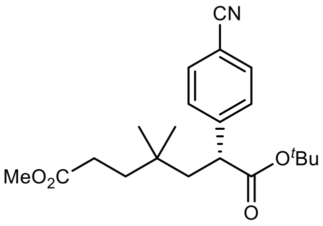

Prepared by **GP-B**. Colorless oil, 34.0 mg, 47% yield. ^{1}H NMR (400 MHz, $CDCl_3$) δ 7.60 – 7.56 (m, 2H), 7.42 – 7.38 (m, 2H), 3.64 (s, 3H), 3.57 (dd, *J* = 9.0, 3.5 Hz, 1H), 2.32 – 2.15 (m, 3H), 1.60 – 1.54 (m, 2H), 1.48 – 1.44 (m, 1H), 1.35 (s, 9H), 0.88 –0.83 (m, 6H); ^{13}C NMR (101 MHz, $CDCl_3$) δ 174.4, 172.4, 146.6, 132.4, 128.5, 118.7, 110.8, 81.3, 51.6, 48.8, 44.7, 36.5, 33.2, 29.2, 27.7, 26.7, 26.5. IR (film): ν (cm^{-1}) 2957, 2935, 2871, 2229, 1727, 1606, 1504, 1473, 1456, 1436, 1392, 1368, 1255, 1198, 1143, 1020, 984, 963, 841, 784, 757, 699, 564; HR-MS (ESI) m/z calcd for $C_{21}H_{29}NNaO_4^+$ [M+Na$^+$] 382.19888, found 382.19871; [α]D $^{23.8}$ = -12.5 (c = 0.1, $CHCl_3$); HPLC conditions: Chiral-

NR column, hexane/2-propanol = 95/5, flow rate = 1.0 mL/min, λ = 246 nm, t_R = 28.1 min (minor), t_R = 32.4 min (major), 97:3 er.

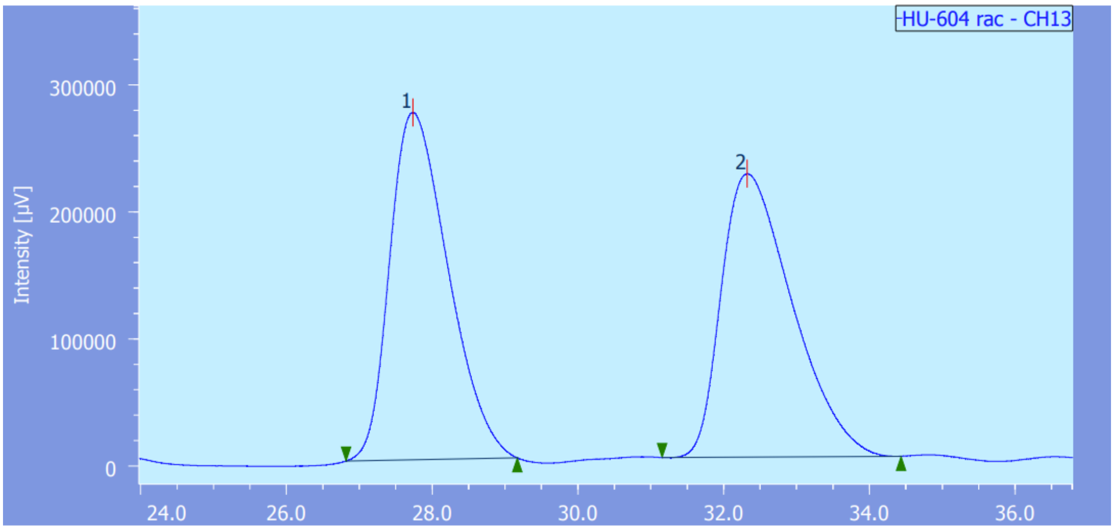

Decision

#	Peak Name	CH	tR [min]	Area [µV·sec]	Height [µV]	Area%	Height%	Quantity	NTP	Resolution	Symmetry Factor	Warning
1	Unknown	13	27.733	15240129	273387	50.227	55.066	N/A	5558	2.791	1.378	
2	Unknown	13	32.320	15102400	223083	49.773	44.934	N/A	5118	N/A	1.558	

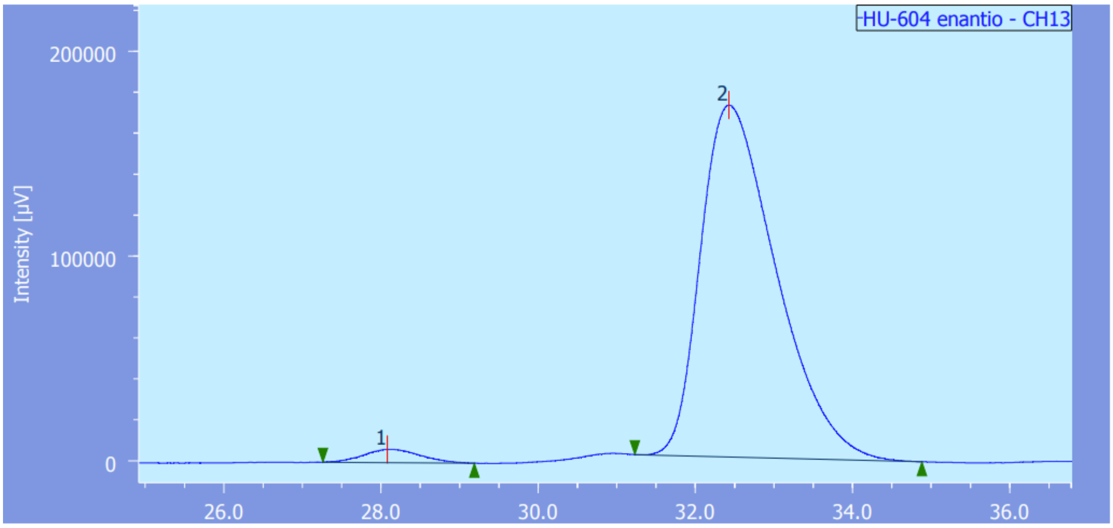

Decision

#	Peak Name	CH	tR [min]	Area [µV·sec]	Height [µV]	Area%	Height%	Quantity	NTP	Resolution	Symmetry Factor	Warning
1	Unknown	13	28.080	325391	6444	2.746	3.616	N/A	6841	2.787	1.149	
2	Unknown	13	32.423	11524922	171783	97.254	96.384	N/A	5384	N/A	1.524	

tert-Butyl (_R_)-2-(4-cyanophenyl)-5-hydroxy-4,4,5-trimethylhexanoate (56)

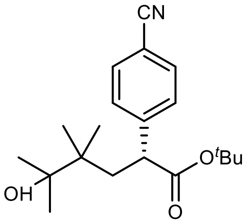

Prepared by **GP-A**. White solid, 53.8 mg, 81% yield. m.p. = 99-100 ºC; ^{1}H NMR (400 MHz, $CDCl_3$) δ 7.61 – 7.56 (m, 2H), 7.44 – 7.38 (m, 2H), 3.69 (dd, *J* = 9.2, 3.2 Hz, 1H), 2.48 (dd, *J* = 14.1, 9.2 Hz, 1H), 1.56 (dd, *J* = 14.1, 3.2 Hz, 1H), 1.36 (s, 9H), 1.21 (s, 3H), 1.18 (s, 3H), 0.91 (s, 3H), 0.90 (s, 3H); ^{13}C NMR (101 MHz, $CDCl_3$) δ 172.8, 147.2, 132.4, 128.5, 118.8, 110.7, 81.1, 75.4, 49.4, 40.5, 40.4, 27.8, 25.5, 22.2, 21.4. IR (film): ν (cm^{-1}) 3533, 2980, 2955, 2929, 2857, 2237, 1726, 1605, 1502, 1463, 1377, 1366, 1347, 1274, 1214, 1174, 1144, 1114, 1103, 946, 879, 835, 756, 573, 556, 477; HR-MS (ESI) m/z calcd for $C_{20}H_{29}NNaO_3^+$ [M+H$^+$] 354.20396, found 354.20409; $[\alpha]_D^{24.1}$ = -23.1 (c = 0.1, $CHCl_3$); HPLC

conditions: IC column, hexane/2-propanol = 90/10, flow rate = 1.0 mL/min, λ = 250 nm, t_R = 11.8 min (major), t_R = 14.1 min (minor), 96:4 er.

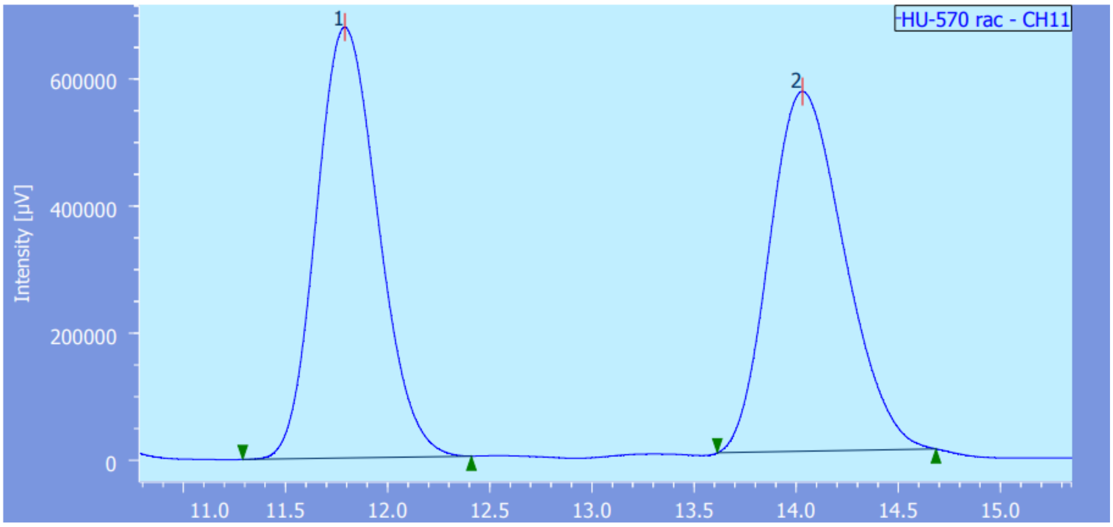

Decision

#	Peak Name	CH	tR [min]	Area [µV·sec]	Height [µV]	Area%	Height%	Quantity	NTP	Resolution	Symmetry Factor	Warning
1	Unknown	11	11.790	14282773	677839	49.706	54.484	N/A	7075	3.609	1.155	
2	Unknown	11	14.030	14451907	566275	50.294	45.516	N/A	6733	N/A	1.216	

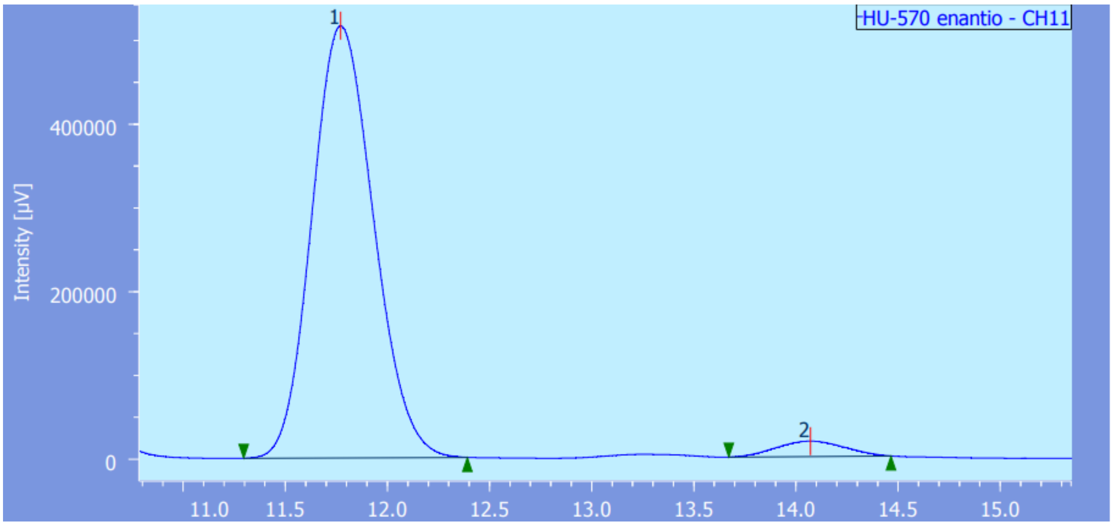

Decision

#	Peak Name	CH	tR [min]	Area [µV·sec]	Height [µV]	Area%	Height%	Quantity	NTP	Resolution	Symmetry Factor	Warning
1	Unknown	11	11.770	10840852	515725	96.175	96.555	N/A	7096	3.815	1.138	
2	Unknown	11	14.070	431179	18401	3.825	3.445	N/A	7496	N/A	1.018	

tert-Butyl (_R_)-2-(4-cyanophenyl)-4-isopropoxy-4-methylpentanoate (57)

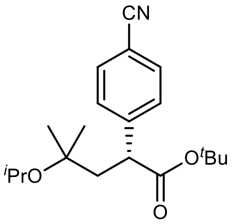

Prepared by **GP-A**. Colorless oil, 47.8 mg, 72% yield. ^{1}H NMR (400 MHz, $CDCl_3$) δ 7.60 – 7.54 (m, 2H), 7.43 – 7.37 (m, 2H), 3.77 (dd, J = 8.6, 3.5 Hz, 1H), 3.72 (dt, J = 12.3, 6.2 Hz, 1H), 2.44 (dd, J = 14.2, 8.7 Hz, 1H), 1.71 (dd, J = 14.2, 3.5 Hz, 1H), 1.34 (s, 9H), 1.14 (s, 3H), 1.10 (s, 3H), 1.07 (d, J = 6.1 Hz, 3H), 1.04 (d, J = 6.1 Hz, 3H); ^{13}C NMR (101 MHz, $CDCl_3$) δ 172.6, 146.8, 132.2, 128.7, 118.8, 110.6, 80.9, 74.4, 63.3, 48.4, 44.5, 27.7, 26.3, 26.0, 25.0, 24.9. IR (film): ν (cm^{-1}) 2973, 2931, 2872, 2229, 1727, 1607, 1503, 1457, 1382, 1367, 1274, 1244, 1172, 1141, 1114, 1006, 844, 822, 568, 552; HR-MS (ESI) m/z calcd for $C_{20}H_{29}NNaO_3^+$ [M+Na$^+$] 354.20396, found 354.20414; $[\alpha]_D^{24.1}$ = -35.4 (c = 0.1, $CHCl_3$); HPLC

conditions: Chiral-NR column, hexane/2-propanol = 98/2, flow rate = 1.0 mL/min, λ = 254 nm, t_R = 11.6 min (minor), t_R = 13.5 min (major), 96:4 er.

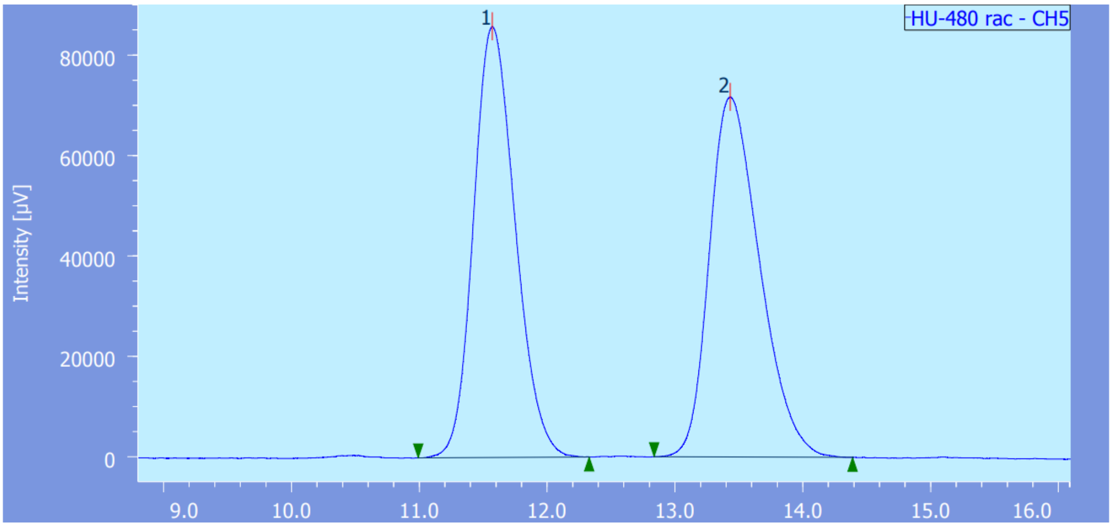

Decision

#	Peak Name	CH	tR [min]	Area [μV·sec]	Height [μV]	Area%	Height%	Quantity	NTP	Resolution	Symmetry Factor	Warning
1	Unknown	5	11.570	1943987	85784	49.712	54.480	N/A	6039	2.830	1.176	
2	Unknown	5	13.430	1966478	71675	50.288	45.520	N/A	5527	N/A	1.321	

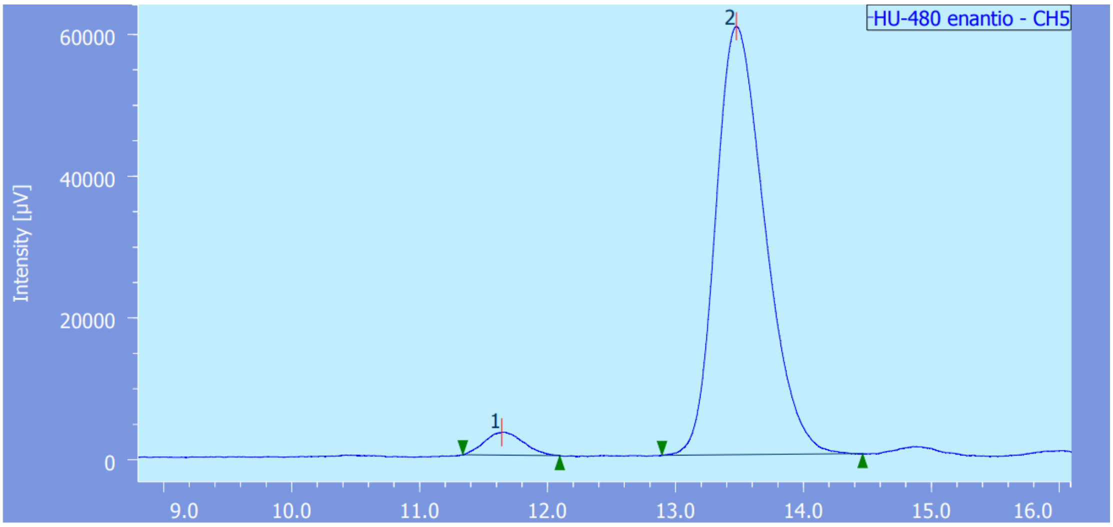

Decision

#	Peak Name	CH	tR [min]	Area [μV·sec]	Height [μV]	Area%	Height%	Quantity	NTP	Resolution	Symmetry Factor	Warning
1	Unknown	5	11.640	69191	3232	4.109	5.080	N/A	6321	2.857	1.169	
2	Unknown	5	13.473	1614860	60389	95.891	94.920	N/A	5909	N/A	1.290	

tert-Butyl (2_R_)-2-([1,1'-biphenyl]-4-yl)-3-(1,4-dioxan-2-yl)propanoate (58)

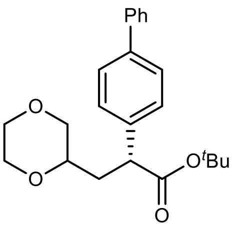

Prepared by **GP-A**. Colorless oil, 48.1 mg, 65% yield. ^{1}H NMR (400 MHz, $CDCl_3$) δ 7.62 – 7.51 (m, 4H), 7.47 – 7.39 (m, 2H), 7.38 – 7.31 (m, 3H), 3.83 – 3.24 (m, 8H), 2.22 – 2.04 (m, 1H), 1.84 – 1.66 (m, 1H), 1.44 – 1.37 (m, 9H); ^{13}C NMR (101 MHz, $CDCl_3$) δ 173.0, 172.6, 140.74, 140.71, 140.0, 139.9, 138.7, 138.0, 128.7, 128.4, 128.0, 127.29, 127.26, 127.2, 127.01, 126.99, 81.0, 80.7, 73.5, 72.8, 71.18, 71.15, 66.8, 66.6, 66.49, 66.47, 47.7, 47.5, 35.6, 34.9, 28.0, 27.9. IR (film): ν (cm^{-1}) 2963, 2913, 2850, 1723, 1487, 1450, 1366, 1260, 1241, 1146, 1121, 1079, 1008, 910, 873, 844, 760, 733, 697, 624; HR-MS (ESI) m/z calcd for $C_{23}H_{28}NaO_4^+$ [M+Na$^+$] 391.18798, found 391.18791; $[\alpha]_D^{24.1}$ = -51.7 (c = 0.1, $CHCl_3$); HPLC

conditions: Chiral-NR column, hexane/2-propanol = 99/1, flow rate = 0.5 mL/min, λ = 254 nm, t_R = 58.0 min (minor), t_R = 85.9 min (major), 93:7 er; t_R = 83.1 min (minor), t_R = 99.5 min (major), 95:5 er.

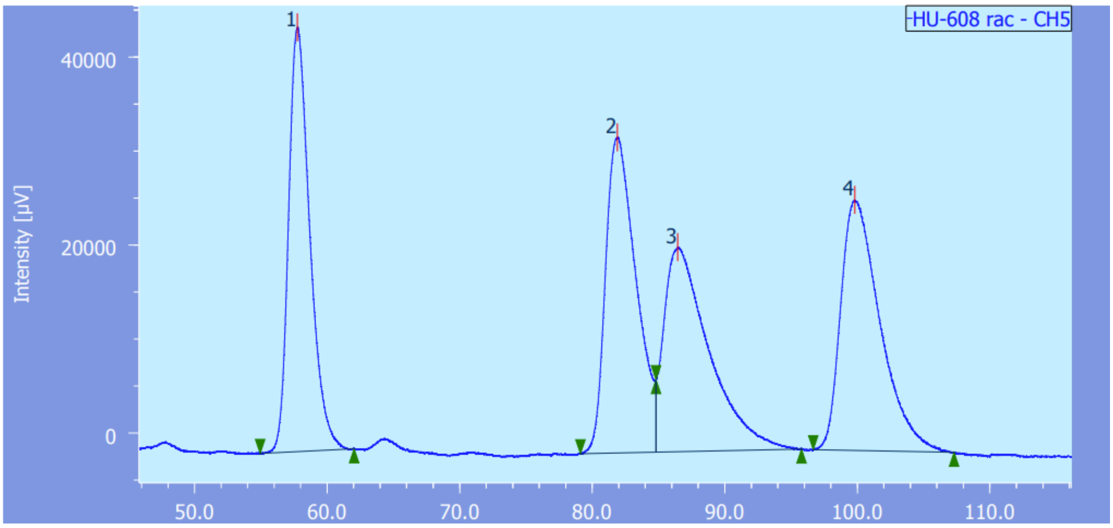

Decision

#	Peak Name	CH	tR [min]	Area [µV·sec]	Height [µV]	Area%	Height%	Quantity	NTP	Resolution	Symmetry Factor	Warning
1	Unknown	5	57.743	5017846	45104	23.866	35.492	N/A	6524	6.744	1.362	
2	Unknown	5	81.880	5383022	33553	25.603	26.402	N/A	5754	0.881	N/A	
3	Unknown	5	86.427	5135575	21759	24.426	17.122	N/A	3286	2.366	N/A	
4	Unknown	5	99.790	5488714	26667	26.105	20.984	N/A	5684	N/A	1.630	

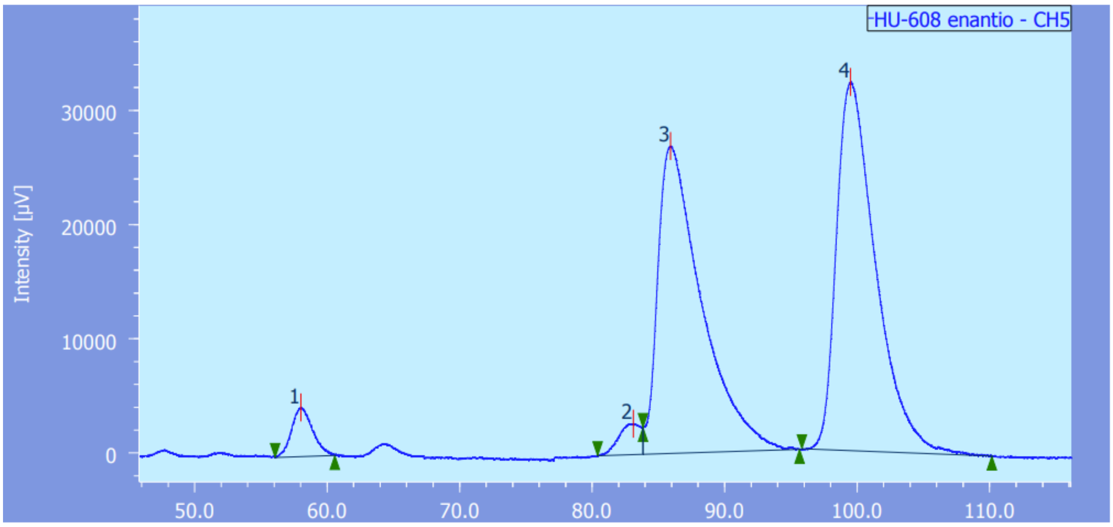

Decision

#	Peak Name	CH	tR [min]	Area [µV·sec]	Height [µV]	Area%	Height%	Quantity	NTP	Resolution	Symmetry Factor	Warning
1	Unknown	5	58.010	475436	4308	3.489	6.506	N/A	6447	N/A	1.155	
2	Unknown	5	83.100	320978	2713	2.355	4.097	N/A	N/A	N/A	N/A	
3	Unknown	5	85.897	6172603	26912	45.294	40.644	N/A	3698	2.497	N/A	
4	Unknown	5	99.477	6658839	32282	48.862	48.753	N/A	5732	N/A	1.667	

***tert*-Butyl (*R*)-2-(4-cyanophenyl)-4-ethyl-4-hydroxyhexanoate (59)**

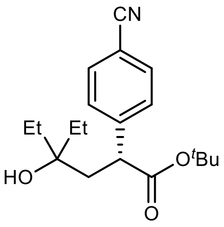

Prepared by **GP-C**. White solid, 51.3 mg, 81% yield. m.p. = 100-101 °C; ^{1}H NMR (400 MHz, $CDCl_3$) δ 7.63 – 7.56 (m, 2H), 7.44 – 7.38 (m, 2H), 3.75 (dd, J = 9.9, 3.1 Hz, 1H), 2.43 (dd, J = 14.5, 9.9 Hz, 1H), 1.63 (dd, J = 14.5, 3.1 Hz, 1H), 1.54 – 1.45 (m, 4H), 1.36 (s, 9H), 0.87 (t, J = 7.5 Hz, 3H), 0.81 (t, J = 7.5 Hz, 3H); ^{13}C NMR (101 MHz, $CDCl_3$) δ 173.0, 146.5, 132.4, 128.5, 118.7, 110.8, 81.4, 74.4, 47.9, 41.9, 31.6, 30.0, 27.8, 8.1, 7.6. IR (film): ν

(cm^{-1}) 3532, 2969, 2932, 2880, 2227, 1719, 1604, 1456, 1365, 1344, 1243, 1233, 1149, 1143, 979, 879, 842, 562, 481; HR-MS (ESI) m/z calcd for $C_{19}H_{27}NNaO_3^+$ [M+Na$^+$] 340.18831, found 340.18826; $[\alpha]_D^{24.0}$ = -21.2 (c = 0.1, $CHCl_3$); HPLC conditions: IC column, hexane/2-propanol = 95/5, flow rate = 0.5 mL/min, λ = 230 nm, t_R = 22.2 min (minor), t_R = 23.6 min (major), 95:5 er.

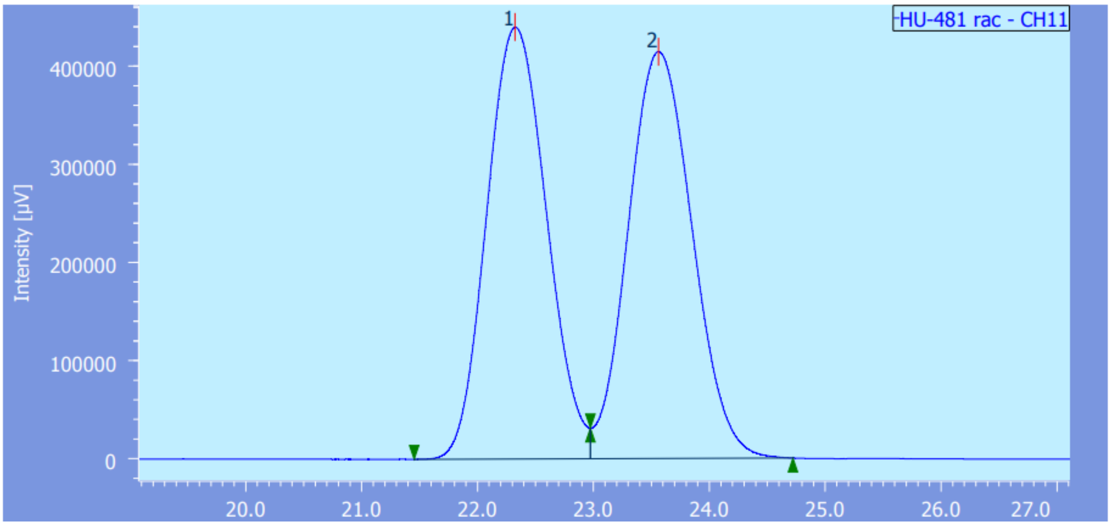

Decision

#	Peak Name	CH	tR [min]	Area [µV·sec]	Height [µV]	Area%	Height%	Quantity	NTP	Resolution	Symmetry Factor	Warning
1	Unknown	11	22.323	15796131	439770	49.722	51.489	N/A	8478	1.238	N/A	
2	Unknown	11	23.560	15972909	414326	50.278	48.511	N/A	8328	N/A	N/A	

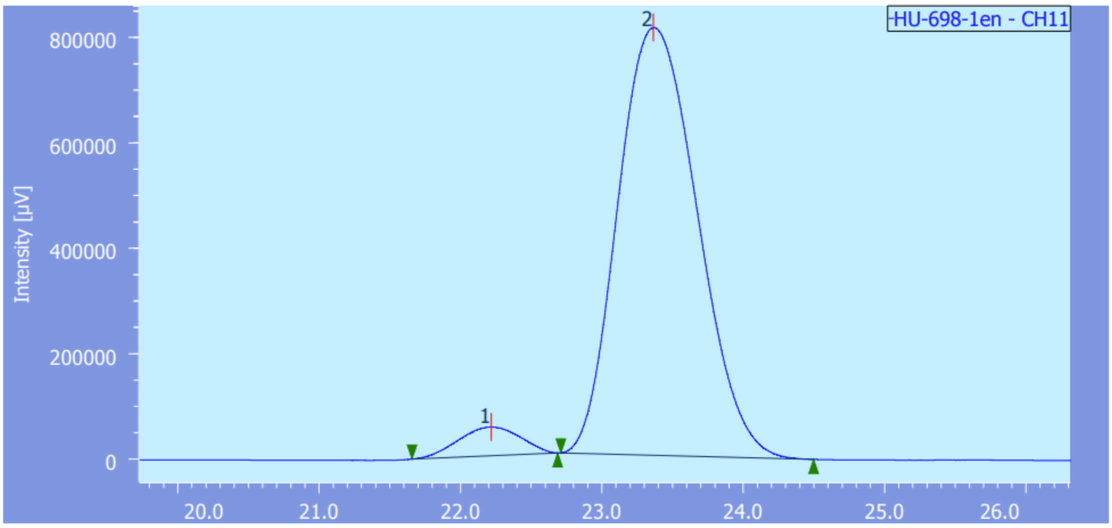

Decision

#	Peak Name	CH	tR [min]	Area [µV·sec]	Height [µV]	Area%	Height%	Quantity	NTP	Resolution	Symmetry Factor	Warning
1	Unknown	11	22.217	1675693	54454	5.021	6.293	N/A	10624	1.194	0.934	
2	Unknown	11	23.363	31697319	810848	94.979	93.707	N/A	7713	N/A	1.188	

***tert*-Butyl (*R*)-2-(4-cyanophenyl)-3-(1-methoxycyclopentyl)propanoate (60)**

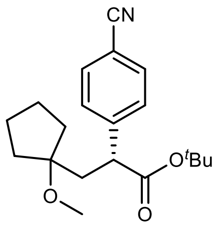

Prepared by **GP-C**. White solid, 36.2 mg, 55% yield. m.p. = 66-67 ºC; ^{1}H NMR (400 MHz, $CDCl_3$) δ 7.62 – 7.57 (m, 2H), 7.46 – 7.41 (m, 2H), 3.73 (dd, *J* = 8.0, 4.3 Hz, 1H), 3.02 (s, 3H), 2.55 (dd, *J* = 14.7, 8.1 Hz, 1H), 1.87 – 1.78 (m, 2H), 1.77 – 1.62 (m, 3H), 1.61 – 1.52 (m, 2H), 1.45 – 1.42 (m, 1H), 1.36 (s, 9H), 1.27 – 1.21 (m, 1H); ^{13}C NMR (101 MHz, $CDCl_3$) δ 172.4, 146.6, 132.3, 128.7, 118.8, 110.8, 86.2, 81.1, 49.3, 48.7, 38.7, 35.7, 35.7, 27.8, 23.31, 23.29. IR (film): ν (cm^{-1}) 2967, 2934, 2872, 2826, 2226, 1719, 1604, 1502, 1456, 1367, 1357, 1223,

1142, 1075, 1019, 857, 839, 760, 562; HR-MS (ESI) m/z calcd for $C_{20}H_{27}NNaO_3^+$ [M+Na$^+$] 352.18831, found 352.18839; $[\alpha]_D^{24.1}$ = -27.2 (c = 0.1, $CHCl_3$); HPLC conditions: Chiral-NR column, hexane/2-propanol = 98/2, flow rate = 1.0 mL/min, λ = 240 nm, t_R = 14.3 min (minor), t_R = 16.8 min (major), 95:5 er.

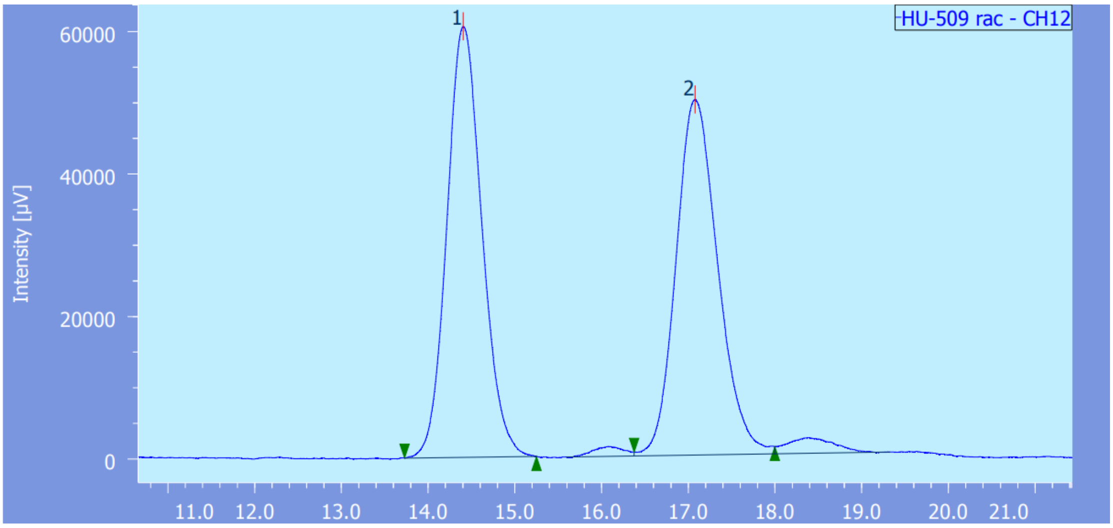

Decision

#	Peak Name	CH	tR [min]	Area [µV·sec]	Height [µV]	Area%	Height%	Quantity	NTP	Resolution	Symmetry Factor	Warning
1	Unknown	12	14.403	1699636	60454	50.381	54.800	N/A	6174	3.340	1.126	
2	Unknown	12	17.077	1673902	49863	49.619	45.200	N/A	6139	N/A	1.173	

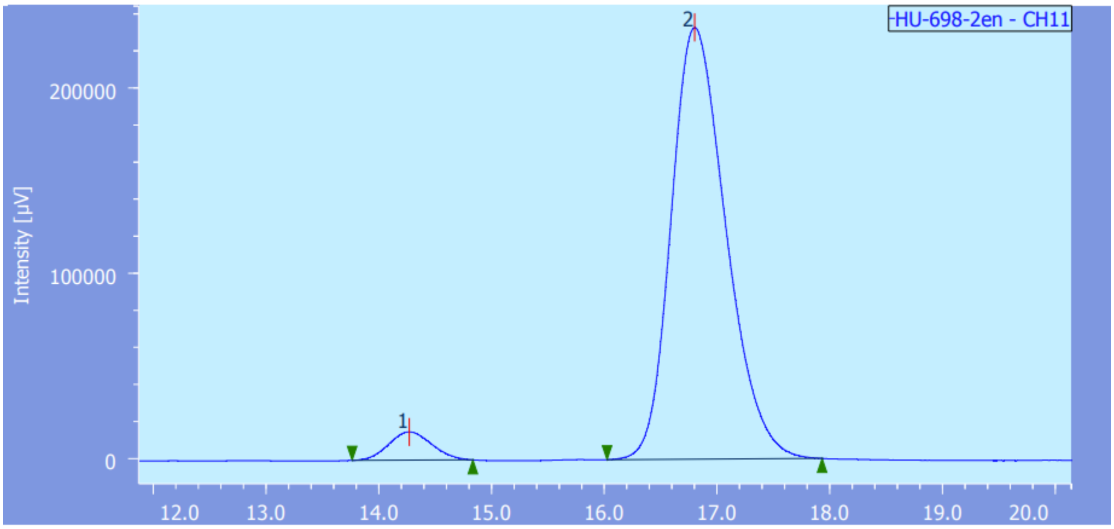

Decision

#	Peak Name	CH	tR [min]	Area [µV·sec]	Height [µV]	Area%	Height%	Quantity	NTP	Resolution	Symmetry Factor	Warning
1	Unknown	11	14.270	407352	15249	4.921	6.146	N/A	6338	3.166	1.084	
2	Unknown	11	16.800	7869866	232874	95.079	93.854	N/A	5759	N/A	1.263	

2-Methyl-4-phenylbutan-2-yl (*R*)-2-(4-cyanophenyl)-3-cyclohexylpropanoate (61)

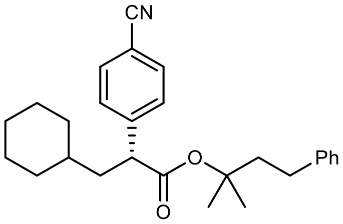

Prepared by **GP-A**. Colorless oil, 58.0 mg, 72% yield. ^{1}H NMR (400 MHz, $CDCl_3$) δ 7.63 – 7.55 (m, 2H), 7.45 – 7.38 (m, 2H), 7.26 – 7.21 (m, 2H), 7.18 – 7.13 (m, 1H), 7.09 – 7.03 (m, 2H), 3.64 (t, *J* = 7.8 Hz, 1H), 2.52 – 2.37 (m, 2H), 2.02 – 1.90 (m, 3H), 1.74 – 1.57 (m, 6H), 1.42 (s, 3H), 1.41 (s, 3H), 1.18 – 1.07 (m, 4H), 0.97 – 0.84 (m, 2H); ^{13}C NMR (101 MHz, $CDCl_3$) δ 172.2, 145.4, 141.7, 132.3, 128.7, 128.4, 128.2, 125.8, 118.7, 110.9, 82.9, 50.0, 42.6, 40.7, 35.4, 33.2, 33.0,

30.1, 26.3, 26.04, 26.02, 25.9, 25.8. IR (film): ν (cm^{-1}) 2975, 2922, 2852, 2228, 1725, 1605, 1498, 1449, 1384, 1368, 1273, 1248, 1203, 1179, 1160, 1154, 1118, 1074, 1021, 968, 848, 838, 765, 744, 698, 576, 552; HR-MS (ESI) m/z calcd for $C_{27}H_{33}NNaO_2^+$ [M+Na$^+$] 426.24035, found 426.24032; $[\alpha]_D^{24.3}$ = -28.8 (c = 0.1, $CHCl_3$); HPLC conditions: Chiral-NR column, hexane/2-propanol = 98/2, flow rate = 1.0 mL/min, λ = 230 nm, t_R = 22.9 min (minor), t_R = 27.3 min (major), 94:6 er.

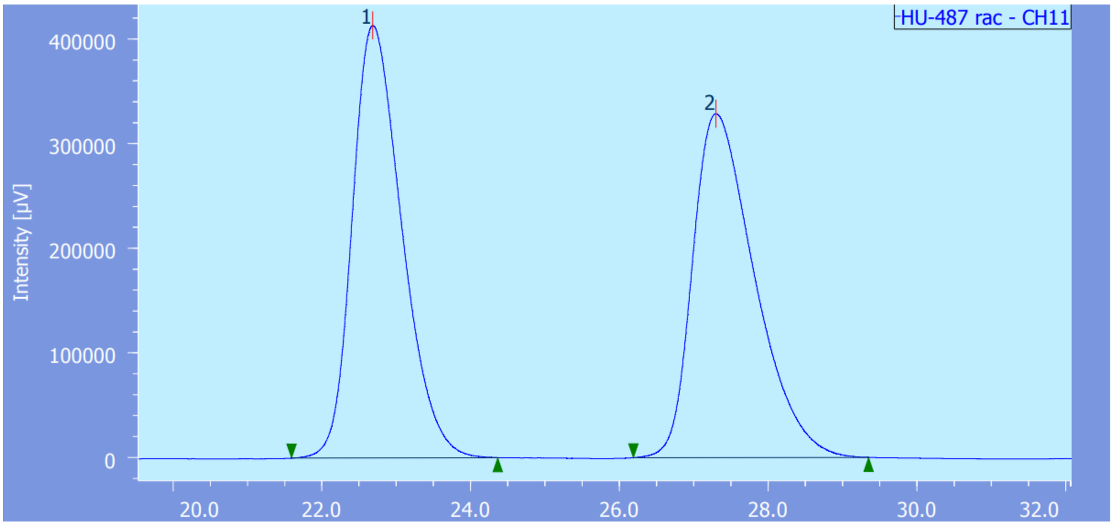

Decision

#	Peak Name	CH	tR [min]	Area [μV·sec]	Height [μV]	Area%	Height%	Quantity	NTP	Resolution	Symmetry Factor	Warning
1	Unknown	11	22.683	19144760	413241	50.144	55.704	N/A	5617	3.399	1.303	
2	Unknown	11	27.297	19034496	328604	49.856	44.296	N/A	5219	N/A	1.493	

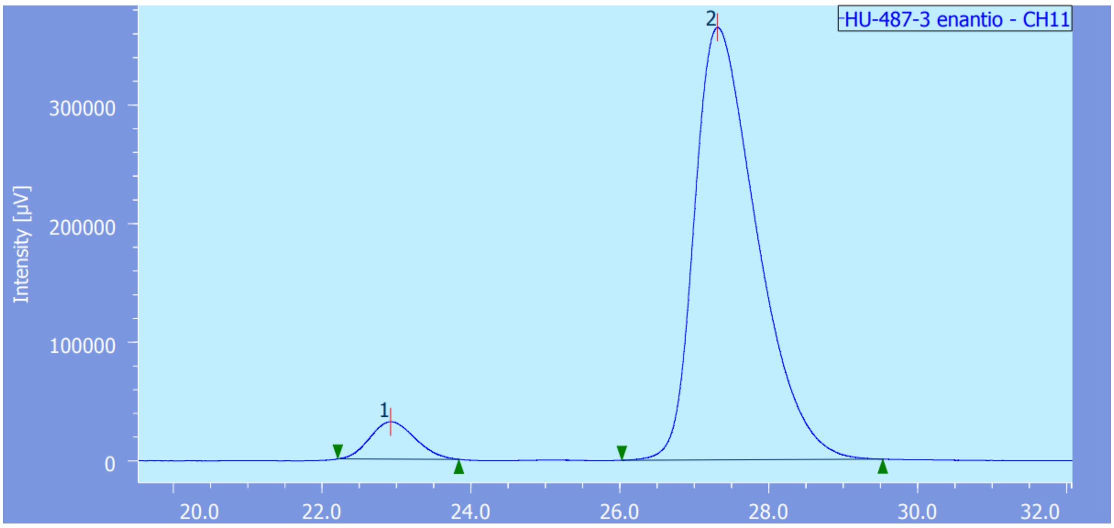

Decision

#	Peak Name	CH	tR [min]	Area [μV·sec]	Height [μV]	Area%	Height%	Quantity	NTP	Resolution	Symmetry Factor	Warning
1	Unknown	11	22.917	1341953	31574	5.944	7.972	N/A	6436	3.312	1.139	
2	Unknown	11	27.310	21234055	364490	94.056	92.028	N/A	5181	N/A	1.527	

2,3,3-Trimethylbutan-2-yl (*R*)-2-(4-cyanophenyl)-3-cyclohexylpropanoate (62)

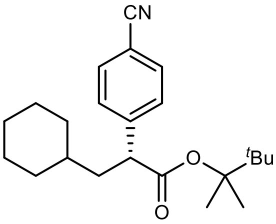

Prepared by **GP-A**. Colorless oil, 51.2 mg, 72% yield. ^{1}H NMR (400 MHz, $CDCl_3$) δ 7.64 – 7.56 (m, 2H), 7.44 – 7.35 (m, 2H), 3.65 (t, *J* = 7.8 Hz, 1H), 1.97 (ddd, *J* = 13.9, 7.9, 7.4 Hz, 1H), 1.76 – 1.57 (m, 6H), 1.45 (s, 3H), 1.37 (s, 3H), 1.19 – 1.08 (m, 4H), 0.98 – 0.89 (m, 2H), 0.86 (s, 9H); ^{13}C NMR (101 MHz, $CDCl_3$) δ 172.2, 145.6, 132.3, 128.8, 118.8, 110.8, 88.0, 50.4, 40.4, 38.3, 35.3, 33.2, 33.0, 26.4, 26.1, 25.0,

20.4, 20.2. IR (film): ν (cm^{-1}) 2978, 2922, 2848, 2229, 1717, 1606, 1505, 1464, 1447, 1377, 1368, 1275, 1261, 1212, 1178, 1169, 1124, 938, 847, 835, 784, 578, 552; HR-MS (ESI) m/z calcd for $C_{23}H_{33}NNaO_2^+$ [M+Na$^+$] 378.24035, found 378.24056; $[\alpha]_D^{24.2}$ = -31.0 (c = 0.1, $CHCl_3$); HPLC conditions: Chiral-NR column, hexane/2-propanol = 98/2, flow rate = 1.0 mL/min, λ = 230 nm, t_R = 15.2 min (minor), t_R = 18.4 min (major), 96:4 er.

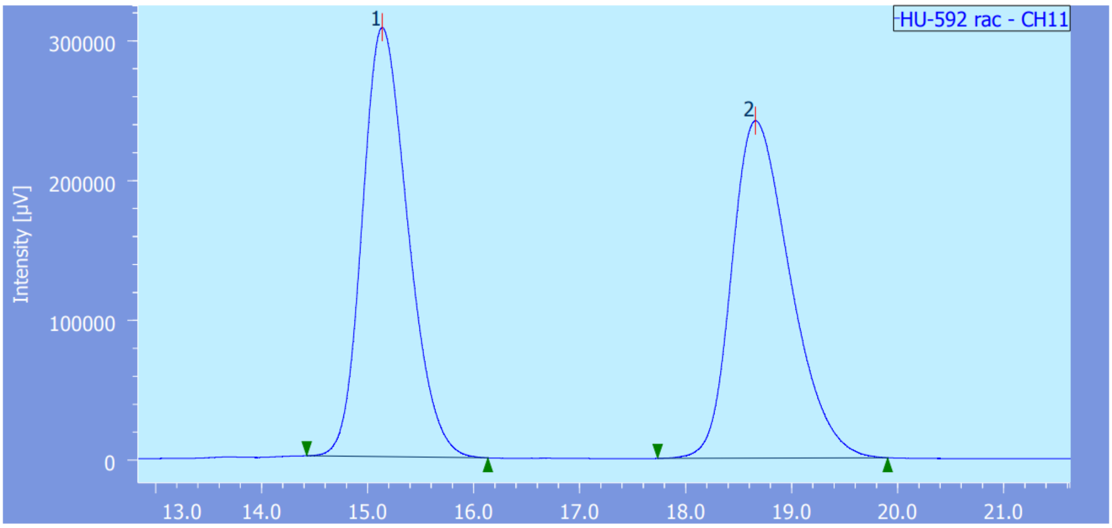

Decision

#	Peak Name	CH	tR [min]	Area [µV·sec]	Height [µV]	Area%	Height%	Quantity	NTP	Resolution	Symmetry Factor	Warning
1	Unknown	11	15.137	9198695	307012	49.791	55.997	N/A	5935	3.937	1.223	
2	Unknown	11	18.657	9275758	241257	50.209	44.003	N/A	5493	N/A	1.343	

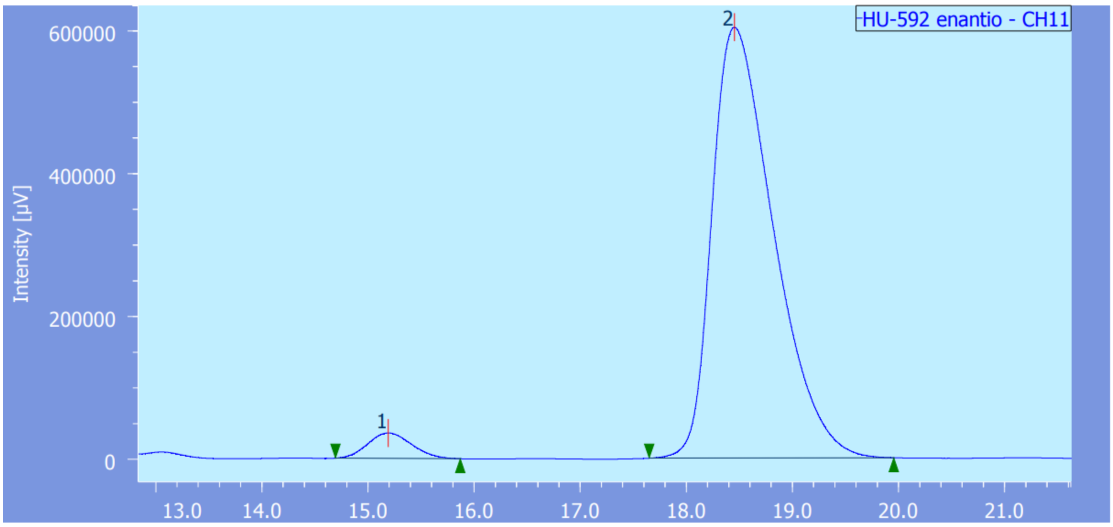

Decision

#	Peak Name	CH	tR [min]	Area [µV·sec]	Height [µV]	Area%	Height%	Quantity	NTP	Resolution	Symmetry Factor	Warning
1	Unknown	11	15.190	1032599	35687	4.076	5.589	N/A	6133	3.572	1.126	
2	Unknown	11	18.453	24300440	602797	95.924	94.411	N/A	4886	N/A	1.553	

1-Methylcyclohexyl (*R*)-2-(4-cyanophenyl)-3-cyclohexylpropanoate (63)

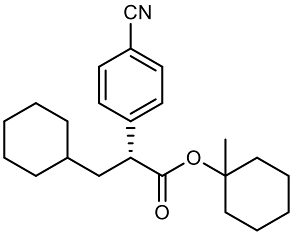

Prepared by **GP-A**. White solid, 45.4 mg, 64% yield. m.p. = 62-63 °C; ^{1}H NMR (400 MHz, $CDCl_3$) δ 7.64 – 7.56 (m, 2H), 7.46 – 7.39 (m, 2H), 3.67 (t, *J* = 7.8 Hz, 1H), 2.12 – 2.03 (m, 2H), 1.97 (ddd, *J* = 13.8, 8.1, 7.2 Hz, 1H), 1.76 – 1.56 (m, 6H), 1.46 – 1.40 (m, 2H), 1.38 (s, 3H), 1.36 – 1.23 (m, 4H), 1.21 – 1.08 (m, 6H), 0.98 – 0.84 (m, 2H); ^{13}C NMR (101 MHz, $CDCl_3$) δ 172.1, 145.5, 132.2, 128.8, 118.8, 110.8,

82.7, 50.2, 40.5, 36.8, 36.2, 35.3, 33.2, 33.0, 26.4, 26.04 (two peaks), 25.4, 25.1, 21.9, 21.8. IR (film): ν (cm^{-1}) 2971, 2918, 2850, 2232, 1712, 1607, 1504, 1447, 1367, 1359, 1274, 1216, 1187, 1144, 1026, 960, 930, 895, 865, 835, 805, 746, 576, 551, 490; HR-MS (ESI) m/z calcd for $C_{23}H_{31}NNaO_2^+$ [M+Na$^+$] 376.22470, found 376.22492; $[\alpha]_D^{24.2}$ = -23.8 (c = 0.1, $CHCl_3$); HPLC conditions: Chiral-NR column, hexane/2-propanol = 98/2, flow rate = 1.0 mL/min, λ = 230 nm, t_R = 16.3 min (minor), t_R = 18.5 min (major), 96:4 er.

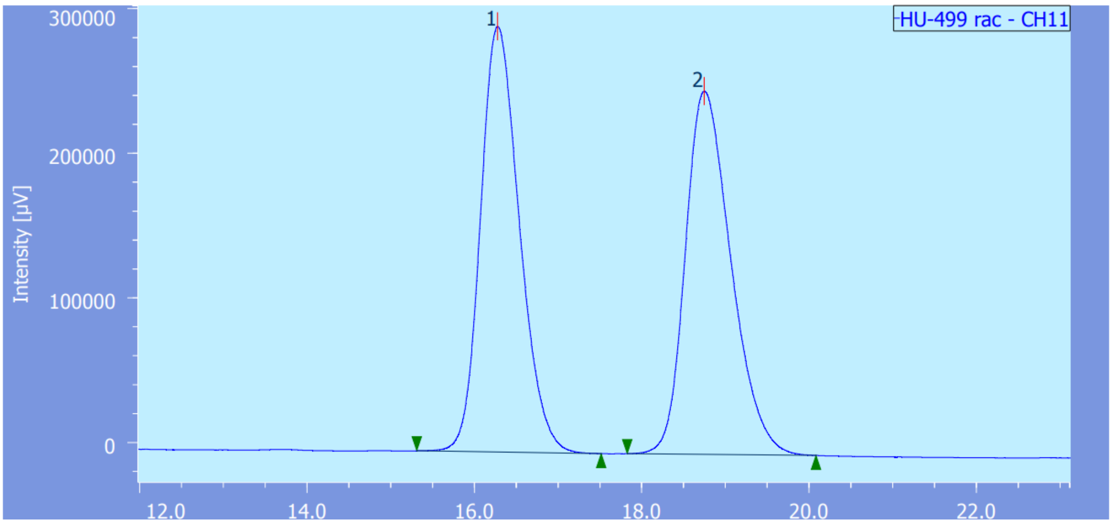

Decision

#	Peak Name	CH	tR [min]	Area [µV·sec]	Height [µV]	Area%	Height%	Quantity	NTP	Resolution	Symmetry Factor	Warning
1	Unknown	11	16.277	9518707	294157	49.977	53.948	N/A	5927	2.697	1.207	
2	Unknown	11	18.747	9527633	251099	50.023	46.052	N/A	5727	N/A	1.299	

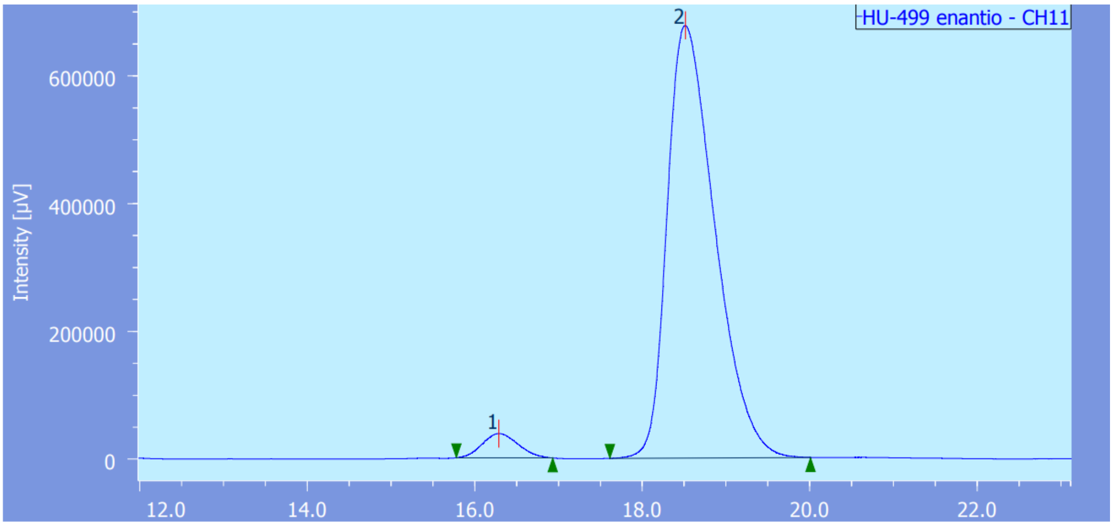

Decision

#	Peak Name	CH	tR [min]	Area [µV·sec]	Height [µV]	Area%	Height%	Quantity	NTP	Resolution	Symmetry Factor	Warning
1	Unknown	11	16.287	1140903	38071	4.075	5.325	N/A	6539	2.418	1.122	
2	Unknown	11	18.513	26857467	676873	95.925	94.675	N/A	5063	N/A	1.473	

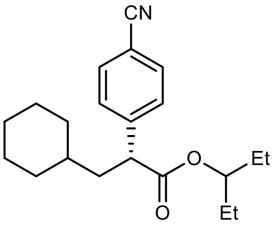

Pentan-3-yl (*R*)-2-(4-cyanophenyl)-3-cyclohexylpropanoate (64)

Prepared by **GP-B**. Colorless oil, 46.1 mg, 70% yield. ^{1}H NMR (400 MHz, $CDCl_3$) δ 7.64 – 7.55 (m, 2H), 7.48 – 7.40 (m, 2H), 4.76 – 4.66 (m, 1H), 3.74 (dd, *J* = 8.3, 7.3 Hz, 1H), 2.00 (ddd, *J* = 13.9, 8.5, 6.8 Hz, 1H), 1.76 – 1.61 (m, 6H), 1.57 – 1.50 (m, 2H), 1.50 – 1.38 (m, 2H), 1.21 – 1.07 (m, 4H), 0.98 – 0.86 (m, 2H), 0.83 (t, *J* = 7.5 Hz, 3H), 0.66 (t, *J*

= 7.4 Hz, 3H); ^{13}C NMR (101 MHz, $CDCl_3$) δ 173.0, 145.2, 132.3, 128.8, 118.8, 111.0, 49.4, 40.8, 35.4, 33.1, 33.1, 26.4, 26.4, 26.3, 26.0, 9.5, 9.3. IR (film): ν (cm^{-1}) 2968, 2924, 2851, 2228, 1727, 1608, 1503, 1461, 1449, 1384, 1273, 1261, 1215, 1165, 1113, 1102, 1034, 974, 921, 891, 848, 837, 578, 552, 504; HR-MS (ESI) m/z calcd for $C_{21}H_{29}NNaO_2^+$ [M+Na$^+$] 350.20905, found 350.20859; $[\alpha]_D^{24.1}$ = -32.7 (c = 0.1, $CHCl_3$); HPLC conditions: Chiral-NR column, hexane/2-propanol = 98/2, flow rate = 1.0 mL/min, λ = 230 nm, t_R = 17.9 min (minor), t_R = 22.5 min (major), 95:5 er.

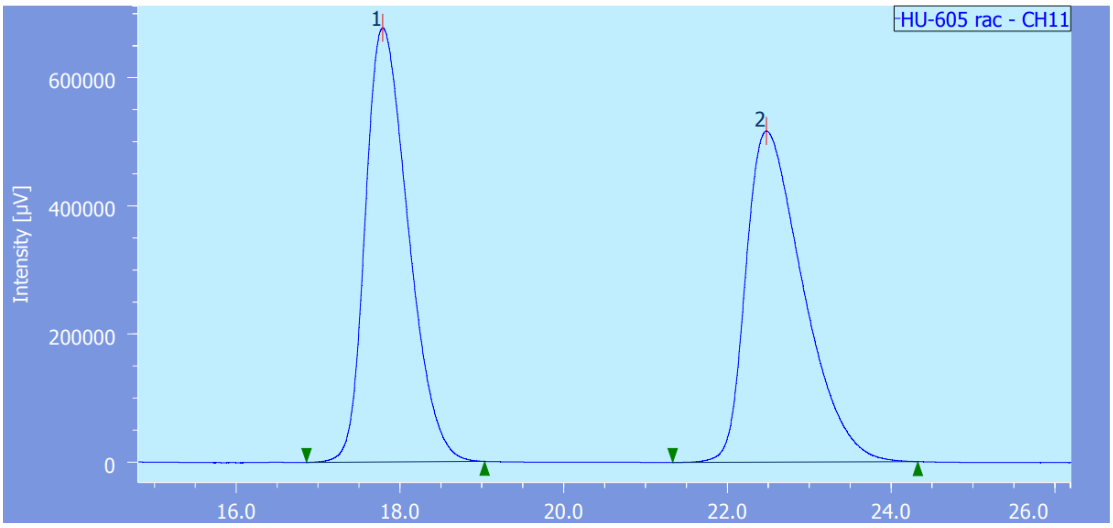

Decision

#	Peak Name	CH	tR [min]	Area [µV·sec]	Height [µV]	Area%	Height%	Quantity	NTP	Resolution	Symmetry Factor	Warning
1	Unknown	11	17.790	24691685	677372	49.590	56.741	N/A	5480	4.203	1.340	
2	Unknown	11	22.473	25100132	516430	50.410	43.259	N/A	4986	N/A	1.601	

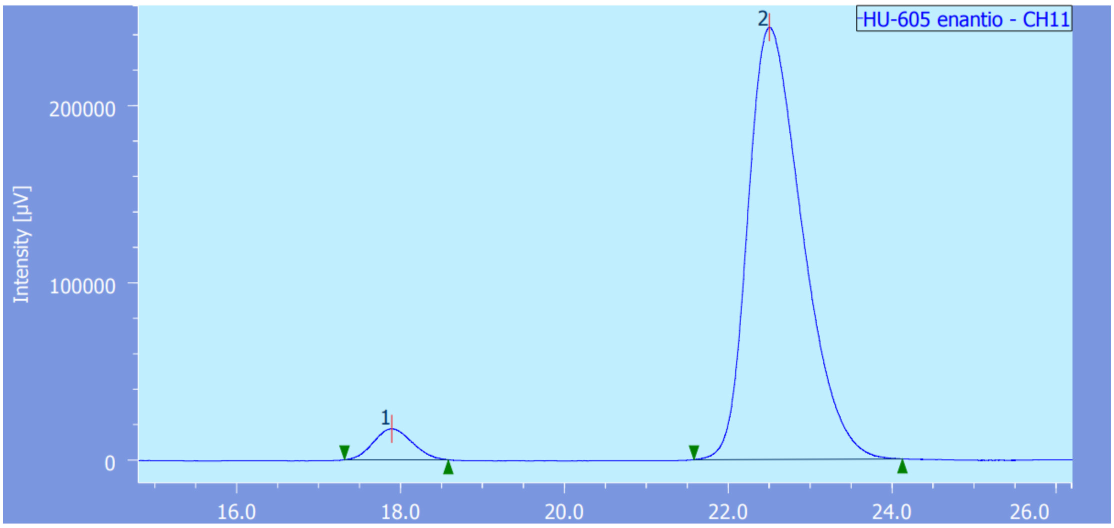

Decision

#	Peak Name	CH	tR [min]	Area [µV·sec]	Height [µV]	Area%	Height%	Quantity	NTP	Resolution	Symmetry Factor	Warning
1	Unknown	11	17.893	579837	17506	4.906	6.702	N/A	6442	4.393	1.095	
2	Unknown	11	22.500	11239798	243703	95.094	93.298	N/A	5523	N/A	1.398	

2,4-Dimethylpentan-3-yl (*R*)-2-(4-cyanophenyl)-3-cyclohexylpropanoate (65)

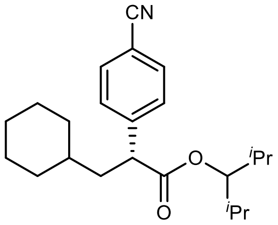

Prepared by **GP-A**. White solid, 50.6 mg, 71% yield. m.p. = 77-78 °C; ^{1}H NMR (400 MHz, $CDCl_3$) δ 7.63 – 7.56 (m, 2H), 7.49 – 7.40 (m, 2H), 4.53 (t, J = 6.1 Hz, 1H), 3.77 (t, J = 7.8 Hz, 1H), 2.07 – 1.96 (m, 1H), 1.88 – 1.76 (m, 2H), 1.75 – 1.61 (m, 5H), 1.19 – 1.07 (m, 4H), 0.96 – 0.84 (m, 3H), 0.82 (d, J = 6.8 Hz, 3H), 0.78 (d, J = 6.7 Hz, 3H), 0.65 (d,

J = 6.8 Hz, 3H), 0.62 (d, J = 6.7 Hz, 3H); ^{13}C NMR (101 MHz, $CDCl_3$) δ 173.0, 145.2, 132.3, 129.0, 118.8, 111.0, 83.3, 49.4, 40.5, 35.2, 33.09, 33.08, 29.4, 29.3, 26.4, 26.0, 19.45, 19.37, 17.2, 16.8. IR (film): ν (cm^{-1}) 2963, 2919, 2851, 2227, 1720, 1604, 1501, 1467, 1450, 1443, 1387, 1370, 1342, 1293, 1261, 1217, 1182, 1170, 1128, 1096, 976, 938, 902, 891, 848, 837, 780, 737, 690, 574, 549, 487; HR-MS (ESI) m/z calcd for $C_{23}H_{33}NNaO_2^+$ [M+Na$^+$] 378.24035, found 378.24027; $[\alpha]_D^{24.1}$ = -29.7 (c = 0.1, $CHCl_3$); HPLC conditions: Chiral-NR column, hexane/2-propanol = 99.5/0.5, flow rate = 0.5 mL/min, λ = 230 nm, t_R = 47.9 min (minor), t_R = 50.5 min (major), 96:4 er.

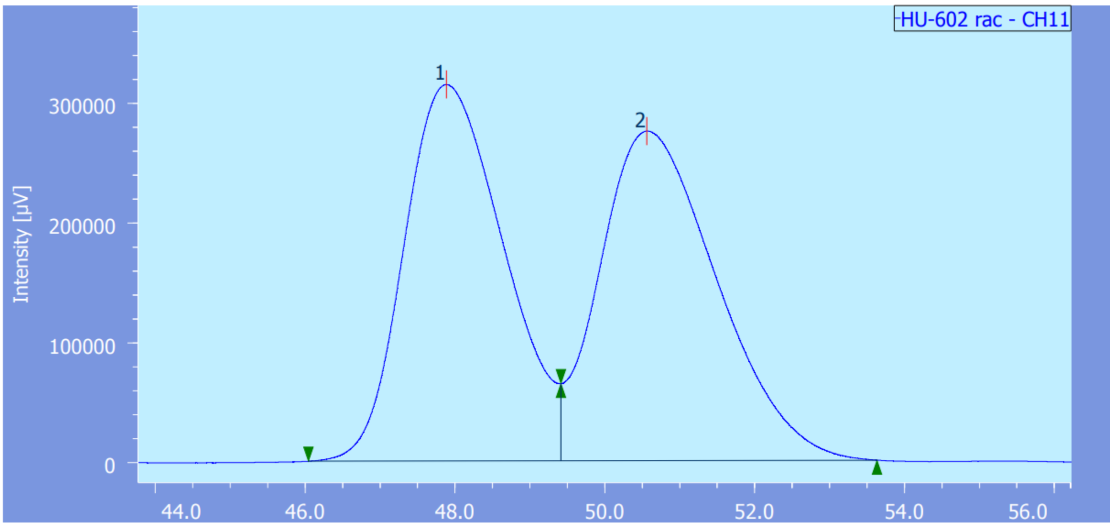

Decision

#	Peak Name	CH	tR [min]	Area [µV·sec]	Height [µV]	Area%	Height%	Quantity	NTP	Resolution	Symmetry Factor	Warning
1	Unknown	11	47.883	29088523	314222	49.276	53.327	N/A	5789	0.987	N/A	
2	Unknown	11	50.557	29943187	275014	50.724	46.673	N/A	4811	N/A	N/A	

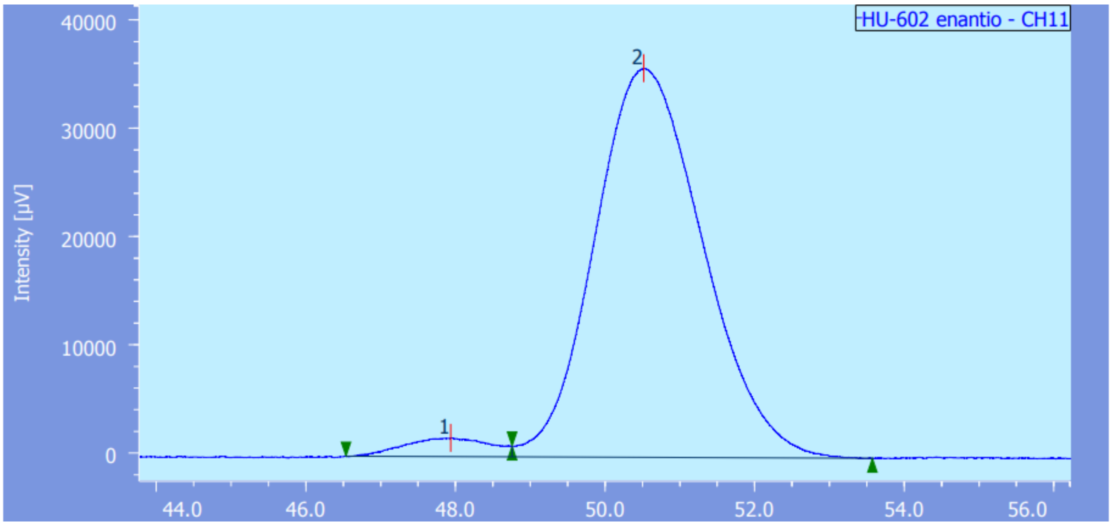

Decision

#	Peak Name	CH	tR [min]	Area [µV·sec]	Height [µV]	Area%	Height%	Quantity	NTP	Resolution	Symmetry Factor	Warning
1	Unknown	11	47.933	139436	1731	3.712	4.598	N/A	N/A	N/A	N/A	
2	Unknown	11	50.517	3616813	35916	96.288	95.402	N/A	5714	N/A	1.139	

Methyl (*R*)-2-(4-cyanophenyl)-3-cyclohexylpropanoate (66)

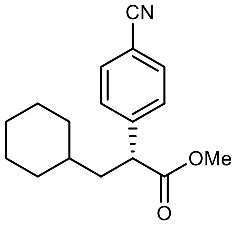

Prepared by **GP-B**. White solid, 42.5 mg, 78% yield. m.p. = 47-48 ºC; ^{1}H NMR (400 MHz, $CDCl_3$) δ 7.63 – 7.58 (m, 2H), 7.44 – 7.39 (m, 2H), 3.74 (t, *J* = 7.8 Hz, 1H), 3.66 (s, 3H), 1.97 (ddd, *J* = 13.8, 7.8, 7.4 Hz, 1H), 1.72 – 1.60 (m, 6H), 1.20 – 1.04 (m, 4H), 0.96 – 0.84 (m, 2H); ^{13}C NMR (101 MHz, $CDCl_3$) δ 173.6, 144.7, 132.4, 128.8, 118.7, 111.1, 52.2, 48.8, 40.9, 35.2, 33.2, 32.8, 26.3, 26.00, 25.95. IR (film): ν (cm^{-1}) 2917, 2846, 2230, 1730, 1604, 1503, 1449, 1435, 1349, 1278, 1215, 1188, 1156, 1129, 1088, 983, 847, 836, 827, 780, 739, 693, 576, 551; HR-MS (ESI) m/z calcd for $C_{17}H_{21}NNaO_2^+$ [M+Na$^+$] 294.14645, found 294.14632; $[\alpha]_D^{24.1}$ = -55.3 (c = 0.1, $CHCl_3$); HPLC conditions: Chiral-NR column, hexane/2-propanol = 95/5, flow rate = 1.0 mL/min, λ = 230 nm, t_R = 17.5 min (minor), t_R = 22.4 min (major), 92:8 er.

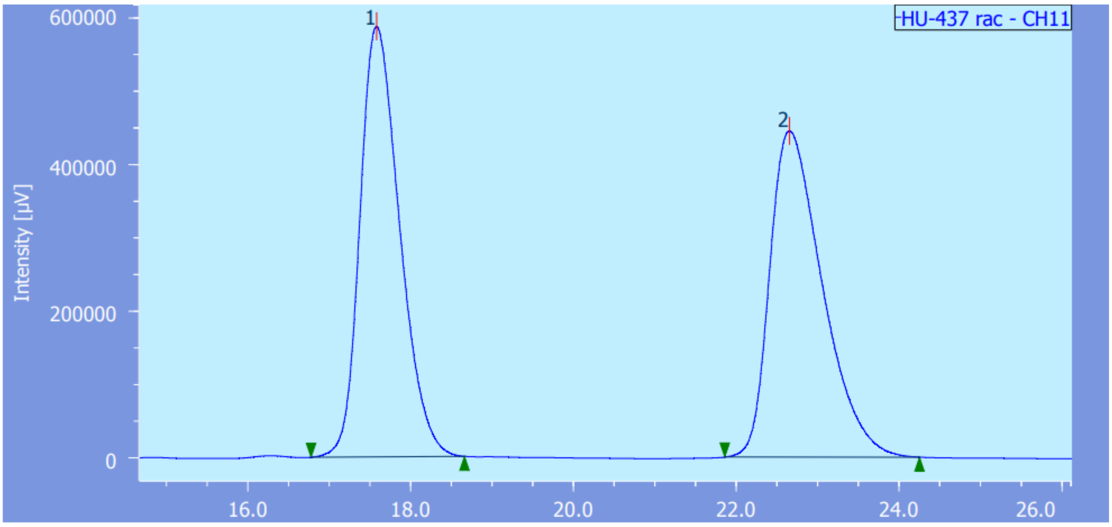

Decision

#	Peak Name	CH	tR [min]	Area [µV·sec]	Height [µV]	Area%	Height%	Quantity	NTP	Resolution	Symmetry Factor	Warning
1	Unknown	11	17.580	19899397	586832	49.879	56.880	N/A	6283	4.933	1.289	
2	Unknown	11	22.650	19995787	444865	50.121	43.120	N/A	5958	N/A	1.517	

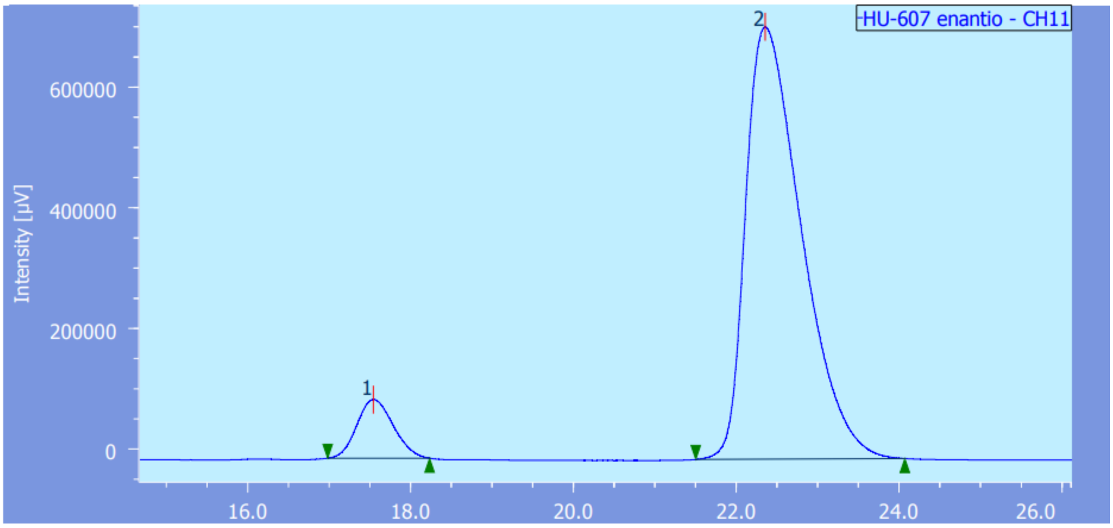

Decision

#	Peak Name	CH	tR [min]	Area [µV·sec]	Height [µV]	Area%	Height%	Quantity	NTP	Resolution	Symmetry Factor	Warning
1	Unknown	11	17.543	3036790	97338	8.185	11.963	N/A	7079	4.631	1.139	
2	Unknown	11	22.353	34066758	716329	91.815	88.037	N/A	5127	N/A	1.642	

Diethyl (*R*)-(1-(4-cyanophenyl)-4-hydroxy-3,3,4-trimethylpentyl)phosphonate (67)

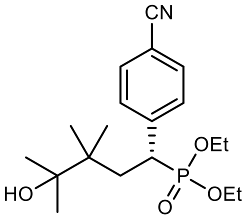

Prepared by **GP-A**. Colorless oil, 31.8 mg, 43% yield. ^{1}H NMR (400 MHz, $CDCl_3$) δ 7.63 – 7.55 (m, 2H), 7.51 – 7.43 (m, 2H), 4.14 – 3.99 (m, 2H), 3.94 – 3.84 (m, 1H), 3.80 – 3.68 (m, 1H), 3.51 (ddd, *J* = 25.3, 9.4, 1.2 Hz, 1H), 2.38 (ddd, *J* = 17.8, 14.7, 1.4 Hz, 1H), 2.05 – 1.94 (m, 1H), 1.63 (br s, 1H), 1.27 (t, *J* = 7.1 Hz, 3H), 1.23 (s, 3H), 1.14 (s, 3H), 1.10 (t, *J* = 7.1 Hz, 3H), 0.85 (s, 3H), 0.60 (s, 3H); ^{13}C NMR (101 MHz, $CDCl_3$) δ 144.9 (d, *J* = 8.1 Hz), 132.0 (d, *J* = 3.1 Hz), 130.3 (d, *J* = 6.9 Hz), 118.8 (d, *J* = 3.3 Hz), 110.6 (d, *J* = 3.8 Hz), 75.48, 62.7 (d, *J* = 7.3 Hz), 62.2 (d, *J* = 7.3 Hz), 41.8 (d, *J* = 97.7 Hz), 41.0 (d, *J* = 24.9 Hz), 37.1 (d, *J* = 3.3 Hz), 25.7, 25.4, 24.2, 21.6, 16.3 (d, *J* = 6.1 Hz), 16.2 (d, *J* = 5.8 Hz); ^{31}P NMR (162 MHz, $CDCl_3$) δ 28.55. IR (film): ν (cm^{-1}) 3412, 2977, 2910, 2876, 2227, 1606, 1504, 1474, 1444, 1391, 1377, 1368, 1237, 1163, 1052, 1020, 960, 949, 858, 787, 749, 578; HR-MS (ESI) m/z calcd for $C_{19}H_{31}NO_4P^{+}$ [M+H^{+}] 368.19852, found 368.19854; $[\alpha]_D^{23.7}$ = 6.2 (c = 0.1, $CHCl_3$); HPLC conditions: IC column, hexane/2-propanol = 70/30, flow rate = 1.0 mL/min, λ = 230 nm, t_R = 12.4 min (minor), t_R = 13.6 min (major), 98:2 er.

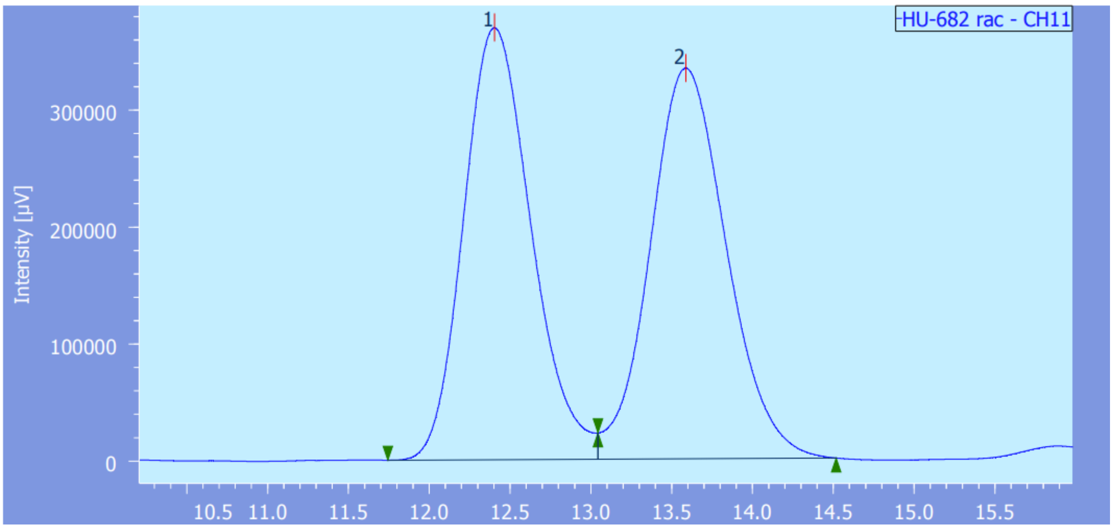

Decision

#	Peak Name	CH	tR [min]	Area [µV·sec]	Height [µV]	Area%	Height%	Quantity	NTP	Resolution	Symmetry Factor	Warning
1	Unknown	11	12.403	10756243	369117	49.523	52.519	N/A	4222	1.465	N/A	
2	Unknown	11	13.587	10963380	333714	50.477	47.481	N/A	4032	N/A	N/A	

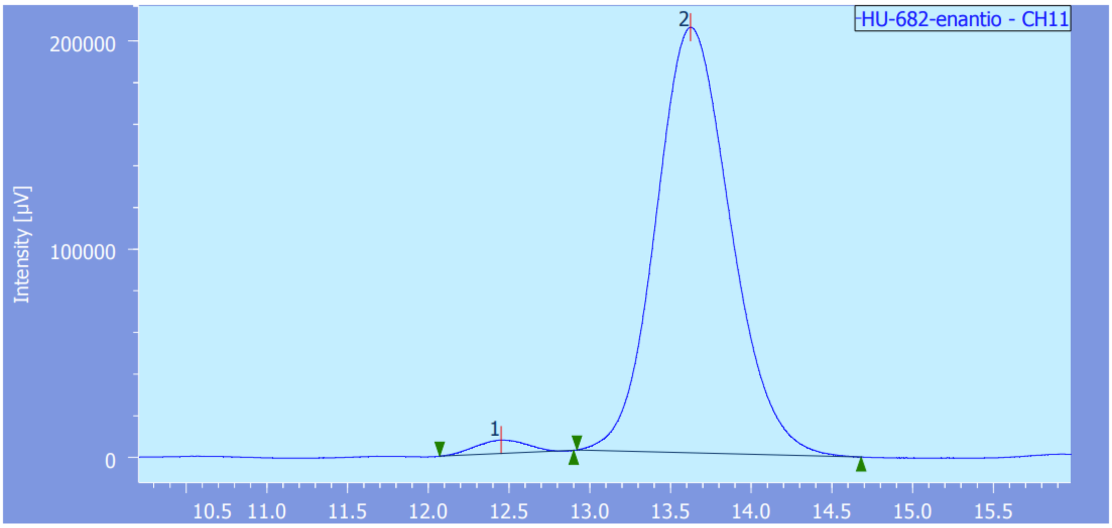

Decision

#	Peak Name	CH	tR [min]	Area [μV·sec]	Height [μV]	Area%	Height%	Quantity	NTP	Resolution	Symmetry Factor	Warning
1	Unknown	11	12.450	145207	6475	2.157	3.073	N/A	6235	1.598	0.977	
2	Unknown	11	13.623	6586974	204251	97.843	96.927	N/A	4190	N/A	1.127	

Diethyl (*R*)-(1-(4-cyanophenyl)-3-hydroxy-3-methylbutyl)phosphonate (68)

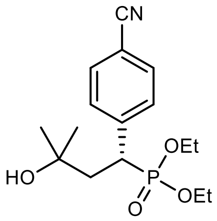

Prepared by **GP-C**. Colorless oil, 38.8 mg, 60% yield. ^{1}H NMR (400 MHz, $CDCl_3$) δ 7.60 (d, *J* = 8.0 Hz, 2H), 7.45 (dd, *J* = 8.4, 2.2 Hz, 2H), 4.12 – 3.83 (m, 4H), 3.45 (ddd, *J* = 24.1, 8.1, 4.2 Hz, 1H), 2.37 (dt, *J* = 15.2, 4.0 Hz, 1 H), 2.24 (br s, 1H), 2.09 (ddd, *J* = 14.7, 11.3, 8.2 Hz, 1H), 1.26 (t, *J* = 7.1 Hz, 3H), 1.21 (s, 3H), 1.17 (t, *J* = 7.1 Hz, 3H), 1.05 (s, 3H); ^{13}C NMR (101 MHz, $CDCl_3$) δ 144.1 (d, *J* = 8.0 Hz), 132.2 (d, *J* = 2.8 Hz), 130.1 (d, *J* = 6.6 Hz), 118.7 (d, *J* = 2.0 Hz), 110.8 (d, *J* = 3.5 Hz), 70.4 (d, *J* = 11.4 Hz), 62.9 (d, *J* = 7.3 Hz), 62.4 (d, *J* = 7.3 Hz), 43.2 (d, *J* = 3.4 Hz), 40.8 (d, *J* = 136.8 Hz), 30.9, 28.8, 16.30 (d, *J* = 11.8 Hz), 16.29; ^{31}P NMR (162 MHz, $CDCl_3$) δ 28.08. IR (film): ν (cm^{-1}) 3411, 2975, 2930, 2909, 2228, 1605, 1505, 1391, 1236, 1155, 1052, 1020, 960, 911, 860, 788, 578; HR-MS (ESI) m/z calcd for $C_{16}H_{25}NO_4P^+$ [M+H$^+$] 326.15157, found 326.15192; $[\alpha]_D^{24.2}$ = -8.2 (c = 0.1, $CHCl_3$); HPLC conditions: IC column, hexane/2-propanol = 70/30, flow rate = 1.0 mL/min, λ = 230 nm, t_R = 14.3 min (minor), t_R = 17.8 min (major), 96:4 er.

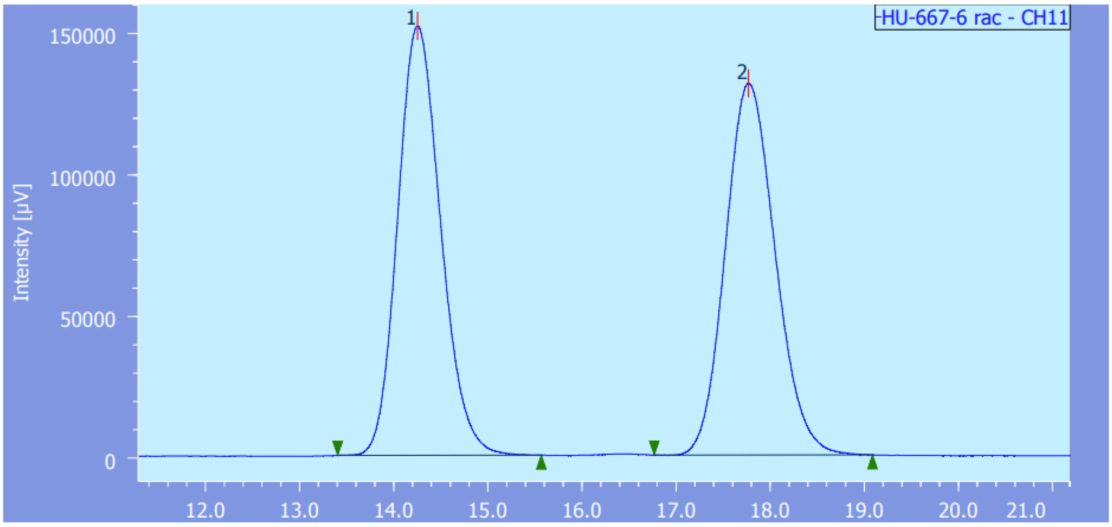

Decision

#	Peak Name	CH	tR [min]	Area [μV·sec]	Height [μV]	Area%	Height%	Quantity	NTP	Resolution	Symmetry Factor	Warning
1	Unknown	11	14.253	4811209	151513	49.828	53.591	N/A	4693	3.904	1.161	
2	Unknown	11	17.767	4844445	131208	50.172	46.409	N/A	5343	N/A	1.140	

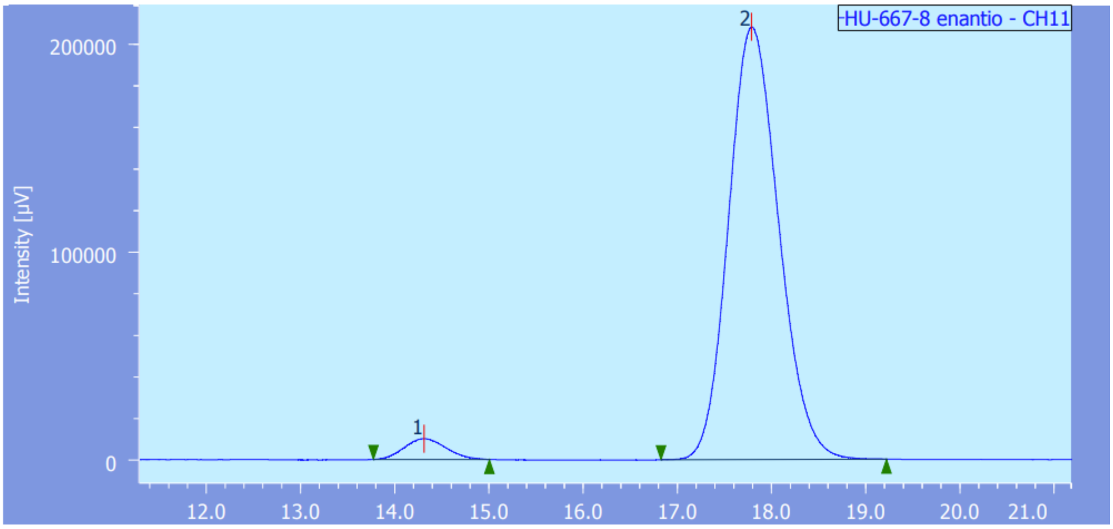

Decision

#	Peak Name	CH	tR [min]	Area [µV·sec]	Height [µV]	Area%	Height%	Quantity	NTP	Resolution	Symmetry Factor	Warning
1	Unknown	11	14.310	310826	10034	3.880	4.604	N/A	4750	3.858	1.115	
2	Unknown	11	17.787	7700649	207893	96.120	95.396	N/A	5308	N/A	1.150	

(*R*)-4-(1-Cyclohexyl-3-oxopentan-2-yl)benzonitrile (69)

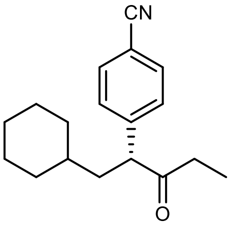

Prepared by **GP-B**. Colorless oil, 38.5 mg, 71% yield. ^{1}H NMR (400 MHz, $CDCl_3$) δ 7.64 – 7.56 (m, 2H), 7.37 – 7.31 (m, 2H), 3.86 (t, *J* = 7.5 Hz, 1H), 2.44 – 2.36 (m, 2H), 1.92 (dt, *J* = 14.4, 7.3 Hz, 1H), 1.73 – 1.54 (m, 6H), 1.16 – 1.06 (m, 3H), 1.05 – 1.00 (m, 1H), 0.97 (t, *J* = 7.2 Hz, 3H), 0.92 – 0.80 (m, 2H); ^{13}C NMR (101 MHz, $CDCl_3$) δ 209.9, 144.9, 132.5, 129.0, 118.6, 111.0, 55.7, 40.0, 35.6, 35.1, 33.6, 32.8, 26.3, 26.02, 25.96, 7.7. IR (film): ν (cm^{-1}) 2976, 2923, 2849, 2227, 1715, 1606, 1504, 1449, 1410, 1351, 1107, 1020, 847, 827, 578, 550; HR-MS (ESI) m/z calcd for $C_{18}H_{23}NNaO^{+}$ [M+Na^{+}] 292.16719, found 292.16692; $[\alpha]_D^{24.1}$ = -140.7 (c = 0.1, $CHCl_3$); HPLC conditions: IC column, hexane/2-propanol = 95/5, flow rate = 1.0 mL/min, λ = 230 nm, t_R = 14.9 min (minor), t_R = 17.1 min (major), 81:19 er.

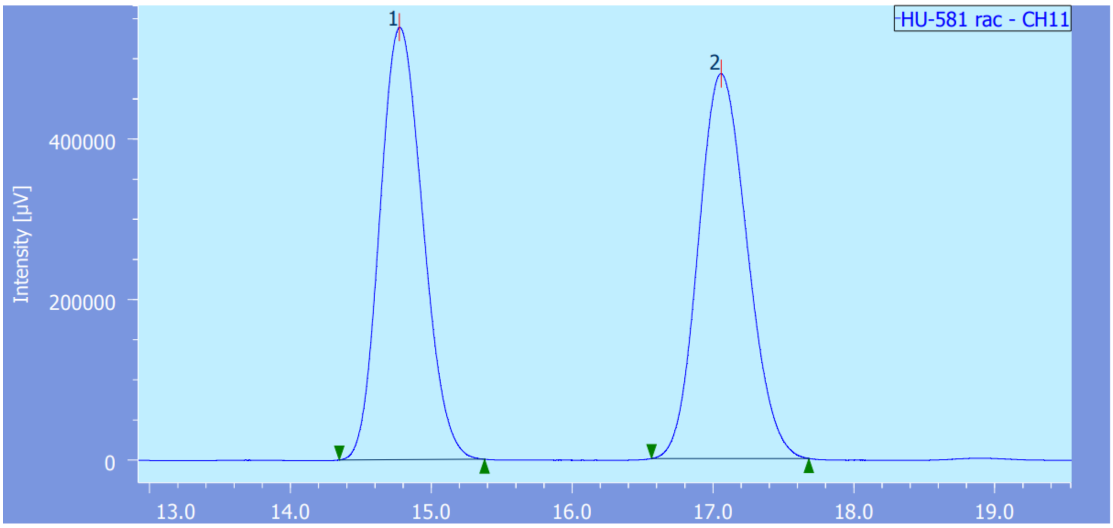

Decision

#	Peak Name	CH	tR [min]	Area [µV·sec]	Height [µV]	Area%	Height%	Quantity	NTP	Resolution	Symmetry Factor	Warning
1	Unknown	11	14.773	11410108	538396	49.666	52.899	N/A	10872	3.785	1.157	
2	Unknown	11	17.057	11563792	479381	50.334	47.101	N/A	11257	N/A	1.126	

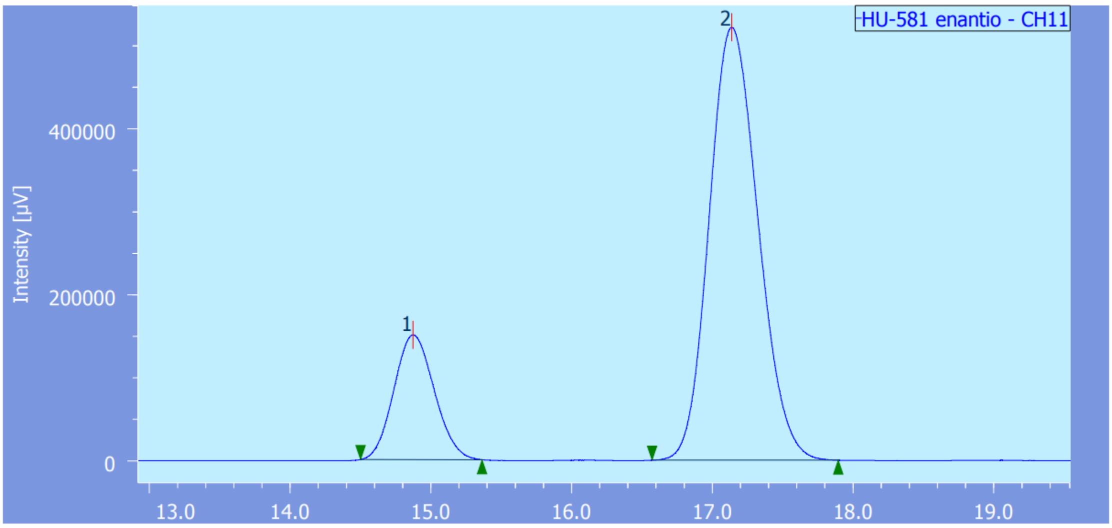

Decision

#	Peak Name	CH	tR [min]	Area [µV·sec]	Height [µV]	Area%	Height%	Quantity	NTP	Resolution	Symmetry Factor	Warning
1	Unknown	11	14.873	2997434	150078	19.214	22.356	N/A	12469	3.862	1.103	
2	Unknown	11	17.137	12602424	521242	80.786	77.644	N/A	11384	N/A	1.143	

(*R*)-*N*-(*tert*-Butyl)-2-(4-cyanophenyl)-3-cyclohexylpropanamide (70)

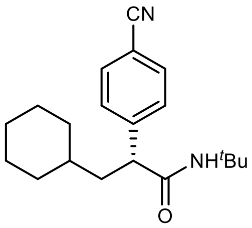

Prepared by **GP-A**. White solid, 38.0 mg, 61% yield. m.p. = 80-81 °C; ^{1}H NMR (400 MHz, $CDCl_3$) δ 7.61 – 7.55 (m, 2H), 7.46 – 7.40 (m, 2H), 5.36 (s, 1H), 3.37 (t, J = 7.7 Hz, 1H), 2.00 – 1.88 (m, 1H), 1.74 – 1.52 (m, 6H), 1.27 (s, 9H), 1.18 – 1.05 (m, 4H), 0.96 – 0.82 (m, 2H); ^{13}C NMR (101 MHz, $CDCl_3$) δ 171.4, 146.4, 132.3, 128.5, 118.8, 110.6, 51.4, 51.2, 41.3, 35.2, 33.3, 32.9, 28.6, 26.3, 26.05, 25.97. IR (film): ν (cm^{-1}) 3335, 2972, 2924, 2903, 2845, 2226, 1641, 1538, 1449, 1362, 1224, 969, 904, 851, 838, 688, 651, 637, 578, 552, 493; HR-MS (ESI) m/z calcd for $C_{20}H_{29}N_2O^+$ [M+H$^+$] 313.22744, found 313.22742; $[\alpha]_D^{24.1}$ = 5.8 (c = 0.1, $CHCl_3$); HPLC conditions: IC column, hexane/2-propanol = 90/10, flow rate = 1.0 mL/min, λ = 240 nm, t_R = 12.8 min (major), t_R = 15.3 min (minor), 63:37 er.

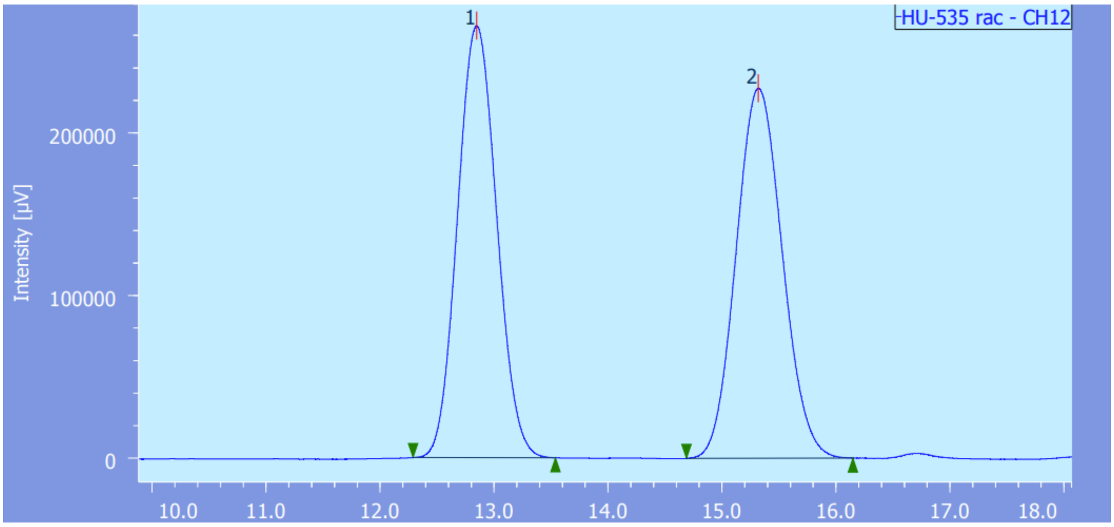

Decision

#	Peak Name	CH	tR [min]	Area [µV·sec]	Height [µV]	Area%	Height%	Quantity	NTP	Resolution	Symmetry Factor	Warning
1	Unknown	12	12.847	6342205	265478	49.719	53.853	N/A	6464	3.565	1.073	
2	Unknown	12	15.317	6413992	227488	50.281	46.147	N/A	6666	N/A	1.090	

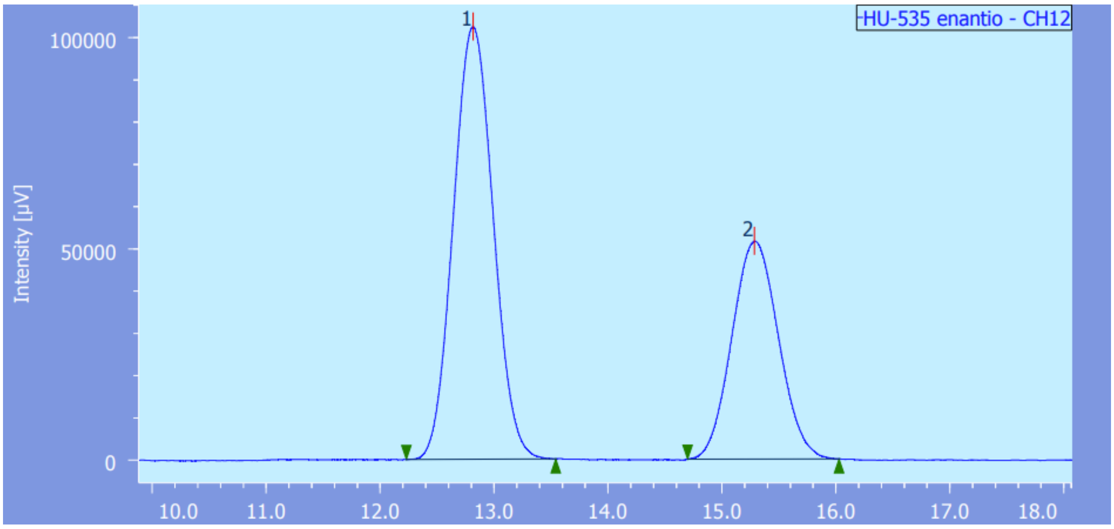

Decision

#	Peak Name	CH	tR [min]	Area [µV·sec]	Height [µV]	Area%	Height%	Quantity	NTP	Resolution	Symmetry Factor	Warning
1	Unknown	12	12.817	2493016	102362	62.929	66.486	N/A	6190	3.503	1.052	
2	Unknown	12	15.283	1468646	51599	37.071	33.514	N/A	6462	N/A	1.073	

(*S*)-4-(2-Cyclohexyl-1-phenylethyl)-1,1'-biphenyl (71)

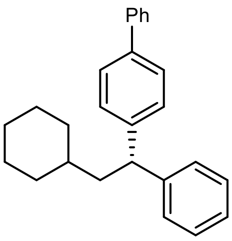

Prepared by **GP-B** (two 40 W 390 nm Kessile lamps were used). White solid, 23.3 mg, 34% yield. m.p. = 75-76 °C; ^{1}H NMR (400 MHz, $CDCl_3$) δ 7.61 – 7.55 (m, 2H), 7.54 – 7.49 (m, 2H), 7.45 – 7.39 (m, 2H), 7.35 – 7.27 (m, 7H), 7.22 – 7.17 (m, 1H), 4.13 (t, *J* = 8.0 Hz, 1H), 2.05 – 1.93 (m, 2H), 1.86 – 1.76 (m, 2H), 1.72 – 1.60 (m, 3H), 1.25 – 1.12 (m, 4H), 1.04 – 0.93 (m, 2H); ^{13}C NMR (101 MHz, $CDCl_3$) δ 145.3, 144.6, 141.0, 138.8, 128.7, 128.4, 128.2, 127.9, 127.1, 126.99, 126.96, 126.0, 47.6, 43.6, 34.9, 33.45, 33.39, 26.6, 26.1. IR (film): ν (cm^{-1}) 3057, 3025, 2919, 2907, 2850, 2838, 1600, 1485, 1446, 1408, 1262, 1167, 1111,1071, 1033, 1008, 965, 907, 890, 846, 829, 773, 756, 729, 714, 695, 596, 578, 494, 478; HR-MS (EI) m/z calcd for $[C_{26}H_{28}]^{+}$ $[M]^{+}$ 340.21910, found 340.21891; $[\alpha]_D^{22.3}$ = 11.9 (c = 0.1, $CHCl_3$); HPLC conditions: AD-H column, hexane/2-propanol = 98/2, flow rate = 0.2 mL/min, λ = 254 nm, t_R = 23.5 min (minor), t_R = 25.8 min (major), 98:2 er.

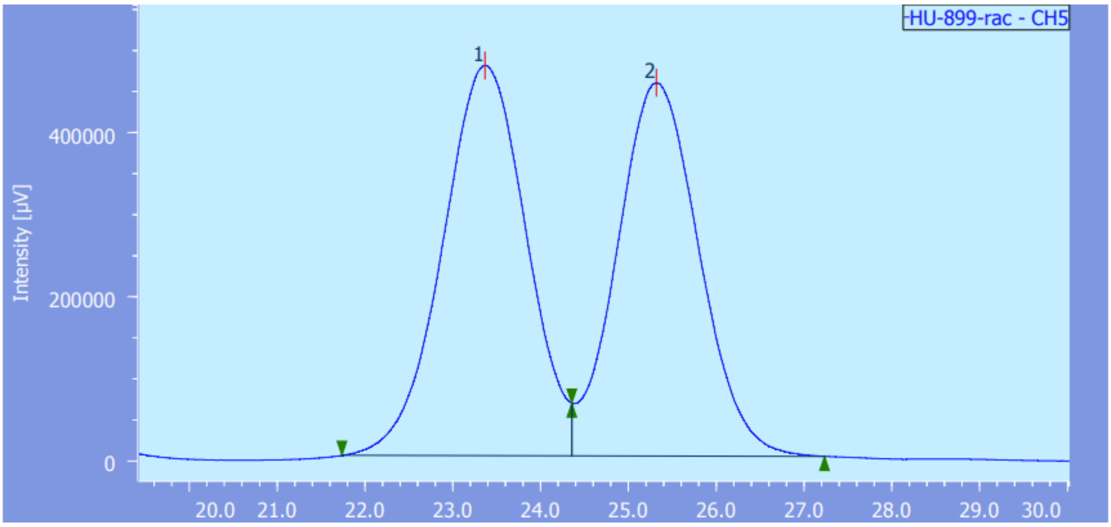

Decision

#	Peak Name	CH	tR [min]	Area [µV·sec]	Height [µV]	Area%	Height%	Quantity	NTP	Resolution	Symmetry Factor	Warning
1	Unknown	5	23.367	31449400	474936	51.510	51.114	N/A	2834	1.125	N/A	
2	Unknown	5	25.317	29605646	454229	48.490	48.886	N/A	3464	N/A	N/A	

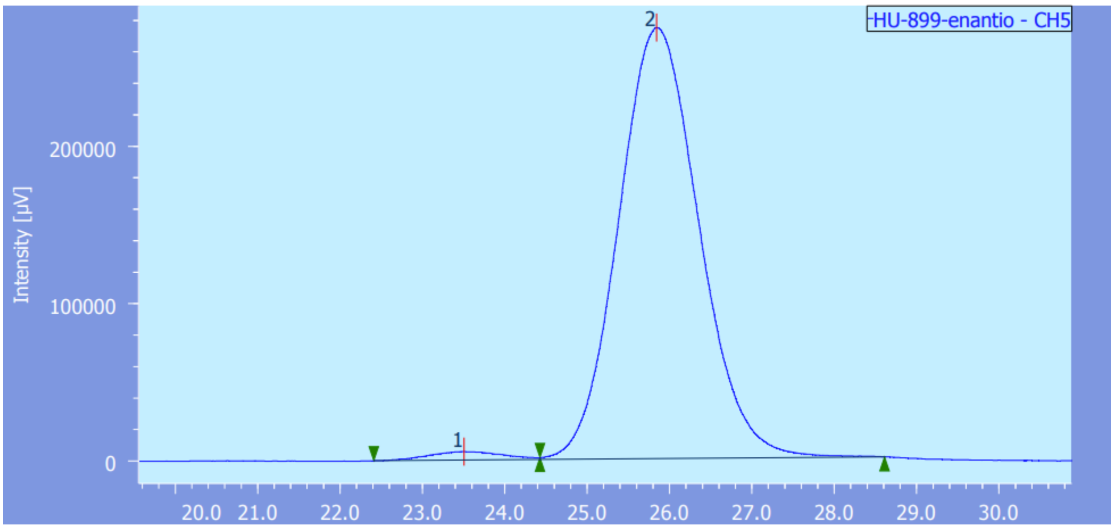

Decision

#	Peak Name	CH	tR [min]	Area [µV·sec]	Height [µV]	Area%	Height%	Quantity	NTP	Resolution	Symmetry Factor	Warning
1	Unknown	5	23.503	336142	5343	1.778	1.915	N/A	3043	1.344	N/A	
2	Unknown	5	25.840	18571070	273724	98.222	98.085	N/A	3366	N/A	1.107	

(*S*)-4-(1-(4-(*tert*-Butyl)phenyl)-2-cyclohexylethyl)-1,1'-biphenyl (72)

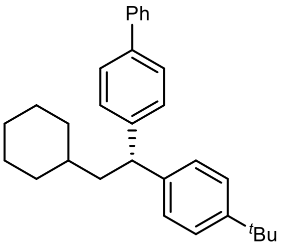

Prepared by **GP-B** (two 40 W 390 nm Kessile lamps were used). White solid, 30.8 mg, 39% yield. m.p. = 80-81 °C; ^{1}H NMR (400 MHz, $CDCl_3$) δ 7.61 – 7.55 (m, 2H), 7.54 – 7.49 (m, 2H), 7.46 – 7.38 (m, 2H), 7.36 – 7.28 (m, 5H), 7.24 – 7.18 (m, 2H), 4.09 (t, J = 7.9 Hz, 1H), 2.03 – 1.89 (m, 2H), 1.87 – 1.74 (m, 2H), 1.72 – 1.59 (m, 3H), 1.31 (s, 9H), 1.25 – 1.11 (m, 4H), 1.04 – 0.91 (m, 2H); ^{13}C NMR (101 MHz, $CDCl_3$) δ 148.6, 144.8, 142.4, 141.0, 138.7, 128.6, 128.3, 127.4, 127.1, 127.0, 125.3, 47.2, 43.7, 34.8, 34.3, 33.6, 33.2, 31.4, 26.6, 26.1. IR (film): ν (cm^{-1}) 3027, 2963, 2920, 2849, 2360, 2333, 1510, 1487, 1446, 1408, 1360, 1269, 1133, 1111, 1017, 1007, 848, 841, 830, 819, 763, 748, 727, 693, 606, 583, 565, 546, 483, 455, 431; HR-MS (EI) m/z calcd for $[C_{30}H_{36}]^+$ $[M]^+$ 396.28170, found 396.28174; $[\alpha]_D^{22.7}$ = -9.3 (c = 0.1, $CHCl_3$); HPLC conditions (Reverse Phase HPLC): Chiralpak IA column, H_2O/MeCN = 25/75, flow rate = 0.2 mL/min, λ = 254 nm, t_R = 147.5 min (major), t_R = 154.3 min (minor), 97:3 er (Note: approximate er value is provided).

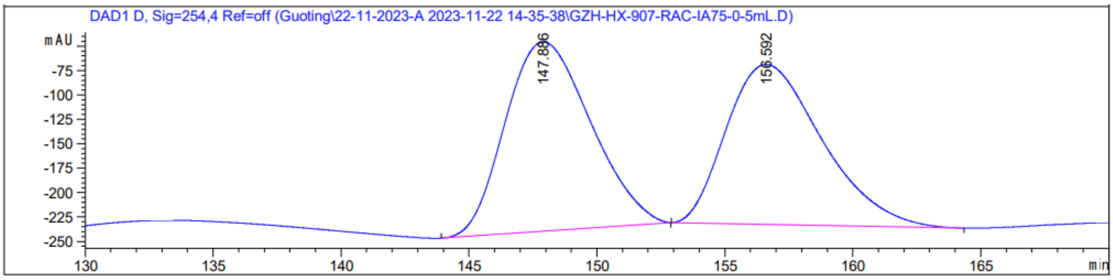

Peak #	RetTime [min]	Type	Width [min]	Area [mAU*s]	Height [mAU]	Area %
1	147.886	BB	3.5428	4.49460e4	194.00752	51.3385
2	156.592	BB	3.7864	4.26023e4	163.90739	48.6615

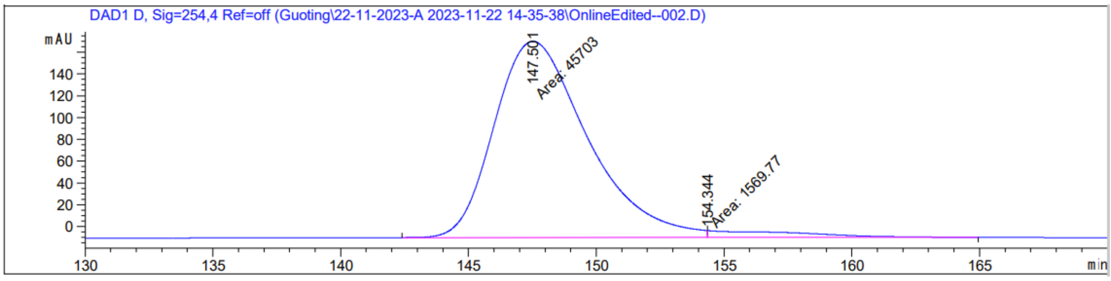

Peak #	RetTime [min]	Type	Width [min]	Area [mAU*s]	Height [mAU]	Area %
1	147.501	MF	4.2340	4.57030e4	179.90648	96.6793
2	154.344	FM	4.1888	1569.76709	6.24594	3.3207

(*R*)-4-(2-Cyclohexyl-1-(4-fluorophenyl)ethyl)-1,1'-biphenyl (73)

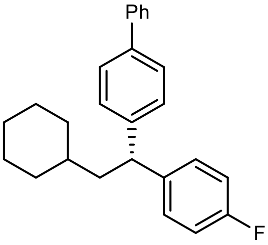

Prepared by **GP-B** (two 40 W 390 nm Kessile lamps were used). White solid, 25.8 mg, 36% yield. m.p. = 77-78 °C; ^{1}H NMR (400 MHz, $CDCl_3$) δ 7.60 – 7.55 (m, 2H), 7.54 – 7.50 (m, 2H), 7.45 – 7.40 (m, 2H), 7.35 – 7.27 (m, 3H), 7.25 – 7.20 (m, 2H), 7.02 – 6.95 (m, 2H), 4.10 (t, *J* = 8.0 Hz, 1H), 1.98 – 1.89 (m, 2H), 1.83 – 1.75 (m, 2H), 1.72 – 1.60 (m, 3H), 1.23 – 1.10 (m, 4H), 1.04 – 0.92 (m, 2H); ^{19}F NMR (377 MHz, $CDCl_3$) δ -117.34; ^{13}C NMR (101 MHz, $CDCl_3$) δ 161.2 (d, *J* = 244.8 Hz), 144.4, 141.0 (d, *J* = 3.1 Hz), 140.9, 139.0, 129.25, 129.17, 128.7, 128.1, 127.2, 127.1, 127.0, 115.2 (d, *J* = 21.1 Hz), 46.9, 43.7, 34.8, 33.5, 33.3, 26.6, 26.1. IR (film): ν (cm^{-1}) 3066, 3026, 2918, 2907, 2847, 2838, 1738, 1601, 1507, 1485, 1445, 1224, 1159, 1075, 1008, 824, 767, 742, 694, 578, 566, 522, 502, 416; HR-MS (EI) m/z calcd for $[C_{26}H_{27}F]^+$ $[M]^+$ 358.20968, found 358.20949; $[\alpha]_D^{22.8}$ = 4.4 (c = 0.1, $CHCl_3$); HPLC conditions: NR column, hexane/2-propanol = 98/2, flow rate = 0.2 mL/min, λ = 254 nm, t_R = 26.2 min (minor), t_R = 28.5 min (major), 97:3 er.

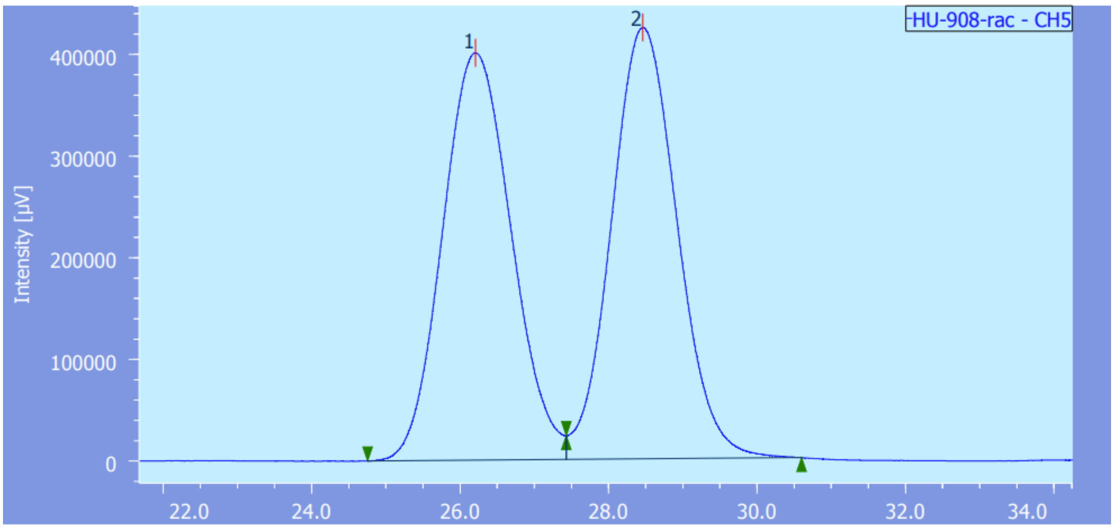

Decision

#	Peak Name	CH	tR [min]	Area [µV·sec]	Height [µV]	Area%	Height%	Quantity	NTP	Resolution	Symmetry Factor	Warning
1	Unknown	5	26.203	25788888	400307	49.718	48.567	N/A	3693	1.353	N/A	
2	Unknown	5	28.460	26081776	423927	50.282	51.433	N/A	4945	N/A	N/A	

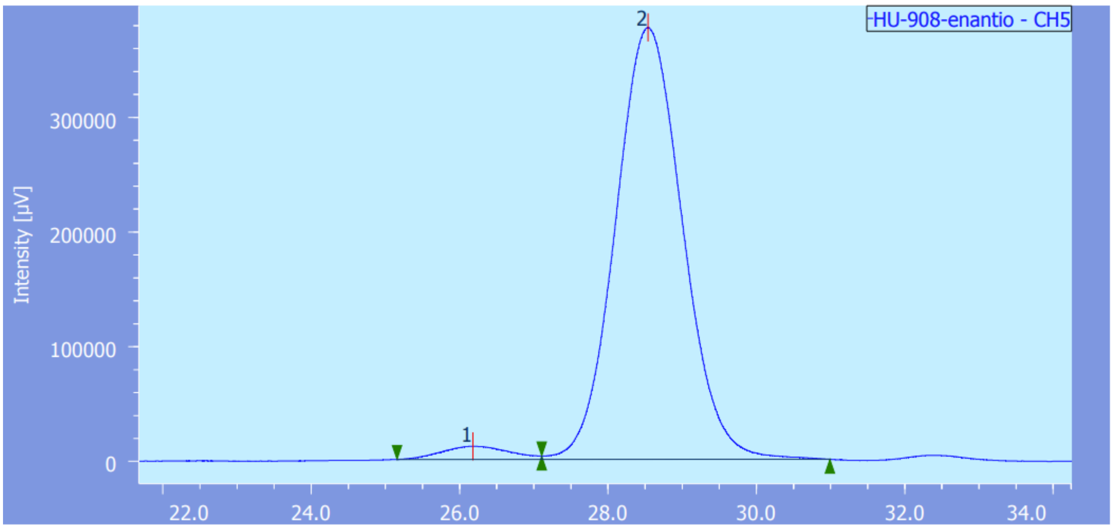

Decision

#	Peak Name	CH	tR [min]	Area [μV·sec]	Height [μV]	Area%	Height%	Quantity	NTP	Resolution	Symmetry Factor	Warning
1	Unknown	5	26.177	727379	11455	2.981	2.954	N/A	3539	1.389	N/A	
2	Unknown	5	28.537	23670320	376305	97.019	97.046	N/A	4800	N/A	1.071	

(*R*)-4-(1-(4-Chlorophenyl)-2-cyclohexylethyl)-1,1'-biphenyl (74)

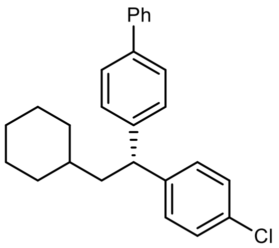

Prepared by **GP-B** (two 40 W 390 nm Kessile lamps were used). Colorless oil, 26.5 mg, 35% yield. ^{1}H NMR (400 MHz, CDCl3) δ 7.51 – 7.46 (m, 2H), 7.45 – 7.40 (m, 2H), 7.36 – 7.30 (m, 2H), 7.26 – 7.16 (m, 5H), 7.14 – 7.09 (m, 2H), 4.00 (t, *J* = 7.9 Hz, 1H), 1.84 (t, *J* = 7.3 Hz, 2H), 1.74 – 1.65 (m, 2H), 1.63 – 1.51 (m, 3H), 1.13 – 1.01 (m, 4H), 0.94 – 0.83 (m, 2H); ^{13}C NMR (101 MHz, $CDCl_3$) δ 144.0, 143.8, 140.8, 139.0, 131.7, 129.2, 128.7, 128.6, 128.1, 127.2, 127.1, 127.0, 47.0, 43.5, 34.8, 33.5, 33.3, 26.6, 26.1. IR (film): ν (cm^{-1}) 3026, 2919, 2849, 2361, 1599, 1488, 1448, 1408, 1092, 1012, 942, 909, 892, 845, 820, 762, 740, 696, 577, 527, 508, 496; HR-MS (EI) m/z calcd for $[C_{26}H_{27}Cl]^{+}$ $[M]^{+}$ 374.18013, found 374.17976; $[\alpha]_D^{22.8}$ = -4.8 (c = 0.1, $CHCl_3$); HPLC conditions (Reverse Phase HPLC): Chiralpak IA column, H_2O/MeCN = 25/75, flow rate = 0.2 mL/min, λ = 254 nm, t_R = 184.0 min (minor), t_R = 196.4 min (major), 97:3 er.

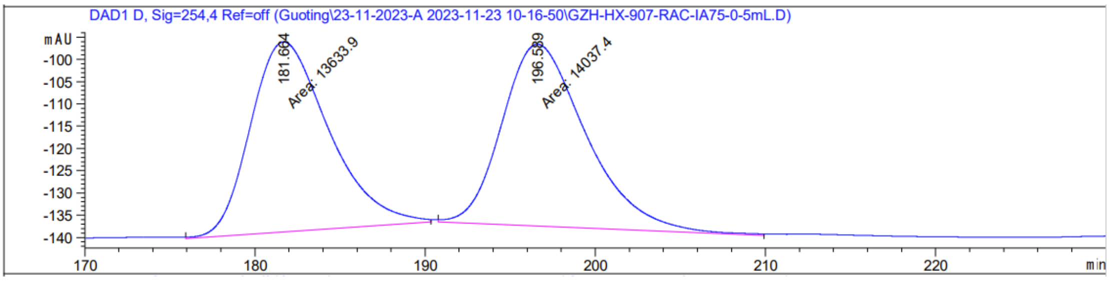

Peak #	RetTime [min]	Type	Width [min]	Area [mAU*s]	Height [mAU]	Area %
1	181.664	MM	5.3249	1.36339e4	42.67295	49.2708
2	196.589	MM	5.7300	1.40374e4	40.83017	50.7292

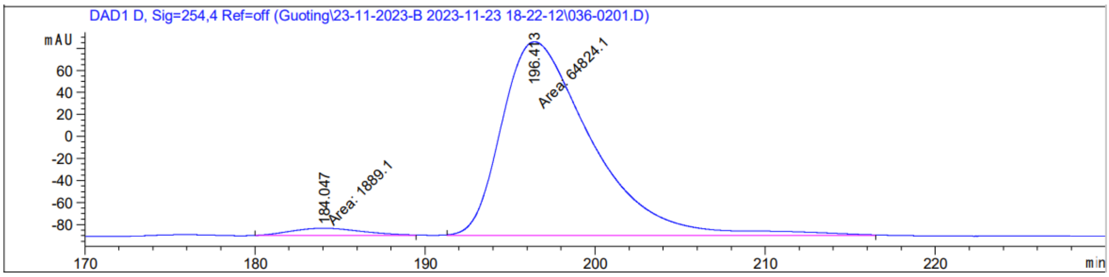

Peak #	RetTime [min]	Type	Width [min]	Area [mAU*s]	Height [mAU]	Area %
1	184.047	MM	4.8023	1889.09729	6.55622	2.8317
2	196.413	MM	6.1470	6.48241e4	175.75975	97.1683

(*R*)-4-(2-Cyclohexyl-1-(4-(trifluoromethyl)phenyl)ethyl)-1,1'-biphenyl (75)

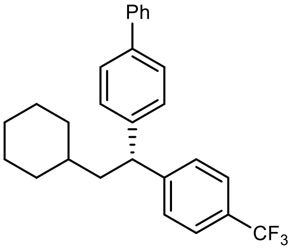

Prepared by **GP-B** (two 40 W 390 nm Kessile lamps were used). Colorless oil, 34.3 mg, 42% yield. ^{1}H NMR (400 MHz, $CDCl_3$) ^{1}H NMR (400 MHz, $CDCl_3$) δ 7.60 – 7.51 (m, 6H), 7.46 – 7.37 (m, 4H), 7.36 – 7.28 (m, 3H), 4.18 (t, *J* = 7.9 Hz, 1H), 2.05 – 1.91 (m, 2H), 1.84 – 1.76 (m, 2H), 1.74 – 1.60 (m, 3H), 1.25 – 1.10 (m, 4H), 1.05 – 0.93 (m, 2H); ^{19}F NMR (377 MHz, $CDCl_3$) δ -62.28; ^{13}C NMR (101 MHz, $CDCl_3$) δ 149.5, 143.4, 140.8, 139.3, 128.7, 128.6 (q, *J* = 5.2 Hz), 128.20, 128.18, 127.3, 127.2, 127.0, 125.4 (q, *J* = 3.7 Hz), 125.3 (q, *J* = 136.5 Hz), 47.6, 43.4, 34.8, 33.4, 33.4, 26.6, 26.1. IR (film): ν (cm^{-1}) 3054, 3032, 2923, 2852, 2358, 1615, 1488, 1448, 1418, 1322, 1159, 1118, 1111, 1066, 1016, 1009, 848, 835, 824, 771, 754, 726, 696, 613, 590, 519, 495, 420; HR-MS (EI) m/z calcd for $[C_{27}H_{27}F_3]^+$ $[M]^+$ 408.20649, found 408.20646; $[\alpha]_D^{22.7}$ = 7.5 (c = 0.1, $CHCl_3$); HPLC conditions (Reverse Phase HPLC): Chiralpak IA column, H_2O/MeCN = 25/75, flow rate = 0.2 mL/min, λ = 254 nm, t_R = 127.7 min (minor), t_R = 141.2 min (major), 97:3 er.

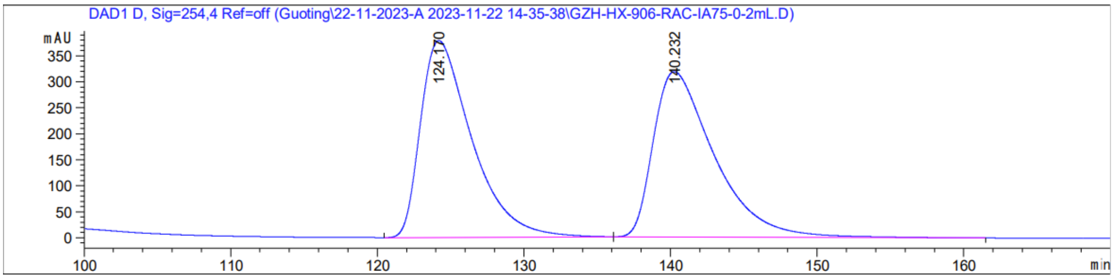

Peak #	RetTime [min]	Type	Width [min]	Area [mAU*s]	Height [mAU]	Area %
1	124.170	BB	3.4979	9.21235e4	378.72668	50.0277
2	140.232	BB	4.1475	9.20215e4	318.05643	49.9723

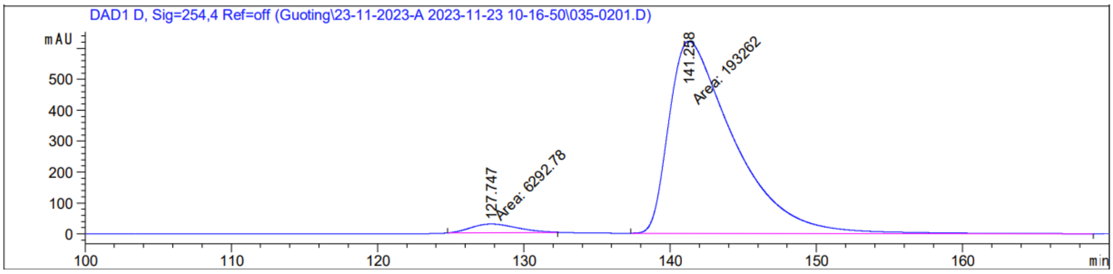

Peak #	RetTime [min]	Type	Width [min]	Area [mAU*s]	Height [mAU]	Area %
1	127.747	MM	3.6772	6292.77783	28.52137	3.1534
2	141.258	MM	5.1767	1.93262e5	622.22205	96.8466

Experimental Procedures and Characterization Data for Synthetic Applications.

4-((*S*)-2-((*tert*-Butoxycarbonyl)amino)-3-methoxy-3-oxopropyl)phenyl (*R*)-3-cyclohexyl-2-(4-(methylsulfonyl)phenyl)propanoate (76)

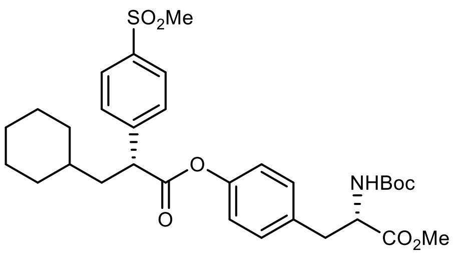

An oven-dried vial was charged with *tert*-butyl (*R*)-3-cyclohexyl-2-(4-(methylsulfonyl)phenyl)propanoate (0.13 mmol, 1.3 equiv) and CH_2Cl_2 (4 mL). TFA (1 mmol, 10 equiv) was added dropwise to the mixture at 0 °C. The reaction was stirred at 25 °C until TLC shows complete consumption of starting material. After reaction was complete, the solvent was evaporated under reduced pressure. The crude acid was dissolved in anhydrous CH_2Cl_2 (3 mL), then Boc-Tyr-OMe (0.1 mmol, 1.0 equiv), DCC (0.12 mmol, 1.2 equiv), and DMAP (0.01 mmol, 0.1 equiv) were added under N_2 atmosphere. The resulting reaction mixture was stirred for 2 h at 25 °C. After this time, 2 M NaOH (aq) was added, and the aqueous phase was extracted with CH_2Cl_2 twice. The combined organic layers were dried (anhydrous $MgSO_4$), filtered, and the solvent was evaporated under reduced pressure. After purification by chromatography on silica gel (hexanes/EtOAc = 1:1), the title compound was obtained as white solid in 66% yield (38.8 mg). m.p. = 91-92 °C; ^{1}H NMR (400 MHz, $CDCl_3$) δ 7.99 – 7.89 (m, 2H), 7.65 – 7.55 (m, 2H), 7.10 (d, *J* = 8.5 Hz, 2H), 6.97 – 6.87 (m, 2H), 4.96 (d, *J* = 7.9 Hz, 1H), 4.55 (dd, *J* = 12.8, 5.8 Hz, 1H), 4.01 (t, *J* = 7.8 Hz, 1H), 3.69 (s, 3H), 3.14 – 2.94 (m, 5H), 2.18 – 2.07 (m, 1H), 1.82 – 1.61 (m, 6H), 1.40 (s, 9H), 1.28 – 1.14 (m, 4H), 1.05 – 0.90 (m, 2H); ^{13}C NMR (101 MHz, $CDCl_3$) δ 172.1, 171.7, 155.0, 149.5, 145.2, 139.6, 133.9, 130.3, 129.1, 127.9, 121.2, 80.0, 54.3, 52.2, 48.9, 44.5, 41.0, 37.6, 35.4, 33.2, 32.9, 28.2, 26.3, 26.04, 25.99. IR (film): ν (cm^{-1}) 3349, 2924, 2852, 2358, 1737, 1656, 1515, 1440, 1301, 1210, 1146, 1090, 958, 839, 772, 742, 571, 537; HR-MS (ESI) m/z calcd for $C_{31}H_{41}NNaO_8S^+$ [M+Na$^+$] 610.24488, found 610.24451; $[\alpha]_D^{24.1}$ = -13.8 (c = 0.1, $CHCl_3$); dr> 20:1.

Methyl ((*R*)-3-cyclohexyl-2-(4-(methylsulfonyl)phenyl)propanoyl)-D-tryptophanate (77)

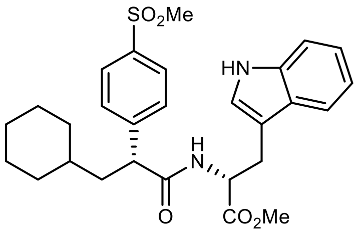

An oven-dried vial was charged with *tert*-butyl (*R*)-3-cyclohexyl-2-(4-(methylsulfonyl)phenyl)propanoate (0.11 mmol, 1.4 equiv) and CH_2Cl_2 (4 mL). TFA (1 mmol, 10 equiv) was added dropwise to the mixture at 0 °C. The reaction was stirred at 25 °C until TLC shows complete consumption of starting material. After reaction was complete, the solvent was evaporated under reduced pressure. The crude acid was dissolved in anhydrous DMF (1 mL), then D-tryptophan methyl ester hydrochloride (0.077 mmol, 1.0 equiv), HATU (0.115 mmol, 1.5 equiv), and DIPEA (0.23 mmol, 3.0 equiv) were added under a N_2 atmosphere. The resulting reaction mixture was stirred for 12 h at 25 °C. After this time, a saturated aqueous NH_4Cl solution was added, and the aqueous phase was extracted with EtOAc twice. The combined organic layers were washed with brine, dried (anhydrous $MgSO_4$), filtered, and the solvent was evaporated under reduced pressure. After purification by chromatography on silica gel (hexanes/EtOAc = 3:7), the title compound was obtained as white solid in 82% yield (32.4

mg). m.p. = 97-98 ºC; ^{1}H NMR (400 MHz, $CDCl_3$) δ 8.24 (s, 1H), 7.84 – 7.77 (m, 2H), 7.47 (d, *J* = 7.8 Hz, 1H), 7.41 – 7.34 (m, 3H), 7.23 – 7.17 (m, 1H), 7.15 – 7.07 (m, 1H), 6.66 (d, *J* = 2.4 Hz, 1H), 5.85 (d, *J* = 7.8 Hz, 1H), 4.82 (dt, *J* = 7.8, 5.5 Hz, 1H), 3.66 (s, 3H), 3.52 (dd, *J* = 8.4, 7.1 Hz, 1H), 3.32 – 3.18 (m, 2H), 3.06 (s, 3H), 2.01 – 1.88 (m, 1H), 1.73 – 1.57 (m, 6H), 1.17 – 1.00 (m, 4H), 0.97 – 0.81 (m, 2H); ^{13}C NMR (101 MHz, $CDCl_3$) δ 172.1, 172.0, 146.0, 139.1, 136.1, 129.0, 127.6, 127.3, 122.6, 122.3, 119.7, 118.4, 111.5, 109.4, 52.6, 52.4, 50.2, 44.2, 40.3, 34.9, 33.5, 32.6, 27.3, 26.3, 26.0, 25.9. IR (film): ν (cm^{-1}) 3370, 2978, 2918, 2848, 1749, 1708, 1597, 1506, 1448, 1364, 1307, 1197, 1165, 1148, 1124, 1055, 1016, 957, 781, 768, 541, 526; HR-MS (ESI) m/z calcd for $C_{28}H_{35}N_2O_5S^+$ [M+H$^+$] 511.22612, found 511.22612; $[\alpha]_D^{24.1}$ = -6.2 (c = 0.1, $CHCl_3$); dr> 20:1.

(*R*)-3-Cyclohexyl-2-(4-(methylsulfonyl)phenyl)propan-1-ol (78)

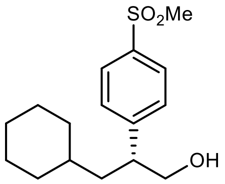

An oven-dried vial was charged with *tert*-butyl (*R*)-3-cyclohexyl-2-(4-(methylsulfonyl)phenyl)propanoate (0.6 mmol, 1.0 equiv), THF (5 mL) was added under a N_2 atmosphere. Then lithium aluminum hydride ($LiAlH_4$, 1 M in THF, 0.72 mmol, 1.2 equiv) was added dropwise to the mixture at 0 ºC. The reaction was stirred at 0 ºC until TLC shows complete consumption of starting material. The reaction was quenched by the dropwise addition of 0.5 mL H_2O and 5 mL of 1M NaOH solution. The resulting mixture was stirred at 25 ºC for 2 h and extracted with EtOAc (3*10 mL). The organic layer was dried (anhydrous $MgSO_4$) and concentrated under reduced pressure. After purification by chromatography on silica gel (hexanes/EtOAc = 1:1), the title compound was obtained as a colorless oil in 78% yield (138.8 mg). ^{1}H NMR (400 MHz, $CDCl_3$) δ 7.90 – 7.85 (m, 2H), 7.44 – 7.38 (m, 2H), 3.73 (ddd, *J* = 18.5, 10.8, 6.6 Hz, 2H), 3.09 – 2.96 (m, 4H), 1.78 – 1.50 (m, 8H), 1.16 – 0.98 (m, 4H), 0.96 – 0.77 (m, 2H). ^{13}C NMR (101 MHz, $CDCl_3$) δ 150.0, 138.6, 129.0, 127.6, 67.5, 45.5, 44.5, 39.5, 34.6, 34.1, 32.6, 26.4, 26.1, 26.0. IR (film): ν (cm^{-1}) 3516, 2921, 2849, 2360, 1737, 1596, 1446, 1411, 1301, 1294, 1146, 1090, 1053, 956, 833, 778, 754, 727, 590, 560, 538, 528; HR-MS (ESI) m/z calcd for $C_{16}H_{24}NaO_3S^+$ [M+Na$^+$] 319.13384, found 319.13396; $[\alpha]_D^{24.0}$ = -32.4 (c = 0.1, $CHCl_3$); HPLC conditions: AD-H column, hexane/2-propanol = 90/10, flow rate = 0.5 mL/min, λ = 230 nm, t_R = 59.1 min (major), t_R = 65.6 min (minor), 97:3 er.

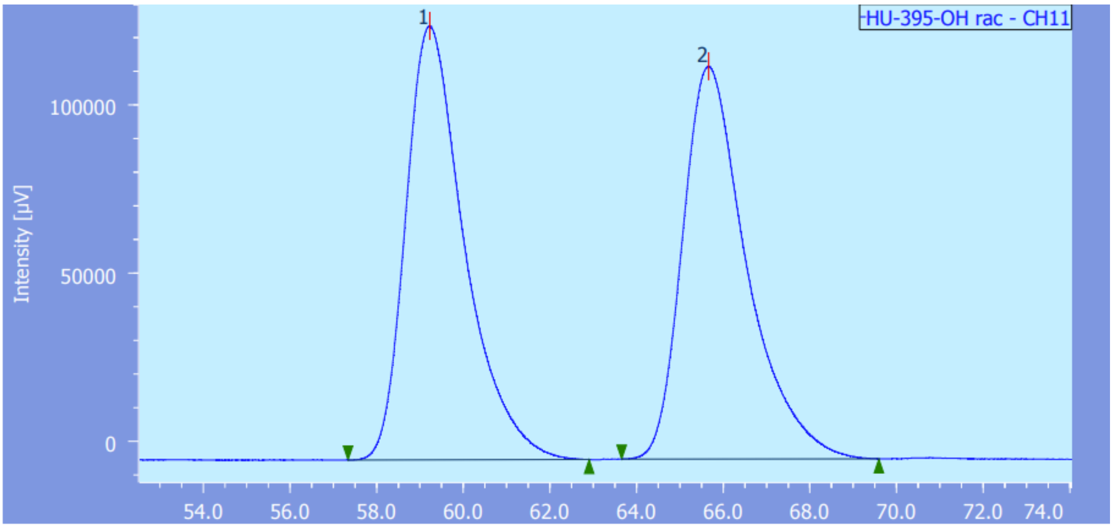

Decision

#	Peak Name	CH	tR [min]	Area [µV·sec]	Height [µV]	Area%	Height%	Quantity	NTP	Resolution	Symmetry Factor	Warning
1	Unknown	11	59.227	12155424	128835	50.273	52.486	N/A	9951	2.599	1.442	
2	Unknown	11	65.657	12023514	116630	49.727	47.514	N/A	10304	N/A	1.468	

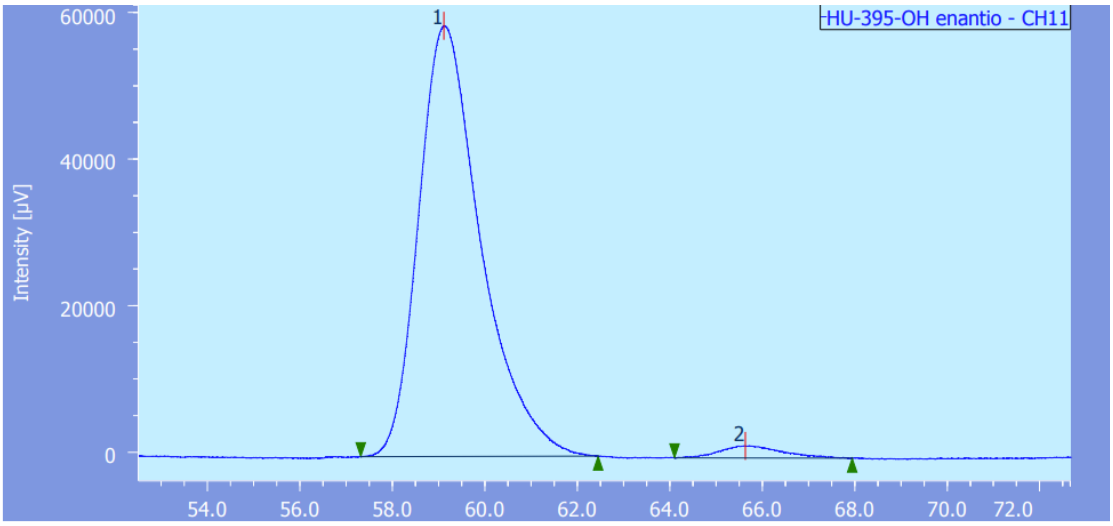

Decision

#	Peak Name	CH	tR [min]	Area [μV·sec]	Height [μV]	Area%	Height%	Quantity	NTP	Resolution	Symmetry Factor	Warning
1	Unknown	11	59.110	5494820	58702	97.224	97.244	N/A	10085	2.729	1.428	
2	Unknown	11	65.633	156890	1664	2.776	2.756	N/A	11580	N/A	1.174	

(*R*)-1-(1-Bromo-3-cyclohexylpropan-2-yl)-4-(methylsulfonyl)benzene (79)

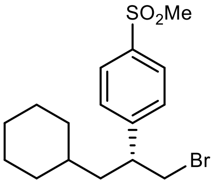

An oven-dried vial was charged with (*R*)-3-cyclohexyl-2-(4-(methylsulfonyl)phenyl)propan-1-ol (0.1 mmol, 1.0 equiv), PPh_3 (0.12 mmol, 1.2 equiv), and CBr_4 (0.11 mmol, 1.1 equiv). CH_2Cl_2 (2 mL) was added at 0 °C under a N_2 atmosphere. The reaction mixture was stirred at 0 °C for 2 h. The solvent was evaporated under reduced pressure, and the residual was purified by chromatography on silica gel (hexanes/EtOAc = 5:1). The title compound was obtained as a white solid in 96% yield (34.4 mg). m.p. = 80-81 °C; ^{1}H NMR (400 MHz, $CDCl_3$) δ 7.90 (d, *J* = 8.3 Hz, 2H), 7.39 (d, *J* = 8.4 Hz, 2H), 3.52 (ddd, *J* = 18.0, 10.1, 6.9 Hz, 2H), 3.24 – 3.14 (m, 1H), 3.08 (s, 3H), 1.78 – 1.52 (m, 7H), 1.18 – 0.97 (m, 4H), 0.97 – 0.81 (m, 2H); ^{13}C NMR (101 MHz, $CDCl_3$) δ 149.2, 139.1, 128.7, 127.6, 45.0, 44.5, 42.1, 38.1, 34.8, 33.9, 32.5, 26.3, 26.0, 25.9. IR (film): ν (cm^{-1}) 2923, 2850, 1737, 1596, 1444, 1412, 1305, 1287, 1268, 1226, 1146, 1124, 1093, 965, 959, 943, 836, 793, 777, 757, 735, 610, 570, 556, 526, 503; HR-MS (ESI) m/z calcd for $C_{16}H_{24}BrO_2S^+$ [M+H$^+$] 359.06749, found 359.06739; $[\alpha]_D^{24.0}$ = -9.0 (c = 0.1, $CHCl_3$); HPLC conditions: OJ-H column, hexane/2-propanol = 95/5, flow rate = 1.0 mL/min, λ = 225 nm, t_R = 60.0 min (major), t_R = 7.5 min (minor), 97:3 er.

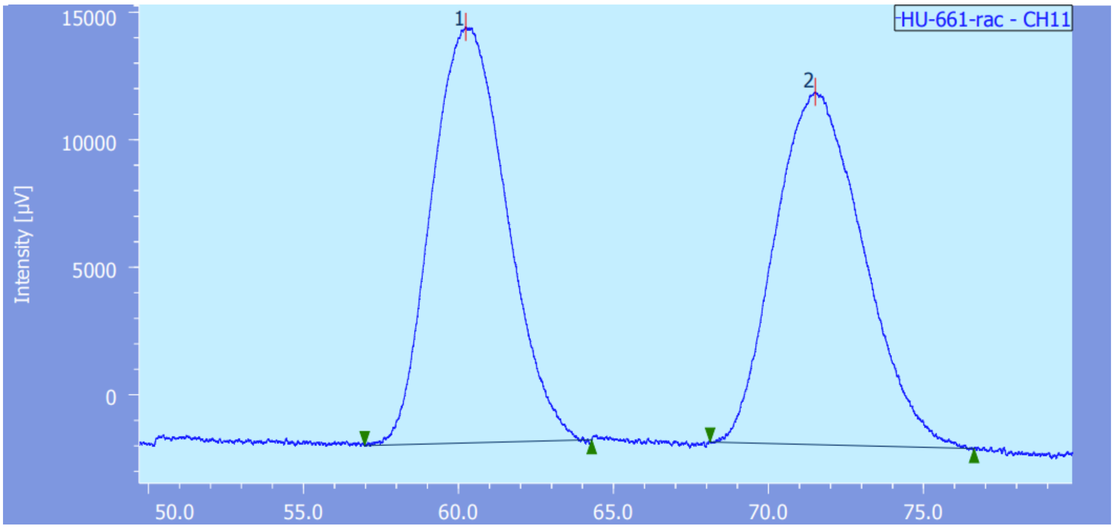

Decision

#	Peak Name	CH	tR [min]	Area [µV·sec]	Height [µV]	Area%	Height%	Quantity	NTP	Resolution	Symmetry Factor	Warning
1	Unknown	11	60.230	2745822	16297	50.041	54.119	N/A	2764	2.252	1.179	
2	Unknown	11	71.500	2741340	13817	49.959	45.881	N/A	2749	N/A	1.196	

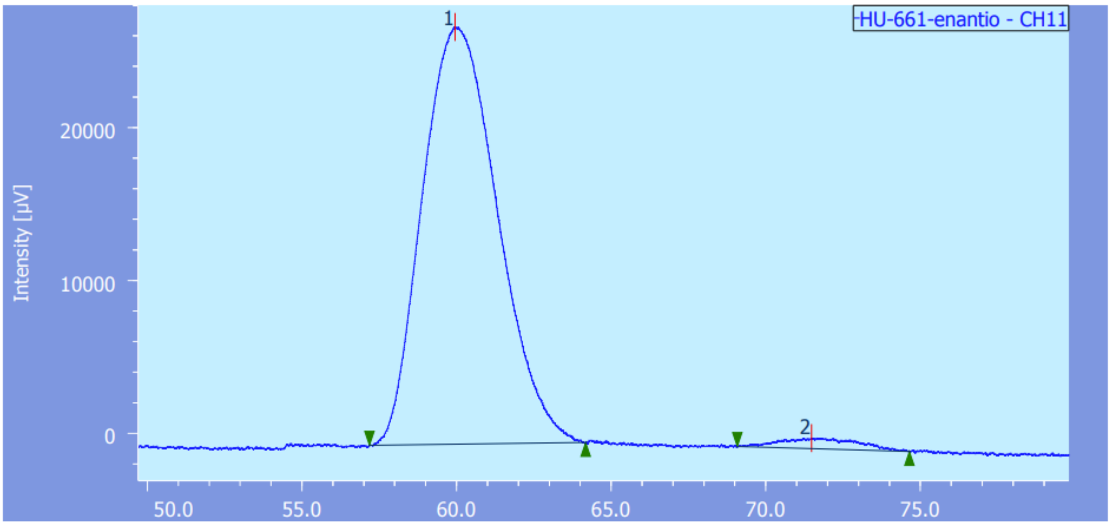

Decision

#	Peak Name	CH	tR [min]	Area [µV·sec]	Height [µV]	Area%	Height%	Quantity	NTP	Resolution	Symmetry Factor	Warning
1	Unknown	11	59.950	4617445	27262	97.385	97.509	N/A	2732	2.250	1.238	
2	Unknown	11	71.477	123991	696	2.615	2.491	N/A	2531	N/A	1.165	

(*R*)-2-(3-Cyclohexyl-2-(4-(methylsulfonyl)phenyl)propyl)isoindoline-1,3-dione (80)

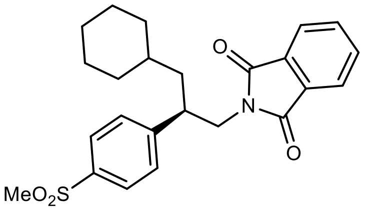

An oven-dried vial was charged with (*R*)-3-cyclohexyl-2-(4-(methylsulfonyl)phenyl)propan-1-ol (0.1 mmol, 1.0 equiv), PPh_3 (0.15 mmol, 1.5 equiv), isoindoline-1,3-dione (0.15 mmol, 1.5 equiv), and THF (2 mL). DIAD (0.15 mmol, 1.5 equiv) was added slowly at 0 °C under a N_2 atmosphere. The reaction mixture was stirred at 25 °C for 20 h. The solvent was evaporated under reduced pressure and the residual was purified by chromatography on silica gel (hexanes/EtOAc = 2:1). The title compound was obtained as a white solid in 88% yield (37.3 mg). m.p. = 140-141°C; ^{1}H NMR (400 MHz, $CDCl_3$) δ 7.80 (d, *J* = 8.3 Hz, 2H), 7.78 – 7.72 (m, 2H), 7.71 – 7.64 (m, 2H), 7.40 (d, *J* = 8.3 Hz, 2H), 3.83 (ddd, *J* = 20.4, 13.6, 8.0 Hz, 2H), 3.55 – 3.40 (m, 1H), 3.00 (s, 3H), 1.78 – 1.48 (m, 7H), 1.16 – 0.97 (m, 4H), 0.95 – 0.76 (m, 2H); ^{13}C NMR (101

MHz, $CDCl_3$) δ 168.0, 148.8, 138.8, 134.0, 131.6, 129.0, 127.5, 123.2, 44.4, 43.6, 41.3, 41.1, 34.7, 34.0, 32.4, 26.4, 26.1, 25.9. IR (film): ν (cm^{-1}) 2922, 2853, 1766, 1710, 1430, 1388, 1369, 1303, 1153, 1089, 1081, 973, 956, 830, 754, 719, 709, 693, 563, 548, 528, 497; HR-MS (ESI) m/z calcd for $C_{24}H_{27}NNaO_4S^+$ [M+Na$^+$] 448.15530, found 448.15538; $[\alpha]_D^{24.0}$ = 71.2 (c = 0.1, $CHCl_3$); HPLC conditions: AD-H column, hexane/2-propanol = 85/15, flow rate = 1.0 mL/min, λ = 230 nm, t_R = 28.0 min (major), t_R = 30.8 min (minor), 97:3 er.

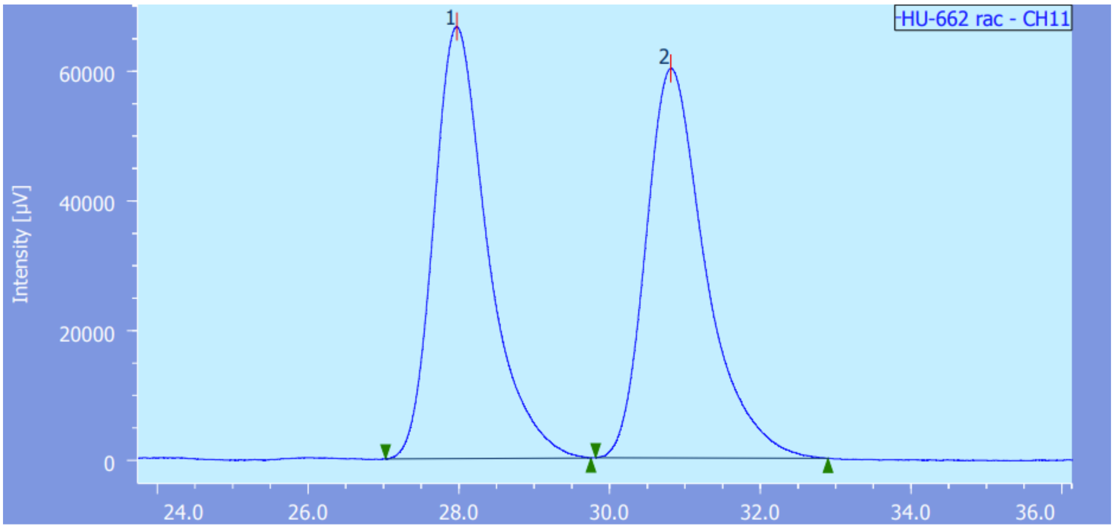

Decision

#	Peak Name	CH	tR [min]	Area [µV·sec]	Height [µV]	Area%	Height%	Quantity	NTP	Resolution	Symmetry Factor	Warning
1	Unknown	11	27.973	3310966	66581	50.059	52.590	N/A	8101	2.172	1.384	
2	Unknown	11	30.810	3303182	60023	49.941	47.410	N/A	8030	N/A	1.423	

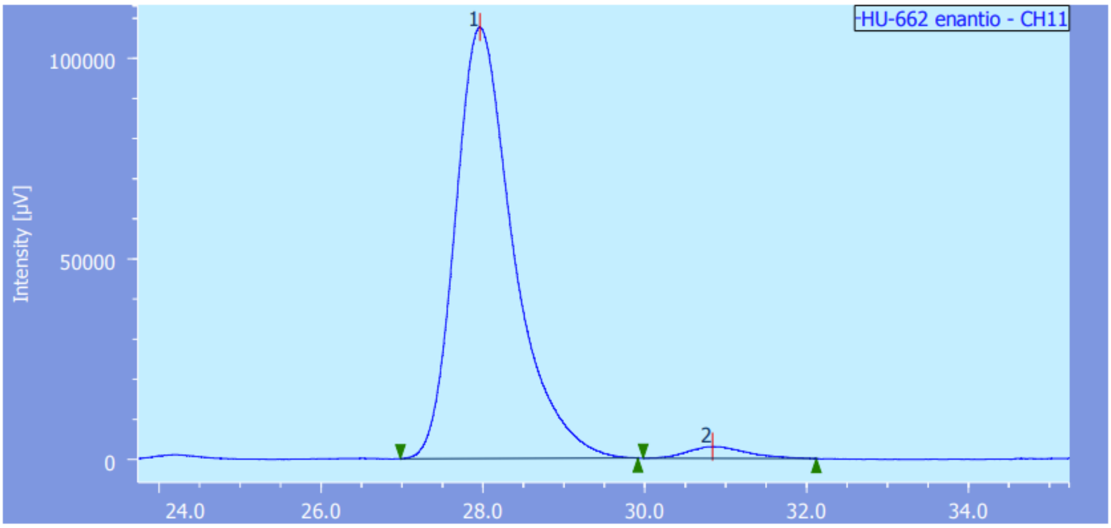

Decision

#	Peak Name	CH	tR [min]	Area [µV·sec]	Height [µV]	Area%	Height%	Quantity	NTP	Resolution	Symmetry Factor	Warning
1	Unknown	11	27.963	5365986	107465	97.292	97.346	N/A	8081	2.235	1.401	
2	Unknown	11	30.837	149342	2930	2.708	2.654	N/A	8548	N/A	1.253	

(*R*)-2-((3-Cyclohexyl-2-(4-(methylsulfonyl)phenyl)propyl)thio)benzo[d]thiazole (81)

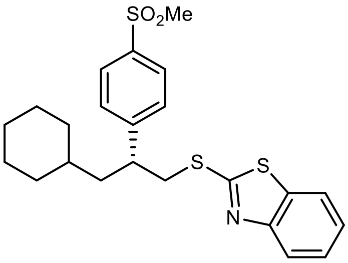

An oven-dried vial was charged with (*R*)-3-cyclohexyl-2-(4-(methylsulfonyl)phenyl)propan-1-ol (0.1 mmol, 1.0 equiv), PPh_3 (0.12 mmol, 1.2 equiv), 2(3H)-benzothiazolethione (0.11 mmol, 1.1 equiv), and THF (2 mL). DEAD (0.12 mmol, 1.2 equiv) was added slowly at 0 °C under a N_2 atmosphere. The reaction mixture was stirred at 25 °C for 4 h. The solvent was evaporated under reduced pressure, and the residual was purified by chromatography on silica gel (hexanes: EtOAc = 3:1), the title compound was obtained as a colorless oil in 99% yield (44.2 mg). ^{1}H NMR (400 MHz, $CDCl_3$) δ 7.88 – 7.82 (m, 3H), 7.76 – 7.70 (m, 1H), 7.46 – 7.38 (m, 3H), 7.33 – 7.27 (m, 1H), 3.72 (dd, J = 13.2, 5.7 Hz, 1H), 3.51 (dd, J = 13.2, 8.7 Hz, 1H), 3.38 – 3.27 (m, 1H), 2.97 (s, 3H), 1.81 – 1.57 (m, 7H), 1.21 – 1.03 (m, 4H), 0.98 – 0.83 (m, 2H); ^{13}C NMR (101 MHz, $CDCl_3$) δ 166.5, 152.7, 149.9, 138.8, 135.0, 128.9, 127.6, 126.2, 124.4, 121.3, 121.0, 44.4, 43.1, 42.7, 39.9, 34.8, 33.8, 32.6, 26.4, 26.07, 25.95. IR (film): ν (cm^{-1}) 2921, 2849, 1597, 1458, 1427, 1411, 1308, 1292, 1239, 1146, 1090, 993, 954, 836, 780, 754, 726, 575, 556, 532, 503; HR-MS (ESI) m/z calcd for $C_{23}H_{28}NO_2S_3^+$ [M+H$^+$] 446.12767, found 446.12805; $[\alpha]_D^{24.0}$ = 74.8 (c = 0.1, $CHCl_3$); HPLC conditions: IB column, hexane/2-propanol = 85/15, flow rate = 1.0 mL/min, λ = 230 nm, t_R = 16.9 min (major), t_R = 20.2 min (minor), 97:3 er.

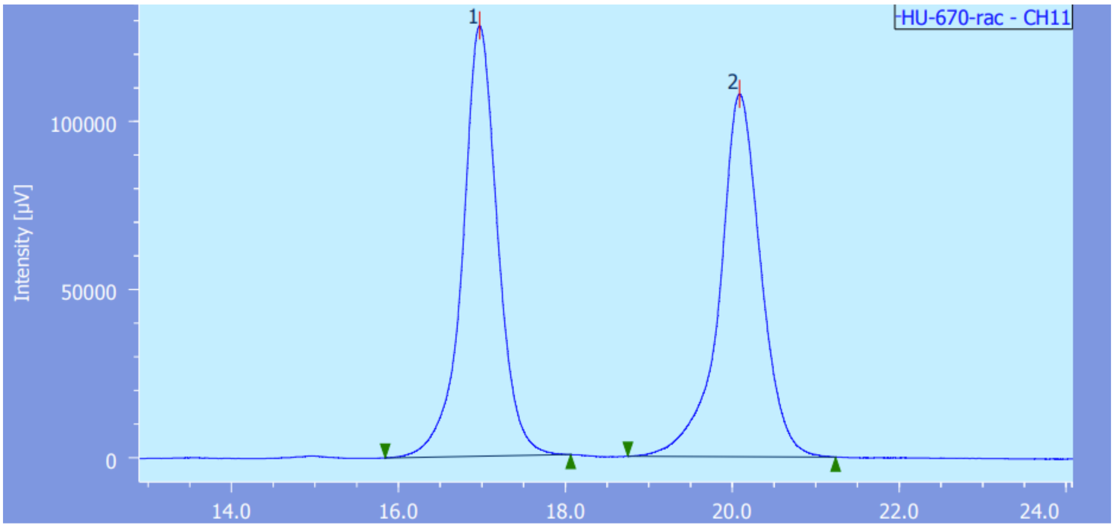

Decision

#	Peak Name	CH	tR [min]	Area [µV·sec]	Height [µV]	Area%	Height%	Quantity	NTP	Resolution	Symmetry Factor	Warning
1	Unknown	11	16.970	3748086	127984	50.106	54.270	N/A	9070	4.062	0.971	
2	Unknown	11	20.083	3732171	107843	49.894	45.730	N/A	9502	N/A	0.924	

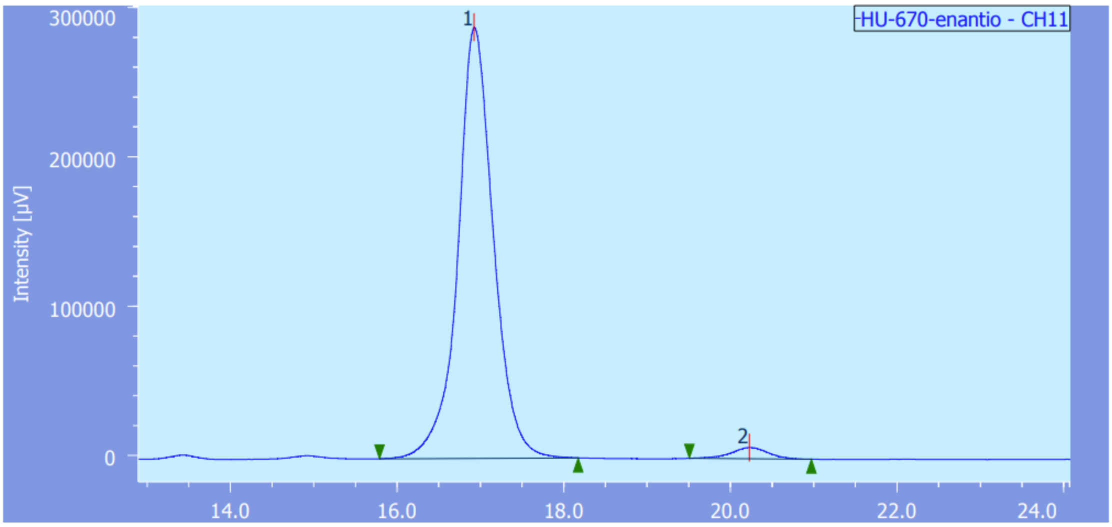

Decision

#	Peak Name	CH	tR [min]	Area [µV·sec]	Height [µV]	Area%	Height%	Quantity	NTP	Resolution	Symmetry Factor	Warning
1	Unknown	11	16.923	8564961	288459	97.333	97.465	N/A	8727	4.357	1.016	
2	Unknown	11	20.227	234661	7502	2.667	2.535	N/A	10334	N/A	0.933	

***tert*-Butyl (*R*)-2-(3-chloro-4-(methylsulfonyl)phenyl)-3-cyclopentylpropanoate (82)**

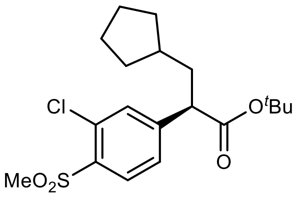

Prepared by **GP-B**. Colorless oil, 50.3 mg, 65% yield. ^{1}H NMR (400 MHz, $CDCl_3$) δ 8.07 (d, *J* = 8.2 Hz, 1H), 7.51 (d, *J* = 1.7 Hz, 1H), 7.40 (dd, *J* = 8.2, 1.7 Hz, 1H), 3.54 (t, *J* = 7.7 Hz, 1H), 3.26 (s, 3H), 2.04 (dt, *J* = 13.4, 7.8 Hz, 1H), 1.78 – 1.69 (m, 3H), 1.65 – 1.56 (m, 3H), 1.50 – 1.45 (m, 2H), 1.40 (s, 9H), 1.16 – 1.02 (m, 2H); ^{13}C NMR (101 MHz, $CDCl_3$) δ 171.8, 147.8, 136.4, 132.5, 131.3, 130.8, 127.1, 81.6, 51.6, 42.8, 39.9, 37.8, 32.7, 32.3, 27.9, 25.1, 25.0. IR (film): ν (cm^{-1}) 3008, 2977, 2950, 2868, 1713, 1590, 1454, 1392, 1367, 1317, 1246, 1137, 1115, 1039, 965, 839, 763, 599, 533, 508; HR-MS (ESI) m/z calcd for $C_{19}H_{27}ClNaO_4S^+$ [M+Na$^+$] 409.12108, found 409.12109; $[\alpha]_D^{24.0}$ = -18.2 (c = 0.1, $CHCl_3$); HPLC conditions: IB column, hexane/2-propanol = 99/1, flow rate = 0.5 mL/min, λ = 230 nm, t_R = 38.3 min (major), t_R = 41.8 min (minor), 97:3 er.

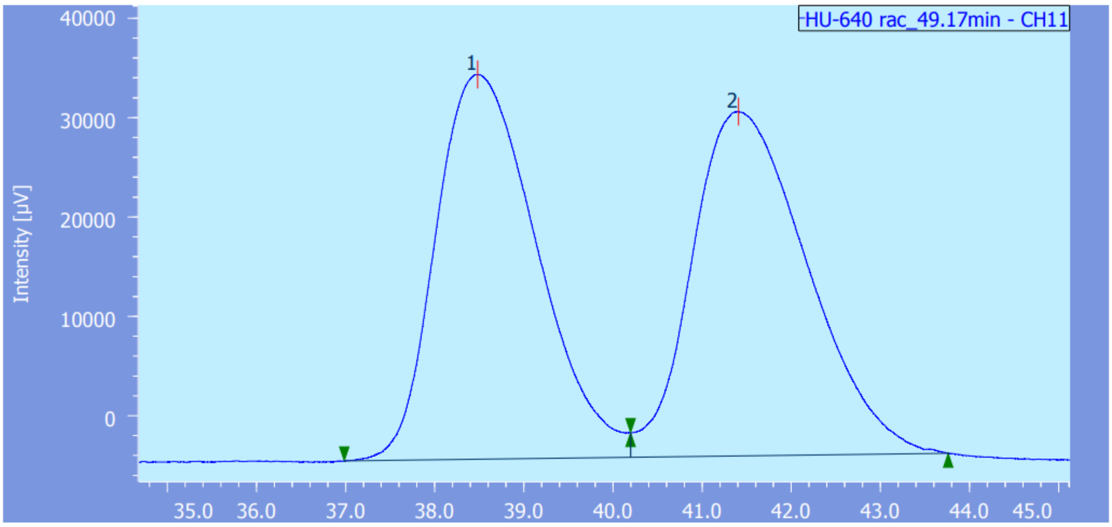

Decision

#	Peak Name	CH	tR [min]	Area [µV·sec]	Height [µV]	Area%	Height%	Quantity	NTP	Resolution	Symmetry Factor	Warning
1	Unknown	11	38.480	3053491	38680	49.514	52.770	N/A	5302	1.300	N/A	
2	Unknown	11	41.407	3113448	34618	50.486	47.230	N/A	4765	N/A	N/A	

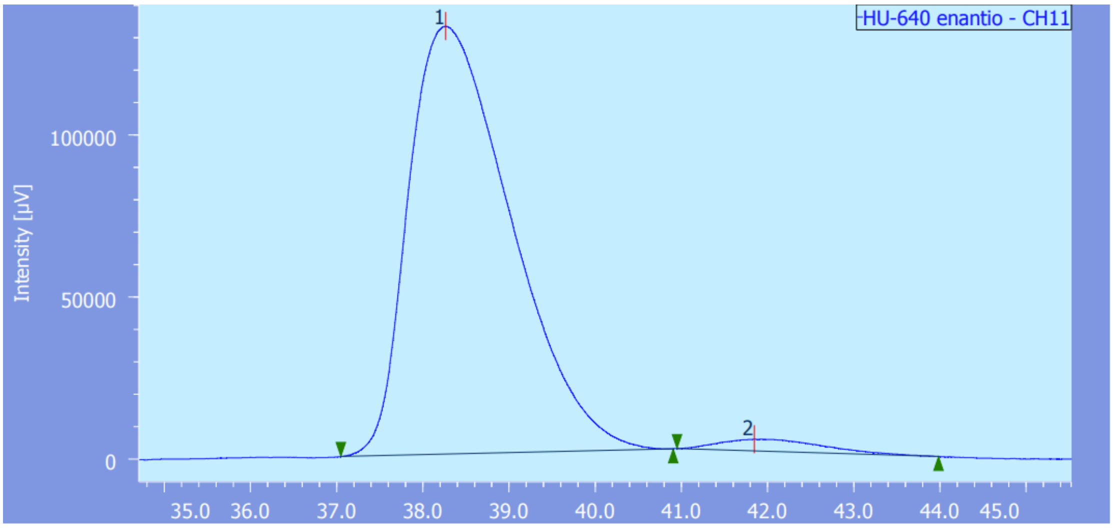

Decision

#	Peak Name	CH	tR [min]	Area [µV·sec]	Height [µV]	Area%	Height%	Quantity	NTP	Resolution	Symmetry Factor	Warning
1	Unknown	11	38.263	10927037	131976	97.221	97.350	N/A	4721	1.564	1.585	
2	Unknown	11	41.843	312305	3592	2.779	2.650	N/A	5015	N/A	1.604	

(*R*)-2-(3-Chloro-4-(methylsulfonyl)phenyl)-3-cyclopentyl-N-(pyrazin-2-yl)propanamide (83)

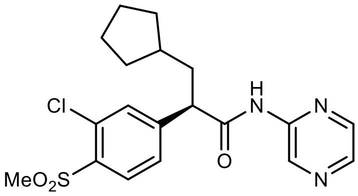

An oven-dried vial was charged with *tert*-butyl (*R*)-2-(3-chloro-4-(methylsulfonyl)phenyl)-3-cyclopentylpropanoate (0.1 mmol, 1.0 equiv) and CH_2Cl_2 (4 mL). TFA (1 mmol, 10 equiv) was added dropwise to the mixture at 0 °C, and the reaction was stirred at 25 °C until TLC shows complete consumption of starting material. After the reaction was complete, the solvent was evaporated under reduced pressure. The crude acid was then dissolved in anhydrous CH_2Cl_2 (2 mL), oxalyl chloride (0.11 mmol, 1.1 equiv) and one drop of DMF was added at 0 °C. The reaction was stirred at 0 °C for 10 min then at room temperature for 1 h to obtain a solution of the acid chloride. A solution of 2-aminopyrazine (0.22 mmol, 2.2 equiv) and pyridine (0.22 mmol, 2.2 equiv) in anhydrous THF (2 mL) was added. The reaction was stirred at 25 °C until TLC shows complete consumption of starting material. After purification by chromatography on silica gel (hexanes/ EtOAc = 2:1), the title compound was obtained as a white solid in 72% yield (29.3 mg). m.p. = 86-87 °C; ^{1}H NMR (400 MHz, $CDCl_3$) δ 9.52 (s, 1H), 8.35 (d, *J* = 2.4 Hz, 1H), 8.27 (s, 1H), 8.23 – 8.18 (m, 1H), 8.10 (d, *J* = 8.2 Hz, 1H), 7.61 (d, *J* = 1.6 Hz, 1H), 7.50 (dd, *J* = 8.2, 1.6 Hz, 1H), 3.71 (t, *J* = 7.4 Hz, 1H), 3.27 (s, 3H), 2.22 (dt, *J* = 13.6, 7.6 Hz, 1H), 1.94 – 1.84 (m, 1H), 1.82 – 1.72 (m, 2H), 1.71 – 1.64 (m, 1H), 1.64 – 1.56 (m, 2H), 1.53 – 1.44 (m, 2H), 1.19 – 1.09 (m, 2H); ^{13}C NMR (101 MHz, $CDCl_3$) δ 170.4, 147.8, 146.9, 142.0, 140.5, 137.0, 136.9, 133.0, 131.3, 131.2, 127.0, 52.7, 42.8, 40.0, 37.7, 32.7, 32.3, 25.0. IR (film): ν (cm^{-1}) 3292, 2949, 2927, 2860, 1696, 1590, 1538, 1513, 1409, 1310, 1292, 1266, 1179, 1148, 1115, 1038, 1010, 956, 843, 761, 596, 507, 418; HR-MS (ESI) m/z calcd for $C_{19}H_{23}ClN_3O_3S^+$ [M+H$^+$] 408.11432, found 408.11428; $[\alpha]_D^{24.0}$ = -33.6 (c = 0.1, $CHCl_3$); HPLC conditions: AD-H column, hexane/2-propanol = 85/15, flow rate = 1.0 mL/min, λ = 230 nm, t_R = 21.6 min (minor), t_R = 23.8 min (major), 97:3 er.

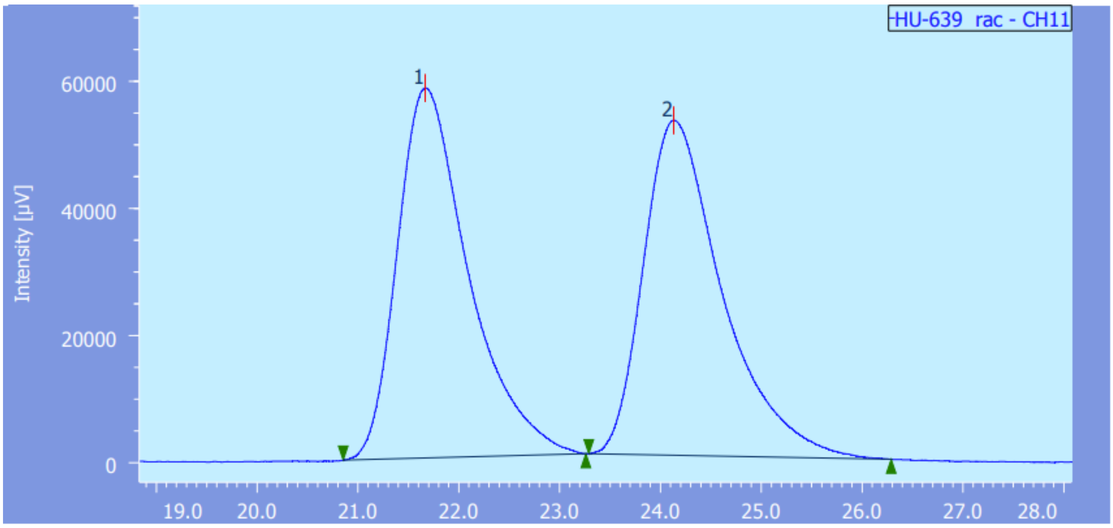

Decision

#	Peak Name	CH	tR [min]	Area [µV·sec]	Height [µV]	Area%	Height%	Quantity	NTP	Resolution	Symmetry Factor	Warning
1	Unknown	11	21.667	2852546	58170	49.910	52.504	N/A	4909	1.902	1.511	
2	Unknown	11	24.130	2862853	52622	50.090	47.496	N/A	5039	N/A	1.574	

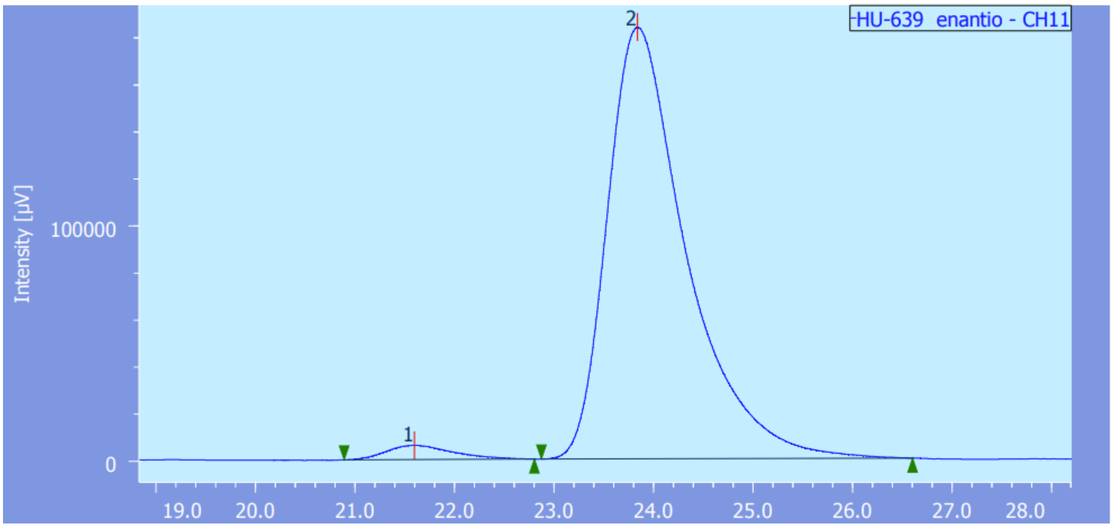

Decision

#	Peak Name	CH	tR [min]	Area [µV·sec]	Height [µV]	Area%	Height%	Quantity	NTP	Resolution	Symmetry Factor	Warning
1	Unknown	11	21.597	287424	6128	2.756	3.232	N/A	5060	1.745	1.348	
2	Unknown	11	23.837	10141524	183491	97.244	96.768	N/A	4918	N/A	1.613	

***tert*-Butyl (*R*)-3-cyclopentyl-2-(4-(methylsulfonyl)phenyl)propanoate (84)**

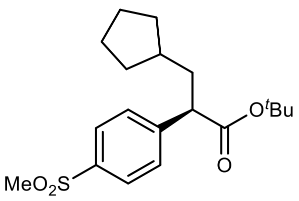

Prepared by **GP-B**. White solid, 48 mg, 68% yield. m.p. = 80-81 ºC; ^{1}H NMR (400 MHz, $CDCl_3$) δ 7.91 – 7.86 (m, 2H), 7.55 – 7.48 (m, 2H), 3.58 (t, *J* = 7.7 Hz, 1H), 3.05 (s, 3H), 2.06 (dt, *J* = 13.5, 7.7 Hz, 1H), 1.80 – 1.68 (m, 3H), 1.65 – 1.56 (m, 3H), 1.47 – 1.43 (m, 2H), 1.39 (s, 9H), 1.19 – 1.02 (m, 2H); ^{13}C NMR (101 MHz, $CDCl_3$) δ 172.4, 146.3, 139.1, 129.0, 127.5, 81.2, 51.9, 44.5, 39.9, 37.8, 32.7, 32.3, 27.9, 25.05, 25.02. IR (film): ν (cm^{-1}) 3005, 2978, 2941, 2867, 1720, 1595, 1453, 1366, 1315, 1305, 1240, 1138, 1090, 962, 843, 771, 747, 565, 547, 529, 465; HR-MS (ESI) m/z calcd for $C_{19}H_{28}NaO_4S^+$ [M+Na$^+$] 375.16005, found 375.15956; $[\alpha]_D^{24.0}$ = -15.6 (c = 0.1, $CHCl_3$); HPLC conditions (er was determined by reducing the product to the corresponding alcohol with $LiAlH_4$): OJ-H column, hexane/2-propanol = 85/15, flow rate = 1.0 mL/min, λ = 230 nm, t_R = 23.1 min (minor), t_R = 30.2

min (major), 97:3 er.

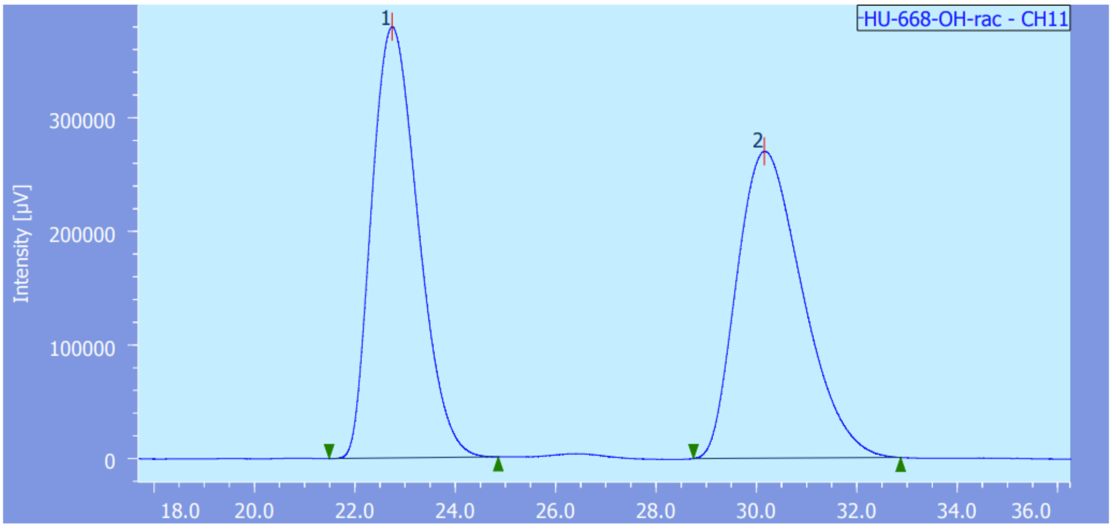

Decision

#	Peak Name	CH	tR [min]	Area [µV·sec]	Height [µV]	Area%	Height%	Quantity	NTP	Resolution	Symmetry Factor	Warning
1	Unknown	11	22.740	24366336	379289	49.757	58.411	N/A	2762	3.562	1.291	
2	Unknown	11	30.153	24604240	270061	50.243	41.589	N/A	2438	N/A	1.358	

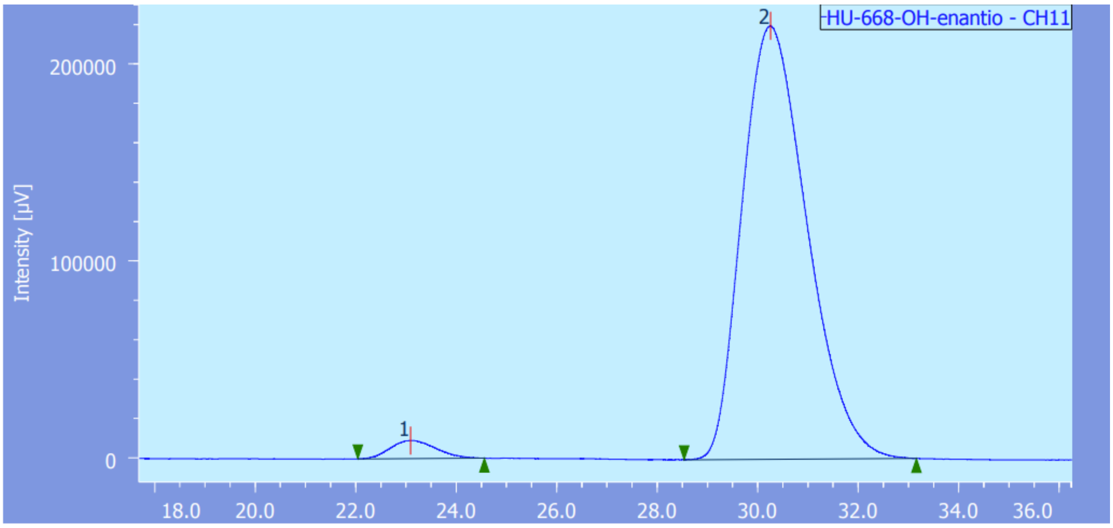

Decision

#	Peak Name	CH	tR [min]	Area [µV·sec]	Height [µV]	Area%	Height%	Quantity	NTP	Resolution	Symmetry Factor	Warning
1	Unknown	11	23.090	582699	9206	2.862	4.019	N/A	2905	3.482	1.196	
2	Unknown	11	30.250	19774561	219853	97.138	95.981	N/A	2522	N/A	1.303	

(*R*)-3-Cyclopentyl-2-(4-(methylsulfonyl)phenyl)-N-(thiazol-2-yl)propanamide (85)

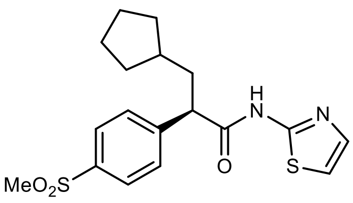

An oven-dried vial was charged with *tert*-butyl (*R*)-3-cyclopentyl-2-(4-(methylsulfonyl)phenyl)propanoate (0.1 mmol, 1.0 equiv) and CH_2Cl_2 (4 mL). TFA (1 mmol, 10 equiv) was added dropwise to the mixture at 0 °C, and the reaction was stirred at 25 ºC until TLC shows complete consumption of starting material. After the reaction was complete, the solvent was evaporated under reduced pressure. Then the crude acid was dissolved in anhydrous CH_2Cl_2 (2 mL), oxalyl chloride (0.11 mmol, 1.1 equiv) and one drop of DMF was added at 0 ºC. The reaction was stirred at 0 ºC for 10 min then room temperature for 1 h to obtain a solution of acid chloride. A solution of 2-thiazolamine (0.22 mmol, 2.2 equiv) and pyridine (0.22 mmol, 2.2 equiv) in anhydrous

THF (2 mL) was added. The reaction was stirred at 25 °C until TLC shows complete consumption of starting material. After purification by chromatography on silica gel (hexanes/EtOAc = 2:1), the title compound was obtained as a white solid in 75% yield (28.5 mg). m.p. = 78-79 °C; ^{1}H NMR (400 MHz, $CDCl_3$) δ 7.85 (d, J = 8.4 Hz, 2H), 7.58 – 7.50 (m, 3H), 7.09 (d, J = 3.7 Hz, 1H), 3.87 (t, J = 7.6 Hz, 1H), 3.02 (s, 3H), 2.27 (dt, J = 13.6, 7.6 Hz, 1H), 1.97 – 1.86 (m, 1H), 1.81 – 1.62 (m, 3H), 1.62 – 1.52 (m, 2H), 1.51 – 1.39 (m, 2H), 1.18 – 1.03 (m, 2H); ^{13}C NMR (101 MHz, $CDCl_3$) δ 170.9, 160.0, 144.9, 139.7, 135.4, 128.9, 127.9, 114.3, 52.0, 44.4, 40.0, 37.8, 32.7, 32.4, 25.0. IR (film): ν (cm^{-1}) 3161, 2943, 2912, 2867, 2849, 1687, 1544, 1305, 1167, 1147, 1089, 955, 770, 719, 544, 524; HR-MS (ESI) m/z calcd for $C_{18}H_{23}N_2O_3S_2^+$ [M+H$^+$] 379.11446, found 379.11478; $[\alpha]_D^{24.0}$ = -68.0 (c = 0.1, $CHCl_3$); HPLC conditions: OD-H column, hexane/2-propanol = 85/15, flow rate = 1.0 mL/min, λ = 230 nm, t_R = 13.8 min (minor), t_R = 18.8 min (major), 97:3 er.

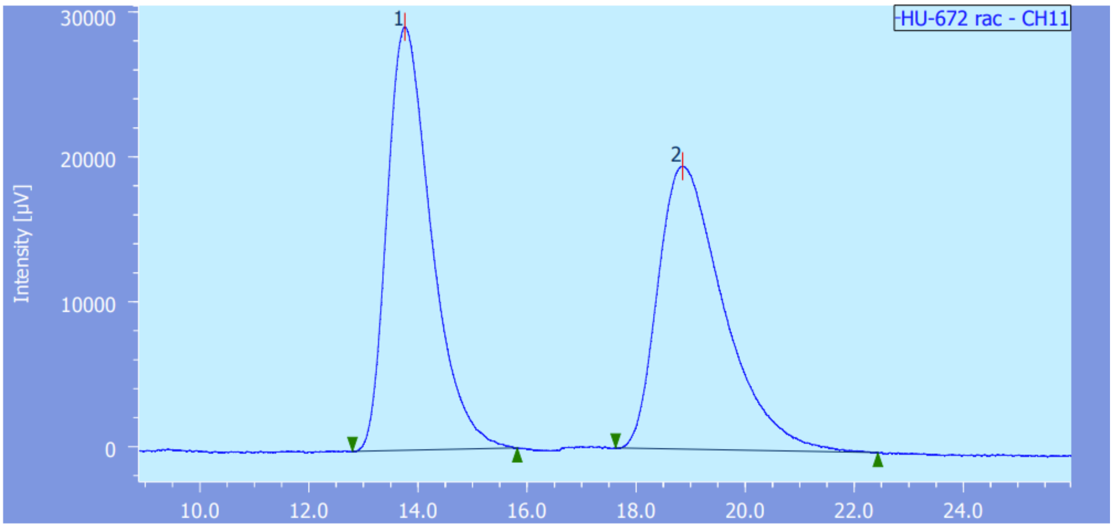

Decision

#	Peak Name	CH	tR [min]	Area [µV·sec]	Height [µV]	Area%	Height%	Quantity	NTP	Resolution	Symmetry Factor	Warning
1	Unknown	11	13.757	1649203	29203	50.201	59.939	N/A	1438	2.841	1.463	
2	Unknown	11	18.850	1635970	19518	49.799	40.061	N/A	1237	N/A	1.598	

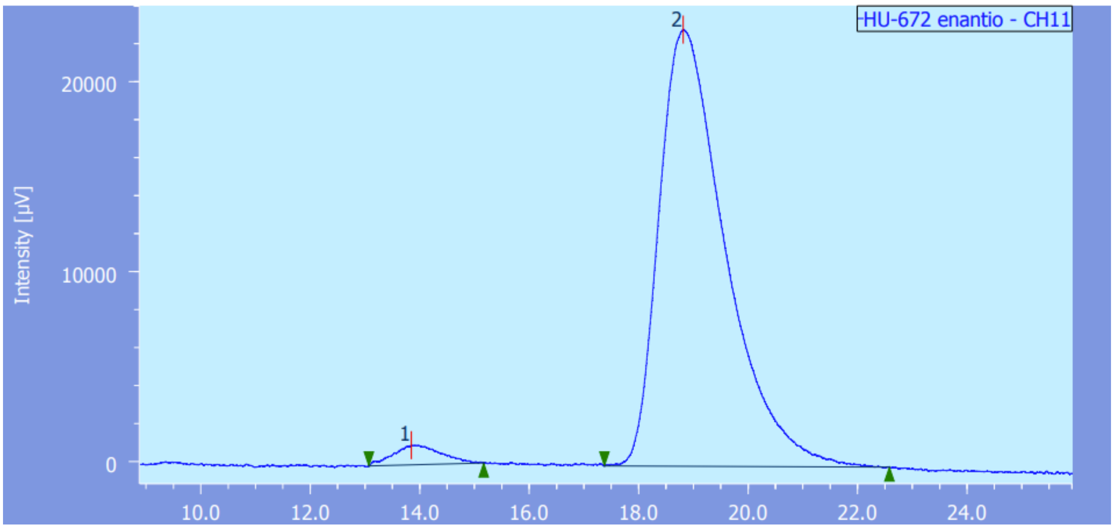

Decision

#	Peak Name	CH	tR [min]	Area [µV·sec]	Height [µV]	Area%	Height%	Quantity	NTP	Resolution	Symmetry Factor	Warning
1	Unknown	11	13.843	63761	1045	3.192	4.351	N/A	1187	2.659	1.345	
2	Unknown	11	18.813	1933655	22981	96.808	95.649	N/A	1236	N/A	1.648	

Experimental Procedures for Radical Trapping Experiment

General Procedure D (GP-D):

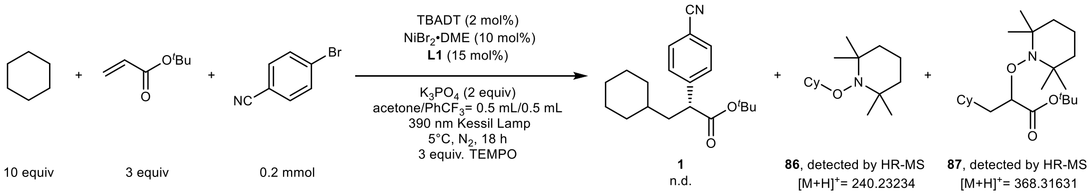

GP-D: In a nitrogen-filled glove box, a reaction vial (5 mL) equipped with a stirring bar was charged with tetrabutylammonium decatungstate (TBADT, 0.004 mmol, 2 mol%), $NiBr_2$·DME (0.02 mmol, 10 mol%), (4*S*,4'*S*)-4,4'-di((*S*)-*sec*-butyl)-1,1'-bis(3-(*tert*-butyl)phenyl)-4,4',5,5'-tetrahydro-1H,1'H-2,2'-biimidazole (**L1**, 0.03 mmol, 15 mol%), anhydrous K_3PO_4 (0.4 mmol, 2 equiv), 4-bromobenzonitrile (0.2 mmol, 1 equiv), 2,2,6,6-tetramethyl-1-piperidinyloxy (TEMPO, 0.6 mmol, 3 equiv), cyclohexane (2 mmol, 10 equiv), *tert*-butyl acrylate (0.6 mmol, 3 equiv), dry acetone (0.5 mL), and dry α,α,α-trifluorotoluene (0.5 mL). The vial was sealed and removed from the glovebox. The reaction mixture was pre-stirred for 20 min, then irradiated with a Kessil® PR160-390nm lamp at 5 °C with a distance of ~3 cm from the surface of the reaction vial. After 18 h of irradiation, the resulting mixture was passed through a pipette plug of Celite/silica gel and eluted with EtOAc. After concentration under reduced pressure, the mixture was analyzed by HR-MS. It was found that the reaction was completely suppressed by adding TEMPO (3.0 equiv) and the TEMPO-adducts **86** and **87** were detected by HR-MS (Supplementary Figure 1), which suggests that a carbon radical derived from the cyclohexane is generated under the applied conditions.

Experimental Procedures for Radical Clock Experiment

General Procedure E (GP-E):

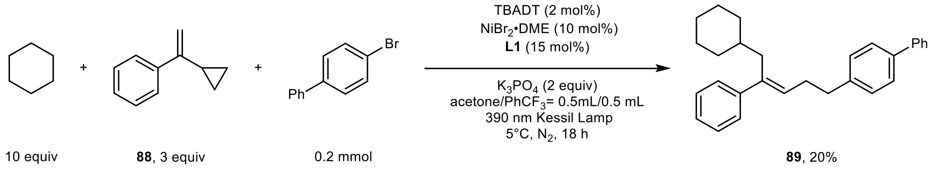

GP-E: In a nitrogen-filled glove box, a reaction vial (5 mL) equipped with a stirring bar was charged with tetrabutylammonium decatungstate (TBADT, 0.004 mmol, 2 mol%), $NiBr_2$·DME (0.02 mmol, 10 mol%), (4*S*,4'*S*)-4,4'-di((*S*)-*sec*-butyl)-1,1'-bis(3-(*tert*-butyl)phenyl)-4,4',5,5'-tetrahydro-1H,1'H-2,2'-biimidazole (**L1**, 0.03 mmol, 15 mol%), anhydrous K_3PO_4 (0.4 mmol, 2 equiv), 4-bromo-1,1'-biphenyl (0.2 mmol, 1 equiv), cyclohexane (2 mmol, 10 equiv), (1-cyclopropylvinyl)benzene (0.6 mmol, 3 equiv), dry acetone (0.5 mL), and dry α,α,α-trifluorotoluene (0.5 mL). The vial was sealed and removed from the glovebox. The reaction mixture was pre-stirred for 20 min, then irradiated with a Kessil® PR160-390nm lamp at 5 °C with a distance of ~3 cm from the surface of the reaction vial. After 18 h of irradiation, the resulting mixture was passed through a pipette plug of Celite/silica gel and eluted with ethyl acetate. After concentration under reduced pressure, the crude mixture was purified by chromatography on silica gel to give the corresponding product **89**.

Synthesis and Characterization of Aryl–Ni(II) Bromide Complex 90

General Procedure F (GP-F):

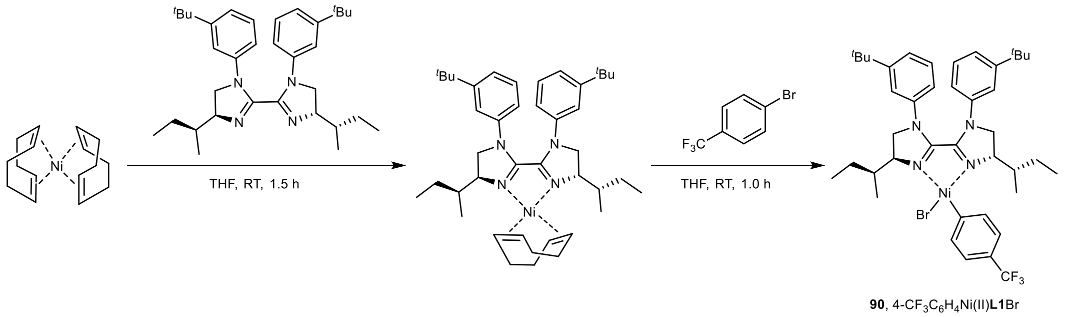

GP-F: A flame dried 10 mL Schlenk flask equipped with a stirring bar was charged with Ni(COD)$_2$ (0.50 mmol, 1 equiv) in a N_2-filled glovebox. (4*S*,4'*S*)-4,4'-di((*S*)-*sec*-Butyl)-1,1'-bis(3-(*tert*-butyl)phenyl)-4,4',5,5'-tetrahydro-1H,1'H-2,2'-biimidazole (**L1**, 0.50 mmol, 1.0 equiv) was added under a flow of N_2, and the solids were dissolved in 5 mL of dry THF. The resulting solution was stirred for 1.5 h at 25 °C. After this time, the mixture was cooled to -35 °C in an acetonitrile/dry ice bath, and 1-bromo-4-(trifluoromethyl)benzene (0.6 mmol, 1.2 equiv) was added via a microsyringe. The resulting mixture was stirred at 25 °C for 1.0 h, and partially THF was removed under high vacuum (approximately 0.5 mL THF should remain in the system). The complex was precipitated by adding dry pentane (8 mL) and then collected by filtration and washed thoroughly with dry pentane (2 x 8 mL) under a N_2 atmosphere.The solid was dried under high vacuum to give 4-$CF_3C_6H_4$Ni(II)**L1**Br **90** as a brown powder (52%, 208 mg). The complex **90** is not sensitive to air, but it degrades slowly in the presence of moisture, and thus was stored in the glovebox at −30 °C. Attempts to obtain crystals suitable for X-ray crystallography were unsuccessful.

*Note: Similar to previous reports,[1] the complex **90** is paramagnetic, ^1H and ^{13}C showed signal broadening.*

EI calcd for $[C_{41}H_{54}BrF_3N_4Ni]^+$ $[M]^+$ 796.28319, found 796.3; HR-MS (EI) m/z calcd for $C_{34}H_{50}BrN_4Ni$ [M-$CF_3C_6H_4$] 651.25723, found 651.25595; IR (film): ν (cm^{-1}) 2960, 2905, 2873, 1600, 1582, 1524, 1489, 1452, 1428, 1394, 1320, 1303, 1279, 1240, 1152, 1109, 1092, 1068, 1010, 816, 789, 699.

*Note: The results for EI and HR-MS (EI) were from two independent measurements. The EI measurement of complex **90** was performed with a resolution of 2300. With this resolution, the masses cannot be measured to 5 decimal places, but the measurement is more sensitive. For HR-MS (EI) measurements with a mass accuracy of 5 decimal places at least a resolution of > 6000 was needed. With this resolution, the signals in the 796.3 range were no longer detectable, but those of the [M-$CF_3C_6H_4$] adduct.*

Stoichiometric Experiment with Aryl–Ni(II) Bromide Complex 90

General Procedure G (GP-G):

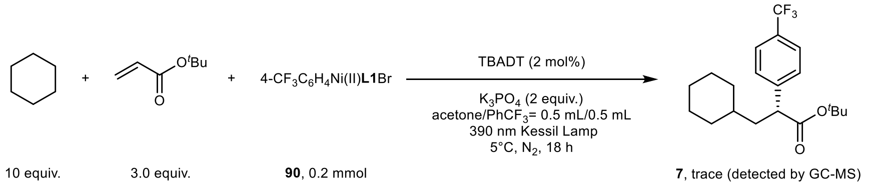

GP-G: In a nitrogen-filled glove box, a reaction vial (5 mL) equipped with a stirring bar was charged

with tetrabutylammonium decatungstate (TBADT, 0.004 mmol, 2 mol%), 4-$CF_3C_6H_4$Ni(II)**L1**Br **90** (0.2 mmol, 1.0 equiv), anhydrous K_3PO_4 (0.4 mmol, 2 equiv), cyclohexane (2 mmol, 10 equiv), *tert*-butyl acrylate (0.6 mmol, 3 equiv), dry acetone (0.5 mL), and dry α,α,α-trifluorotoluene (0.5 mL). The vial was sealed and removed from the glovebox. The reaction mixture was pre-stirred for 20 min, then irradiated with a Kessil® PR160-390nm lamp at 5 °C with a distance of ~3 cm from the surface of the reaction vial. After 18 h of irradiation, the resulting mixture was passed through a pipette plug of Celite/silica gel and eluted with EtOAc. After concentration under reduced pressure, the mixture was analyzed by GC-MS.

Catalytic Experiment with Aryl–Ni(II) Bromide Complex 90

General Procedure H (GP-H):

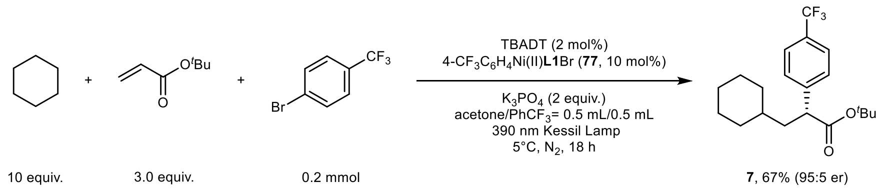

GP-H: In a nitrogen-filled glove box, a reaction vial (5 mL) equipped with a stirring bar was charged with tetrabutylammonium decatungstate (TBADT, 0.004 mmol, 2 mol%), 4-$CF_3C_6H_4$Ni(II)**L1**Br **90** (0.01 mmol, 10 mol%), anhydrous K_3PO_4 (0.4 mmol, 2 equiv), 1-bromo-4-(trifluoromethyl)benzene (0.2 mmol, 1.0 equiv), cyclohexane (2 mmol, 10 equiv), *tert*-butyl acrylate (0.6 mmol, 3 equiv), dry acetone (0.5 mL), and dry α,α,α-trifluorotoluene (0.5 mL). The vial was sealed and removed from the glovebox. The reaction mixture was pre-stirred for 20 min, then irradiated with a Kessil® PR160-390nm lamp at 5 °C with a distance of ~3 cm from the surface of the reaction vial. After 18 h of irradiation, the resulting mixture was passed through a pipette plug of Celite/silica gel and eluted with EtOAc. After concentration under reduced pressure, the desired product was obtained as a white solid in 67% yield and 95:5 er.

General Procedures for Laser Flash Photolysis

Laser flash photolysis (LFP) experiments were performed to study the decay of the reactive excited state of decatungstate (TBADT*) in the presence of increasing concentrations of cyclohexane, *tert*-butyl acrylate and 4-bromobenzonitrile at 780 nm. The decay of the excited state of the decatungstate was observed in the presence of cyclohexane following linear Stern-Volmer behavior. In contrast, the decay of the reactive excited state of decatungstate by *tert*-butyl acrylate and 4-bromobenzonitrile could not be observed. The quenching constants for cyclohexane was obtained through relevant Stern-Volmer plots, and a bimolecular rate constant $k_Q = 3.14\times10^7\ M^{-1}\cdot s^{-1}$ was calculated.

Nanosecond kinetics were studied using an Edinburgh Instruments LP 920 transient absorption setup. The probe beam, derived from a Xe Arc Lamp, was pulsed with a duration of 10 ms and attenuated with a long pass filter (Schott RG 610) before passing through the sample to minimize the sample's direct exposure to the probe light. The pump light pulses of 355 nm were obtained by third harmonic generation of the 1064 nm light from a Continuum Surelight Nd: YAG laser source. The excitation beam was overlapped in the sample at a 90° angle with respect to the probe light. A second long pass filter (Schott RG 1- cutoff also at 610 nm) placed behind the sample cuvette prevented scattered pump light from reaching the photomultiplier detector. The laser system was operated at a repetition rate of 10 Hz with a pulse duration of 10 ns. The excitation energy before

the sample was ~4.0 mJ /pulse. A limited number of shots i.e., 16 averaged scans, minimized permanent changes of the sample.

The starting solutions of TBADT $(^nBu_4N)_4[W_{10}O_{32}]$ in CH_3CN had a concentration of 2.0×10^{-4} M and an optical density of 0.62 at 355 nm. The sample solutions were prepared in the glove box and sealed to prevent any contact with oxygen in a 1×1 cm quartz cell which was excited with single pulses from the laser. The resulting changes in absorption at 780 nm (Δλ= 10 nm) were recorded. The lifetimes of the reactive transient TBADT* were determined by fitting the obtained decay profiles using the first-order equation (1) specified below.

$$y = y^0 + A \bullet e^{-x/\tau} \qquad (1)$$

From decays reported in Supplementary Figure 2, it is possible to derive the Stern-Volmer plots shown in Supplementary Figure 3 following the equation (2):

$$\tau_0/\tau = 1 + \overbrace{k_Q \bullet \tau_0}^{b} \bullet [Q] \qquad (2)$$

$$\tau_0 = 50.06\times10^{-9}\ \text{s} \qquad k_Q = 3.14\times10^7\ \text{M}^{-1}\cdot\text{s}^{-1}$$

General Procedures for Cyclic Voltammetry Studies

Cyclic voltammetry experiments were performed in a three-electrode cell connected to nitrogen at room temperature. A working glass carbon electrode, platinum wire counter electrode, and Ag|AgCl reference electrode were employed. Anhydrous degassed CH_3CN (5 mL) containing 1.0 mmol $^nBu_4NBF_4$ were poured into the electrochemical cell in all experiments. The concentration of compounds is 3 mM. The scan rate is 100 mV/s. All cyclic voltammograms were normalized by adding 1.0 equiv freshly-sublimed ferrocene and collecting a new voltammogram. The ½ wave potential of the Fc/Fc+ peak was identified and set to 0.0 V. Data was analyzed by subtracting a background current prior to identifying the maximum current (Cp) and determining the potential (Ep/2) at half this value (Cp/2).[2] As shown in Supplementary Figure 10, two reductive peaks of **L1**$NiBr_2$ were observed at Ni^{II}/Ni^{I} = -1.44 V (*vs* Fc/Fc^+) and Ni^{I}/Ni^{0} = -1.82 V (*vs* Fc/Fc^+), and the reductive half-peak potential of **L1**$NiBr_2$ was measured as Ni^{II}/Ni^{I} = -1.25 V (*vs* Fc/Fc^+) and Ni^{I}/Ni^{0} = -1.74 V (*vs* Fc/Fc^+). Two reductive peaks of TBADT were observed at $[W_{10}O_{32}]^{4-}/[W_{10}O_{32}]^{5-}$ = -1.37 V (*vs* Fc/Fc^+) and $[W_{10}O_{32}]^{5-}/[W_{10}O_{32}]^{6-}$ = -1.93 V (*vs* Fc/Fc^+), and the reductive half-peak potential of TBADT was measured as $[W_{10}O_{32}]^{4-}/[W_{10}O_{32}]^{5-}$ = -1.28 V (*vs* Fc/Fc^+) and $[W_{10}O_{32}]^{5-}/[W_{10}O_{32}]^{6-}$ = -1.85 V (*vs* Fc/Fc^+).

General Procedures for X-Ray Diffraction Analysis of Compound 1

The crystal was growth from EtOAc/*n*-hexane by liquid diffusion. Single crystal X-ray diffraction data were collected at 160(1) K on a Rigaku OD Synergy-Hypix diffractometer using the copper X-ray radiation (l = 1.54184 Å) from a dual wavelength X-ray source and an Oxford Instruments Cryojet XL cooler. The selected suitable single crystal was mounted using polybutene oil on a flexible loop fixed on a goniometer head and immediately transferred to the diffractometer. Pre-experiment, data collection, data reduction and analytical absorption correction[3] were performed with the program suite *CrysAlisPro*.[4] Using *Olex2*,[5] the structure was solved with the *SHELXT*[6] small molecule structure solution program and refined with the *SHELXL* program package[7] by full-

matrix least-squares minimization on F^4. *PLATON*[8] was used to check the result of the X-ray analysis. For more details about the data collection and refinement parameters, see the CIF file. **(**Deposition Number 2268422). The title chiral compound crystallizes in the chiral space group P212121. The configuration at the C1 carbon atom is R.

Crystal Data for $C_{20}H_{27}NO_2$ (*M* =313.42 g/mol): orthorhombic, space group $P2_12_12_1$ (no. 19), a = 5.56013(5) Å, b = 9.55435(7) Å, c = 34.0101(3) Å, V = 1806.74(3) Å^3, Z = 4, T = 160(1) K, μ(Cu Kα) = 0.575 mm^{-1}, *Dcalc* = 1.152 g/cm^3, 25290 reflections measured (5.196° ≤ 2Θ ≤ 154.59°), 3809 unique (R_{int} = 0.0274, R_{sigma} = 0.0140) which were used in all calculations. The final R_1 was 0.0270 (I > 2σ(I)) and wR_2 was 0.0726 (all data).

Refinement model description

Number of restraints - 0, number of constraints - unknown.

Details:

1. Fixed Uiso

At 1.2 times of:

All C(H) groups, All C(H,H) groups

At 1.5 times of

All C(H,H,H) groups

2. a Ternary CH refined with riding coordinates:

C1(H1), C8(H8)

2. b Secondary CH2 refined with riding coordinates:

C7(H7A,H7B), C9(H9A,H9B), C10(H10A,H10B), C11(H11A,H11B), C12(H12A,H12B), C13(H13A,H13B)

2. c Aromatic/amide H refined with riding coordinates:

C15(H15), C16(H16), C18(H18), C19(H19)

2. d Idealised Me refined as rotating group:

C4(H4A,H4B,H4C), C5(H5A,H5B,H5C), C6(H6A,H6B,H6C)

This report has been created with Olex2, compiled on 2022.04.12 svn.rca3783a0 for Rigaku Oxford Diffraction

Supplementary Discussion

<u>General Procedure I (GP-I): Evidence for excluding the process of product deracemization.</u>

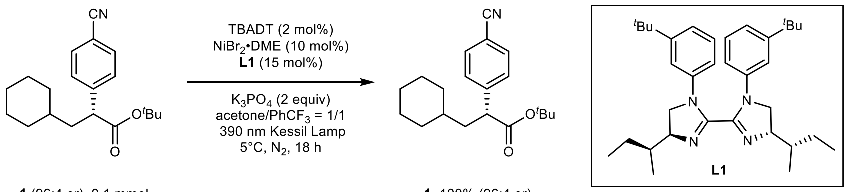

1 (96:4 er), 0.1 mmol **1**, 100% (96:4 er)

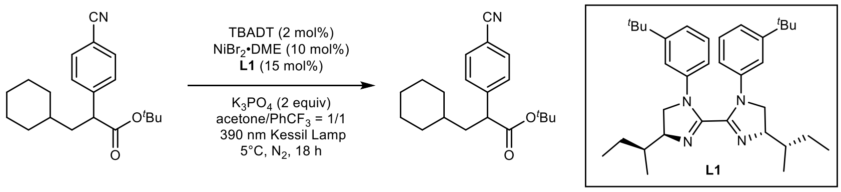

***Rac*-1** (50:50 er), 0.1 mmol ***Rac*-1**, 100% (50:50 er)

GP-I: In a nitrogen-filled glove box, a reaction vial (5 mL) equipped with a stirring bar was charged with tetrabutylammonium decatungstate (TBADT, 0.002 mmol, 2 mol%), NiBr$_2$·DME (0.01 mmol, 10 mol%), (4*S*,4'*S*)-4,4'-di((*S*)-*sec*-butyl)-1,1'-bis(3-(*tert*-butyl)phenyl)-4,4',5,5'-tetrahydro-1H,1'H-2,2'-biimidazole (**L1**, 0.015 mmol, 15 mol%), anhydrous K$_3$PO$_4$ (0.2 mmol, 2 equiv), *tert*-butyl (R)-2-(4-cyanophenyl)-3-cyclohexylpropanoate (**1**, 0.1 mmol, 1 equiv) or *tert*-butyl 2-(4-cyanophenyl)-3-cyclohexylpropanoate (***Rac*-1**, 0.1 mmol, 1 equiv), dry acetone (0.5 mL), and dry α,α,α-trifluorotoluene (0.5 mL). The vial was sealed and removed from the glovebox. The reaction mixture was pre-stirred for 20 min, then irradiated with a Kessil® PR160-390nm lamp at 5 °C with a distance of ~3 cm from the surface of the reaction vial. After 18 h of irradiation, the resulting mixture was passed through a pipette plug of Celite/silica gel and eluted with EtOAc. After concentration under reduced pressure, the crude mixture was purified by chromatography on silica gel.

<u>General Procedure J (GP-J): Evidence for excluding the process of Giese addition followed by enantioselective *alpha*-arylation.</u>

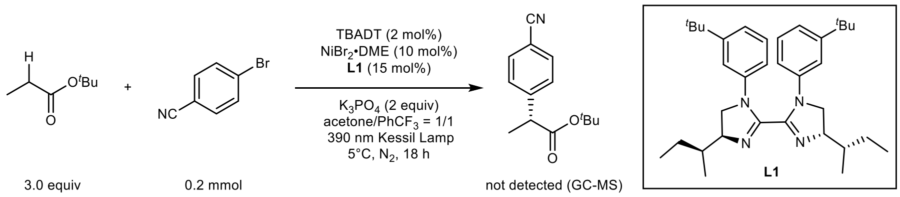

3.0 equiv 0.2 mmol not detected (GC-MS)

GP-J: In a nitrogen-filled glove box, a reaction vial (5 mL) equipped with a stirring bar was charged with tetrabutylammonium decatungstate (TBADT, 0.004 mmol, 2 mol%), NiBr$_2$·DME (0.02 mmol, 10 mol%), (4*S*,4'*S*)-4,4'-di((*S*)-*sec*-butyl)-1,1'-bis(3-(*tert*-butyl)phenyl)-4,4',5,5'-tetrahydro-1H,1'H-2,2'-biimidazole (**L1**, 0.03 mmol, 15 mol%), anhydrous K$_3$PO$_4$ (0.4 mmol, 2 equiv), *tert*-butyl propionate (0.2 mmol, 1 equiv), 4-bromobenzonitrile, dry acetone (0.5 mL), and dry α,α,α-trifluorotoluene (0.5 mL). The vial was sealed and removed from the glovebox. The reaction mixture was pre-stirred for 20 min, then irradiated with a Kessil® PR160-390nm lamp at 5 °C with a distance of ~3 cm from the surface of the reaction vial. After 18 h of irradiation, the resulting mixture was passed through a pipette plug of Celite/silica gel and eluted with EtOAc. After concentration under reduced pressure, the mixture was analyzed by GC-MS.

General Procedure K (GP-K): Evidence for excluding the process of atom transfer radical addition (ATRA) followed by asymmetric Ni-catalyzed aryl cross coupling.

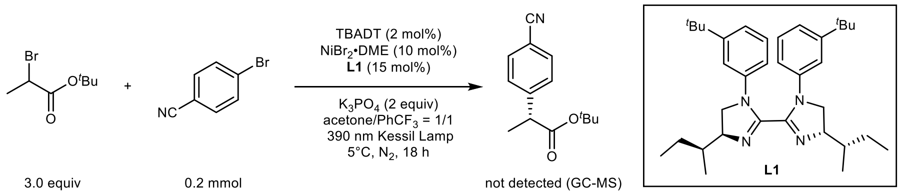

GP-K: In a nitrogen-filled glove box, a reaction vial (5 mL) equipped with a stirring bar was charged with tetrabutylammonium decatungstate (TBADT, 0.004 mmol, 2 mol%), $NiBr_2$·DME (0.02 mmol, 10 mol%), (4*S*,4'*S*)-4,4'-di((*S*)-*sec*-butyl)-1,1'-bis(3-(*tert*-butyl)phenyl)-4,4',5,5'-tetrahydro-1H,1'H-2,2'-biimidazole (**L1**, 0.03 mmol, 15 mol%), anhydrous K_3PO_4 (0.4 mmol, 2 equiv), *tert*-butyl 2-bromopropionate (0.2 mmol, 1 equiv), 4-bromobenzonitrile, dry acetone (0.5 mL), and dry α,α,α-trifluorotoluene (0.5 mL). The vial was sealed and removed from the glovebox. The reaction mixture was pre-stirred for 20 min, then irradiated with a Kessil® PR160-390nm lamp at 5 °C with a distance of ~3 cm from the surface of the reaction vial. After 18 h of irradiation, the resulting mixture was passed through a pipette plug of Celite/silica gel and eluted with EtOAc. After concentration under reduced pressure, the mixture was analyzed by GC-MS.

Supplementary Tables

Supplementary Table 1. Effect of chiral ligands on cyclohexane radical precursor[a]

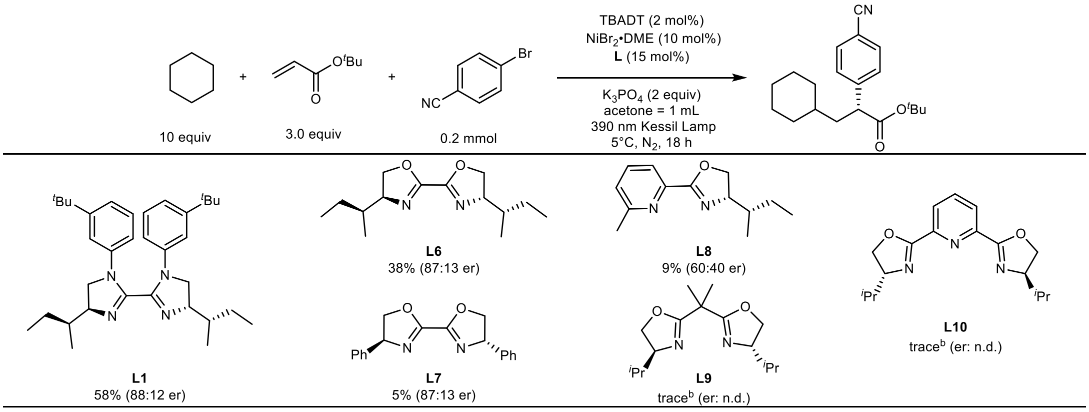

[a]Reactions conditions: cyclohexane (2 mmol, 10 equiv), 4-bromobenzonitrile (0.2 mmol, 1 equiv), *tert*-butyl acrylate (0.6 mmol, 3 equiv), TBADT (0.004 mmol, 2 mol %), $NiBr_2$•DME (0.02 mmol, 10 mol%), Ligand (0.03 mmol, 15 mol%), K_3PO_4 (0.4 mmol, 2 equiv), acetone = 1.0 mL, 40 W 390 nm Kessil Lamp, N_2, 5 °C, 18 h. Isolated yields were shown. The enantiomeric ratio (er) values were determined by chiral HPLC. [b]Detected by GC-MS. n.d. = not determined.

Supplementary Table 2. Effect of solvent on cyclohexane radical precursor[a]

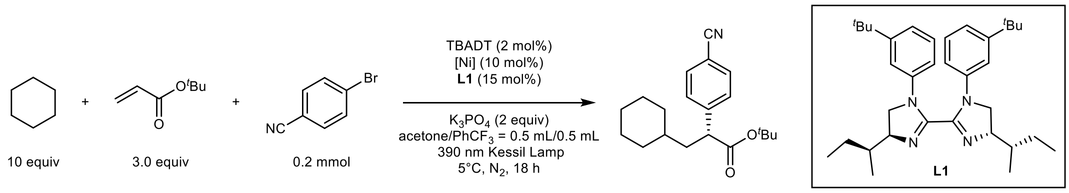

Entry	solvent	yield (%)	er
1	acetone = 1.0 mL	58	88:12
2	CH_3CN = 1.0 mL	30	67:33
3	$PhCF_3$ = 1.0 mL	N.D.[b]	--
4	acetone/$PhCF_3$ = 0.5 mL/0.5 mL	80	96:4
5	acetone/EtOAc = 0.5 mL/0.5 mL	N.D.[b]	--
6	CH_3CN/$PhCF_3$ = 0.5 mL/0.5 mL	62	85:15

[a]Reactions conditions: cyclohexane (2 mmol, 10 equiv), 4-bromobenzonitrile (0.2 mmol, 1 equiv), *tert*-butyl acrylate (0.6 mmol, 3 equiv), TBADT (0.004 mmol, 2 mol %), $NiBr_2$•DME (0.02 mmol, 10 mol%), **L1** (0.03 mmol, 15 mol%), K_3PO_4 (0.4 mmol, 2 equiv), solvent, 40 W 390 nm Kessil Lamp, N_2, 5 °C, 18 h. Isolated yields were shown. The enantiomeric ratio (er) values were determined by chiral HPLC. [b]Detected by GC-MS. N.D.= not detected.

Supplementary Table 3. Effect of nickel sources on cyclohexane radical precursor[a]

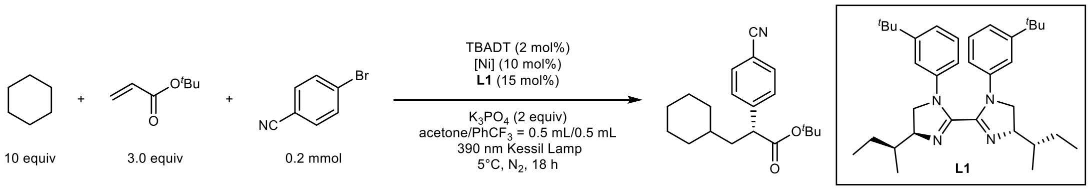

Entry	[Ni]	yield (%)	er
1	$NiBr_2$•DME	80	96:4
2	$NiCl_2$•DME	64	87:13
3	$NiBr_2$•diglyme	72	95:5
4	$NiCl_2(Py)_4$	53	66:34
5	$NiBr_2$•$3H_2O$	76	94:6
6	$NiBr_2$-**L1** complex	70	90:10

[a]Reactions conditions: cyclohexane (2 mmol, 10 equiv), 4-bromobenzonitrile (0.2 mmol, 1 equiv), *tert*-butyl acrylate (0.6 mmol, 3 equiv), TBADT (0.004 mmol, 2 mol %), $NiBr_2$•DME (0.02 mmol, 10 mol%), **L1** (0.03 mmol, 15 mol%), K_3PO_4 (0.4 mmol, 2 equiv), acetone/$PhCF_3$ = 0.5 mL/0.5 mL, 40 W 390 nm Kessil Lamp, N_2, 5 °C, 18 h. Isolated yields were shown. The enantiomeric ratio (er) values were determined by chiral HPLC.

Supplementary Table 4. Effect of nickel/ligand ratio and loading on cyclohexane radical precursor[a]

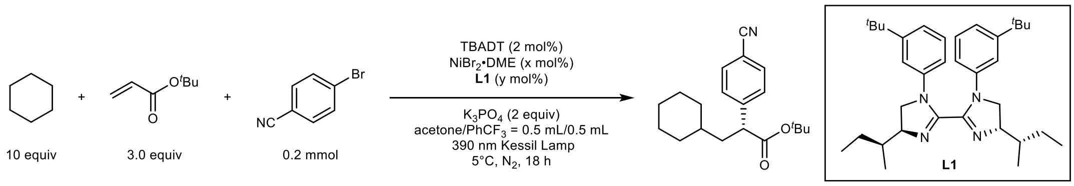

Entry	x/y	yield (%)	er
1	10/15	80	96:4
2	10/12	75	95:5
3	5/7.5	68	94:6
4	15/22.5	78	96:4

[a]Reactions conditions: cyclohexane (2 mmol, 10 equiv), 4-bromobenzonitrile (0.2 mmol, 1 equiv), *tert*-butyl acrylate (0.6 mmol, 3 equiv), TBADT (0.004 mmol, 2 mol %), $NiBr_2$•DME (x mol%), **L1** (y mol%), K_3PO_4 (0.4 mmol, 2 equiv), acetone/$PhCF_3$ = 0.5 mL/0.5 mL, 40 W 390 nm Kessil Lamp, N_2, 5 °C, 18 h. Isolated yields were shown. The enantiomeric ratio (er) values were determined by chiral HPLC.

Supplementary Table 5. Effect of chiral biimidazole ligands in $PhCF_3$/acetone on cyclohexane radical precursor[a]

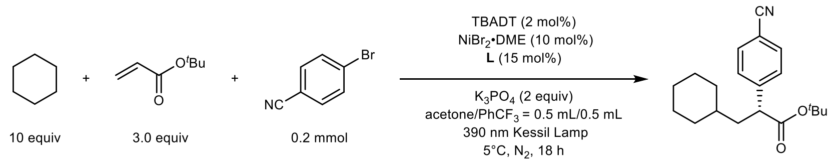

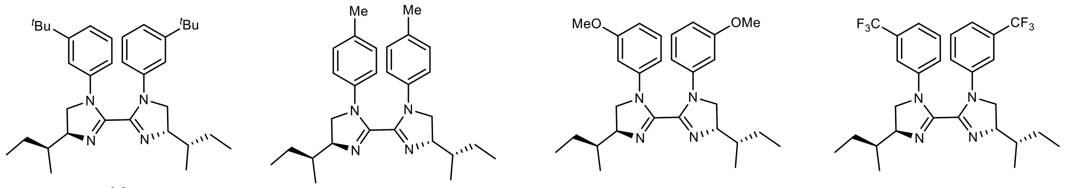

L1 80% (96:4 er) 71% (96:4 er)[c]

L2 81% (94:6 er)

L3 80% (90:10 er)

L4 86% (86:14 er)

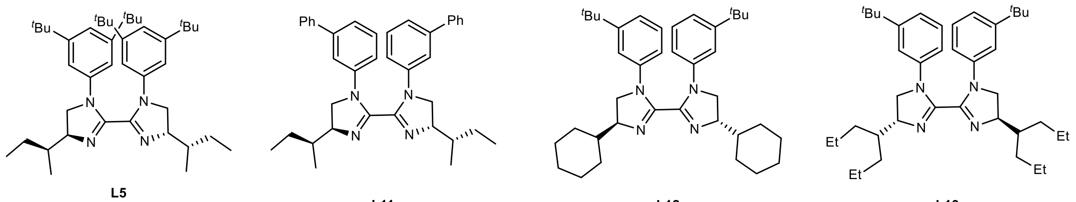

L5 60% (97:3 er) 68% (97:3 er)[c]

L11 trace[b] (er: n.d.)

L12 60% (92:8 er)

L13 56% (16:84 er)

[a]Reactions conditions: cyclohexane (2 mmol, 10 equiv), 4-bromobenzonitrile (0.2 mmol, 1 equiv), *tert*-butyl acrylate (0.6 mmol, 3 equiv), TBADT (0.004 mmol, 2 mol %), $NiBr_2$•DME (0.02 mmol, 10 mol%), Ligand (0.03 mmol, 15 mol%), K_3PO_4 (0.4 mmol, 2 equiv), acetone/$PhCF_3$ = 0.5 mL/0.5 mL, 40 W 390 nm Kessil Lamp, N_2, 5 °C, 18 h. Isolated yields were shown. The enantiomeric ratio (er) values were determined by chiral HPLC. [b]Detected by GC-MS. n.d. = not determined. [c]Acetone/$PhCF_3$ = 1.0 mL/1.0 mL was used.

Supplementary Table 6. Effect of the amount of cyclohexane[a]

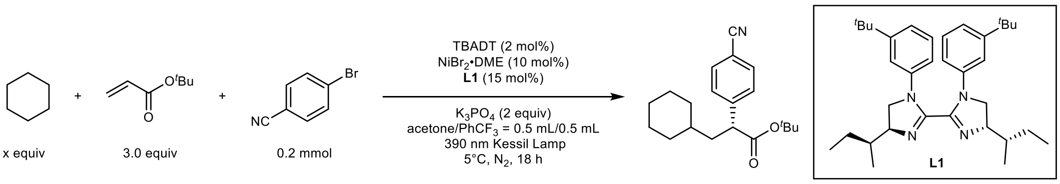

Entry	x equiv	yield (%)	er
1	5	61	95:5
2	10	80	96:4
3	15	75	96:4

[a]Reactions conditions: cyclohexane (x equiv), 4-bromobenzonitrile (0.2 mmol, 1 equiv), *tert*-butyl acrylate (0.6 mmol, 3 equiv), TBADT (0.004 mmol, 2 mol %), $NiBr_2$•DME (0.02mmol, 10 mol%), **L1** (0.03 mmol, 15 mol%), K_3PO_4 (0.4 mmol, 2 equiv), acetone/$PhCF_3$ = 0.5 mL/0.5 mL, 40 W 390 nm Kessil Lamp, N_2, 5 °C, 18 h. Isolated yields were shown. The enantiomeric ratio (er) values were determined by chiral HPLC.

Supplementary Table 7. Control experiments for cyclohexane radical precursor[a]

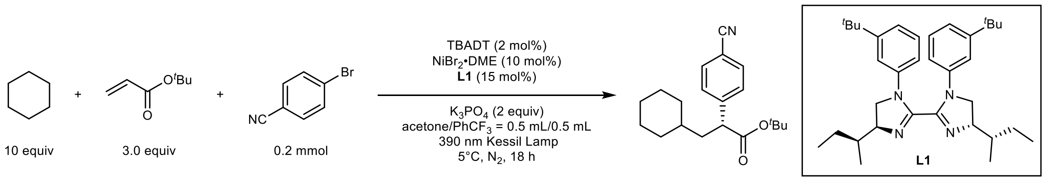

Entry	variation from above conditions	yield (%)	er
1	none	80	96:4
2	w/o light	N.D.[b]	--
3	w/o TBADT	N.D.[b]	--
4	w/o $NiBr_2$•DME	N.D.[b]	--
5	w/o **L1**	N.D.[b]	--

[a]Reactions conditions: cyclohexane (10 equiv), 4-bromobenzonitrile (0.2 mmol, 1 equiv), *tert*-butyl acrylate (0.6 mmol, 3 equiv), TBADT (0.004 mmol, 2 mol %), $NiBr_2$•DME (0.02mmol, 10 mol%), **L1** (0.03 mmol, 15 mol%), K_3PO_4 (0.4 mmol, 2 equiv), acetone/$PhCF_3$ = 0.5 mL/0.5 mL, 40 W 390 nm Kessil Lamp, N_2, 5 °C, 18 h. Isolated yields were shown. The enantiomeric ratio (er) values were determined by chiral HPLC. [b]Detected by GC-MS. N.D. = not detected.

Supplementary Table 8. Effect of photocatalyst, ligand, base and solvent on isopropanol radical precursor[a]

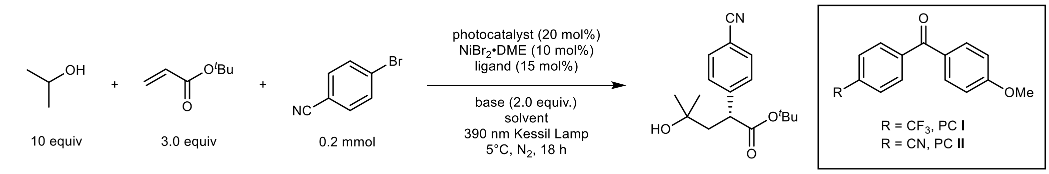

Entry	photocatalyst	ligand	base	solvent	yield (%)	er
1	PC **I**	**L1**	K_3PO_4	$PhCF_3$/acetone= 0.5 mL/0.5 mL	trace[b]	--
2	PC **I**	**L1**	Na_2CO_3	$PhCF_3$/acetone= 0.5 mL/0.5 mL	44	93:7
3	PC **I**	**L1**	Na_2CO_3	$PhCF_3$/acetone= 1.0 mL/1.0 mL	80	91:9
4	PC **I**	**L5**	Na_2CO_3	$PhCF_3$/acetone= 1.0 mL/1.0 mL	85	96:4
5	PC **I**	**L5**	Na_2CO_3	EtOAc= 2.0 mL	81	94:6
6	PC **II**	**L5**	Na_2CO_3	$PhCF_3$/acetone= 1.0 mL/1.0 mL	85	94:6

[a]Reactions conditions: isopropanol (10 equiv), 4-bromobenzonitrile (0.2 mmol, 1 equiv), *tert*-butyl acrylate (0.6 mmol, 3 equiv), photocatalyst (0.04 mmol, 20 mol %), $NiBr_2$•DME (0.02 mmol, 10 mol%), ligand (0.03 mmol, 15 mol%), base (0.4 mmol, 2 equiv), solvent, 40 W 390 nm Kessil Lamp, N2, 5 °C, 18 h. Isolated yield. Isolated yields were shown. The enantiomeric ratio (er) values were determined by chiral HPLC. [b]Detected by GC-MS.

Supplementary Table 9. Unsuccessful examples[a]

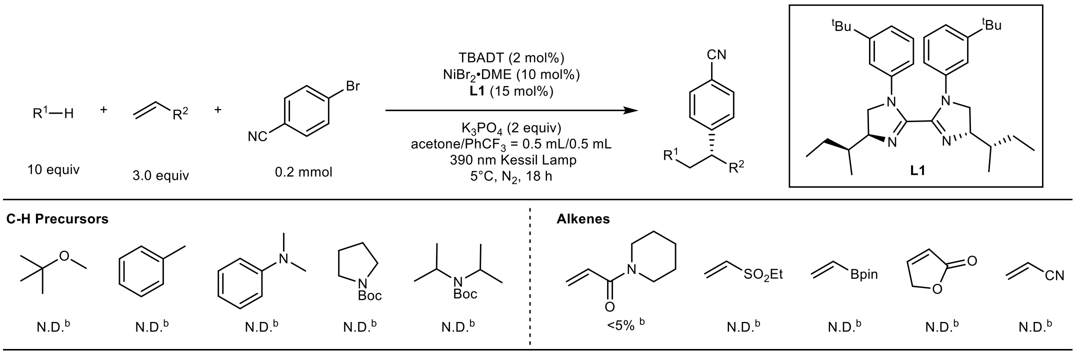

[a]Reactions conditions: C-H precursor (10 equiv), 4-bromobenzonitrile (0.2 mmol, 1 equiv), alkene (0.6 mmol, 3 equiv), TBADT (0.004 mmol, 2 mol %), $NiBr_2$•DME (0.02mmol, 10 mol%), **L1** (0.03 mmol, 15 mol%), K_3PO_4 (0.4 mmol, 2 equiv), acetone/$PhCF_3$ = 0.5 mL/0.5 mL, 40 W 390 nm Kessil Lamp, N_2, 5 °C, 18 h. [b]Detected by GC-MS. N.D. = not detected.

Supplementary Table 10. X-Ray structure and crystal data of compound **1**.

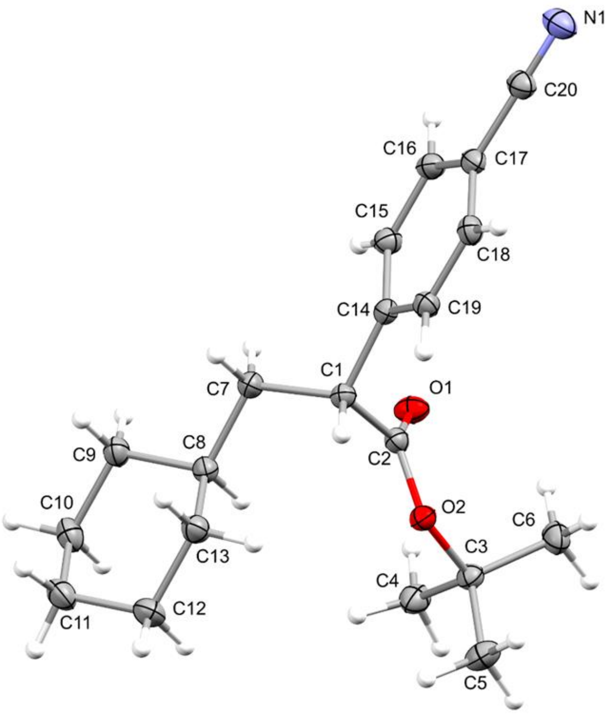

Crystal data and structure refinement for compound 1

Crystallised from	EtOAc / *n*-hexane
Identification code	xia2408
Empirical formula	$C_{20}H_{27}NO_2$
Formula weight	313.42
Temperature/K	160(1)
Crystal system	orthorhombic
Space group	$P2_12_12_1$
a/Å	5.56013(5)
b/Å	9.55435(7)
c/Å	34.0101(3)
α/°	90
β/°	90
γ/°	90
Volume/Å^3	1806.74(3)
Z	4
ρ_{calc}g/cm^3	1.152
μ/mm^{-1}	0.575
F(000)	680.0
Crystal size/mm^3	0.23 × 0.1 × 0.07
Radiation	Cu Kα (λ = 1.54184)
2Θ range for data collection/°	5.196 to 154.59
Index ranges	$-7 \leq h \leq 7$, $-12 \leq k \leq 9$, $-42 \leq l \leq 43$
Reflections collected	25290
Independent reflections	3809 [R_{int} = 0.0274, R_{sigma} = 0.0140]
Data/restraints/parameters	3809/0/212
Goodness-of-fit on F^2	1.020
Final R indexes [I>=2σ (I)]	R_1 = 0.0270, wR_2 = 0.0720
Final R indexes [all data]	R_1 = 0.0276, wR_2 = 0.0726
Largest diff. peak/hole / e Å^{-3}	0.14/-0.11
Flack parameter	-0.02(5)

Supplementary Table 11. Fractional Atomic Coordinates (×10^4) and Equivalent Isotropic Displacement Parameters (Å^2×10^3) for xia2408. U_{eq} is defined as 1/3 of the trace of the orthogonalised U_{IJ} tensor.

**Atom**	***x***	***y***	***z***	**U(eq)**
C1	6696(2)	6608.4(13)	3949.6(4)	28.0(3)
C2	4853(2)	6942.2(13)	3631.9(4)	28.3(3)
C3	4498(3)	7334.5(14)	2920.6(4)	31.3(3)
C4	2873(3)	6087.6(16)	2848.3(4)	41.4(4)
C5	6448(3)	7404(2)	2607.9(4)	44.4(4)
C6	3118(3)	8698.2(17)	2945.3(5)	44.7(4)
C7	6208(3)	5116.2(14)	4104.8(4)	32.1(3)
C8	6386(3)	3972.0(13)	3791.1(4)	30.4(3)
C9	5698(3)	2549.7(14)	3964.9(4)	35.9(3)
C10	5835(3)	1382.9(15)	3658.8(5)	41.7(3)
C11	8318(3)	1305.6(17)	3473.6(5)	45.3(4)
C12	9049(3)	2717.1(17)	3300.9(5)	43.8(4)
C13	8893(3)	3880.4(15)	3607.9(4)	36.7(3)
C14	6591(2)	7720.8(13)	4267.4(4)	27.8(3)
C15	4639(3)	7831.8(15)	4523.7(4)	32.6(3)
C16	4531(3)	8903.4(15)	4798.4(4)	34.0(3)
C17	6383(3)	9882.9(14)	4817.6(4)	31.5(3)
C18	8370(3)	9769.8(14)	4570.1(4)	32.9(3)
C19	8457(2)	8685.5(14)	4298.7(4)	30.8(3)
C20	6214(3)	11033.7(15)	5091.8(4)	35.9(3)
N1	6055(3)	11943.7(15)	5310.4(4)	48.0(3)
O1	2719.0(18)	7020.6(12)	3689.4(3)	38.6(3)
O2	5925.5(17)	7108.4(10)	3282.6(2)	30.1(2)

Supplementary Table 12. Anisotropic Displacement Parameters ($Å^2×10^3$) for xia2408. The Anisotropic displacement factor exponent takes the form: $-2\pi^2[h^2a^{*2}U_{11}+2hka^*b^*U_{12}+…]$.

**Atom**	**U_{11}**	**U_{22}**	**U_{33}**	**U_{23}**	**U_{13}**	**U_{12}**
C1	29.6(6)	27.4(6)	27.1(6)	-0.2(5)	2.0(5)	0.0(5)
C2	32.3(6)	24.4(6)	28.3(6)	-2.0(5)	2.0(5)	-0.8(5)
C3	35.7(7)	31.6(6)	26.5(6)	1.4(5)	-3.2(5)	0.0(6)
C4	50.6(9)	38.6(8)	34.8(7)	-0.6(6)	-5.6(7)	-9.0(7)
C5	42.3(8)	60.7(10)	30.1(7)	7.4(7)	0.2(6)	-1.4(8)
C6	55.6(10)	35.5(7)	43.0(8)	-0.2(6)	-11.3(7)	9.5(7)
C7	40.1(7)	27.7(6)	28.5(6)	1.1(5)	2.9(6)	1.0(6)
C8	34.9(7)	26.6(6)	29.6(6)	0.0(5)	-1.1(5)	2.1(6)
C9	40.7(8)	27.5(6)	39.5(7)	0.9(5)	3.3(6)	-0.1(6)
C10	45.6(8)	27.9(6)	51.5(9)	-4.3(6)	-4.2(7)	2.1(6)
C11	50.6(9)	35.0(7)	50.2(9)	-8.8(7)	-0.9(7)	9.5(7)
C12	48.8(9)	43.8(8)	38.9(8)	-6.1(6)	6.8(7)	8.2(7)
C13	39.2(7)	34.1(7)	36.9(7)	-2.6(6)	6.0(6)	0.1(6)
C14	31.6(6)	25.9(6)	25.8(6)	2.0(5)	-1.7(5)	2.6(5)
C15	34.1(7)	33.4(7)	30.2(6)	-0.5(5)	1.8(5)	-2.9(6)
C16	35.1(7)	39.0(7)	28.0(6)	-1.2(5)	3.3(5)	1.8(6)
C17	37.8(7)	29.4(6)	27.3(6)	-1.0(5)	-4.1(5)	5.8(6)
C18	34.4(7)	27.6(6)	36.5(7)	0.1(5)	-2.4(6)	-1.1(6)
C19	31.3(6)	29.3(6)	31.9(6)	0.4(5)	2.6(5)	1.3(5)
C20	37.6(7)	36.2(7)	33.7(7)	-3.6(6)	-3.6(6)	4.8(6)
N1	52.7(8)	46.4(7)	45.0(7)	-14.5(6)	-3.0(6)	7.6(7)
O1	30.4(5)	52.6(6)	32.9(5)	-2.8(5)	2.1(4)	1.8(5)
O2	31.3(5)	33.0(5)	26.0(4)	2.2(3)	0.1(4)	0.7(4)

Supplementary Table 13. Bond Lengths for xia2408.

Atom	Atom	Length/Å	Atom	Atom	Length/Å
C1	C2	1.5231(18)	C9	C10	1.527(2)
C1	C7	1.5443(18)	C10	C11	1.519(2)
C1	C14	1.5170(17)	C11	C12	1.526(2)
C2	O1	1.2049(17)	C12	C13	1.527(2)
C2	O2	1.3388(15)	C14	C15	1.3958(19)
C3	C4	1.515(2)	C14	C19	1.392(2)
C3	C5	1.520(2)	C15	C16	1.3873(19)
C3	C6	1.514(2)	C16	C17	1.393(2)
C3	O2	1.4807(16)	C17	C18	1.393(2)
C7	C8	1.5308(17)	C17	C20	1.4447(18)
C8	C9	1.5306(18)	C18	C19	1.3884(19)
C8	C13	1.529(2)	C20	N1	1.1474(19)

Supplementary Table 14. Bond Angles for xia2408.

Atom	Atom	Atom	Angle/°	Atom	Atom	Atom	Angle/°
C2	C1	C7	108.52(11)	C11	C10	C9	111.31(13)
C14	C1	C2	109.44(10)	C10	C11	C12	111.02(13)
C14	C1	C7	113.36(10)	C11	C12	C13	111.40(12)
O1	C2	C1	124.08(12)	C12	C13	C8	111.83(13)
O1	C2	O2	125.13(13)	C15	C14	C1	121.88(12)
O2	C2	C1	110.78(11)	C19	C14	C1	119.31(12)
C4	C3	C5	110.24(12)	C19	C14	C15	118.78(12)
C6	C3	C4	112.55(13)	C16	C15	C14	120.68(13)
C6	C3	C5	111.26(13)	C15	C16	C17	119.70(13)
O2	C3	C4	109.86(11)	C16	C17	C18	120.38(12)
O2	C3	C5	101.87(11)	C16	C17	C20	119.58(13)
O2	C3	C6	110.54(11)	C18	C17	C20	120.03(13)
C8	C7	C1	114.18(10)	C19	C18	C17	119.16(13)
C9	C8	C7	110.40(11)	C18	C19	C14	121.25(13)
C13	C8	C7	112.57(12)	N1	C20	C17	179.25(18)
C13	C8	C9	109.54(11)	C2	O2	C3	121.09(11)
C10	C9	C8	111.86(12)				

Supplementary Table 15. Torsion Angles for xia2408.

A	B	C	D	Angle/°	A	B	C	D	Angle/°
C1	C2	O2	C3	175.33(10)	C9	C8	C13	C12	55.76(16)
C1	C7	C8	C9	-175.89(12)	C9	C10	C11	C12	-54.92(18)
C1	C7	C8	C13	61.35(16)	C10	C11	C12	C13	54.78(19)
C1	C14	C15	C16	-176.64(12)	C11	C12	C13	C8	-55.88(18)
C1	C14	C19	C18	176.25(12)	C13	C8	C9	C10	-55.96(16)
C2	C1	C7	C8	60.07(15)	C14	C1	C2	O1	-60.57(17)
C2	C1	C14	C15	70.17(15)	C14	C1	C2	O2	120.56(11)
C2	C1	C14	C19	-108.01(13)	C14	C1	C7	C8	-178.13(12)
C4	C3	O2	C2	-61.00(16)	C14	C15	C16	C17	0.3(2)
C5	C3	O2	C2	-177.87(12)	C15	C14	C19	C18	-1.99(19)
C6	C3	O2	C2	63.80(16)	C15	C16	C17	C18	-1.8(2)
C7	C1	C2	O1	63.60(17)	C15	C16	C17	C20	177.23(13)
C7	C1	C2	O2	-115.27(11)	C16	C17	C18	C19	1.3(2)
C7	C1	C14	C15	-51.11(17)	C17	C18	C19	C14	0.6(2)
C7	C1	C14	C19	130.70(13)	C19	C14	C15	C16	1.55(19)
C7	C8	C9	C10	179.52(12)	C20	C17	C18	C19	-177.65(12)
C7	C8	C13	C12	179.00(11)	O1	C2	O2	C3	-3.5(2)
C8	C9	C10	C11	56.32(17)					

Supplementary Table 16. Hydrogen Atom Coordinates (Å×10^4) and Isotropic Displacement Parameters (Å^2×10^3) for xia2408.

**Atom**	***x***	***y***	***z***	**U(eq)**
H1	8334.37	6626.35	3828.43	34
H4A	3800.6	5221.75	2877.85	62
H4B	2215.83	6138.77	2581.34	62
H4C	1551.9	6095.74	3039.03	62
H5A	7543.33	8179.5	2666.73	67
H5B	5707.14	7554.24	2349.8	67
H5C	7349.43	6522.64	2605.42	67
H6A	1886.36	8626.4	3150.38	67
H6B	2347.33	8889.72	2691.83	67
H6C	4228.64	9461.17	3009.71	67
H7A	7373.49	4906.46	4316.86	39
H7B	4577.1	5090.45	4221.44	39
H8	5214.34	4198.51	3577.33	36
H9A	6793.67	2327.37	4185.7	43
H9B	4041.13	2600.32	4070.63	43
H10A	5455.08	476.34	3785.39	50
H10B	4621.67	1553.79	3451.27	50
H11A	8316.6	589.45	3263.27	54
H11B	9505.36	1019.34	3675.06	54
H12A	7981.39	2945.62	3076.99	53
H12B	10716.77	2657.04	3200.42	53
H13A	9292.67	4786.53	3482.71	44
H13B	10091.13	3703.64	3817.44	44
H15	3372.03	7166.59	4509.91	39
H16	3200.07	8968.89	4972.57	41
H18	9649.36	10426.16	4586.84	39
H19	9816.19	8600.33	4131.1	37

Supplementary Figures

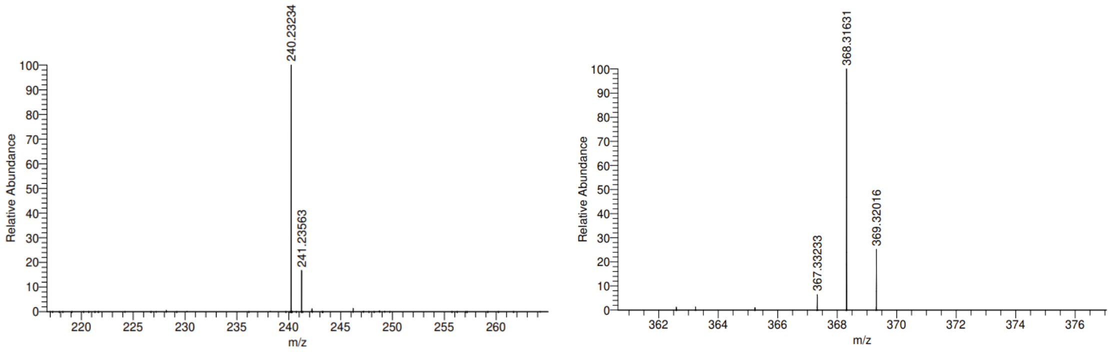

Supplementary Figure 1. HR-MS for radical capture experiment.

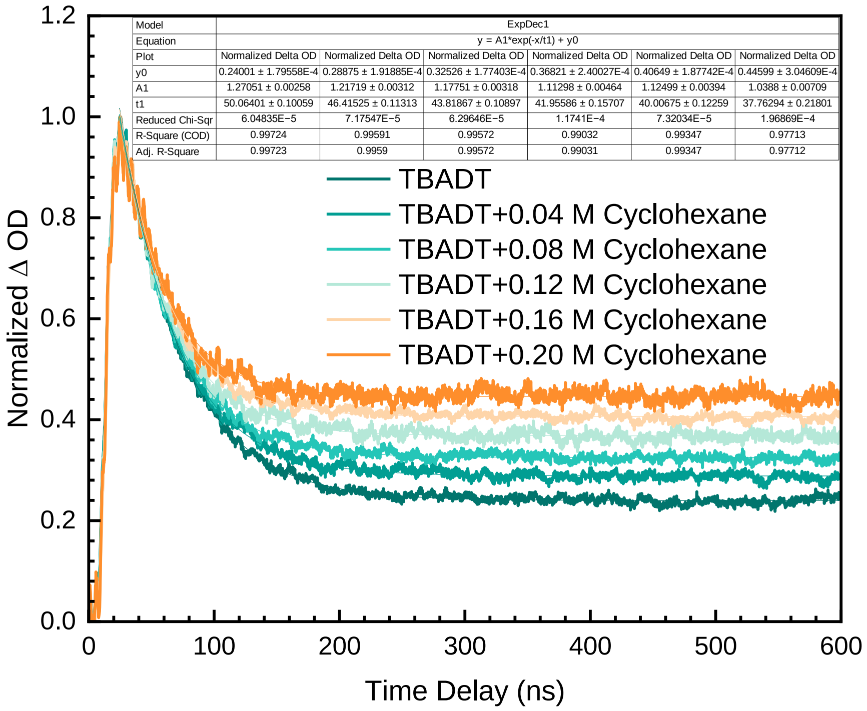

Model	ExpDec1					
Equation	y = A1*exp(-x/t1) + y0					
Plot	Normalized Delta OD	Normalized Delta OD	Normalized Delta OD	Normalized Delta OD	Normalized Delta OD	Normalized Delta OD
y0	0.24001 ± 1.79558E-4	0.28875 ± 1.91885E-4	0.32526 ± 1.77403E-4	0.36821 ± 2.40027E-4	0.40649 ± 1.87742E-4	0.44599 ± 3.04609E-4
A1	1.27051 ± 0.00258	1.21719 ± 0.00312	1.17751 ± 0.00318	1.11298 ± 0.00464	1.12499 ± 0.00394	1.0388 ± 0.00709
t1	50.06401 ± 0.10059	46.41525 ± 0.11313	43.81867 ± 0.10897	41.95586 ± 0.15707	40.00675 ± 0.12259	37.76294 ± 0.21801
Reduced Chi-Sqr	6.04835E−5	7.17547E−5	6.29646E−5	1.1741E−4	7.32034E−5	1.96869E−4
R-Square (COD)	0.99724	0.99591	0.99572	0.99032	0.99347	0.97713
Adj. R-Square	0.99723	0.9959	0.99572	0.99031	0.99347	0.97712

Supplementary Figure 2. Decay profiles and first order fitting of TBADT (2.0×10^{-4} M in CH_3CN) upon addition of increasing amounts of cyclohexane.

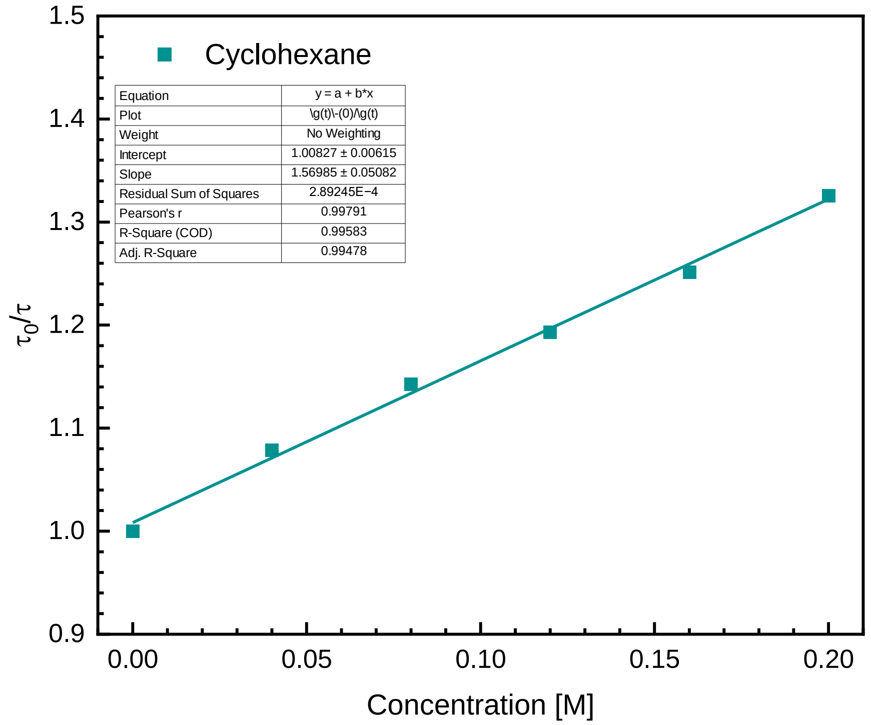

Supplementary Figure 3. Stern-Volmer plots of TBADT (2.0×10^{-4} M in CH_3CN) by cyclohexane.

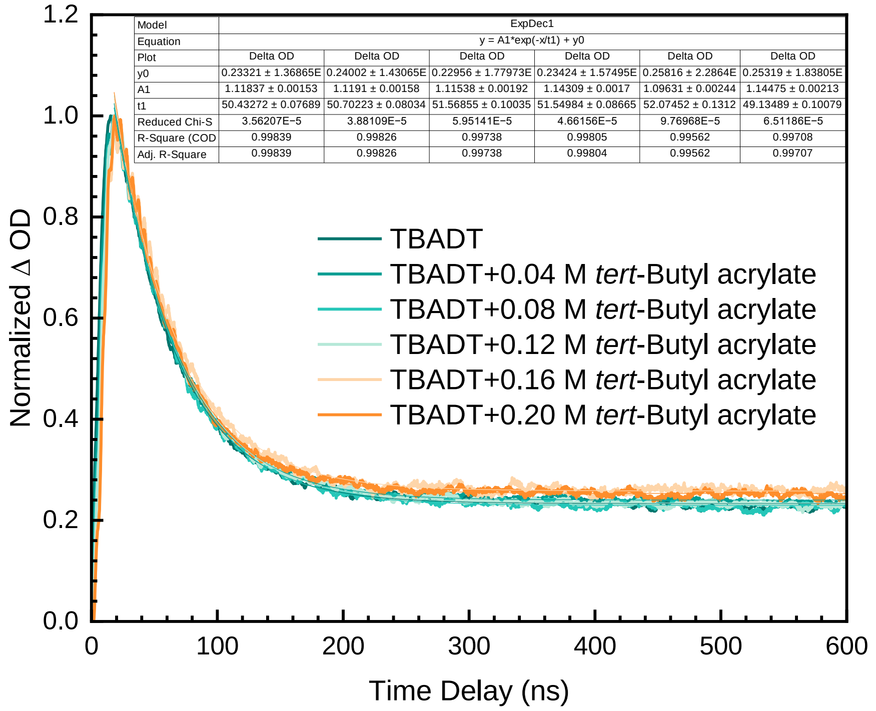

Supplementary Figure 4. Decay profiles and first order fitting of TBADT (2.0×10^{-4} M in CH_3CN) upon addition of increasing amounts of *tert*-butyl acrylate.

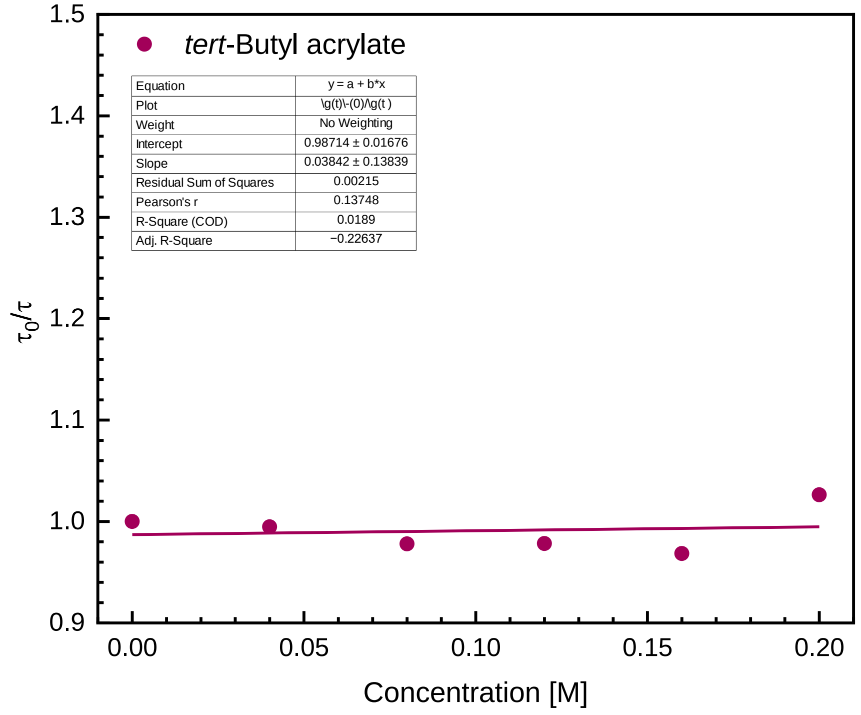

Equation	y = a + b*x
Plot	\g(t)\-(0)/\g(t)
Weight	No Weighting
Intercept	0.98714 ± 0.01676
Slope	0.03842 ± 0.13839
Residual Sum of Squares	0.00215
Pearson's r	0.13748
R-Square (COD)	0.0189
Adj. R-Square	−0.22637

Supplementary Figure 5. Stern-Volmer plots of TBADT (2.0×10^{-4} M in CH_3CN) by *tert*-butyl acrylate.

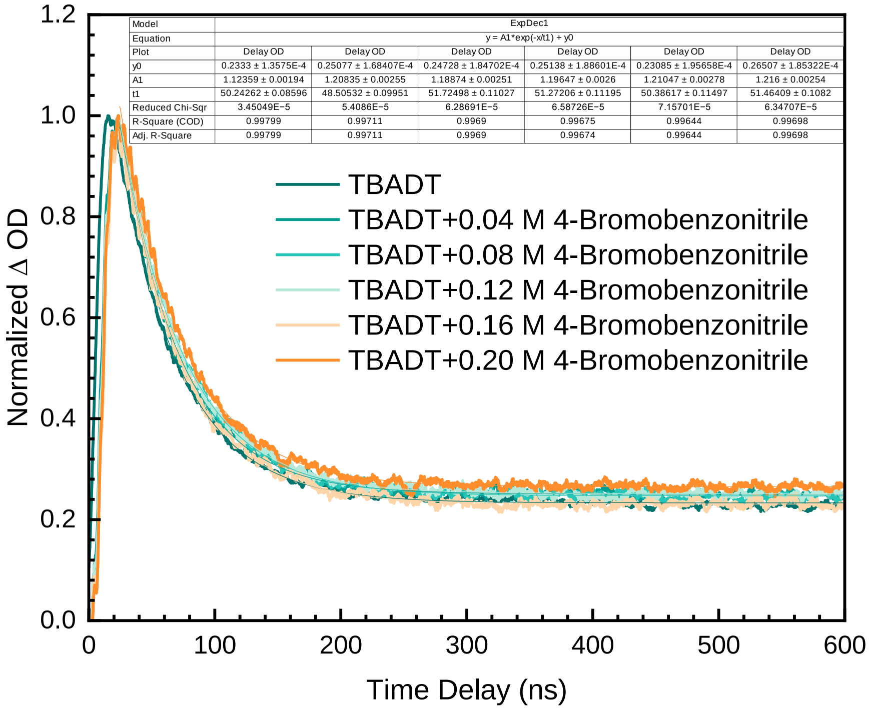

Model	ExpDec1					
Equation	y = A1*exp(-x/t1) + y0					
Plot	Delay OD	Delay OD	Delay OD	Delay OD	Delay OD	Delay OD
y0	0.2333 ± 1.3575E-4	0.25077 ± 1.68407E-4	0.24728 ± 1.84702E-4	0.25138 ± 1.88601E-4	0.23085 ± 1.95658E-4	0.26507 ± 1.85322E-4
A1	1.12359 ± 0.00194	1.20835 ± 0.00255	1.18874 ± 0.00251	1.19647 ± 0.0026	1.21047 ± 0.00278	1.216 ± 0.00254
t1	50.24262 ± 0.08596	48.50532 ± 0.09951	51.72498 ± 0.11027	51.27206 ± 0.11195	50.38617 ± 0.11497	51.46409 ± 0.1082
Reduced Chi-Sqr	3.45049E−5	5.4086E−5	6.28691E−5	6.58726E−5	7.15701E−5	6.34707E−5
R-Square (COD)	0.99799	0.99711	0.9969	0.99675	0.99644	0.99698
Adj. R-Square	0.99799	0.99711	0.9969	0.99674	0.99644	0.99698

Supplementary Figure 6. Decay profiles and first order fitting of TBADT (2.0×10^{-4} M in CH_3CN) upon addition of increasing amounts of 4-bromobenzonitrile.

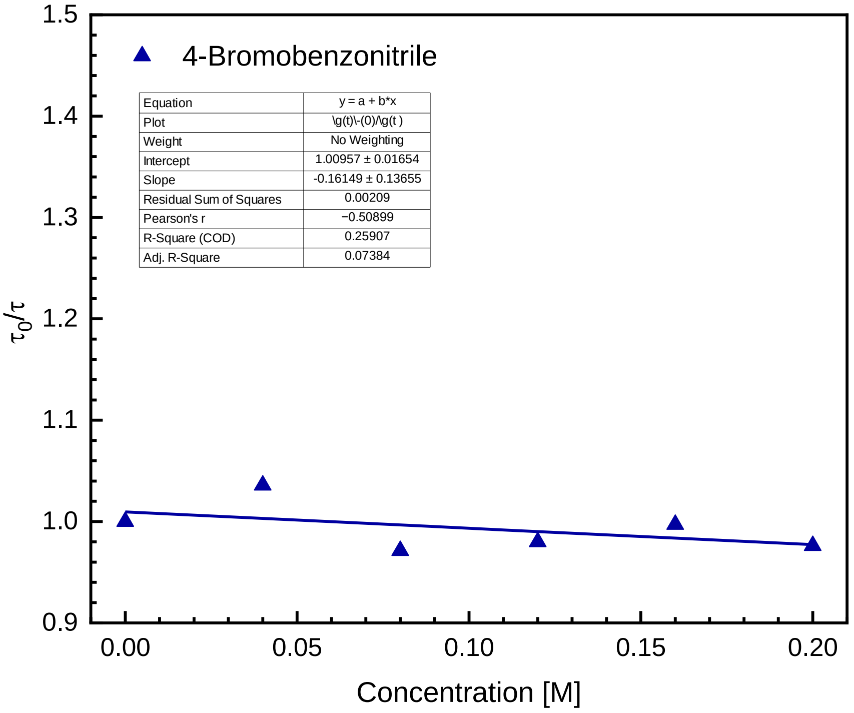

Supplementary Figure 7. Stern-Volmer plots of TBADT (2.0×10^{-4} M in CH_3CN) by 4-bromobenzonitrile.

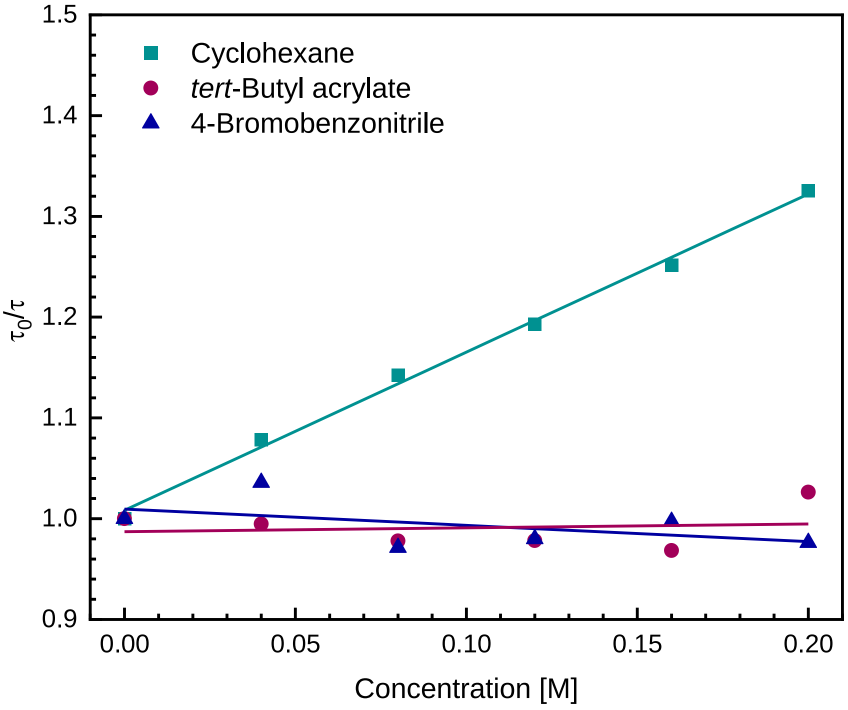

Supplementary Figure 8. Stern-Volmer studies of TBADT (2.0×10^{-4} M in CH_3CN) by cyclohexane, *tert*-butyl acrylate, and 4-bromobenzonitrile.

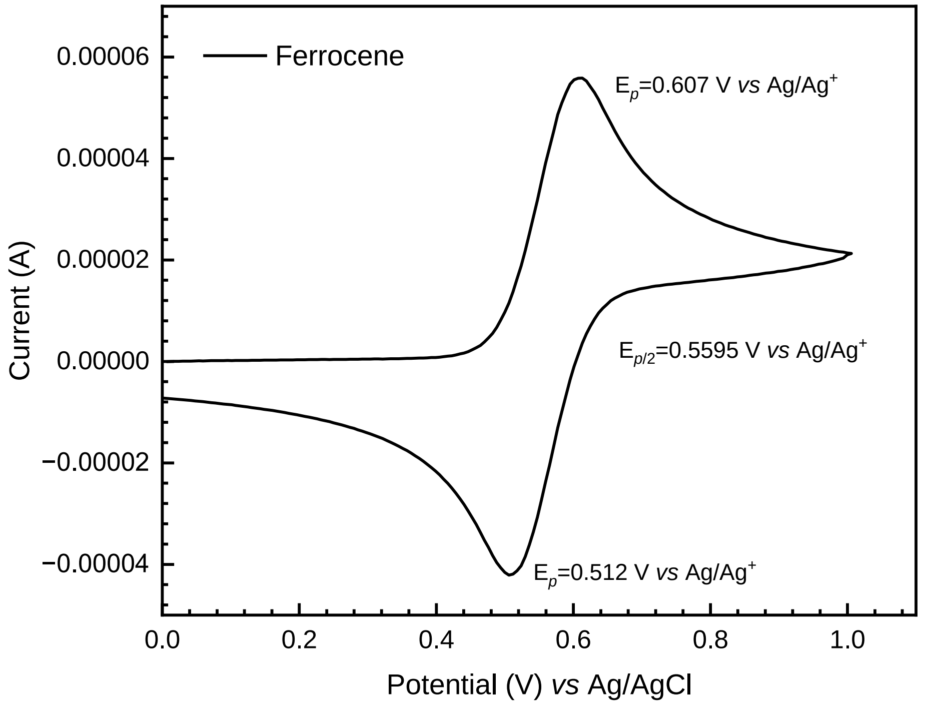

Supplementary Figure 9. Cyclic voltammograms of ferrocene.

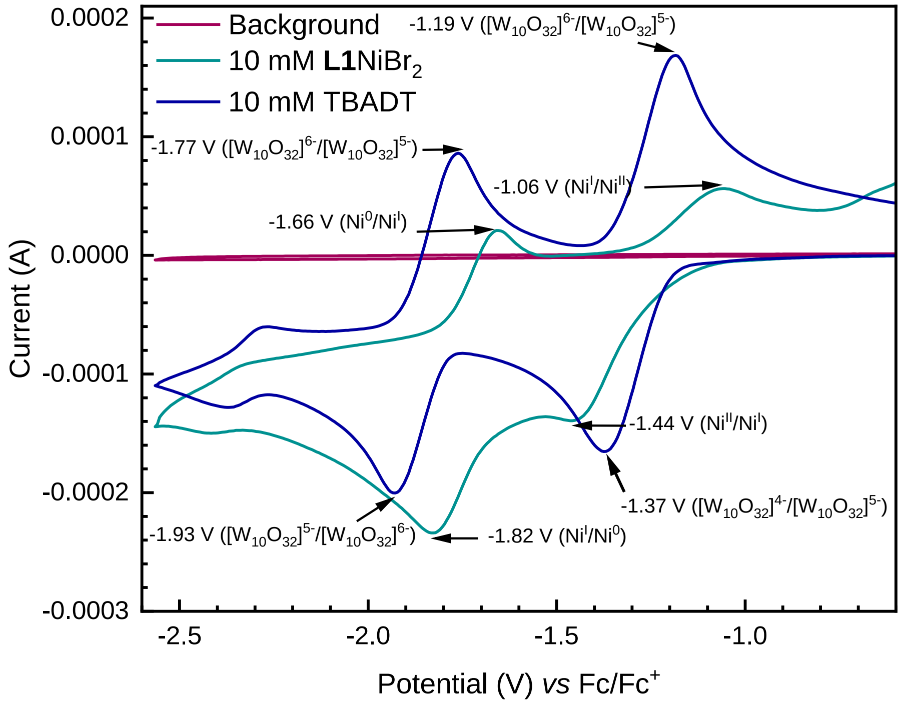

Supplementary Figure 10. Cyclic voltammograms of TBADT and **L1**$NiBr_2$ in CH_3CN at 1 V/s scan rate.

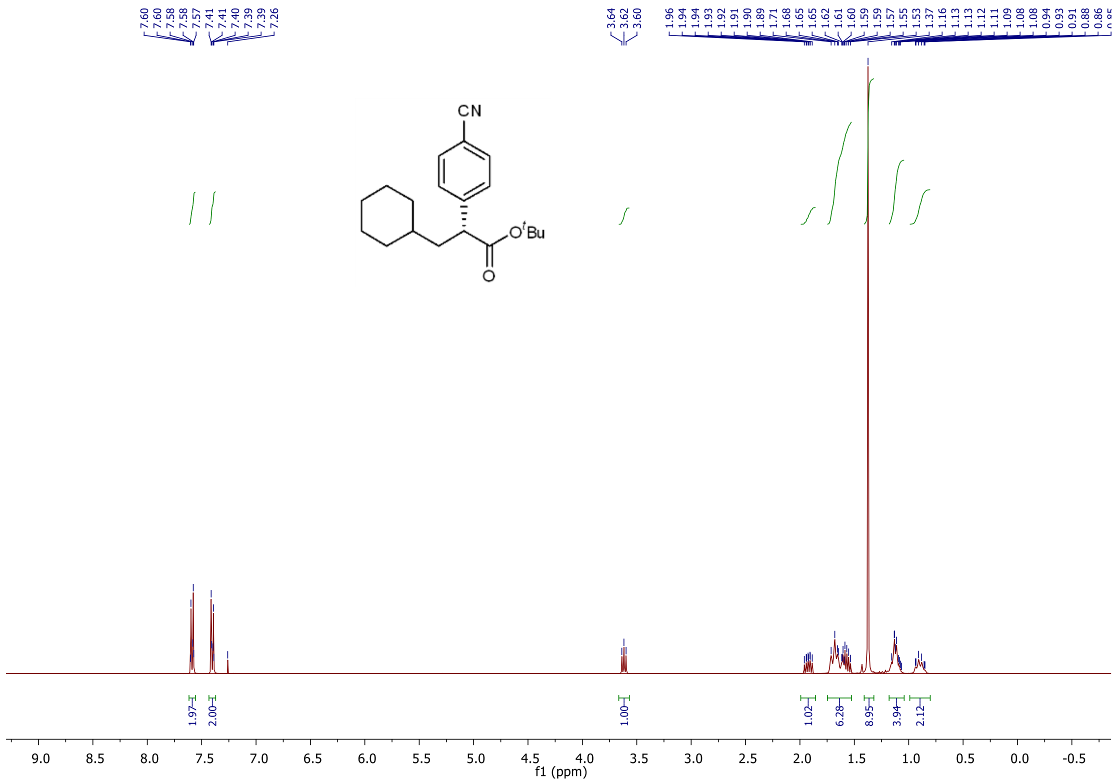

Supplementary Figure 11. ^{1}H NMR of compound **1**.

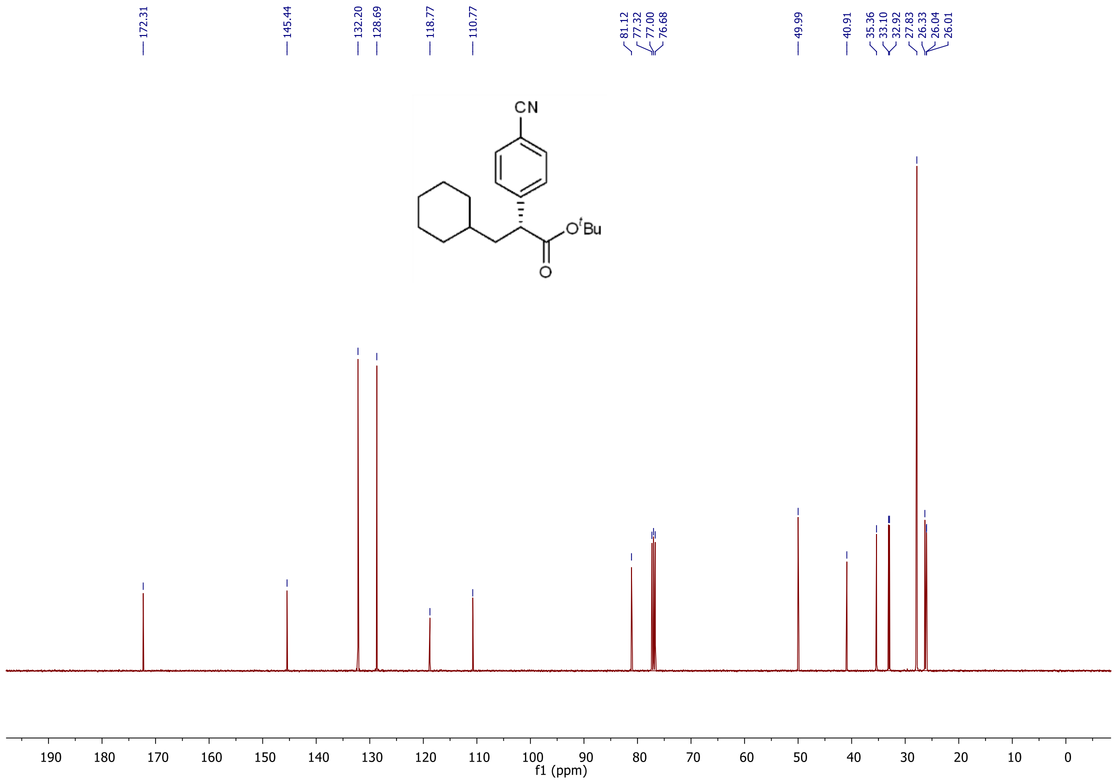

Supplementary Figure 12. ^{13}C NMR of compound **1**.

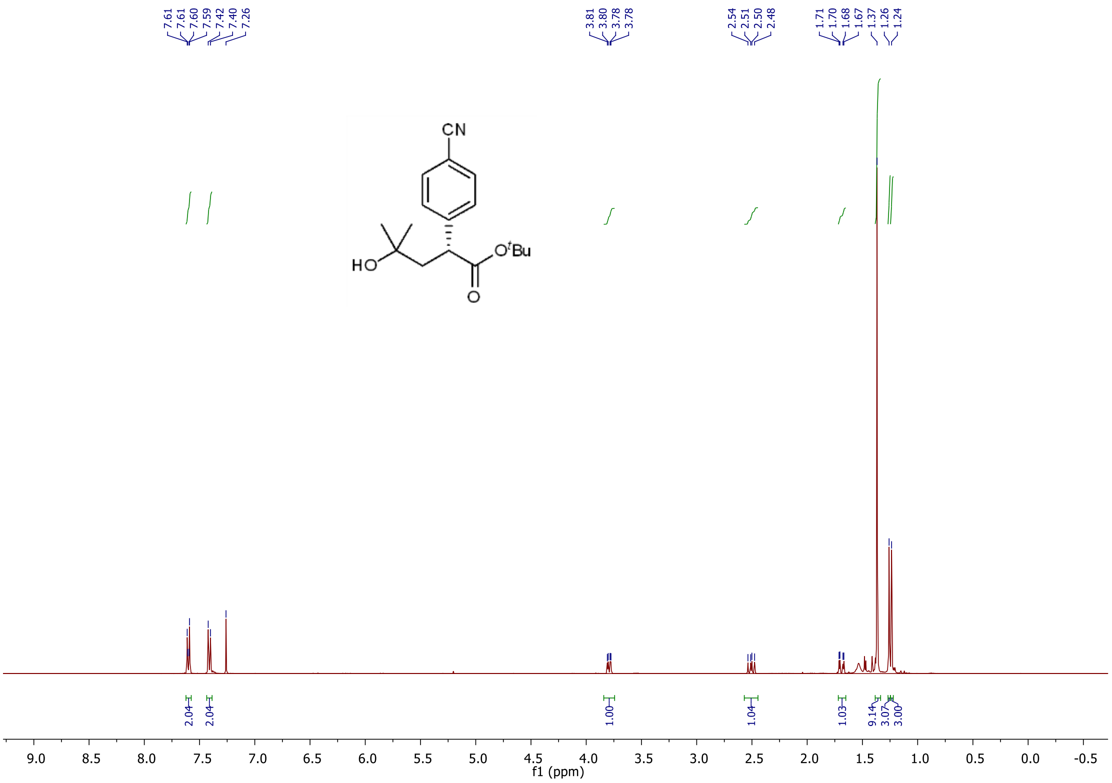

Supplementary Figure 13. ^{1}H NMR of compound **2**.

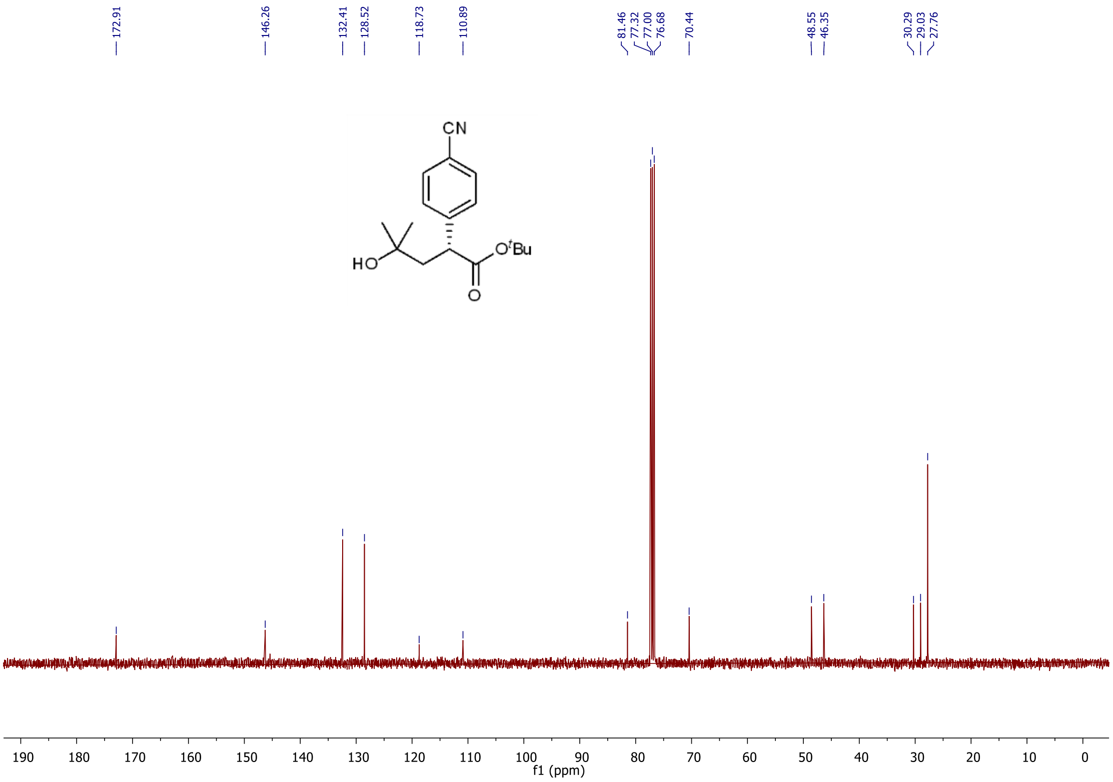

Supplementary Figure 14. ^{13}C NMR of compound **2**.

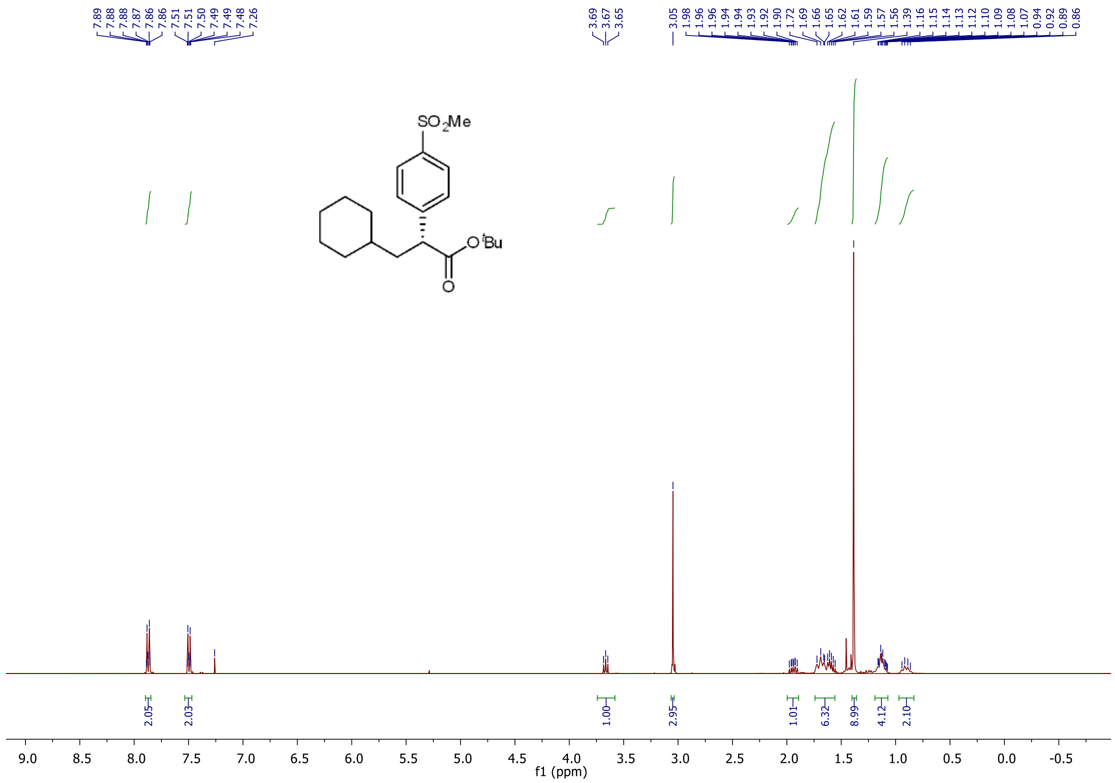

Supplementary Figure 15. ^{1}H NMR of compound **3**.

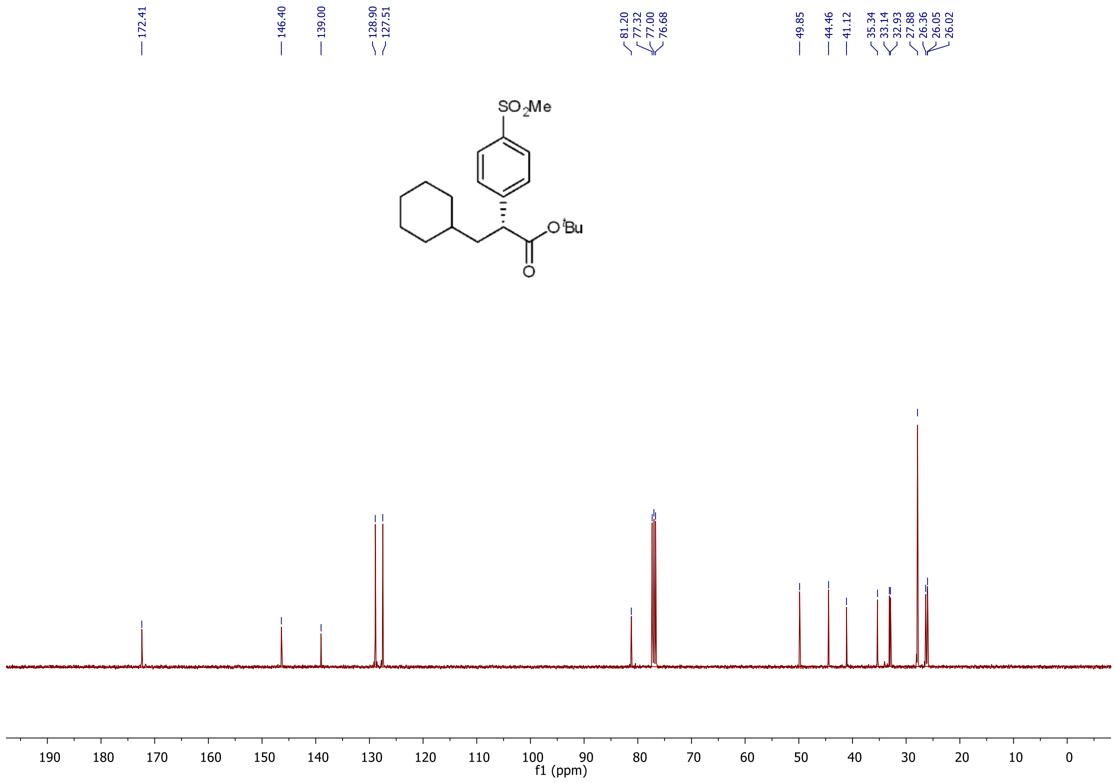

Supplementary Figure 16. ^{13}C NMR of compound **3**.

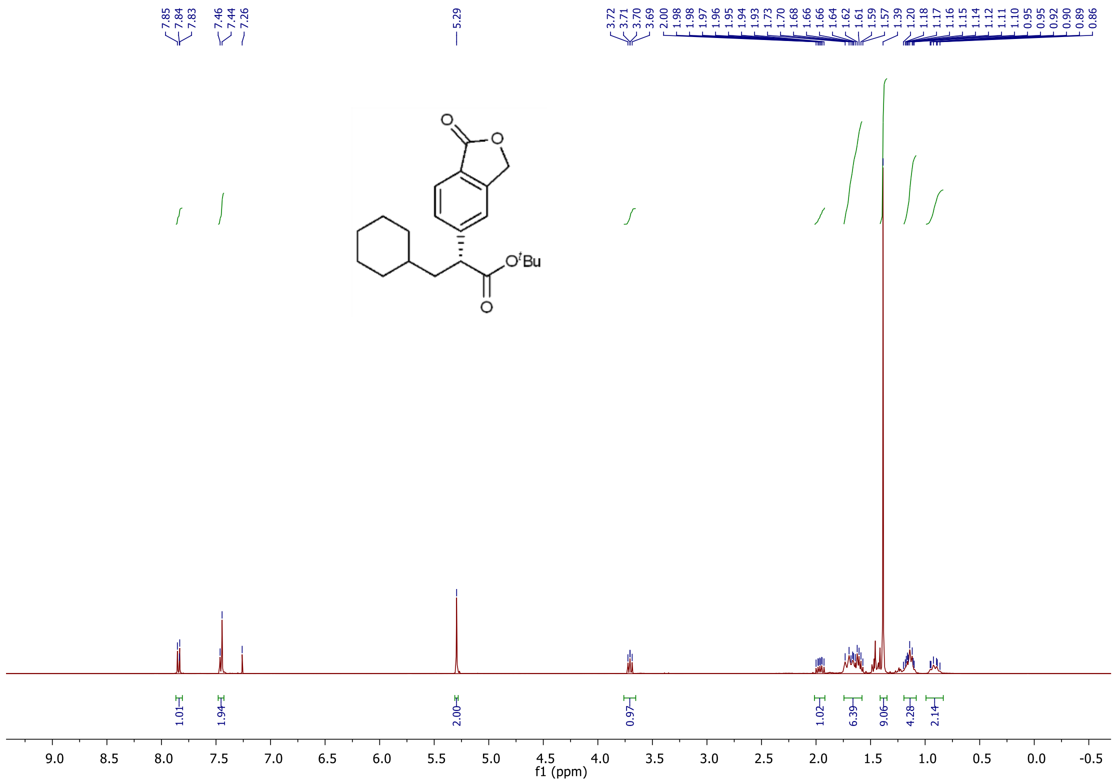

Supplementary Figure 17. ^{1}H NMR of compound **4**.

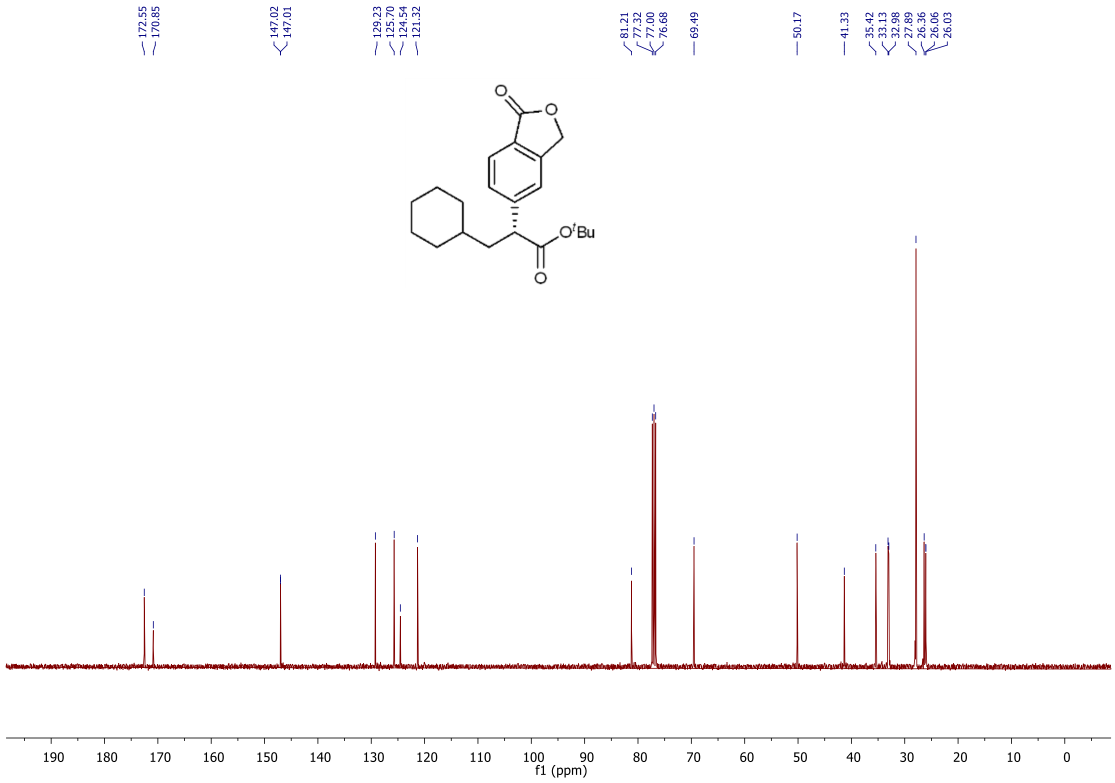

Supplementary Figure 18. ^{13}C NMR of compound **4**.

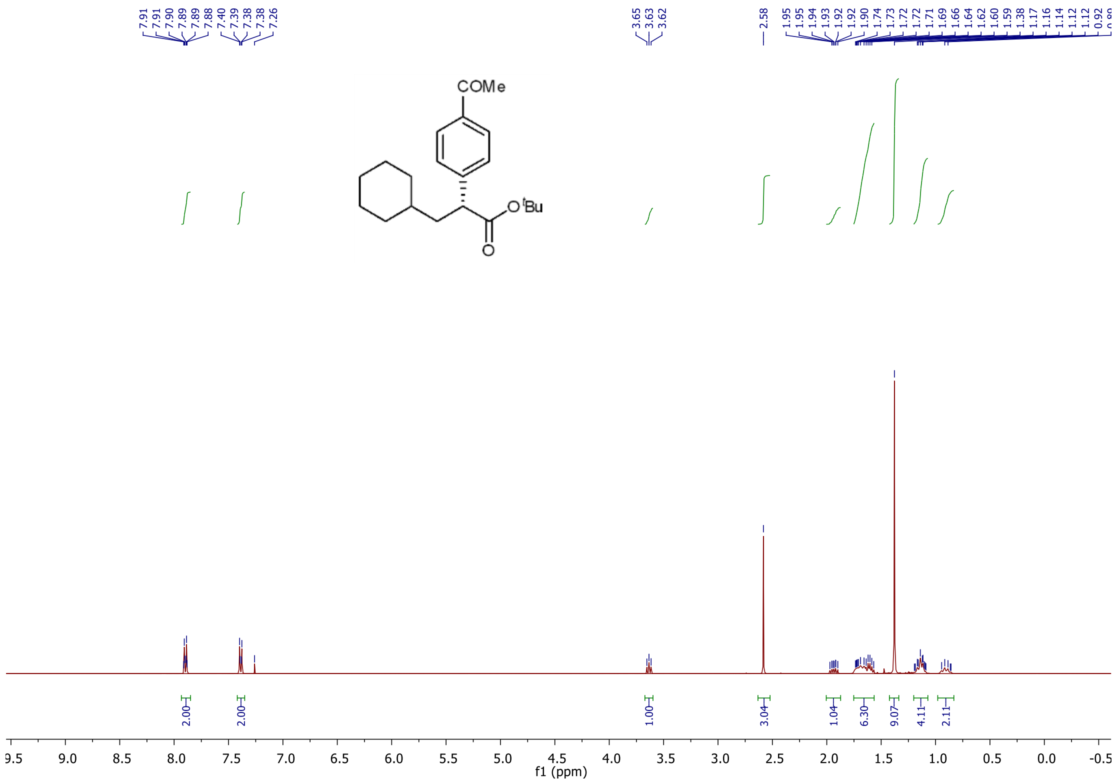

Supplementary Figure 19. ^{1}H NMR of compound **5**.

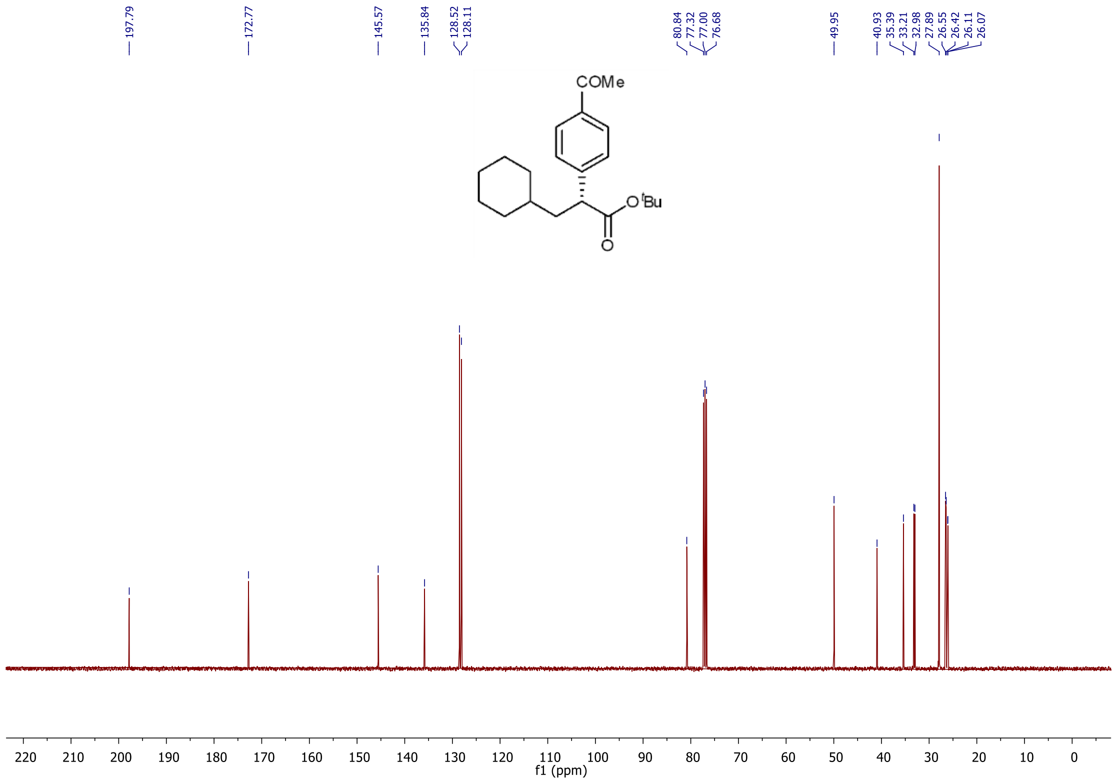

Supplementary Figure 20. ^{13}C NMR of compound **5**.

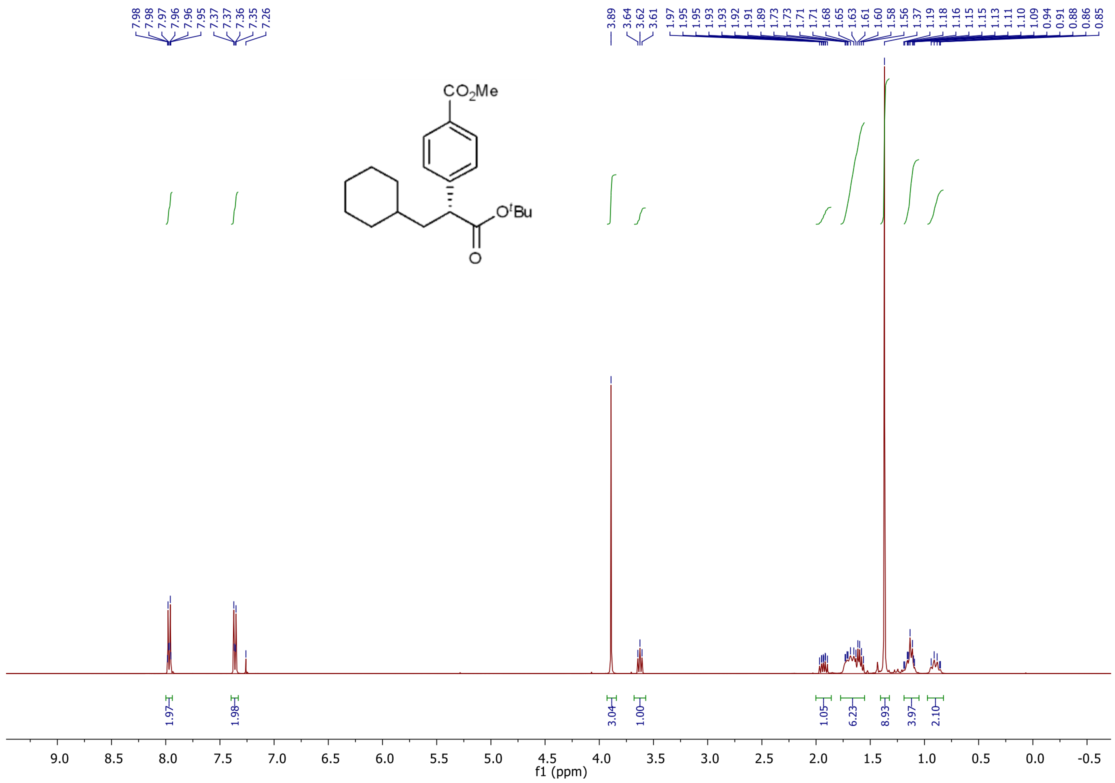

Supplementary Figure 21. ^{1}H NMR of compound **6**.

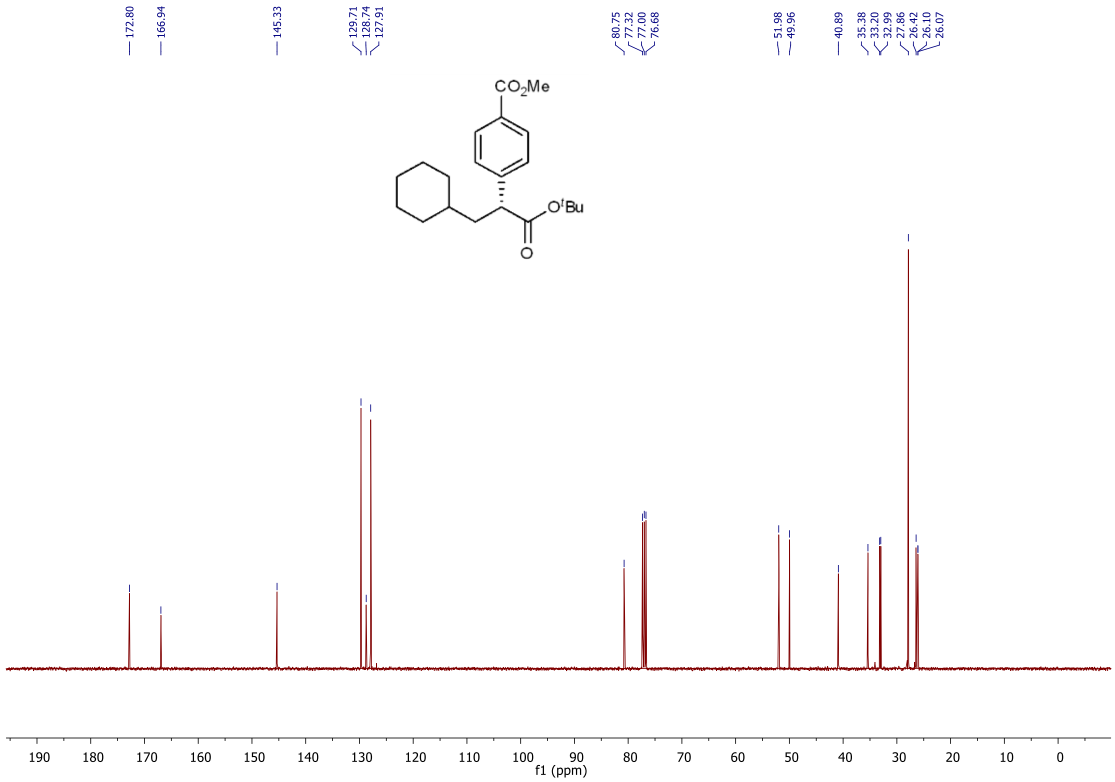

Supplementary Figure 22. ^{13}C NMR of compound **6**.

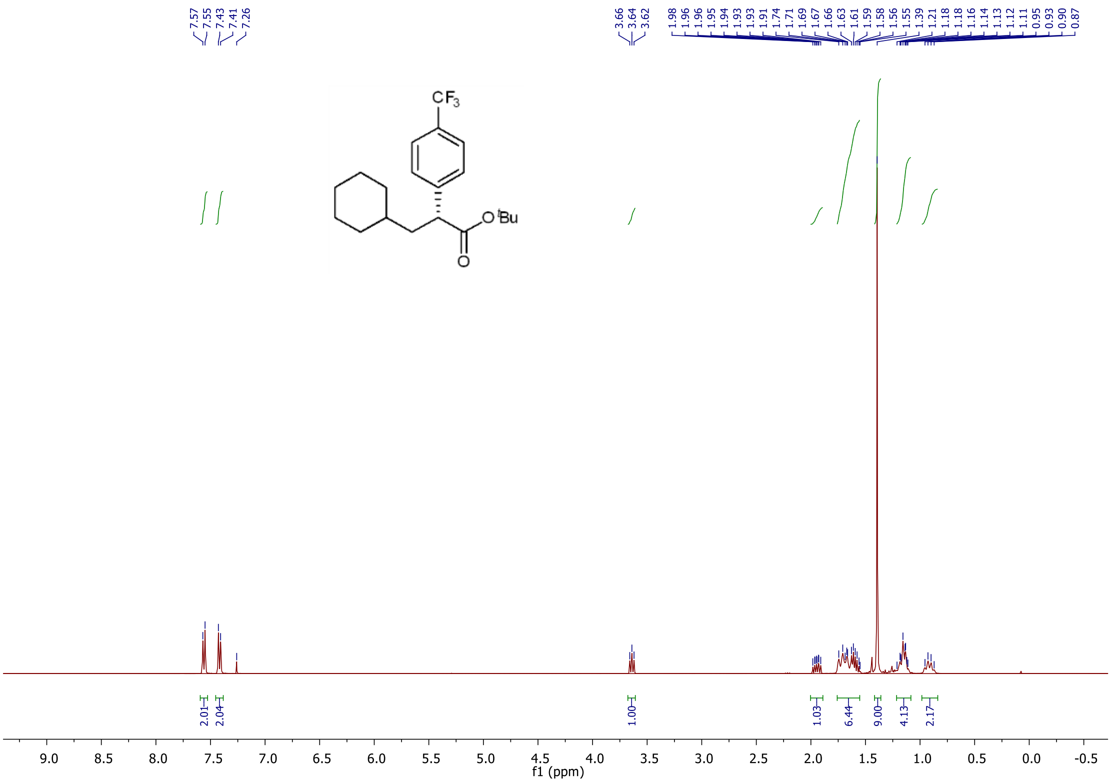

Supplementary Figure 23. ^{1}H NMR of compound **7**.

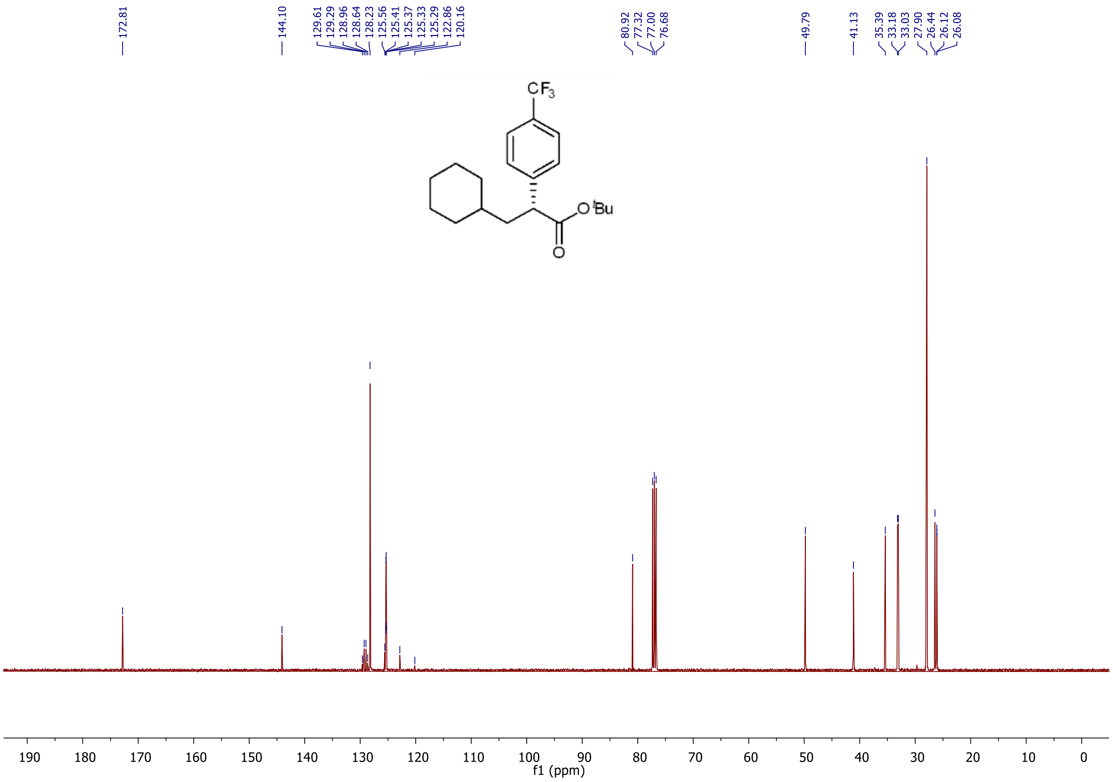

Supplementary Figure 24. ^{13}C NMR of compound **7**.

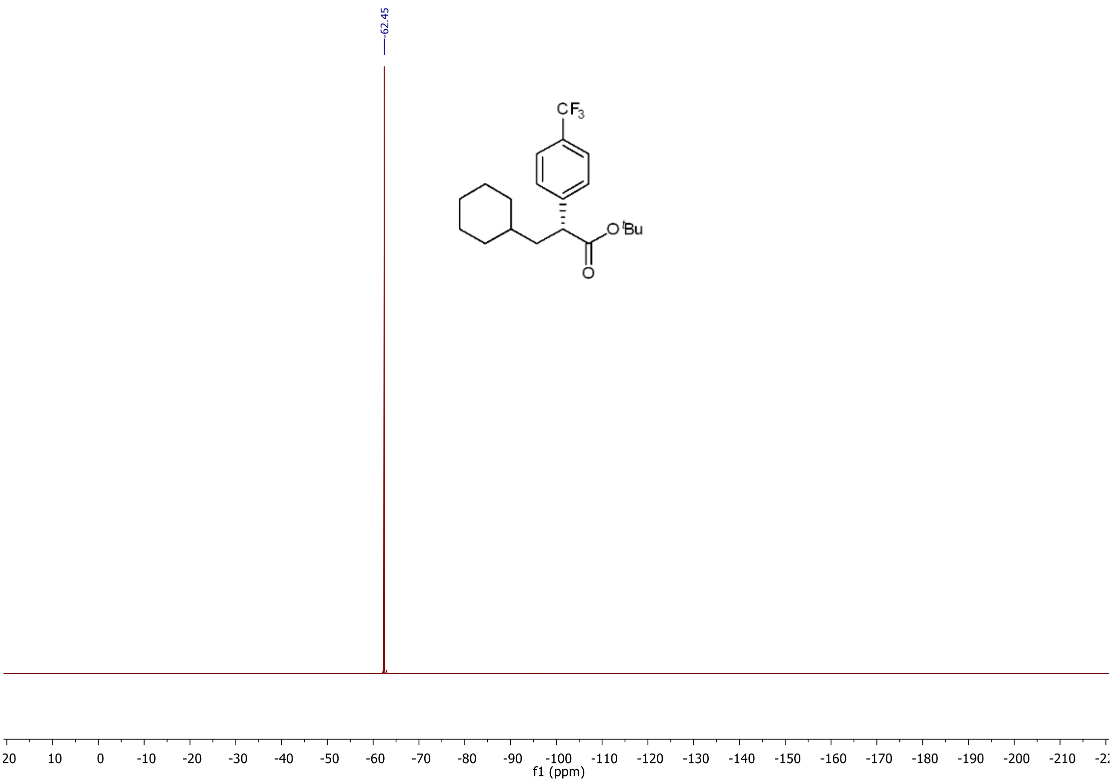

Supplementary Figure 25. ^{19}F NMR of compound **7**.

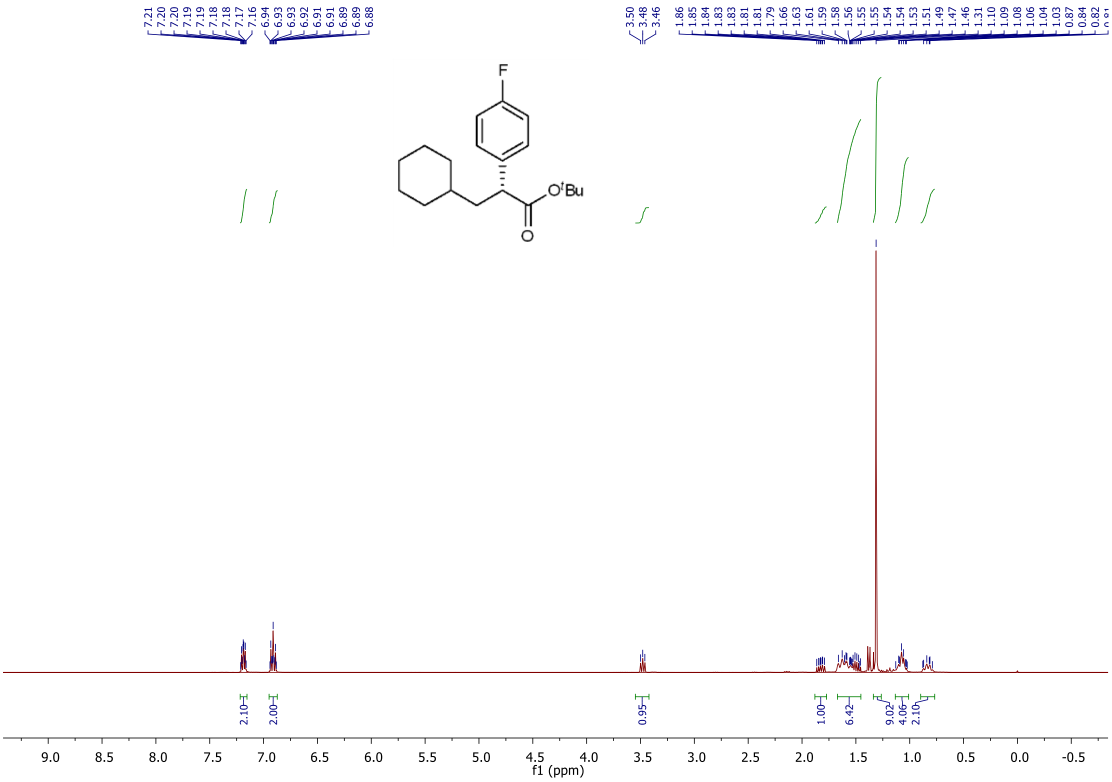

Supplementary Figure 26. ^{1}H NMR of compound **8**.

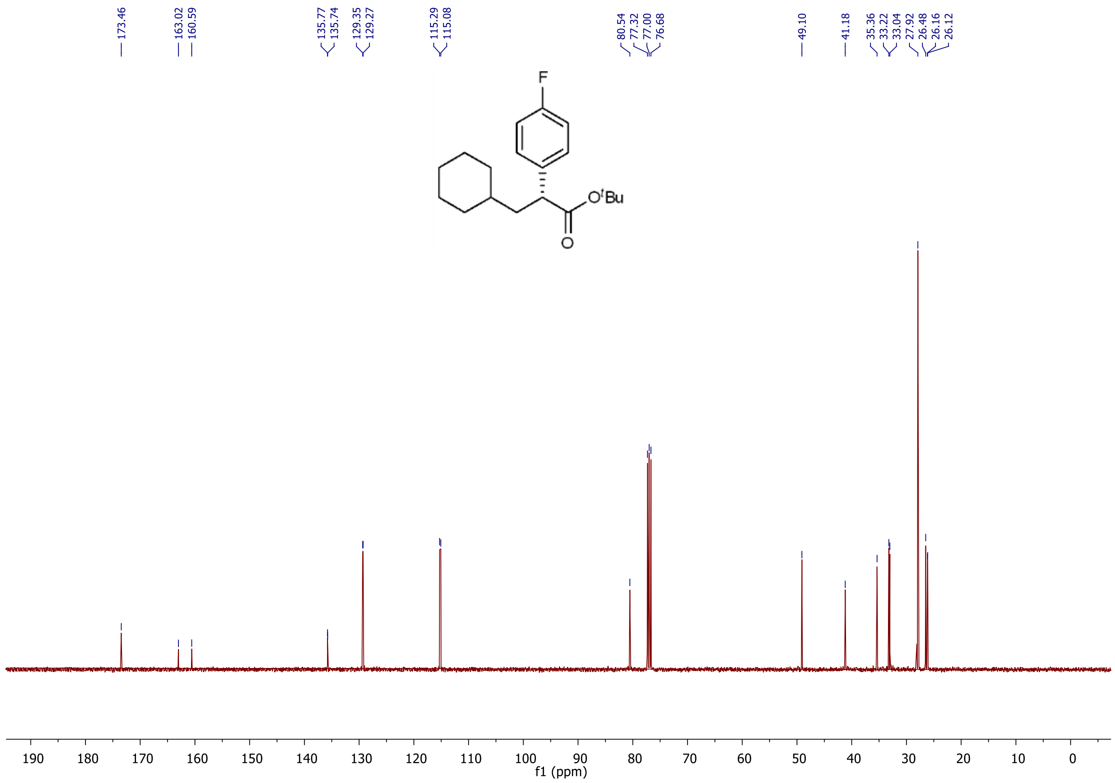

Supplementary Figure 27. ^{13}C NMR of compound **8**.

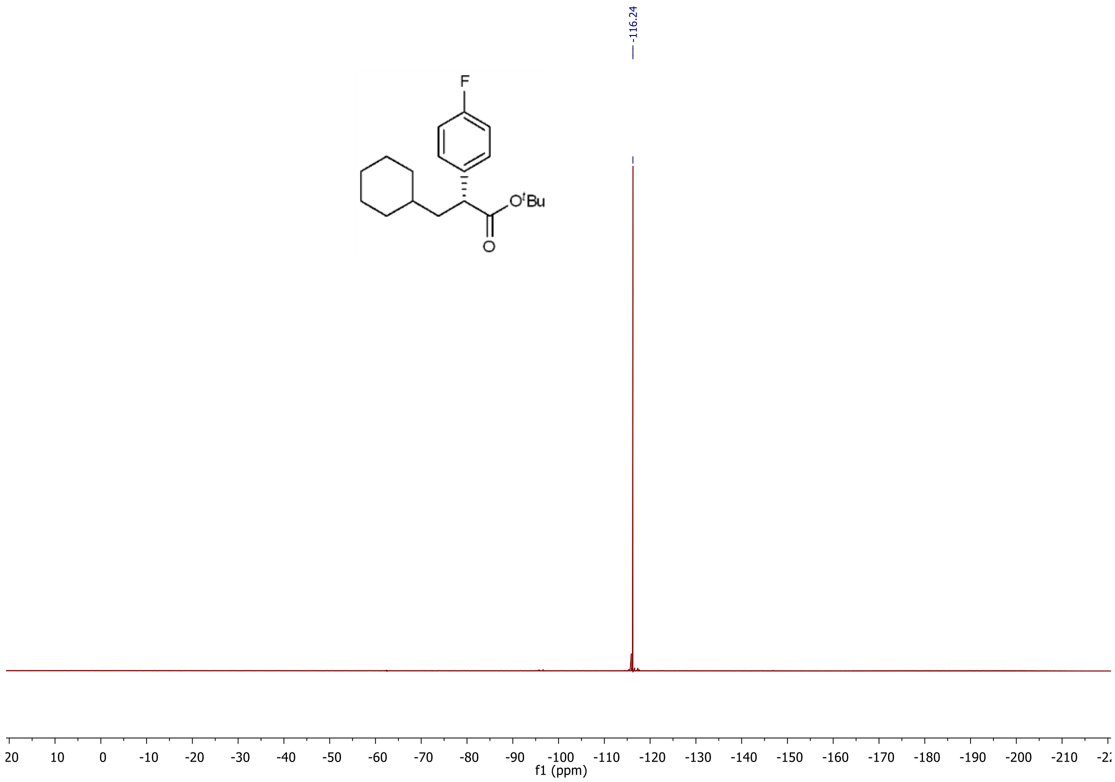

Supplementary Figure 28. ^{19}F NMR of compound **8**.

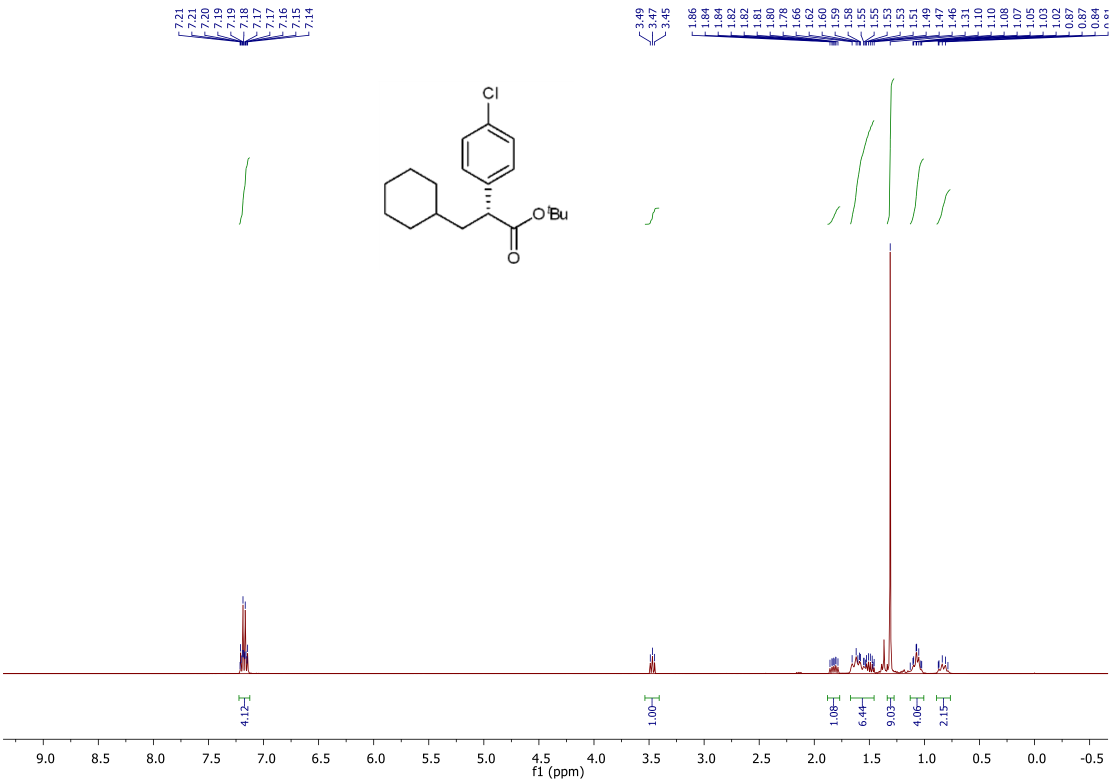

Supplementary Figure 29. ^{1}H NMR of compound **9**.

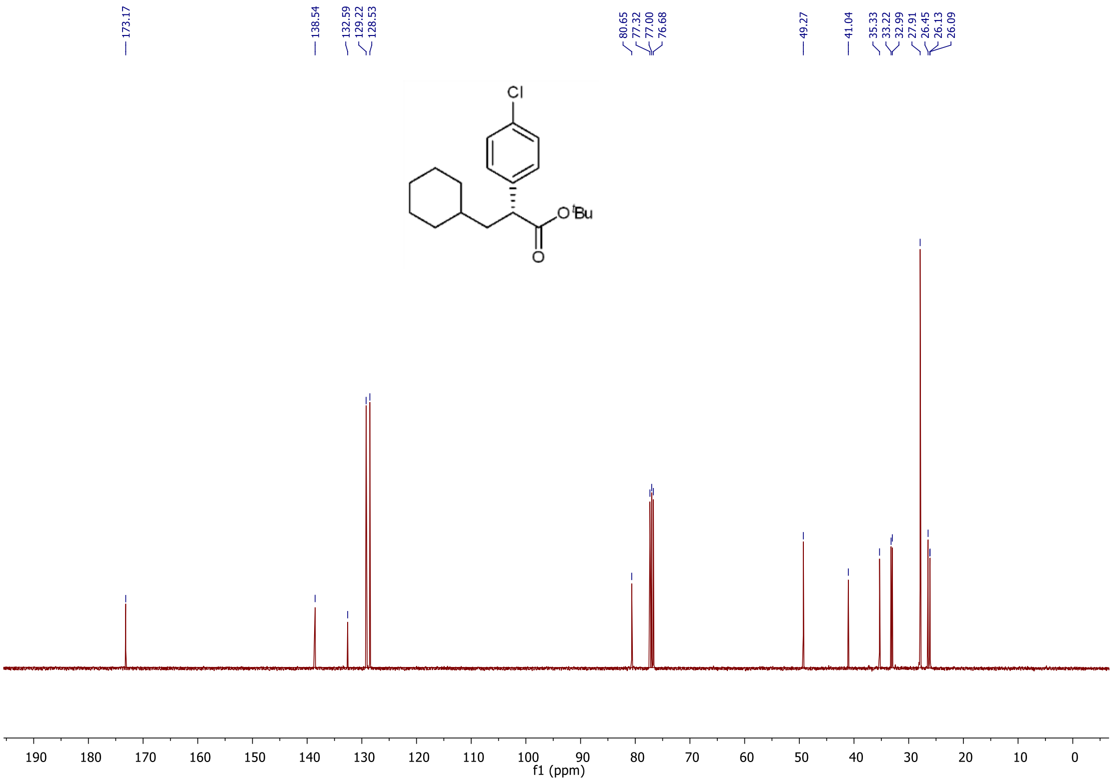

Supplementary Figure 30. ^{13}C NMR of compound **9**.

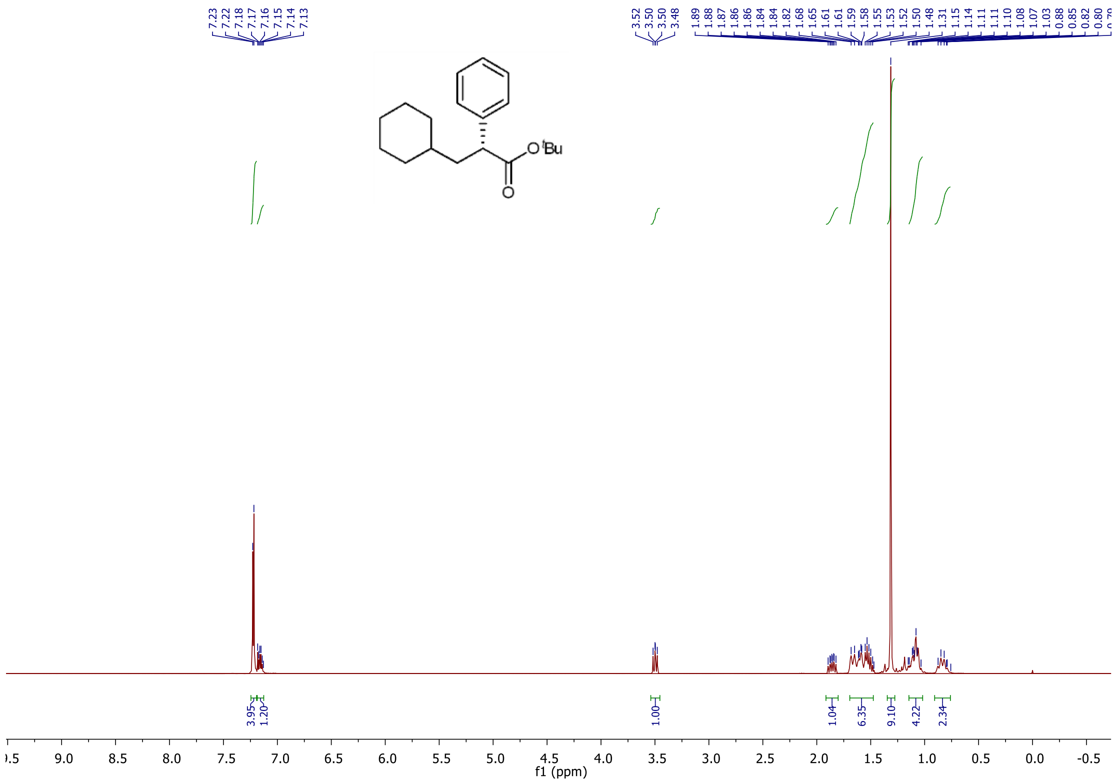

Supplementary Figure 31. ^{1}H NMR of compound **10**.

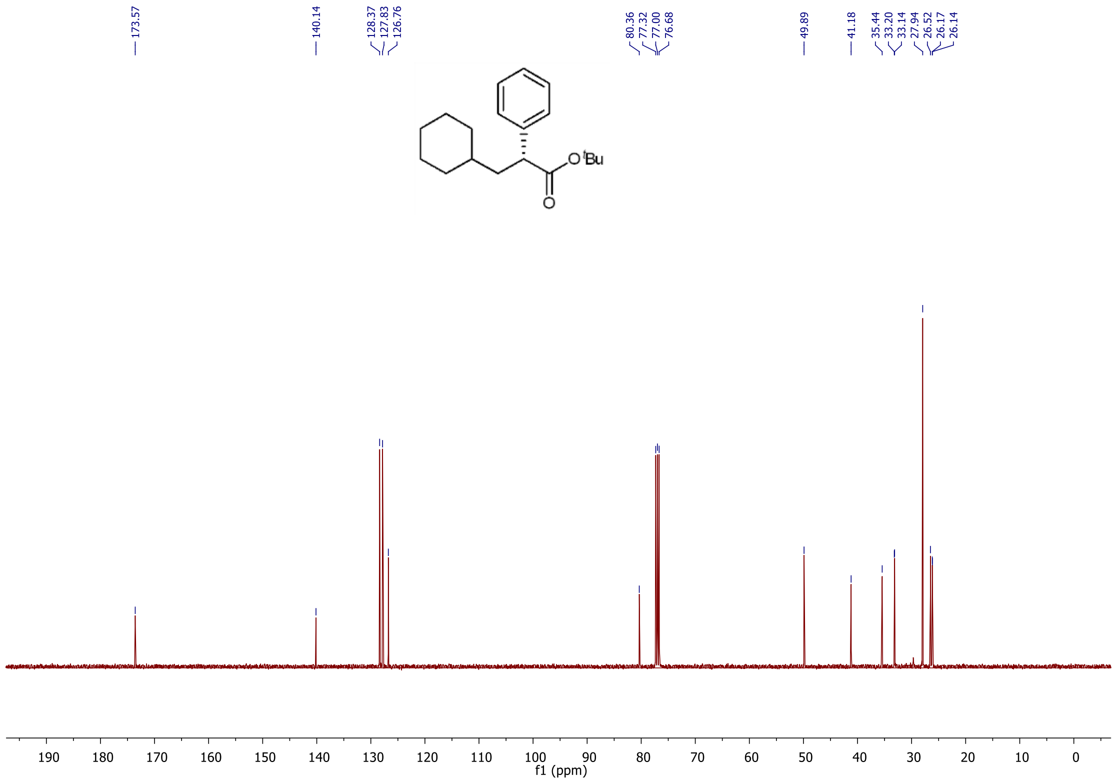

Supplementary Figure 32. ^{13}C NMR of compound **10**.

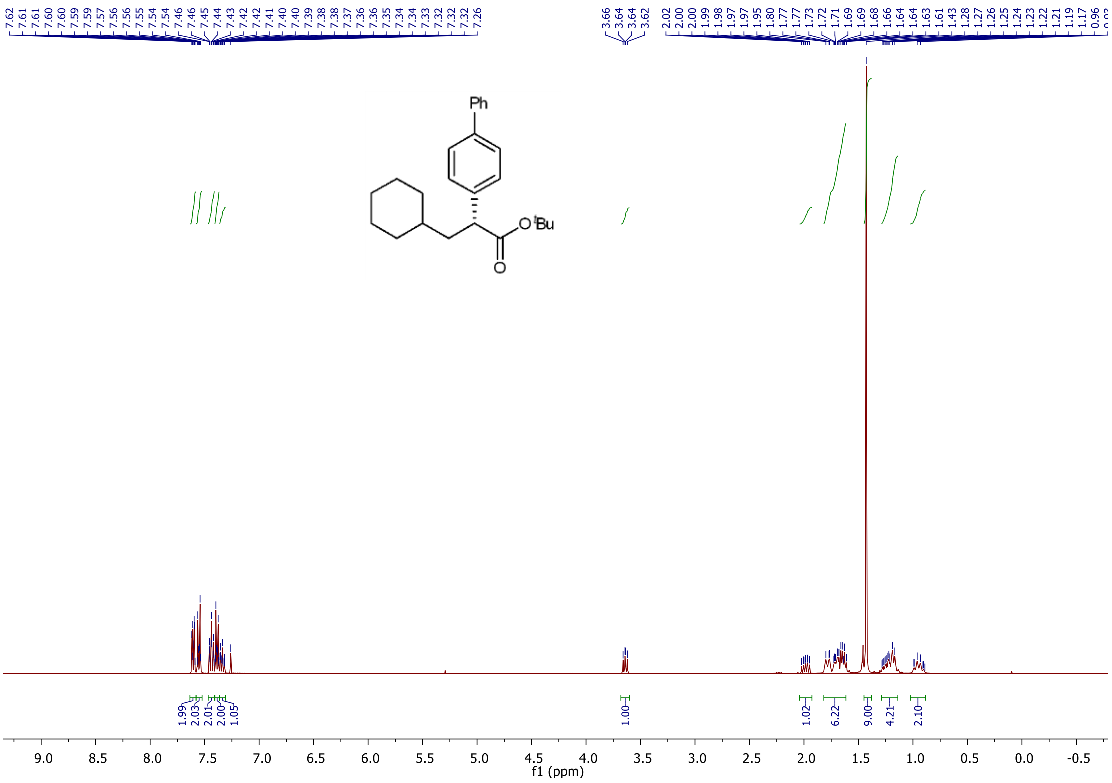

Supplementary Figure 33. ^{1}H NMR of compound **11**.

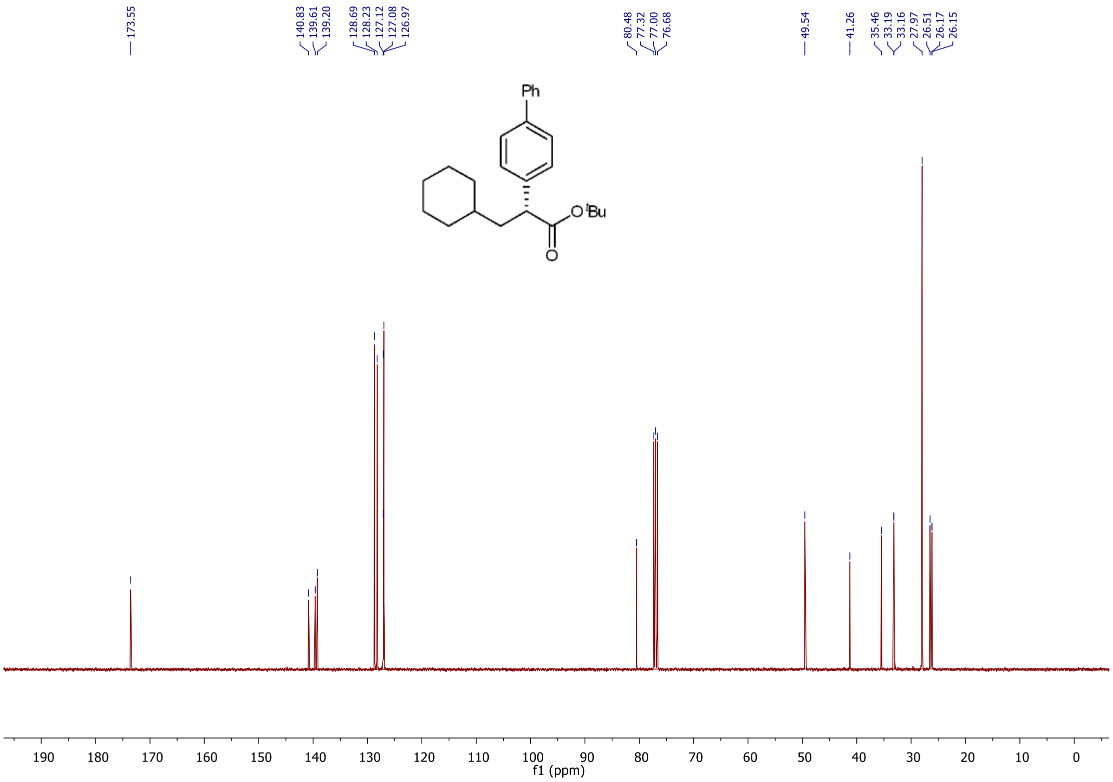

Supplementary Figure 34. ^{13}C NMR of compound **11**.

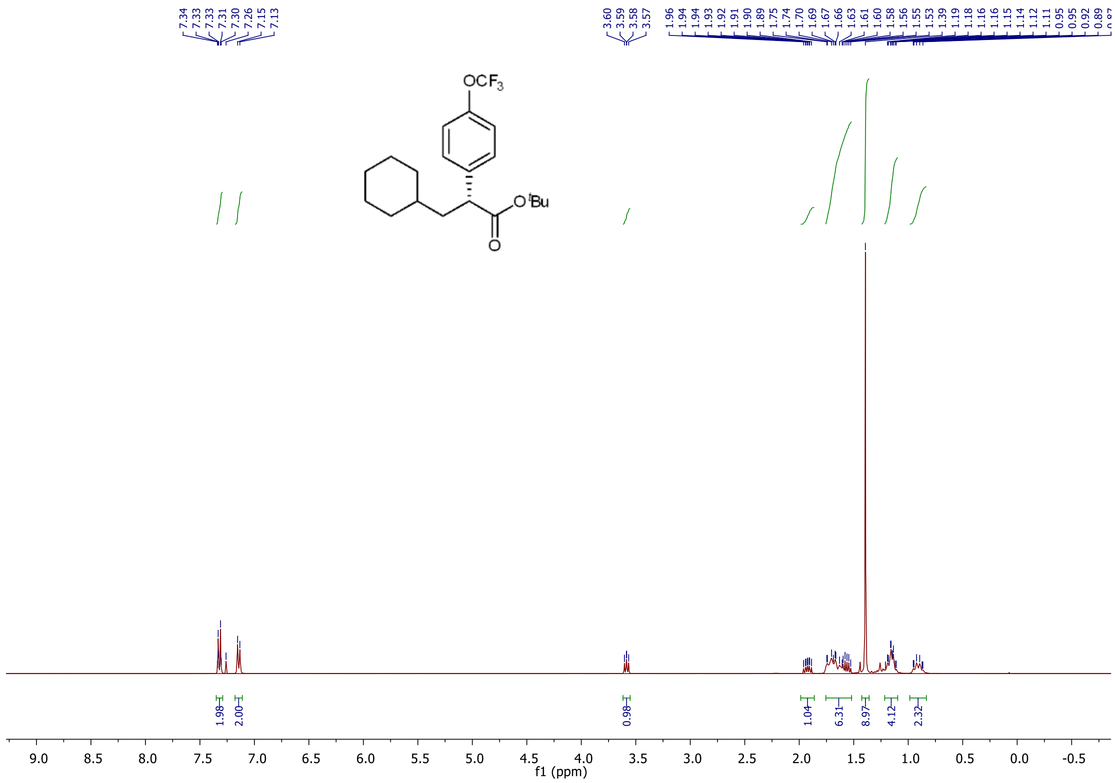

Supplementary Figure 35. ^{1}H NMR of compound **12**.

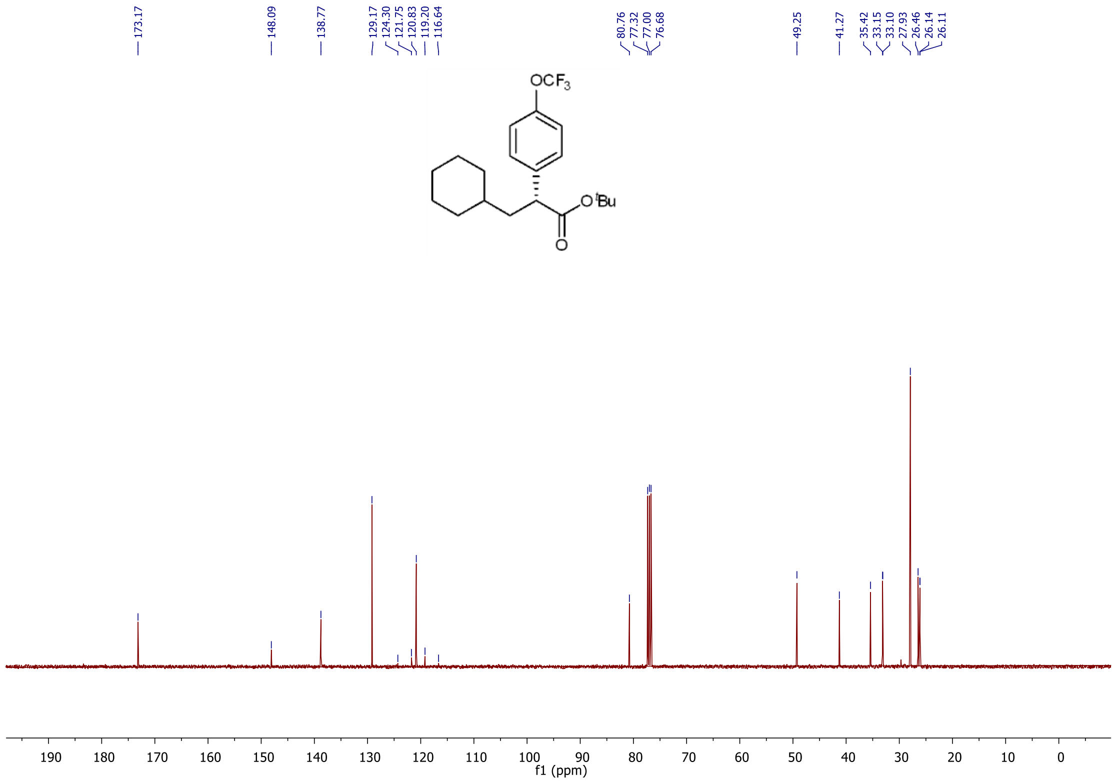

Supplementary Figure 36. ^{13}C NMR of compound **12**.

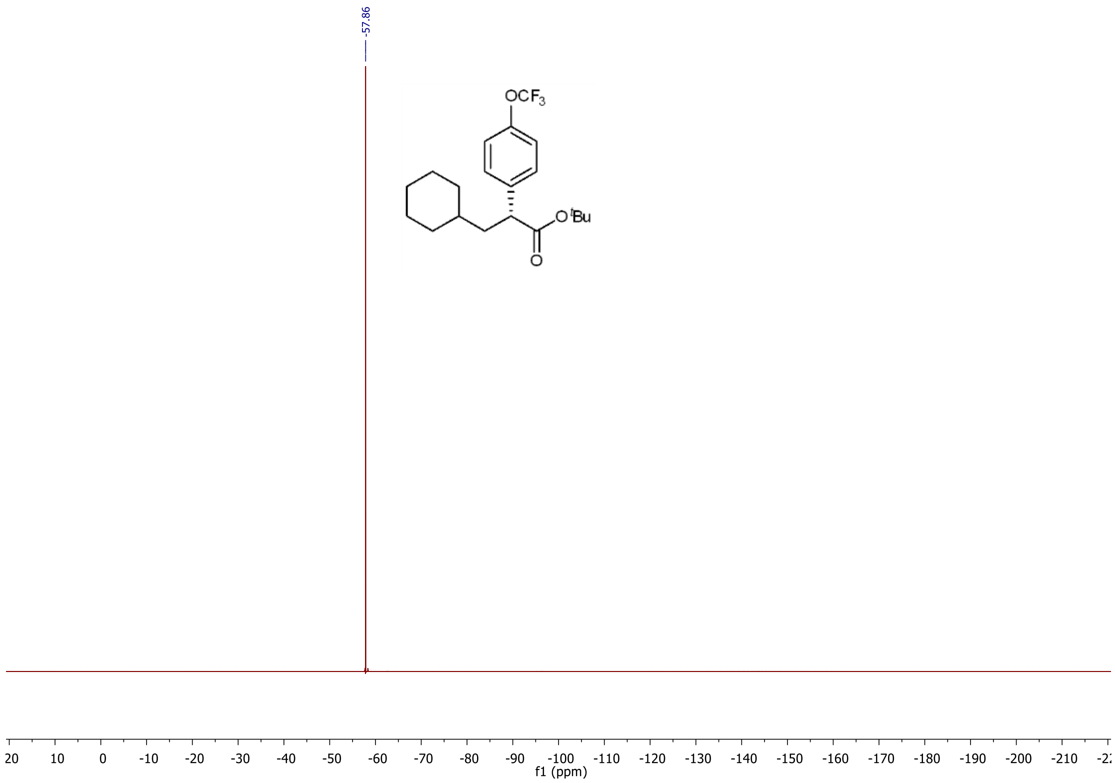

Supplementary Figure 37. ^{19}F NMR of compound **12**.

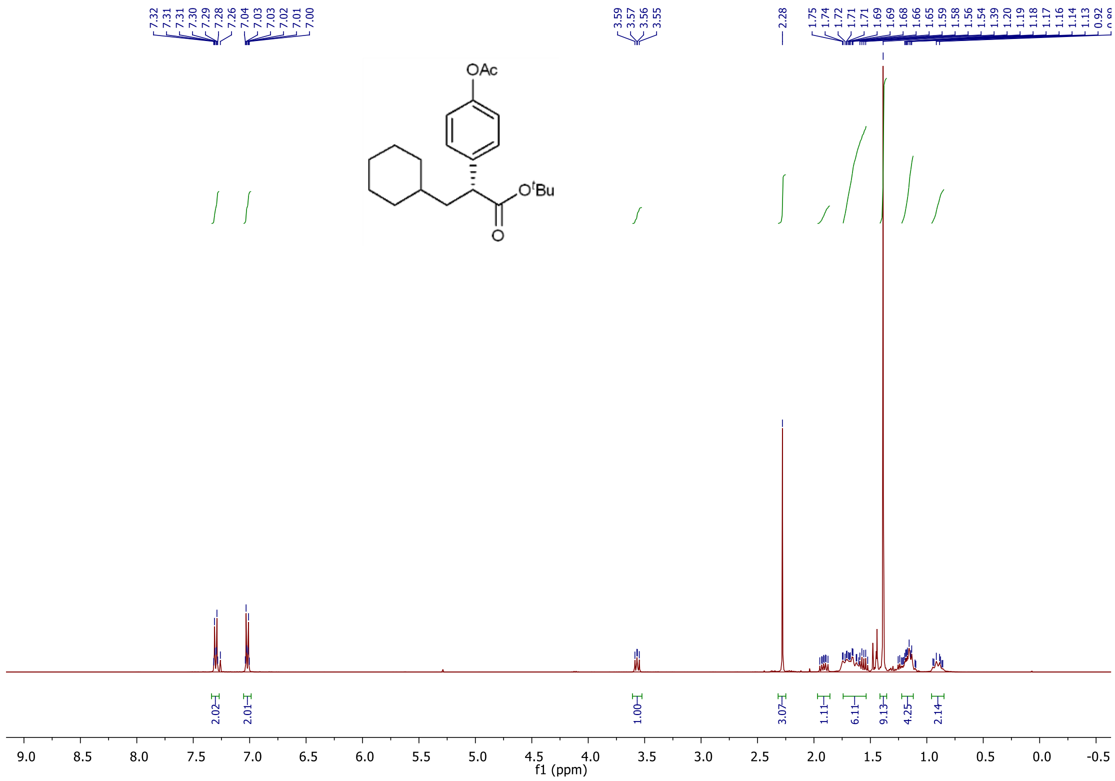

Supplementary Figure 38. ^{1}H NMR of compound **13**.

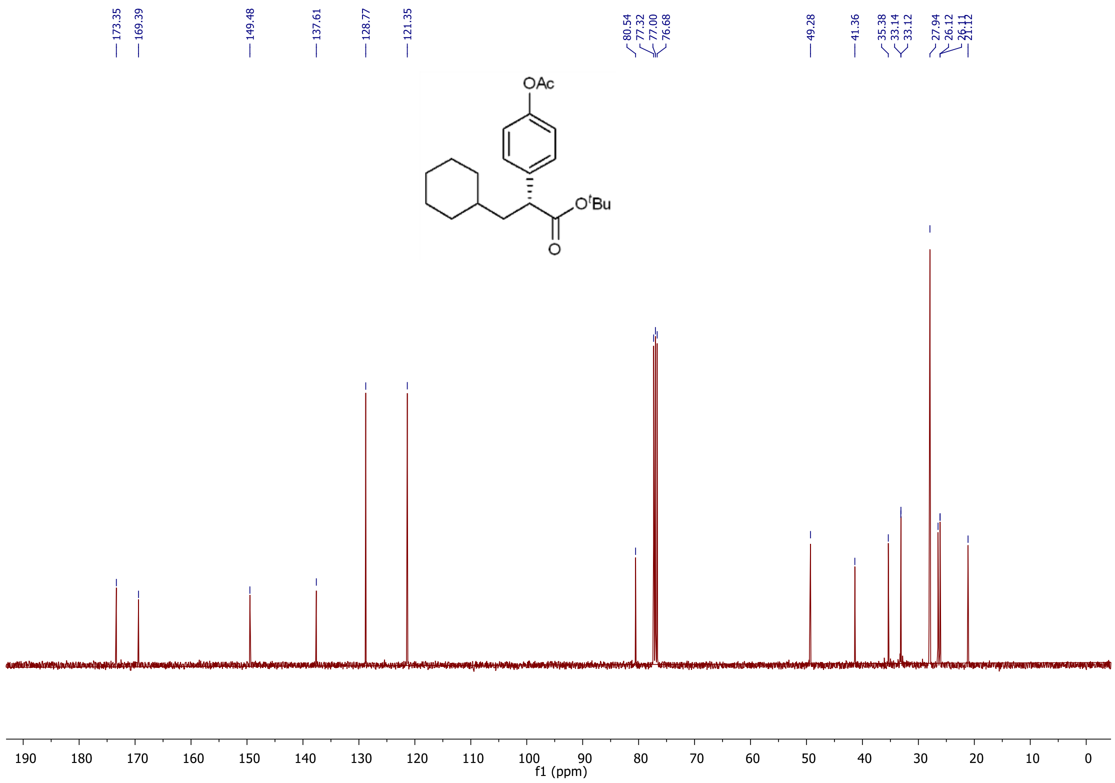

Supplementary Figure 39. ^{1}H NMR of compound **13**.

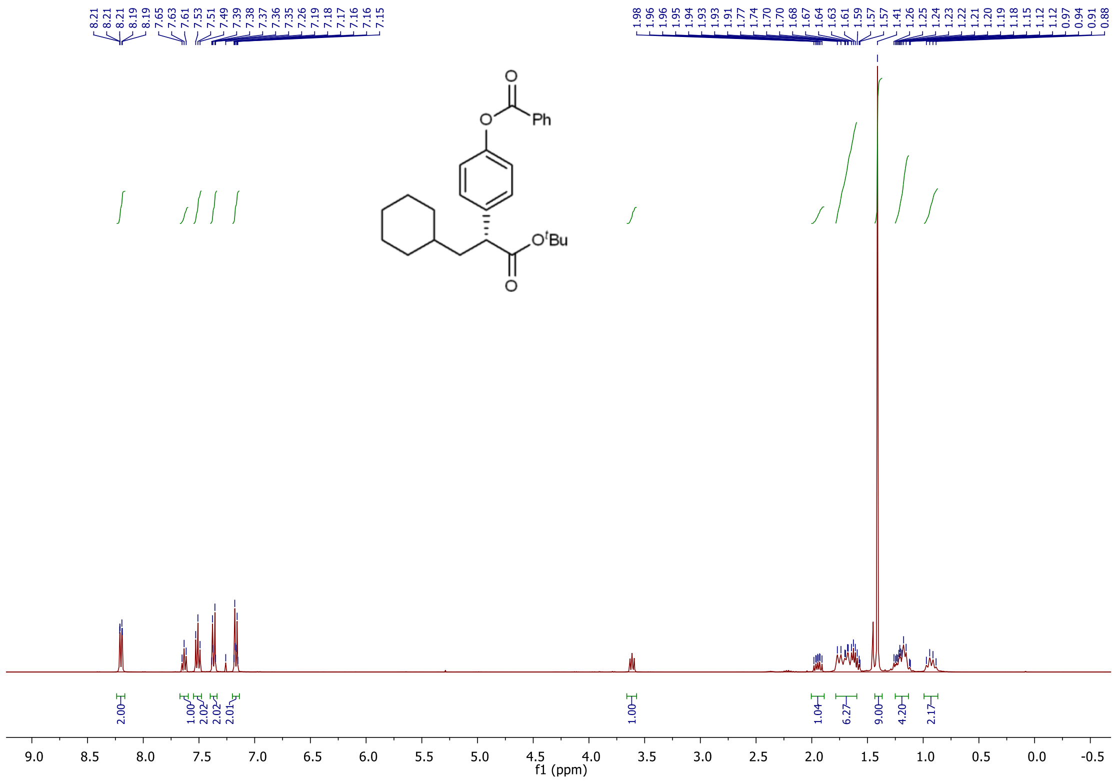

Supplementary Figure 40. ^{1}H NMR of compound **14**.

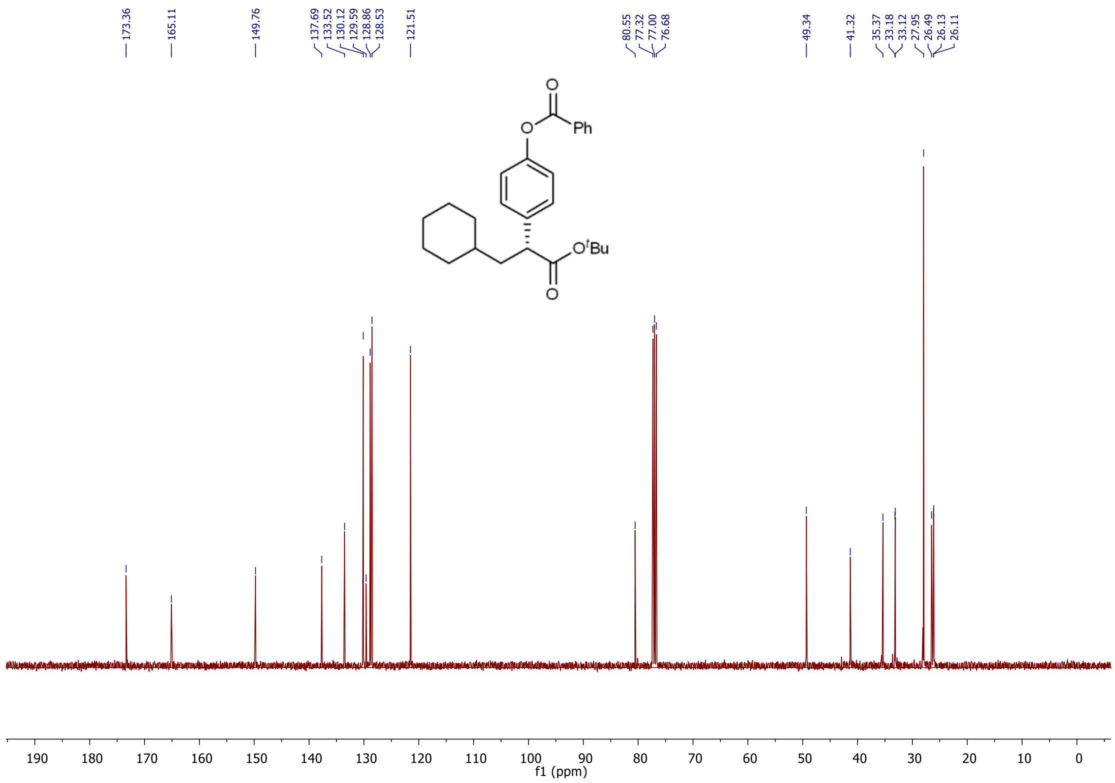

Supplementary Figure 41. ^{13}C NMR of compound **14**.

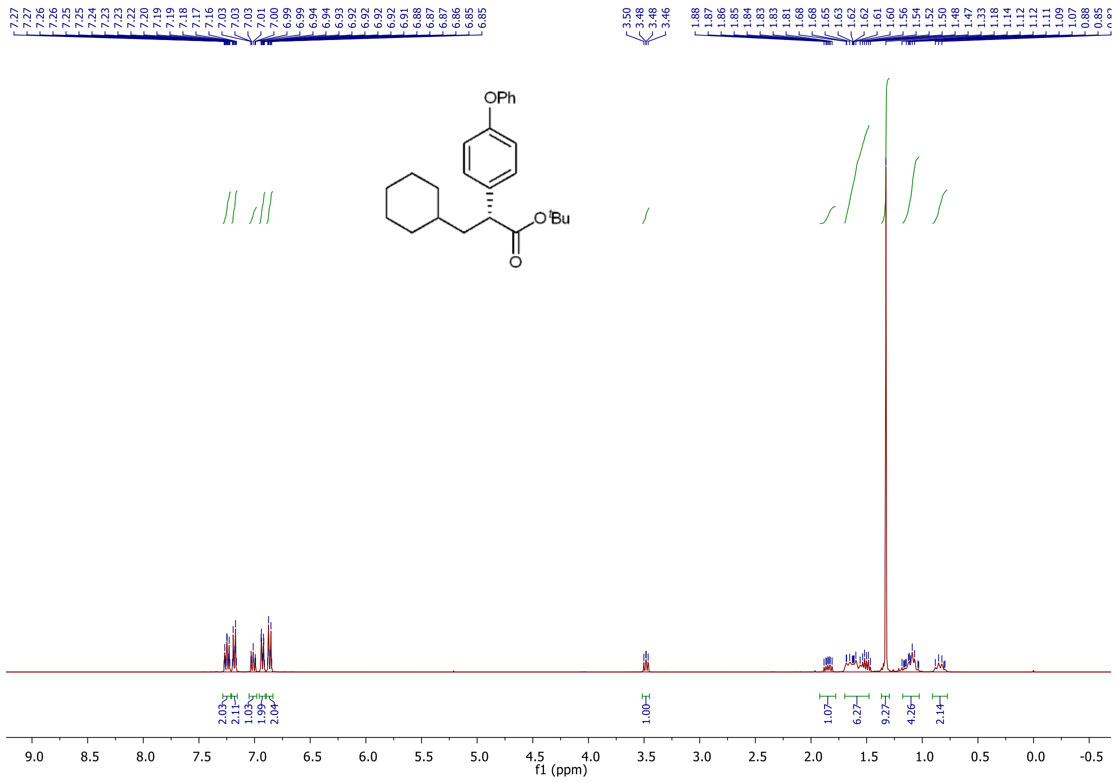

Supplementary Figure 42. ^{1}H NMR of compound **15**.

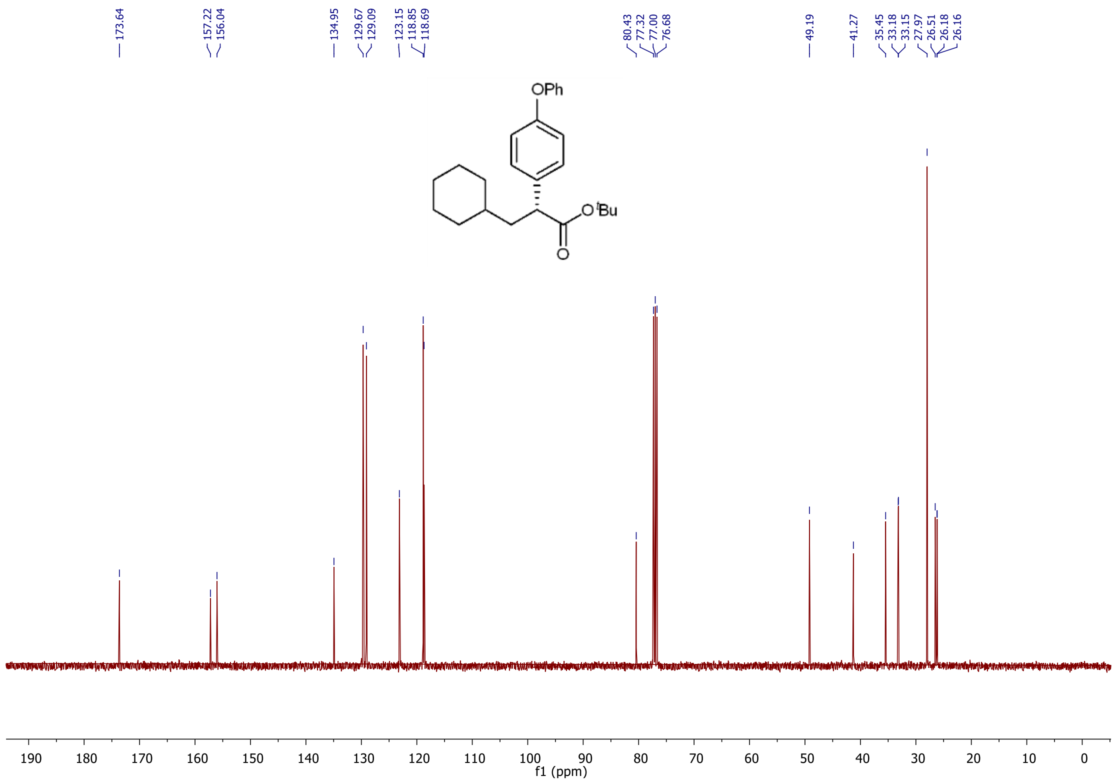

Supplementary Figure 43. ^{13}C NMR of compound **15**.

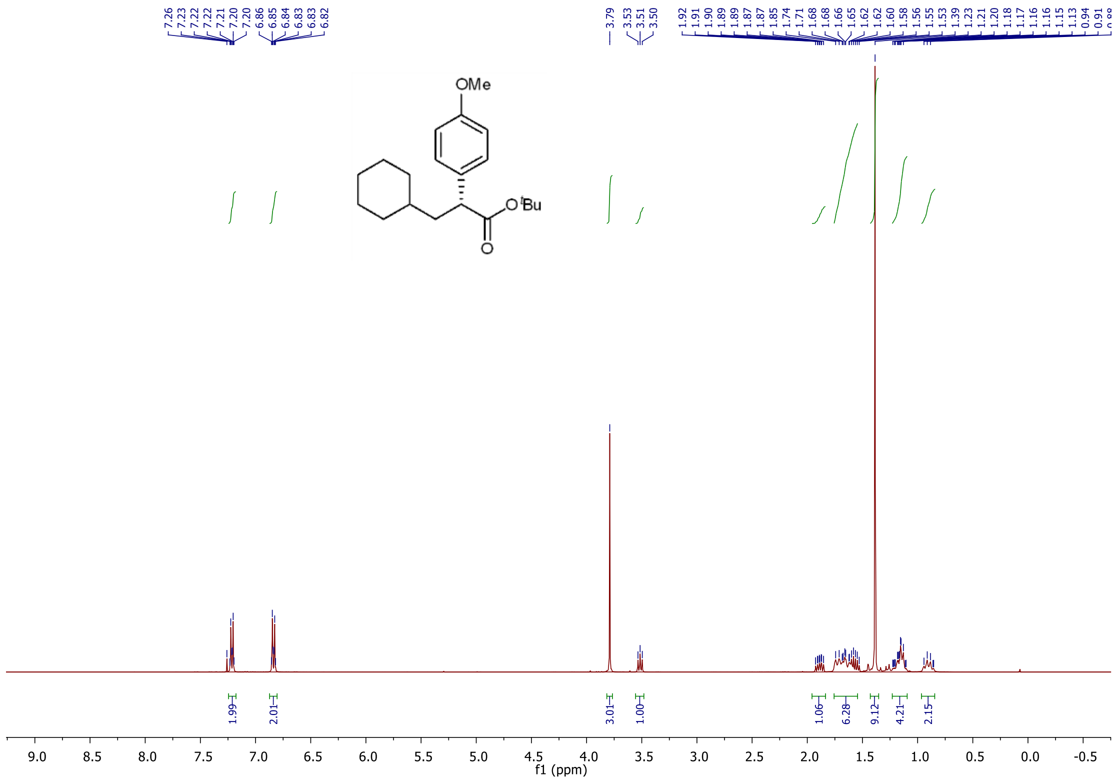

Supplementary Figure 44. ^{1}H NMR of compound **16**.

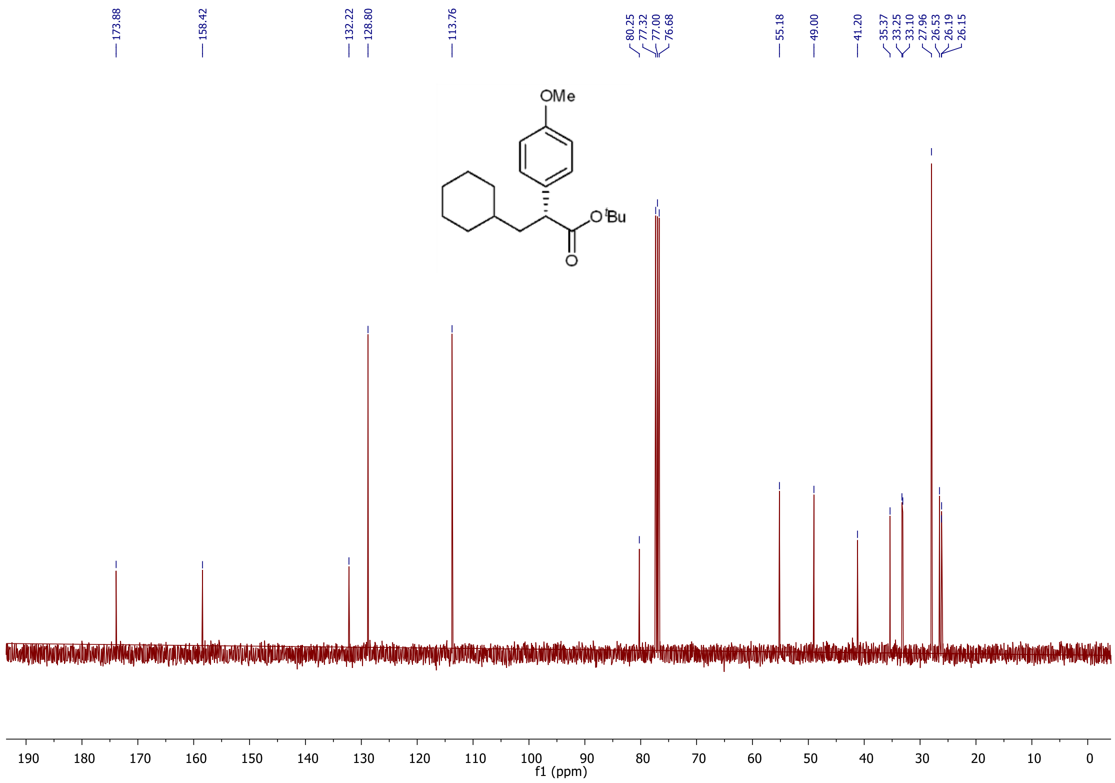

Supplementary Figure 45. ^{13}C NMR of compound **16**.

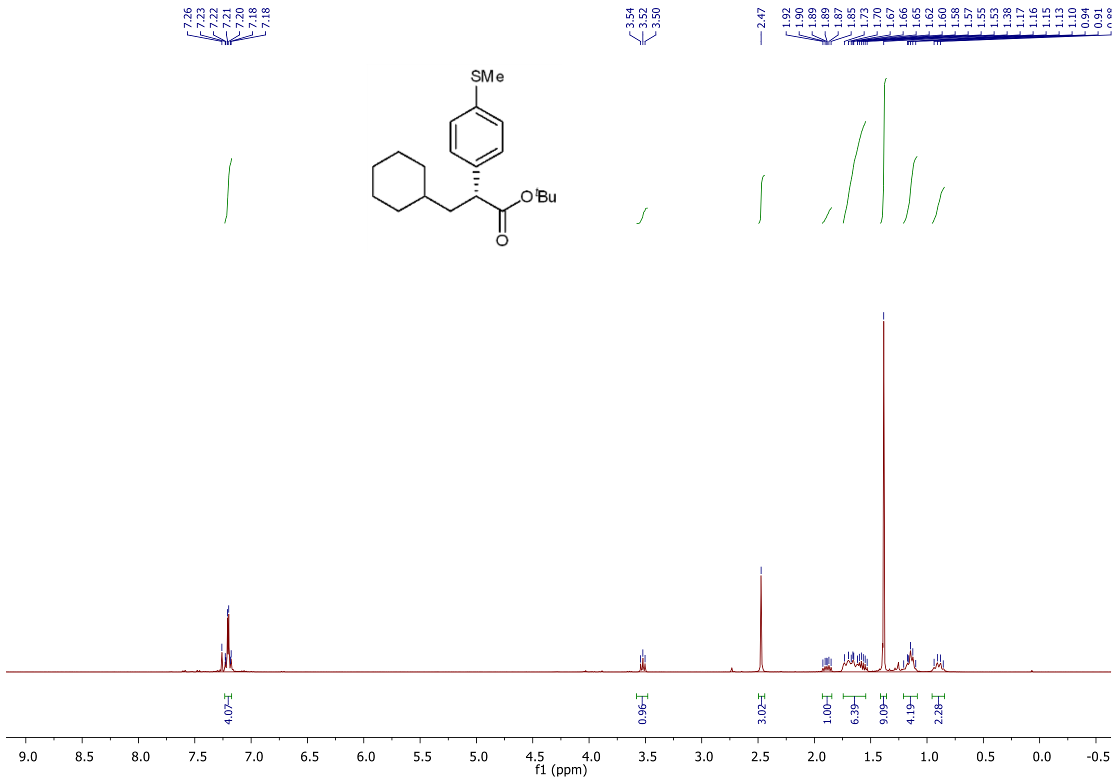

Supplementary Figure 46. ^{1}H NMR of compound **17**.

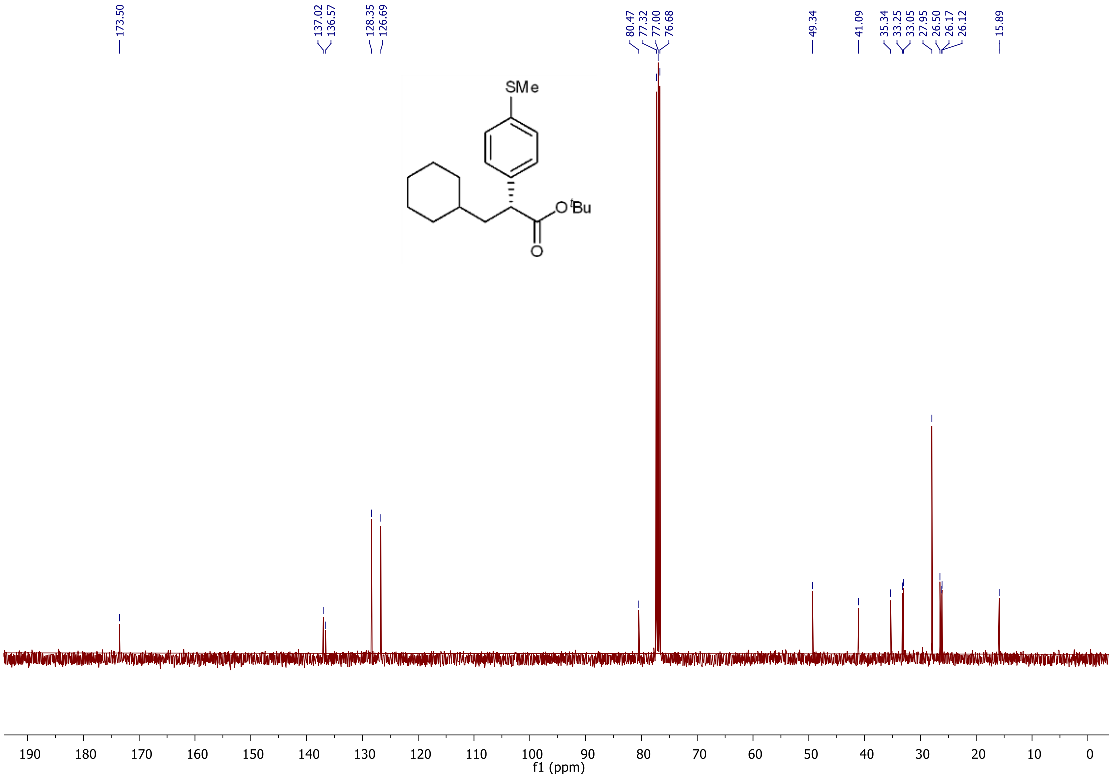

Supplementary Figure 47. ^{13}C NMR of compound **17**.

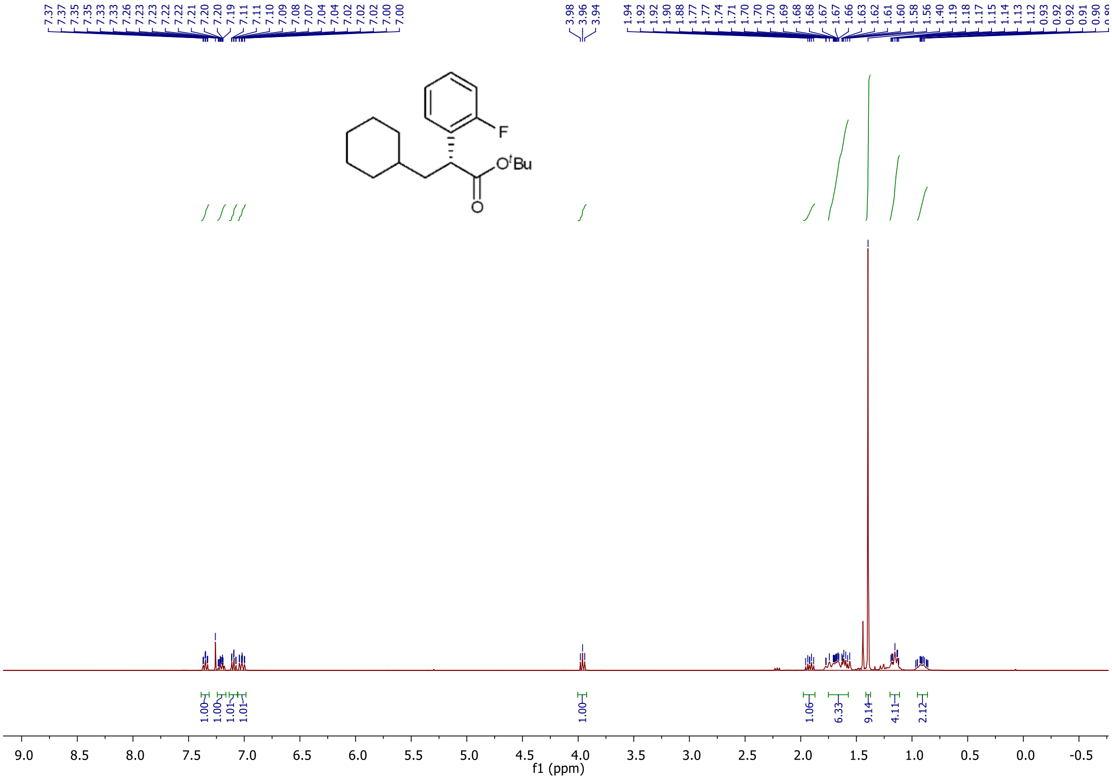

Supplementary Figure 48. ^{1}H NMR of compound **18**.

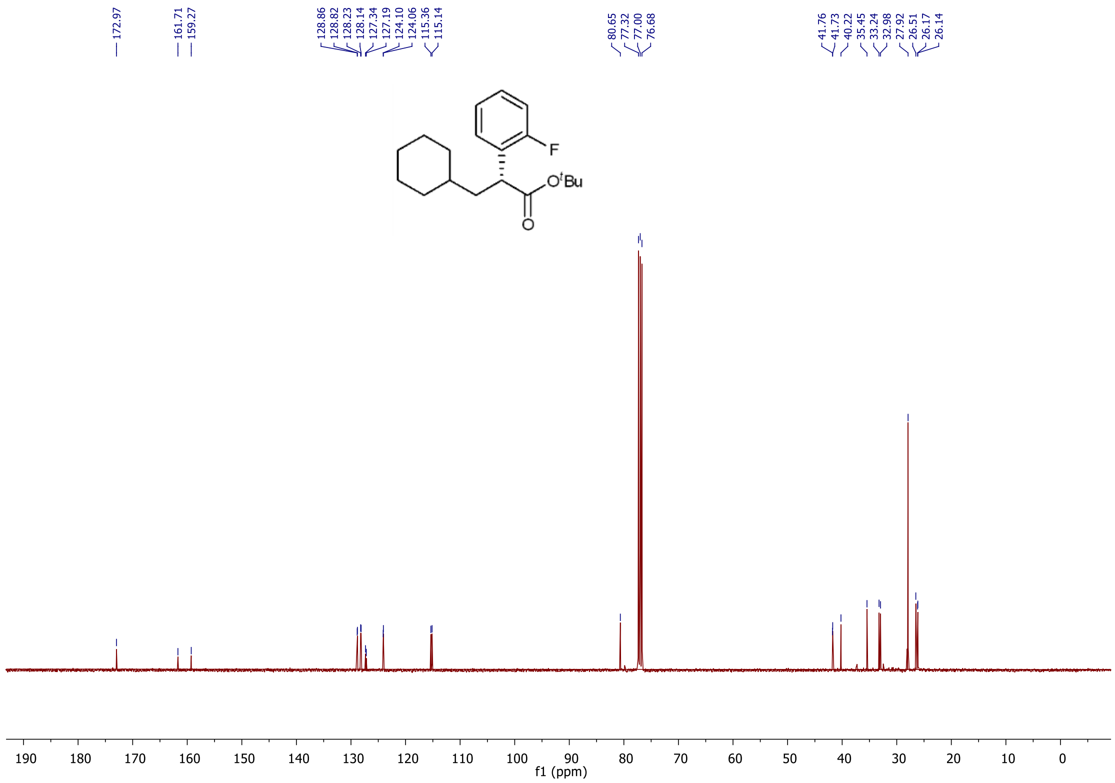

Supplementary Figure 49. ^{13}C NMR of compound **18**.

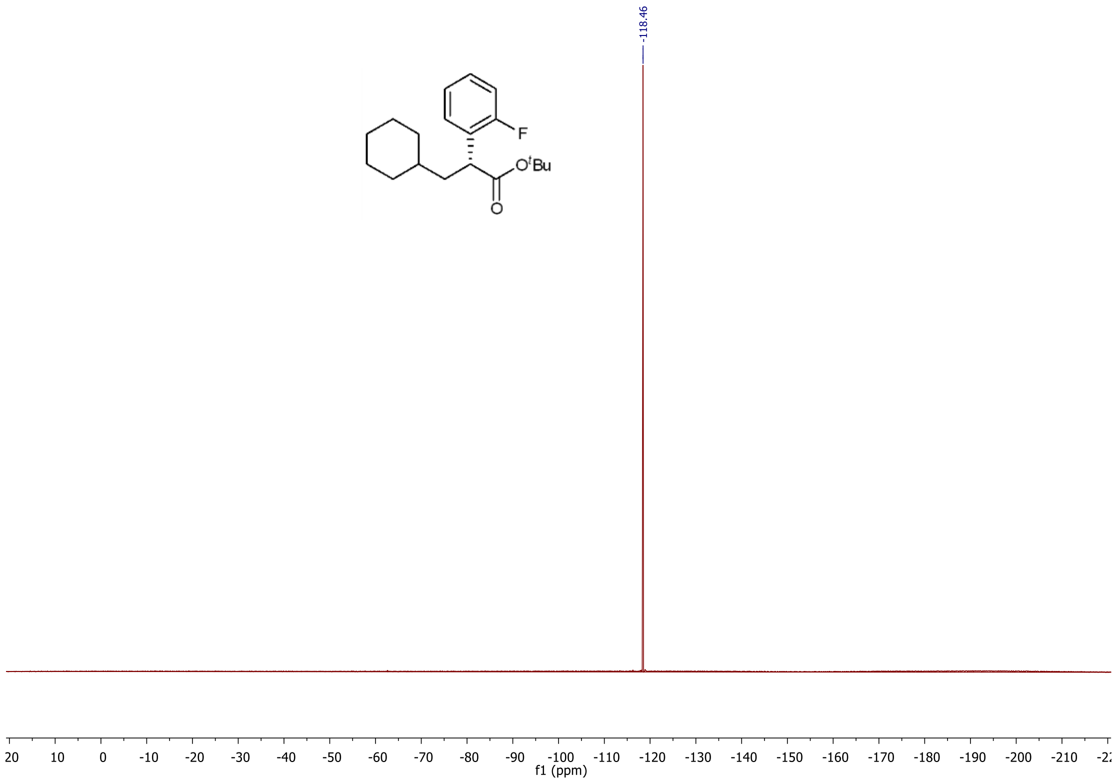

Supplementary Figure 50. ^{19}F NMR of compound **18**.

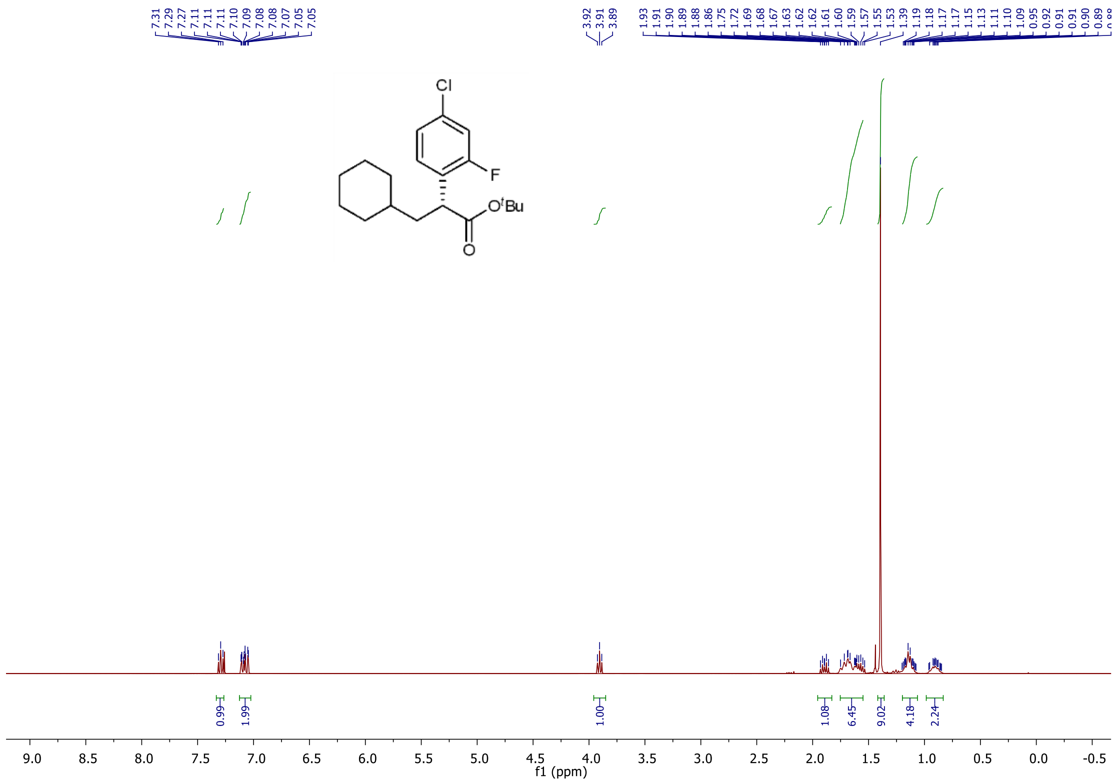

Supplementary Figure 51. ^{1}H NMR of compound **19**.

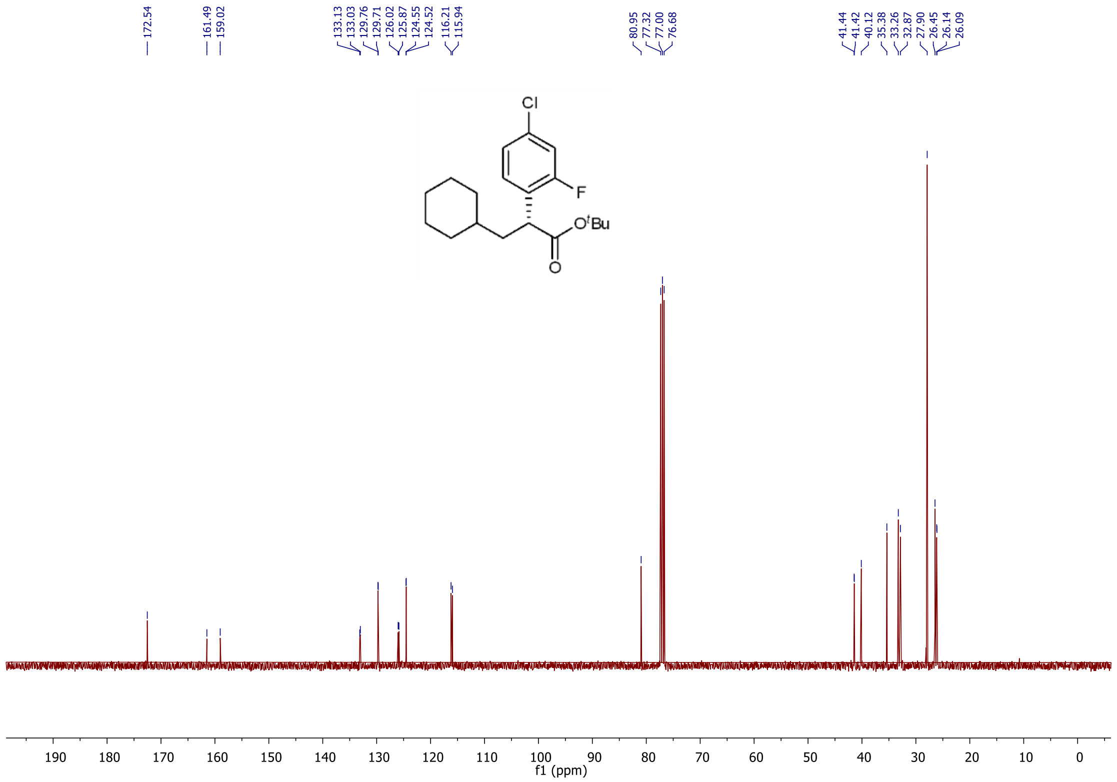

Supplementary Figure 52. ^{13}C NMR of compound **19**.

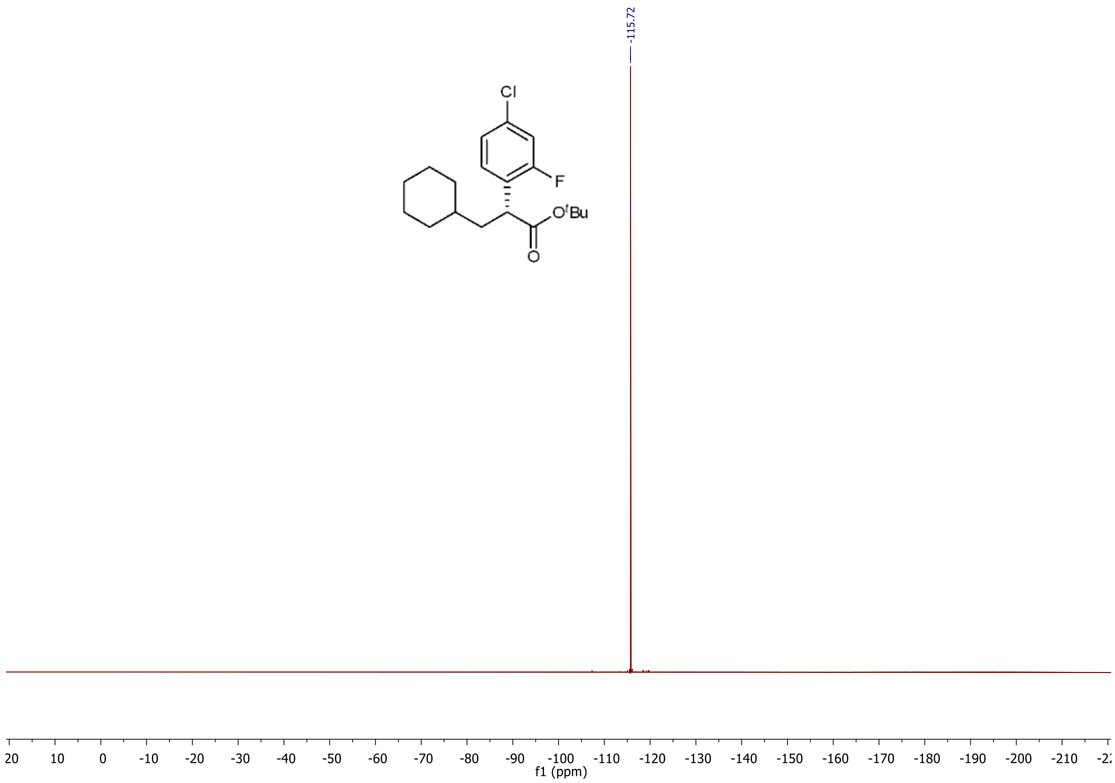

Supplementary Figure 53. ^{19}F NMR of compound **19**.

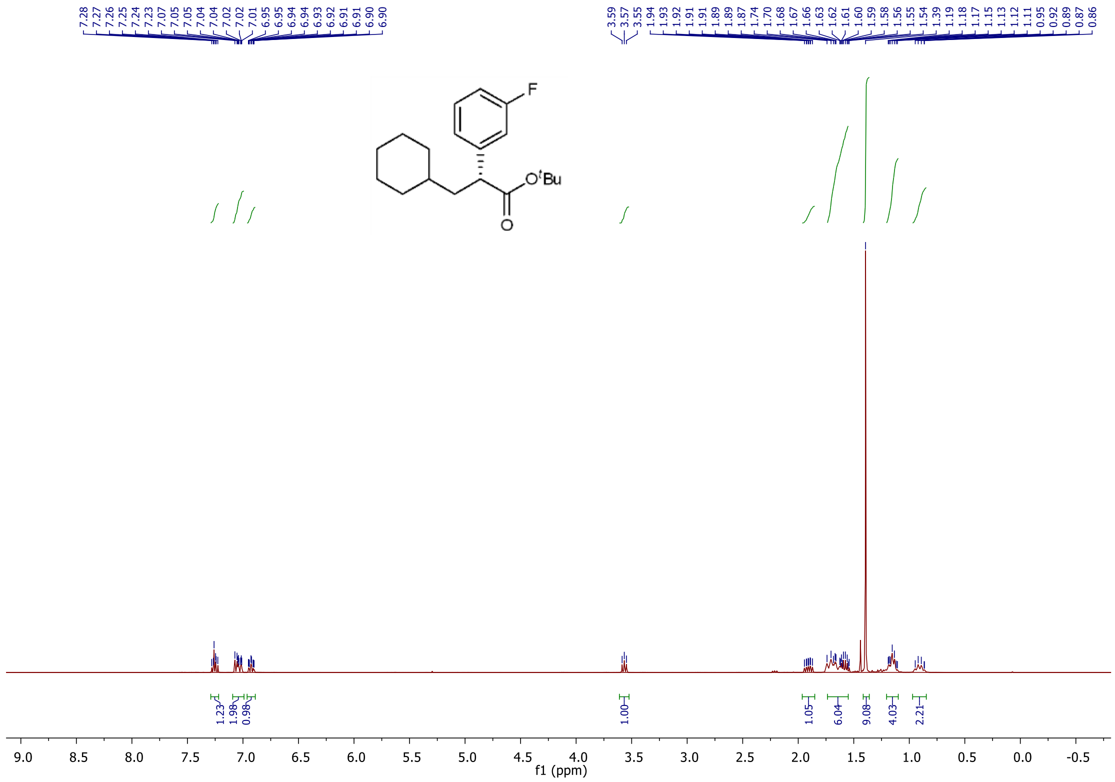

Supplementary Figure 54. ^{1}H NMR of compound **20**.

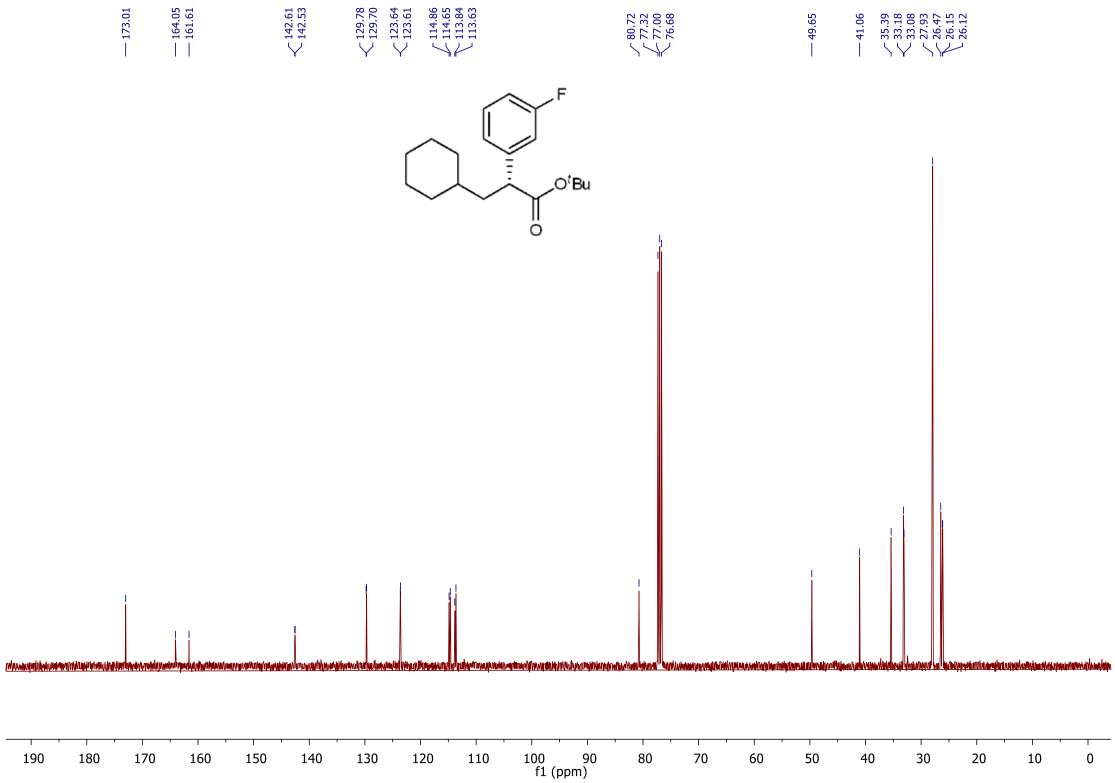

Supplementary Figure 55. ^{13}C NMR of compound **20**.

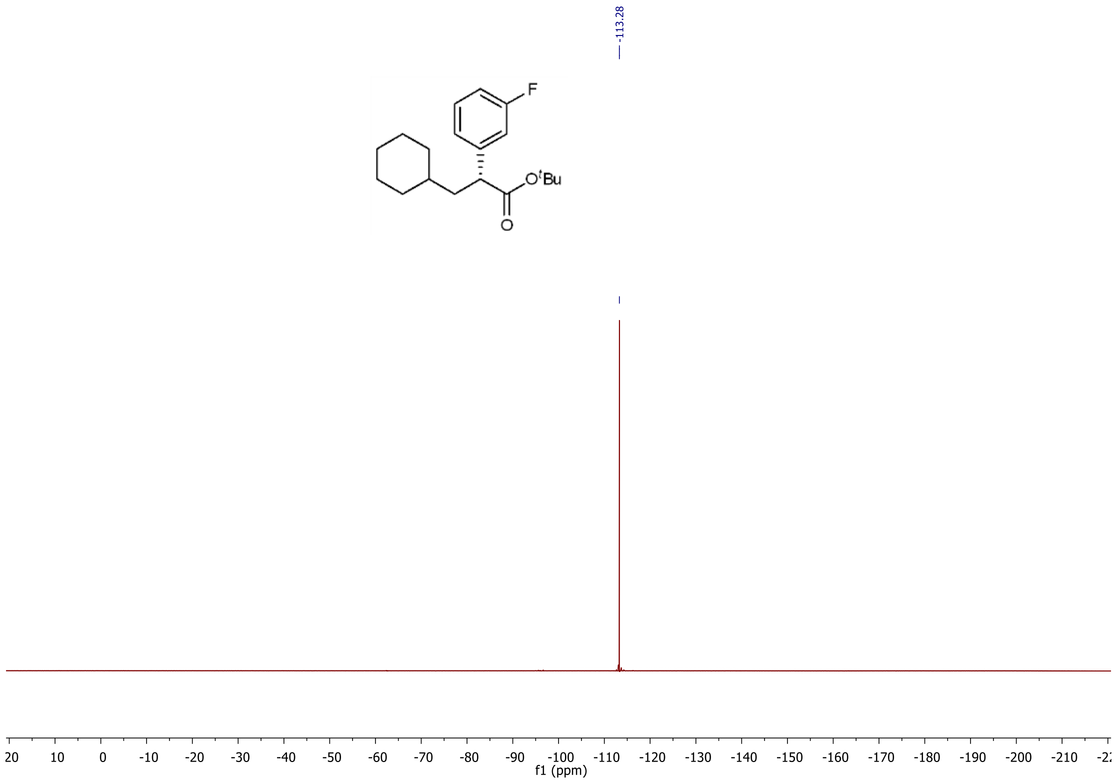

Supplementary Figure 56. ^{19}F NMR of compound **20**.

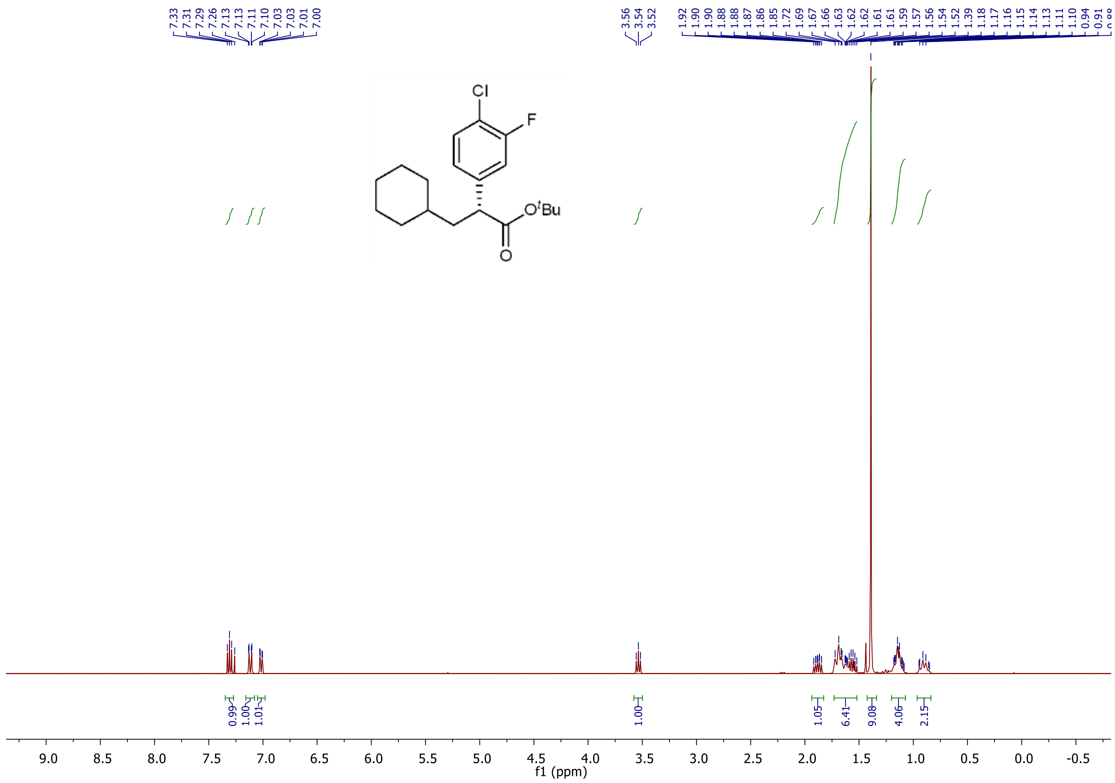

Supplementary Figure 57. ^{1}H NMR of compound **21**.

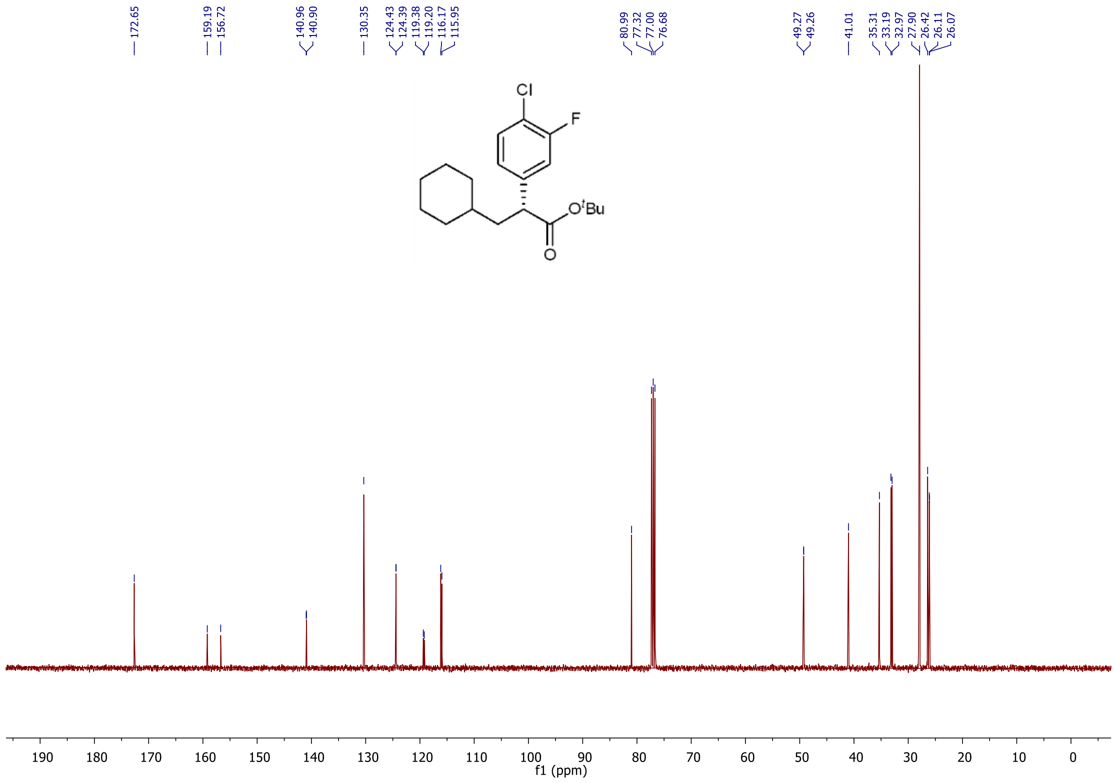

Supplementary Figure 58. ^{13}C NMR of compound **21**.

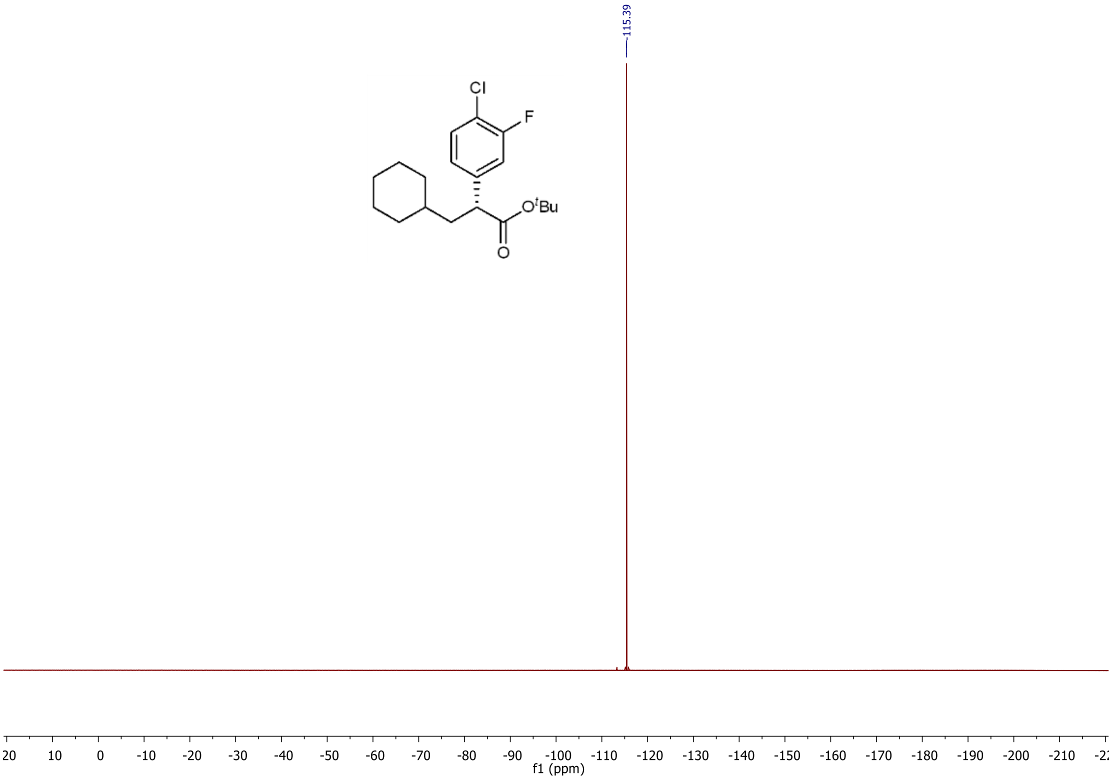

Supplementary Figure 59. ^{19}F NMR of compound **21**.

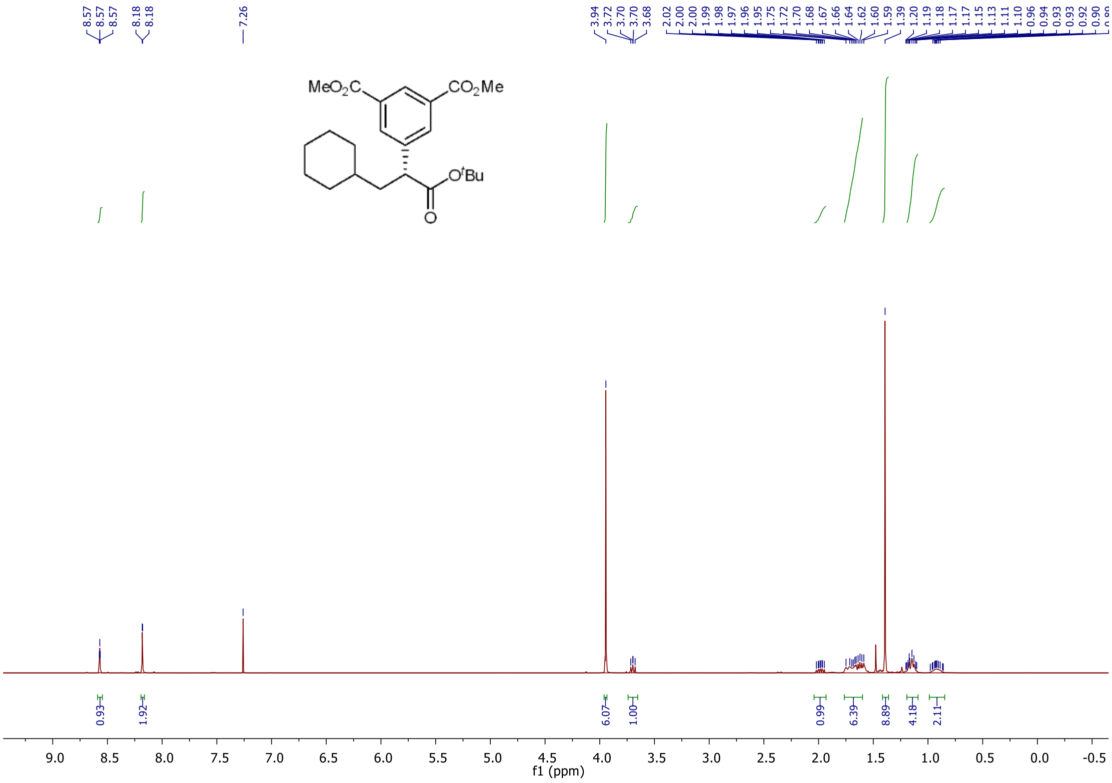

Supplementary Figure 60. ^{1}H NMR of compound **22**.

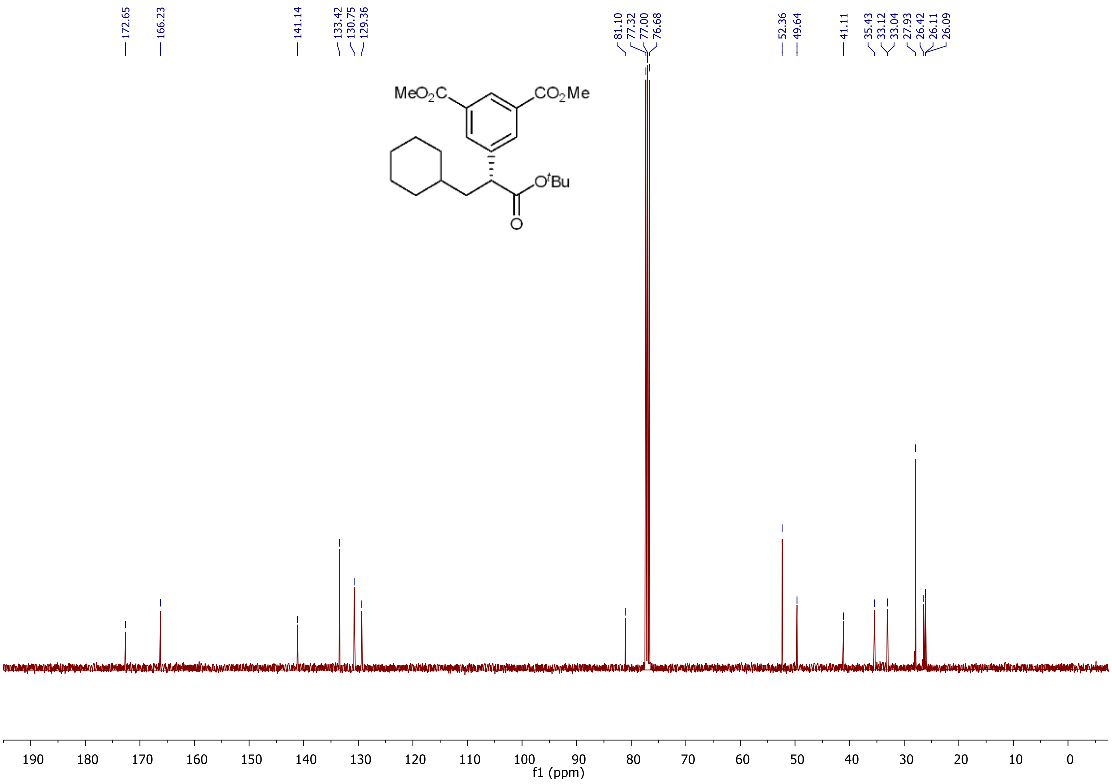

Supplementary Figure 61. ^{13}C NMR of compound **22**.

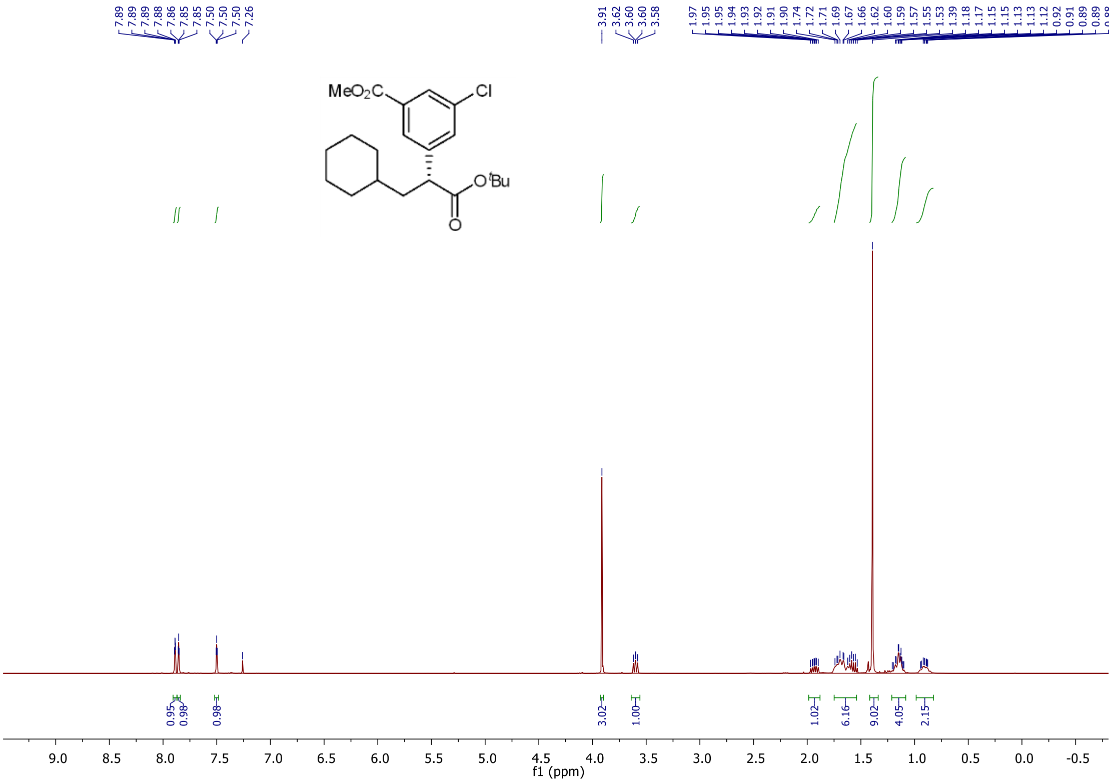

Supplementary Figure 62. ^{1}H NMR of compound **23**.

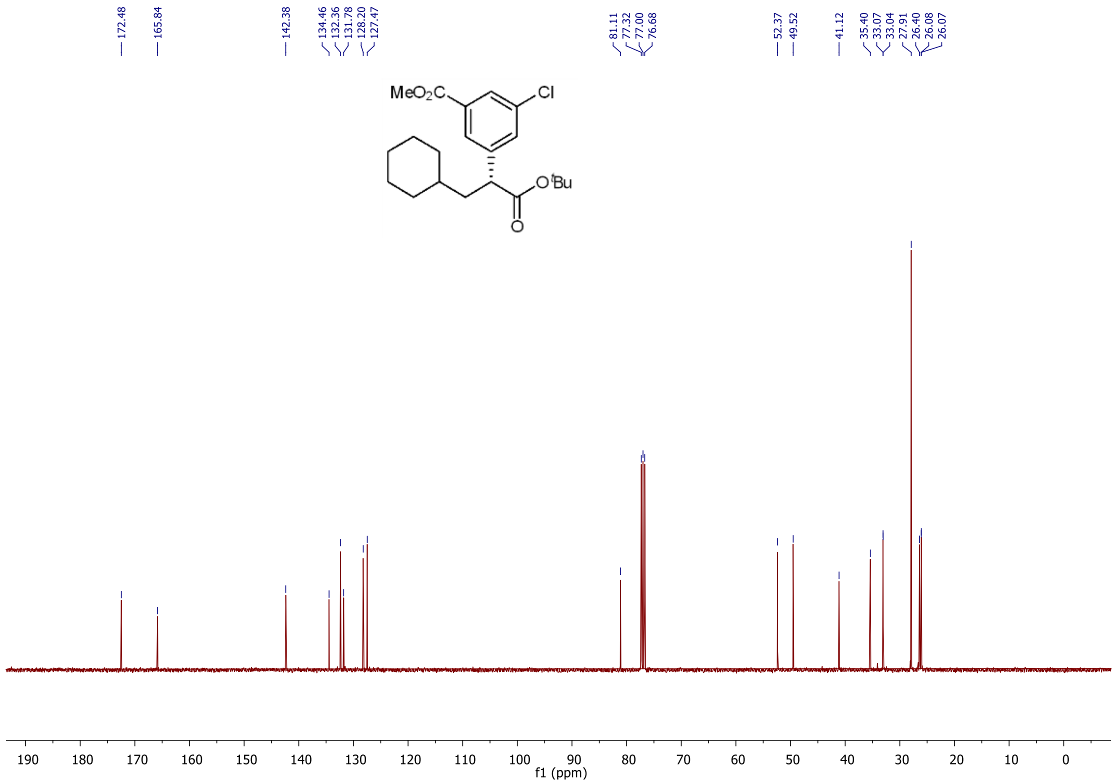

Supplementary Figure 63. ^{13}C NMR of compound **23**.

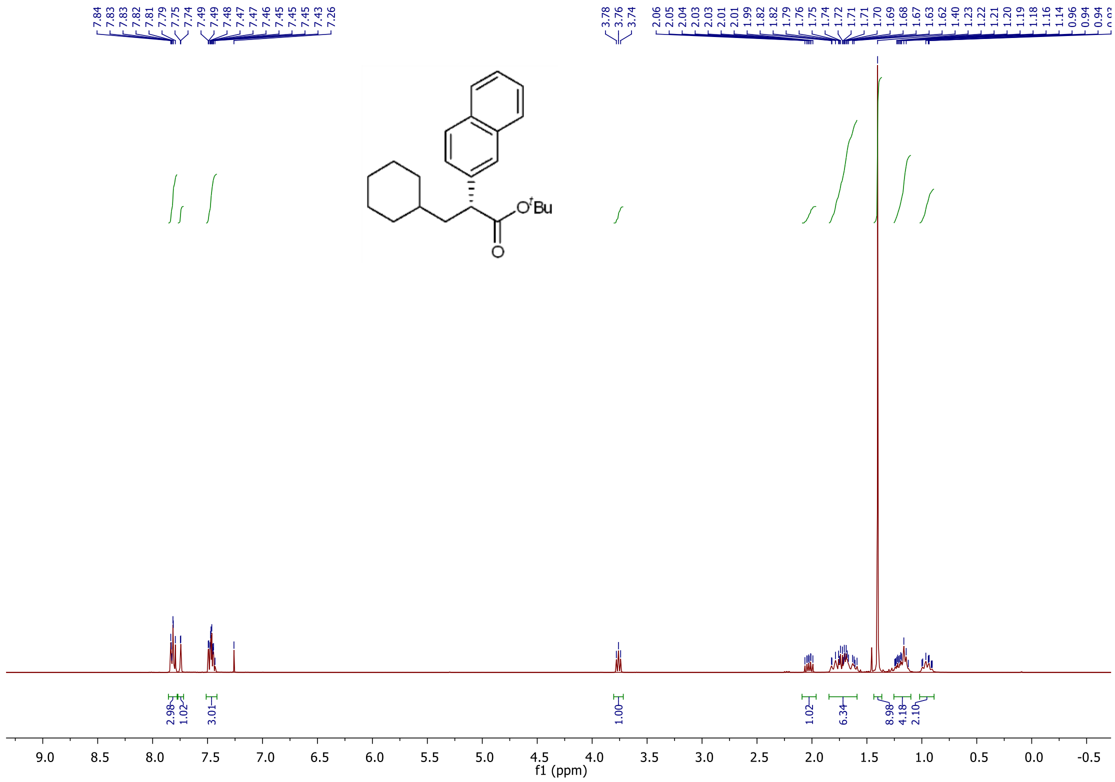

Supplementary Figure 64. ^{1}H NMR of compound **24**.

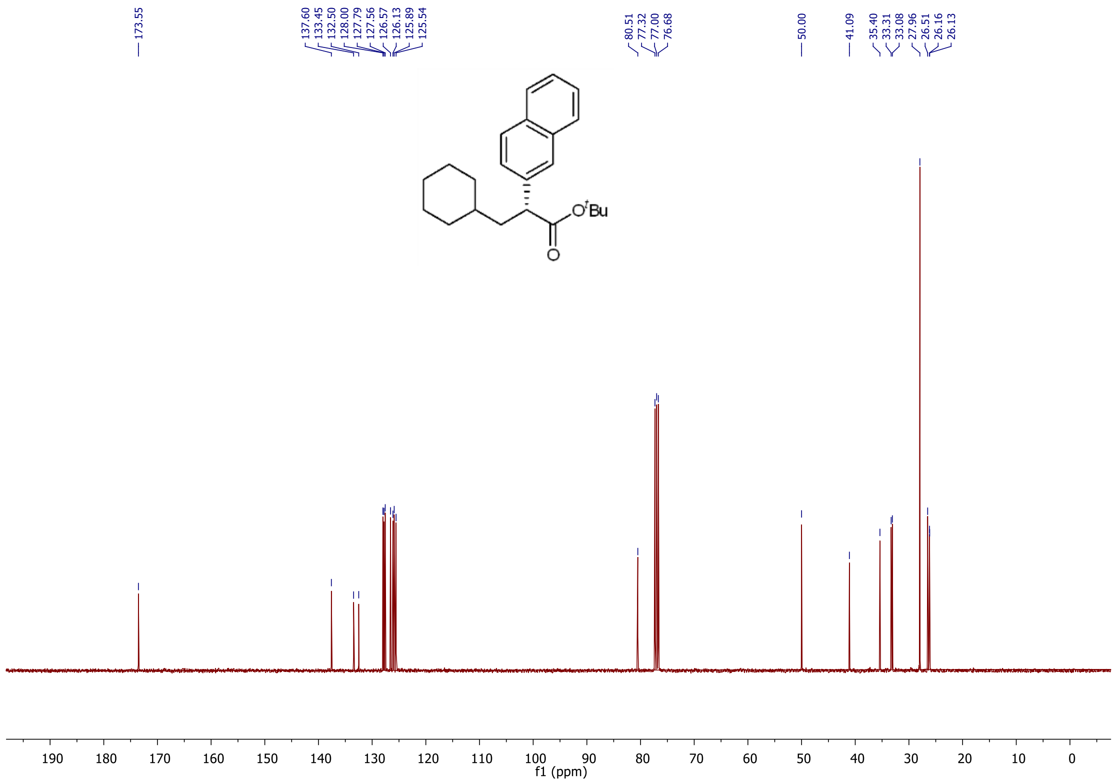

Supplementary Figure 65. ^{13}C NMR of compound **24**.

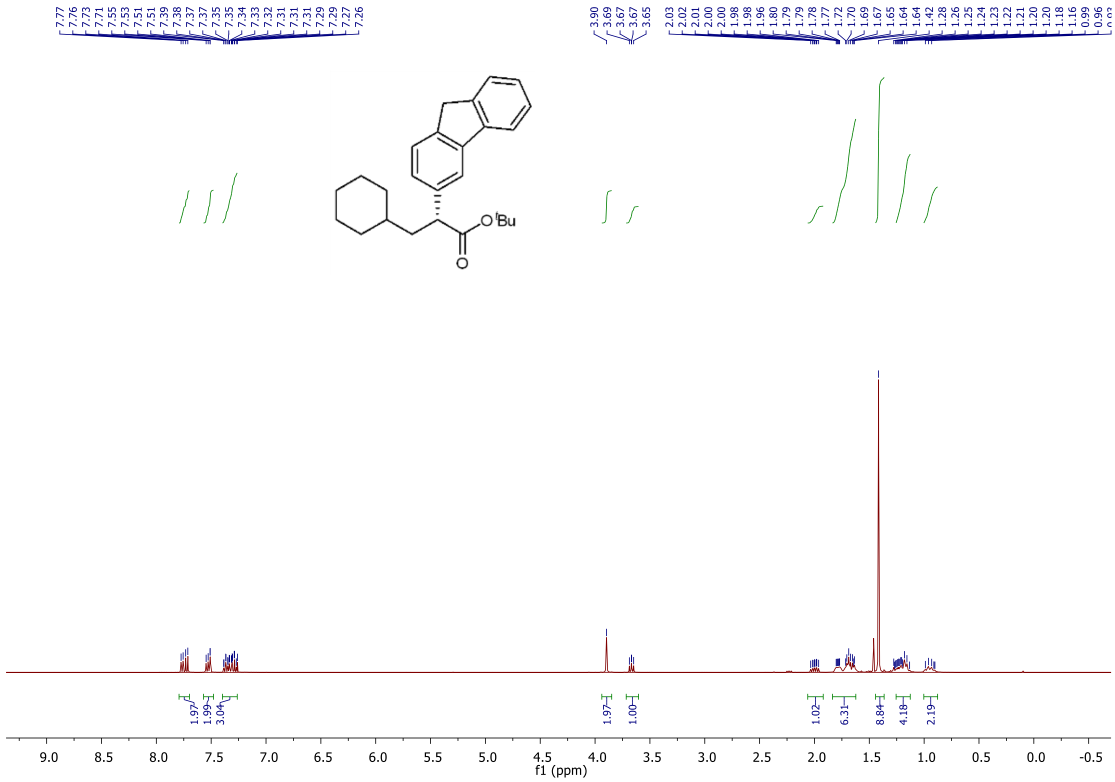

Supplementary Figure 66. ^{1}H NMR of compound **25**.

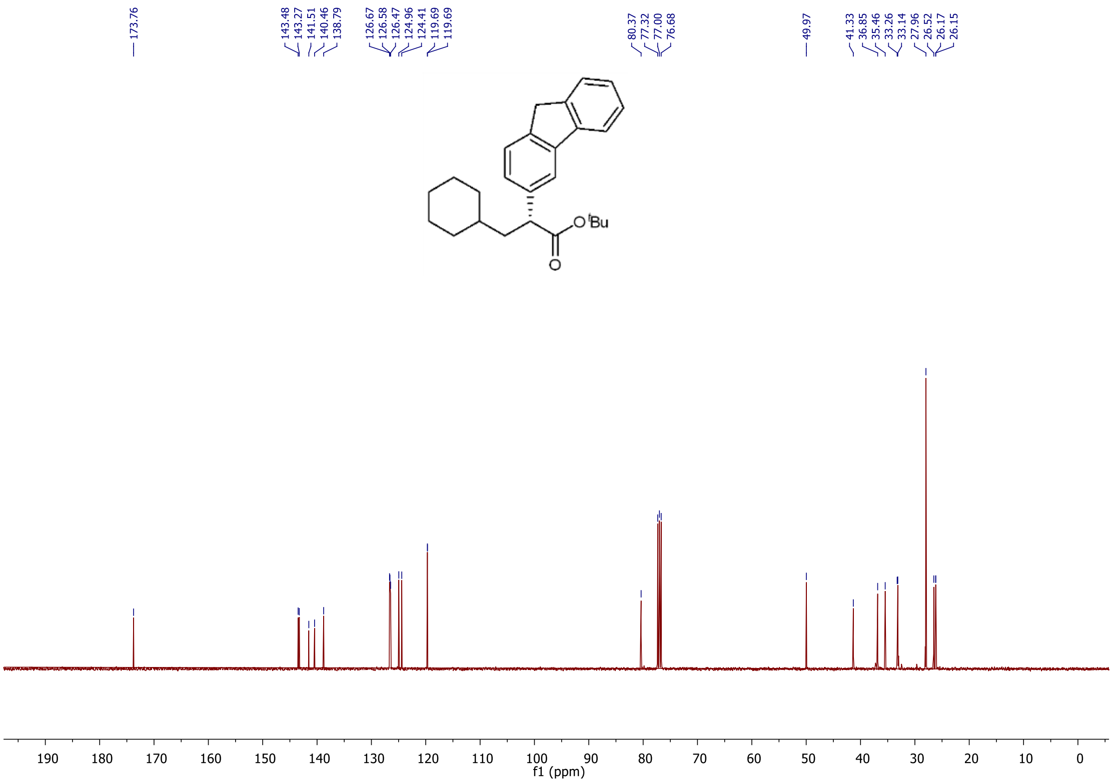

Supplementary Figure 67. ^{13}C NMR of compound **25**.

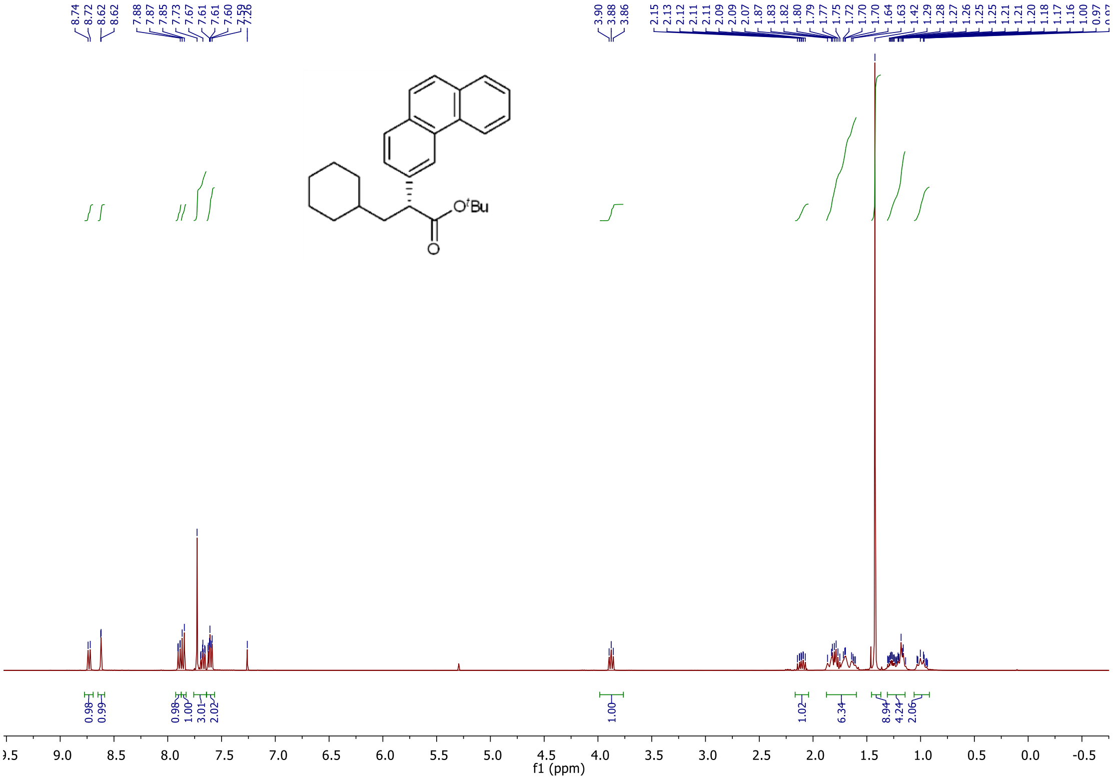

Supplementary Figure 68. ^{1}H NMR of compound **26**.

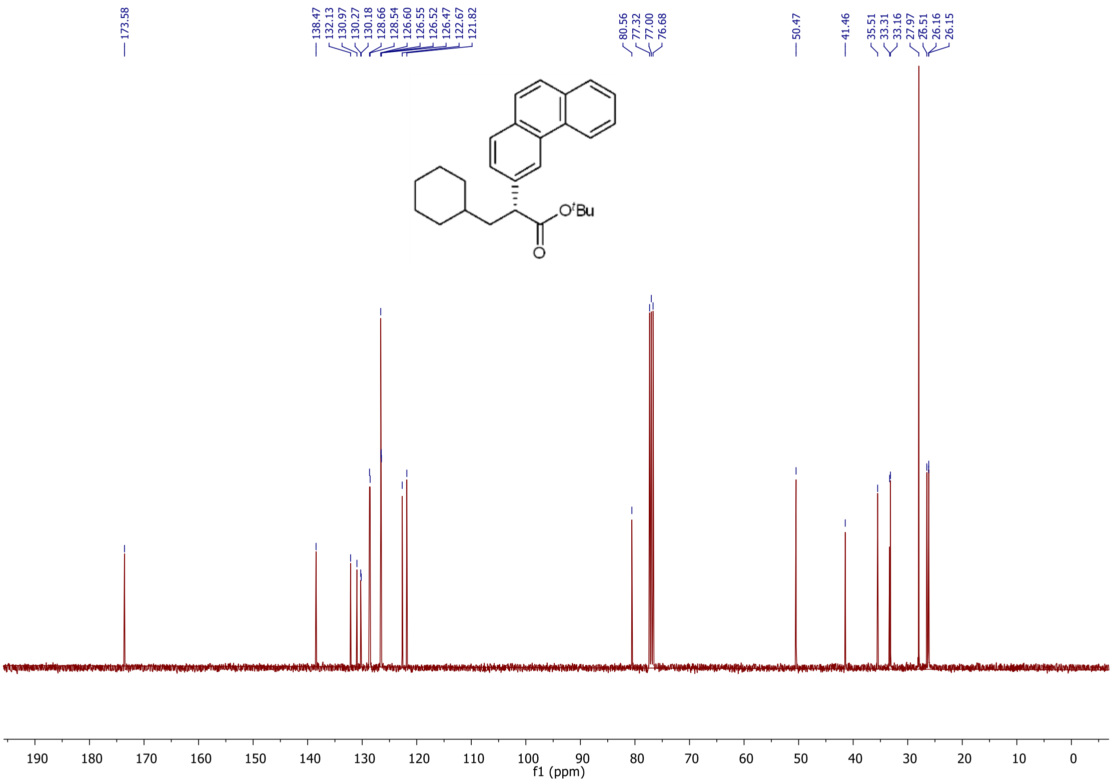

Supplementary Figure 69. ^{13}C NMR of compound **26**.

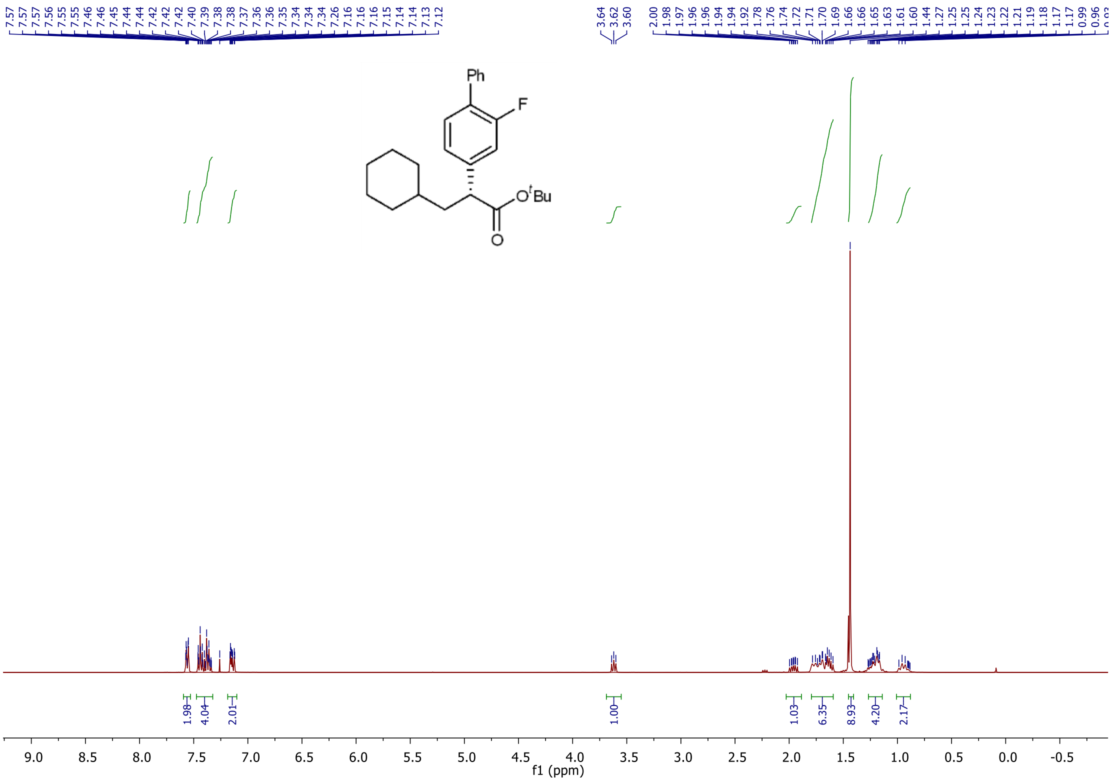

Supplementary Figure 70. ^{1}H NMR of compound **27**.

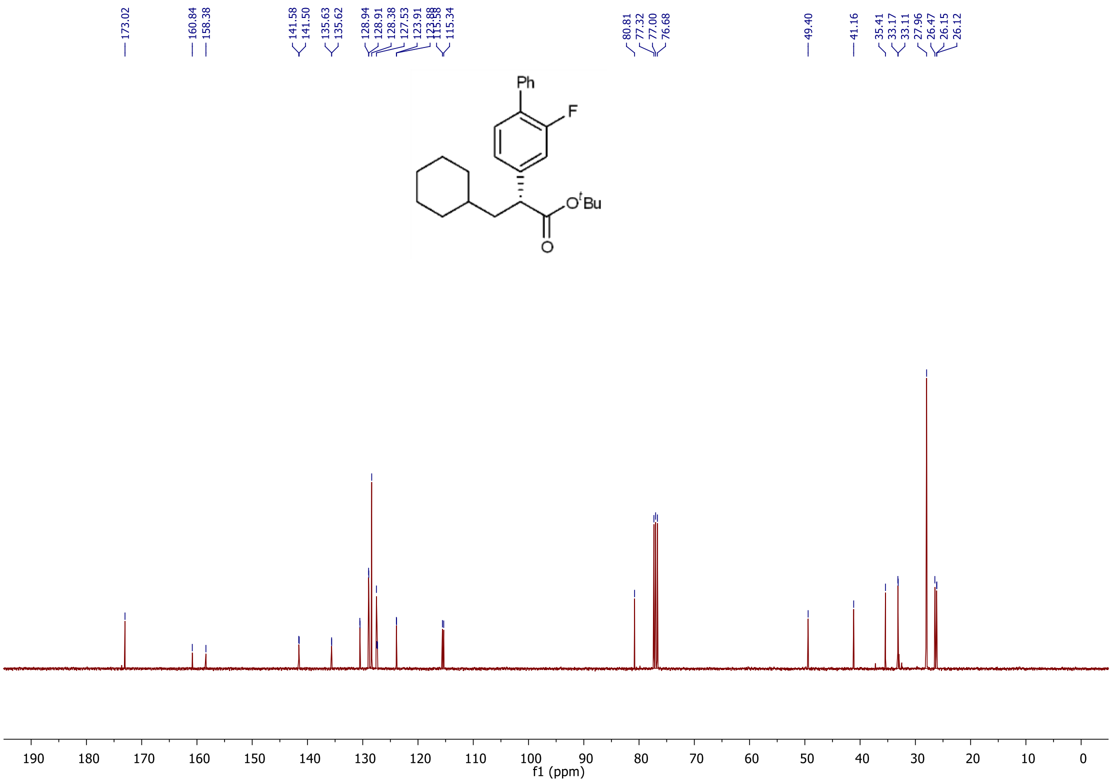

Supplementary Figure 71. ^{13}C NMR of compound **27**.

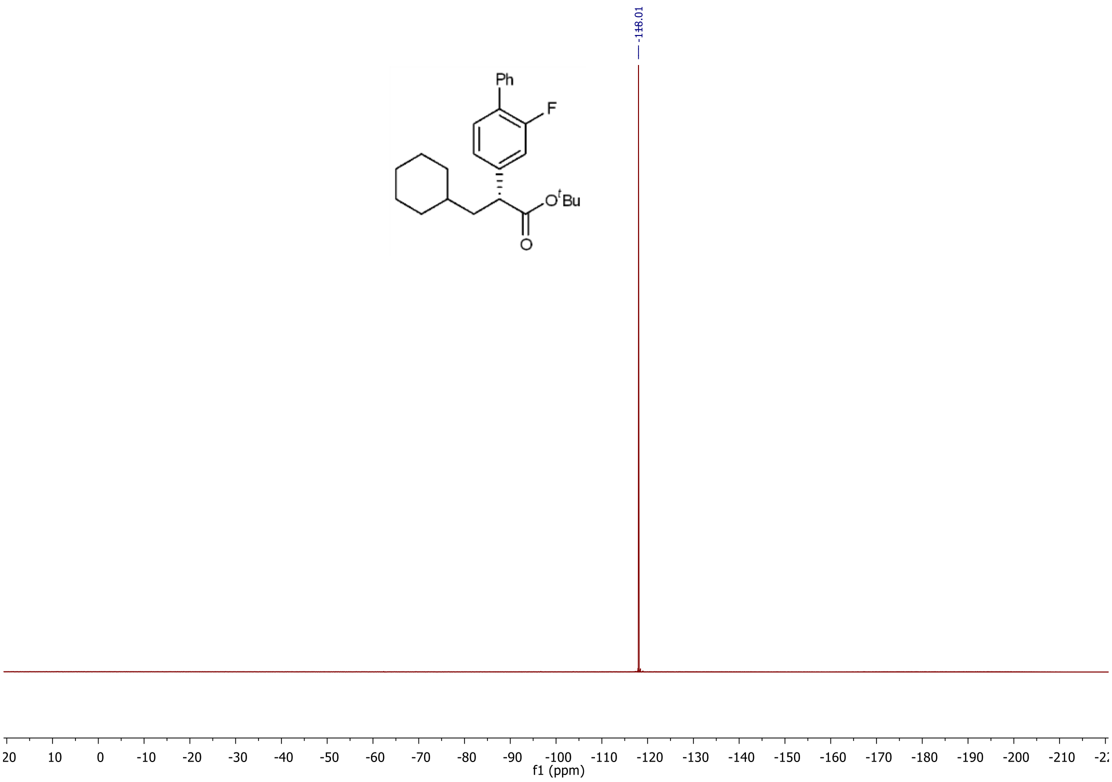

Supplementary Figure 72. ^{19}F NMR of compound **27**.

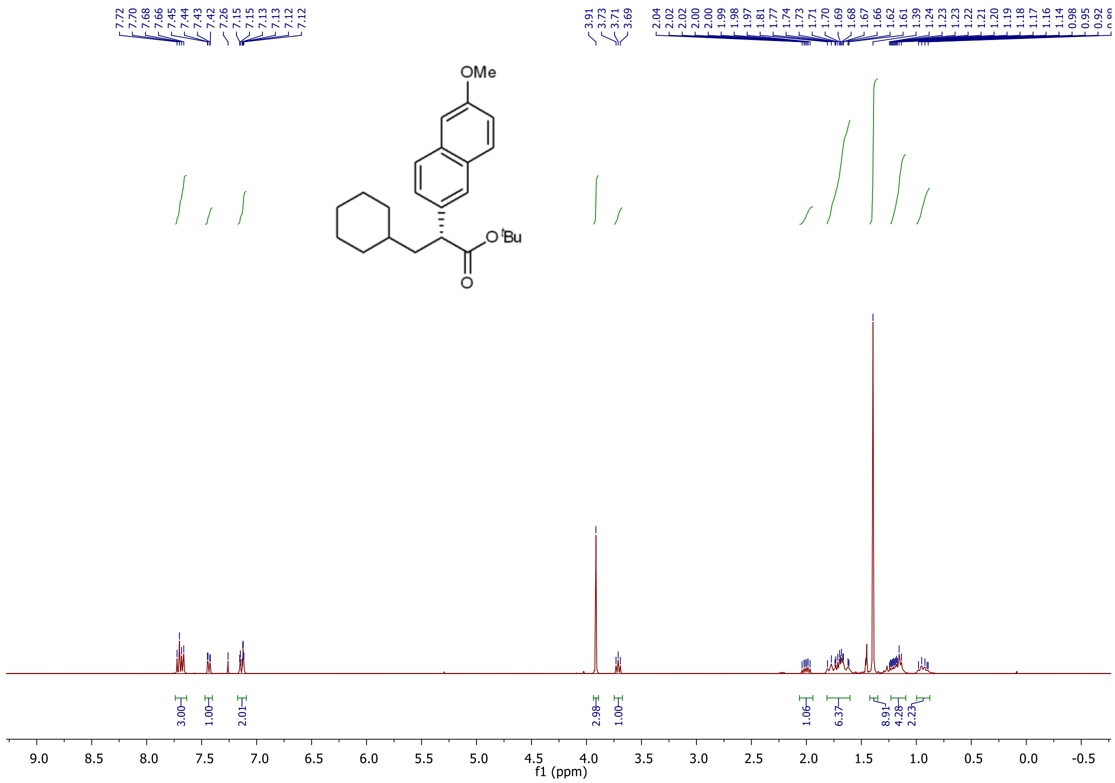

Supplementary Figure 73. ^{1}H NMR of compound **28**.

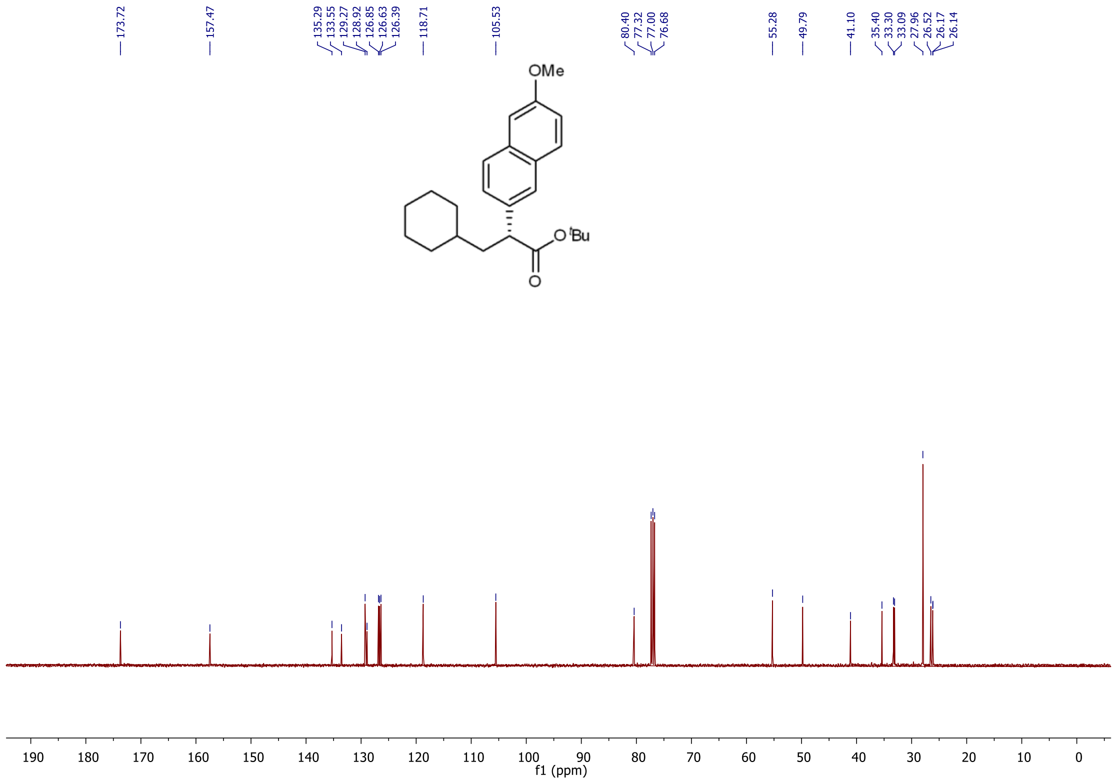

Supplementary Figure 74. ^{13}C NMR of compound **28**.

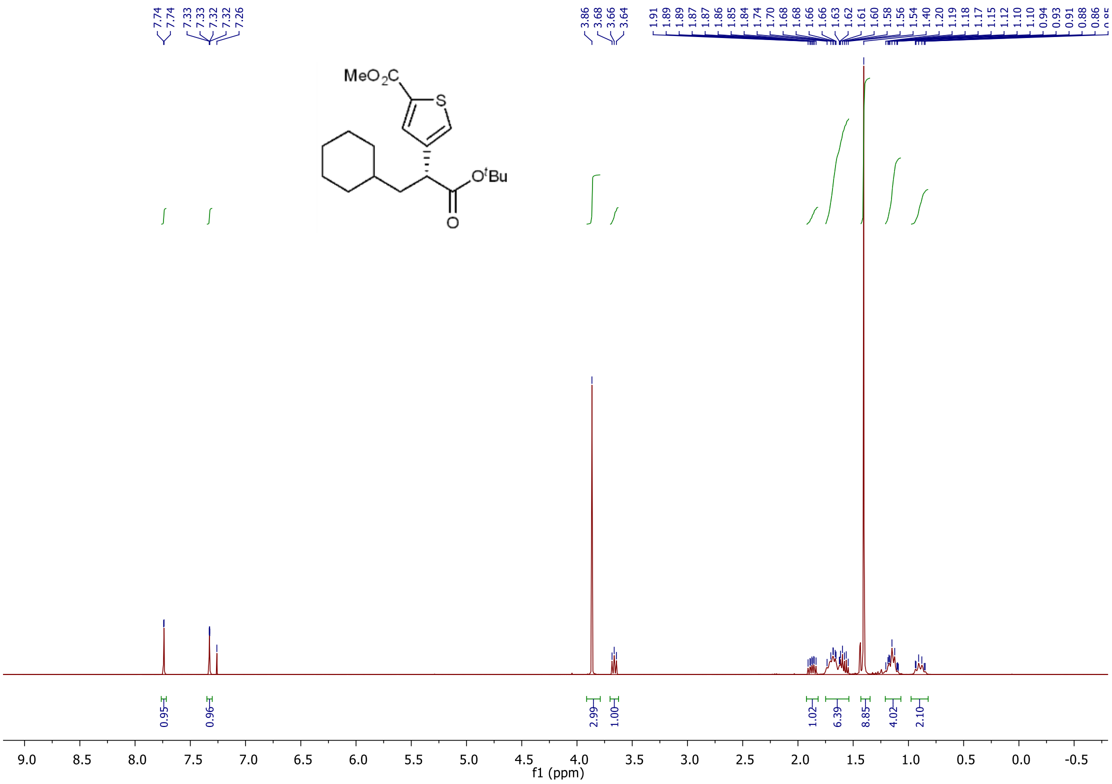

Supplementary Figure 75. ^{1}H NMR of compound **29**.

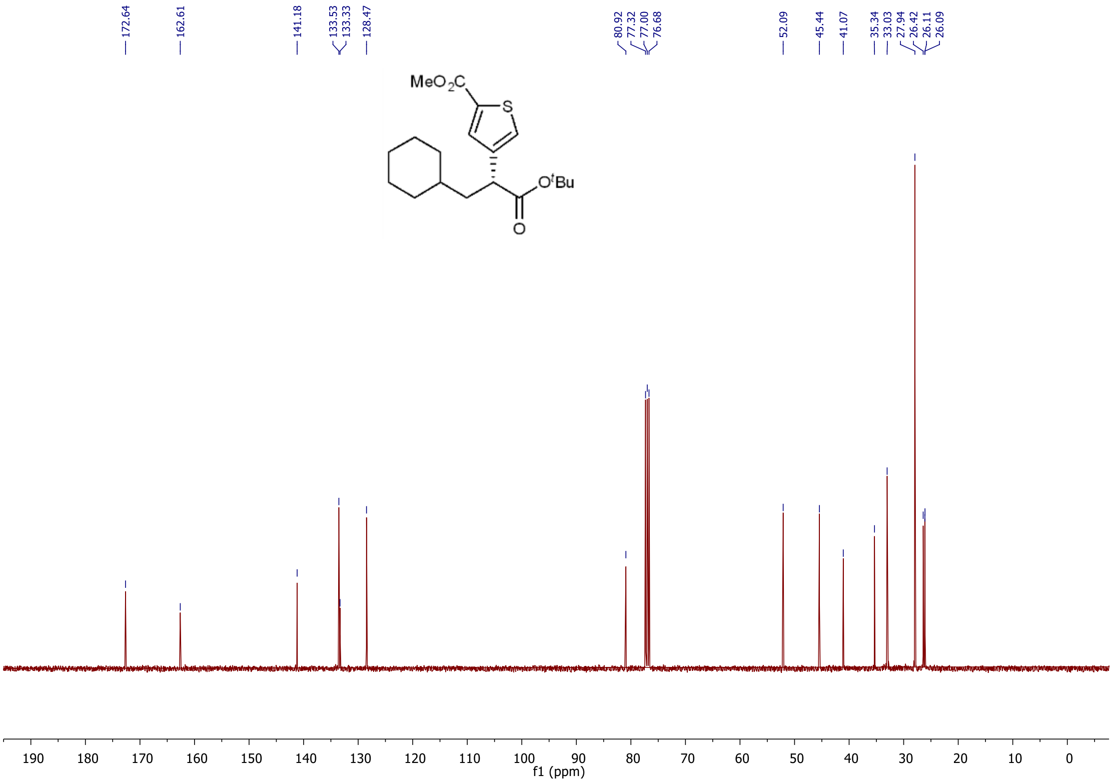

Supplementary Figure 76. ^{13}C NMR of compound **29**.

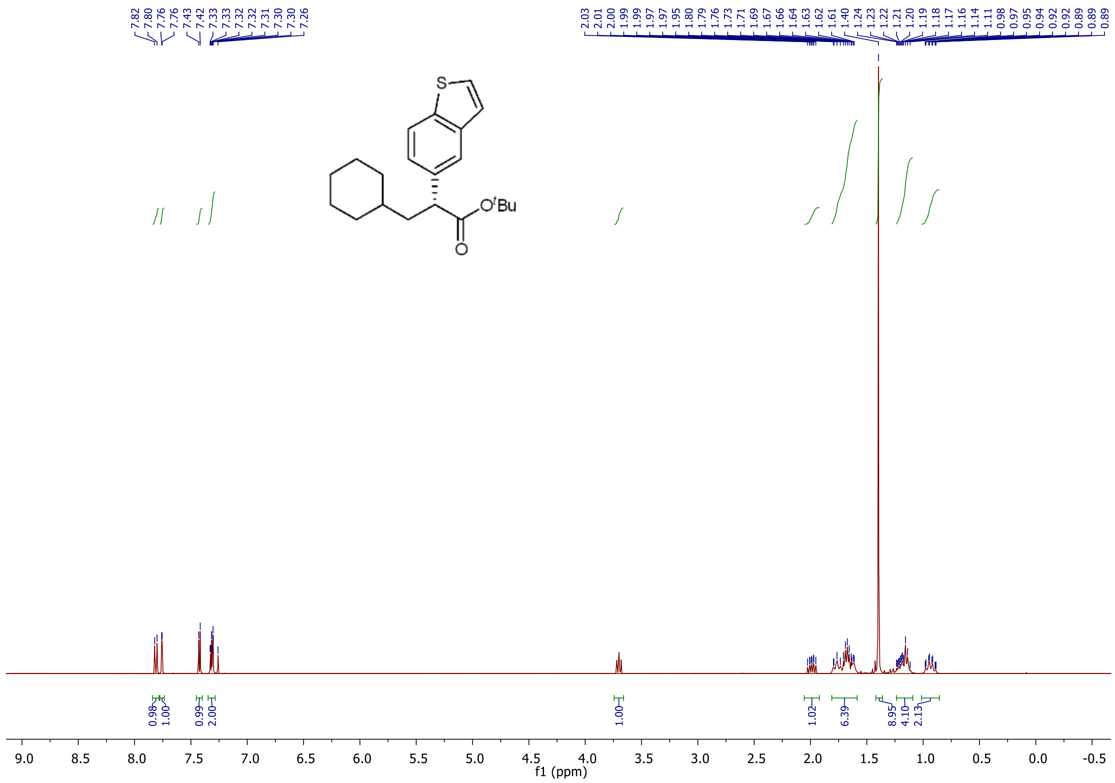

Supplementary Figure 77. ^{1}H NMR of compound **30**.

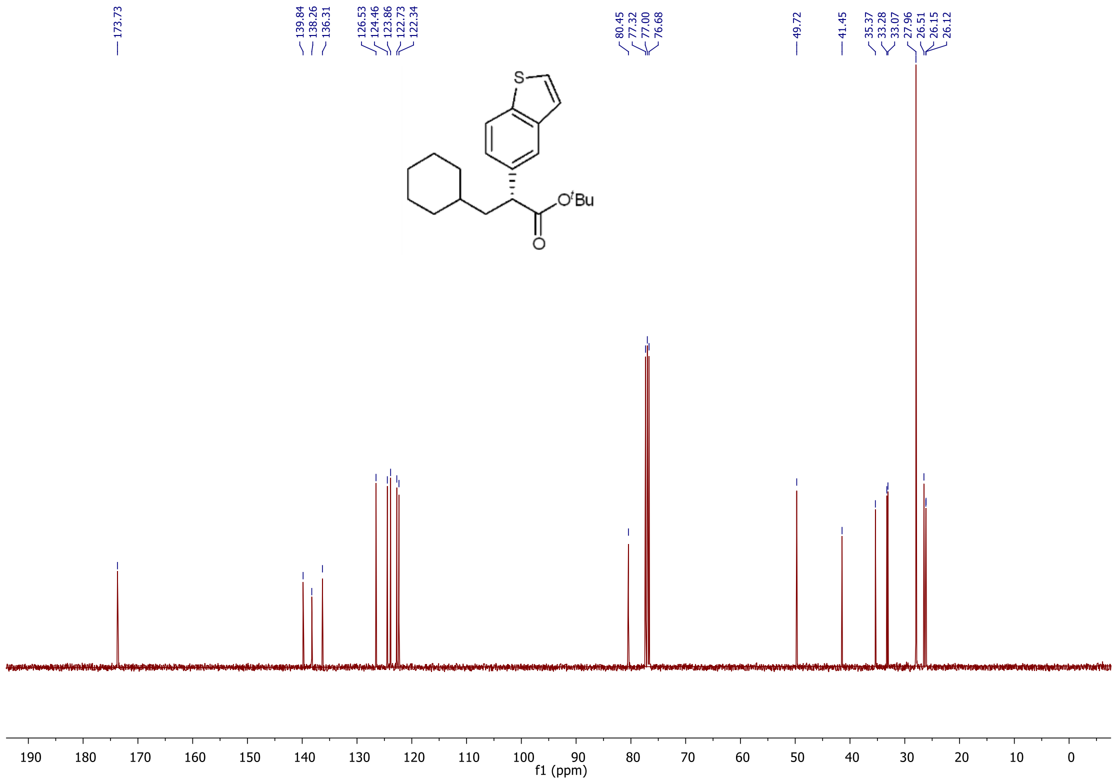

Supplementary Figure 78. ^{13}C NMR of compound **30**.

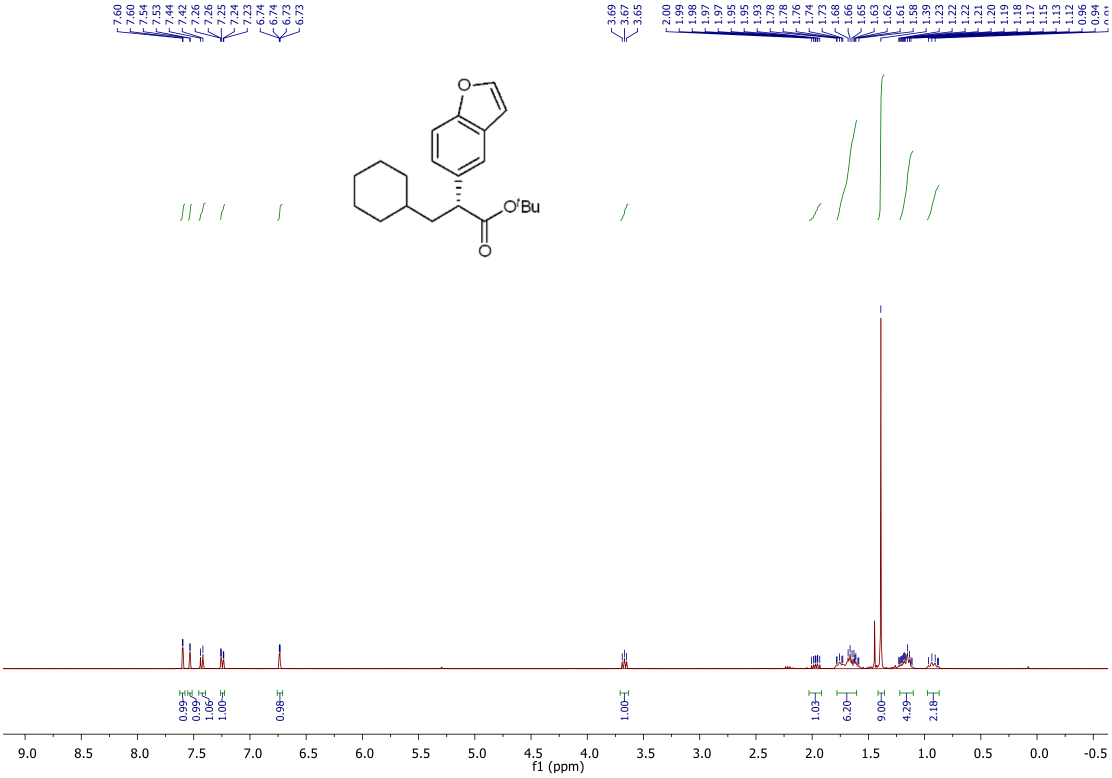

Supplementary Figure 79. ^{1}H NMR of compound **31**.

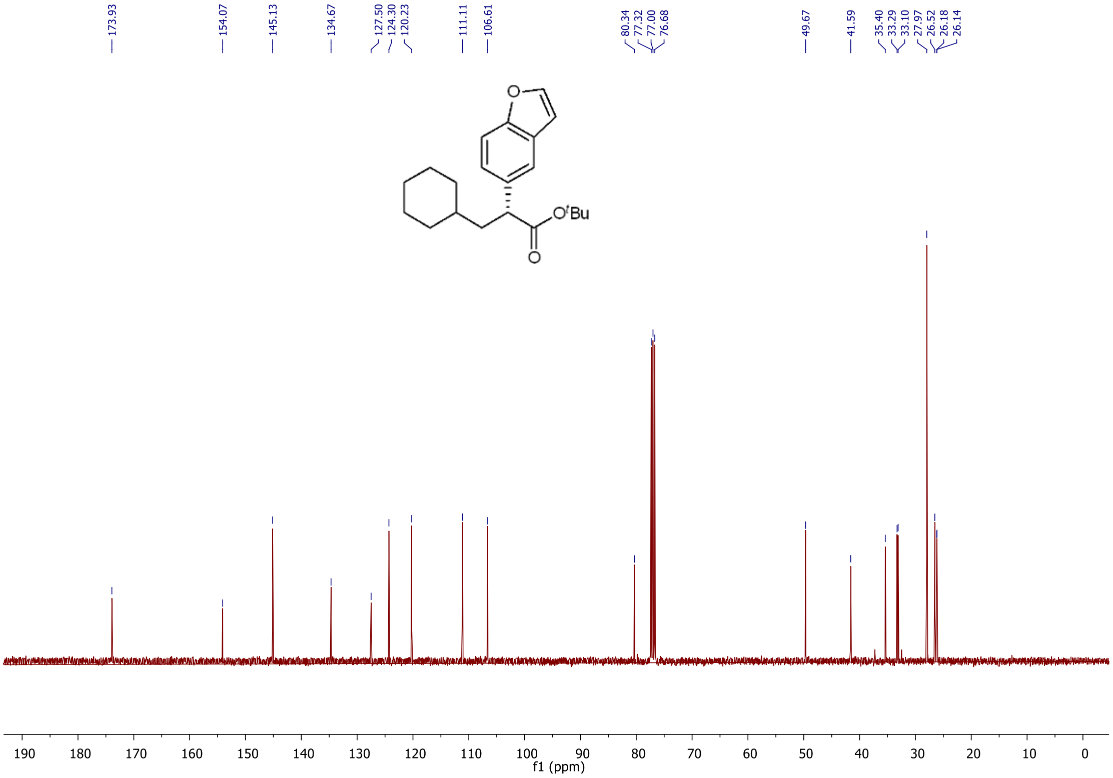

Supplementary Figure 80. ^{13}C NMR of compound **31**.

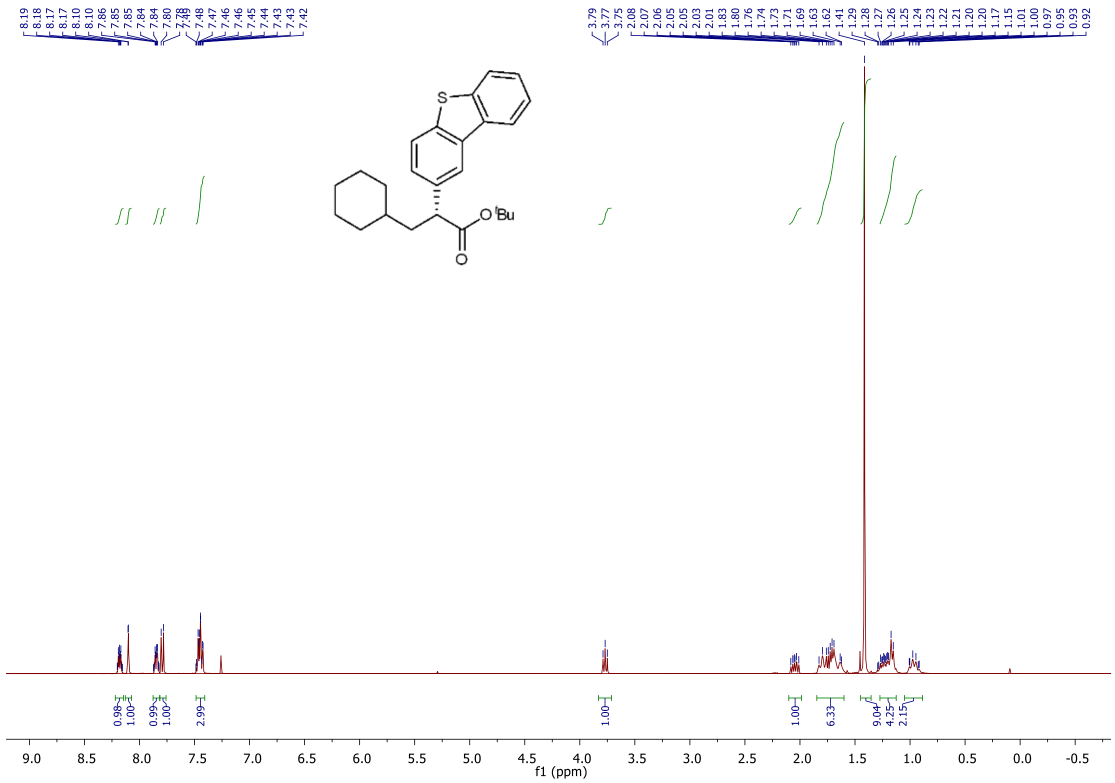

Supplementary Figure 81. ^{1}H NMR of compound **32**.

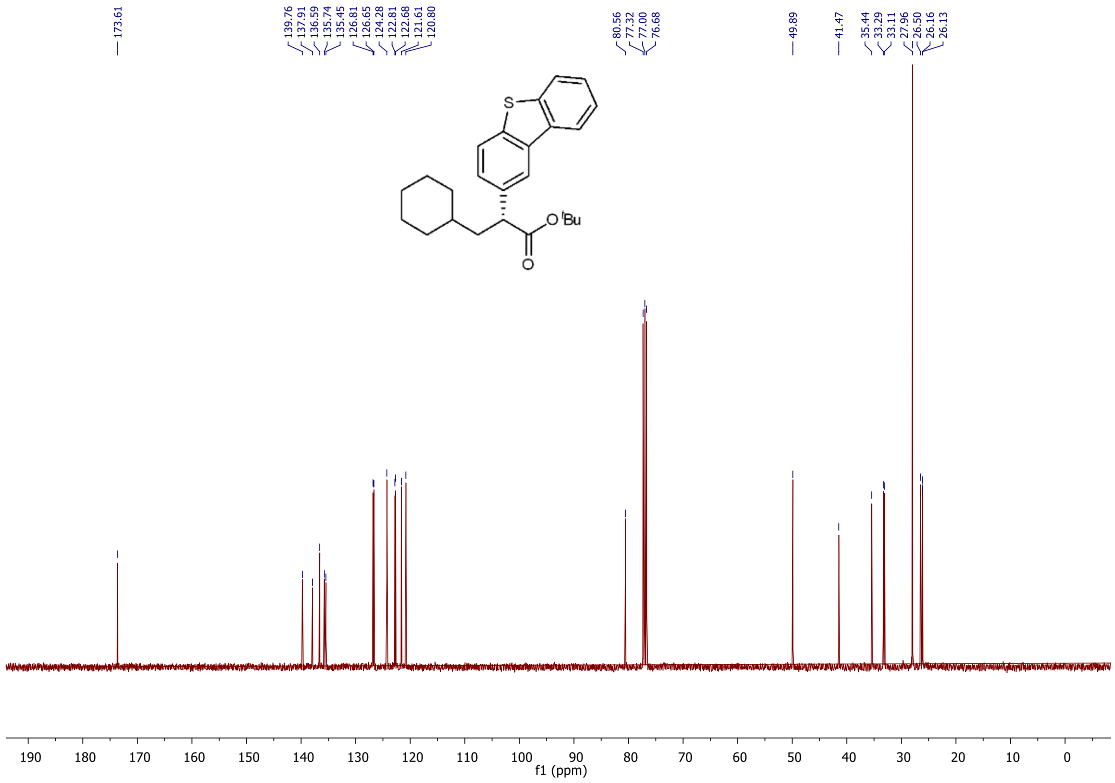

Supplementary Figure 82. ^{13}C NMR of compound **32**.

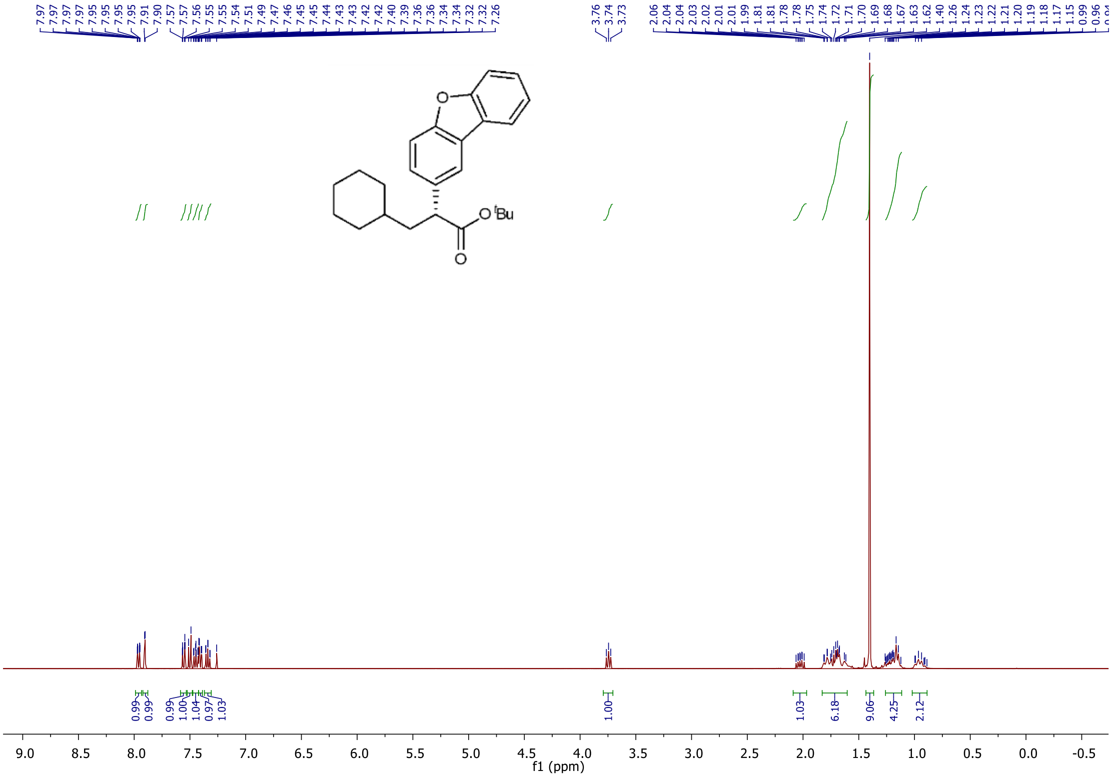

Supplementary Figure 83. ^{1}H NMR of compound **33**.

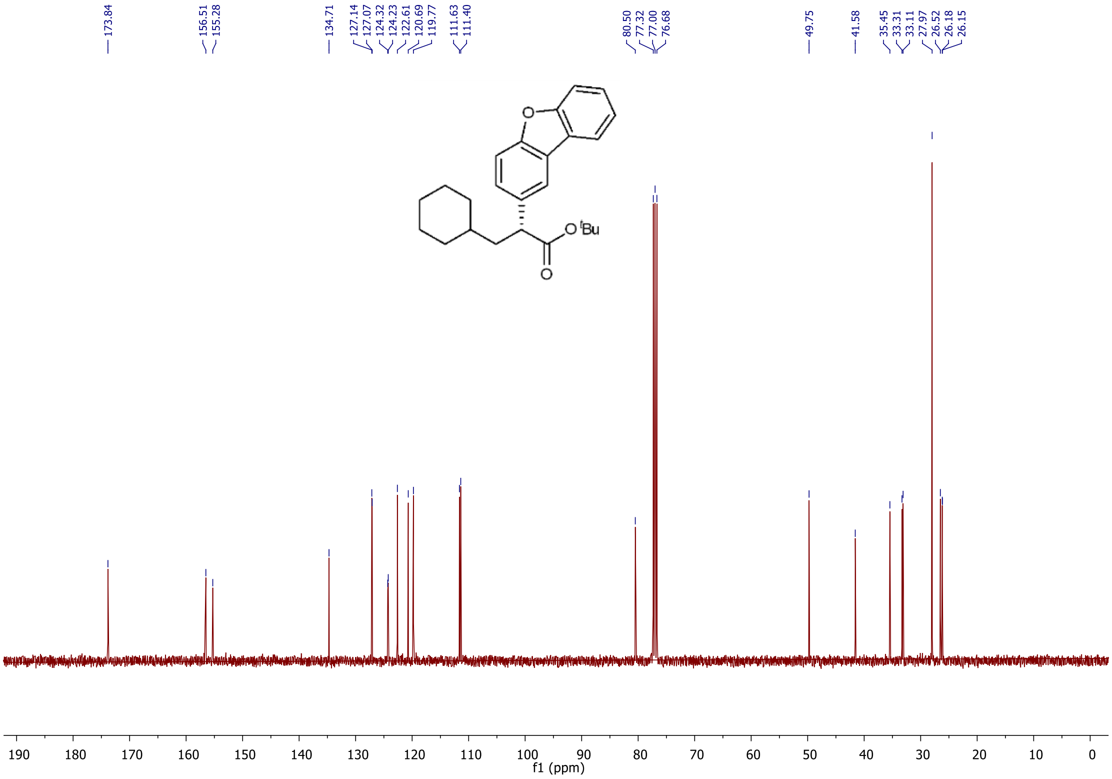

Supplementary Figure 84. ^{13}C NMR of compound **33**.

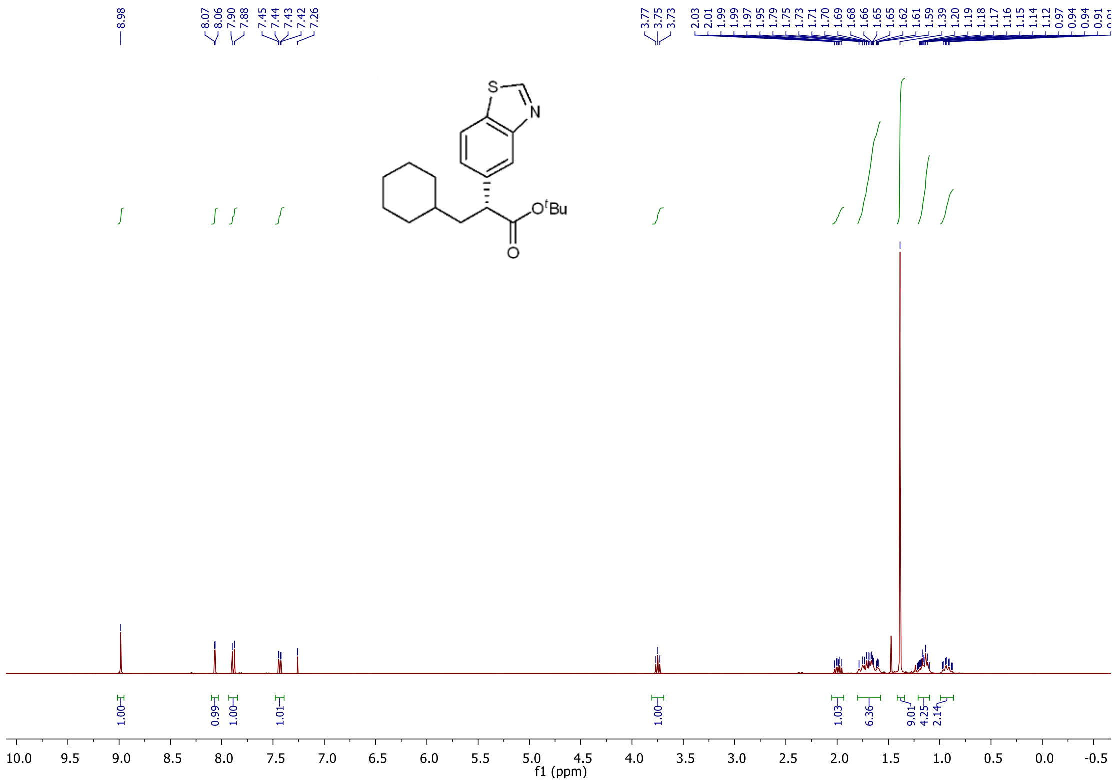

Supplementary Figure 85. ^{1}H NMR of compound **34**.

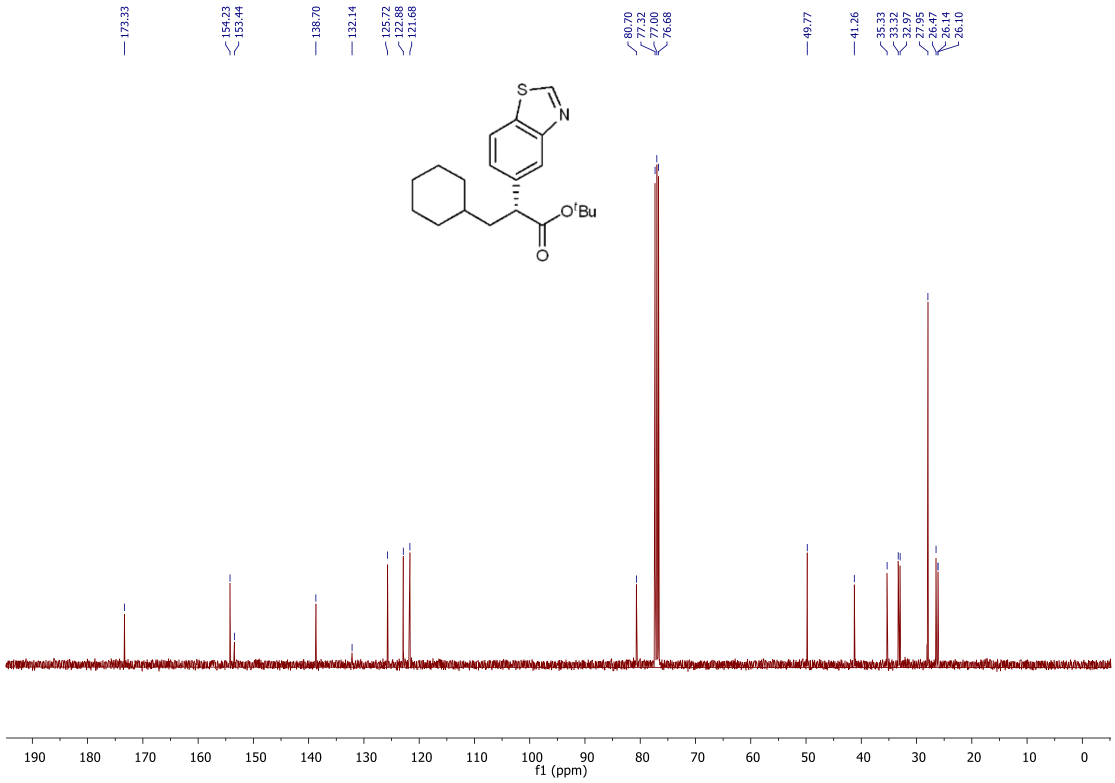

Supplementary Figure 86. ^{13}C NMR of compound **34**.

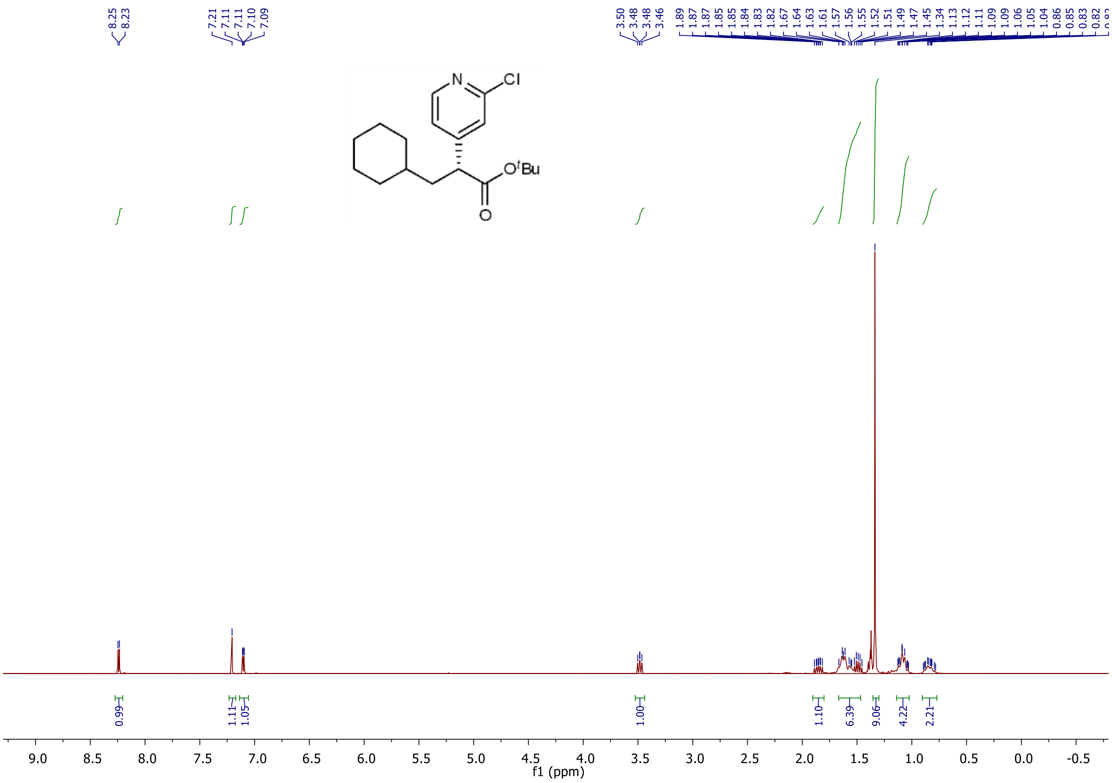

Supplementary Figure 87. ^{1}H NMR of compound **35**.

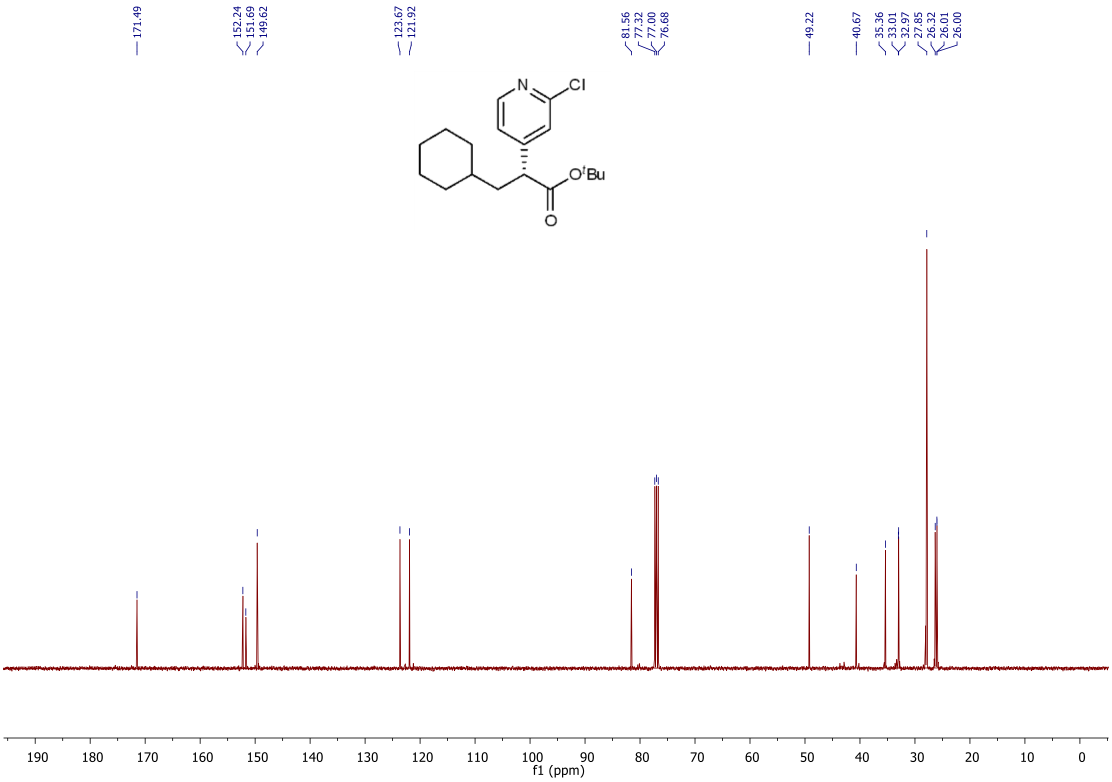

Supplementary Figure 88. ^{13}C NMR of compound **35**.

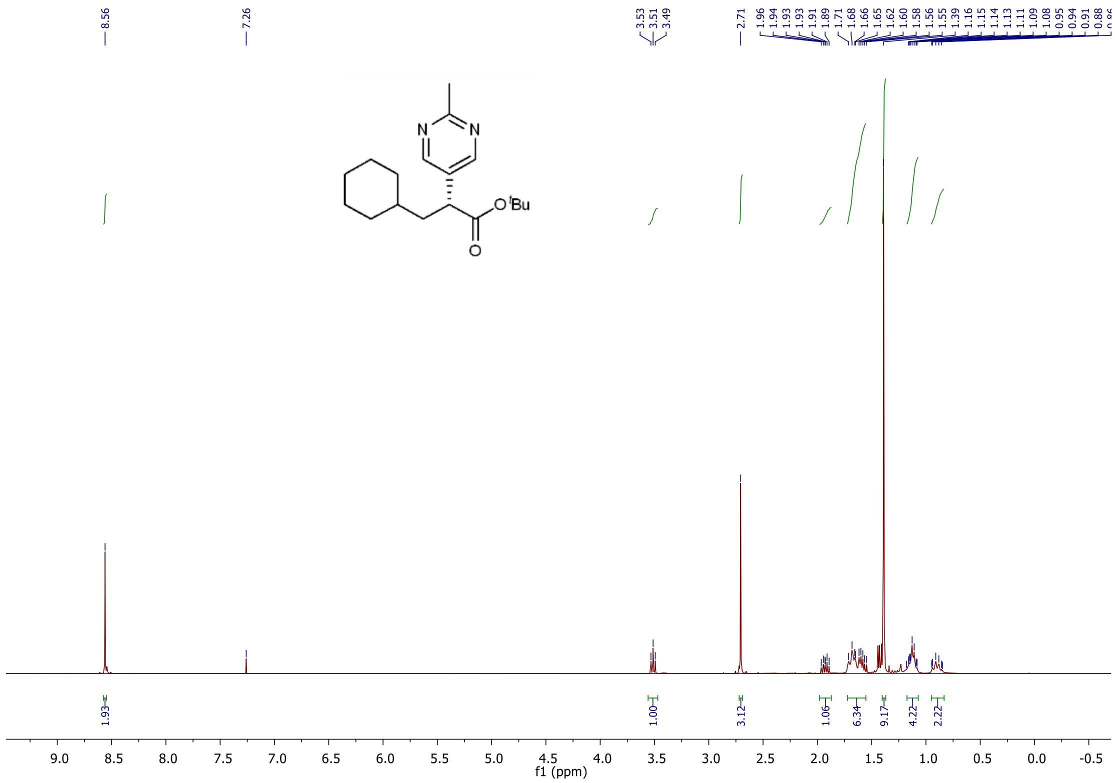

Supplementary Figure 89. ^{1}H NMR of compound **36**.

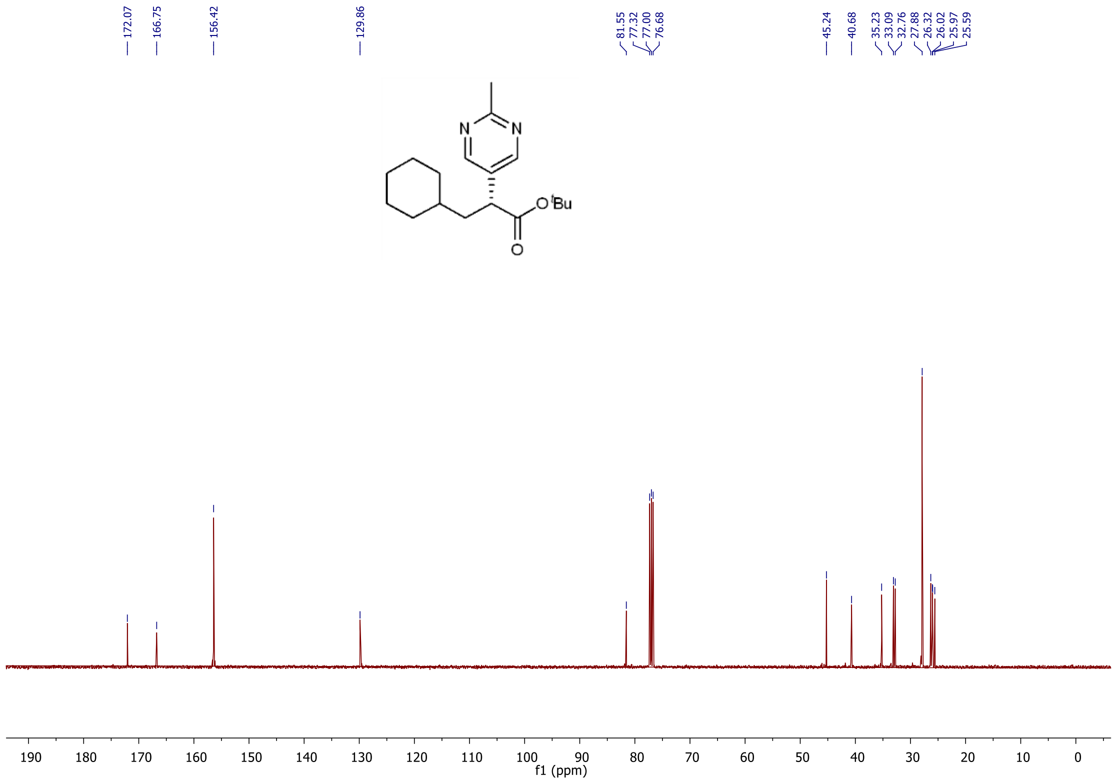

Supplementary Figure 90. ^{13}C NMR of compound **36**.

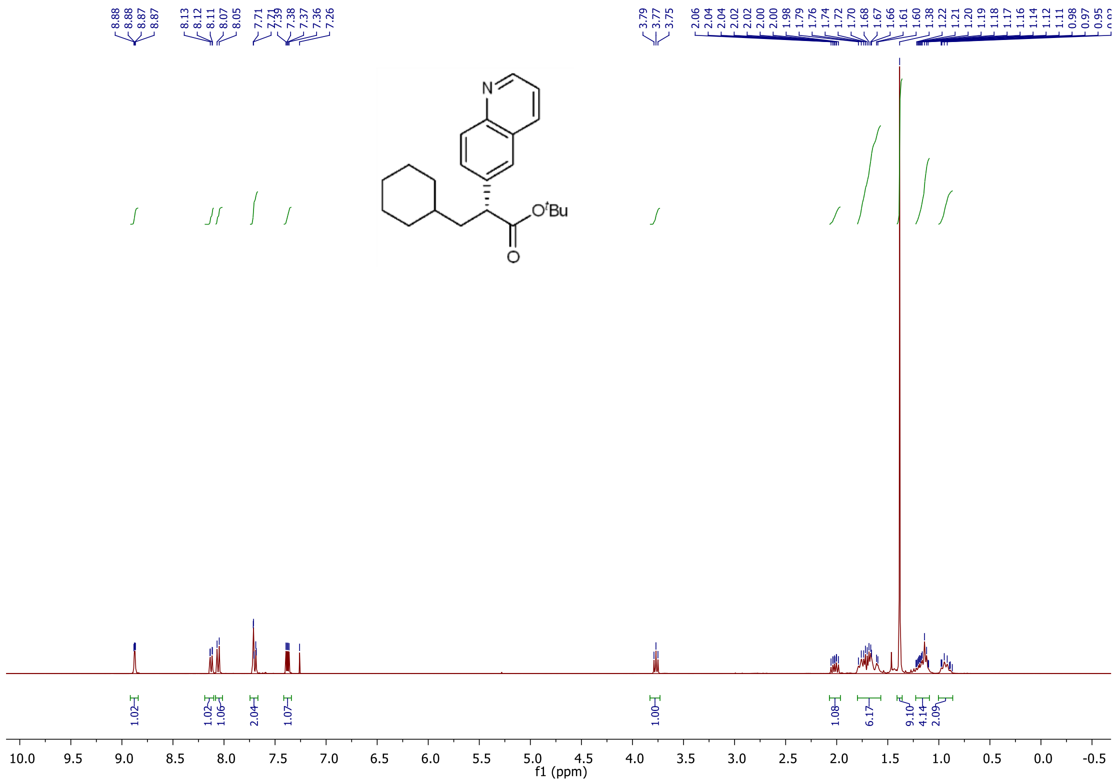

Supplementary Figure 91. ^{1}H NMR of compound **37**.

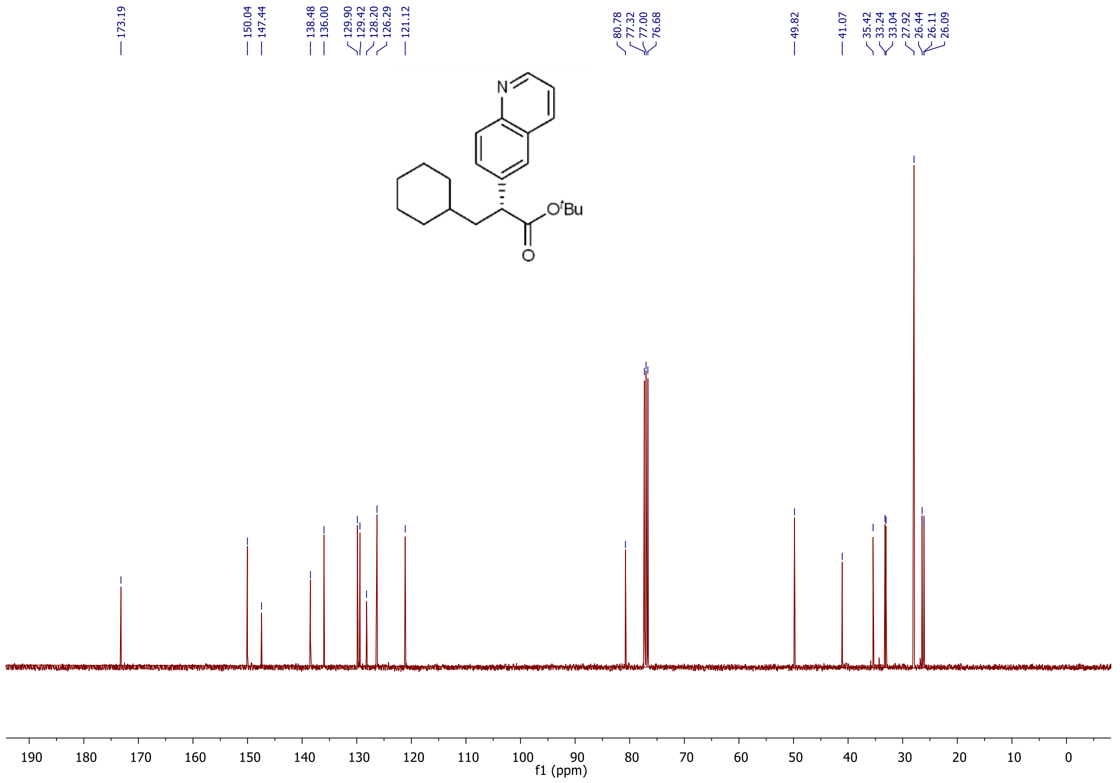

Supplementary Figure 92. ^{13}C NMR of compound **37**.

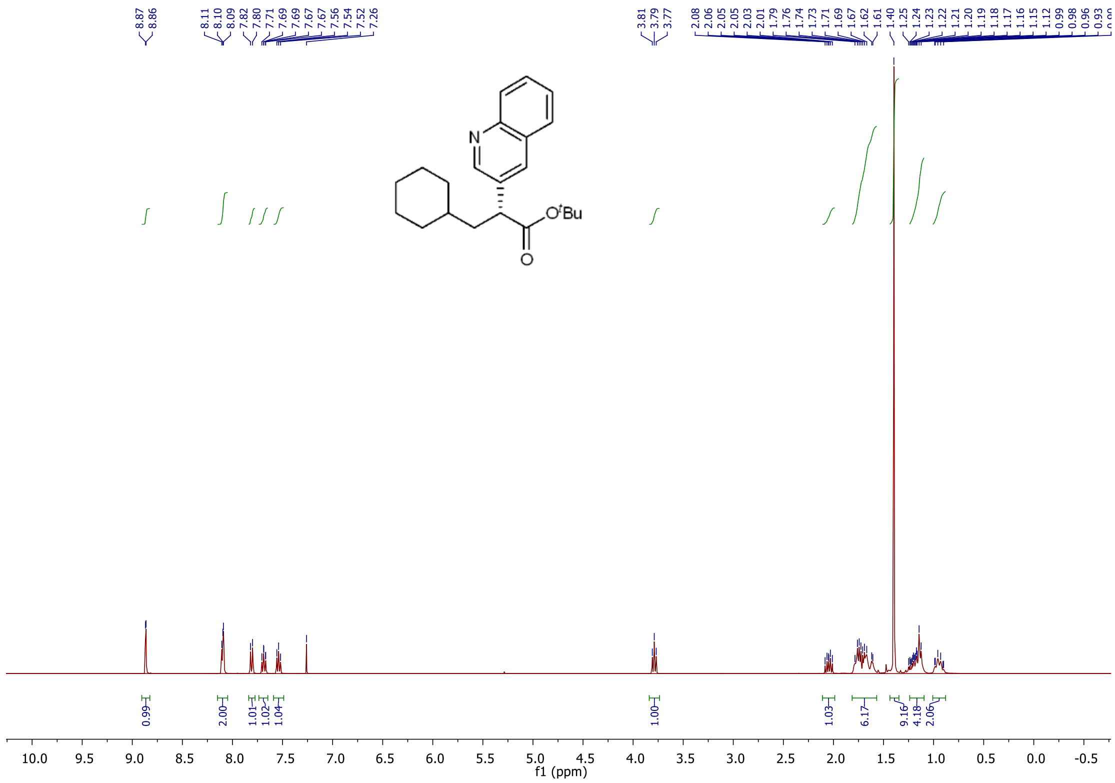

Supplementary Figure 93. ^{1}H NMR of compound **38**.

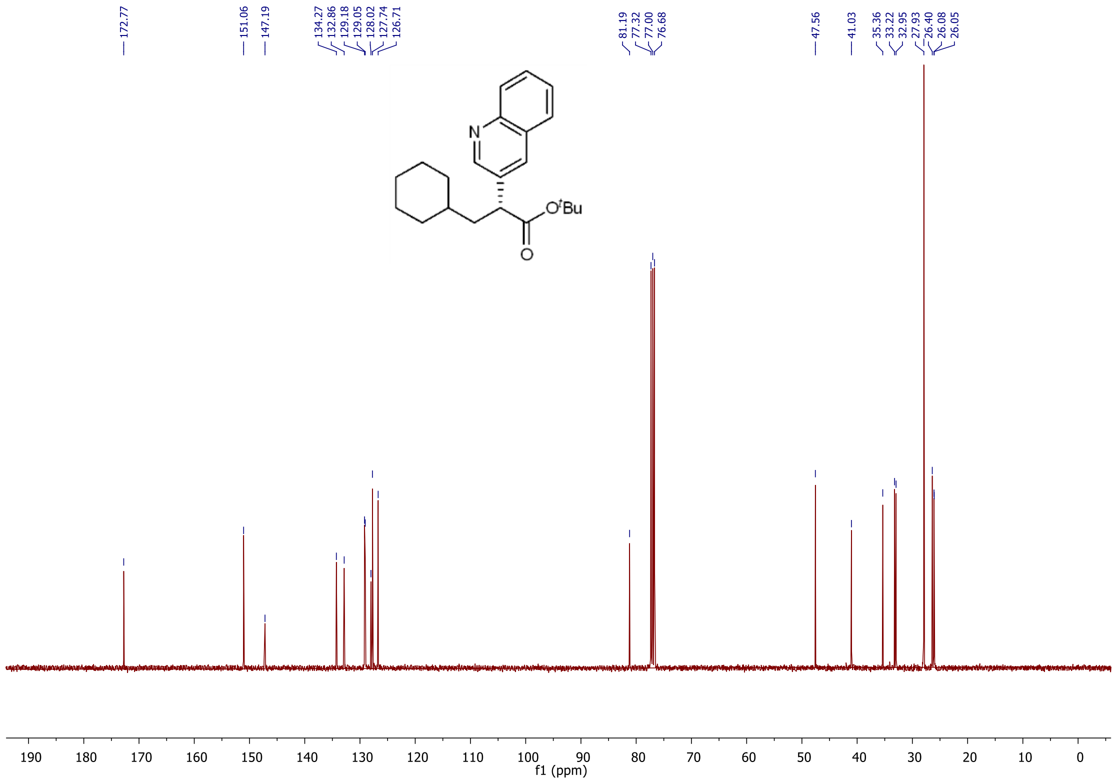

Supplementary Figure 94. ^{13}C NMR of compound **38**.

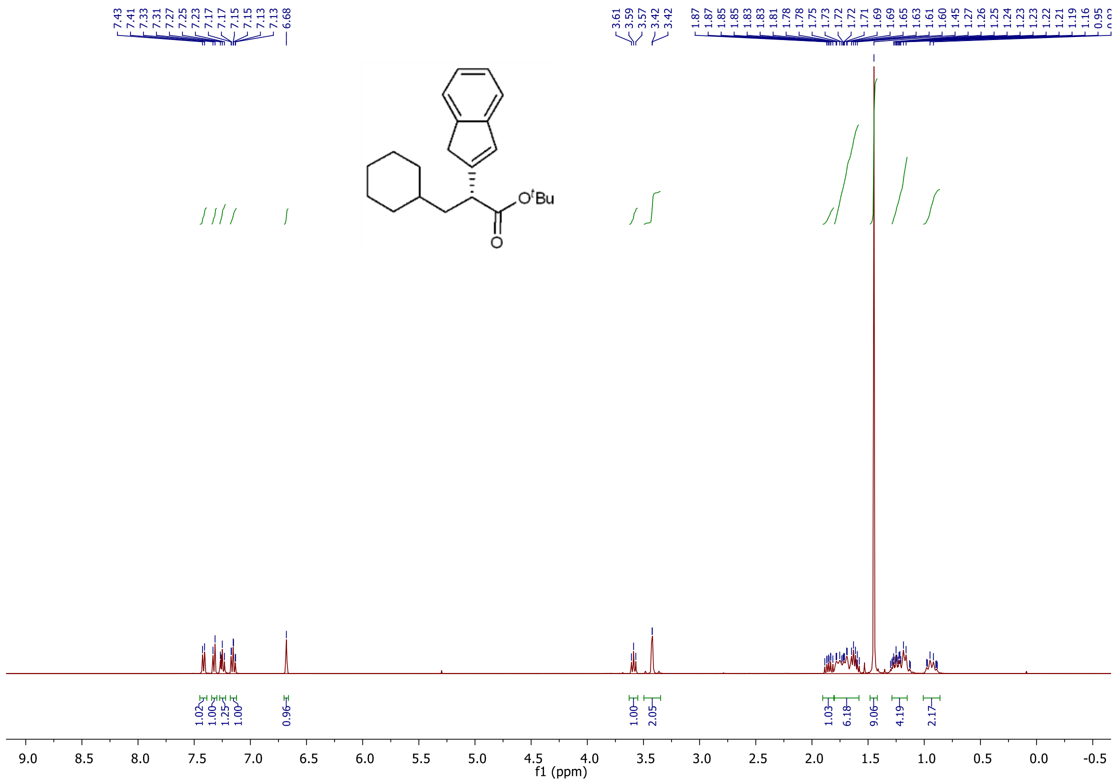

Supplementary Figure 95. ^{1}H NMR of compound **39**.

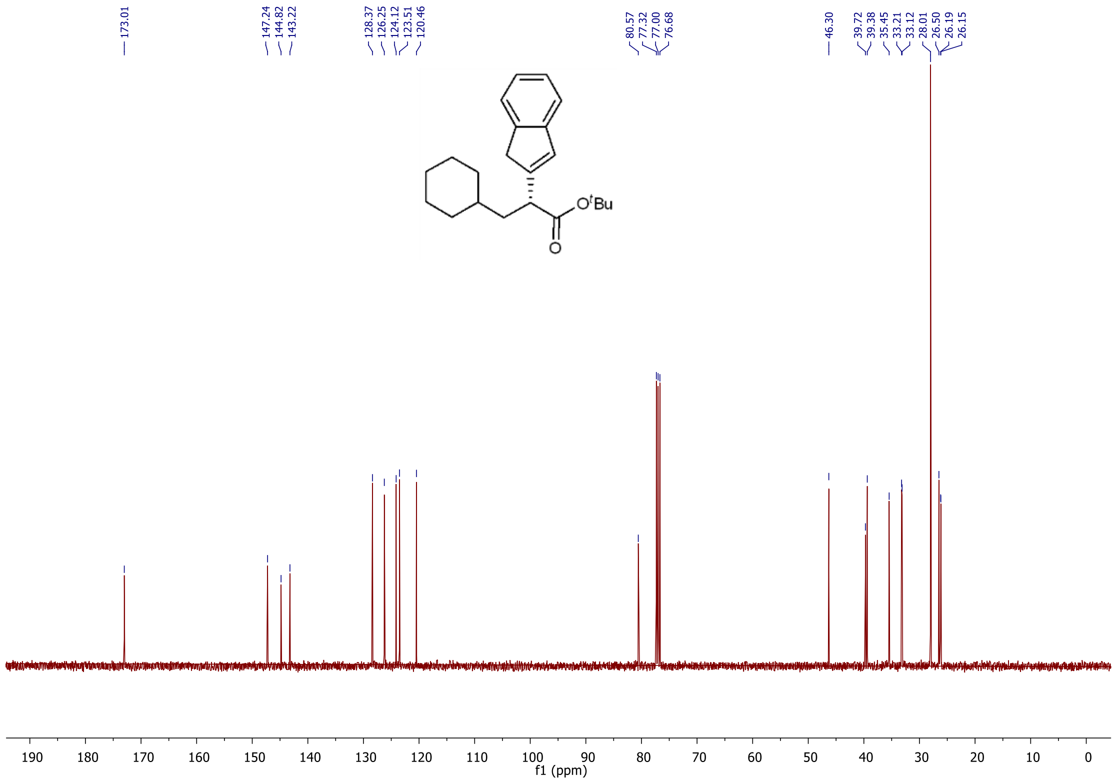

Supplementary Figure 96. ^{13}C NMR of compound **39**.

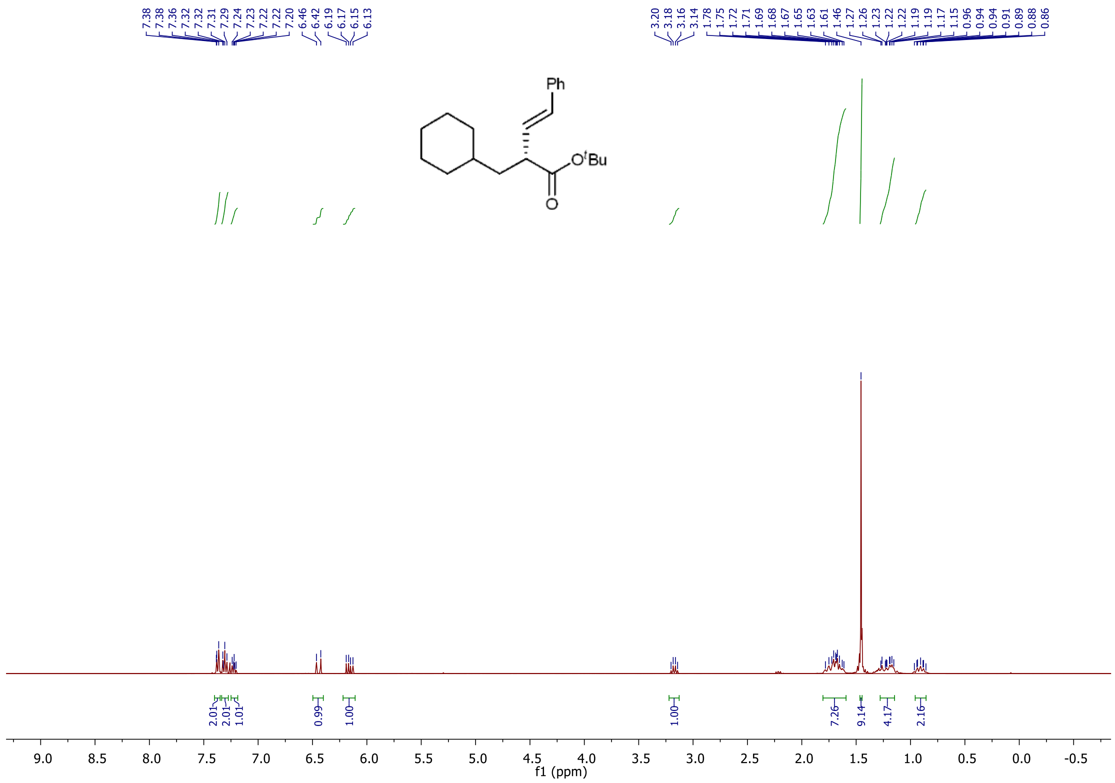

Supplementary Figure 97. ^{1}H NMR of compound **40**.

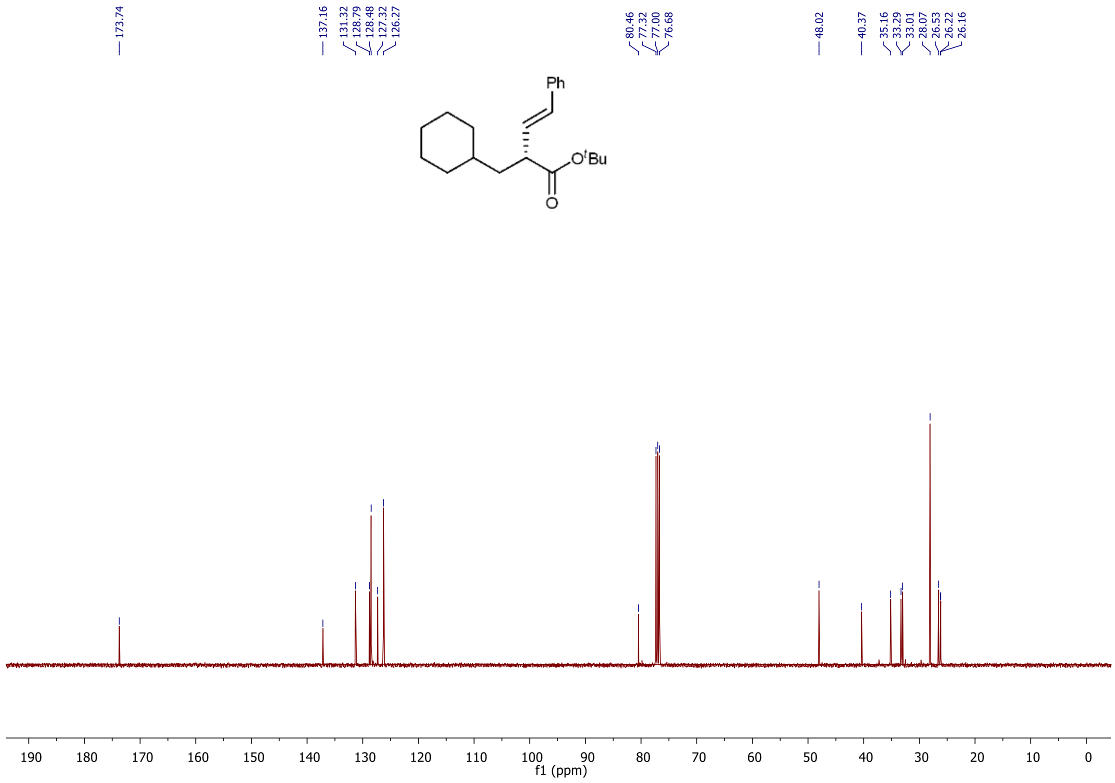

Supplementary Figure 98. ^{13}C NMR of compound **40**.

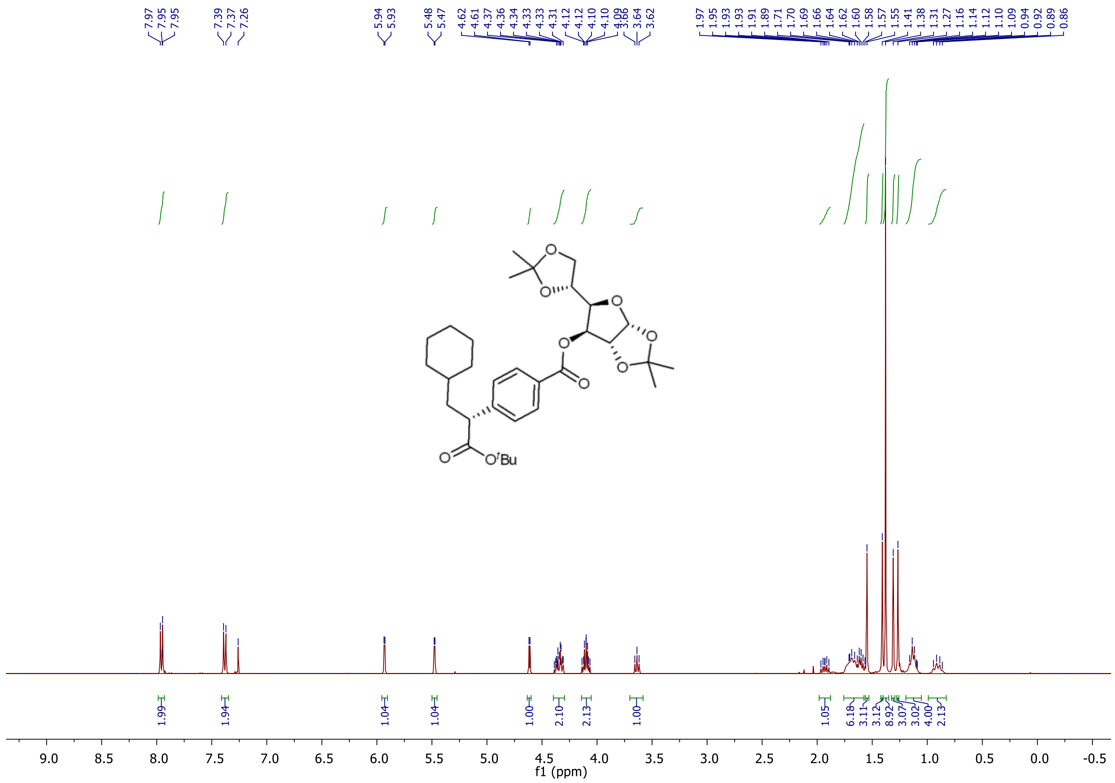

Supplementary Figure 99. ^{1}H NMR of compound **41**.

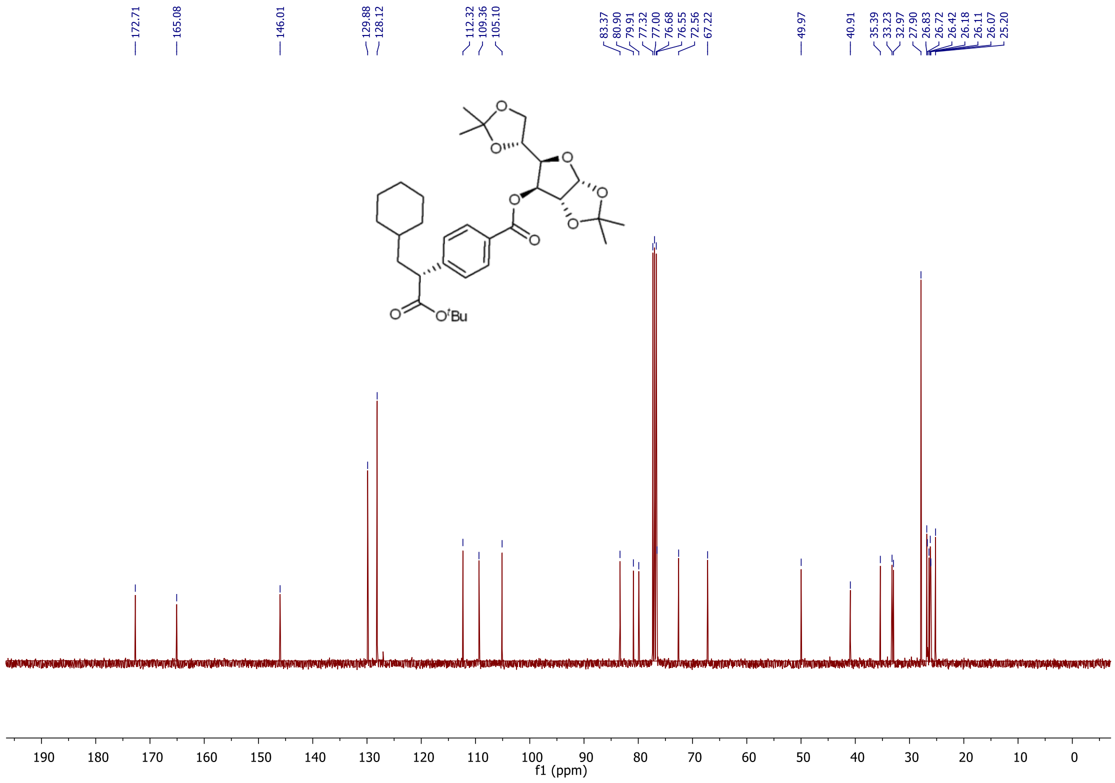

Supplementary Figure 100. ^{13}C NMR of compound **41**.

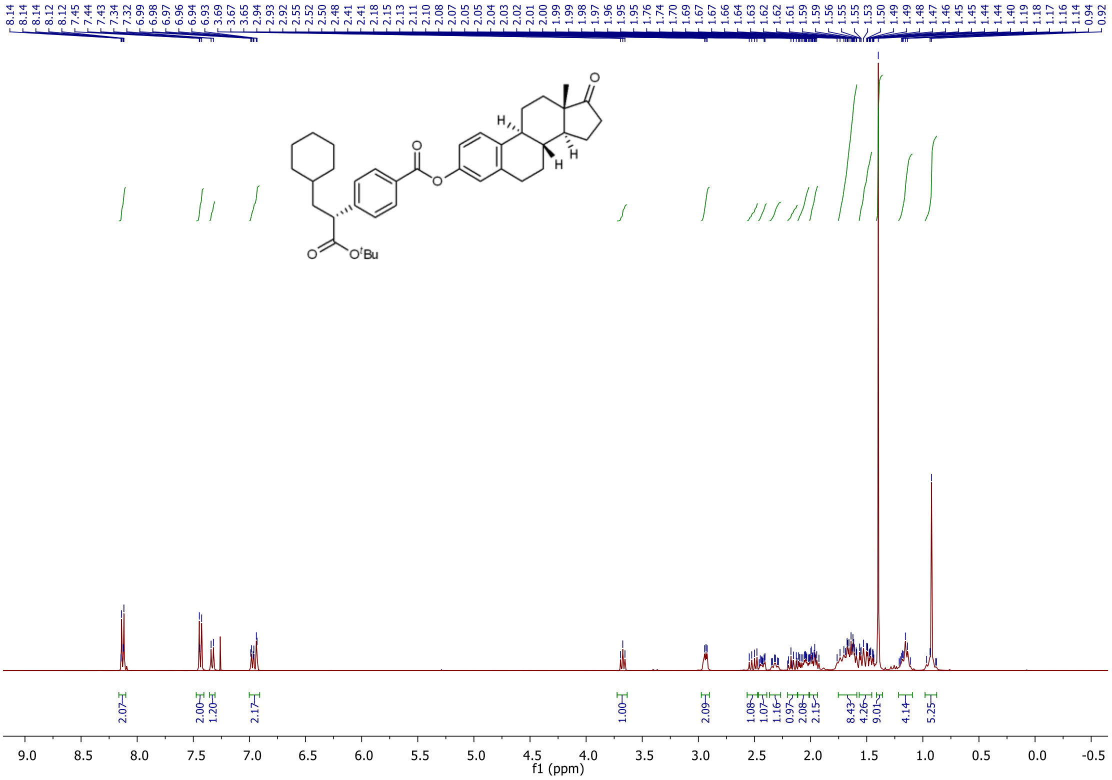

Supplementary Figure 101. ^{1}H NMR of compound **42**.

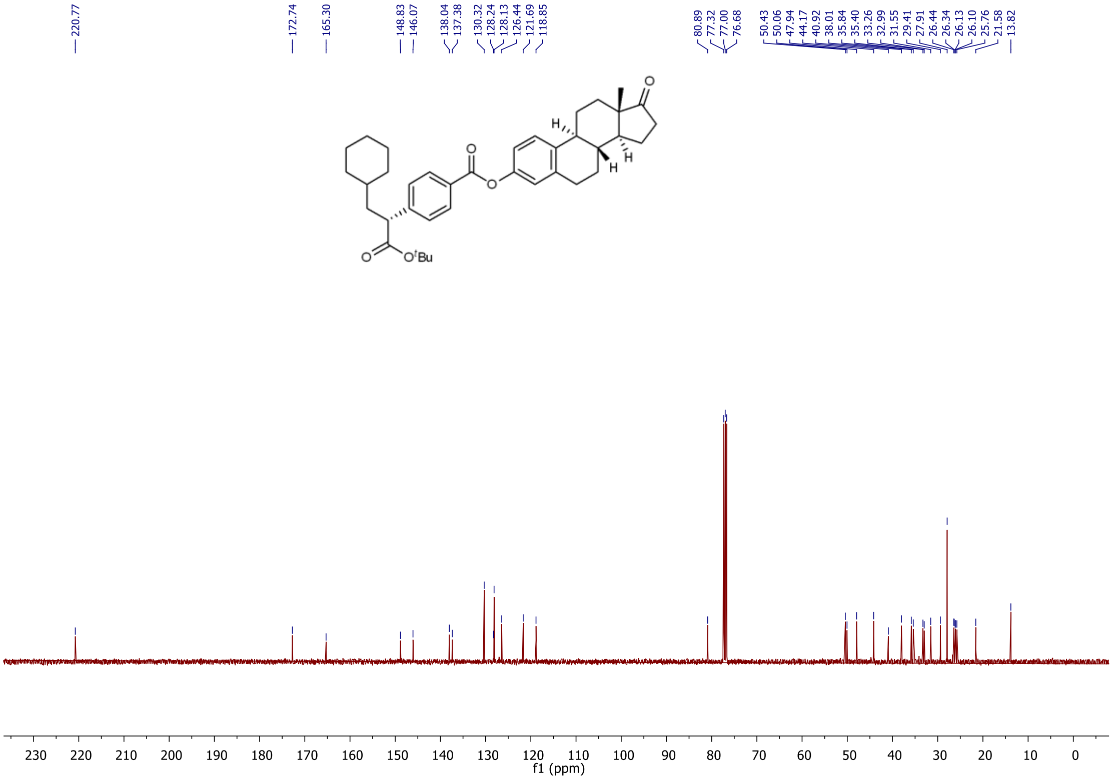

Supplementary Figure 102. ^{13}C NMR of compound **42**.

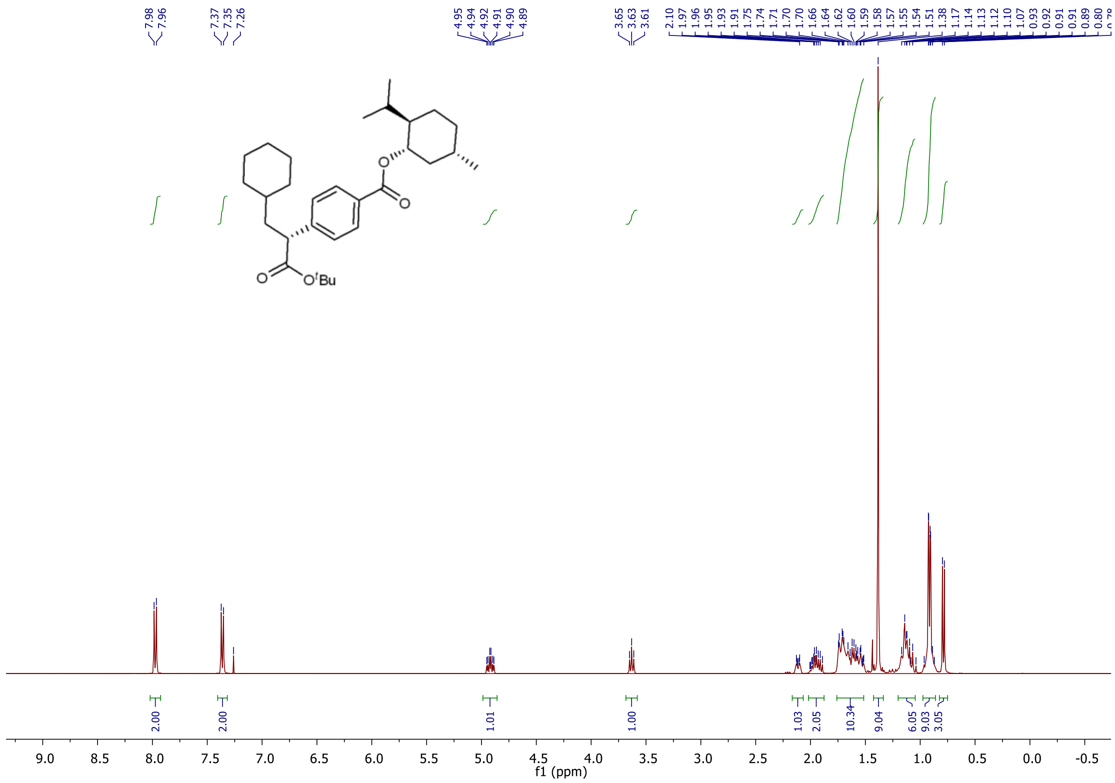

Supplementary Figure 103. ^{1}H NMR of compound **43**.

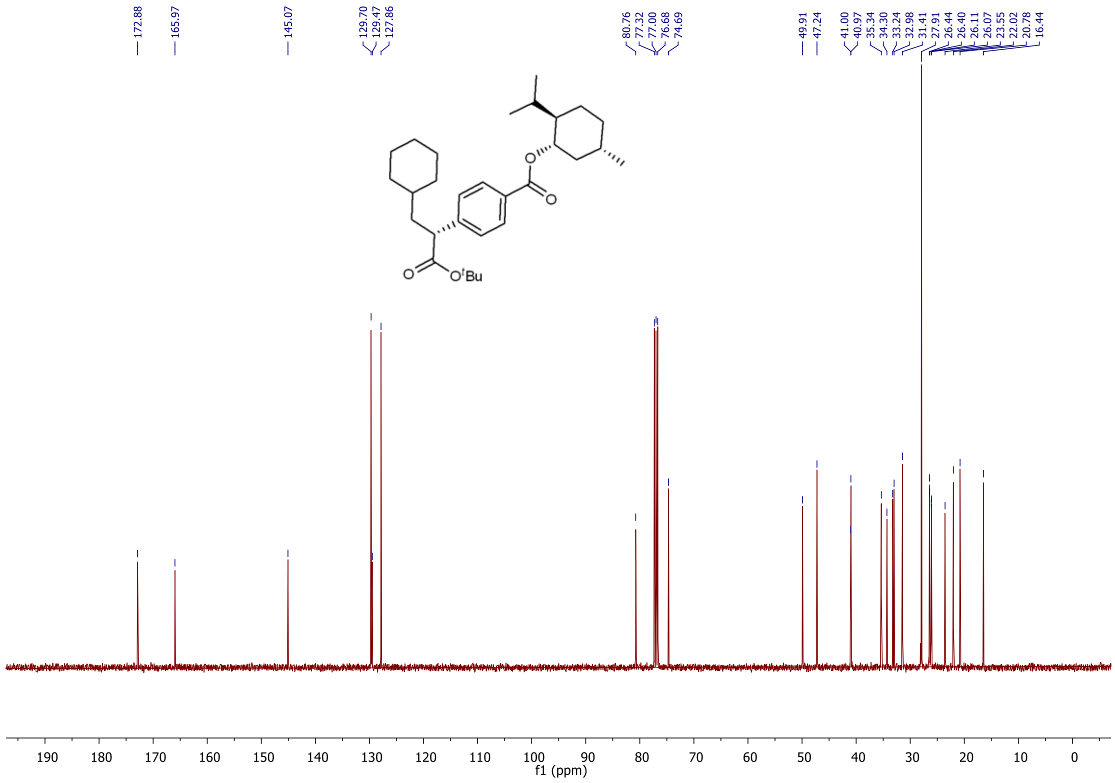

Supplementary Figure 104. ^{13}C NMR of compound **43**.

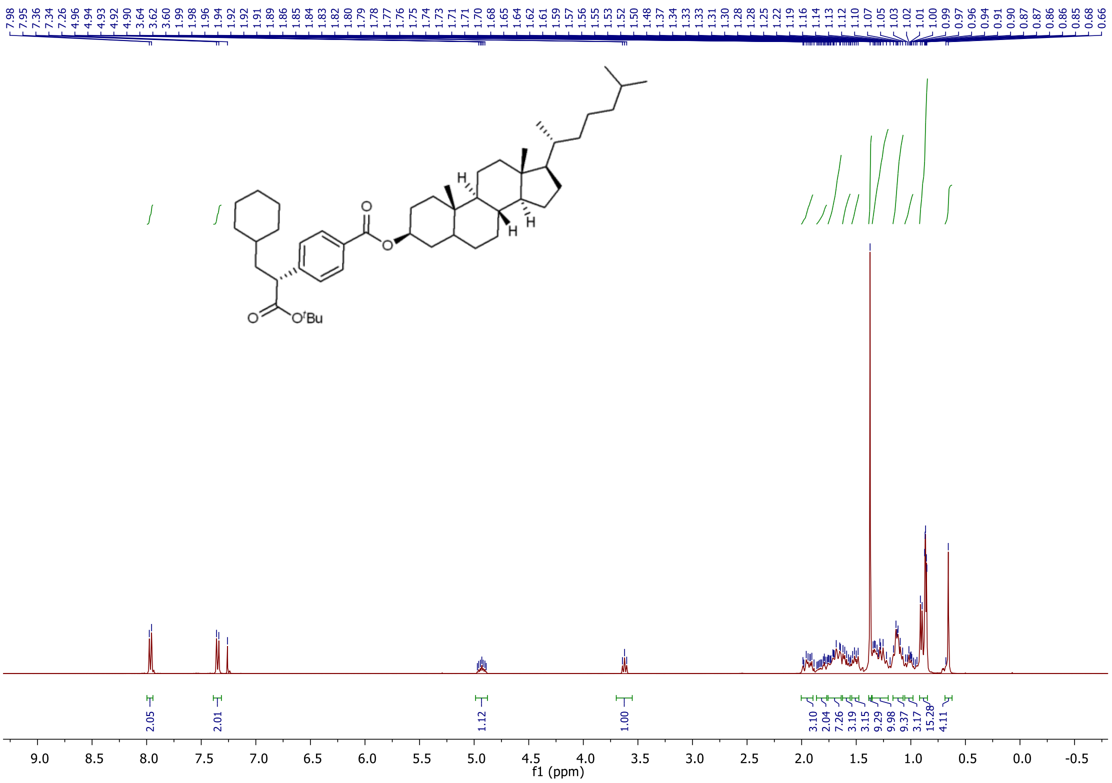

Supplementary Figure 105. ^{1}H NMR of compound **44**.

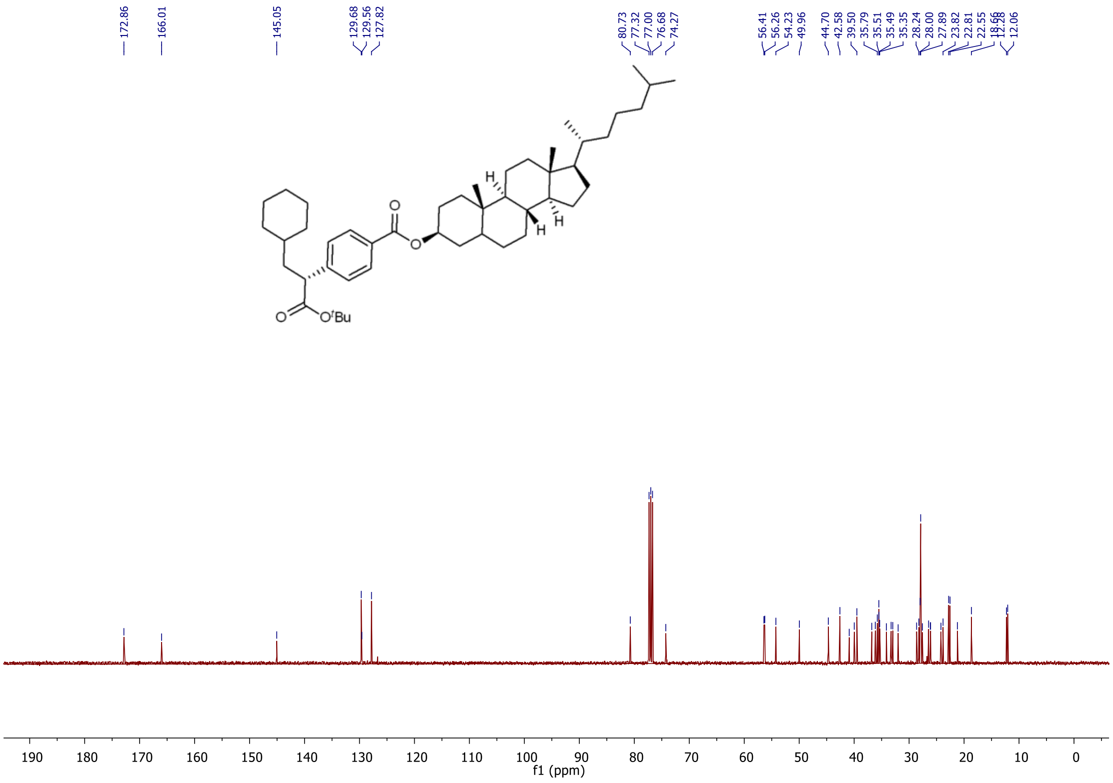

Supplementary Figure 106. ^{13}C NMR of compound **44**.

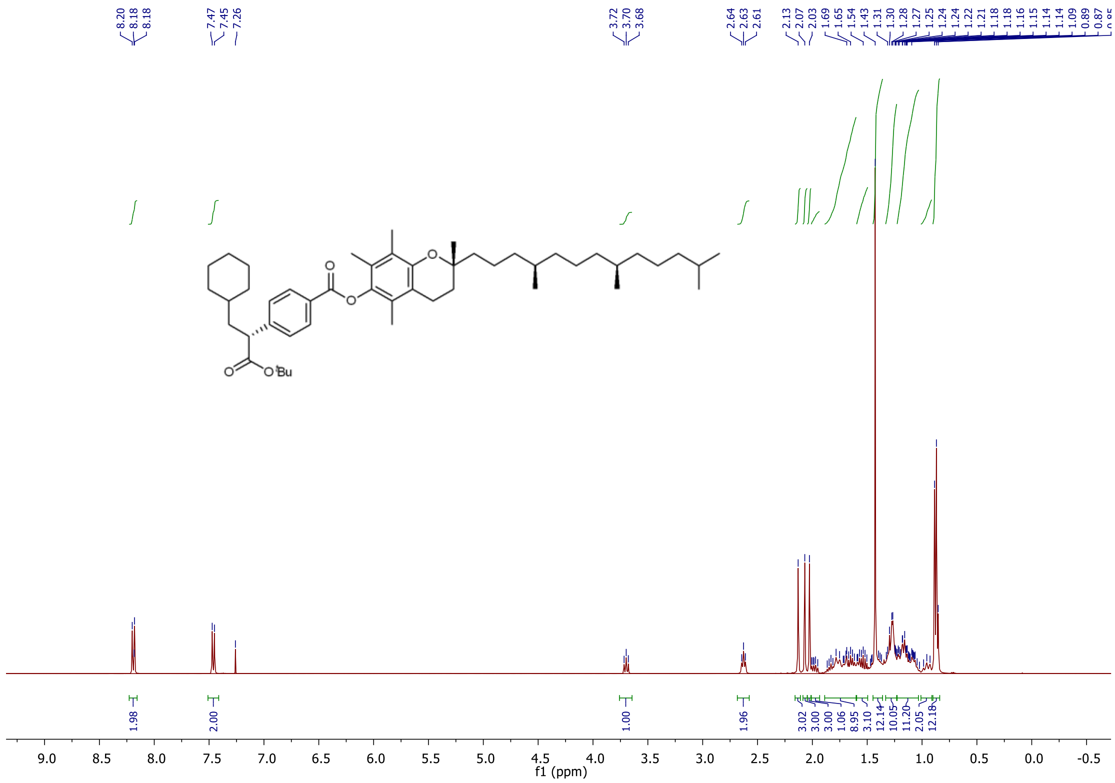

Supplementary Figure 107. ^{1}H NMR of compound **45**.

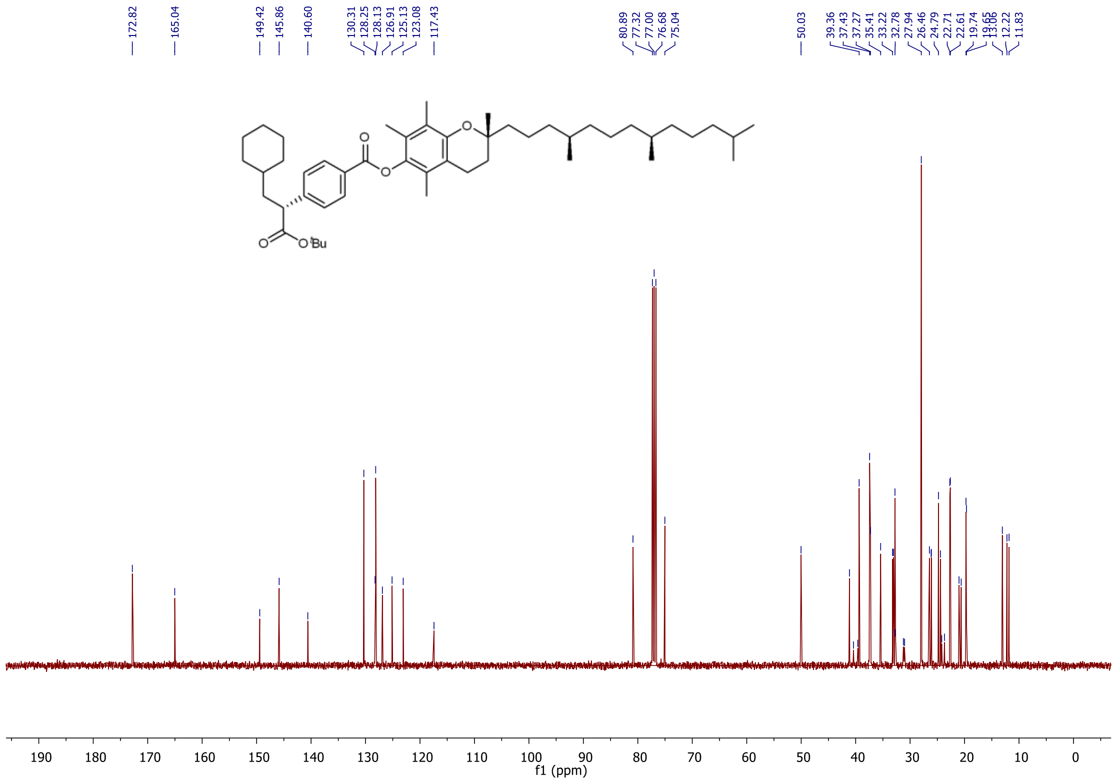

Supplementary Figure 108. ^{13}C NMR of compound **45**.

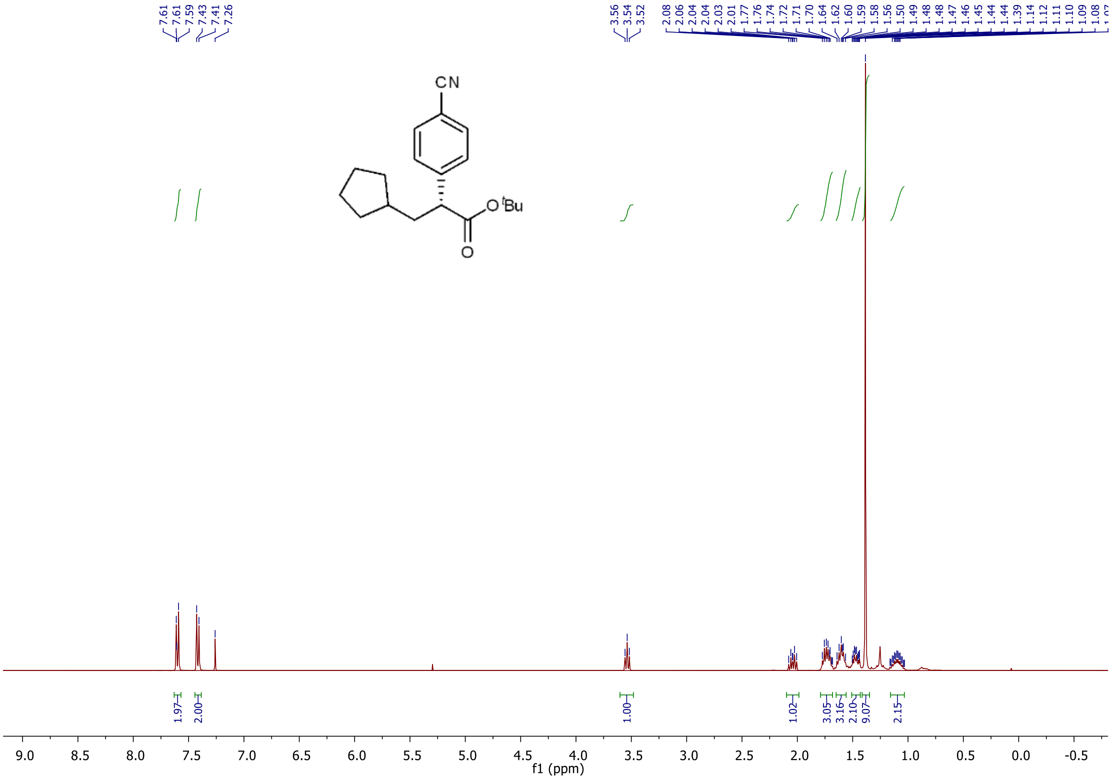

Supplementary Figure 109. ^{1}H NMR of compound **46**.

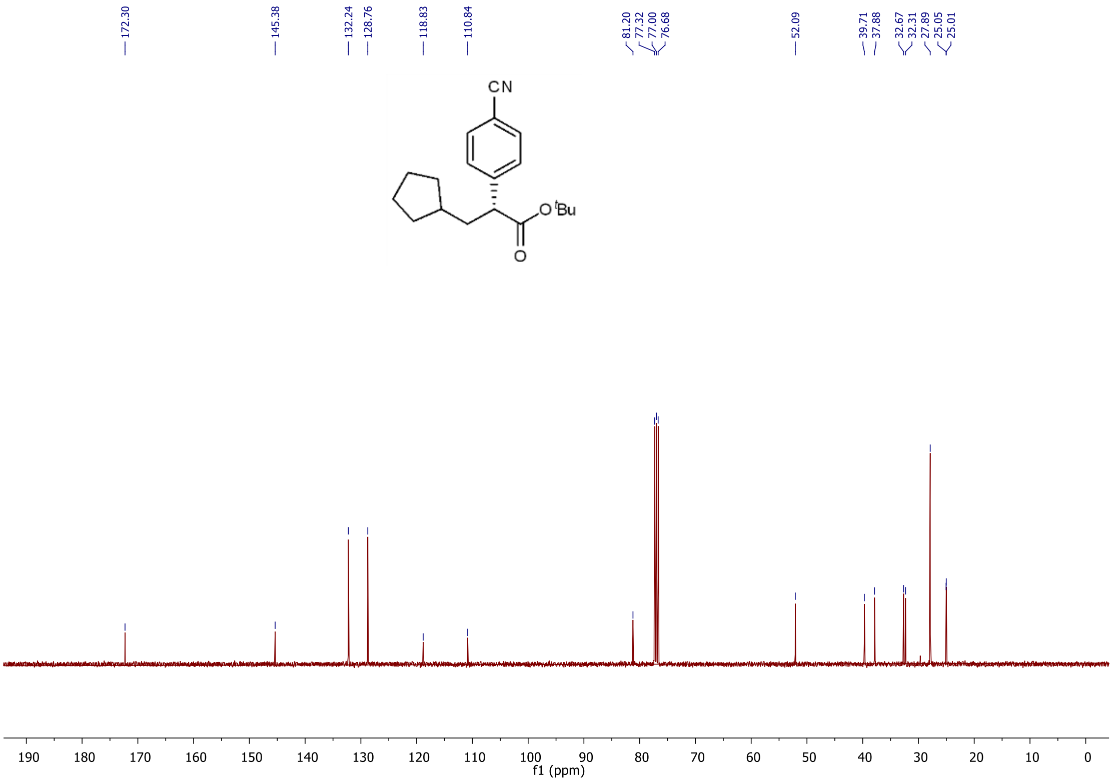

Supplementary Figure 110. ^{13}C NMR of compound **46**.

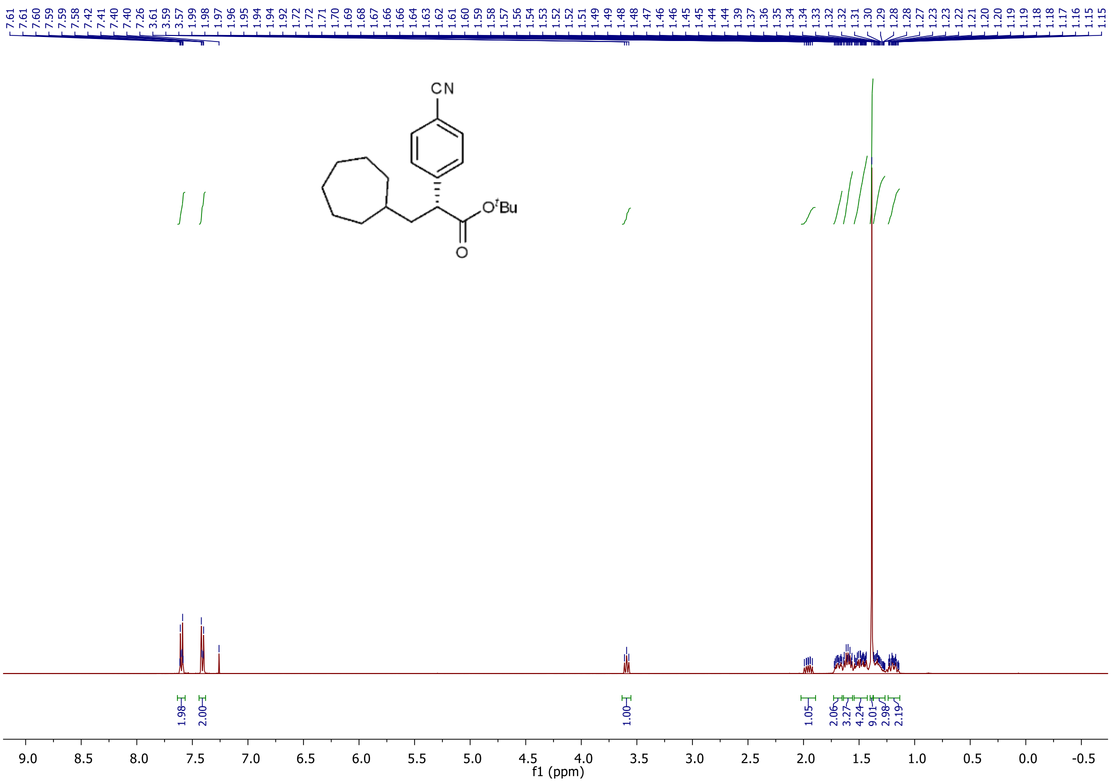

Supplementary Figure 111. ^{1}H NMR of compound **47**.

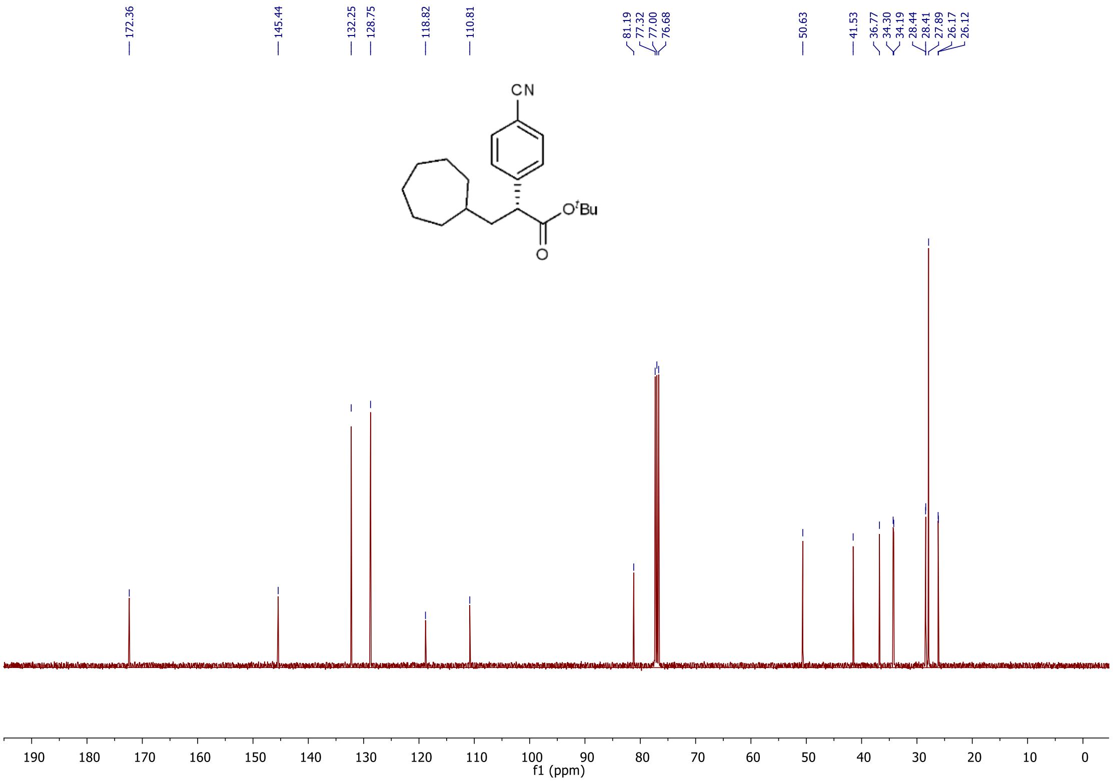

Supplementary Figure 112. ^{13}C NMR of compound **47**.

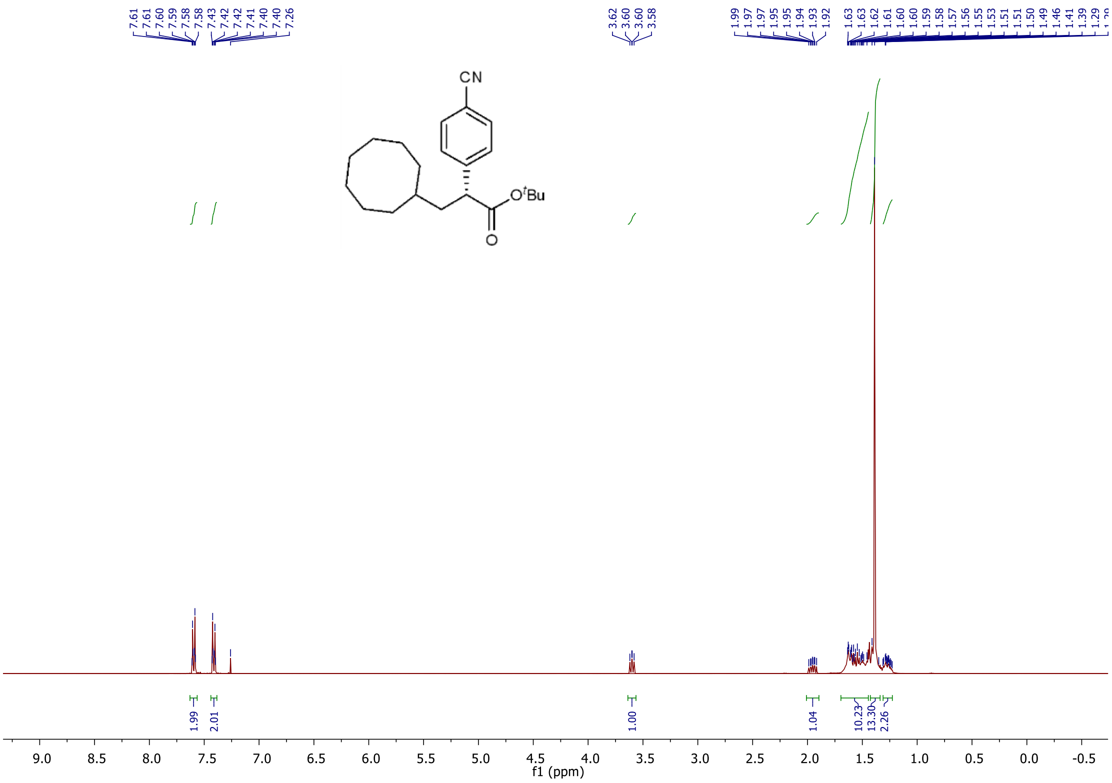

Supplementary Figure 113. ^{1}H NMR of compound **48**.

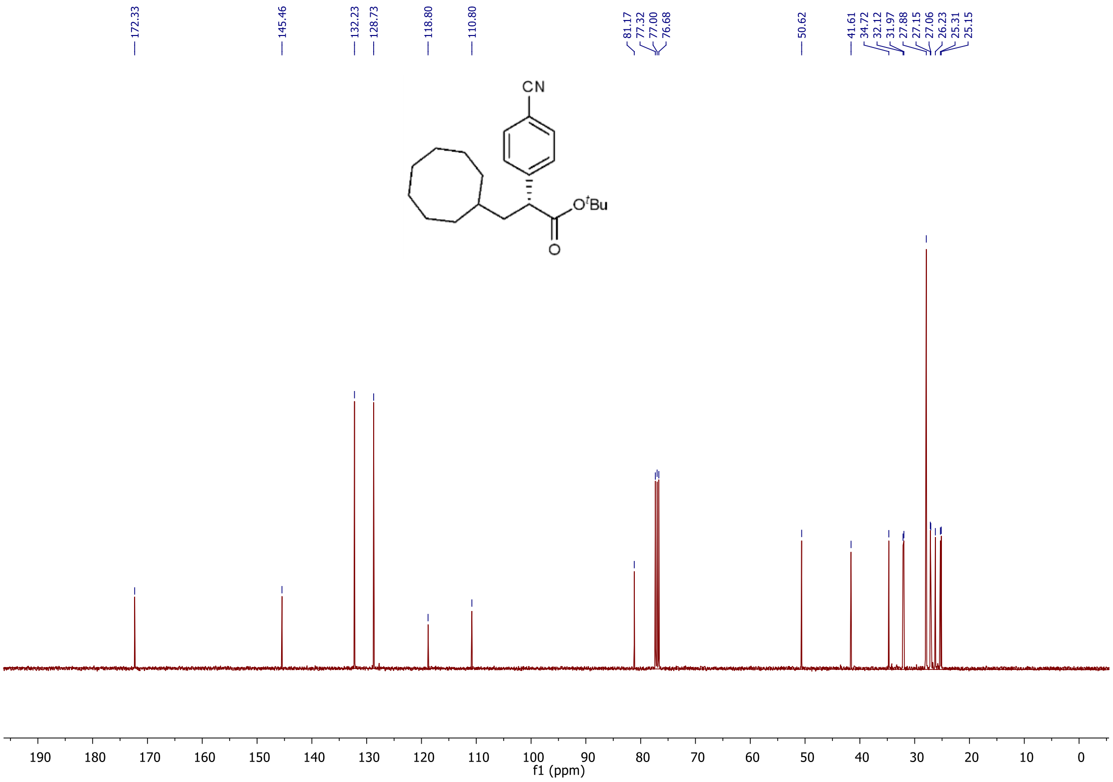

Supplementary Figure 114. ^{13}C NMR of compound **48**.

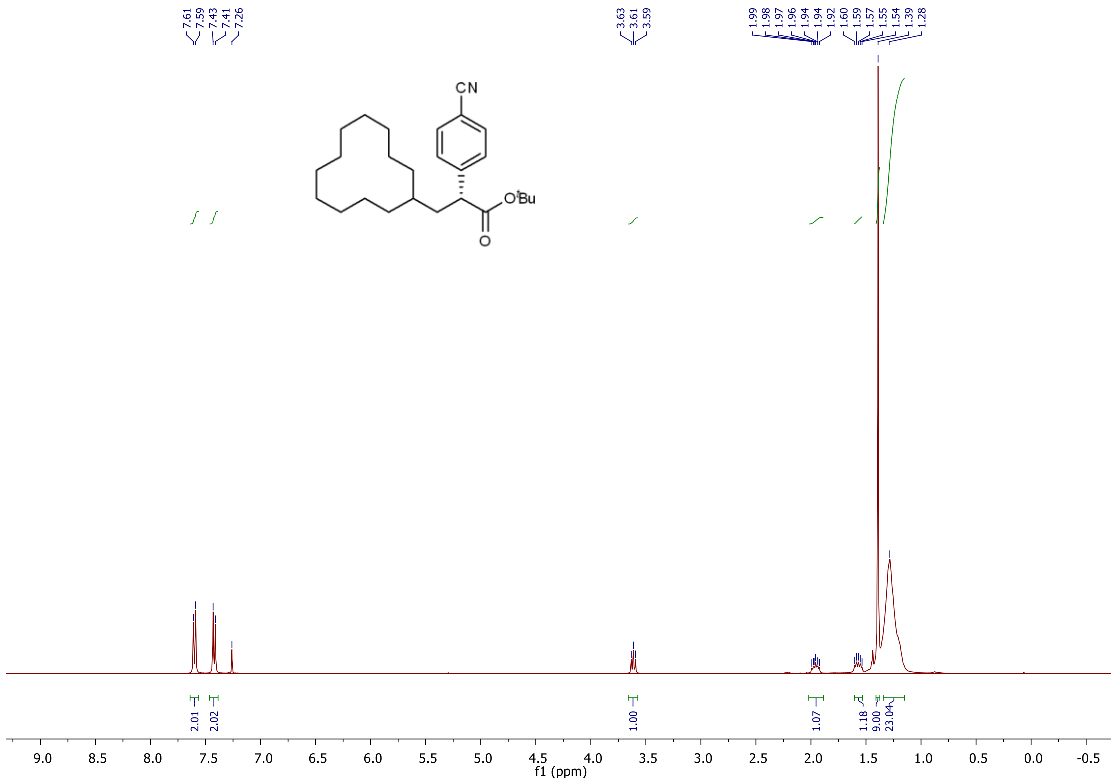

Supplementary Figure 115. ^{1}H NMR of compound **49**.

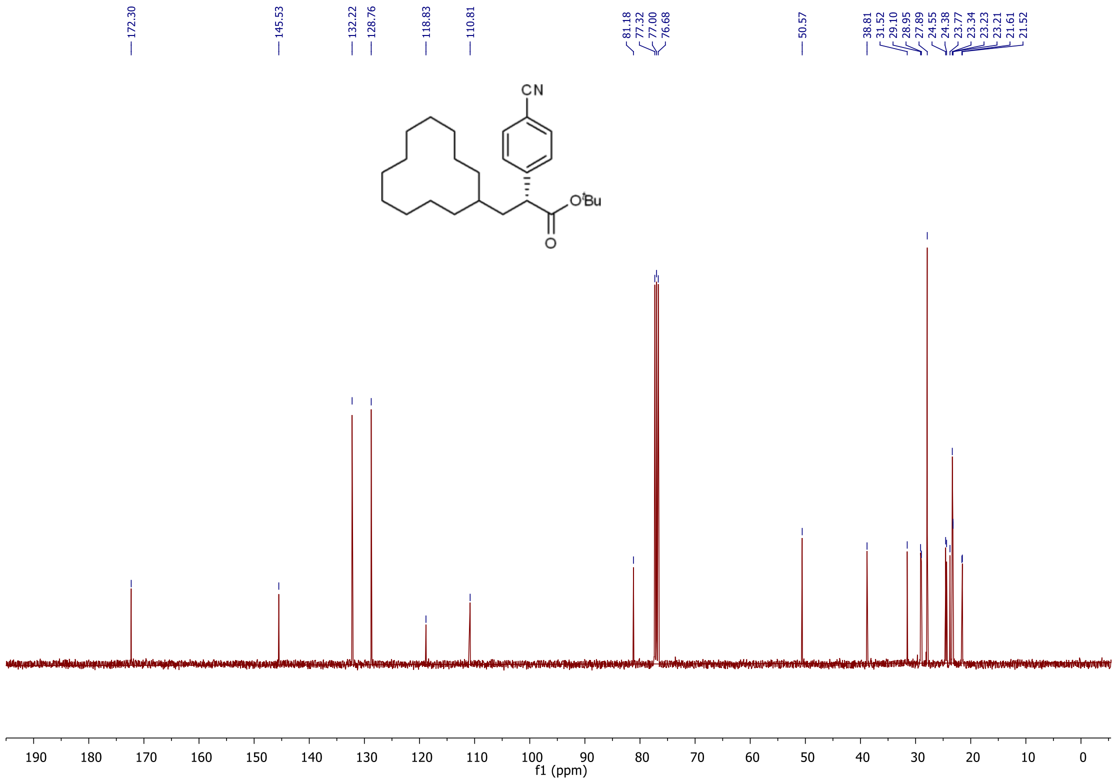

Supplementary Figure 116. ^{13}C NMR of compound **49**.

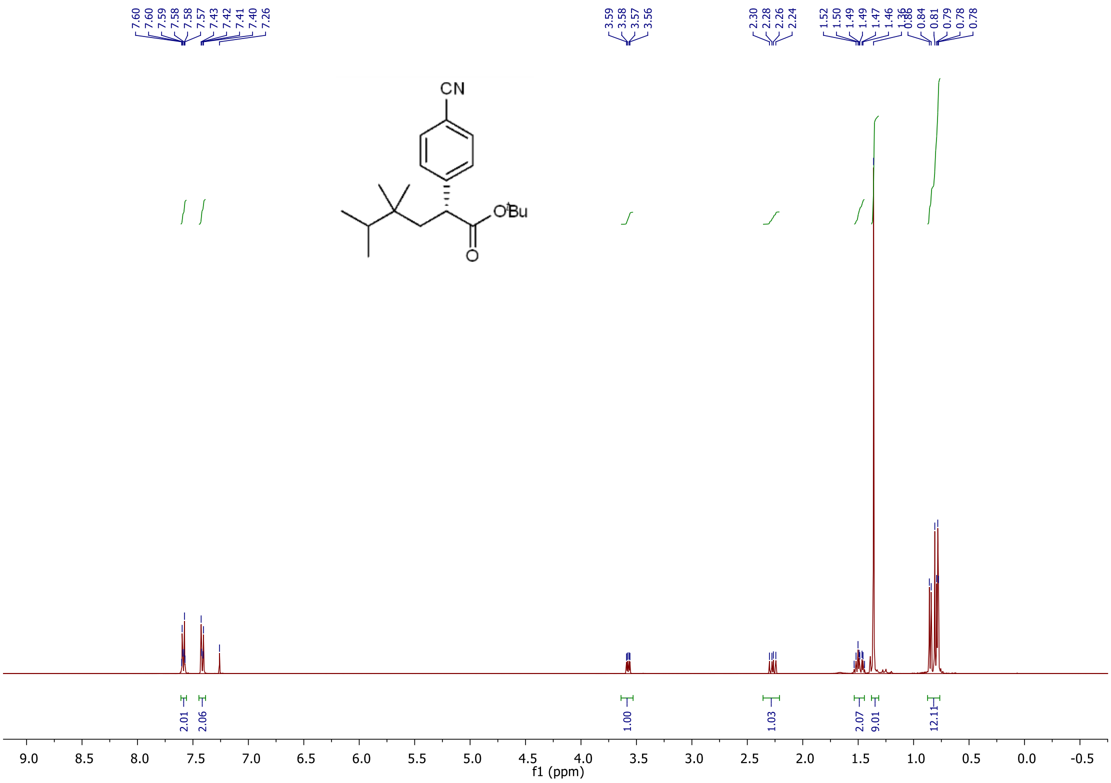

Supplementary Figure 117. ^{1}H NMR of compound **50**.

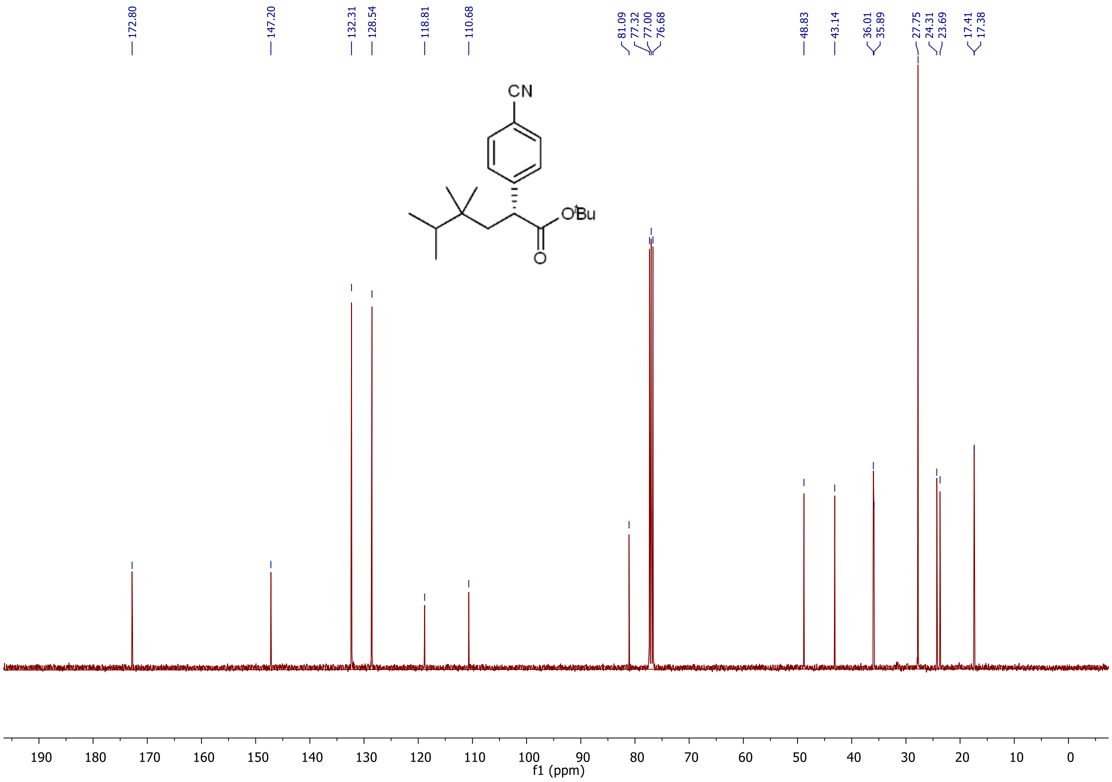

Supplementary Figure 118. ^{13}C NMR of compound **50**.

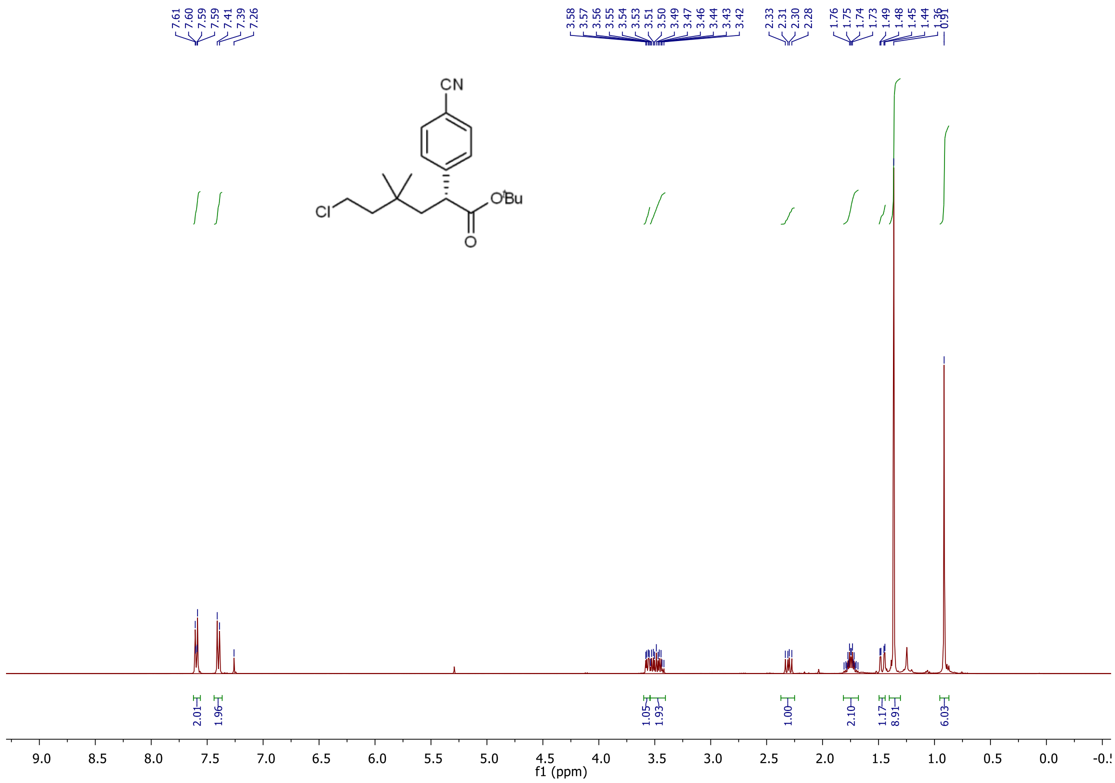

Supplementary Figure 119. ^{1}H NMR of compound **51**.

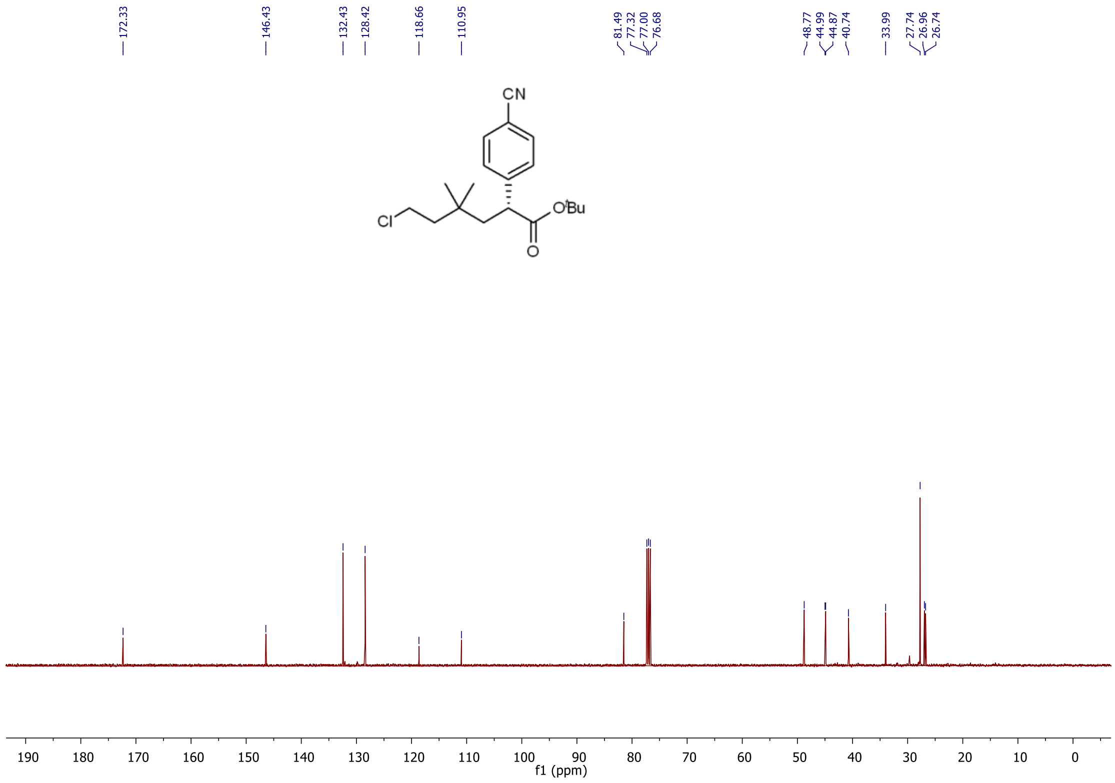

Supplementary Figure 120. ^{13}C NMR of compound **51**.

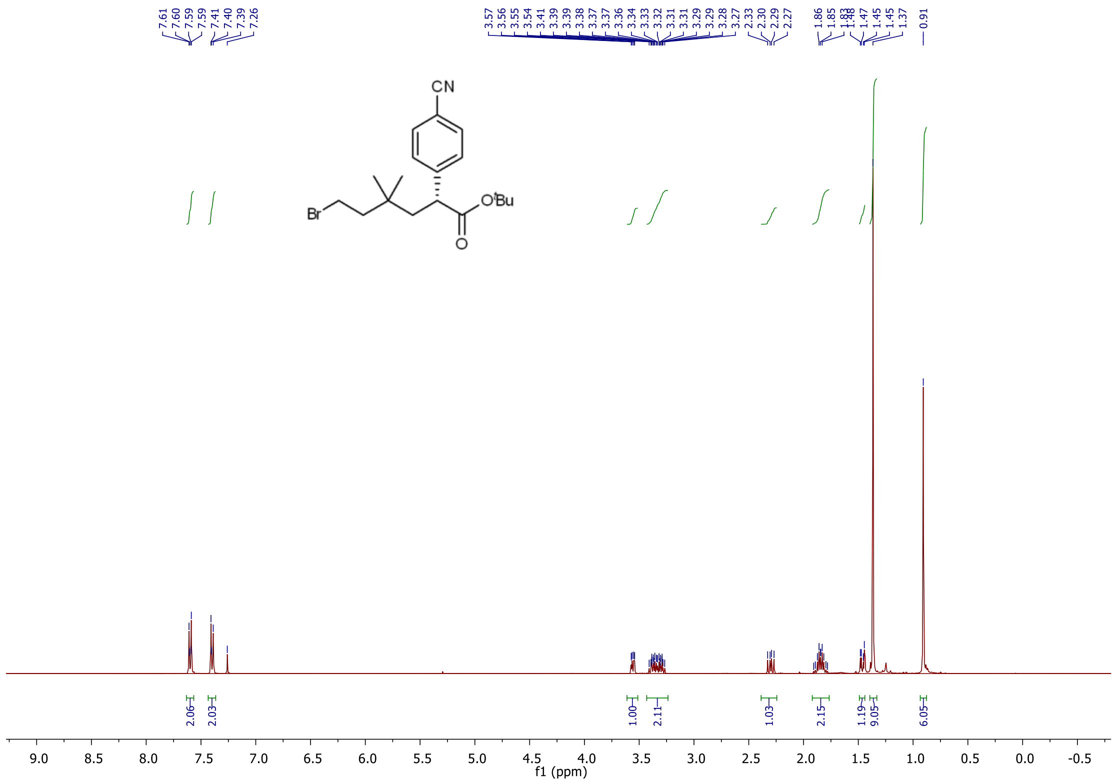

Supplementary Figure 121. ^{1}H NMR of compound **52**.

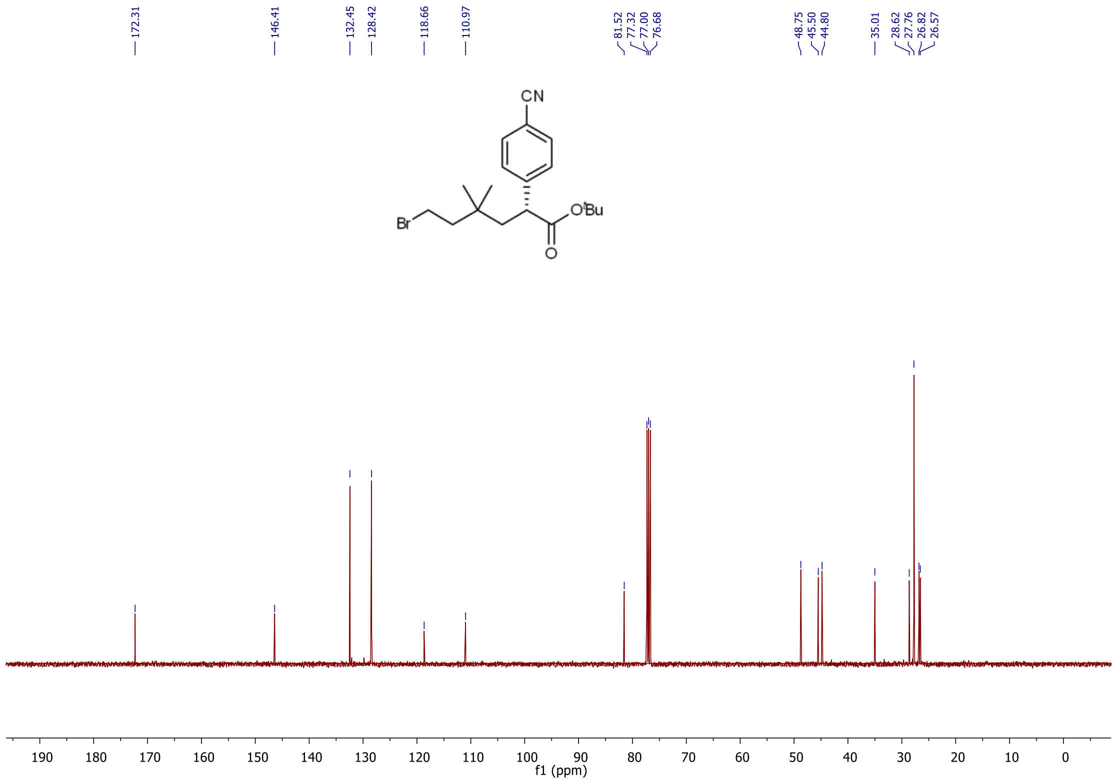

Supplementary Figure 122. ^{13}C NMR of compound **52**.

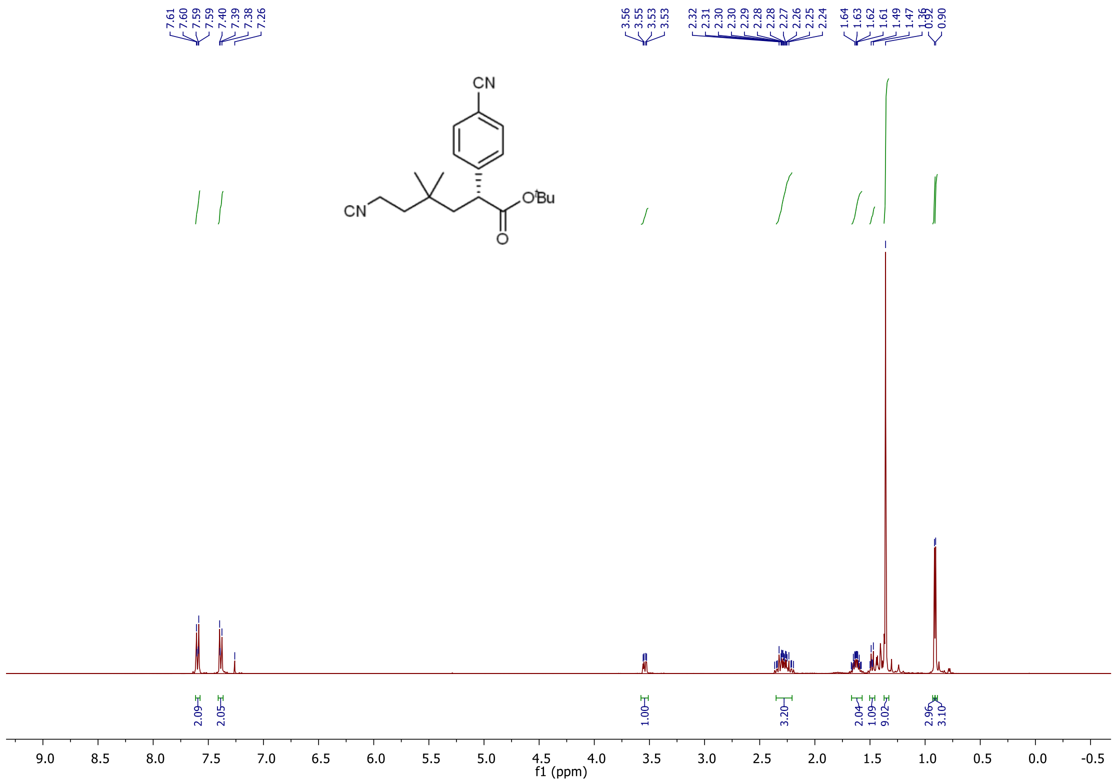

Supplementary Figure 123. ^{1}H NMR of compound **53**.

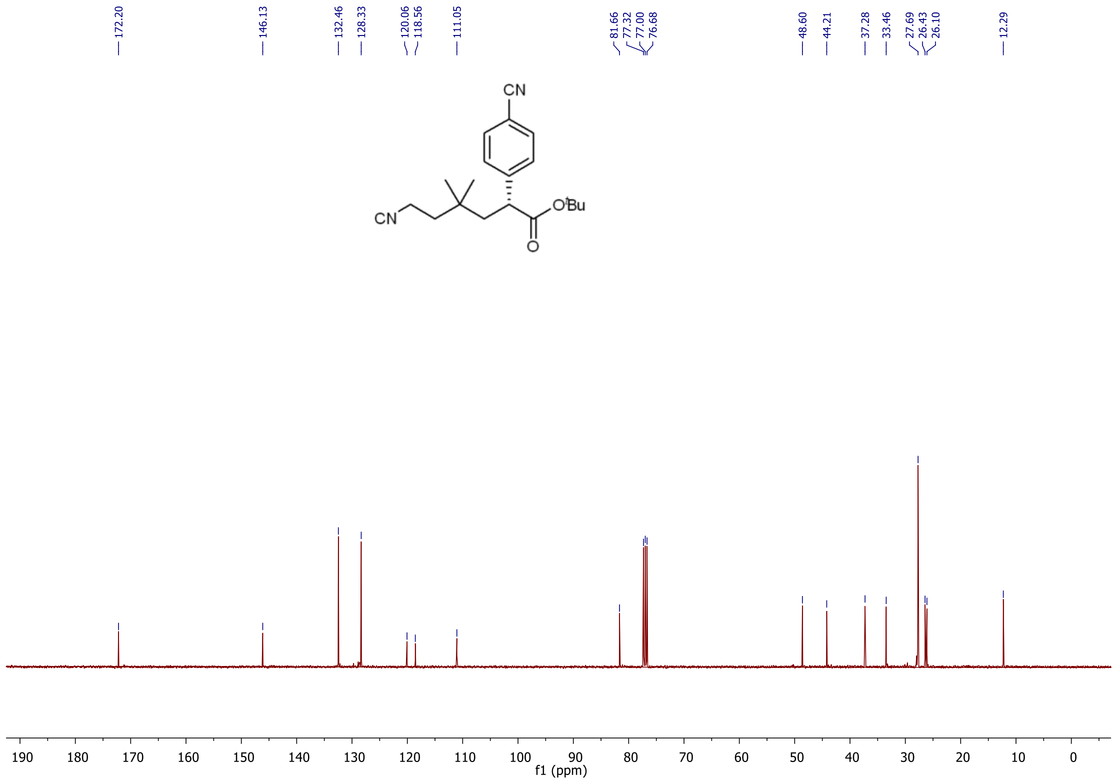

Supplementary Figure 124. ^{13}C NMR of compound **53**.

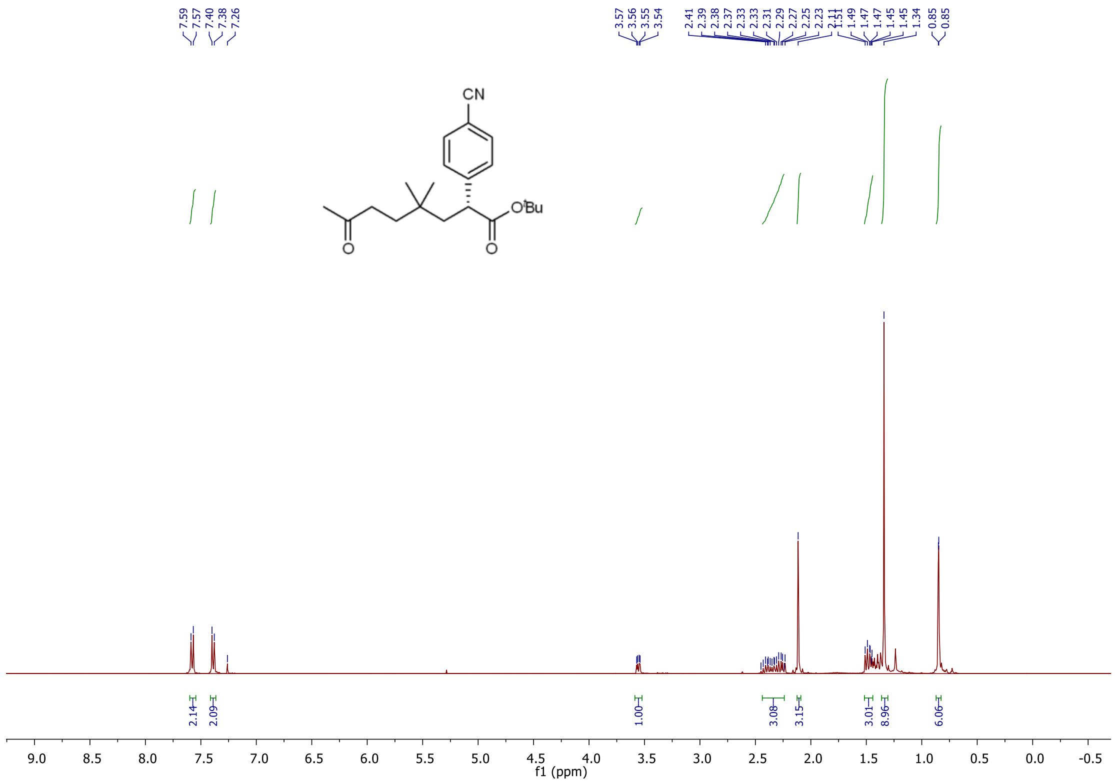

Supplementary Figure 125. ^{1}H NMR of compound **54**.

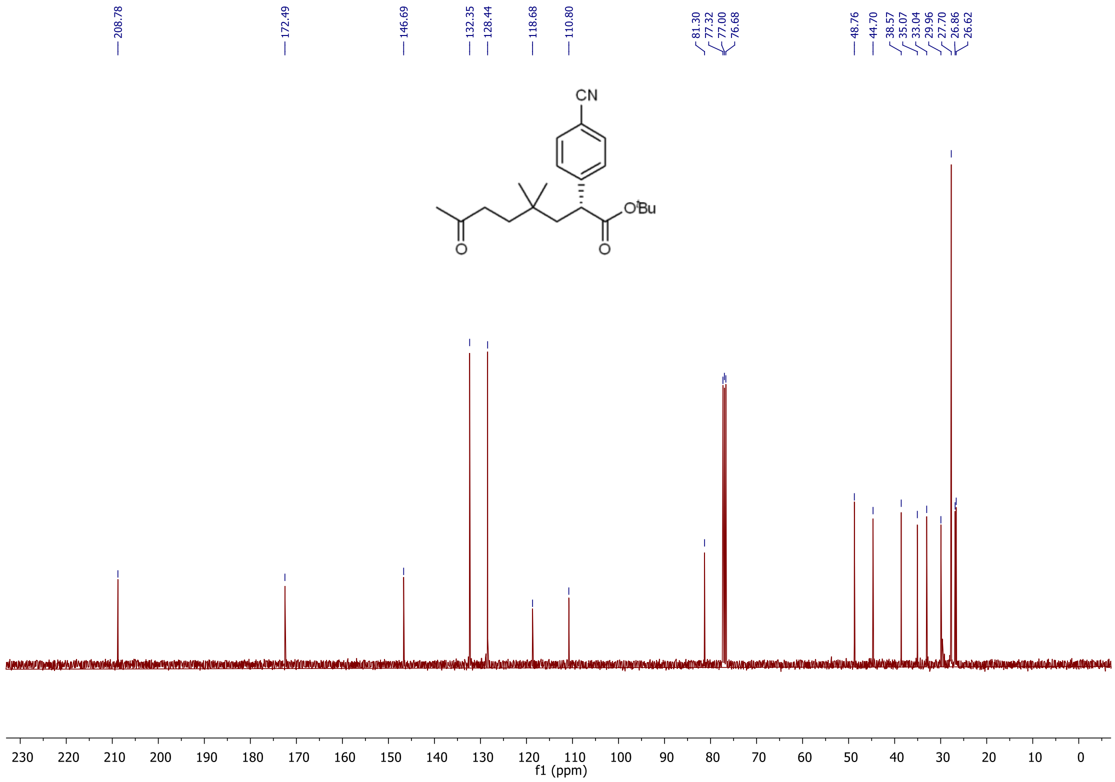

Supplementary Figure 126. ^{13}C NMR of compound **54**.

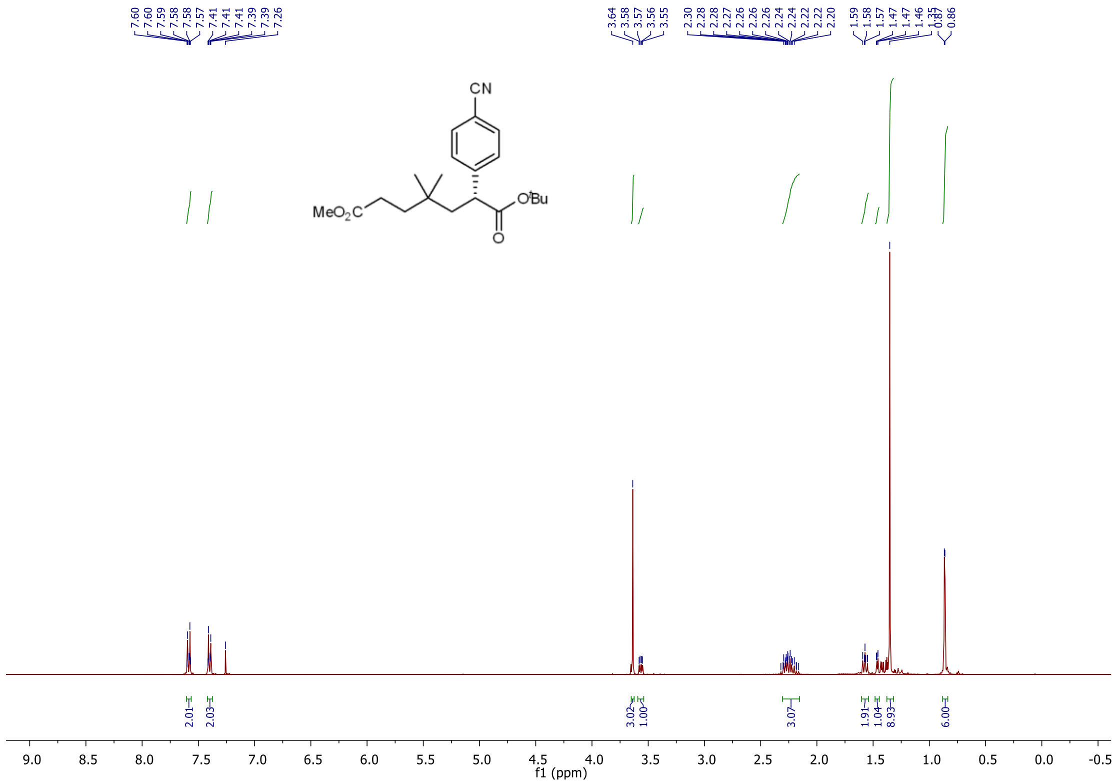

Supplementary Figure 127. ^{1}H NMR of compound **55**.

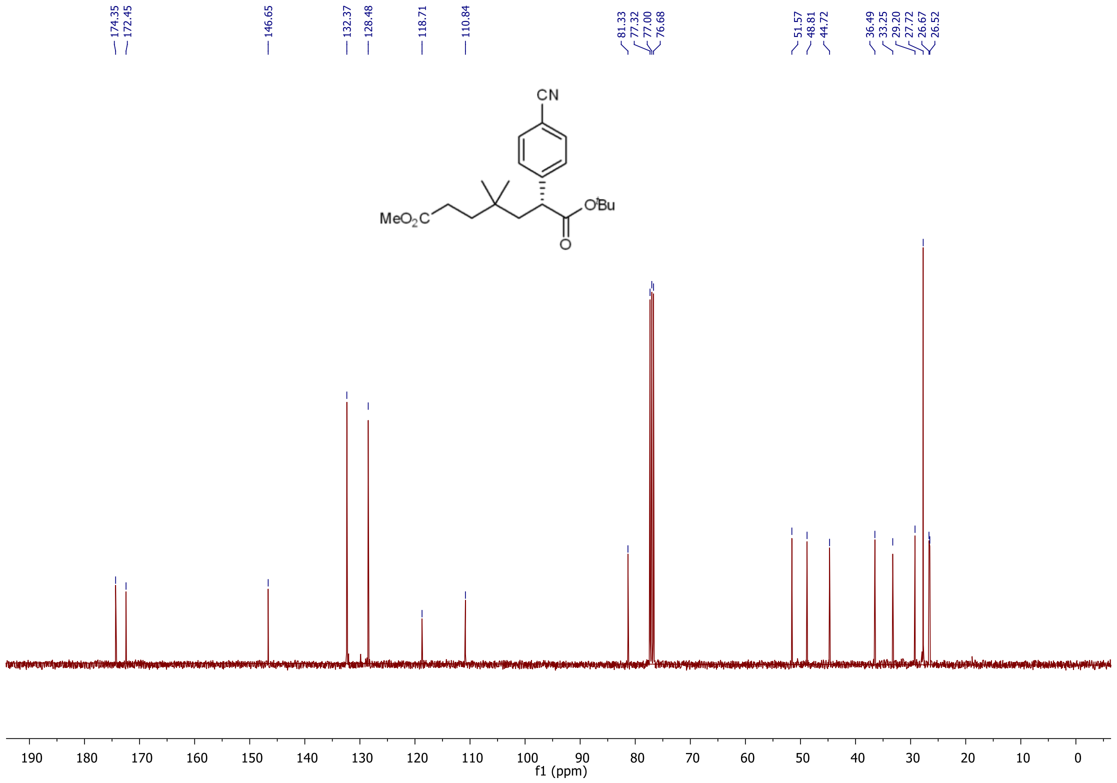

Supplementary Figure 128. ^{13}C NMR of compound **55**.

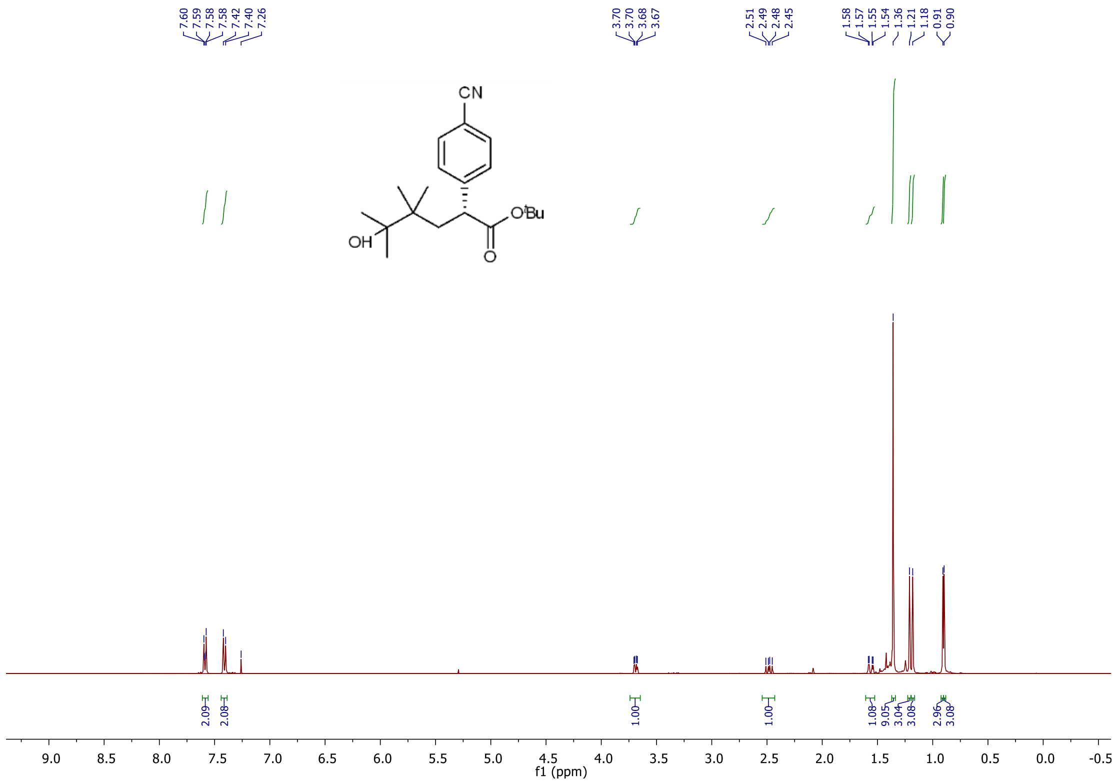

Supplementary Figure 129. ^{1}H NMR of compound **56**.

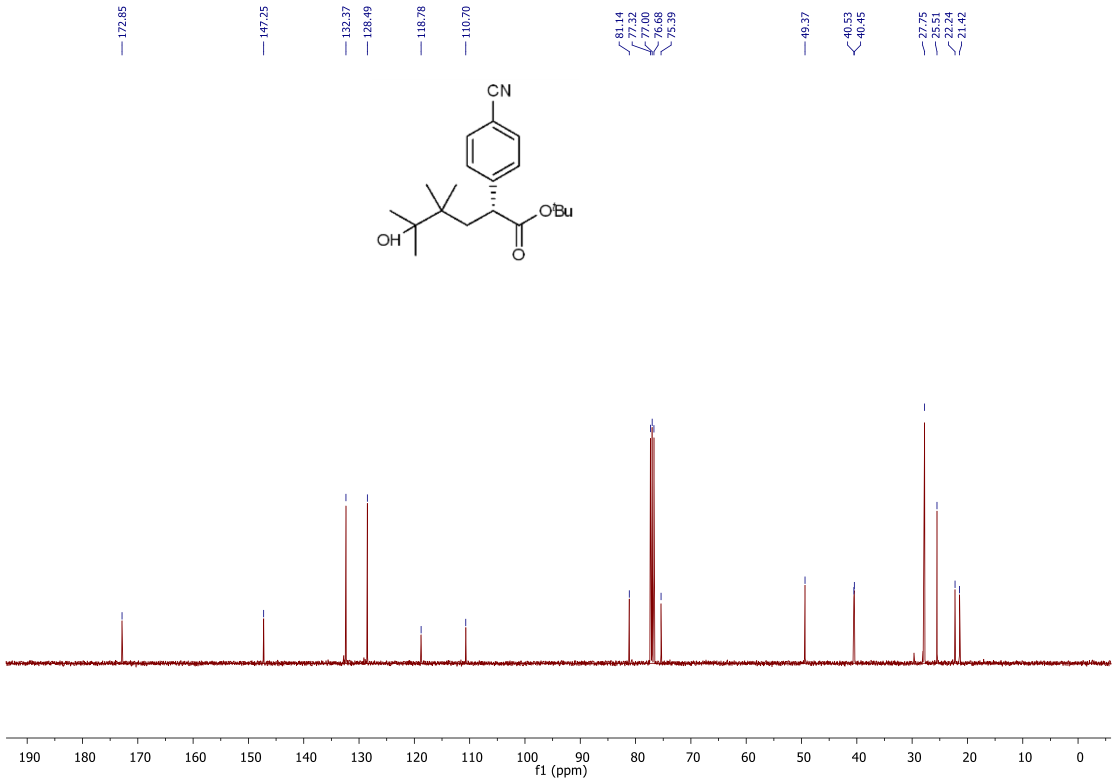

Supplementary Figure 130. ^{13}C NMR of compound **56**.

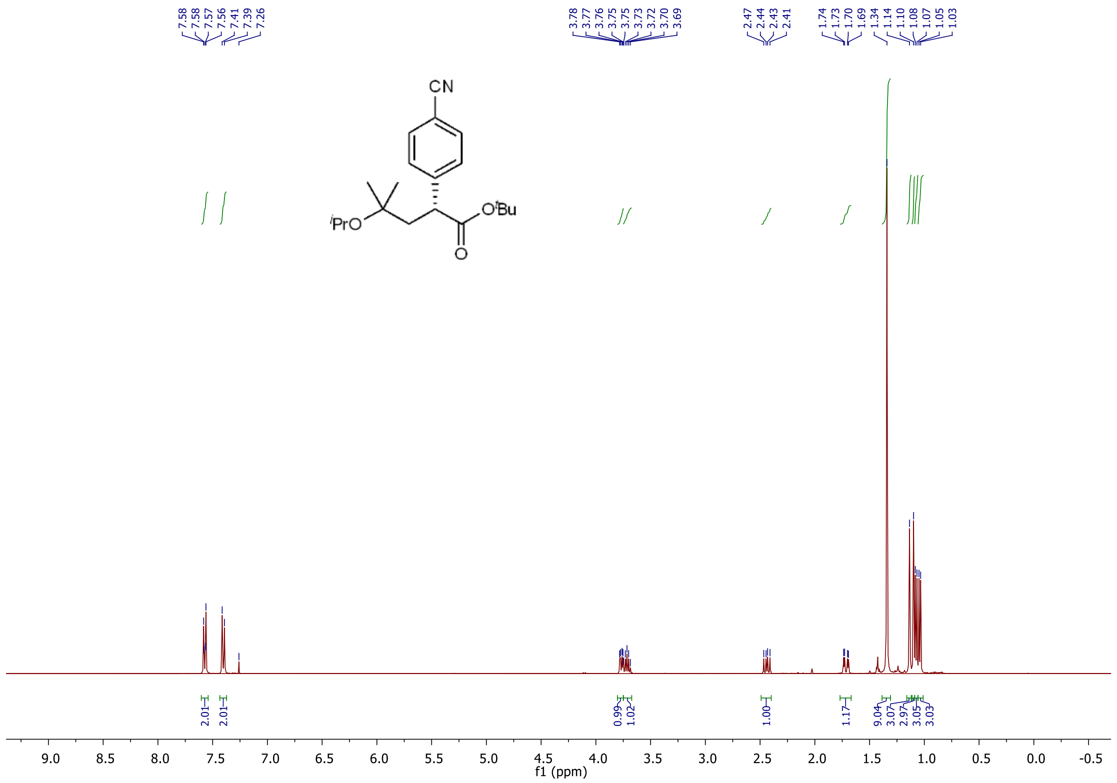

Supplementary Figure 131. ^{1}H NMR of compound **57**.

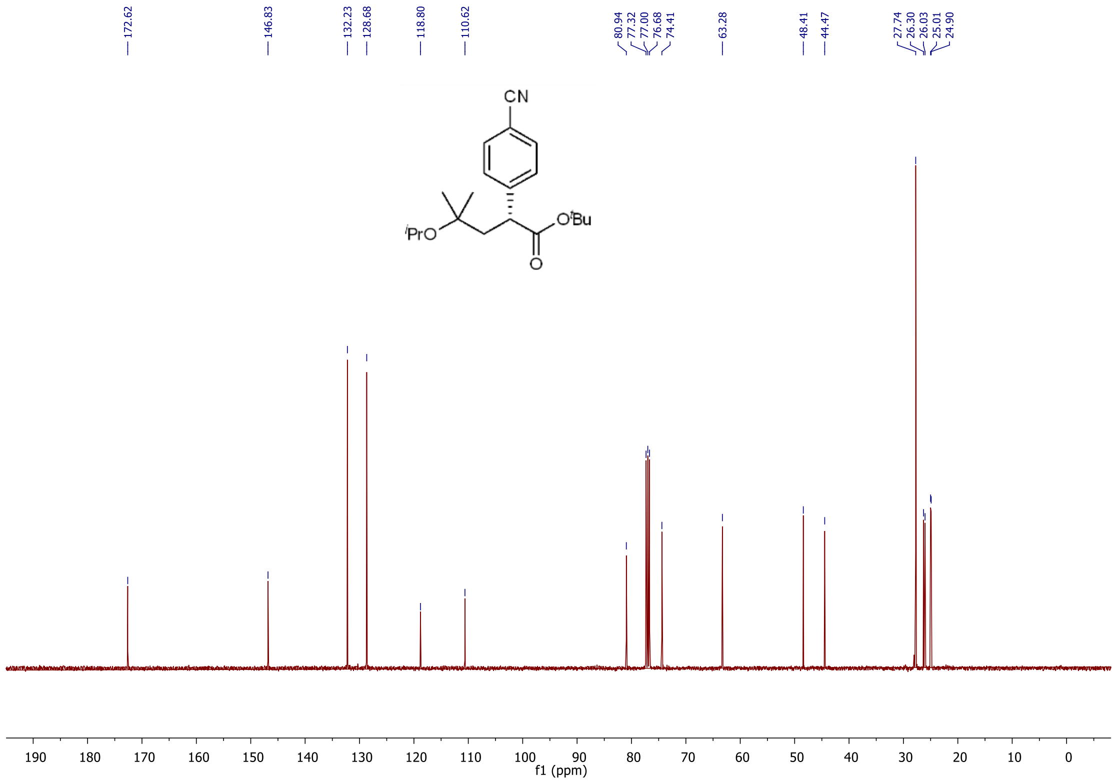

Supplementary Figure 132. ^{13}C NMR of compound **57**.

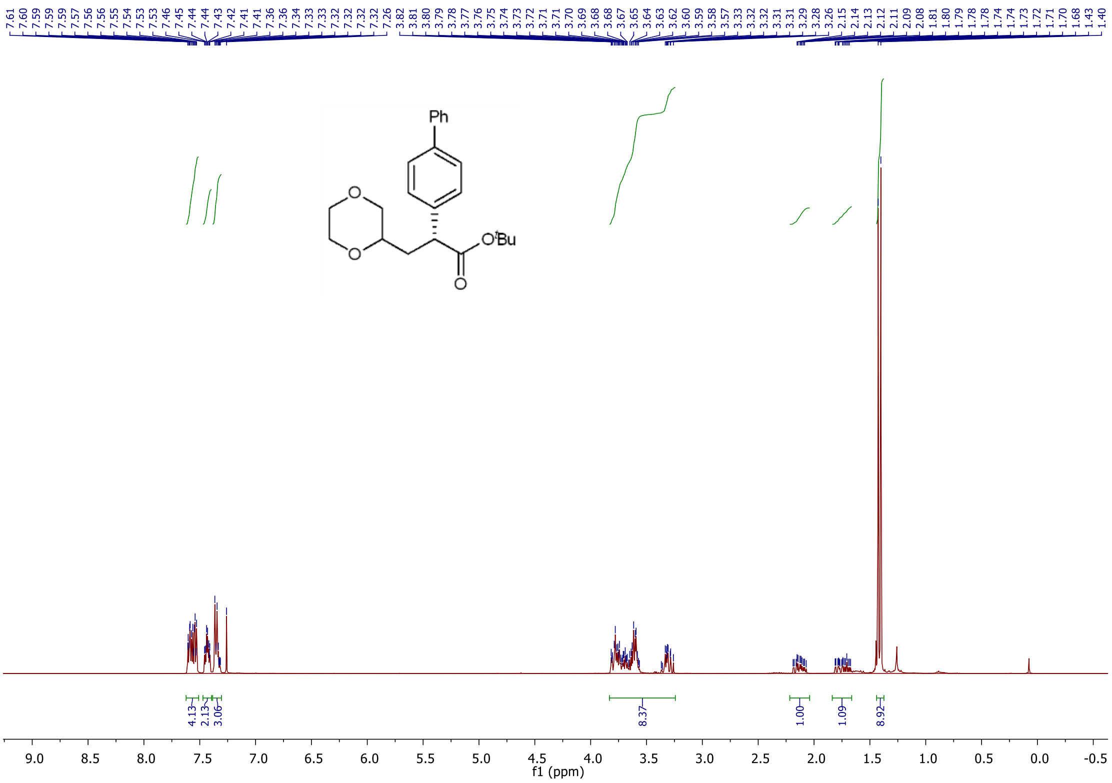

Supplementary Figure 133. ^{1}H NMR of compound **58**.

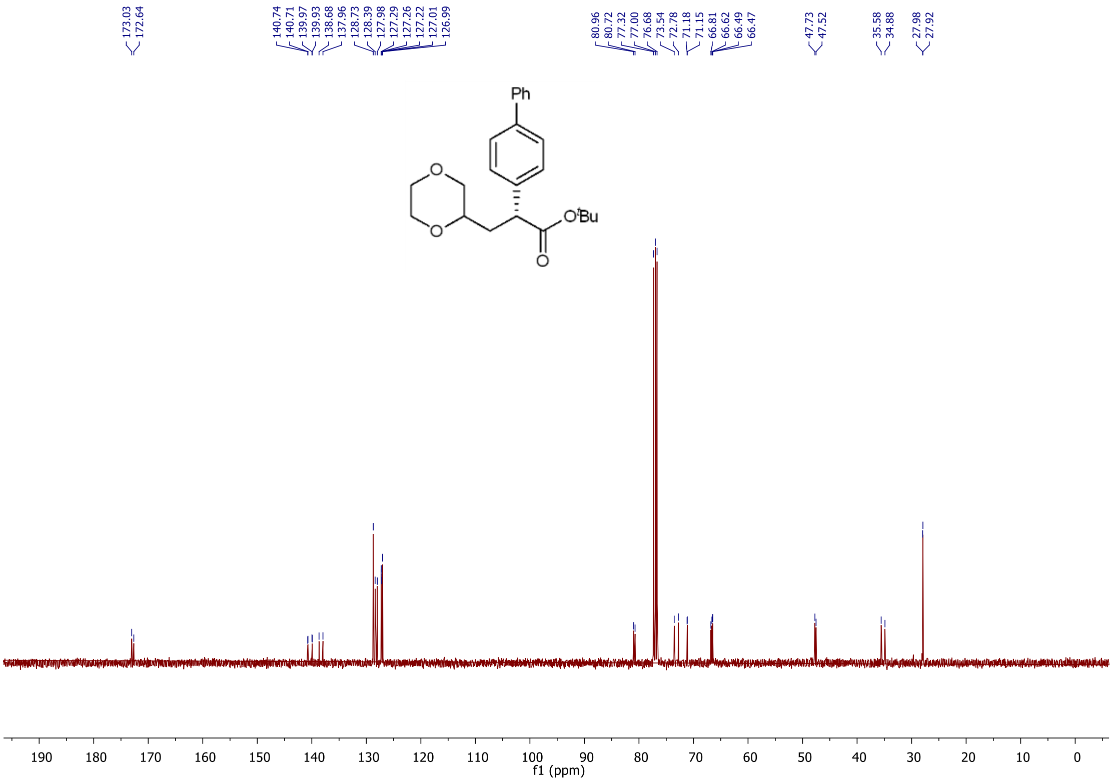

Supplementary Figure 134. ^{13}C NMR of compound **58**.

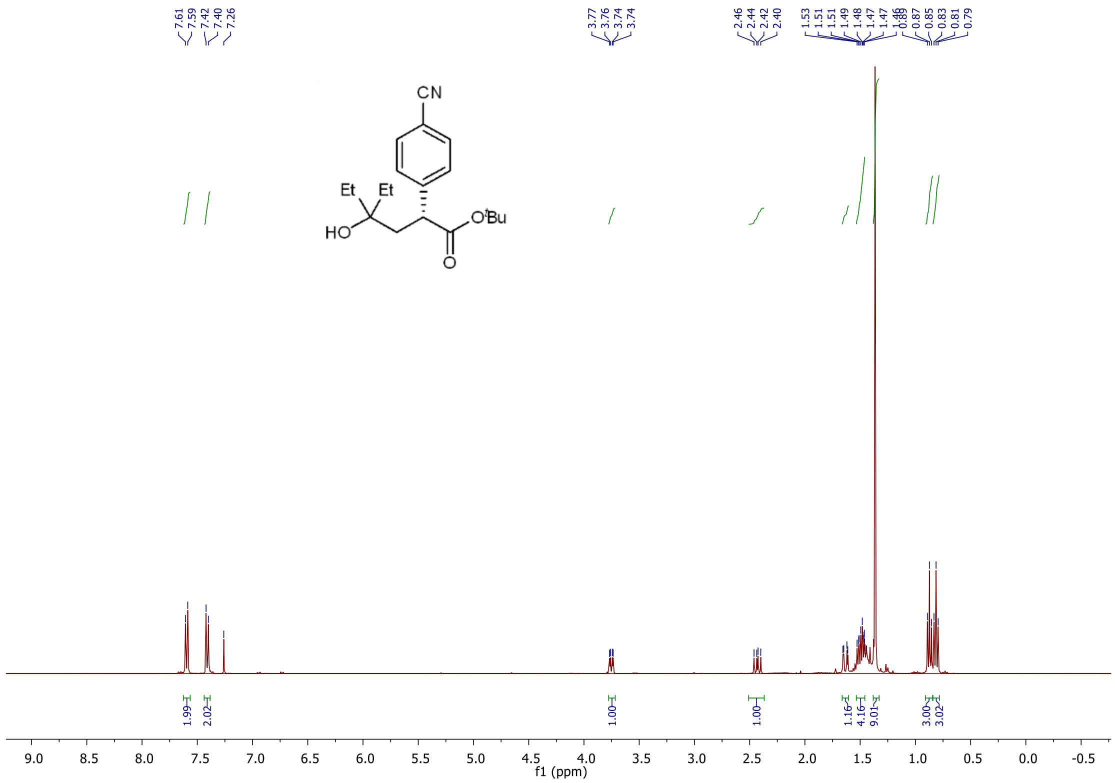

Supplementary Figure 135. ^{1}H NMR of compound **59**.

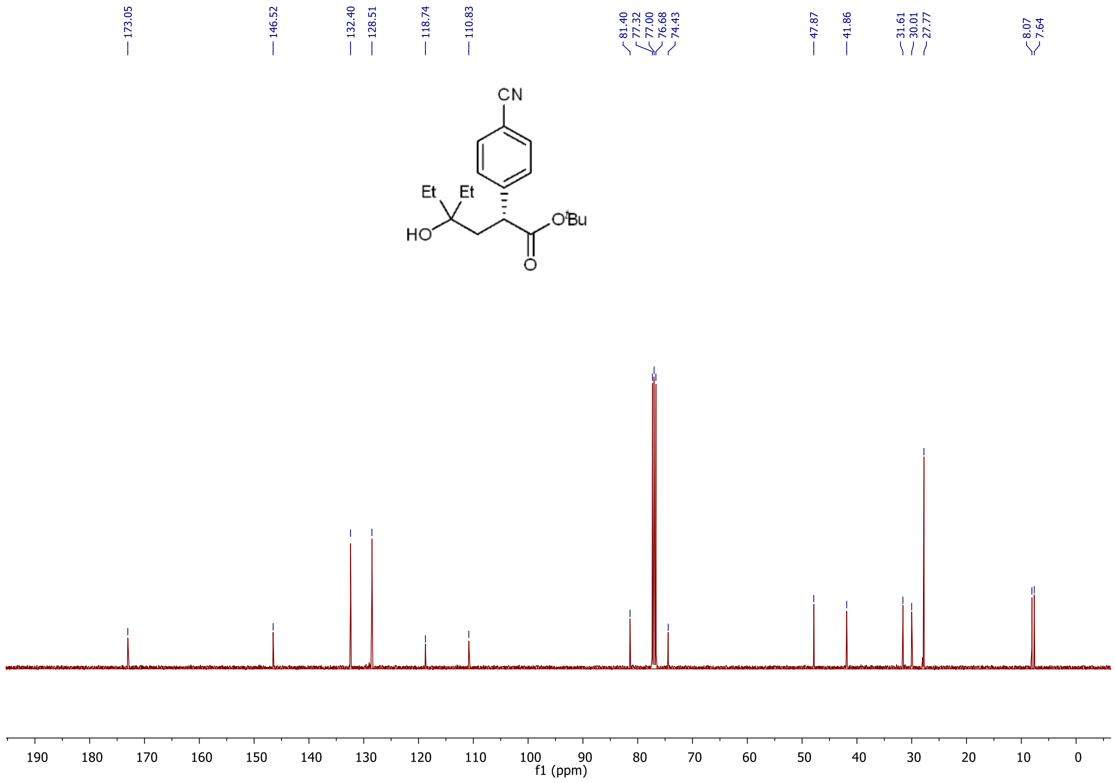

Supplementary Figure 136. ^{13}C NMR of compound **59**.

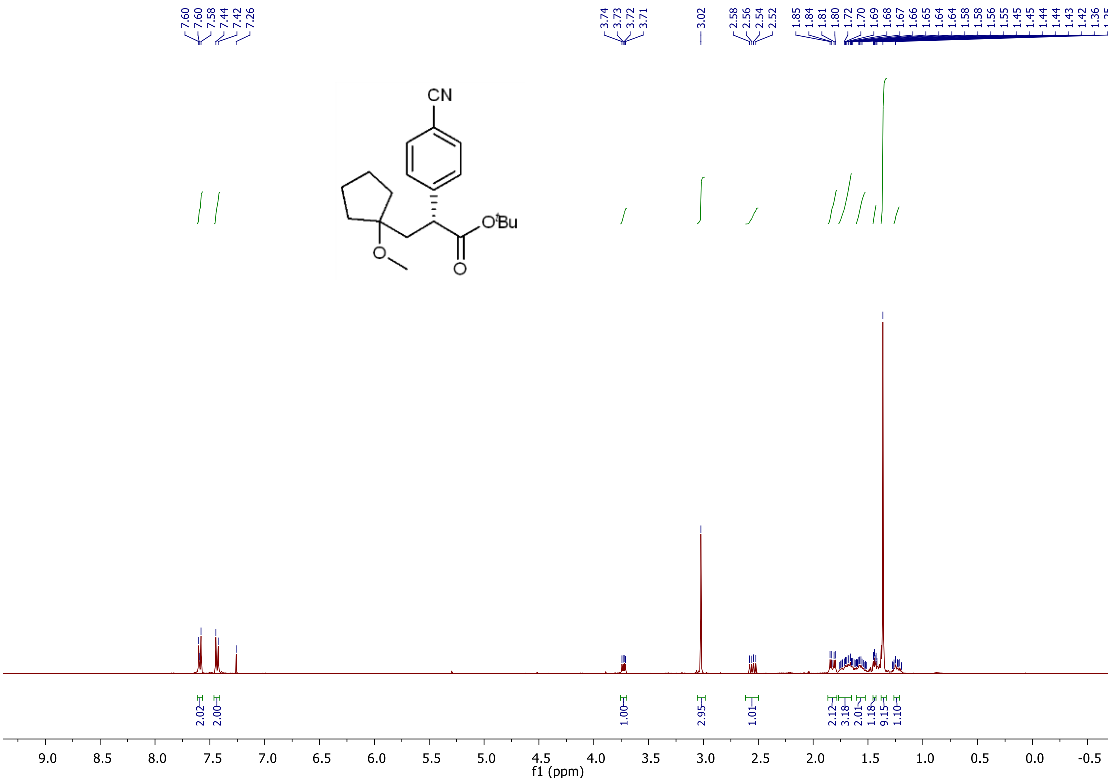

Supplementary Figure 137. ^{1}H NMR of compound **60**.

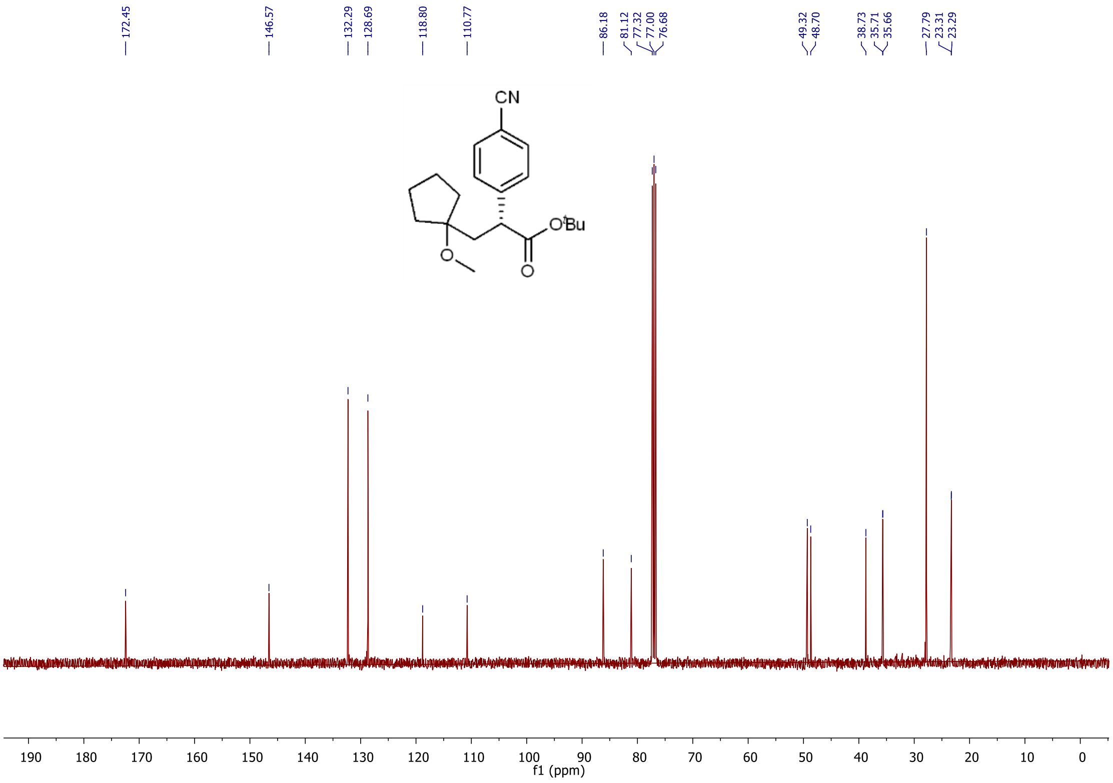

Supplementary Figure 138. ^{13}C NMR of compound **60**.

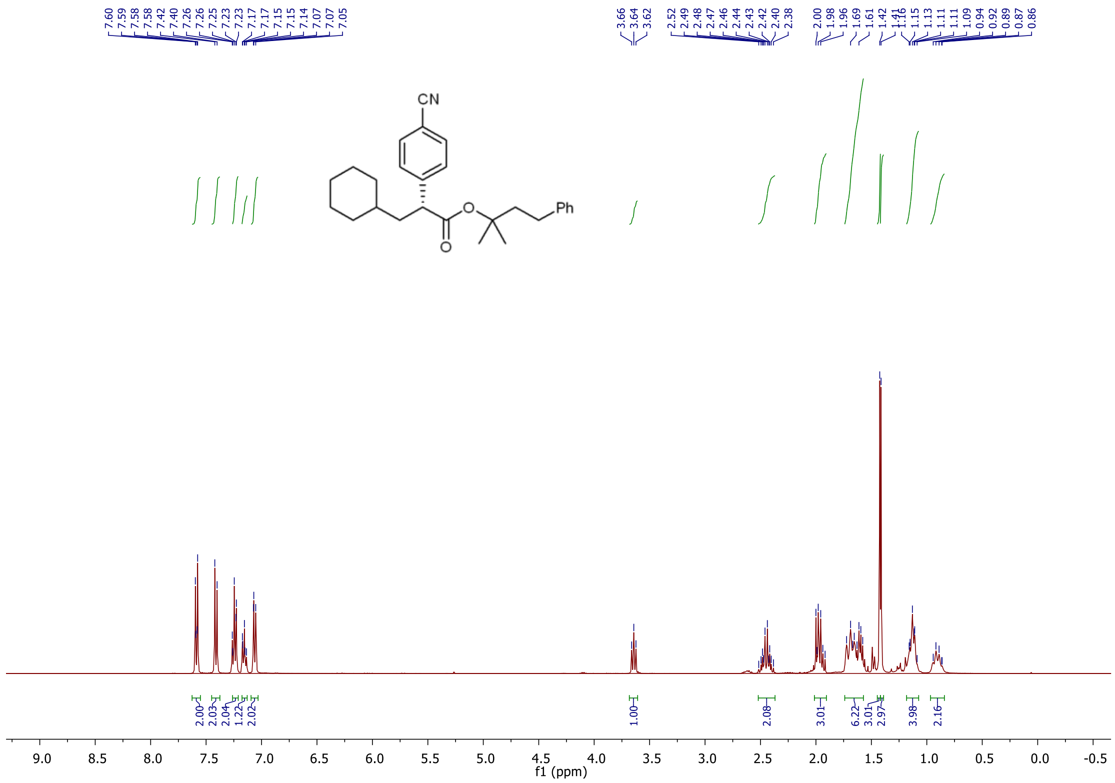

Supplementary Figure 139. ^{1}H NMR of compound **61**.

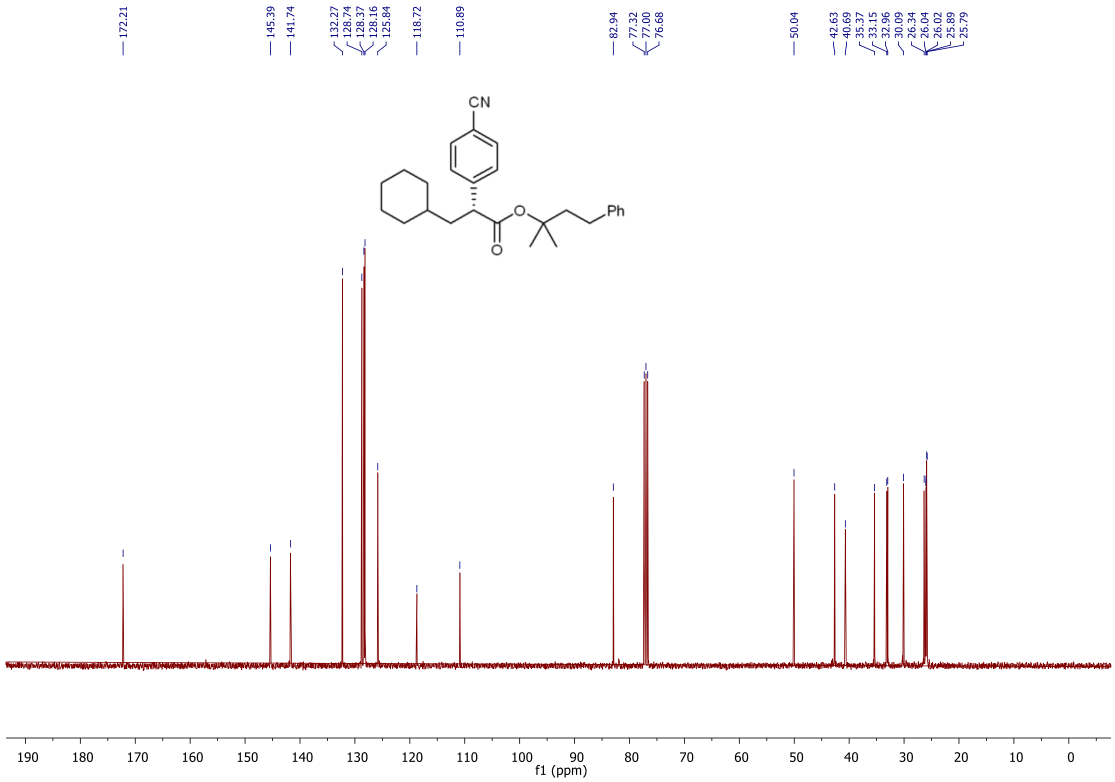

Supplementary Figure 140. ^{13}C NMR of compound **61**.

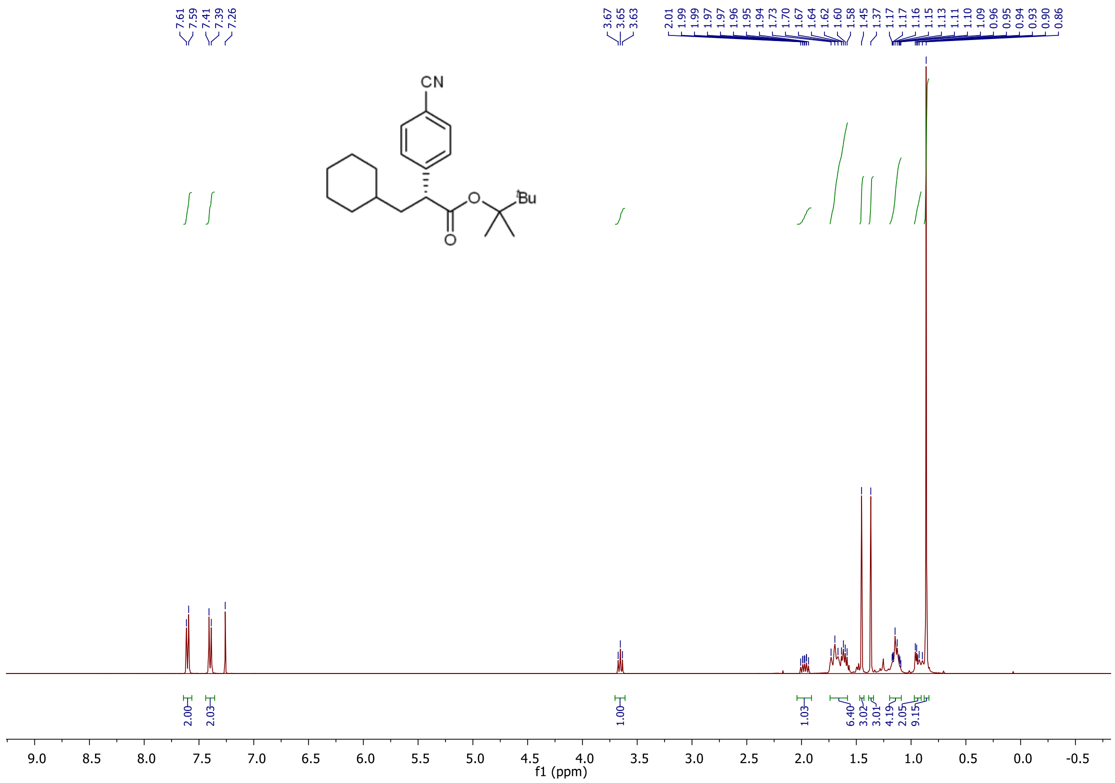

Supplementary Figure 141. ^{1}H NMR of compound **62**.

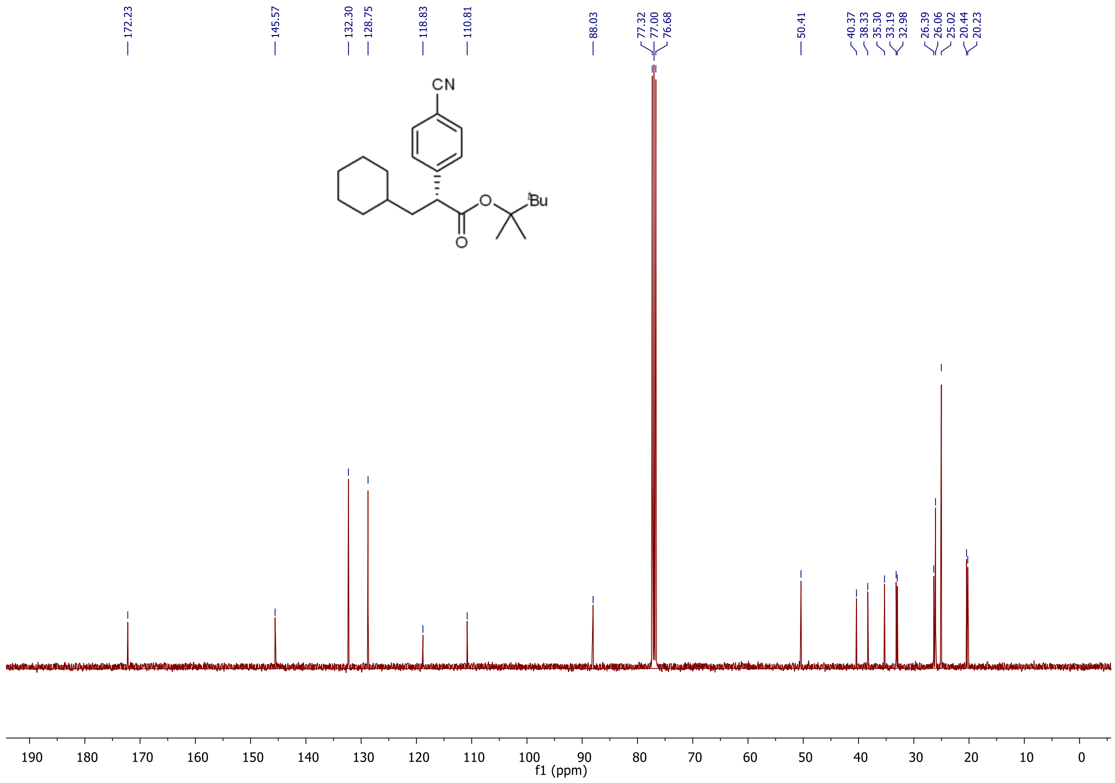

Supplementary Figure 142. ^{13}C NMR of compound **62**.

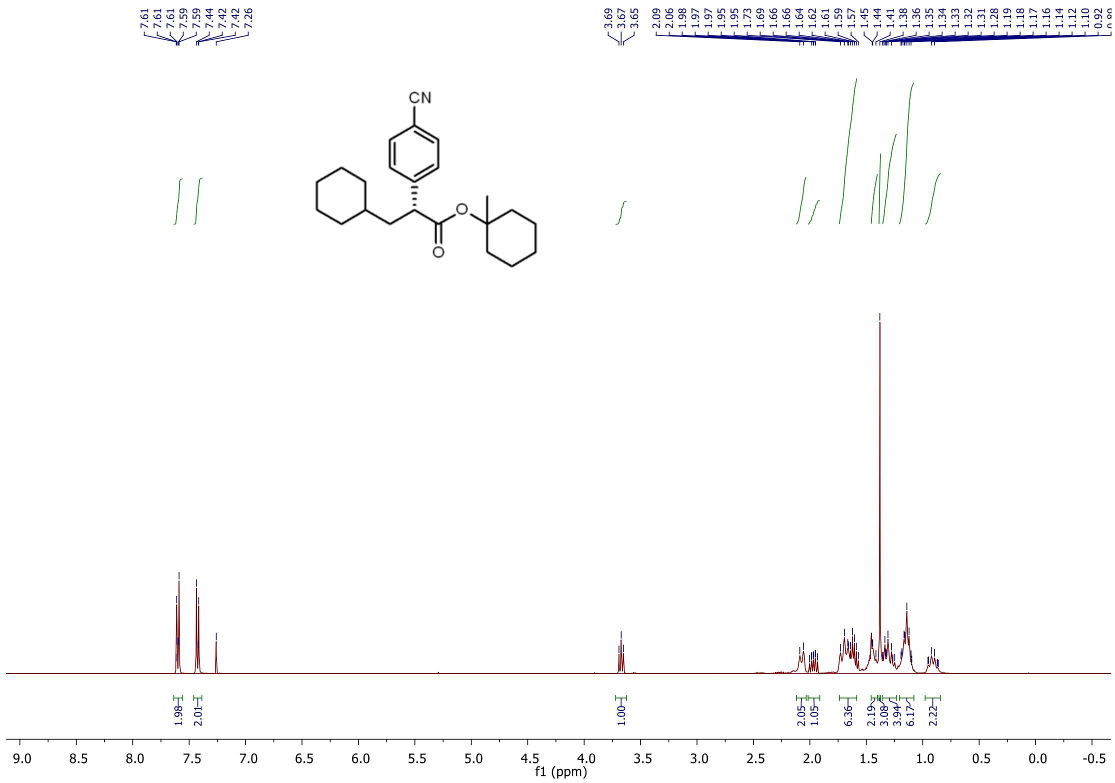

Supplementary Figure 143. ^{1}H NMR of compound **63**.

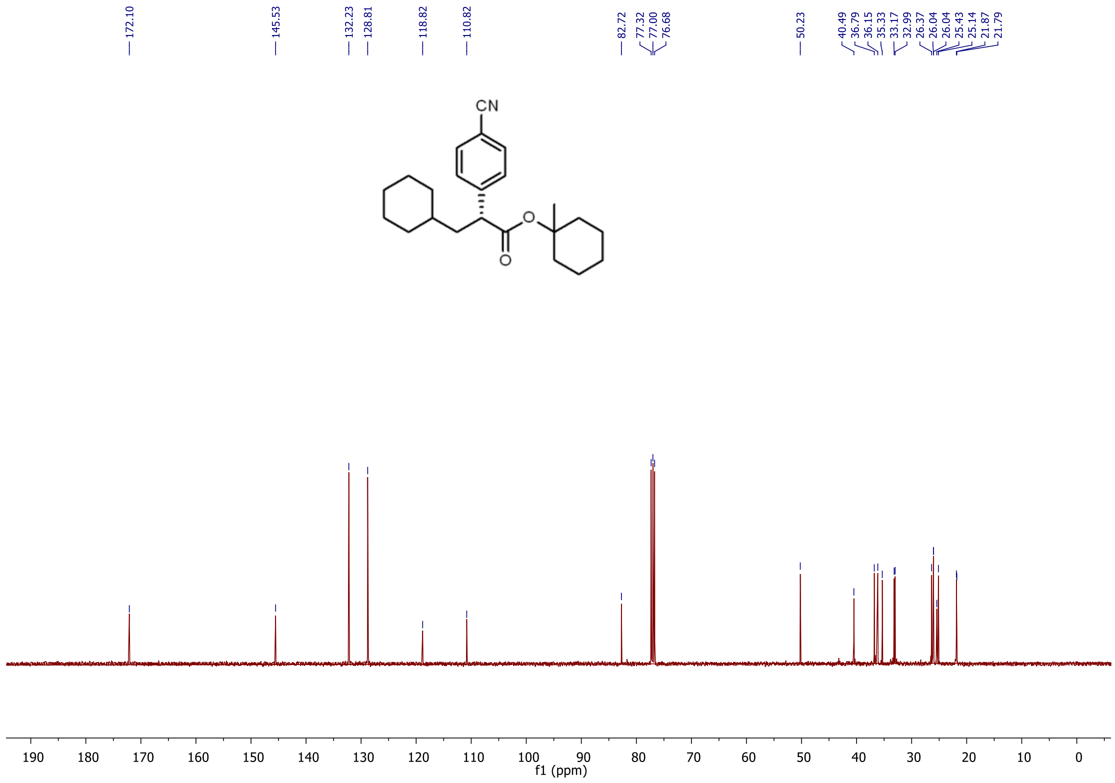

Supplementary Figure 144. ^{13}C NMR of compound **63**.

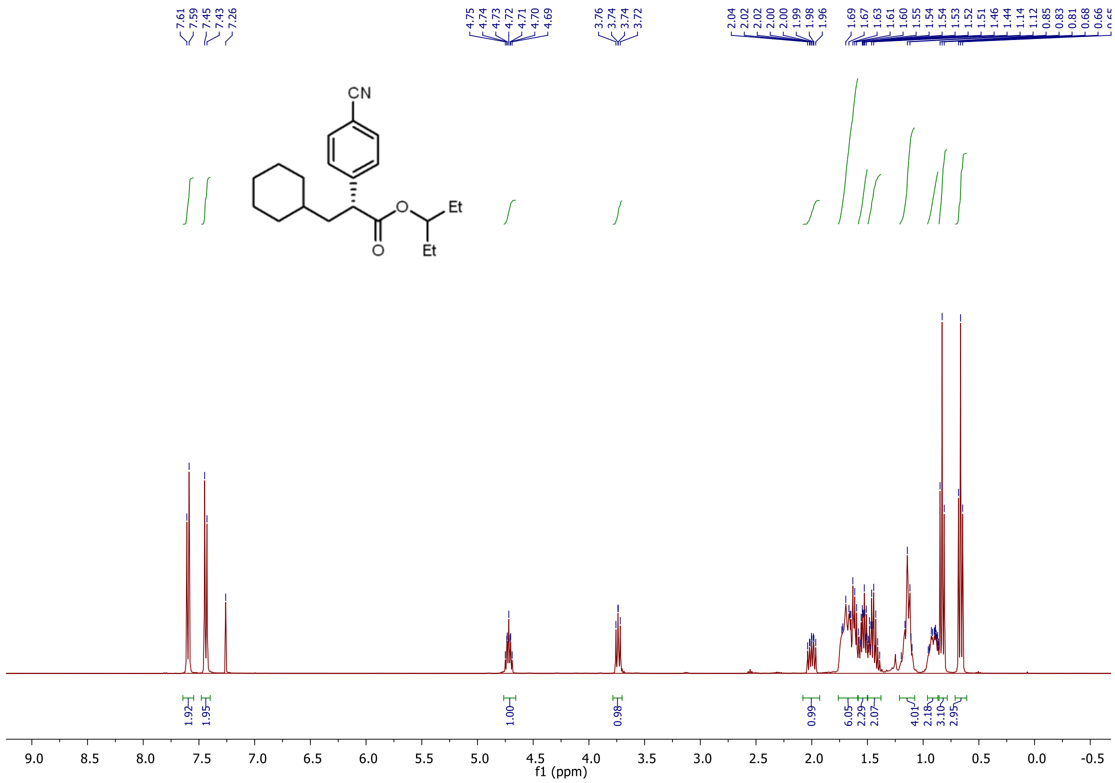

Supplementary Figure 145. ^{1}H NMR of compound **64**.

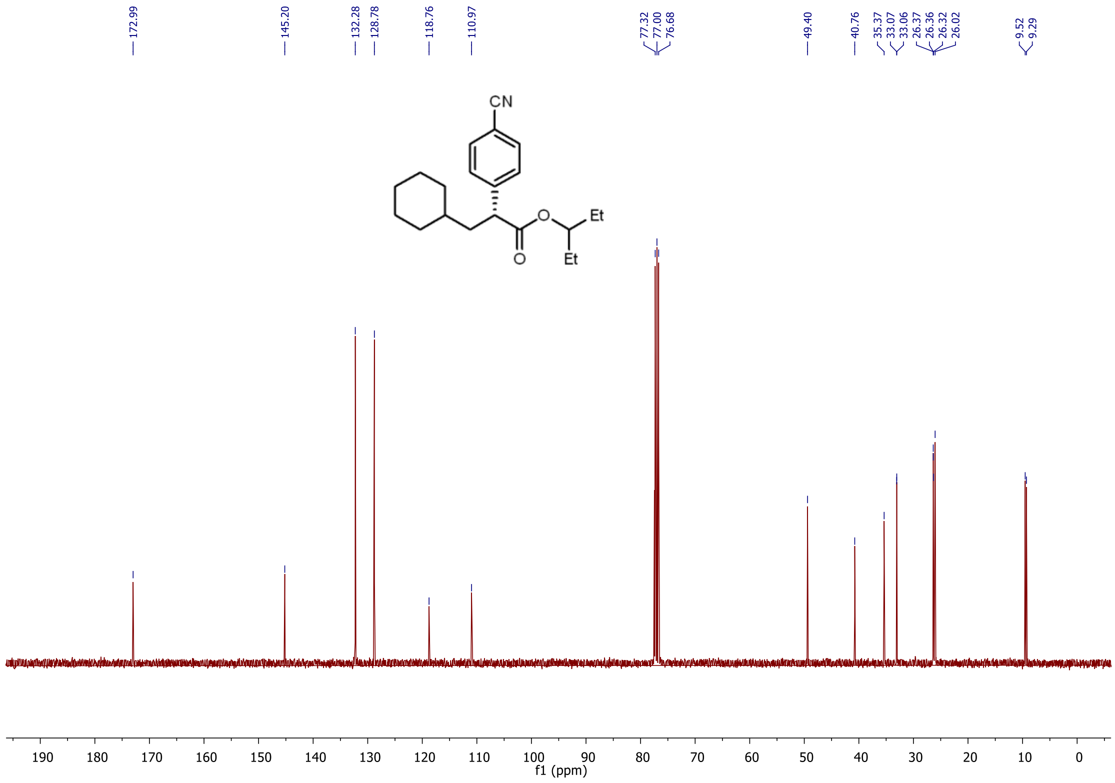

Supplementary Figure 146. ^{13}C NMR of compound **64**.

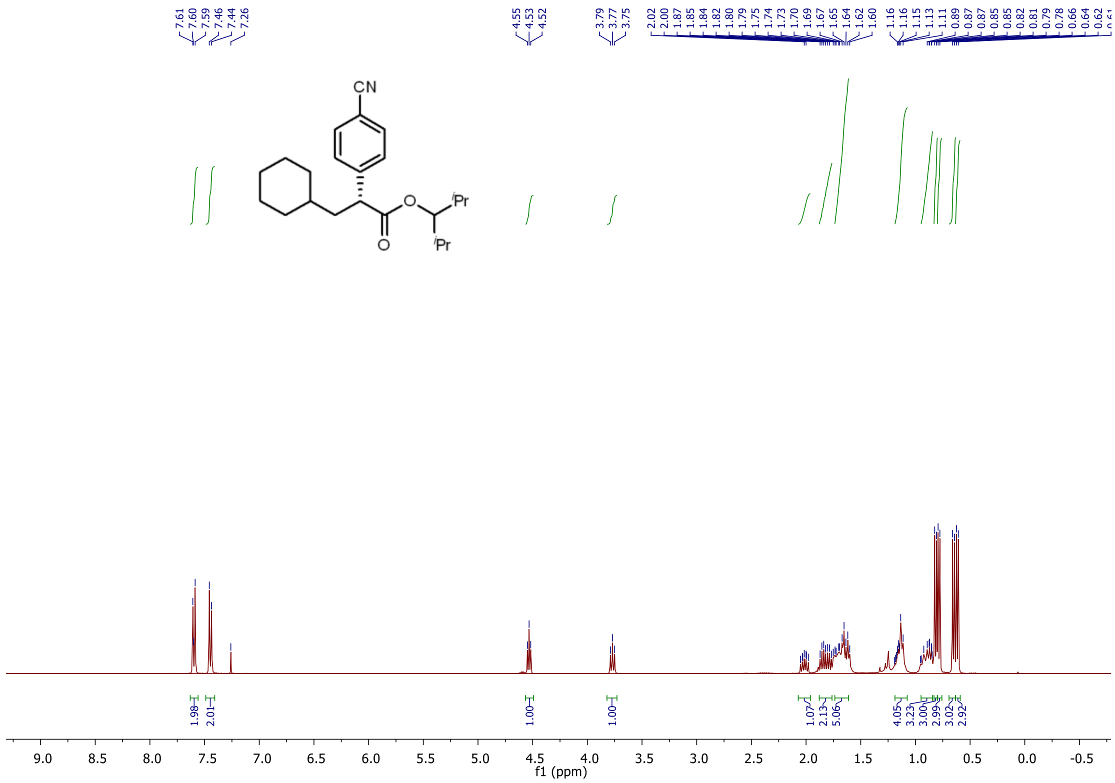

Supplementary Figure 147. ^{1}H NMR of compound **65**.

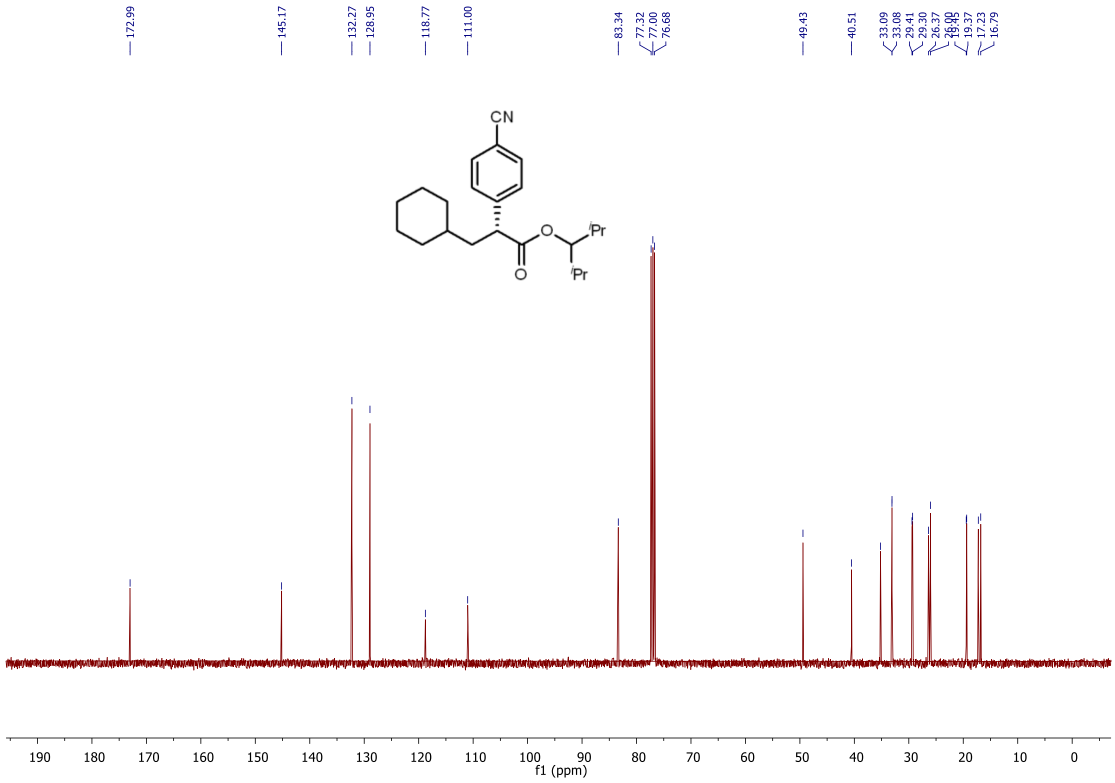

Supplementary Figure 148. ^{13}C NMR of compound **65**.

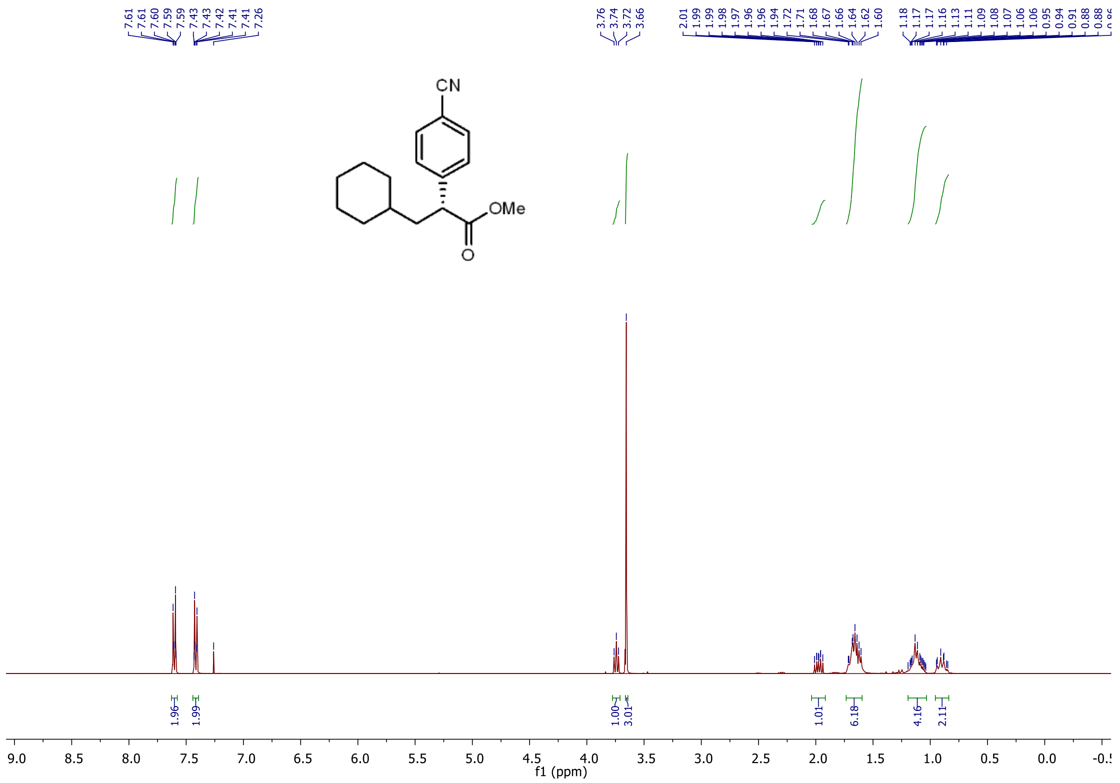

Supplementary Figure 149. ^{1}H NMR of compound **66**.

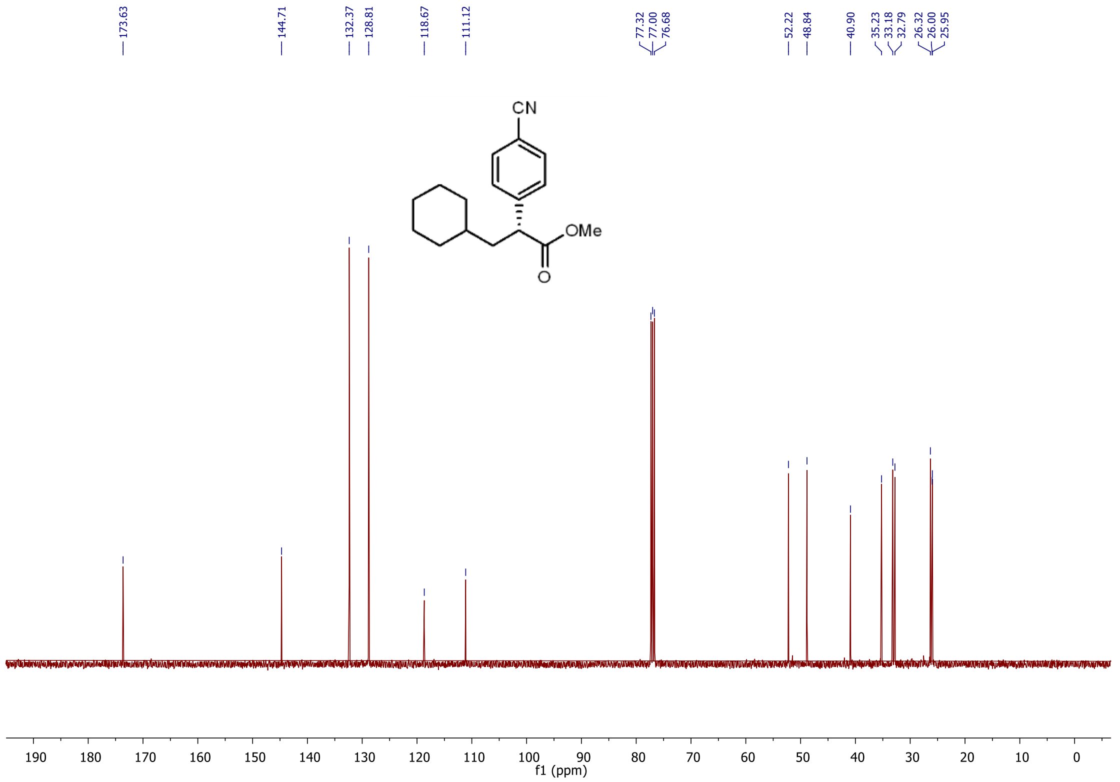

Supplementary Figure 150. ^{13}C NMR of compound **66**.

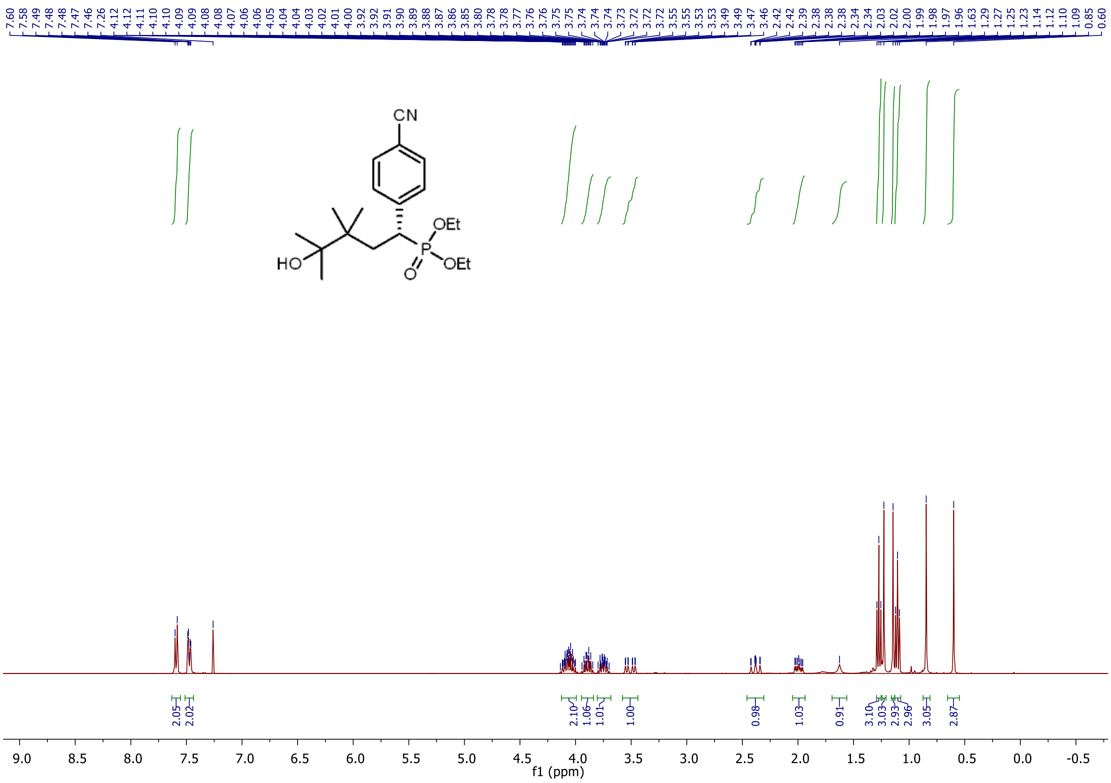

Supplementary Figure 151. ^{1}H NMR of compound **67**.

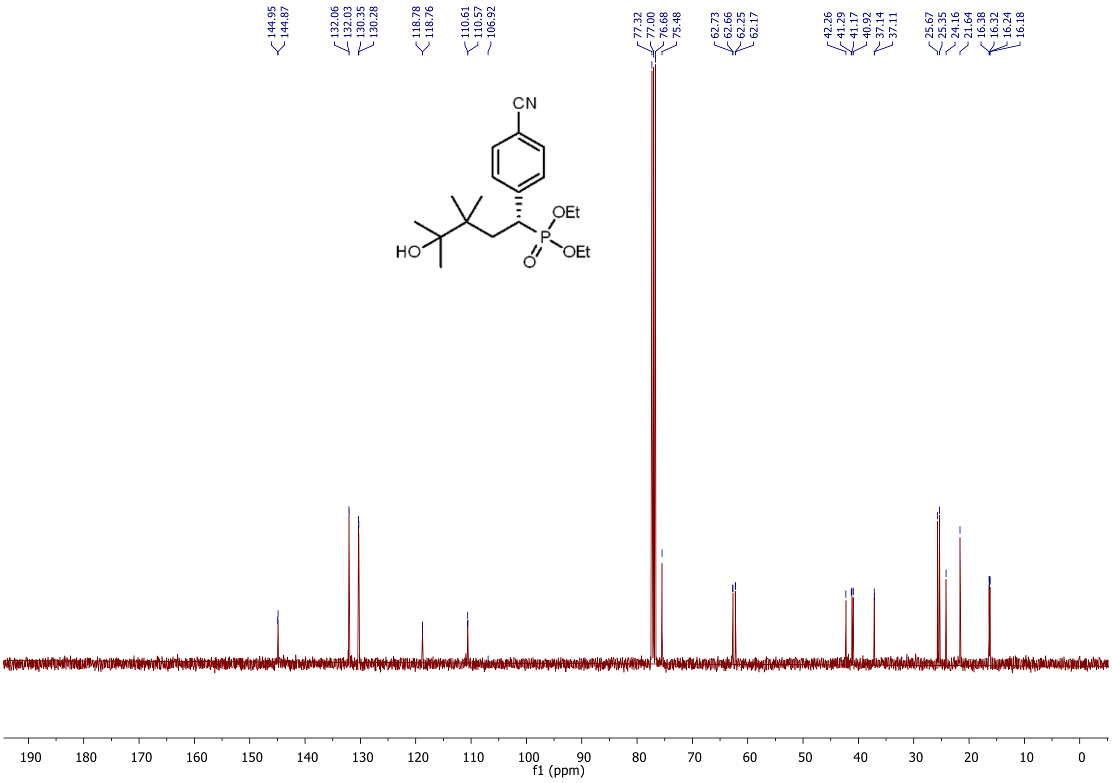

Supplementary Figure 152. ^{13}C NMR of compound **67**.

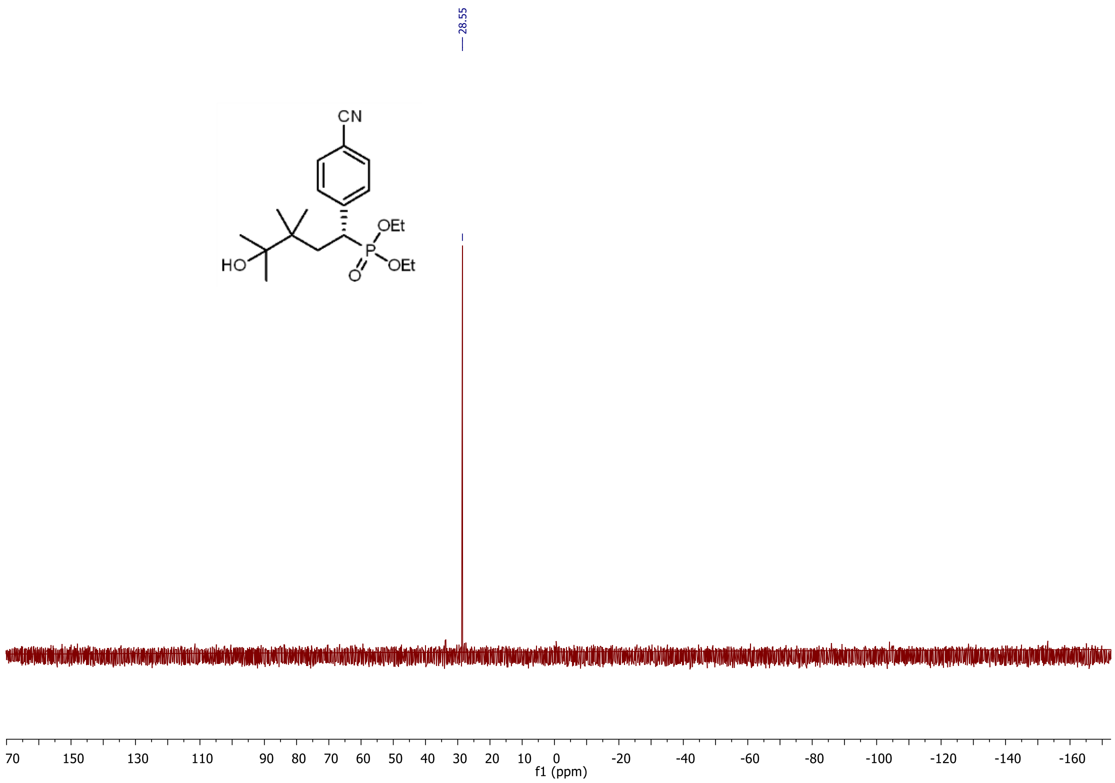

Supplementary Figure 153. ^{31}P NMR of compound **67**.

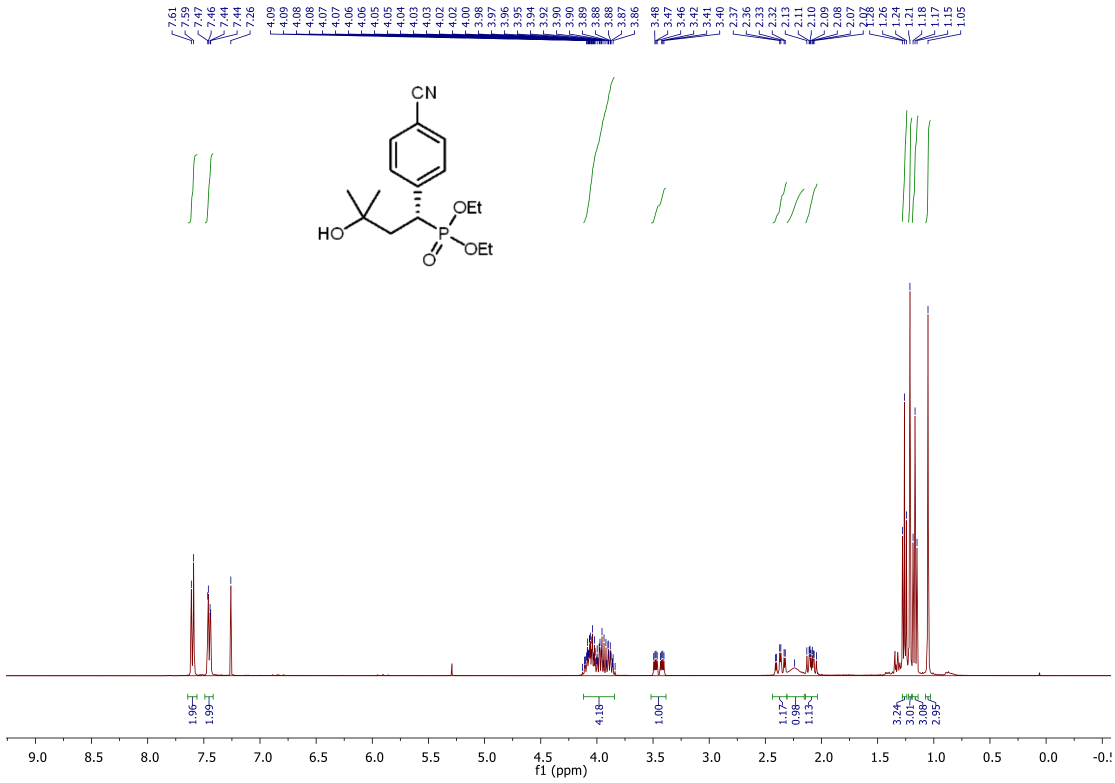

Supplementary Figure 154. ^{1}H NMR of compound **68**.

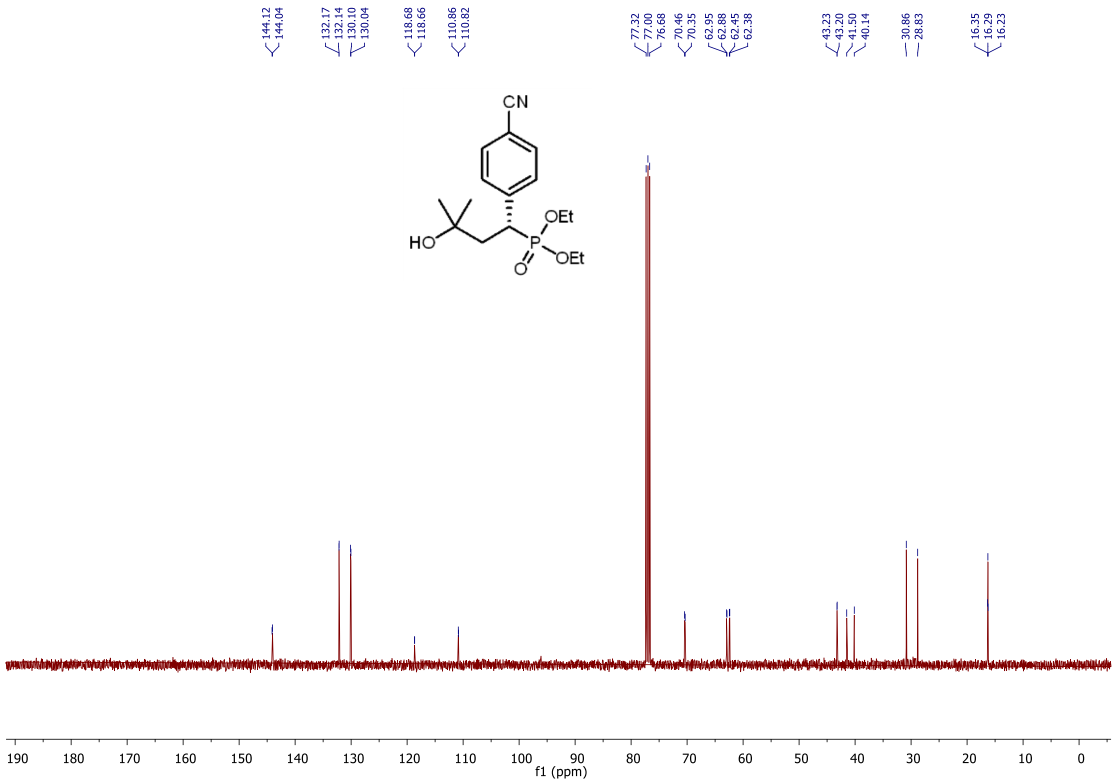

Supplementary Figure 155. ^{13}C NMR of compound **68**.

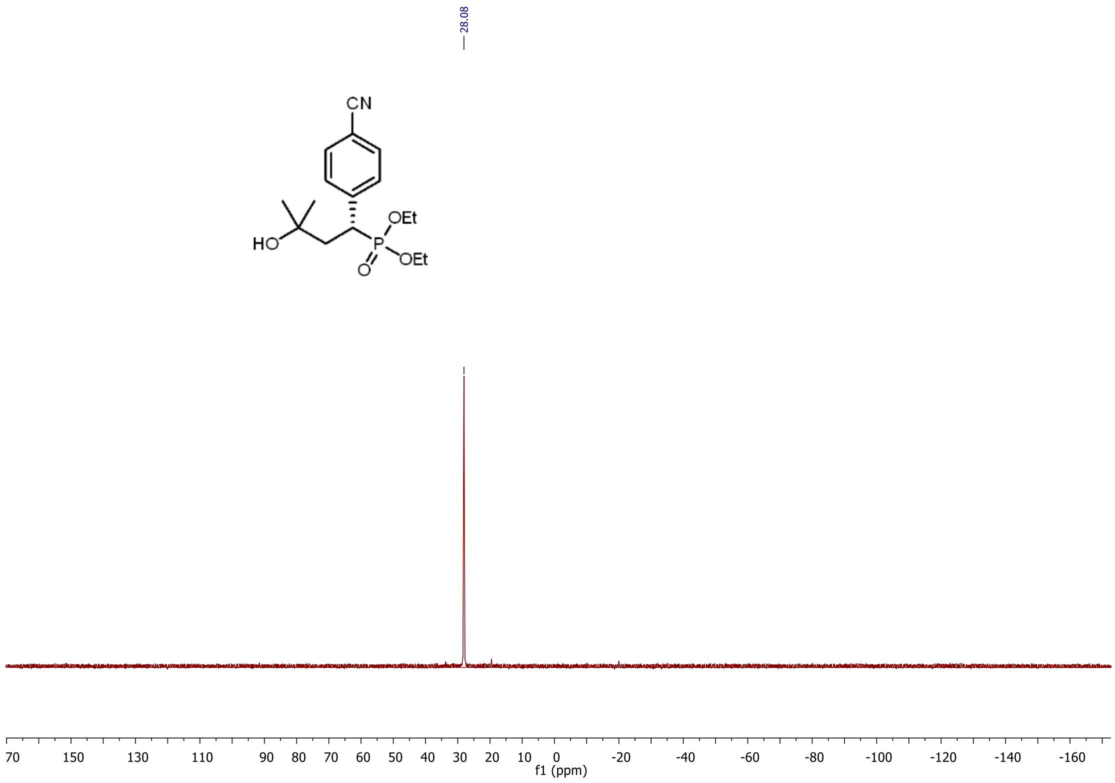

Supplementary Figure 156. ^{31}P NMR of compound **68**.

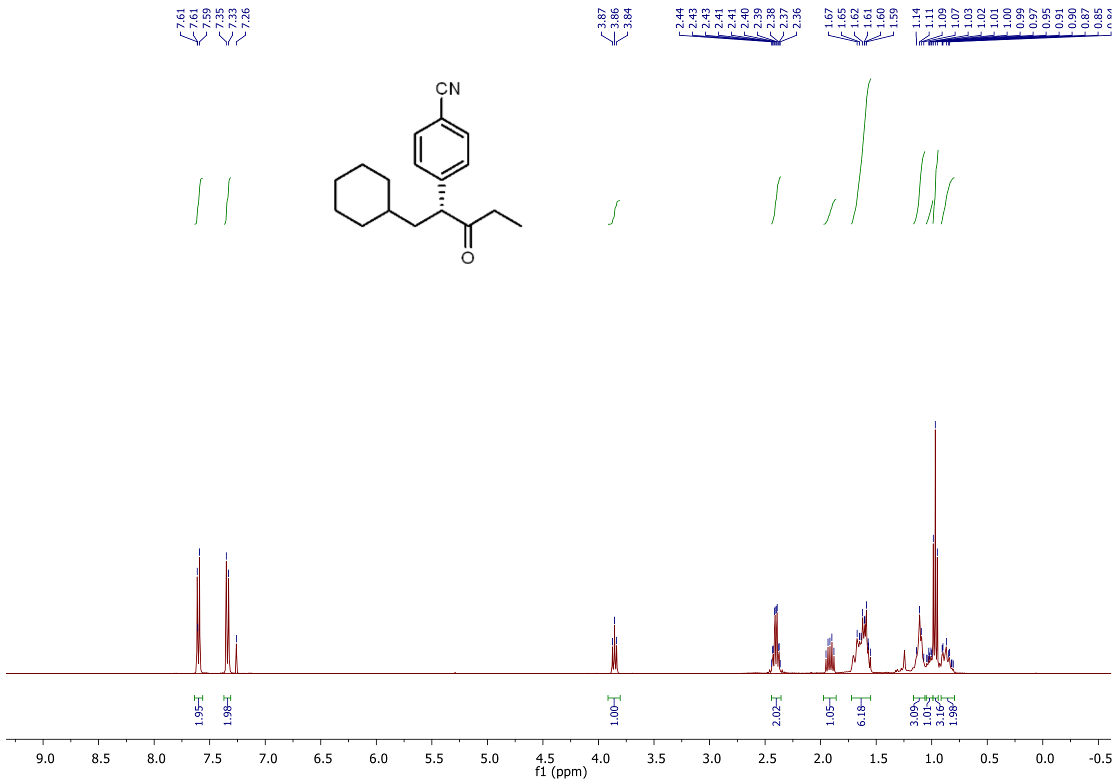

Supplementary Figure 157. ^{1}H NMR of compound **69**.

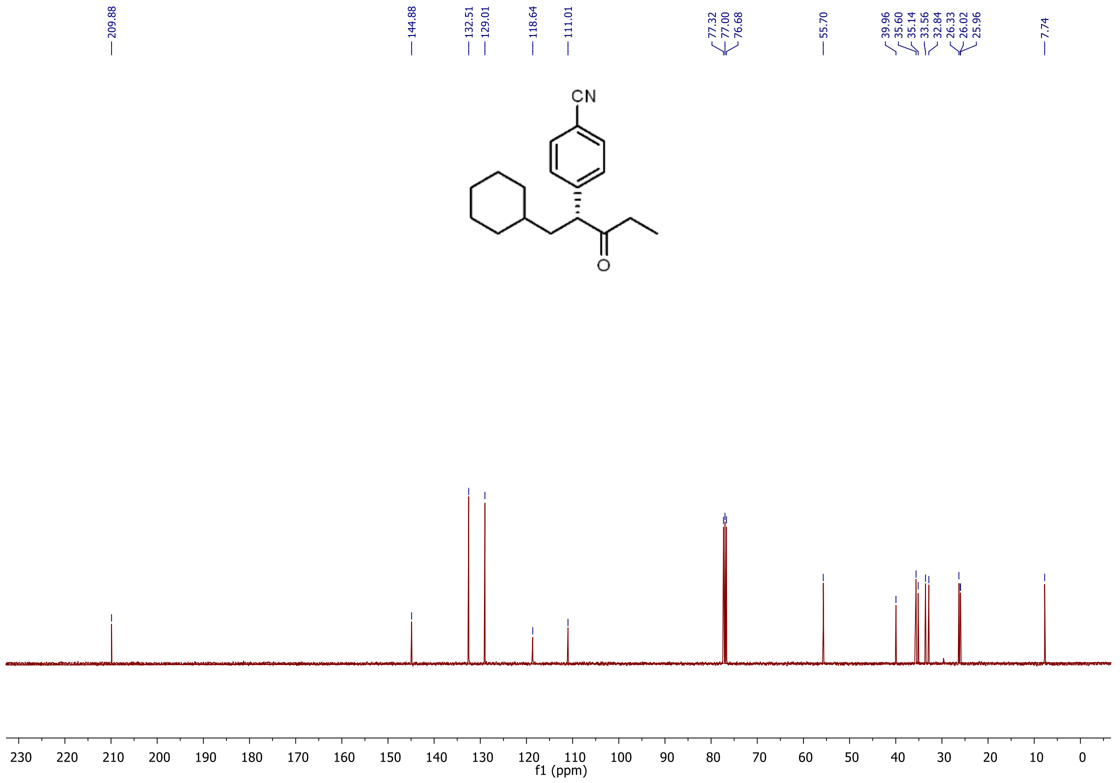

Supplementary Figure 158. ^{13}C NMR of compound **69**.

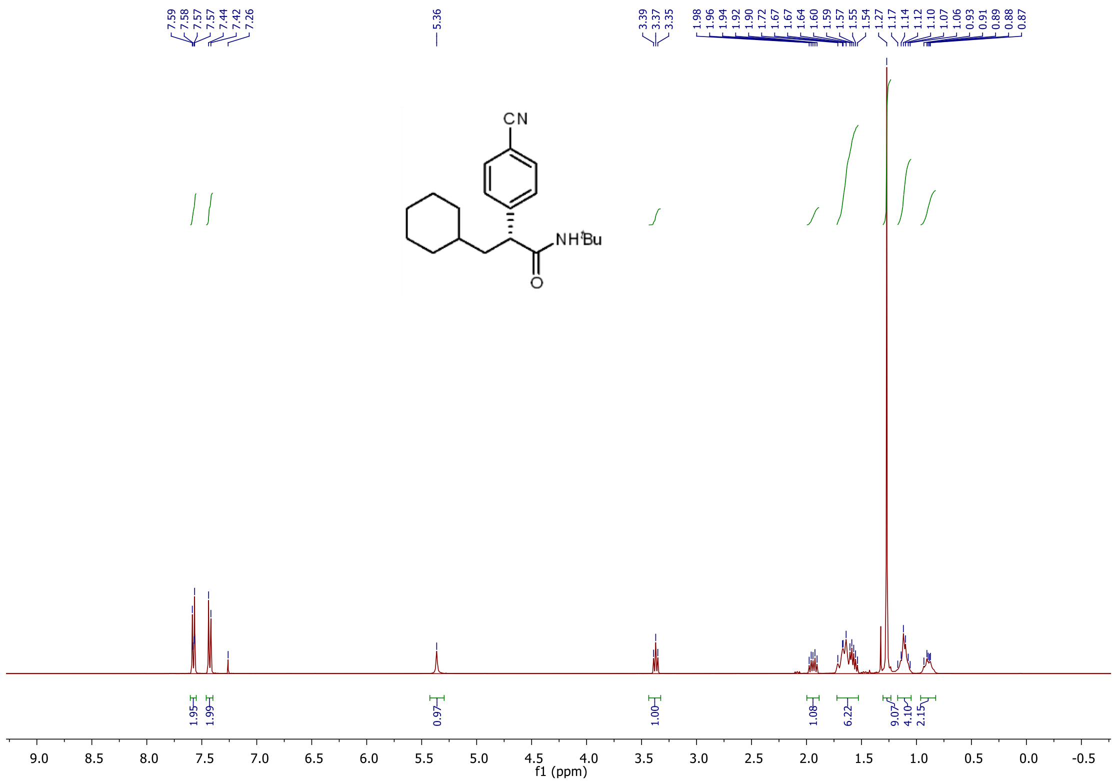

Supplementary Figure 159. ^{1}H NMR of compound **70**.

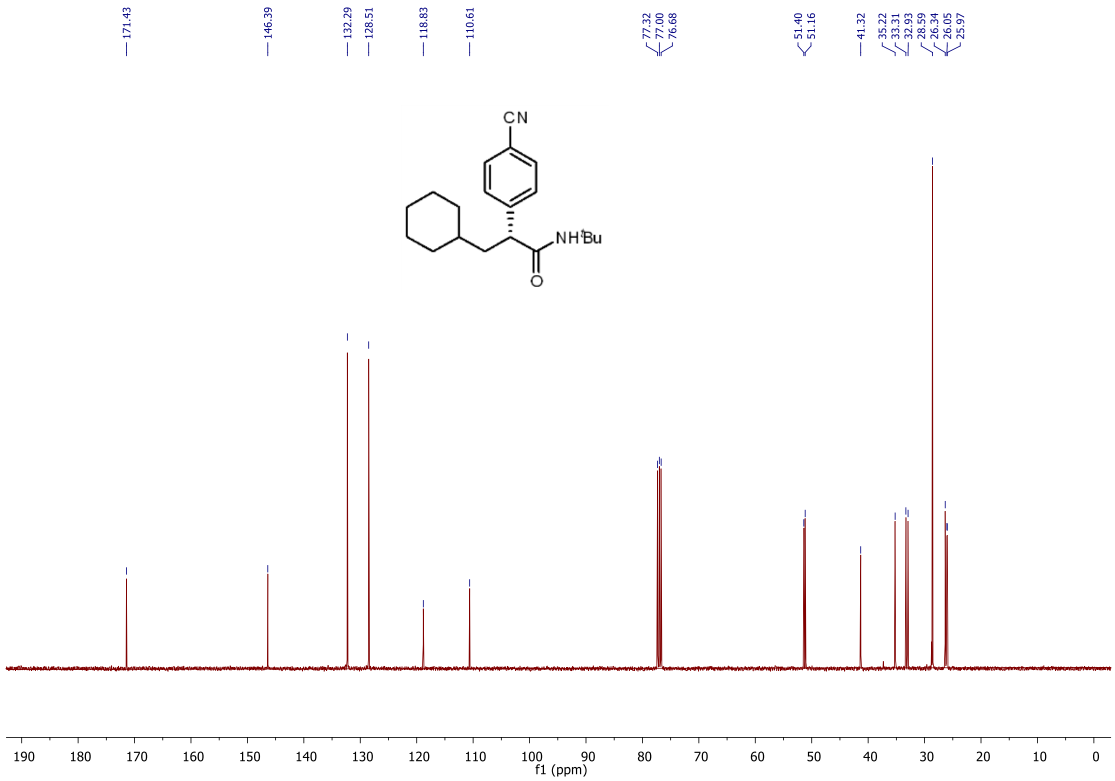

Supplementary Figure 160. ^{13}C NMR of compound **70**.

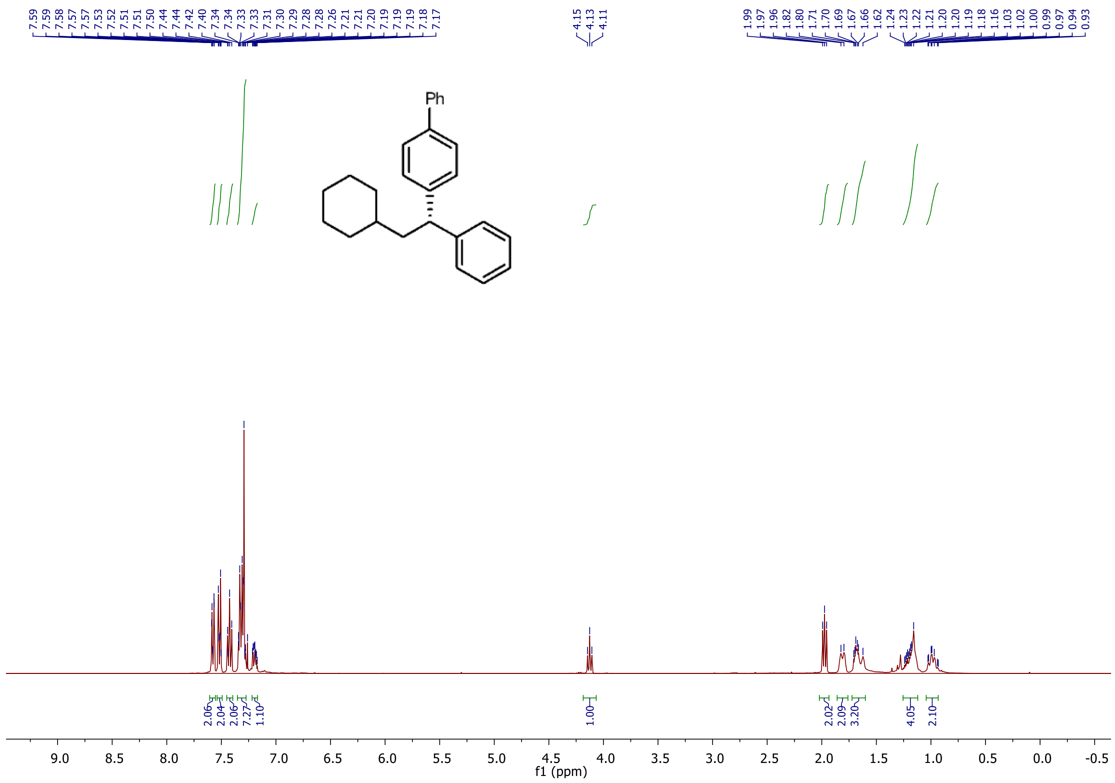

Supplementary Figure 161. ^{1}H NMR of compound **71**.

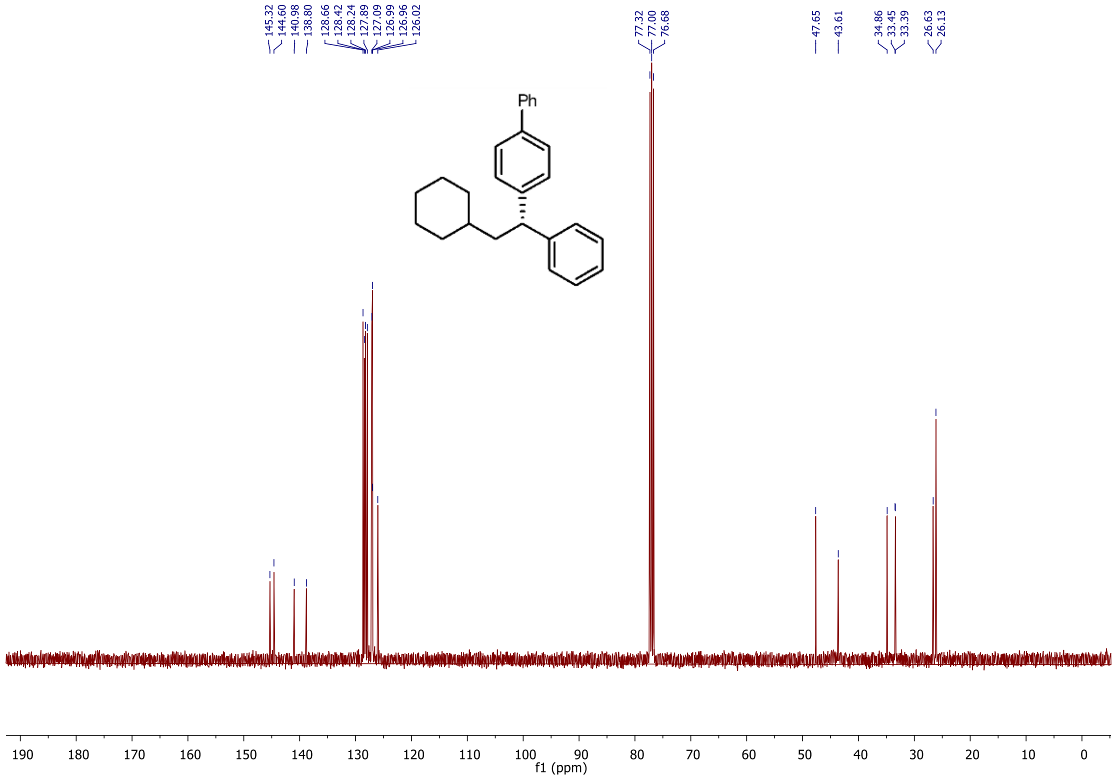

Supplementary Figure 162. ^{13}C NMR of compound **71**.

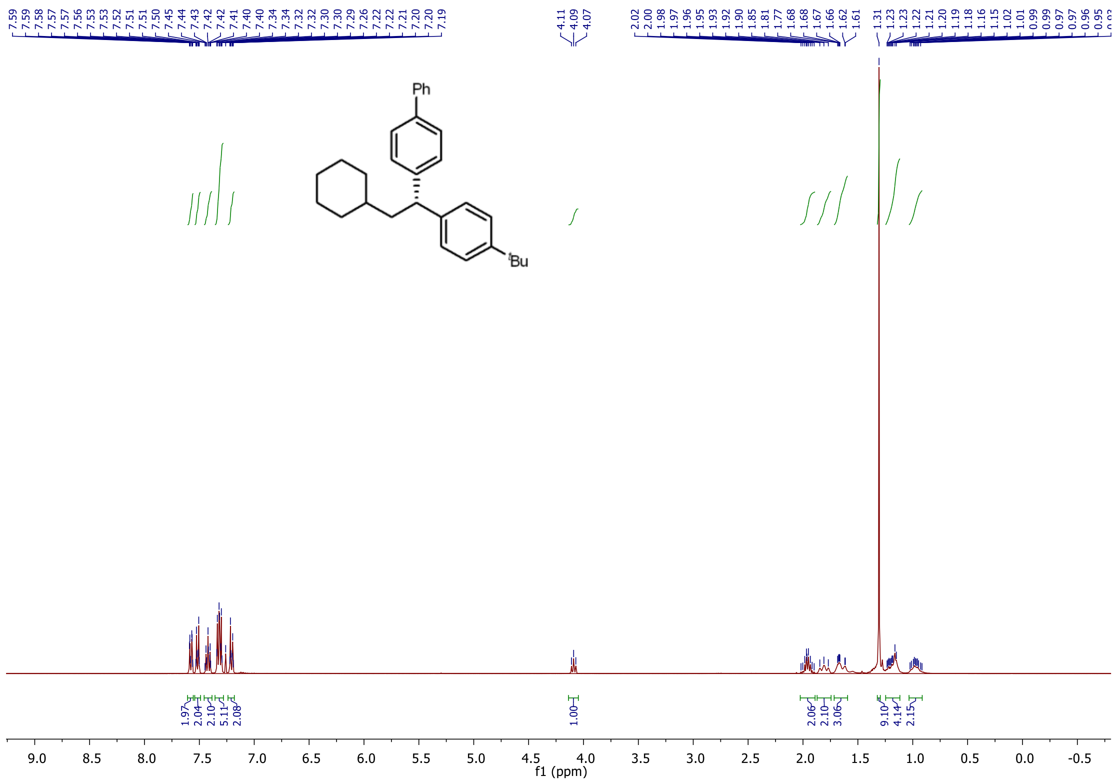

Supplementary Figure 163. ^{1}H NMR of compound **72**.

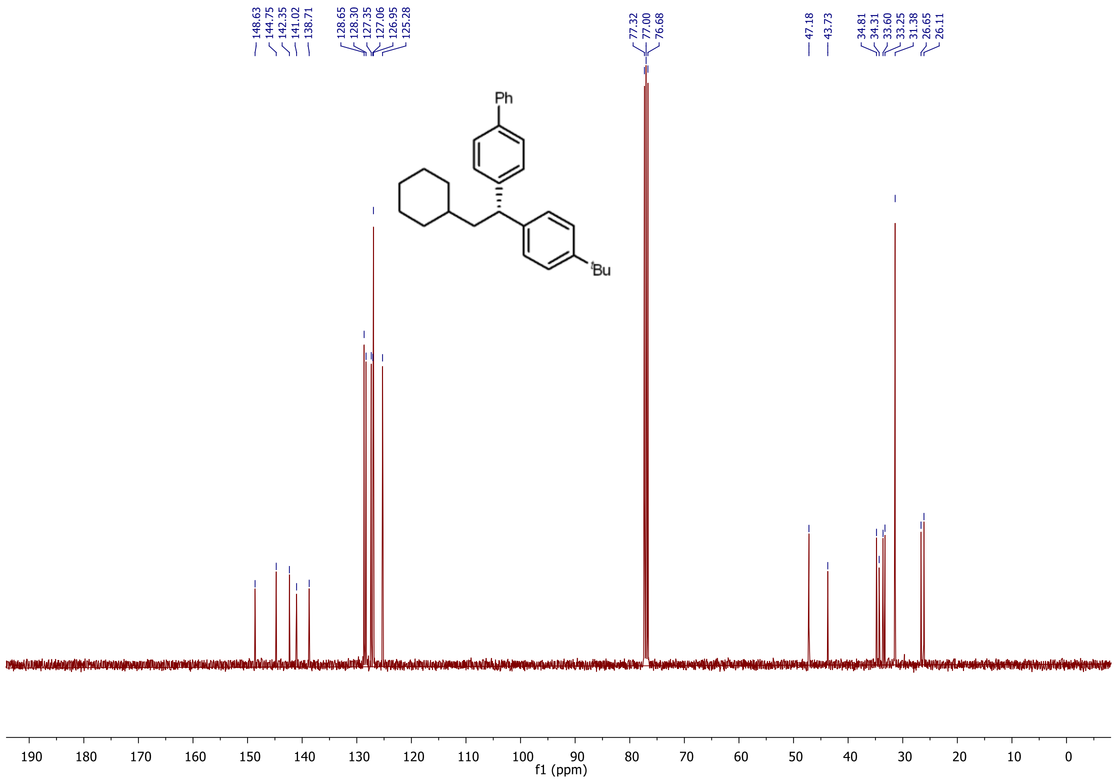

Supplementary Figure 164. ^{13}C NMR of compound **72**.

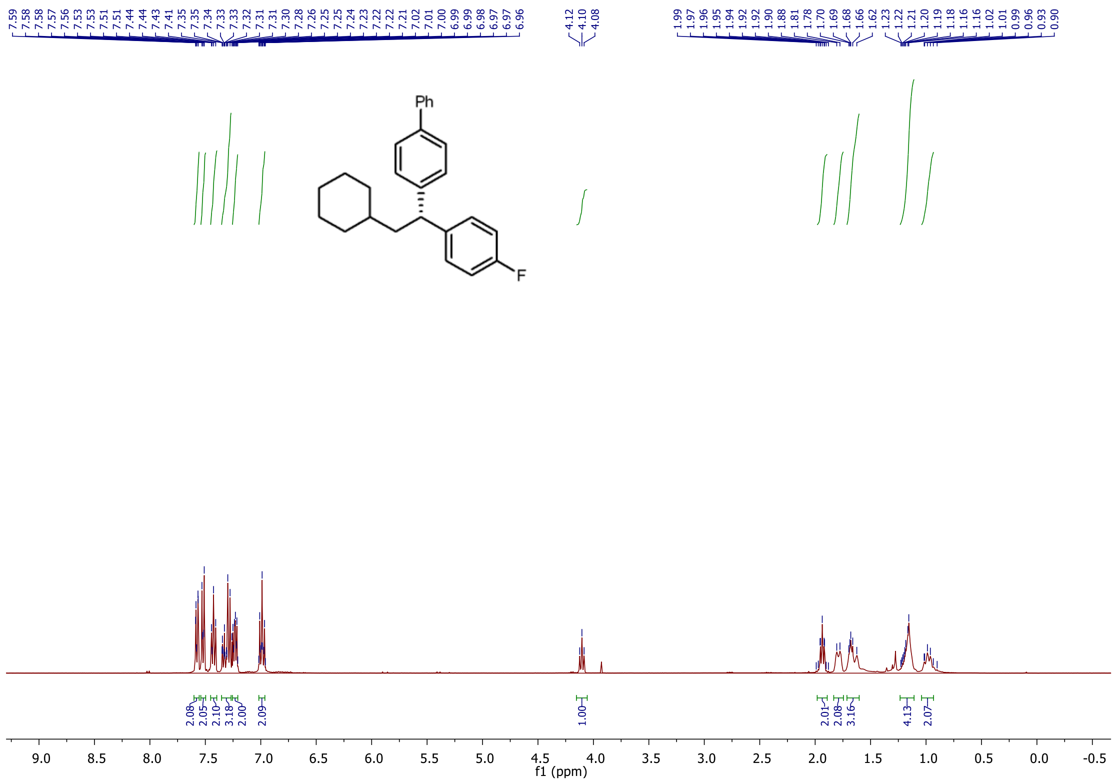

Supplementary Figure 165. ^{1}H NMR of compound **73**.

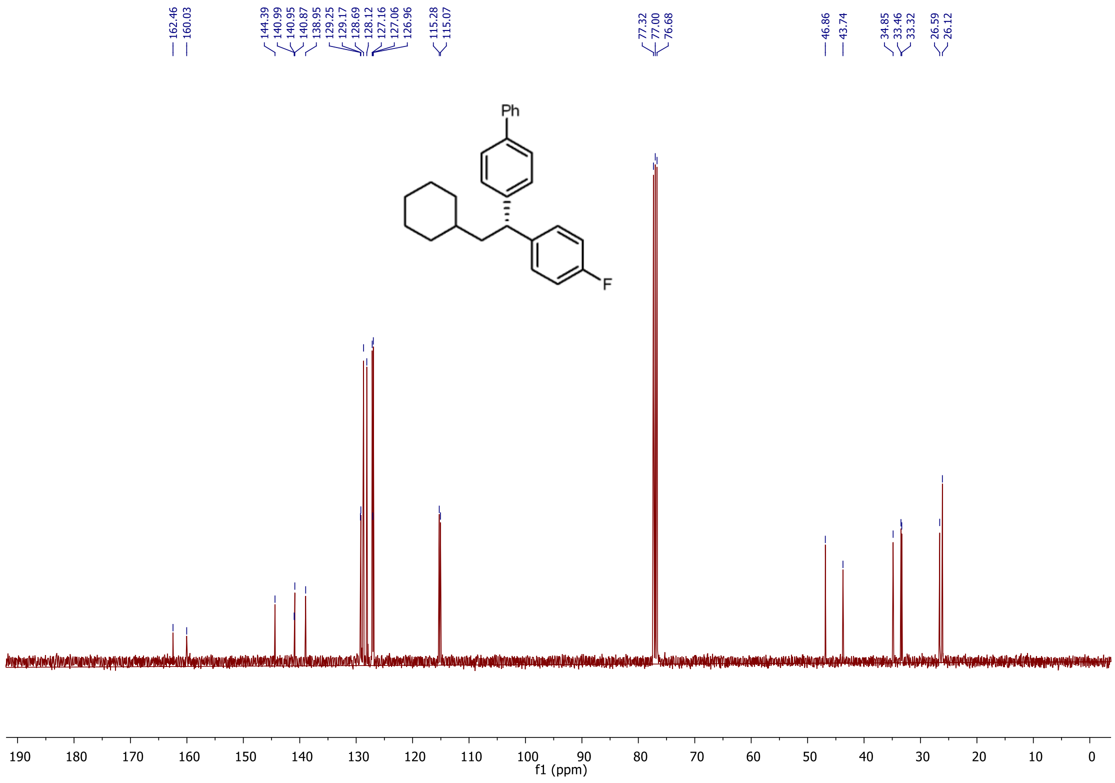

Supplementary Figure 166. ^{13}C NMR of compound **73**.

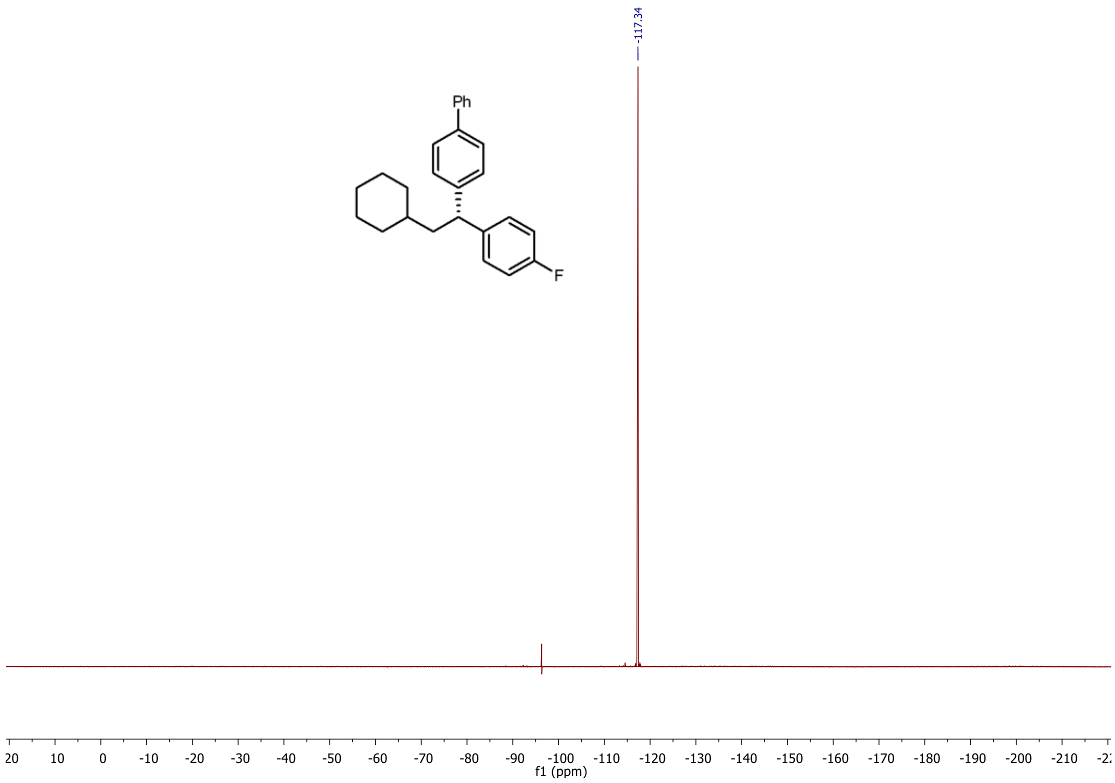

Supplementary Figure 167. ^{19}F NMR of compound **73**.

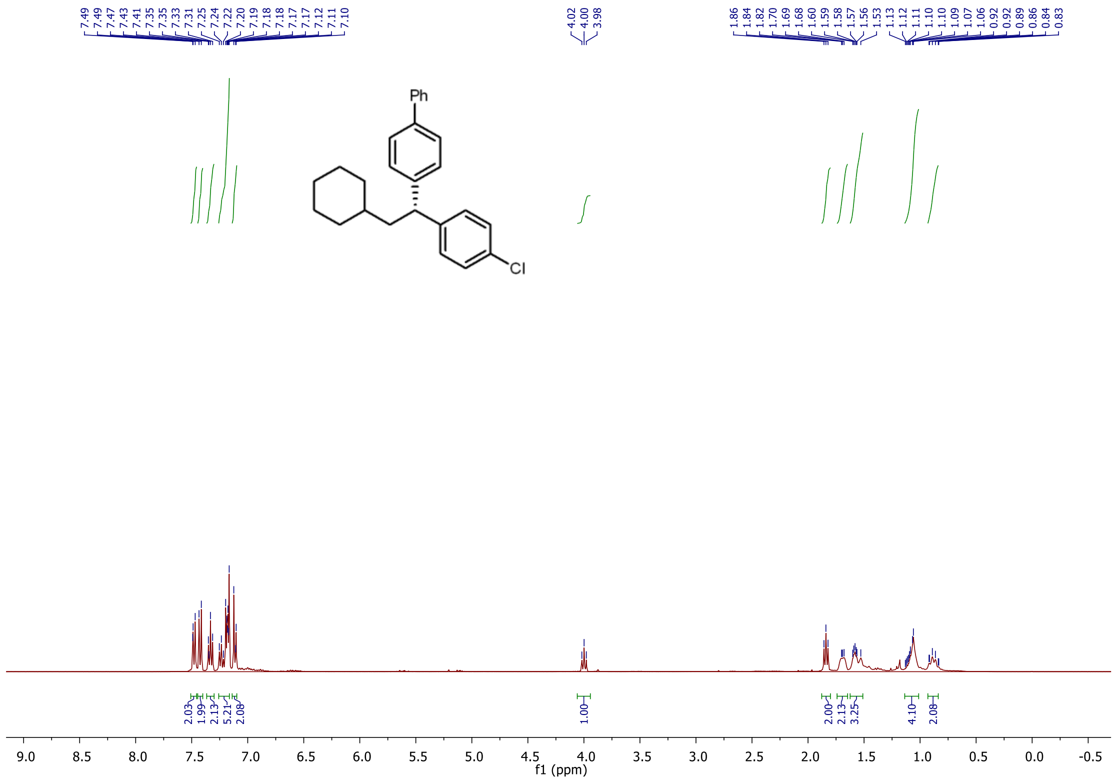

Supplementary Figure 168. ^{1}H NMR of compound **74**.

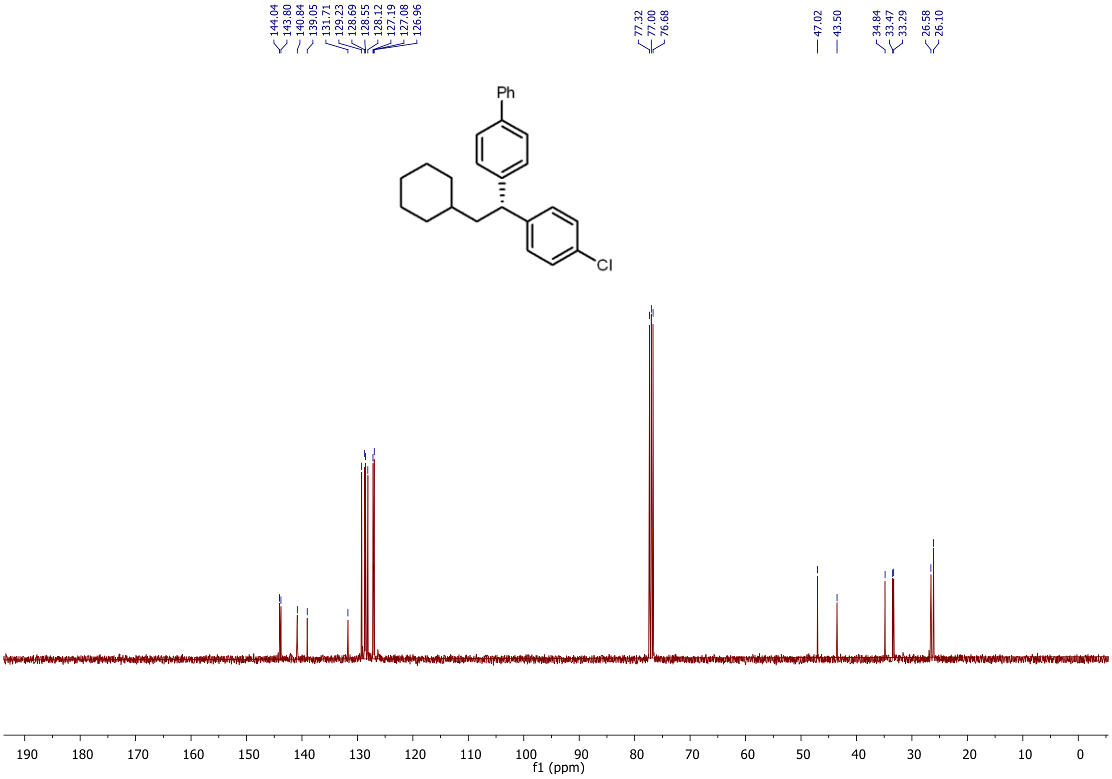

Supplementary Figure 169. ^{13}C NMR of compound **74**.

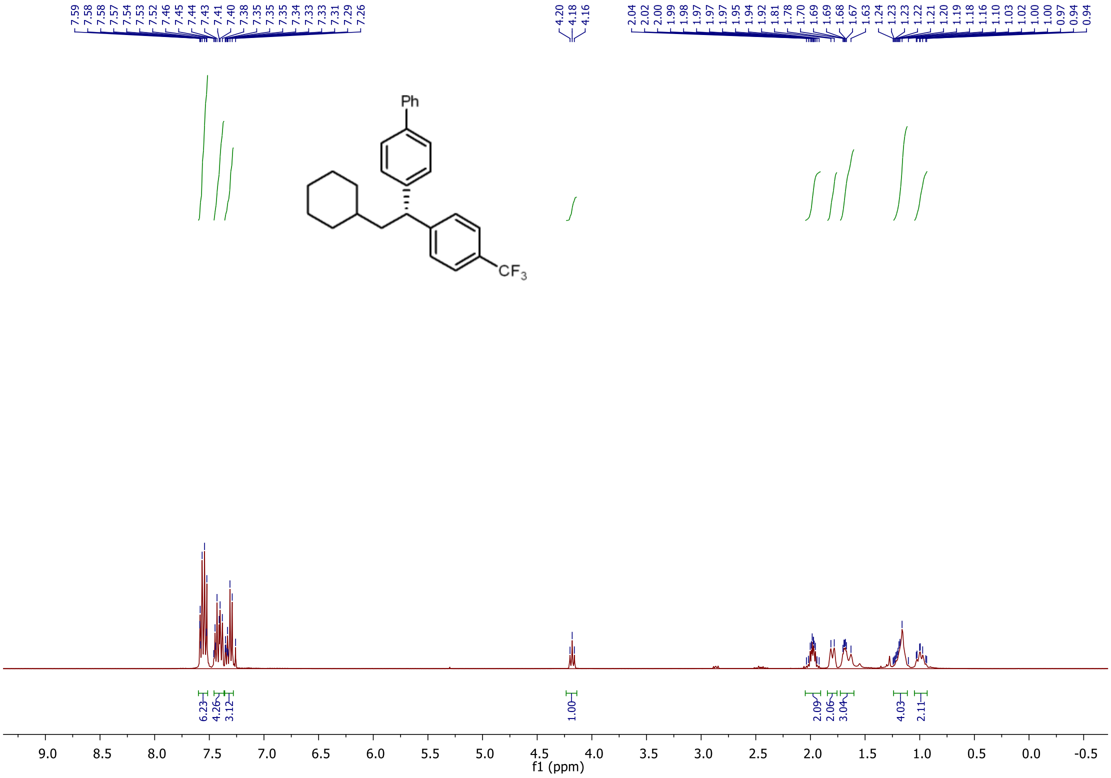

Supplementary Figure 170. ^{1}H NMR of compound **75**.

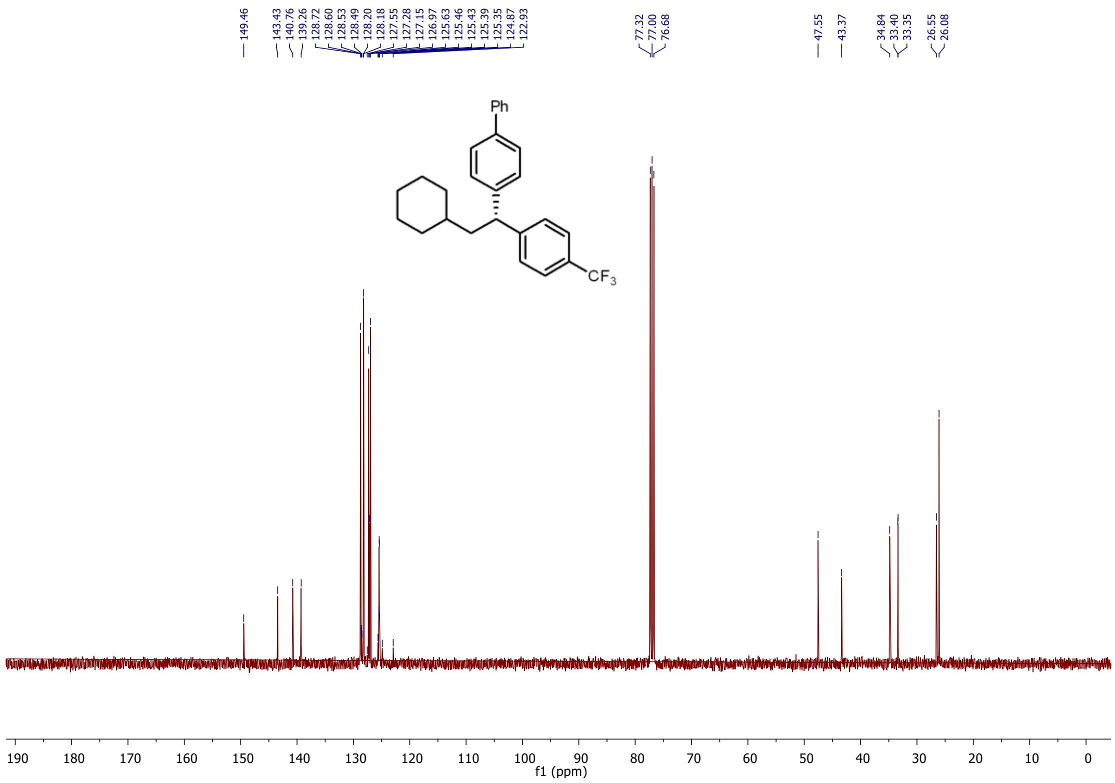

Supplementary Figure 171. ^{13}C NMR of compound **75**.

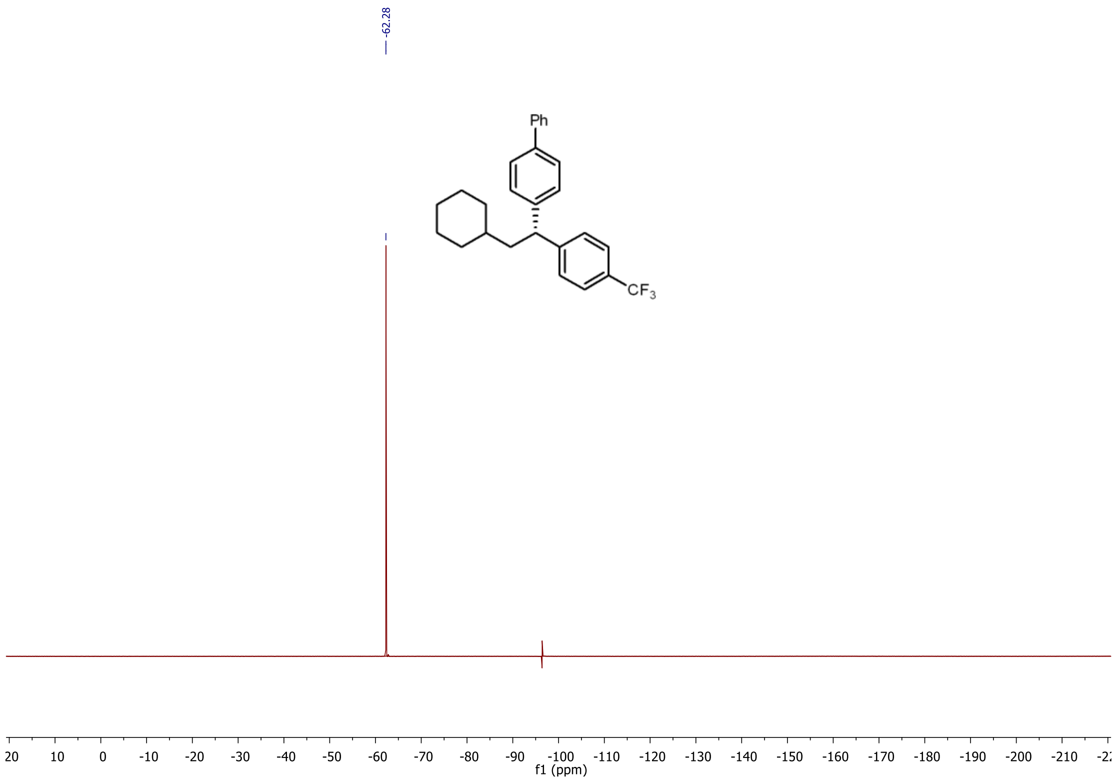

Supplementary Figure 172. ^{19}F NMR of compound **75**.

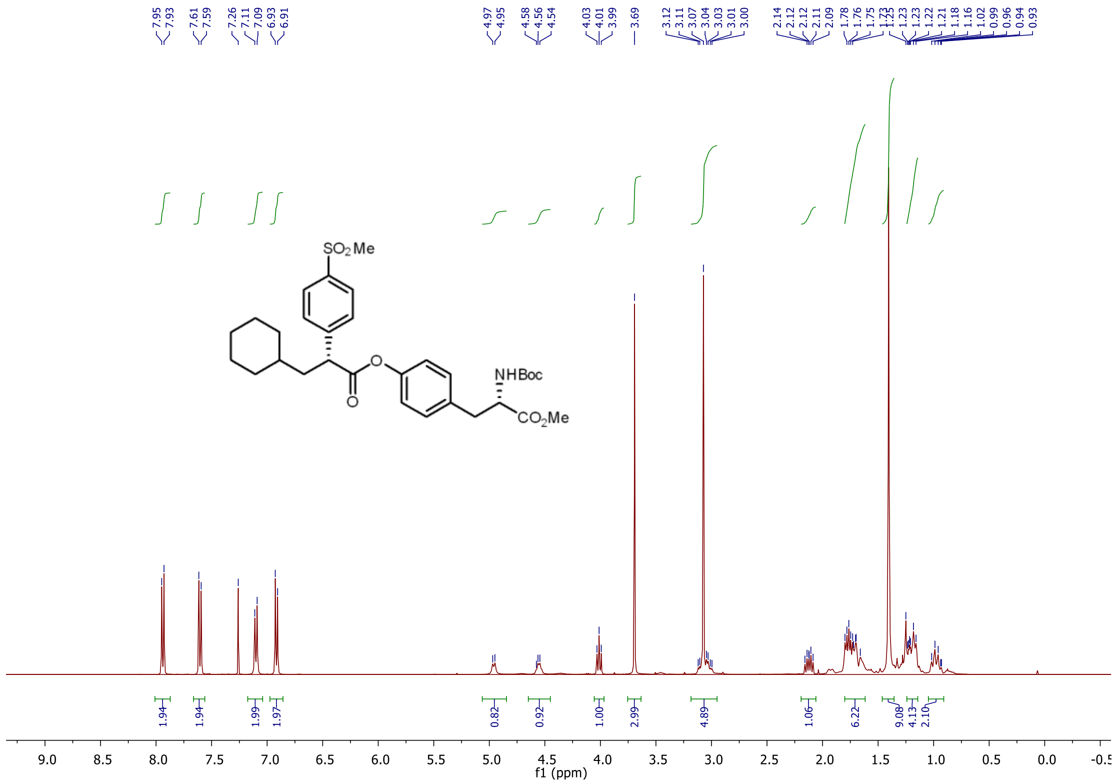

Supplementary Figure 173. ^{1}H NMR of compound **76**.

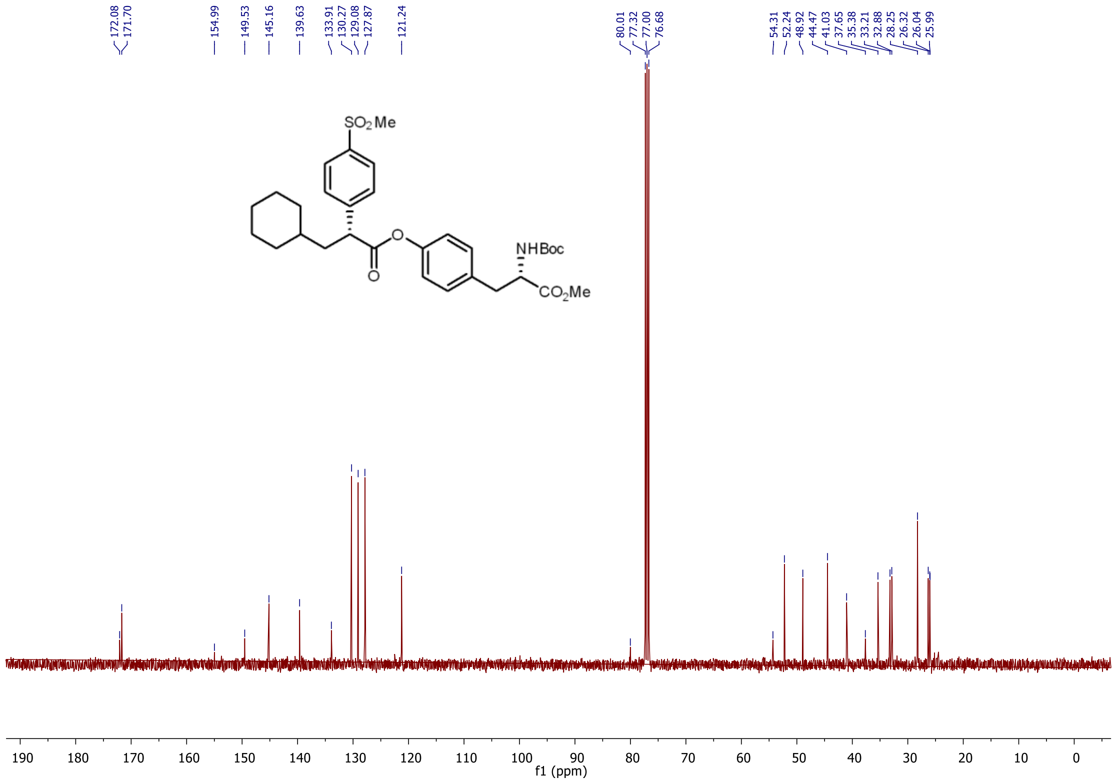

Supplementary Figure 174. ^{13}C NMR of compound **76**.

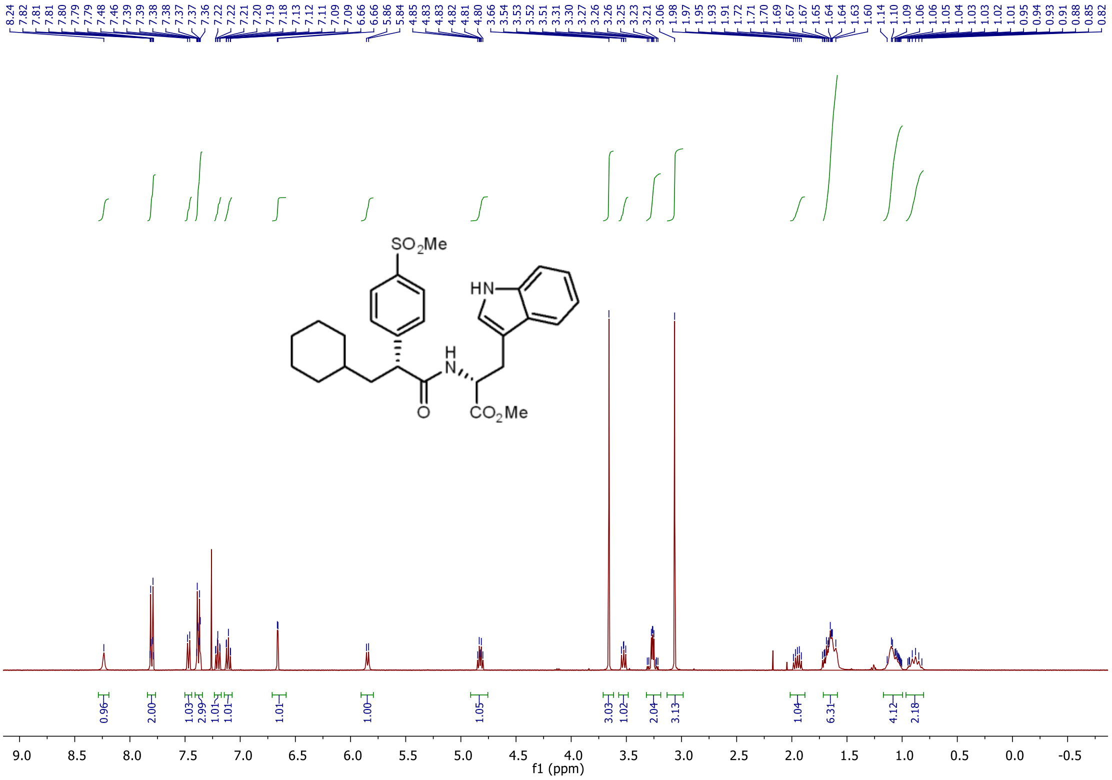

Supplementary Figure 175. ^{1}H NMR of compound **77**.

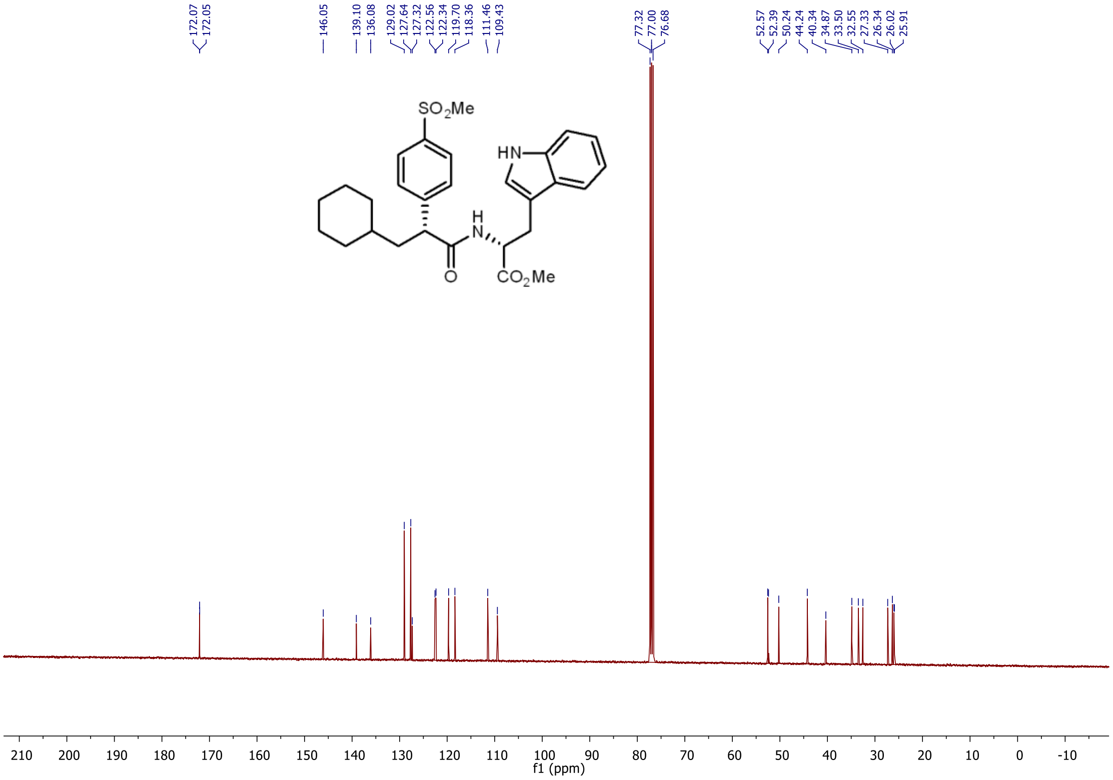

Supplementary Figure 176. ^{13}C NMR of compound **77**.

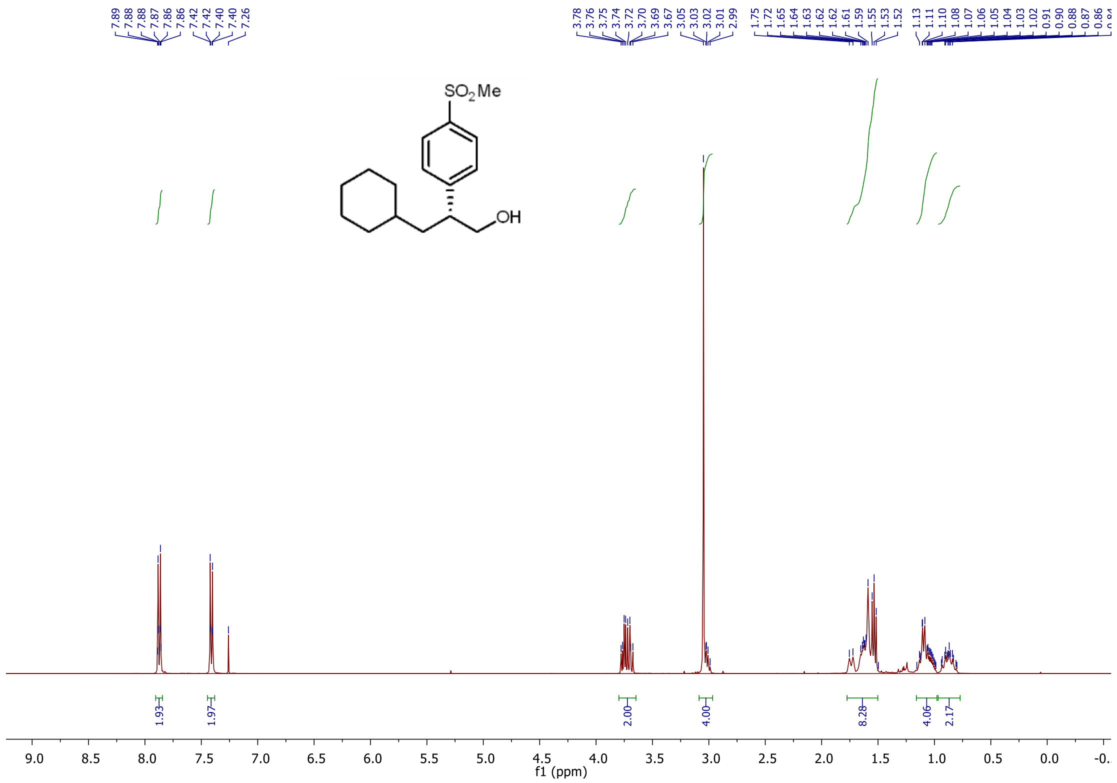

Supplementary Figure 177. ^{1}H NMR of compound **78**.

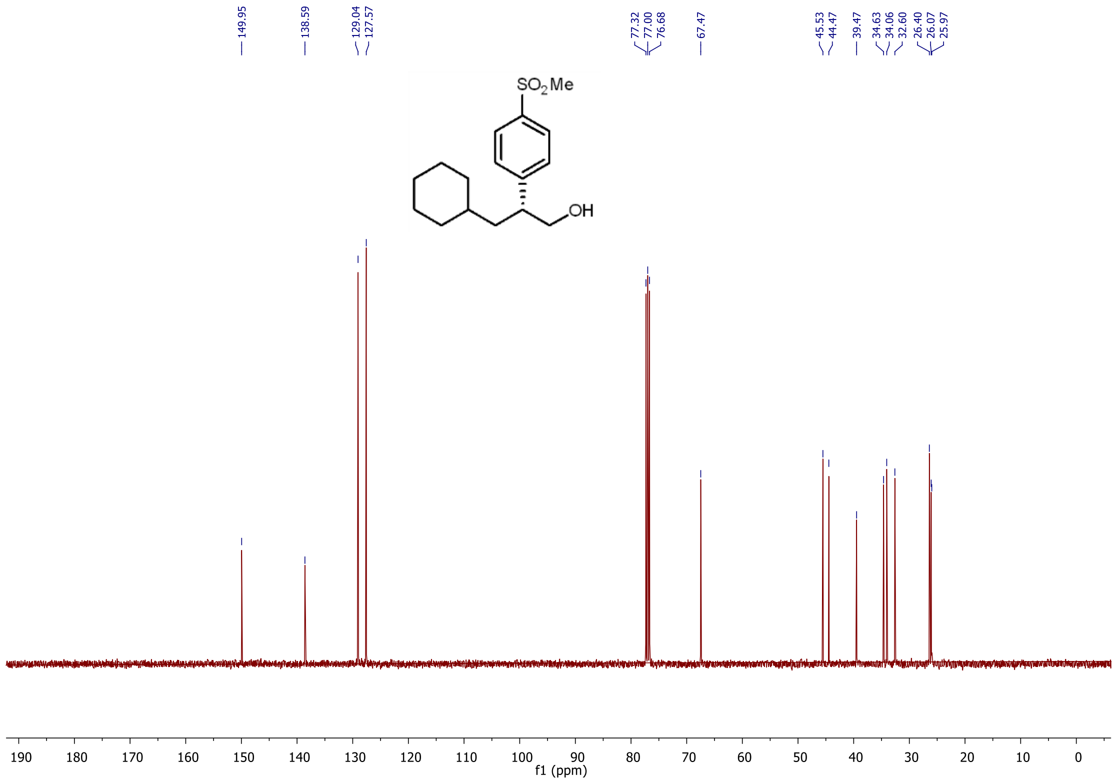

Supplementary Figure 178. ^{13}C NMR of compound **78**.

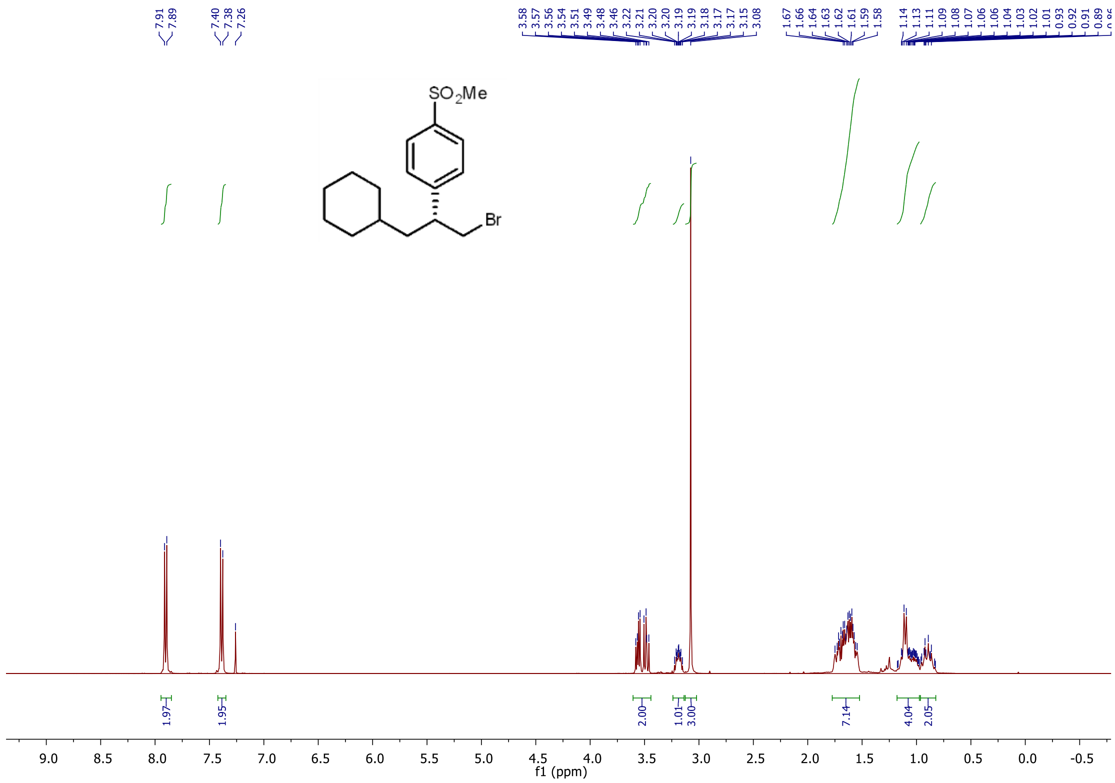

Supplementary Figure 179. ^{1}H NMR of compound **79**.

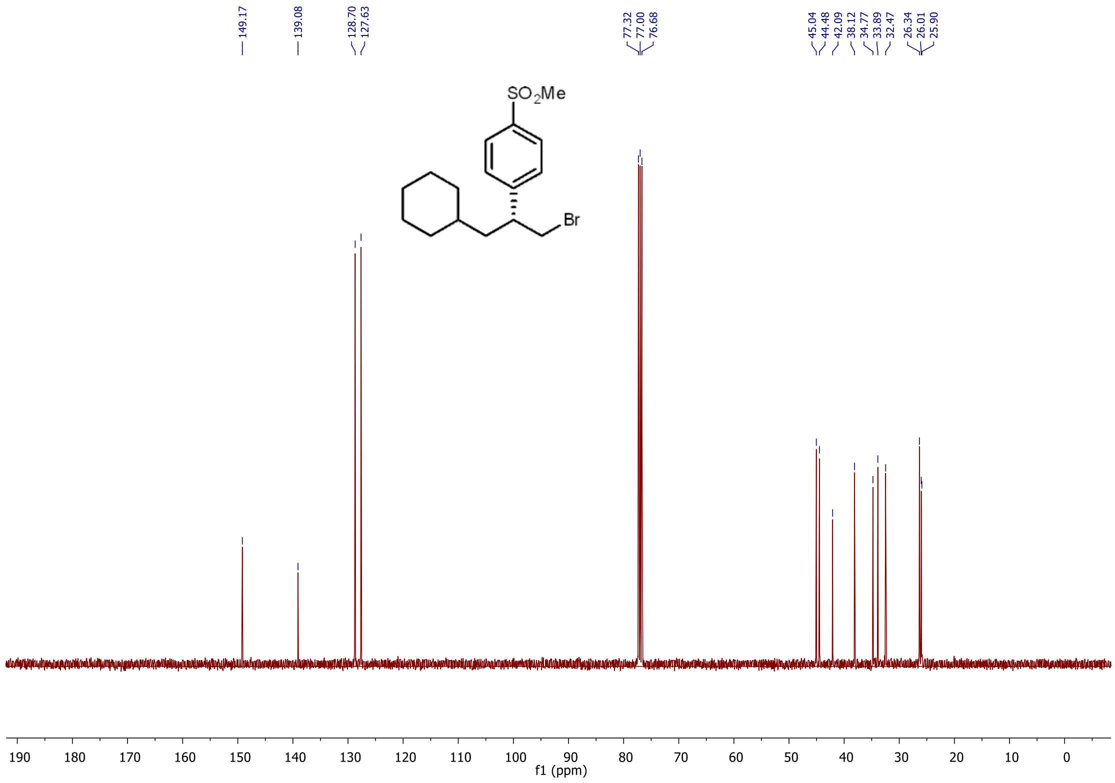

Supplementary Figure 180. ^{13}C NMR of compound **79**.

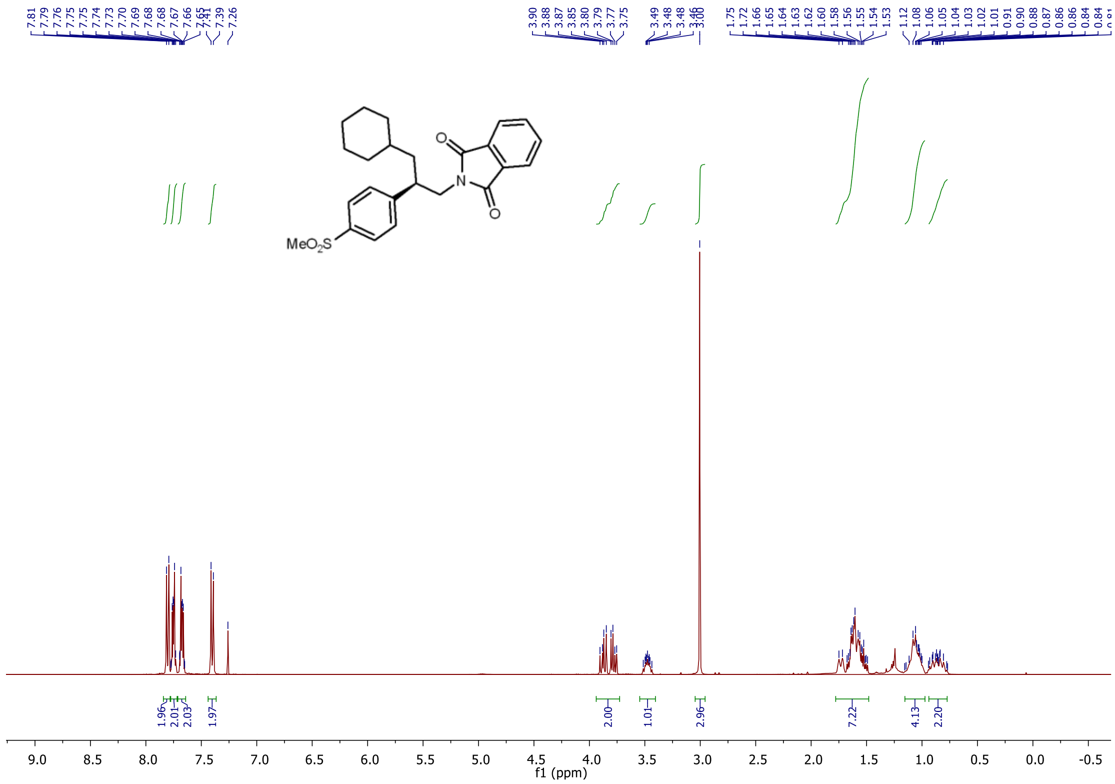

Supplementary Figure 181. ^{1}H NMR of compound **80**.

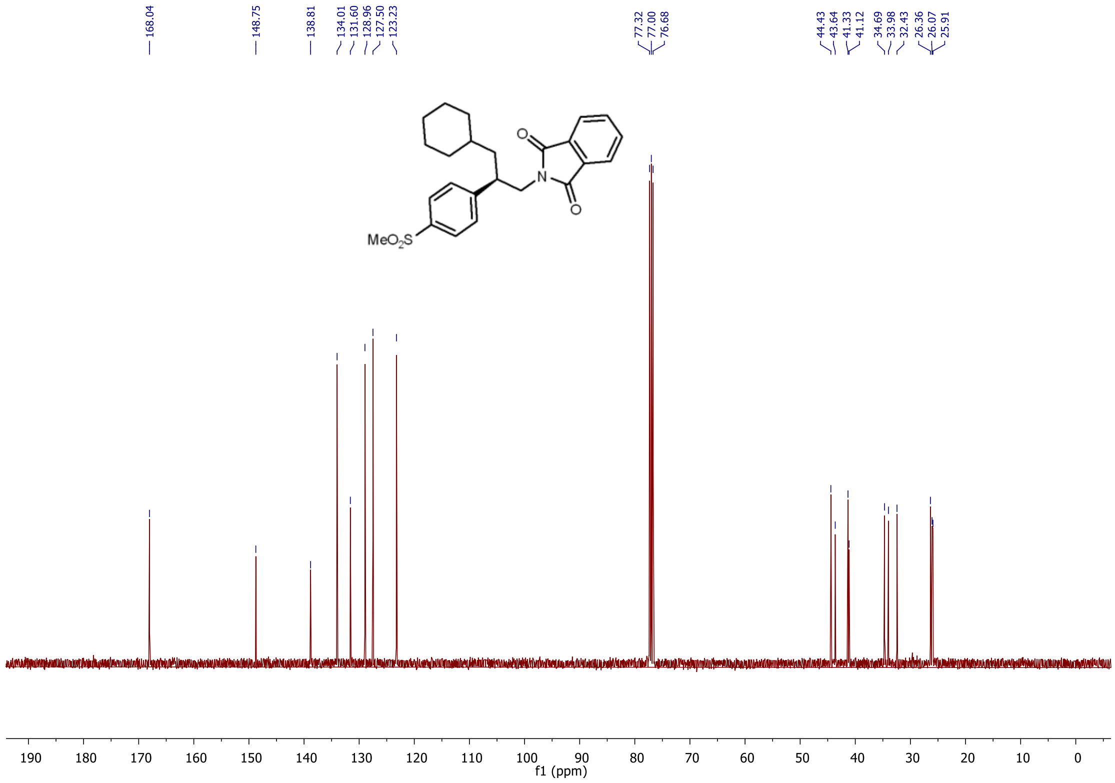

Supplementary Figure 182. ^{13}C NMR of compound **80**.

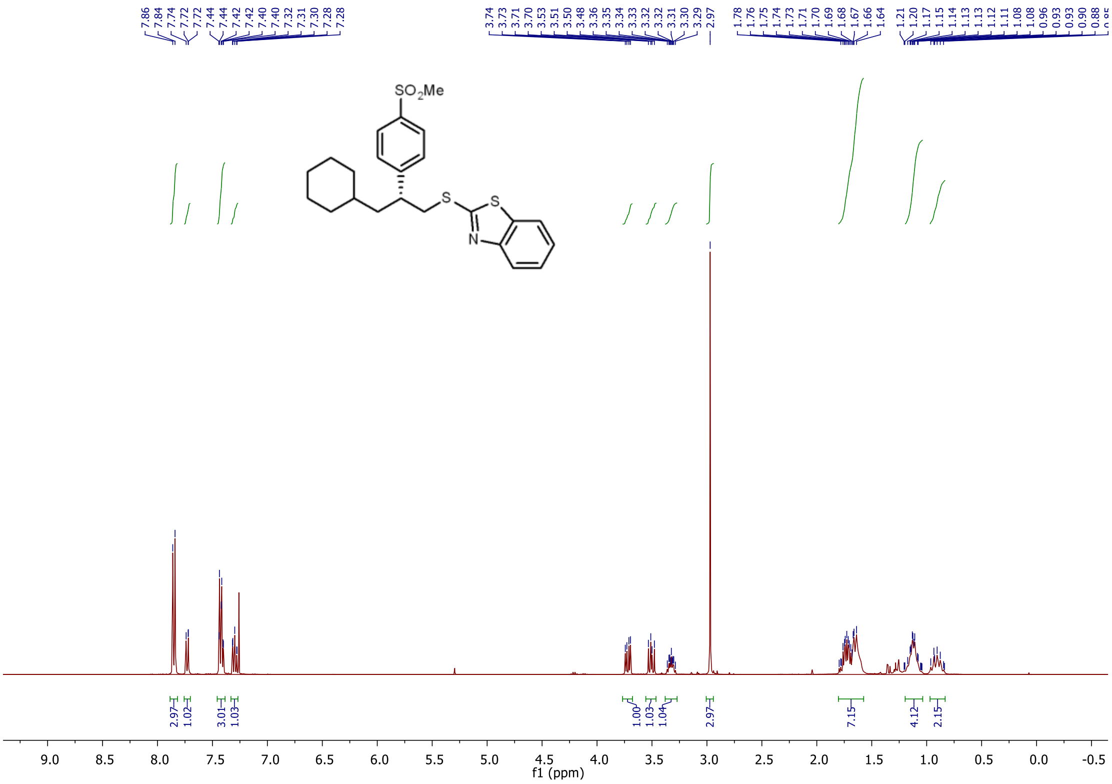

Supplementary Figure 183. ^{1}H NMR of compound **81**.

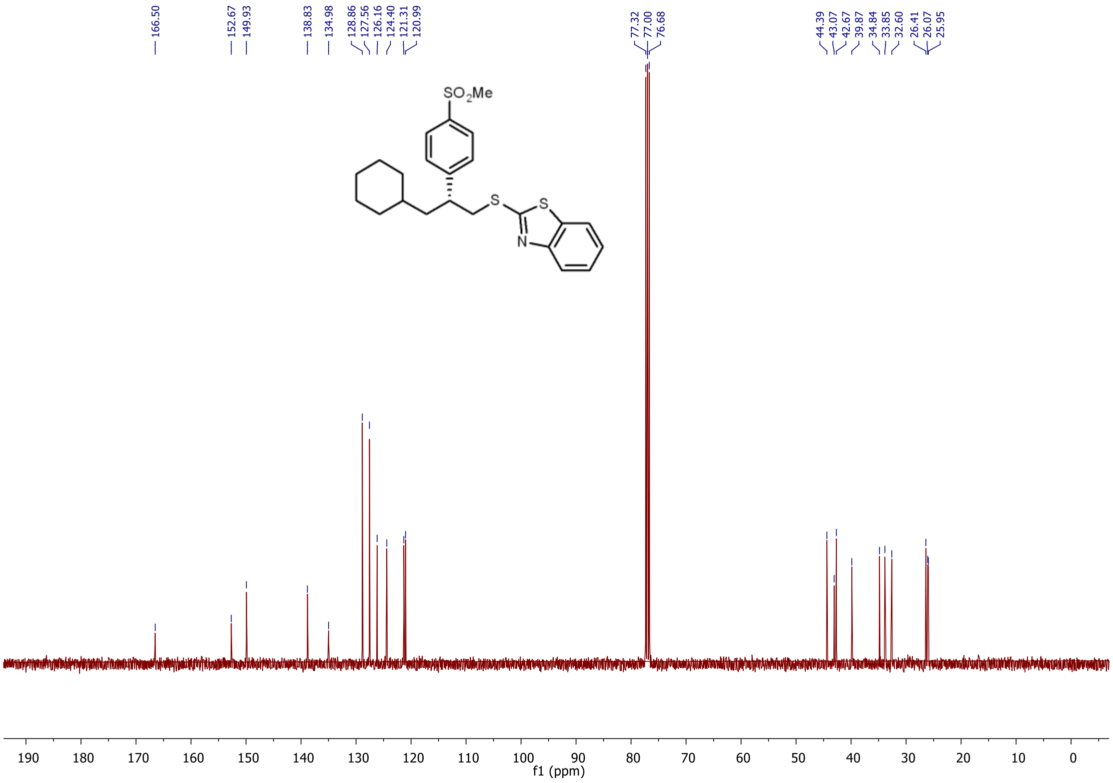

Supplementary Figure 184. ^{13}C NMR of compound **81**.

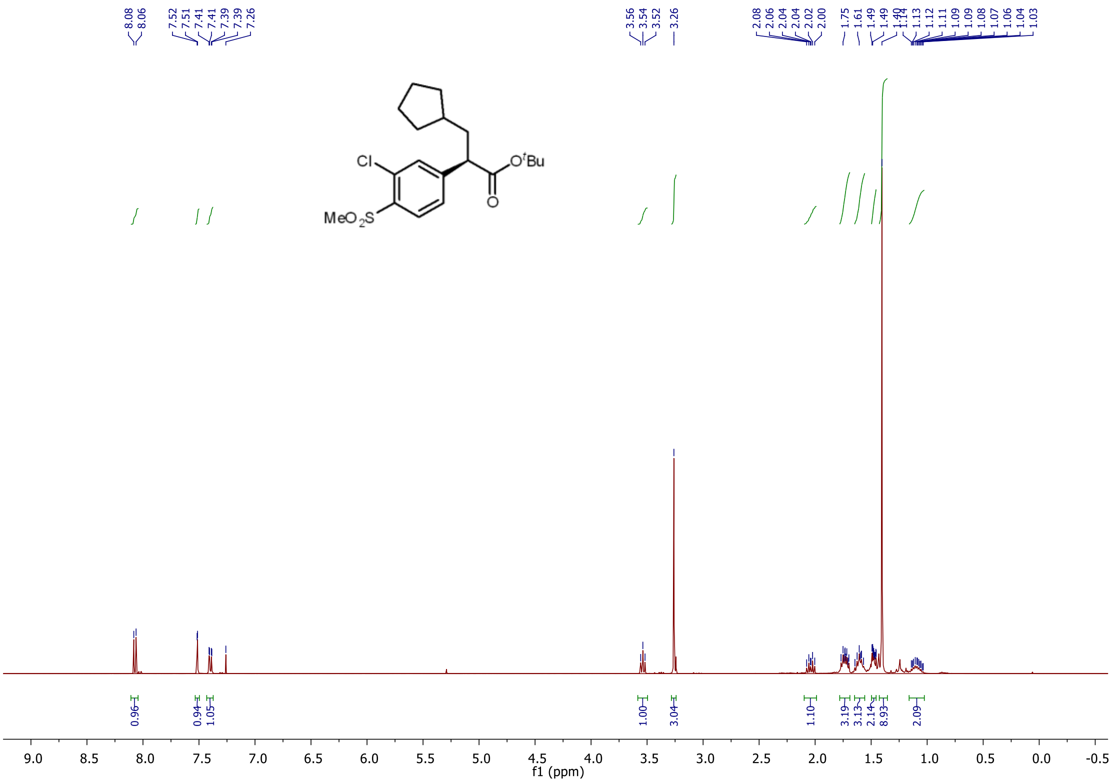

Supplementary Figure 185. ^{1}H NMR of compound **82**.

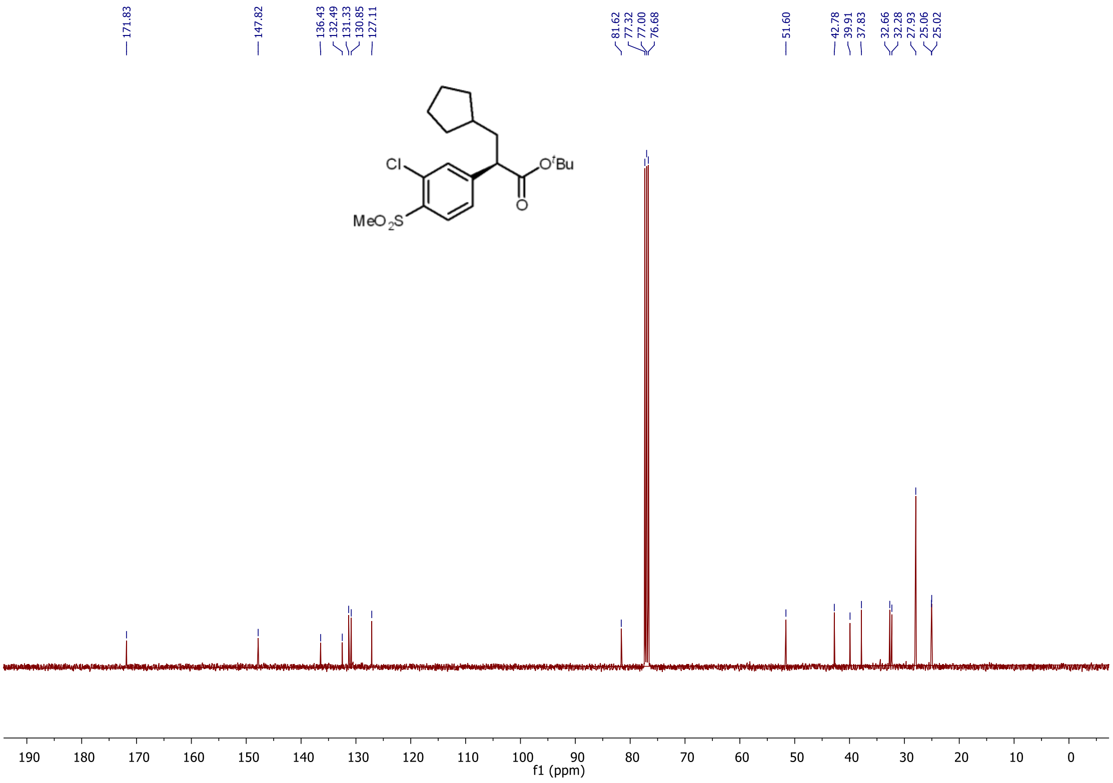

Supplementary Figure 186. ^{13}C NMR of compound **82**.

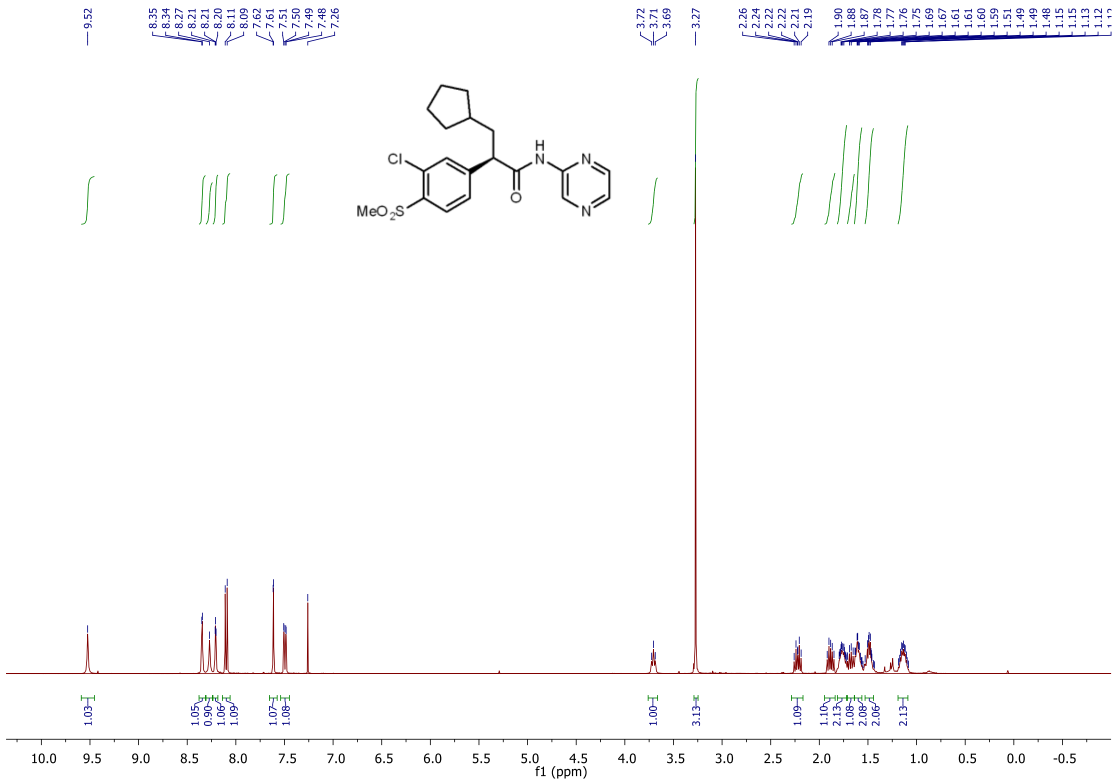

Supplementary Figure 187. ^{1}H NMR of compound **83**.

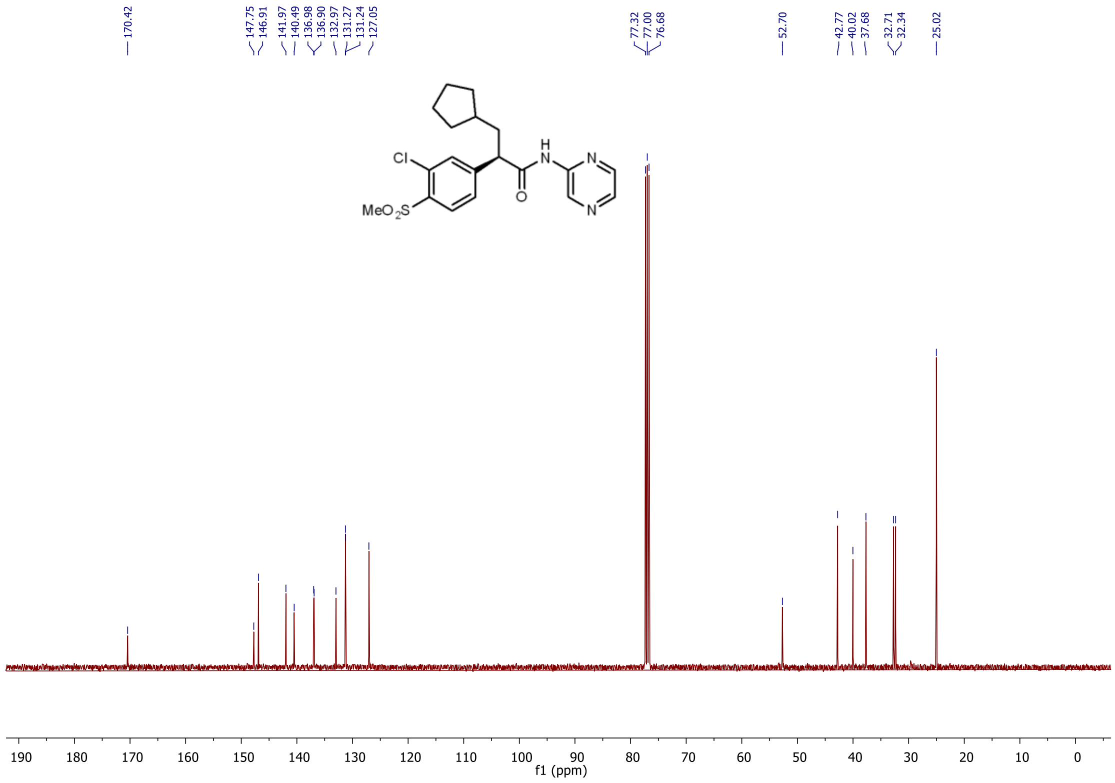

Supplementary Figure 188. ^{13}C NMR of compound **83**.

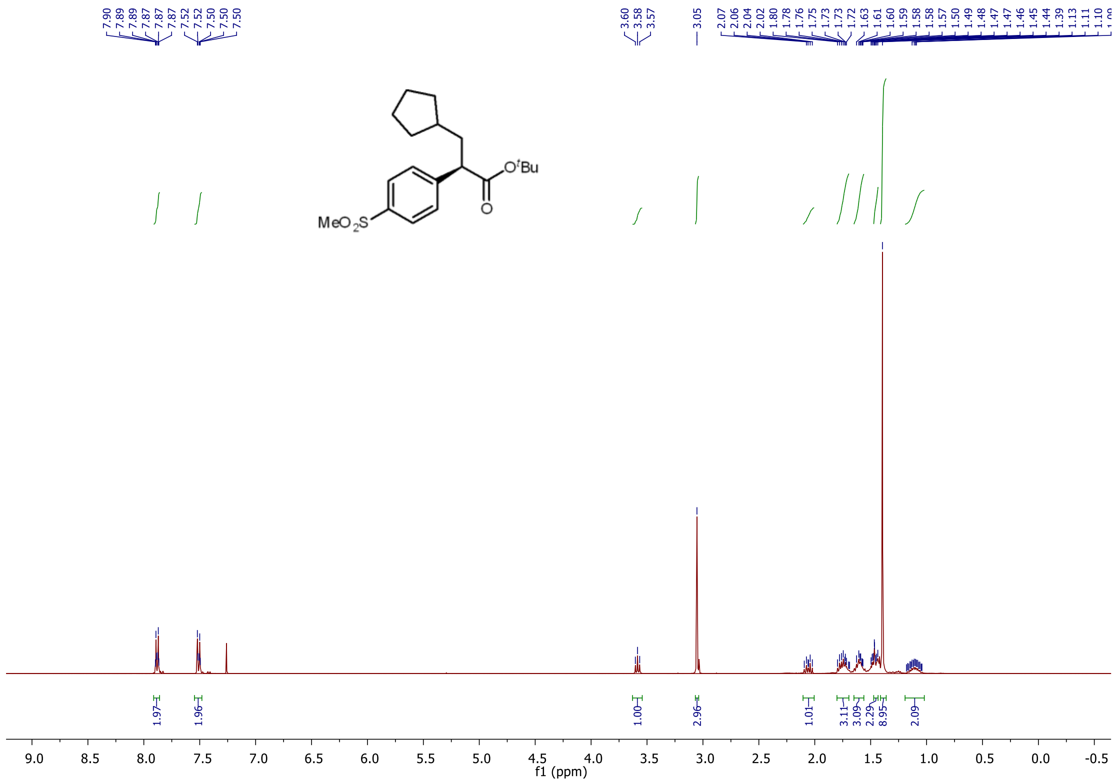

Supplementary Figure 189. ^{1}H NMR of compound **84**.

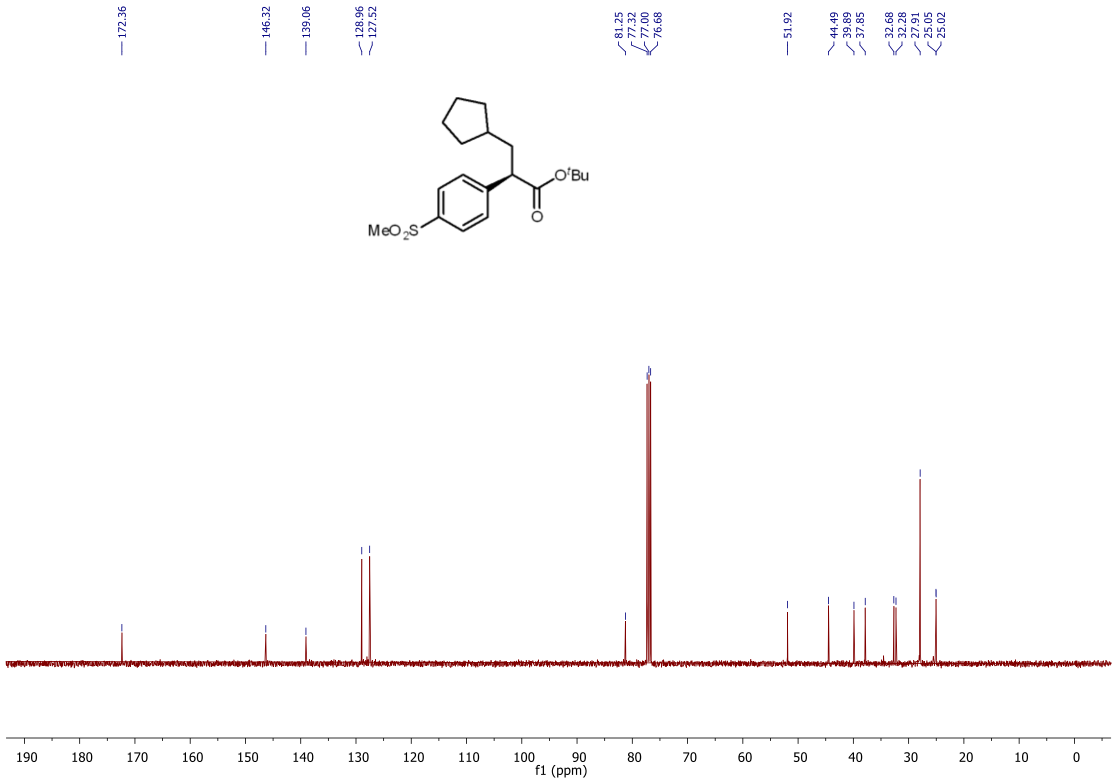

Supplementary Figure 190. ^{13}C NMR of compound **84**.

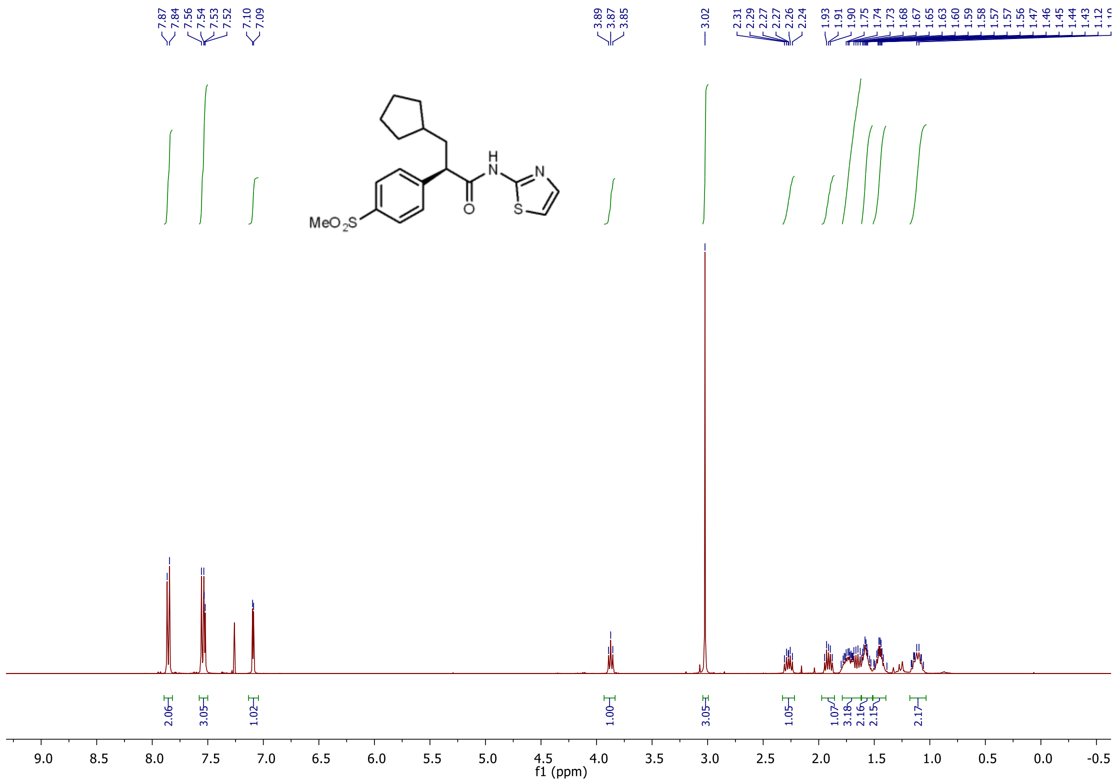

Supplementary Figure 191. ^{1}H NMR of compound **85**.

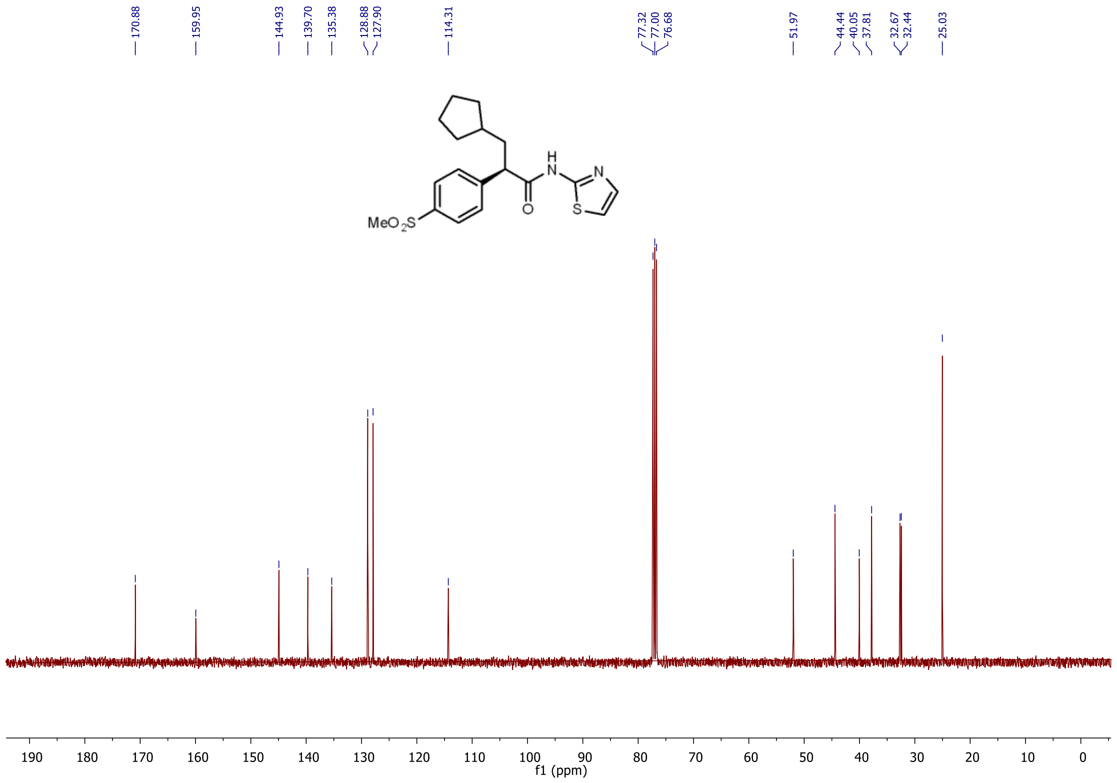

Supplementary Figure 192. ^{13}C NMR of compound **85**.

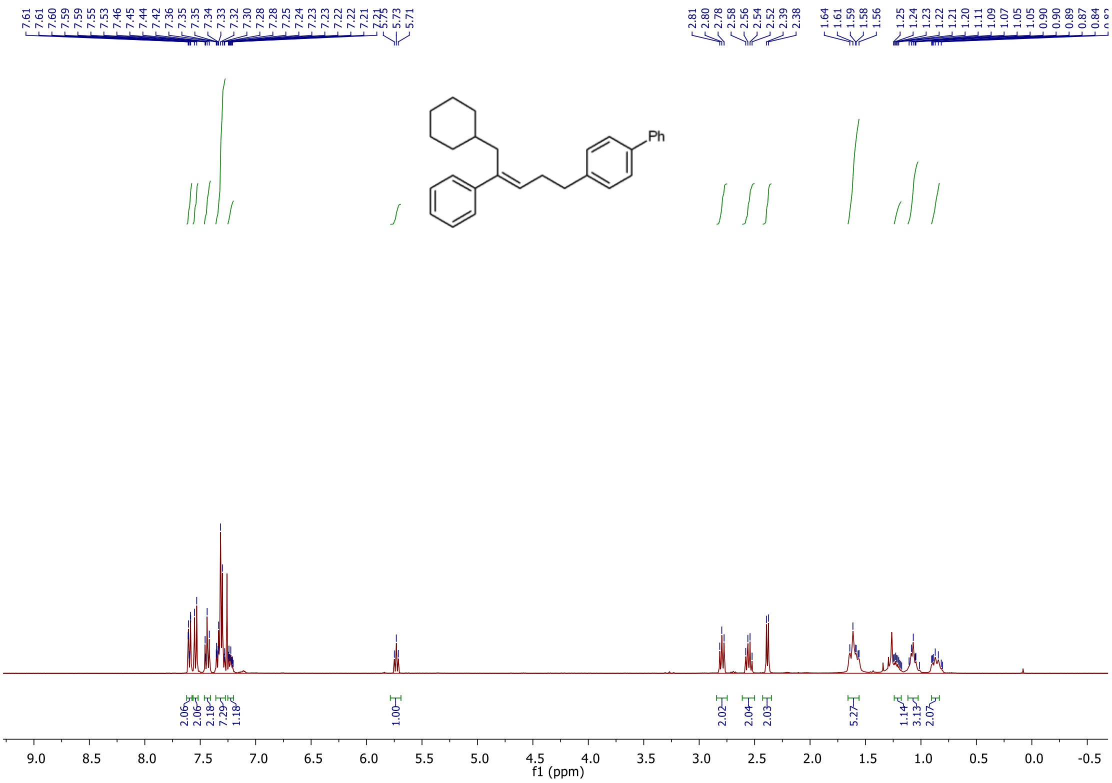

Supplementary Figure 193. ^{1}H NMR of compound **89**.

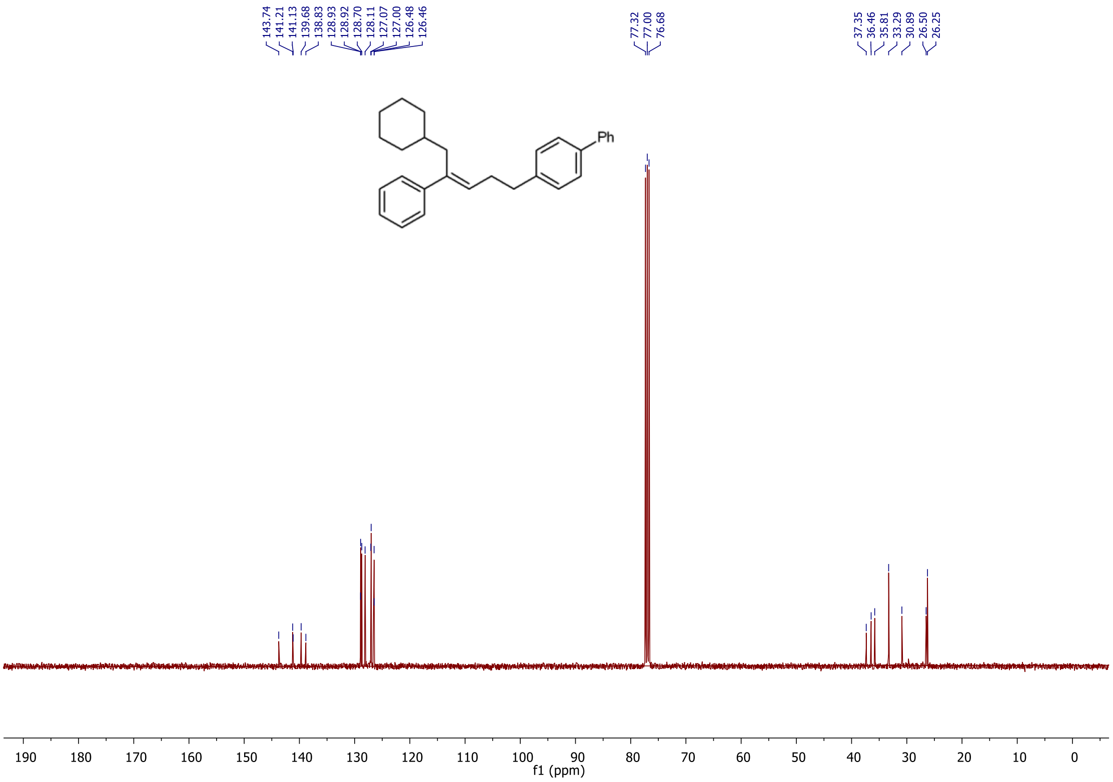

Supplementary Figure 194. ^{13}C NMR of compound **89**.

Supplementary References

1. Shu, X., Zhong, D., Lin, Y., Qin, X., Huo, H. Modular Access to Chiral alpha-(Hetero)aryl Amines via Ni/Photoredox-Catalyzed Enantioselective Cross-Coupling. *J. Am. Chem. Soc.* **144**, 8797-8806 (2022).

2. Espinoza, E. M, Clark, J. A., Soliman, J., Derr, J. B., Morales, M., Vullev, V. I. Practical Aspects of Cyclic Voltammetry: How to Estimate Reduction Potentials When Irreversibility Prevails. *J. Electrochem. Soc.* **166**, 3175-3187 (2019).

3. Clark, R. C.; Reid, J. S. The analytical calculation of absorption in multifaceted crystals. Acta Cryst. A **51**, 887-897 (1995).

4. CrysAlisPro (version 1.171.42.57a), Rigaku Oxford Diffraction Ltd, Yarnton, Oxfordshire, England (2022).

5. Dolomanov, O. V.; Bourhis, L. J.; Gildea, R. J.; Howard, J. A. K.; Puschmann, H. OLEX2: A complete structure solution, refinement and analysis program. J. Appl. Cryst., **42**, 339-341 (2009).

6. Sheldrick, G. M. SHELXT - Integrated space-group and crystal-structure determination. Acta Cryst. A **71**, 3-8 (2015).

7. Sheldrick, G. M. Crystal structure refinement with SHELXL. Acta Cryst. C **71**, 3-8 (2015).

8. Spek, A. L. Structure validation in chemical crystallography. Acta Cryst. D **65**, 148-155 (2009).
