# Supplementary material for: Nickel-catalysed enantioselective alkene dicarbofunctionalization enabled by photochemical aliphatic C–H bond activation
Source: Nat Catal. 2024 Apr 29;7(6):655–65. doi: 10.1038/s41929-024-01153-0 (PMC11208155; doi:10.1038/s41929-024-01153-0)

checkCIF/PLATON report

Structure factors have been supplied for datablock(s) xia2408

THIS REPORT IS FOR GUIDANCE ONLY. IF USED AS PART OF A REVIEW PROCEDURE FOR PUBLICATION, IT SHOULD NOT REPLACE THE EXPERTISE OF AN EXPERIENCED CRYSTALLOGRAPHIC REFEREE.

No syntax errors found.   CIF dictionary   Interpreting this report

Datablock: xia2408

```
Bond precision:   C-C = 0.0019 A          Wavelength=1.54184

Cell:              a=5.56013(5)      b=9.55435(7)      c=34.0101(3)
                   alpha=90          beta=90           gamma=90
Temperature:       160 K

                 Calculated                    Reported
Volume           1806.73(3)                    1806.74(3)
Space group      P 21 21 21                    P 21 21 21
Hall group       P 2ac 2ab                     P 2ac 2ab
Moiety formula   C20 H27 N O2                  C20 H27 N O2
Sum formula      C20 H27 N O2                  C20 H27 N O2
Mr               313.43                        313.42
Dx,g cm-3        1.152                         1.152
Z                4                             4
Mu (mm-1)        0.575                         0.575
F000             680.0                         680.0
F000'            681.87
h,k,lmax         7,12,43                       7,12,43
Nref             3814[ 2247]                   3809
Tmin,Tmax        0.933,0.961                   0.896,0.971
Tmin'            0.876

Correction method= # Reported T Limits: Tmin=0.896 Tmax=0.971
AbsCorr = ANALYTICAL

Data completeness= 1.70/1.00       Theta(max)= 77.295

R(reflections)= 0.0270( 3726)                     wR2(reflections)=
                                                  0.0726( 3809)
S = 1.020                   Npar= 212
```

The following ALERTS were generated. Each ALERT has the format
test-name_ALERT_alert-type_alert-level.
Click on the hyperlinks for more details of the test.

Alert level G

```
PLAT142_ALERT_4_G s.u. on b - Axis Small or Missing .............    0.00007 Ang.
PLAT143_ALERT_4_G s.u. on c - Axis Small or Missing .............    0.00030 Ang.
PLAT791_ALERT_4_G Model has Chirality at C1          (Sohnke SpGr)         R Verify
PLAT912_ALERT_4_G Missing # of FCF Reflections Above STh/L=  0.600         1 Note
PLAT955_ALERT_1_G Reported (CIF) and Actual (FCF) Lmax Differ by .         1 Units
PLAT978_ALERT_2_G Number C-C Bonds with Positive Residual Density.        10 Info
```

0 **ALERT level A** = Most likely a serious problem - resolve or explain
0 **ALERT level B** = A potentially serious problem, consider carefully
0 **ALERT level C** = Check. Ensure it is not caused by an omission or oversight
6 **ALERT level G** = General information/check it is not something unexpected

1 ALERT type 1 CIF construction/syntax error, inconsistent or missing data
1 ALERT type 2 Indicator that the structure model may be wrong or deficient
0 ALERT type 3 Indicator that the structure quality may be low
4 ALERT type 4 Improvement, methodology, query or suggestion
0 ALERT type 5 Informative message, check

It is advisable to attempt to resolve as many as possible of the alerts in all categories. Often the minor alerts point to easily fixed oversights, errors and omissions in your CIF or refinement strategy, so attention to these fine details can be worthwhile. In order to resolve some of the more serious problems it may be necessary to carry out additional measurements or structure refinements. However, the purpose of your study may justify the reported deviations and the more serious of these should normally be commented upon in the discussion or experimental section of a paper or in the "special_details" fields of the CIF. checkCIF was carefully designed to identify outliers and unusual parameters, but every test has its limitations and alerts that are not important in a particular case may appear. Conversely, the absence of alerts does not guarantee there are no aspects of the results needing attention. It is up to the individual to critically assess their own results and, if necessary, seek expert advice.

Publication of your CIF in IUCr journals

A basic structural check has been run on your CIF. These basic checks will be run on all CIFs submitted for publication in IUCr journals (*Acta Crystallographica*, *Journal of Applied Crystallography*, *Journal of Synchrotron Radiation*); however, if you intend to submit to *Acta Crystallographica Section C* or *E* or *IUCrData*, you should make sure that full publication checks are run on the final version of your CIF prior to submission.

Publication of your CIF in other journals

Please refer to the *Notes for Authors* of the relevant journal for any special instructions relating to CIF submission.

PLATON version of 18/05/2022; check.def file version of 17/05/2022

Datablock xia2408 - ellipsoid plot

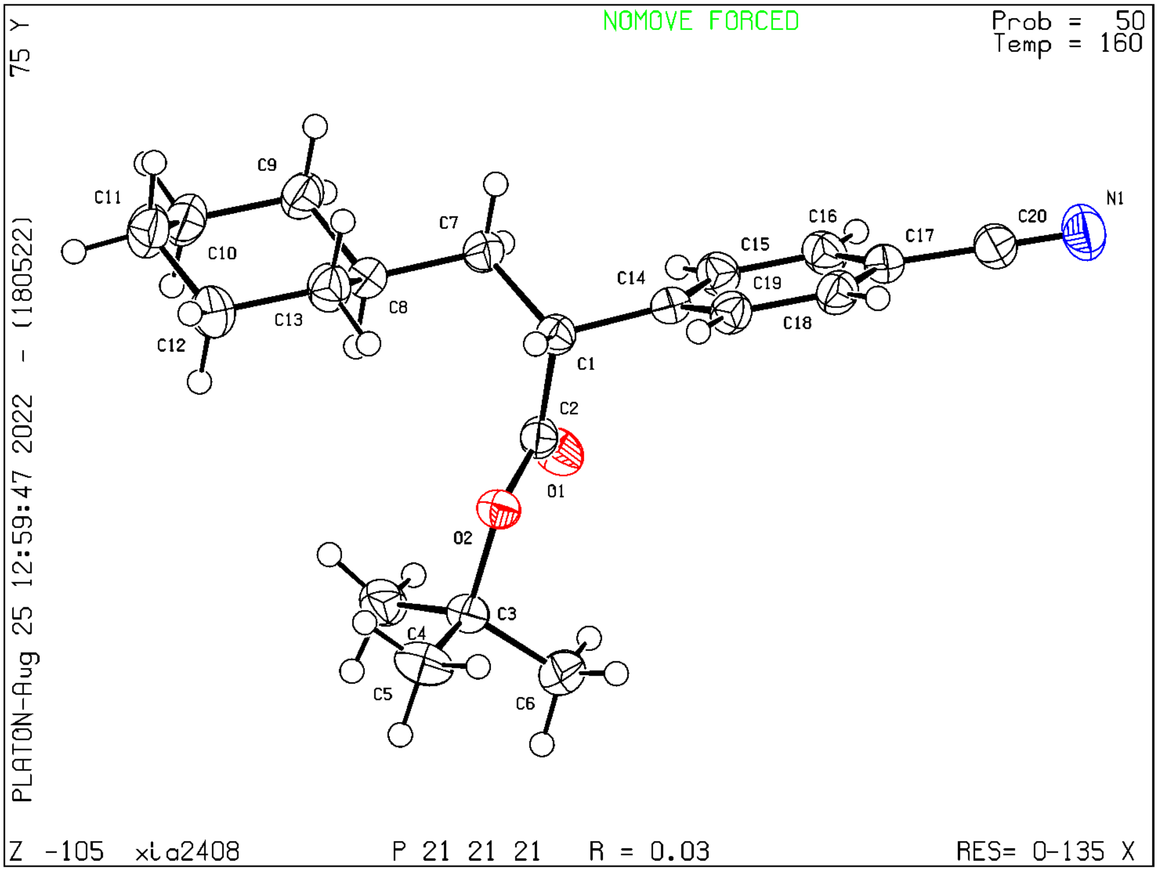

Supplement: Supplementary file 3 — CheckCIF report for compound 1. [file 41929_2024_1153_MOESM3_ESM.pdf]
